# Linear motifs regulating protein secretion, sorting and autophagy in Leishmania parasites are diverged with respect to their host equivalents

**DOI:** 10.1371/journal.pcbi.1011902

**Published:** 2024-02-16

**Authors:** Andras Zeke, Toby J. Gibson, Laszlo Dobson

**Affiliations:** 1 Institute of Molecular Life Sciences, Research Centre for Natural Sciences, Budapest, Hungary; 2 Structural and Computational Biology Unit, European Molecular Biology Laboratory, Heidelberg, Germany; 3 Department of Bioinformatics, Semmelweis University, Budapest, Hungary; Uppsala Universitet, SWEDEN

## Abstract

The pathogenic, tropical *Leishmania* flagellates belong to an early-branching eukaryotic lineage (Kinetoplastida) with several unique features. Unfortunately, they are poorly understood from a molecular biology perspective, making development of mechanistically novel and selective drugs difficult. Here, we explore three functionally critical targeting short linear motif systems as well as their receptors in depth, using a combination of structural modeling, evolutionary sequence divergence and deep learning. Secretory signal peptides, endoplasmic reticulum (ER) retention motifs (KDEL motifs), and autophagy signals (motifs interacting with ATG8 family members) are ancient and essential components of cellular life. Although expected to be conserved amongst the kinetoplastids, we observe that all three systems show a varying degree of divergence from their better studied equivalents in animals, plants, or fungi. We not only describe their behaviour, but also build models that allow the prediction of localization and potential functions for several uncharacterized *Leishmania* proteins. The unusually Ala/Val-rich secretory signal peptides, endoplasmic reticulum resident proteins ending in Asp-Leu-COOH and atypical ATG8-like proteins are all unique molecular features of kinetoplastid parasites. Several of their critical protein-protein interactions could serve as targets of selective antimicrobial agents against Leishmaniasis due to their systematic divergence from the host.

## Author summary

Leishmaniasis is a detrimental disease which causes significant decrease in quality of life and in severe cases can be lethal. The disease is caused by the pathogen *Leishmania* spread by sandfly vectors with vertebrates (including humans) being the primary hosts. Leishmaniasis affects over 10 million people worldwide, with ongoing environmental warming being considered to be helping the disease to spread to new regions. *Leishmania* pathogenesis is currently poorly understood and to combat the infection and to better control the disease further insights into molecular details of the pathogen are needed. Here we describe three protein sorting systems critical for Leishmania cellular housekeeping and infection processes that are greatly diverged from their host counterparts: secretory signal peptides, endoplasmic reticulum retention motifs, and autophagy signals. We provide state-of-the-art computational models to illuminate these systems. Exploring these major cellular sorting motifs may provide new insights into Leishmania cell biology and aid in developing novel therapeutics.

## Introduction

Proper protein trafficking and localization within cells are crucial for maintaining cellular integrity and homeostasis. Eukaryotic organisms carry a largely conserved set of core signaling and sorting systems ensuring the proper execution of critical cellular processes. These not only include the signals for protein secretion (through the Sec61 translocon), but also instructions for the subsequent targeting of secreted proteins into appropriate compartments. Cytoplasmic proteins might also be subject to a broad variety of sorting phenomena, from nuclear import and export to secondary cytoplasm-to-vesicle targeting through autophagy. The receptors involved in these sorting systems typically utilize short, disordered protein motifs, highly conserved among various Eukaryotic crown groups despite their divergence at >1 billion years ago [1–4]. Secretory signal recognition particles (SRPs), endoplasmic reticulum (ER) retaining receptors (KDEL receptors), and autophagy signaling ATG8 proteins are essential components of these processes, contributing to the regulation of protein localization, retrieval, and degradation. While each process has distinct mechanisms and functions, they collectively ensure efficient protein trafficking and cellular quality control.

The signal recognition particle (SRP) is a ribonucleoprotein complex in Eukaryota (as well as Prokarya and Archaea) that plays a vital role in protein targeting and translocation across cellular membranes [5]. By recognizing specific signal sequences on the N-terminus of newly synthesized proteins, the SRP guides them to the Sec61 translocon on the endoplasmic reticulum (ER), allowing the biogenesis of both secreted and transmembrane proteins [6]. Within this complex, the SRP54 protein is responsible for the recognition of N-terminal signal peptides or signal-anchors protruding from the translating ribosome [7]. While signal peptides are co-translationally removed by a dedicated signal peptidase after targeting, the similarly SRP-dependently recognized signal-anchors are inserted into the membrane [8]. Eukaryotic ER-resident proteins with a signal peptide but no transmembrane segment are retained in the lumen by a specific pH-dependent transport system involving the so-called KDEL receptors [9]. These receptors (that obtained their namesake from the Lys/His-Asp–Glu-Leu-COOH sequence they recognize) orchestrate the retrograde transport of target proteins, retrieving proteins containing the C-terminal KDEL motif from the Golgi apparatus back to the ER [10,11]. By maintaining the proper localization of ER-resident chaperones, KDEL receptors are also critical components of cellular quality control and homeostasis [12].

Autophagy is a cellular process responsible for the degradation and recycling of cytoplasmic components. It is regulated by an intricate signaling network involving autophagy-related proteins (ATGs) and key signaling pathways [13]. Through cargo recognition mechanisms, autophagy selectively degrades damaged or unwanted proteins and organelles, promoting cellular renewal and adapting to nutrient deprivation. ATG8, a small ubiquitin-like modifier, is one of the critical core components of this machinery, especially in the case of macroautophagy [14,15]. ATG8 proteins are conjugated to phosphatidylethanolamine lipids by the sequential action of ubiquitin protease-like ATG4 and E2-like ATG3 proteins [16]. Lipidated ATG8 not only directs phagophore membrane formation, but it also serves as a recruiting agent to the autophagosome through multiple linear motifs. Among these motifs, the so-called LIR (LC3 interacting region) motifs are the best known autophagy regulators in animals, fungi and plants [15,17]. These motifs can direct macroautophagy of various cytoplasmic targets, such as damaged mitochondria (mitophagy), peroxisomes (pexophagy), protein aggregates (aggrephagy), glycogen polymers (glycophagy), invading microorganisms (xenophagy), etc [15,18].

These three major molecular systems also represent desirable molecular targets for novel pharmaceuticals. Numerous co-translational secretion modulators (including KZR-8445, HUN-7293 and cotransin) have been identified with therapeutic potential for human diseases. These compounds have the ability to inhibit the production of certain human proteins depending on their signal peptide or transmembrane segment sequence by selectively interfering with their secretion [19,20]. While the ER retaining receptor is yet to be targeted by small molecules, the LIR site of ATG8 proteins has both been successfully targeted by inhibitors [21] as well as cargo-specific autophagy modulators (AUTACs: autophagy targeting chimeras) [22]. Therefore, our findings in *Leishmania* as well as in the broader kinetoplastid group, might also have therapeutic implications.

Leishmaniasis is a neglected tropical disease with cutaneous and systemic forms, caused by unicellular flagellates of the genus *Leishmania*, with a complex life cycle involving insect and mammalian hosts [23]. These protozoan parasites are members of the basal eukaryotic group Kinetoplastida that includes pathogenic *Trypanosoma* species, causing African sleeping sickness as well as the South American Chagas disease. It is now recognized that these dangerous pathogens are part of a much broader group also including free-living species (e.g. *Bodo saltans*) alongside the obligate pathogenic trypanosomatids [24]. Among trypanosomatids, it is now clear that the ability to parasitize mammalian hosts evolved multiple times independently. Hence, the closest genetically studied relatives of *Leishmania* species are the insect parasite *Leptomonas* flagellates (forming the *Leishmaniinae* family or subfamily), and they are only very distantly related to the *Trypanosoma* genus [25,26]. While the heterotrophic kinetoplastids are evolutionary related to the secondarily photosynthetic *Euglena* species, they represent an early branch on the eukaryotic tree of life (crown group Discoba, phylum Euglenozoa): Only the anaerobic metamonads (e.g. *Giardia intestinalis* from the group Fornicata) are thought to have diverged earlier [27]. Kinetoplastids present a whole array of unusual molecular biology features, including a polycistronic genome, with transcripts processed by trans-splicing, as well as a highly complex mitochondrial mRNA editing apparatus [26]. While kinetoplastids appear to possess secretory signal peptides resembling other eukaryotes and bacteria [28,29], as well as a fairly canonical ER physiology and autophagy, we sought to explore if these systems do match with those of higher eukaryotes at a molecular level. During our analysis, we made extensive use of the LeishMANIAdb [30] database developed by our group. Our studies suggest that while these systems have an ancient eukaryotic heritage, they heavily diverged in Kinetoplastids and can be difficult to identify by comparative sequence analysis approaches.

## Results and discussion

### Kinetoplastid signal peptides differ considerably from the usual eukaryotic pattern

**Fig 1:**
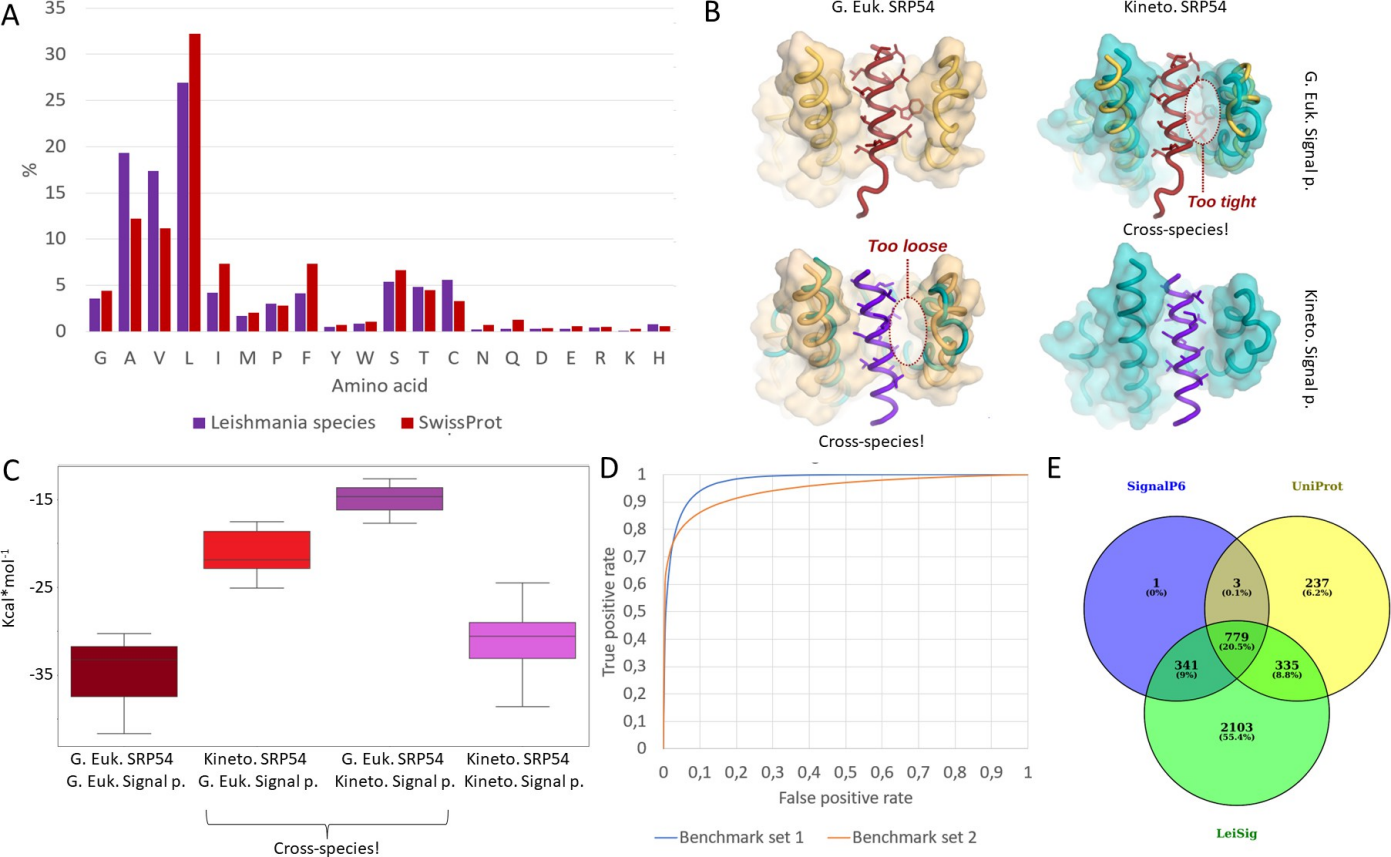
A: Amino acid distribution in the hydrophobic regions of signal peptides. B: left: AF2 predicted structures with different combinations between SRP54 proteins and signal peptides from L. infantum and S. cerevisiae (G. Euk.: Generic Eukaryotic, Kineto: Kinetoplastid, Signal p: Signal peptide). Pdb models used: signal_sacs2_sc.pdb, signal_sacs2_lei.pdb, signal_leiin_sc.pdb. signal_leiin_lei.pdb (structures are available in the S1 Structures). C: Energy (free enthalpy) calculation results from different combinations of SRP54 and signal peptides D: Receiver Operating Characteristics (ROC) of the Leishmania specific signal peptide prediction method (LeiSig) E: Signal peptide predictions on 5 Leishmania species with different approaches.

*Leishmania* species are known to have an unusual way to secrete proteins by utilizing exosomes [31], often considered to be the primary way to export proteins into host cells. However, as in other kinetoplastids, the canonical eukaryotic Sec61-dependent secretion system is also present. The latter is probably indispensable for soluble lysosomal or ER-resident as well as transmembrane protein biogenesis. While the known *Leishmania* signal peptides (SPs) do have a broad similarity to generic eukaryotic signals, they more closely resemble bacterial type II signal peptides in several aspects, such as their amino acid content. For a concise analysis, we collected SPs predicted by SignalP6 [32] in LeishMANIAdb [30] and in SwissProt [33]. When examining their hydrophobic core, we noticed that SPs in *Leishmania* strongly prefer small hydrophobic amino acids, resulting in higher alanine (relative frequency (r.f.): 0.19) and valine (r.f.: 0.17) and lower leucine (r.f.: 0.26) and phenylalanine (r.f.: 0.04) content, compared to other eukaryotes (r.f.: 0.12, 0.11, 0.32, 0.07, respectively; Fig 1/A; S1 Table). Although the difference is not significant in purely statistical terms (using proteome-level statistics), it is still strikingly apparent in many SP examples. The frequency shift seems to be a signature of the Leishmania signal peptides themselves, as the whole proteome amino acid distributions of Leishmania species and SwissProt are quite comparable (S2 Table).

To study the structural background of this unusual finding, we resorted to structural modeling of SRP54 M domains, responsible for the recognition of signal peptides. The only experimentally determined structure was of archaeal origin, whereas most research in eukaryotes has focused on animal and fungal systems. Therefore, we had to model a complex from the animal-fungi-plant crown group for which we chose the yeast *Saccharomyces cerevisiae* SRP54 together with a cognate signal peptide. In the original archaeal complex, three helices (αM1, αM2, αM4) in the M domain recognize and form contacts with a signal peptide. Additionally, αM1b and αMF might also help the recognition by closing on the signal peptide [34] (S1 Fig). Although no eukaryotic complex has been determined yet, homology modeling by Phyre 2 [35] and AlphaFold2 [36] (AF2) could readily be utilized to assess structural differences. Despite the generally similar topologies, Phyre2 struggles with capturing secondary structure elements around the SP, likely because we could only model the monomer and not the bound structure (S2 Fig). We compared the AF2 predicted structure of SRP54 M domain together with a SP in different combinations from *L*. *infantum* and *S*. *cerevisiae*. While cognate complexes including proteins from the same species seem to be compact and do not contain empty volumes or clashes, predicted structures of cross-species combinations are markedly problematic: *S*. *cerevisiae* SRP54 *and L*. *infantum* SP form a too loose structure, while *L*. *infantum* SRP54 and *S*. *cerevisiae* SP contains visible clashes (Fig 1/B).

To follow up our initial observation, we generated sixteen combinations between SRP54 M domains from eight select species (*A*. *thaliana*, *H*. *sapiens*, *S*. *solfataricus*, *B*. *saltans*, *T*. *cruzi*, *T*. *brucei*, *L*. *seymouri*, *L*. *infantum*) with two SP (*L*. *infantum* and *S*. *cerevisiae)* using AF2 (see S1 Structures: signal*.pdb and S3 Table for ptm scores) and calculated the overall stability of each binary structure using FoldX [37]. The rationale behind these calculations is, that kinetoplastid SRP54s might recognize somewhat different signal peptides, thus stability of general eukaryotic SRP54s and signal peptides and kinetoplastid SRP54s and signal peptides should have a lower energy value, while cross combinations (general eukaryotic—kinetoplastid) should have less favorable energetically values. Although relying on yeast signal peptide should slightly affect energy calculations, in this test we only analyze trends and did not expect values to be highly precise. Considering a “generic” eukaryotic SRP54 and *S*. *cerevisiae* SP, their mean stability suggested a reasonable energy threshold for the system (calculated on 3 structures; -35.07 kcal/mol). On the other hand, when we used cross-species combinations (“generic” eukaryotic SRP54s and *L*. *infantum* SP (3 structures), or kinetoplastid SRP54s and generic eukaryotic SP (5 structures)) their calculated binding energy was much lower (-14.79 kcal/mol and -21.20 kcal/mol, respectively) in absolute terms. Importantly, with cognate kinetoplastid SRP54 and *L*. *infantum* SP the calculated mean energy is similar (5 structures; -31.17 kcal/mol) to the other eukaryotic domains with the yeast SP (Fig 1/C and S4 Table).

These calculations suggest that the Leishmania receptor becomes unnaturally strained, with a weaker energy estimate when modeled with general eukaryotic SPs, thus the complex between a "usual eukaryotic" signal peptide and the kinetoplastid receptor was unlikely to form *in vivo*. It was earlier shown that trypanosomatid signal peptides have difficulty targeting recombinant proteins for secretion in other eukaryotes [28]. Note that the yeast peptide also serves as a decent surrogate for animal or plant signals as well. This means that the recognition surface of kinetoplastid SRP45 is unusually narrow, explaining the preference for the shorter Ala/Val side chains. We believe that this also means that the narrower binding groove of the *Leishmania* protein latter might be unique enough to serve as a target for novel small molecule inhibitors.

If the kinetoplastid signal peptides are so different from other, better studied eukaryotic organisms, we wondered if some SP examples might be divergent enough to be missed by classical prediction algorithms. To this aim, we extended the set of known *Leishmania* signal peptides using the homologous information stored in LeishMANIAdb: when SignalP6 predicted a signal peptide (S5 Table), we checked (I) the number of gaps in the signal peptide alignment and (II) the sequence identity of the signal peptides within the protein’s homology group (S6 Table). Using this approach, we collected hundreds of proteins where SignalP6 predicts a signal peptide on some sequences, but not on other closely related ones, even when the sequence identity was strikingly high and there were no gaps in the segment. Altogether we assembled 732 such candidate sequences, from which 411 had lower than 50% gap content and more than 50% sequence identity to SignalP6 signal peptides.

Homology mapping is only helpful when we can reliably assign information between related proteins, however in LeishMANIAdb we have thousands of proteins without experimentally studied homologs. To evaluate whether these proteins might have a signal peptide, we developed a machine learning-based prediction method. First, we created a training set using positive examples from SignalP6 predicted signal peptides (1024 proteins) and the 411 proteins where homology could be used to assign signal peptides. For negative predictions we included membrane proteins (where the first membrane region predicted by CCTOP [38] is within the N-terminal 50 residues—1709 proteins) together with proteins without a predicted membrane region or signal peptide (2212 proteins). The dataset was divided into training and independent test sets (benchmark set 1, S7 and S8 Tables). We also prepared an additional dataset of 89 proteins (by randomly selecting proteins from *Leishmania* proteomes), where we manually checked the presence of signal peptides using deep homology searches (benchmark set 2, S9 Table). Next, we used transfer learning to fine-tune a pre-trained protein language model on the training dataset [39]. On benchmark set 1 we achieved 93% balanced accuracy and 95% Area Under Curve, while on benchmark set 2 we achieved 81% balanced accuracy and 92% Area Under Curve (Fig 1/D; Further details: S10 Table). By setting the cutoff to a higher value (from default 0.5) specificity can be further raised. The developed method (LeiSig) is available for download at https://leishmaniadb.ttk.hu/files/LeiSig.zip. LeiSig takes an amino acid sequence as input, and it predicts whether the Leishmania protein has a signal peptide. Although predicting cleavage sites is still a challenge, for Leishmania species, it is already a huge stride forward to predict likely secreted proteins, potentially narrowing the set of proteins that might play a role in establishing the infection. While our method has clear shortcomings (see Methods), this approach (that confers higher sensitivity (S11 Table) has revealed 3558 candidate proteins with SPs in 5 Leishmania species, from which 2103 were not predicted by other methods (Fig 1/E and S12 and S13 Tables). This suggests that a good number of potentially novel secreted or transmembrane proteins are present in *Leishmania* proteomes.

### The endoplasmic reticulum retaining KDEL motif is unusually divergent in parasitic kinetoplastids

**Fig 2:**
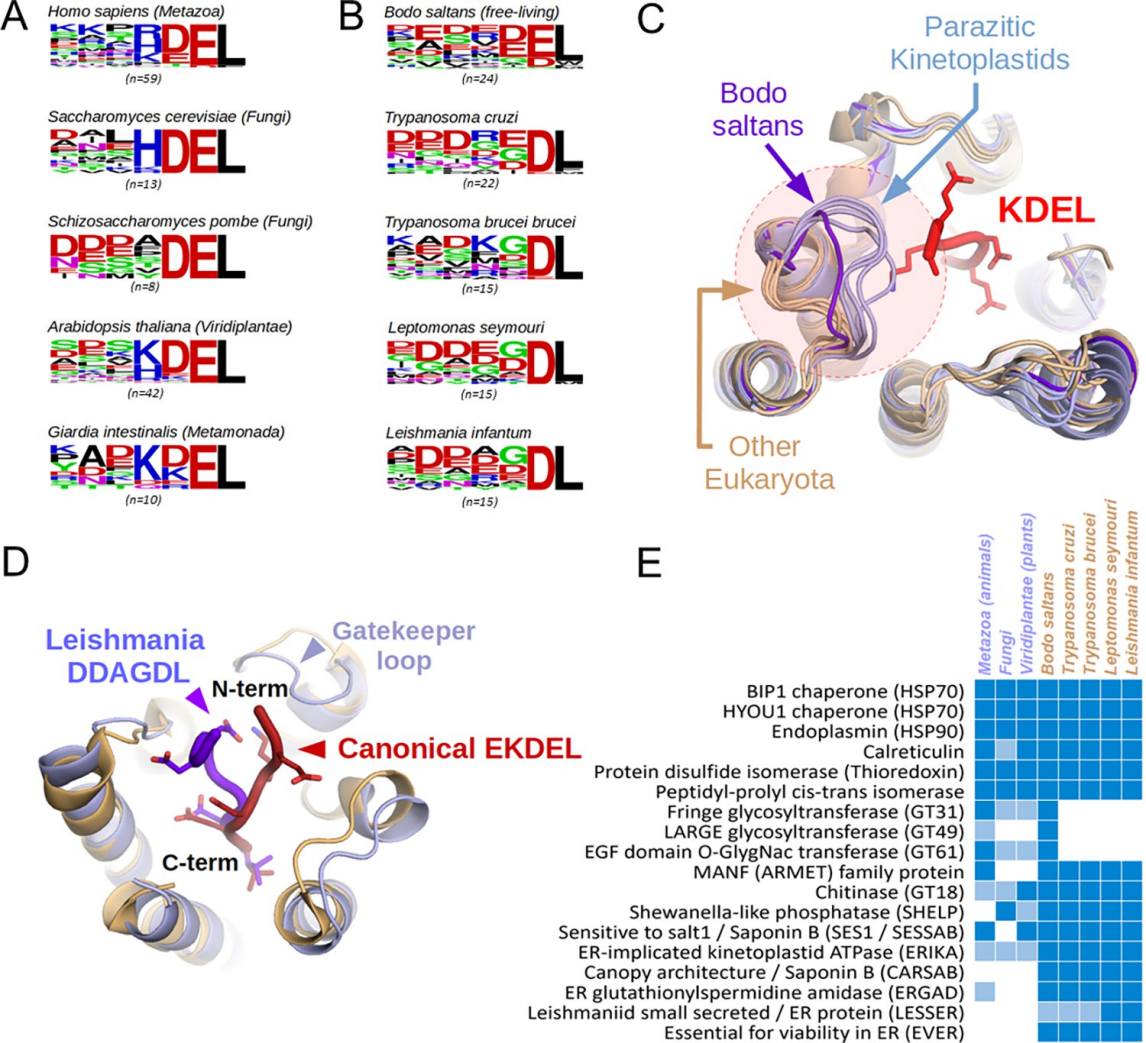
A: Comparison of the sequence logos of all known or predicted KDEL-like motifs of five eukaryotes from different lineages show its conservation. B: This original K/H/RDEL consensus is altered in kinetoplastids, mostly yielding peptides ending in DL. C: Superimposed protein models show that the gatekeeper loop of the ER retrieval receptor (KDEL receptor) of kinetoplastids differs from other eukaryotes, clashing with the main chain of an incoming, canonical KDEL peptide (pdb:6I6H) in case of all examined parasitic species, but not in the free-living Bodo saltans. D: HADDOCK models show that the long gatekeeper loop forces the ligands of the Leishmania receptor to take a different main chain conformation than in the mammalian KDEL receptor. E: Recognizing the altered consensus of receptors allows the identification of conserved families of kinetoplastid ER-resident proteins including newly-identified protein groups (Blue bars show the presence of KDEL-like peptide-containing orthologs, while light blue bars indicate homologous proteins without KDEL).

After the signal peptides, we turned our attention to another unusual feature of *Leishmania*. The group of constitutively endoplasmic reticulum (ER) lumenal proteins encompasses many highly conserved proteins with roles in secreted protein folding, glycosylation, and quality control. In all known Eukaryota, these proteins are constitutively sorted to the ER with the help of a retrograde transport system from the Golgi apparatus, utilizing a single receptor (also known as the KDEL receptor). However, the usual, highly conserved (H/K)DEL consensus at the C-termini of these proteins, that is strongly conserved in the animals, plants and fungi crown group (Fig 2/A) cannot be found in proteins of *Leishmania* species. Instead, careful examination shows that the conserved, known or predicted ER resident *Leishmania* proteins appear to always carry a carboxy-terminal DL motif (Fig 2/B). This consensus differs from all other known eukaryotic organisms outside Kinetoplastida, including the early-branching flagellate *Giardia intestinalis*, but matches with other kinetoplastid species.

A close examination of the AlphaFold2-predicted structures for the ER-retaining KDEL receptor helps to explain this discrepancy. Although the base of the receptor binding cleft has almost 100% identity in terms of the amino acids lining the receptor cavity, the entrance to the KDEL receptor in *Leishmania* is narrowed by an elongated loop, leading to a clash when superimposed on animal KDEL receptor-ligand crystal structures (Fig 2/C). This forces the peptide chain in *Leishmania* species to adopt a substantially different geometry when entering the receptor. To demonstrate this phenomenon, we built a model of *Leishmania infantum* KDEL receptor based on the AlphaFold2 structure with a peptide ligand (using the optimal consensus sequence), docked and optimized its geometry with HADDOCK [40] (see Methods). Comparison with the crystal structure of an animal KDEL receptor complex clearly shows the different main chain geometry (Fig 2/D).

Interestingly, this deviation from the classical patterns is not restricted to *Leishmania* species only. The same inwardly oriented "gatekeeper" loop features in the KDEL receptor are also seen in other kinetoplastid species, such as *Trypanosoma brucei brucei or Trypanosoma cruzi*. However, due to a difference in gatekeeper amino acids, the resulting KxDL-like motif is slightly closer to the canonical one in the Trypanosoma genus than in *Leishmania sp*. or any other kinetoplastids (see S14 Table). Curiously, we found that the aberrant DL-preference (Asp-Glu-COOH) at the C-terminus is restricted to the obligately parasitic Trypanosomatidae. A survey of the motifs in the free-living kinetoplastid *Bodo saltans* shows that it still prefers the canonical KDEL motif, although with a relatively relaxed variation of individual residue positions. In this early-branching species, the elongated ‘gatekeeper’ is directed outwards, leading to a broadly open cavity, presumably with relatively permissive ligand binding properties. It is the gradual alteration of this "gatekeeper" loop that led to a restricted, but non-canonical KDEL-like DL motif in parasites. While this retention motif differs considerably from their host organisms, it is unclear if it is truly orthogonal to the ER receptor of their (animal or plant) host. Therefore, the biological role of this unusual divergence (if any) is currently unclear.

A more extensive analysis of KDEL receptors and their ligand motifs captures this evolution even more precisely (see the evolutionary trees and sequences on S3 Fig, involving 12 extra species in addition to the ones shown on Fig 2). It includes several trypanosomatids as well as *Neobodo designis*, *Perkinsela sp*p. (non-parasitic kinetoplastids), *Eutreptiella gymnastica* (Euglenida) and *Naegleria gruberi* (Heterolobosea, crown group Discoba) as outgroups, for a total of n = 17 species (S15 Table). Despite the relatively poor conservation of the gatekeeper loop sequence (and even its exact length), the evolutionary trajectory appears clear. Most AlphaFold2 models within Trypanosomatidae display inward positioned gatekeeper loops (except for *Strigomonas culicis* and *Phytomonas sp*. EM1). By contrast, all the models of other organisms show outwardly positioned loops. Even if AlphaFold2 is not 100% correct at generating the most favourable conformation of flexible loops (having low pLDDT values), this is very well in-line with the assumption that the exclusively parasitic trypanosomatids have a preferentially inward-facing conformation, while the same gatekeeper loop is always outward-facing for other species (the non-parasitic kinetoplastids *Bodo saltans*, *Neobodo designis*, *Perkinsela spp*. And the euglenid *Eutreptiella gymnastica*) (S4 and S5 Figs). The set of inwardly facing amino acids (especially an Arg) severely restricts the space within the receptor, forcing the ligand motifs to adopt a different main chain geometry together with a different sequence preference. A specific Pro within the loop also appears to stabilize this inward geometry in most species. These alignments also reveal the probable cause of the slight Arg/Lys preference at the -4 position of KDEL for the Trypanosoma genus. In their case, a Glu->Asp exchange immediately below the gatekeeper loop enlarges the cavity of the receptor, permitting the canonical Arg/Lys residues to be present in this motif (S6 Fig). The elongated “gatekeeper” loop of the KDEL receptor is a feature unique to the phylum Euglenozoa, an evolutionary innovation not seen in other eukaryotes. The latter is supported by the observation that Naegleria amoebae (belonging to another branch of Discoba) still show perfectly canonical receptors with short loops and canonical K/HDEL motifs. This evolutionary path of receptors correlates exquisitely with the motif logos, showing conserved EL motif endings in Euglenida (widely outwards loop), mixed EL/DL endings in non-parasitic kinetoplastids (loop still moderately outwards), and almost exclusively DL endings among members of the obligate parasitic Trypanosomatidae (mostly inward-positioned loops) (S3 Fig).

The redefined motif regular expression together with the newly developed signal prediction method enabled us to scan species from LeishMANIAdb for candidate ER proteins (S16 Table). To further limit possible false positive hits, we also supplied information about intrinsic protein disorder and cellular localization. We also supplied the position-specific scoring matrix calculated by PSSMSearch [41] to perform a more sensitive scan (S17 Table). Recognizing the correct KDEL-equivalent motif in Leishmania species, we not only managed to confirm conservation of well-known ER-resident proteins: Hsp70 (both the BIP and HYOU1 families) and Hsp90 (endoplasmin) chaperones, calreticulin, protein-disulfide isomerases and peptidyl-prolyl isomerases in *Leishmania* species, but also several other, unusual proteins (Fig 2/E and S14 Table). We located an ER-resident chitinase in *Leishmaniinae*, with a conserved KDEL-like signal, that is also found in a few other eukaryotes, though not in animals. The relatively poorly known MANF (Mesencephalic astrocyte-derived neurotrophic factor, also known as ARMET) proteins that have previously only been identified in animals, might be ancient eukaryotic chaperones [42]. We uncovered more than one family of Saponin B domain containing proteins (putative chaperones), one that is clearly homologous to the Arabidopsis sensitive to salt (SES1) protein (SESSAB) [43][44], and a second example showing some architectural similarity to the Canopy proteins found in animals and plants (CARSAB) [45]. We also confidently identified a kinetoplastid *Shewanella*-like phosphatase (SHELP), with homologous ER retention signals already found in euglenids, hinting at an ancient origin, even if their function is currently unclear [46].

Notably, kinetoplastids also seem to have paralogous pairs of some enzymes, with one protein lineage consistently bearing a KDEL-equivalent signal (in addition to a signal peptide), and a second lineage pair that does not. This is the case with torsin ER-implicated kinetoplastid ATPases (ERIKA; animal torsins have no such motifs) [47], as well as with the better-known trypanothione synthases. In the latter case, the ER-resident glutathionylspermidine amidase domain protein (ERGAD), carrying only one domain, might have a physiological role that is different from its’ better-known, cytosolic paralog (with two catalytic domains) involved in trypanothione metabolism [48].

To assess the functionality of these newly-described proteins, we also examined the predicted catalytic site of kinetoplastid ER-retained chitinase, glutathionylspermidine amidase domain (ERGAD), ERIKA and Shewanella-like phosphatase (SHELP) proteins using sequence alignments and AlphaFold2 models (S7, S8, S9 and S10 Fig, respectively). Interestingly, apart from ERGAD, where the single hydrolase domain consistently lacks a nucleophilic residue necessary for catalysis, all the others are expected to be catalytically active. This is especially interesting in the case of the predicted glycohydrolase 18 (chitinase) enzyme, as it is unclear what substrate(s) it processes within the endoplasmic reticulum. However, non-kinetoplastid euglenids and some other unicellular eukaryotes (e.g. green algae) also possess a homologous ER-resident protein (with a catalytic site compatible to beta-1-4 N-acetyl-glucosamine oligosaccharides, and bearing none of the mutations observed in inactive chitinases), hinting at its involvement in a hitherto unknown biochemical pathway.

Finally, there are examples with protein families consistently bearing an ER retention signal (in addition to the signal peptide) in *Leishmania* species as well as their relatives, but without any known homology to proteins in other eukaryotes. One such protein (LESSER), with a very small domain, has an ER retention signal in *Leishmaniinae*, but appears to be secreted in other kinetoplastids. Another, even more intriguing kinetoplastid-specific protein (EVER) has recently been identified to be essential for viability in *L. donovani [49].* Although originally described as a secreted protein (due to the signal peptide), we now confidently predict it to be an ER component. The fact that *Leishmania* species (as well as other parasitic trypanosomatids) seem to have an overall reduced inventory of ER-resident enzymes compared to free living *Bodo saltans* might have therapeutic relevance. Given these gene losses, the retained ER-resident proteins might have functions that are important for the *Leishmania* life-cycle, potentially providing drug targets due to their uniqueness or divergence from homologous host proteins.

### The identification of LIR-like motifs reveals a fast-evolving autophagy network in Leishmaniids

ATG8 proteins are small ubiquitin-like modifiers essential for macroautophagy in all known eukaryotic organisms [50]. Unlike ubiquitin, they are covalently (but reversibly) attached to the membrane lipid phosphatidylethanolamine (by ATG4 enzymes), thereby directing the formation of the phagophore membrane. The location, extent and cellular contents engulfed by the autophagosome are dictated by ATG8-interacting proteins that typically bind ATG8 through the so-called LIR motifs in both animals, fungi, and plants. Although minor variations exist (e.g. in animals, ATG8 proteins have split into LC3 and GABARAP subfamilies, with slightly different LIR motif ligand preferences) [51], this essential linear motif is largely conserved across known eukaryotes with only mild alterations.

**Fig 3:**
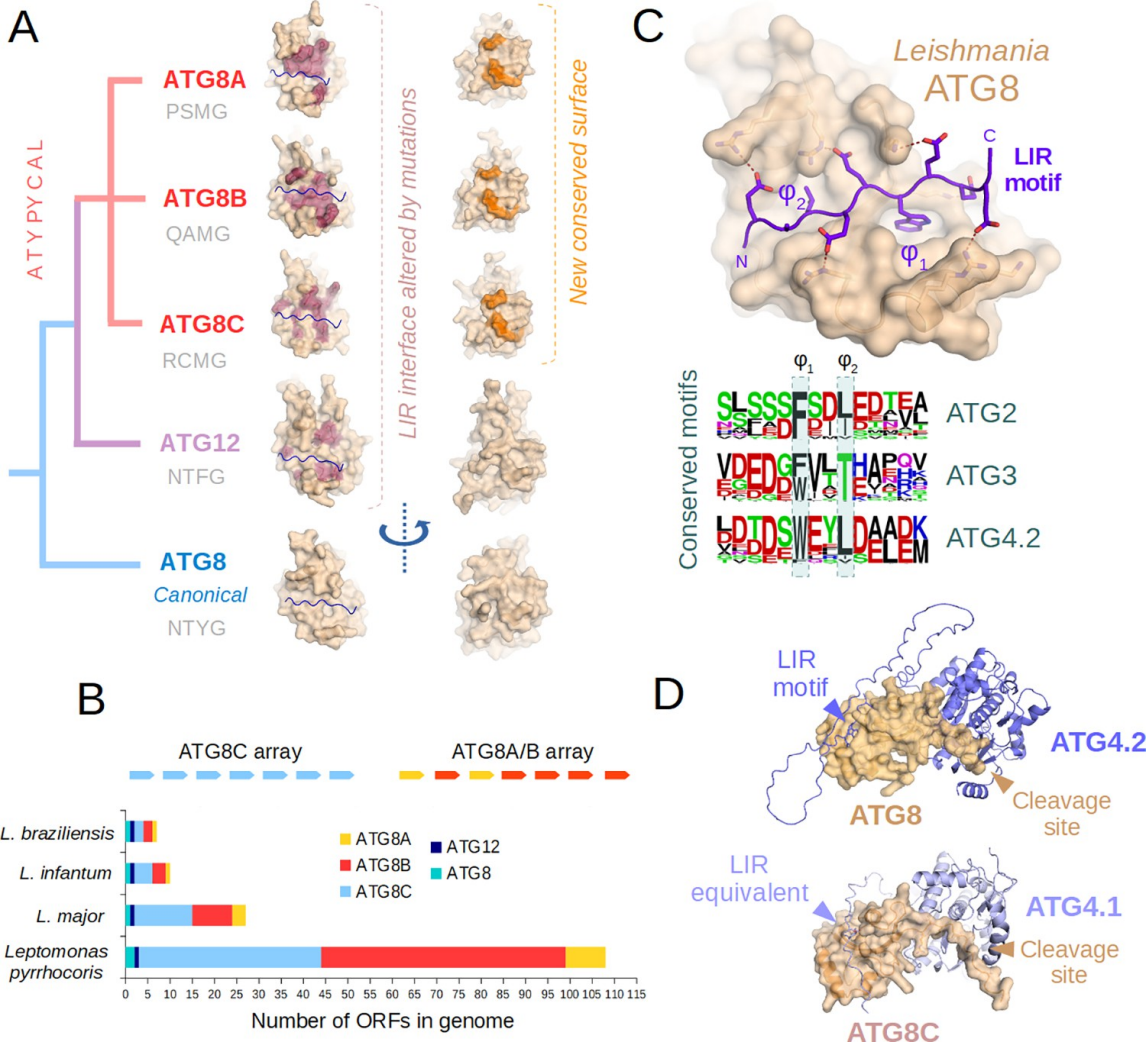
A: Our evolutionary model of ATG8 protein groups in Leishmaniinae. For each paralog, the surface mutations expected to block LIR binding are shown in red, while the novel invariant surface is in orange. The small structural figures show matching protein surfaces from the same angle. Predicted ATG4 cleavage sites (P-4 to P-1) for each protein group are exemplified by a sequence written in gray below each group name. B: The ATG8A, ATG8B and ATG8C genes are typically found in two tandem arrays within leishmanial genomes but their copy numbers vary highly across species. C: The preserved LIR peptide-binding ability of ATG8 proteins is illustrated by a simulated model between this protein and a Leishmania LIR motif model peptide, similar to the ones seen in conserved autophagy apparatus proteins ATG2, ATG3 and ATG4 (frequency logos from proteins in group Discoba written below). D: The simulated complexes between ATG8 and the enzyme ATG4.2 and between ATG8C and ATG4.2 are highly similar and betray a potential existence of a LIR-equivalent motif for atypical ATG8s.

As already noted by previous studies, *Leishmania* and *Leptomonas* species have proliferated their ancestral ATG8 / ATG12 proteins into a multitude of paralogs [52], with a complicated nomenclature, but major differences were shown in other autophagy related systems too [53]. Our careful, detailed analysis of kinetoplastid ATG8 evolutionary trees shows that there are five different, major families of ATG8-like proteins in *Leishmania* species [54]. The previously identified ATG8A, ATG8B and ATG8C subgroups are highly divergent and atypical in terms of sequence and structure. We observed that the previously annotated *Leishmania* ATG12 gene has a sibling evolutionary relationship to divergent *Leishmania* ATG8A, ATG8B and ATG8C proteins, and these groups all seem to have evolved from a single ATG12-like gene also found in other trypanosomatids (S11 Fig). Unfortunately, it cannot be decided from the scarce available data whether the kinetoplastid ATG12 would biochemically more resemble canonical eukaryotic ATG8 or ATG12 in function. ATG8 and ATG12 proteins share strong structural and sequence homology, having evolved from the same ancestral ubiquitin-like modifier [55]. Notably, the kinetoplastid ATG12 proteins require cleavage by proteases, similarly to ATG8s (processed by ATG4 enzymes) and unlike other eukaryotic ATG12s [54]. Finally, the ATG8 gene and protein of *Leishmania* species is fairly canonical, with a surface highly similar to other eukaryotic ATG8 proteins. A careful comparison of surfaces shows that the LIR binding surface has been severely altered in ATG8A, B, C and ATG12 proteins (Fig 3/A). Therefore, we assume that the latter proteins are incapable of binding the canonical LIR motifs and that these motifs can only target ATG8.

As a further striking finding, neither the kinetoplastid ATG12s, nor any of the atypical ATG8-family proteins have a LIR interface compatible with the AIM (also known as AIM12) linear motif found in ATG3. The latter motif is crucial for ATG12-mediated ATG8 conjugation in the majority of known eukaryotic organisms, allowing ATG12 conjugates to act upstream of ATG8 [56–58]. The nearly-ubiquitous AIM12 motif, found in animals, plants and fungi, is not even conserved in kinetoplastids (although our ATG3 alignments betray that it is present in other groups within Discoba, suggesting a secondary loss, see S14 Fig). Hence the role of kinetoplastid ATG12 in (macro)autophagy and ATG8 deployment is currently unclear.

While neither the ATG8A, ATG8B or ATG8C proteins appear to be capable of canonical LIR motif binding (as the interaction interface is not conserved compared to ATG8), they do have other putative protein-protein interaction surfaces (Fig 3/A). A careful surface analysis of atypical ATG8-like proteins reveals that while little is preserved across all of them, each of the ATG8A, ATG8B and ATG8C proteins have two potential protein-protein interaction surfaces that are conserved within each subtype (see S12 Fig). One of them is the heavily altered LIR interface that is nevertheless likely able to recruit motifs radically different from canonical LIRs. The other region that is also reasonably conserved across these atypical ATG8s falls fairly close to the so-called UIM (ubiquitin interacting motif) binding surface of ATG8 proteins found in other eukaryotes [59]. Thus, it is quite likely that the latter interface is also functional, although its partners are currently unknown. Notably, most ATG8A, ATG8B or ATG8C *Leishmania* proteins present two cysteine amino acids adjacent to each other at this region, suggesting a possible redox-dependent regulation. Interestingly, each of these proteins preferably conserve either the altered LIR site (ATG8A, ATG8C) or the latter, novel site (ATG8B) instead, hinting at some subtle functional divergence among them (S12 Fig).

As evidence of recent diversification, most atypical ATG8 genes are found as part of large gene arrays in *Leishmaniinae*. ATG8C genes form an array of their own, while ATG8A/B genes form joint arrays at a different genomic location, with a lot of identical or highly similar genes (Fig 3/B and S18 and S19 Tables). Most of these arrays also contain fragmentary, incomplete ATG8-like ORFs, that nevertheless might contribute to recombination between related genes. This rampant gene amplification is clearly not due to adaptation to mammalian hosts, as matching arrays are already seen in the insect parasite *Leptomonas pyrrhocoris*. Still, the ATG8-like gene copy number increases at each separate cluster are remarkably consistent across species. Interestingly, ATG12 genes do not seem to be subject to gene amplification, nor are the canonical ATG8 genes (Fig 3/B).

To test the functionality of the LIR interface of canonical ATG8 proteins, we searched for homologs of key proteins involved in autophagy in other eukaryotes (*H*. *sapiens*, *S*. *cerevisiae*, *A*. *thaliana*). While the homologs of many proteins are missing from kinetoplastid genomes entirely, we managed to find three examples, where the LIR motif has also been preserved during evolution (S13 and S15 Figs). Since AlphaFold2 multimer has proven rather successful in predicting the correct geometry of LIR motifs [60] and other ATG8 partners, we applied it to *Leishmania* complexes as well. ATG3 is the E2-like conjugating enzyme involved in transferring the activated ATG8 from ATG7 (E1-like) to the target lipids. As in most other eukaryotes (save certain fungi), the internal loop of ATG3 carries an atypical LIR motif, also conserved in *Leishmania*, as our alignments and AlphaFold2 models show (S14 Fig). A conserved, canonical LIR motif is also found at the disordered C-terminus of the *Leishmania* ATG4.2 enzyme (S15 Fig). ATG4s are ubiquitin hydrolase-like enzymes involved in ATG8 pre-processing (interestingly the other homolog, ATG4.1 consistently lacks a LIR motif, and the motif of ATG4.2 has also been altered in certain other kinetoplastids). Finally, the lipid transfer protein ATG2 also seems to be present in kinetoplastids, with a canonical LIR motif at an identical position (on a predicted disordered loop) as seen in animals. (S13 Fig). The sequence logos for each protein alignment as well as a further, idealized model LIR peptide complex with *Leishmania infantum* ATG8 is shown on Fig 3/C.

Encouraged by the conservation of the ATG8 surface, we set out to identify potential LIR motif-containing, autophagy-related proteins in *Leishmania* genomes (Table 1, also see S20 and S21 Tables for more details). Taking the conserved instances also into consideration, the simplest regular expression of kinetoplastid LIR motifs would be [DE].{0,2}[WFY]..[LIV] or [DEST].{0,2}[WFY]..[LIV], also allowing phosphorylation to provide the charges to the termini, as commonly seen in many eukaryotes. However, this fails to take into account that the inner positions strongly select against structure breaking (Pro, Gly) or positively charged (Arg, Lys) amino acids, and also disallow aromatics (Phe, Tyr, Trp, His) at the first intervening position [1,15]. The flanking regions, especially the N-terminal flank, also strongly disfavour positively charged residues. Motifs that contain Tyr at the first hydrophobic position (Y-LIR) are rare, owing to the property that they bind more weakly than their F-LIR or W-LIR counterparts. Hence a better, stricter definition that retrieves motif candidates that are more likely to be functional, would be (allowing the C-flank to take the same anchoring role as the N-flank, as it is also seen in a number of eukaryotic LIR motifs, e.g. vertebrate Beclin-1): [^RK][^RK][DE][WF][^PGRKFYWH][^PGRK][LIV] | [^RK][DE][^RK][WF][^PGRKFYWH][^PGRK][LIV] | [DE][^RK][^RK][WF][^PGRKFYWH][^PGRK][LIV] | [^RK][^RK][^RK][WF][^PGRKFYWH][^PGRK][LIV][^RK]{0,1}[DE]. This latter definition covers almost all canonical, conserved LIR motifs observed among kinetoplastids and their relatives (crown group Discoba) with only sporadic exceptions (and the atypical ATG3 motif only differs from this by using Thr at the second hydrophobic pocket). After assessing this definition on a broad collection of experimentally tested, canonical LIR motifs (from *H*. *sapiens*, *S*. *cerevisiae* and *A*. *thaliana*) featured on LIRcentral [61], we found that this strict definition still recovers 48% of all known functional LIR motifs (and all core autophagy apparatus motifs), while rejecting 78% of non-functional ones (S20 Table). However, even with these restrictions, the number of motif hits in Leishmania proteomes were still very large. Therefore, we only analyzed a small number of hand-picked examples, where the molecular architecture or evolutionary context hinted at possibly functional motifs (shown on Tables 1 and S21).

**Table 1:** Select Leishmania infantum proteins with likely functional LIR motifs and the evolutionary conservation of the motifs. Motif cores are bolded and underlined.

*UniProt accession* *(L*. *infantum)*	*Suggested* *protein name*	*Motif* *sequence(s)*	*Taxonomic* *range*
*A4IBB7_LEIIN*	*ATG2*	*SLSSSFSDLEDTVA*	*Eukaryota*
*A4I8P6_LEIIN*	*ATG3*	*VDEDDFVLTEATQV*	*Eukaryota (atypical motif)*
*A4I521_LEIIN*	*ATG4*.*2*	*VDTDSWEYLD—-*	*Eukaryota (save certain kinetoplastids)*
*A4HYW1_LEIIN*	*Inactive calpain protease*	*NDEAQWTEIEEEAP*	*Leishmaniinae*
*A4I2N5_LEIIN*	*Inactive calpain protease*	*DEDAEFVALEDEWR* *(33 repeats)* *DEDAEFLALEDEWR* *(10 repeats)*	*Leishmaniinae*
*A4I049_LEIIN*	*Neurobeachin-related protein*	*EEEEQWEVLPDPTT*	*Leishmaniinae*
*A4HWW7_LEIIN*	*Tandem LysM domain protein*	*QRLQEWSELDDPLE*	*Leishmaniinae*
*A4IA45_LEIIN*	*Leishmaniid-specific disordered protein*	*YRTPQWTELSECIR* *APAETWQALSERQR*	*Leishmaniinae*
*A4IE26_LEIIN*	*Mitochondrial fission protein FIS1*	*RVDDEWVDIFGSPA*	*Leishmaniinae*
*E9AGR1_LEIIN*	*DnaJ (HSP40) chaperone*	*ITQAEWSELVERHE*	*Kinetoplastida*
*A4HZ48_LEIIN*	*DnaJ (HSP40) chaperone*	*AEEDEFEDVTDDDD*	*Leishmaniinae*
*A4I0Q3_LEIIN*	*DeSI-family deubiquitinase (active)*	*EPGNEFSDITGALV*	*Leishmaniinae*

Calpain-like proteases are a large and diverse, but enigmatic protein family in *Leishmania*, where only a portion of proteins preserve the proteolytic functions [62]. Intriguingly, we found that many of these calpain-like proteins carry LIR motifs (both F-LIR and W-LIR subtypes), even including motif multiplets. For the first time, this suggests a potentially important physiological function of *Leishmania* calpain-like proteins in autophagy. Notably, none of our LIR motif-bearing calpain examples have an intact, conserved catalytic site, therefore their most likely purpose lies in mediating protein-protein interactions. We also identified LIR motifs in *Leishmania* Neurobeachin/ALFY and FIS1 orthologs (both protein families are involved in autophagy in animals) [63,64]. Other LIR instances also show some intrinsic logic, such as the presence of such motifs together with LysM domains. The latter protein domain is a glycan-sensing module (some LysM domains bind chitin, others peptidoglycan) involved in detection and clearance of invading organisms in other eukaryotes (as a cytoplasmic sensor), although its ligand specificity in Leishmania is unclear [65]. Another interesting finding is the frequent presence of putative LIR motifs on DnaJ chaperones, a protein family highly expanded in Leishmania species [66]. DnaJ (HSP40) proteins are known to be involved in chaperone-mediated autophagy [67] therefore at least some of these motifs might have functional significance. Intriguingly, most of the LIR motif instances we identified had a very shallow ancestry. These motifs were rarely conserved beyond the *Leishmania + Leptomonas* (*Leishmaniinae*) group, despite the overall good surface conservation of ATG8. Thus, we assume the *Leishmania* autophagy network might have been rewired extensively and relatively recently in terms of evolution.

Out of the conserved autophagy regulators, ATG4.2 is also interesting for a number of reasons. The presence of a LIR motif on the extreme C-terminus of ATG4 is almost warranted, as it is also found there in many other eukaryotes. Interestingly, all *Leishmania* relatives possess not one, but two clearly distinguishable ATG4 paralogs (ATG4.1 and ATG4.2), where only ATG4.2 has a LIR motif. *Leishmania* ATG4.1 and ATG4.2 were suggested to selectively process different ATG8-like proteins in the past. However, no evolutionarily or structurally consistent pattern has been reported [54]. Based on structural and evolutionary arguments alone, we speculate that the canonical *Leishmania* ATG8 is probably processed by the matching LIR-motif containing ATG4.2 in cells. This would leave ATG4.1 to be the main protease responsible for the activation of ATG8B, ATG8C and possibly ATG8A, that all have a similar cleavage site, but different from that of ATG8. To corroborate this theory, we used AlphaFold2 multimer to build a complete structure of the hypothetical complex between ATG4.2 and ATG8. This complex (Fig 3/D) suggests excellent structural compatibility between partners, a perfectly fit cleavage site, complete with a LIR motif from the C-terminus docked into ATG8. We also had the potential complex of ATG4.1 and ATG8C modeled, and this gave a surprising suggestion: The C-terminus of ATG4.1 has a sequence very different from canonical LIR motifs, and yet it has the potential to interact with the atypical ATG8 relatives in a similar manner. Unfortunately, we cannot predict these LIR-equivalent linear motifs yet, requiring at least one example to be experimentally validated first. In the light of specialization of ATG8 and/or ATG4 paralogs seen in other eukaryotes, we cannot help but speculate that similar phenomena happen in Leishmania parasites [68,69].

Out of the three biochemical systems studied in the current article, the ATG8 system is the most complex, yet least amenable to antimicrobial development. *Leishmania* LIR motifs are too similar to human ones, and we still do not know enough of the novel, kinetoplastid-specific protein-protein interactions and linear motifs to pinpoint an exact molecular target.

## Summary

In this work we have examined three classes of short linear motifs that are important for proteins that are associated with membranous systems of the cell. In all three cases, the known and predicted SLiMs and their interactors have diverged from their better studied equivalents in the animal, fungal and plant kingdoms. The signal peptide and KDEL motif interactions both occur in deep grooves of their receptor proteins. The unique features of the Leishmania systems suggest that these systems can potentially be directly targeted for therapeutic discovery. The classical LIR motifs by contrast are more similar to those of the host organisms making them less suited as direct targets. In addition, it is clear that most of the motifs in this Leishmania system have yet to be defined. Nevertheless, the dramatic expansion of the LIR/ATG systems in Leishmania suggest that identification of these divergent LIR-equivalent SLiMs would be likely to promote fruitful insights into kinetoplastid cell biology. We hope that the predictions for the three motif classes presented here will be of use to Leishmania researchers.

## Methods

### Signal peptide predictions

We downloaded Leishmania reference proteomes (*L*. *major*, *L*. *infantum*, *L*. *mexicana*, *L*. *braziliensis*, *L*. *donovani)* from LeishMANIAdb 1.0 [30], while SwissProt 2023_2 [33] was also used as a reference. Signal peptides were predicted using SignalP6.0 [32]. Amino acid distribution was calculated using these results, on the SignalP predicted hydrophobic regions and on full length proteins (S1 and S2 Tables). SRP54 protein folds (*A*. *thaliana*, *H*. *sapiens*, *S*. *solfataricus*, *B*. *saltans*, *T*. *cruzi*, *T*. *brucei*, *L*. *seymouri*, *L*. *infantum*) combined with signal peptides (*S*. *cerevisiae*, *L*. *infantum*) were predicted using ColabFold 1.5.2 [70] using the archeal SRP54 crystal structure as a template [34] (ptm scores: S3 Table). In order to use a second, fundamentally different approach, the SRP54 fold was also predicted using the Phyre2.0 server [35]. We used BLAST [71] to search for the closest hit for the *L*. *infantum* signal peptide (UniProt: A4HY78/A4HY79) considering the *S*. *cerevisiae* sequence from the crystal structure (notably the first hit was also predicted to have an SP by SignalP6, Fig 1/B). FoldX 5.0 [37] was used to relax the ColabFold 1.5.2 generated structures and to calculate their inferred structured stability (Fig 1/C and S4 Table).

For prediction, we prepared positive (signal peptide) and negative datasets (other) using the following approach: we selected proteins with predicted signal peptide (S5 Table), then extended these predictions to homologs using LeishMANIAdb (max 50% gap content and more than 50% sequence identity were required, identical sequences were used only once; S6 Table). Negative set contains proteins with membrane regions predicted in the first 50 residues by CCTOP [38], and other proteins (without any kind of prediction) (S6 Table). Positive data were used multiple times so the two dataset had equal size. The positive and the negative dataset were split into training (S7 Table) and test (benchmark set 1; S8 Table) sets (90% and 10%). In parallel, we randomly selected 100 proteins and manually annotated whether they have a signal peptide, or not (out of which 11 proteins were impossible to classify, these were omitted; benchmark set 2; S9 Table). To develop the prediction method we used transfer learning: the language model used the pretrained model: esm2_t12_35M_UR50D [39] and it was fine-tuned on the constructed training dataset (Fig 1/D). The prediction takes an amino acid sequence as input, and predicts whether the sequence has a signal peptide or not (but it cannot predict the cleavage site). After predicting signal peptides on the 5 Leishmania reference proteomes, we compared our result with other methods (Fig 1/E and S10–S13 Tables).

As for the limitation of our prediction algorithm, the lack of experimental data is a key problem. In addition, we could not ensure that the test set is fully independent: reducing redundancy to 70% would remove 80% of the sequences (as these are short, N-terminal segments only), therefore this approach would not have left enough data to train and test the network. Therefore our results should better be interpreted as an extension to already recognized signal peptide prediction methods (and to some extent it reflects SignalP6 parameters, as its’ prediction on the SignalP6 training set achieved 91% accuracy (S11 Table)). There is also a high ambiguity whether membrane regions from the negative set are truly membrane segments, and not signal peptides. Although CCTOP is equipped to discriminate against these regions, it also relies on earlier versions of SignalP that we meant to amend here. Notably, this rather strict approach will be less likely to yield false positive hits.

### Modeling of KDEL receptors and identification of ER-resident proteins

To explore KDEL-equivalent motifs in kinetoplastid proteomes, we first observed the C-termini of proteins from universal eukaryotic ER-resident protein families (BIP1, HYOU1, endoplasmin, calreticulin, thioredoxins, cyclophilins, etc., Fig 2/A and S14 Table). As a next step, we used the observation to search for similar, signal peptide-containing proteins in the *Leishmania infantum* and *Leishmania donovani* proteomes with either C-terminal El or DL consensus sequence (Fig 2/B). Then we assessed conservation of hits to increase our confidence in predictions. To compare them to the ER-resident proteome of the free-living *Bodo saltans*, we applied evolutionary conservation inferences (all protein families that have a KDEL-equivalent motif in at least one kinetoplastid proteome). Alignments were prepared using ClustalOmega [72]. We used IUPred3[73] and AlphaFold2 [74] to find disordered regions, and DeepLoc [75] to predict protein localizations.

Kinetoplastid KDEL-like motif logos presented on Fig 2/B include both known and novel (predicted ER-resident) families. For non-kinetoplastid eukaryotic organisms, we used annotated ER-resident examples for logo generation, except for *Giardia intestinalis*, where we had to resort to the same EL / DL C-terminus search in conjunction with the signal peptide requirement to predict ER-retained proteins. To see our complete motif collection with evolutionary comparisons across these organisms, see S1 Material. Novel kinetoplastid protein families were analyzed in evolutionary terms using UniProt BLAST searches and alignments. Families that either had no previously known homologs, or possessed multiple paralogs with only different branches studied were given new, human-readable names and suggested abbreviations (Fig 2/C).

For comparative analysis, we used the AlphaFold2 (AF2) predicted models of orthologous KDEL receptors from the following species: *Homo sapiens* (UniProt: P24390), *Saccharomyces cerevisiae* (UniProt: P18414), *Schizosaccharomyces pombe* (UniProt: O94270), *Arabidopsis thaliana* (UniProt: P35402), *Giardia intestinalis* (UniProt: A8B6P2), *Bodo saltans* (UniProt: A0A0S4JT51), *Trypanosoma cruzi* (UniProt: Q4DMI8), *Trypanosoma brucei brucei* (UniProt: Q384K5), *Leptomonas seymouri* (UniProt: A0A0N1I068), and *Leishmania infantum* (UniProt: A4I3D7). Visual analysis of the structures was done by superimposing them over an experimentally determined KDEL-receptor complex (Fig 2/D, pdb: 6I6H). The extended analysis also used predicted KDEL receptor structures for *Porcisia hertigi* (A0A836IGX2), *Phytomonas spp*. Hart1 (W6LFI0), *Phytomonas spp*. EM1 (W6KYJ3), *Strigomonas culicis* (S9UDF7), *Angomonas deanei* (A0A7G2CK51), *Trypanosoma theileri* (A0A1X0NYI5), *Trypanosoma conorhini* (A0A422Q6J1), *Neobodo designis* (A0A7S1LPH7), *Perkinsela spp*. CCAP1540/4 (A0A0L1KLY5) and *Eutreptiella gymnastica* (multiple paralogs: A0A7S1IBA2, A0A7S1NIW0, A0A7S4G7V5), as well as *Naegleria gruberi* (D2V4W2). The KDEL receptor for *Trypanosoma vivax* was not available in UniProt.

For all the above-mentioned species, we used BLAST searches in UniProt (limited to taxonomic class Discoba) to identify homologs of putative ER-resident proteins established for the core species. The KDEL-like sequence-containing homologs (see S15 Table) were used to generate all sequence logos by WebLogo. The evolutionary tree used the consensus branching topology of euglenozoan and kinetoplastid species, as established in the literature [76–78]. The orientation of receptor loops (inwards, outwards, very outwardly exposed) was assessed visually in PyMol (version 1.8) after superposition of all models. Their ClustalOmega alignments were also amended manually, by taking the orientations and 3D distances of amino acids on the gatekeeper loops of models into consideration (S4 and S5 Figs), and the amended alignments then used to establish the sequence-structure-function correlations.

To obtain a working model for the Leishmania C-terminal DL (Asp-Leu-COOH) motif recognition, we built a model of an optimal Leishmania ligand (using the peptide in pdb: 6I6H as a guide), and docked this peptide into the AlphaFold2 predicted receptor using HADDOCK 2.4 [40] (Fig 2/E). Flexible docking methods were deemed superior to homology modelling (including AlphaFold2 multimer), as the main chain geometry was expected to be substantially different from previously known examples. Models from the best energy cluster were merged into a multistate PDB (S1 Structures: KDEL-best-HADDOCK-run-01.pdb) and a representative instance was used to generate Fig 2/D, by superimposing it over the animal receptor-ligand structure (pdb: 6I6H).

To assess functionality of predicted ER-resident enzymes, we used extended multiple alignments (after repeated BLAST searches over Discoba) over their probable catalytic sites and AlphaFold2-predicted structural models for *Leishmania infantum* chitinase (A4HWX6_LEIIN), glutathionylspermidine amidase domain protein (the trypanothione synthase homolog ERGAD) (A4I1M7_LEIIN), ER-implicated kinetoplastid ATPase (the torsin homolog ERIKA) (A4HU03_LEIIN) and Shewanella-like phosphatase (SHELP) (A4I008_LEIIN). In all cases, reference crystal structures loaded with ligands or substrates served as a point of comparison (PDB: 1E6N, 2VPS, 5J1S and 2Z72, respectively–S7–S10 Figs).

### ATG8 structural analysis and LIR motif-containing partner prediction

To build a tree of ATG8/ATG12 family proteins in kinetoplastids, we retrieved individual sample proteins from UniProt, and performed BLAST searches to obtain more representatives of the same family. For reference, we included better-annotated ATG8 and ATG12 protein sequences from various animal, fungal and plant proteomes. The alignment was done using the UniProt interface with default settings (see S11 Fig for the resulting tree). These multiple alignments suggested that rooting of the tree is problematic, and while leishmanial ATG8A, ATG8B, ATG8C and ATG12 clearly belong to the same family and diverged only in *Leishmaniinae*, deeper relationships are unclear. AlphaFold2 modeled structures of the *Leishmania infantum* ATG8 (A4HYJ2), ATG12 (E9AH00), ATG8C.x (A4HTT6), ATG8B.2 (A4HYA4) and ATG8A.1 (E9AGS4) proteins were generated and superimposed in PyMOL 1.8 and critical amino acids were manually colored (Fig 3/A). For the purpose of surface conservation analyses (S12 Fig), the following sequences (UniProt Accession Numbers) were aligned to color the surface of Leishmania infantum ATG8-family proteins listed above: for ATG8: A4HYJ2, Q4QD46, A4HAB2, A0A0M9G613; for ATG12: E9AH00, Q4QBL7, A4HCG9, A0A0N0DZX5; for ATG8C: A4HTT6, Q4QI09, Q4QI15, Q4QI18, Q4QI08, Q4QI19, Q4QI13, Q4QI11, A4H5J3, A4H5J2, A0A381MCF0, A0A381MAT2, A0A381MBG2, A0A0M9FWM3, A0A0M9FWE5, A0A0N0VEA7, A0A0N0DTH2; for ATG8B: A4HYA4, A0A381MFR9, A4HYA5, Q4QDD6, Q4QDD3, Q4QDC5, Q4QDD0, Q4QDC8, A4HA43, A4HA42, A0A0M9G5V4, A0A0M9G5H5; for ATG8A: E9AGS4, Q4QDD1, Q4QDD4, A4HA39, A0A0N0DXB5 (all structurally fully intact, non-redundant ATG8-family protein sequences from L. infantum, L. major, L. braziliensis and Leptomonas pyrrhocoris). Figures of superimposed structures were prepared in PyMol 1.8 after overloading their B-factor according to ClustalOmega similarity symbols in each Clustal Omega alignment (*: 0.0,:: 0.33,.: 0.67, _:1.0) using a short custom Python script.

To identify conserved ATG8 partners with LIR motifs, we used UniProt BLAST (with default settings) querying with a set of known LIR-motif containing proteins (H. sapiens, S. cerevisiae, A. thaliana) involved in autophagy, to search proteins restricted to the taxonomic class Discoba. We only accepted hits as valid where the LIR motifs were also located in the same region (+50/-50 amino acids) in alignments, in case they were not matched perfectly. Homologous proteins were realigned using ClustalOmega via Jalview [79] to review the motif matches with reference sequences (S13–S15 Figs). Motif logos were prepared using the WebLogo server, in frequency plot mode [80]. To gain insights into the functionality of these hits, we modelled full-length *Leishmania infantum* ATG3 (A4I8P6_LEIIN) with end-processed ATG8 (A4HYJ2_LEIIN 1–120) using AlphaFold2 multimer over the ColabFold platform (S1 Structures: LeiATG3ATG8.pdb; ptm score: S3 Table). We repeated the same process for a short peptide segment from *Leishmania infantum* ATG2 (A4IBB7_LEIIN) as the whole protein is almost 3000 amino acids long (S1 Structures: LeiATG2pepATG8.pdb; ptm score: S3 Table). The ATG8-ATG4 complexes (A4HYJ2 [ATG8] with A4I521 [ATG4.2] and A4HTT6 [ATG8C.x] with A4I8K7 [ATG4.1] respectively) were also generated using AlphaFold2 multimer through the Colabfold platform. One representative structure of the 5 output models is shown in S13 and S14 Figs and on Fig 3/D for each complex (S1 Structures: LeiATG41ATG8C.pdb LeiATG42ATG8.pdb; ptm score: S3 Table). The sample LIR motif-containing *L*. *infantum* ATG8 complex model was also generated by AlphaFold2 multimer, and individual amino acids at the flanks were manually adjusted in PyMol 1.8, to better illustrate all polar H-bonding possibilities (Fig 3/B, S1 Structures: Lei-ATG8-LIR-best-model.pdb). The same peptide outline was overlain over the first row of structures on Fig 3/A to indicate the LIR binding interface. The incompatibility of Leishmania ATG12 LIR interface with the AIM12 motif was assessed by superimposing its AlphaFold2 model over the experimental crystal structure of the latter in complex with plant ATG12 (PDB: 7EU4).

Genetic architecture of ATG8/ATG12 family ORFs was analyzed by re-mapping UniProt annotated proteins to the chromosomal models at NCBI (*Leishmania infantum* JPCM5, *Leishmania major* strain Friedlin, *Leishmania braziliensis* MHOM/BR/75/M2904 and *Leptomonas pyrrhocoris*). Although shorter, interspersed, unannotated ORFs were also found to align with the ATG8 sequence, we only counted intact, full-length ATG8-family proteins to generate (Fig 3/C). However, all ORFs are listed in S18 and S19 Table.

### Performing proteome-wide scan for motifs presented in the paper

The LeiSig method that we developed to perform signal peptide prediction on Leishmania proteomes is available at https://leishmaniadb.ttk.hu/files/LeiSig.zip and at https://zenodo.org/records/10201868.

The modified KDEL motif can be searched using the regular expression (S14 Table; also see S1 Material for a short script).

The modified LIR motif can be searched using the regular expression (S21 Table; also see S1 Material for a short script).

### Structure models from proteins analyzed in the manuscript

All structures used for the analysis are listed in the S1 Structures file.

## Supporting information

S1 Table Amino acid distribution in hydrophobic regions of signal peptides. (XLSX)

S2 Table Full length protein amino acid distribution. (XLSX)

S3 Table AlphaFold2 ptm scores. (XLSX)

S4 Table Energy calculations for SRP54 + signal peptides. (XLSX)

S5 Table Signal peptides detected in LeishMANIAdb by SignalP6. (XLSX)

S6 Table Signal peptides mapped by considering orthologs. (XLSX)

S7 Table Training sets for Neural Networks. (XLSX)

S8 Table Benchmark set 1 for signal peptide prediction. (XLSX)

S9 Table Benchmark set 2 for signal peptide prediction. (XLSX)

S10 Table Evaluation of the Neural Network. (XLSX)

S11 Table Prediction result on SignalP6 training set. (XLSX)

S12 Table Positive Prediction result of 5 Leishmania species. (XLSX)

S13 Table Signal peptide prediction statistics for 5 Leishmania species. (XLSX)

S14 Table List of predicted ER-resident proteins for core species. (XLSX)

S15 Table List of homologous ER-resident proteins for extended species. (XLSX)

S16 Table Search results for kinetoplastid KDEL regular expression. (XLSX)

S17 Table PSSM for modified KDEL motif. (XLSX)

S18 Table ATG8 chromosome locations. (XLSX)

S19 Table ATG8 99% identical sequences. (XLSX)

S20 Table LIR motifs used from LIRcentral to establish regular expression. (XLSX)

S21 Table Search results for kinetoplastid LIR regular expression. (XLSX)

S1 Fig Left: X-ray structure of *Saccharomyces cerevisiae* SRP54 together with the signal peptide (PDB: 3KL4)—green: SRP54 helices mediating the interaction with the signal peptide; orange: poorly conserved loops; grey: signal peptide. Right: structural superimposition of kinetoplastid SRP54 proteins. Protein segment names were taken from Janda et al, *Nature*, 2010.
(TIF)

S2 Fig Superimposed Phyre2 (blue) and AlphaFol2 (orange) model of SRP54 *Leishmania infantum*
(TIF)

S3 Fig A detailed evolutionary trajectory analysis of KDEL receptors and motifs across Discoba (with n = 17 species). Branches are coloured according to the observed structure of the KDEL receptor gatekeeper loop. The logos of KDEL motifs for species featured in main Fig 2 are the same. The logos of additional species (n = 12) were derived from homology-based BLAST searches to predicted kinetoplastid ER-resident proteins (see supplementary tables). The small numbers below the logos denote the number of individual KDEL-bearing sequences per species (this might vary due to gene duplications, losses or incompletely annotated proteomes). The overall topology of the tree was based on literature.
(TIF)

S4 Fig A detailed analysis of KDEL receptors with structurally-guided alignments of the gatekeeper loops for each receptor (above) and their superimposed structure (below). The motif crystallized with the vertebrate KDEL receptor (pdb: 6I6H) is shown for wheat, while the xxDL motif modelled using HADDOCK is shown in orange. The arrow shows the displacement of the main chain due to the inward-facing loops in Trypanosomatidae.
(TIF)

S5 Fig The same KDEL receptors (as on S4 Fig) and ligand peptides viewed from a different angle. (TIF)

S6 Fig The features of the kinetoplastid KDEL receptor gatekeeper loops identified on the alignment of S3 Fig, on AlphaFold2 models (from EBI). (TIF)

S7 Fig ClustalO sequence alignment of core catalytic region of predicted ER-resident chitinases from diverse Euglenozoans, with diverse active and inactive chitinases (GH18 family, above). Despite a consistent exchange of the catalytic site Asp to Asn, these proteins all appear to be catalytically active (red = catalytic site residues). Structural model (**below**) of predicted ER-resident chitinase catalytic site from *Leishmania infantum* (A4HWX6_LEIIN), with the AlphaFold model superimposed on a bacterial chitinase crystal structure (pdb:1E6N), showing substrate oligosaccharide (yellow), selected important substrate coordinating residues (sticks) and conserved catalytic residues (red sticks)
(TIF)

S8 Fig Schematic domain architecture and ClustalO sequence alignment of catalytic triad residues (above) in bacterial and kinetoplastid trypanothione synthase enzymes (A) and the related ERGAD proteins (B), showing that the latter are likely inactive as hydrolases. Structural model of AlphaFold predicted ER-resident glutathionylspermidine amidase domain protein (below) from *Leishmania infantum* (A4I1M7_LEIIN, wheat), superimposed onto the amidase catalytic site of Leishmania major bifunctional trypanothione synthase-amidase protein (pdb: 2VPS, red). These putative ER-targeted kinetoplastid proteins lack the Cys amino acid, rendering them inactive as a hydrolase, unlike their closely related cytoplasmic homologs.
(TIF)

S9 Fig ClustalO sequence alignment (above) of the ATP binding pocket of two paralogous subfamilies of kinetoplastid torsins identified in the current study. Both the more conventional torsins (lacking the KDEL-like motif) and the retention signal containing ER-implicated kinetoplastid ATPases (ERIKA) conserve an intact ATPase catalytic site. Hyperconserved ATP coordinating residues (also found in human torsins) are highlighted in red. Structural model (below) of the ATPase catalytic site of the AlphaFold predicted *Leishmania infantum* ER retention signal containing kinetoplastid-specific torsin paralog ERIKA (A4HU03_LEIIN, ER-implicated kinetoplastid ATPase). The hyperconserved residues are shown as red sticks, while other important ATP-coordinating residues are also shown in stick representation. The position of ATP has been inferred by superposition onto the crystal structure of human Torsin 1A (pdb: 5J1S). The perfect ATP coordination implies an active enzyme.
(TIF)

S10 Fig Structural model of AlphaFold predicted ER-resident Shewanella-like phosphatase (SHELP) from *Leishmania infantum* (A4I008_LEIIN, orange) superimposed on the catalytic site of the crystal structure of phosphatase from Shewanella sp. bacterium (pdb: 2Z72, light blue). All metal ion coordinating and other catalytic site residues are perfectly conserved, implying an active enzyme.
(TIF)

S11 Fig ClustalO-based tree of ATG8/ATG12 family proteins in kinetoplastids. (TIF)

S12 Fig Surface conservation analysis of typical and atypical ATF8-family proteins in Leishmaniinae (front and back side of the same protein). The coloring of residues reflects the alignment symbols used by ClustalO. Conserved (identical) regions are displayed in red. Putative protein-protein interaction surfaces are indicated on each protein, while the +++ symbols indicate the surface that is most conserved in each atypical ATG8 subfamily. The numbers in brackets indicate the number of non-identical sequences used for alignment. The structures are AlphaFold2-predicted proteins for *Leishmania infantum*, (A4HYJ2_LEIIN, E9AH00_LEIIN, A4HTT6_LEIIN, A4HYA4_LEIIN, E9AGS4_LEIIN) accessed from the EBI website (identical to main Fig 3)
(TIF)

S13 Fig ClustalO alignment of kinetoplastid ATG2 protein sequences surrounding the conserved ATG8-binding LIR motif (above). To illustrate conservation, the *Caenorhabditis elegans* motif (conserved up to human ATG2A and ATG2B) is shown as a reference. The lack of perfect alignment in certain species is likely due to very low conservation of the surrounding sequence. The LIR motif is located on a disordered loop of the protein, as suggested by AlphaFold2 models (below). The AlphaFold2 multimer modelled structure of Leishmania infantum ATG8 with the ATG2 peptide also shows excellent surface compatibility.
(TIF)

S14 Fig ClustalO alignment of ATG3 protein sequences from Discoba (kinetoplastids and their relatives) surrounding the conserved, but atypical ATG8-binding LIR motif, as well as the ATG12-binding AIM12 motif only conserved outside of kinetoplastids. The human protein is shown in the alignment to illustrate conservation. The AlphaFold2 multimer modelled structure of the complex is shown below. The atypical LIR motif is located on a loop that fits the surface of *Leishmania infantum* ATG8.
(TIF)

S15 Fig ClustalO alignment and frequency logo of ATG4 (ATG4.2) protein sequences from Discoba (kinetoplastids and their relatives) surrounding the conserved ATG8-binding LIR motif. The motif turned into an atypical (possibly reverse) variant among certain kinetoplastid genera (*Trypanosoma spp*. and *Bodo saltans*) but is retained in its original form in others. Multiple human, yeast and Arabidopsis reference sequences illustrate conservation.
(TIF)

S1 Material S Material includes, scripts to screen LIR/KDEL motifs defined by regular expression in Leishmania proteomes, Web-servers to screen LIR/KDEL motifs defined by regular Expression; KDEL receptors and identification of ER-resident proteins and ATG8 family protein alignments. (PDF)

S1 Structures SRP54-signal peptide models:signal_*_lei.pdb. signal_*_sc.pdb. Best energy HADDOCK structures for L infantum KDEL models: KDEL-best-HADDOCK-run-01.pdb. AlphaFold2 Multimer output for ATG8-ATG2 peptide complex: Lei-ATG2pep-ATG8.pdb. AlphaFold2 Multimer output for ATG8-ATG3 complex: Lei-ATG-3ATG8.pdb. AlphaFold2 Multimer outputs for ATG8-ATG4 complexes: LeiATG41ATG8C.pdb. LeiATG42ATG8.pdb. Cognate idealized LIR peptide modelled onto the L infantum ATG8 protein: Lei-ATG8-LIR-best-model.pdb.
(ZIP)
