# Supplementary figures and images for: Linear motifs regulating protein secretion, sorting and autophagy in Leishmania parasites are diverged with respect to their host equivalents
Source: PLoS Comput Biol. 2024 Feb 16;20(2):e1011902. doi: 10.1371/journal.pcbi.1011902 (PMC10903960; doi:10.1371/journal.pcbi.1011902)

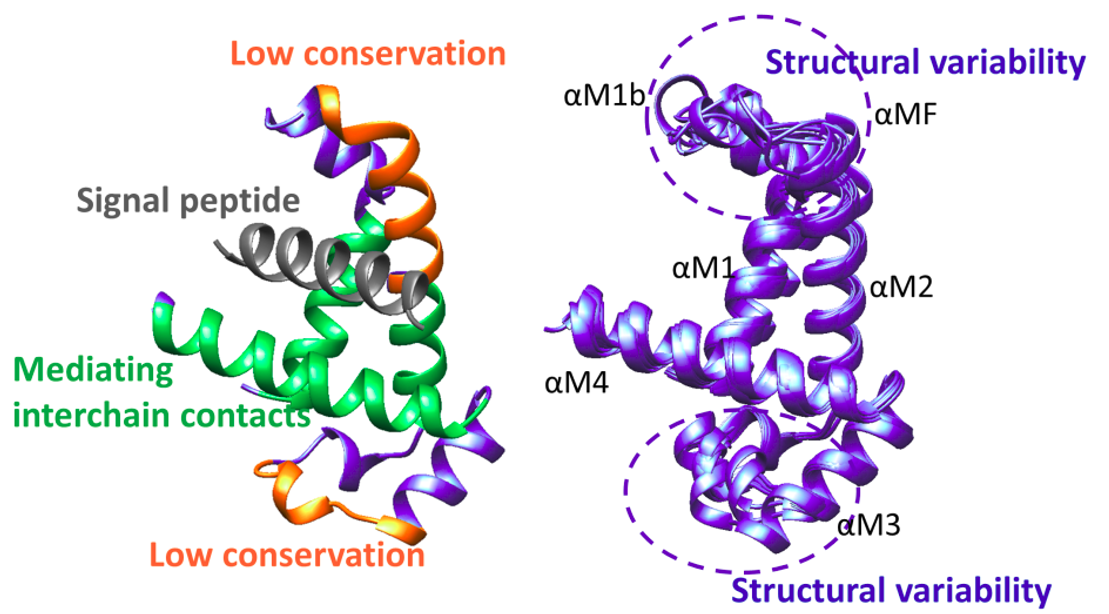

Supplement: S1 Fig — Left: X-ray structure of Saccharomyces cerevisiae SRP54 together with the signal peptide (PDB: 3KL4)—green: SRP54 helices mediating the interaction with the signal peptide; orange: poorly conserved loops; grey: signal peptide. Right: structural superimposition of kinetoplastid SRP54 proteins. Protein segment names were taken from Janda et al, Nature, 2010. (TIF) [file pcbi.1011902.s022.tif]

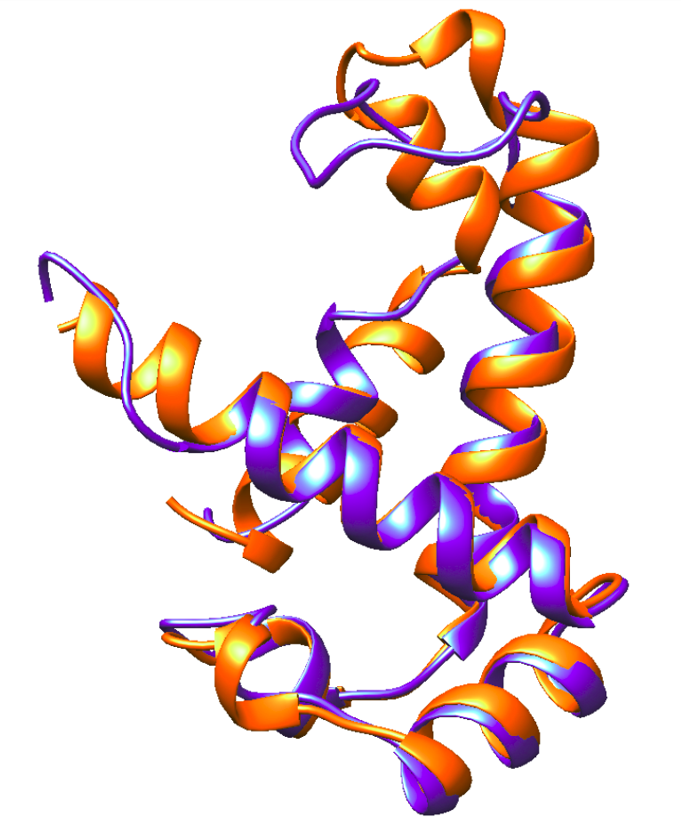

Supplement: S2 Fig — Superimposed Phyre2 (blue) and AlphaFol2 (orange) model of SRP54 Leishmania infantum (TIF) [file pcbi.1011902.s023.tif]

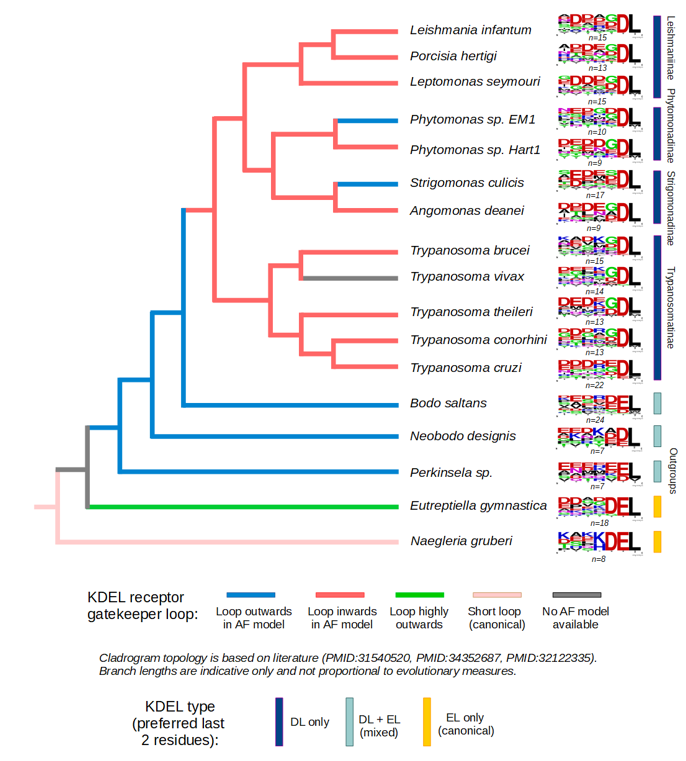

Supplement: S3 Fig — Branches are coloured according to the observed structure of the KDEL receptor gatekeeper loop. The logos of KDEL motifs for species featured in main Fig 2 are the same. The logos of additional species (n = 12) were derived from homology-based BLAST searches to predicted kinetoplastid ER-resident proteins (see supplementary tables). The small numbers below the logos denote the number of individual KDEL-bearing sequences per species (this might vary due to gene duplications, losses or incompletely annotated proteomes). The overall topology of the tree was based on literature. (TIF) [file pcbi.1011902.s024.tif]

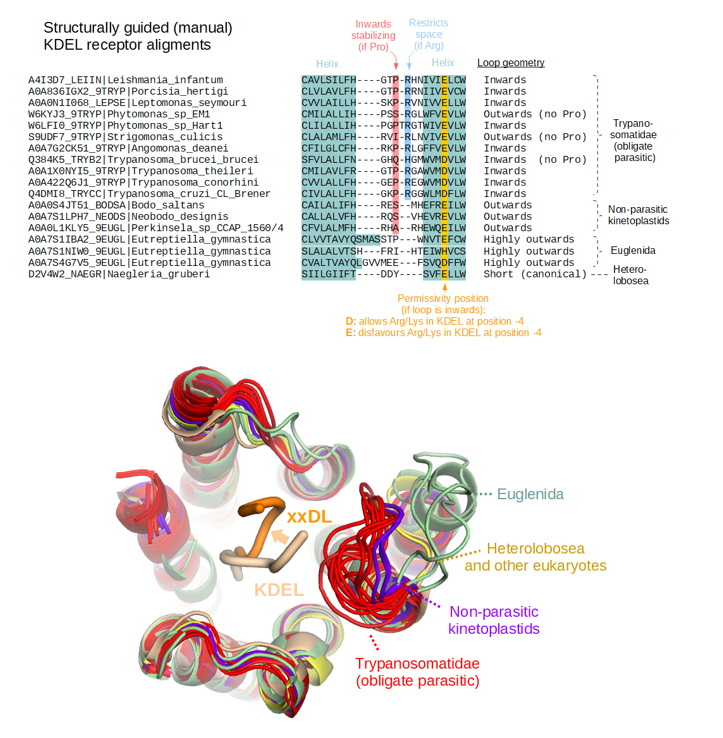

Supplement: S4 Fig — A detailed analysis of KDEL receptors with structurally-guided alignments of the gatekeeper loops for each receptor (above) and their superimposed structure (below). The motif crystallized with the vertebrate KDEL receptor (pdb: 6I6H) is shown for wheat, while the xxDL motif modelled using HADDOCK is shown in orange. The arrow shows the displacement of the main chain due to the inward-facing loops in Trypanosomatidae. (TIF) [file pcbi.1011902.s025.tif]

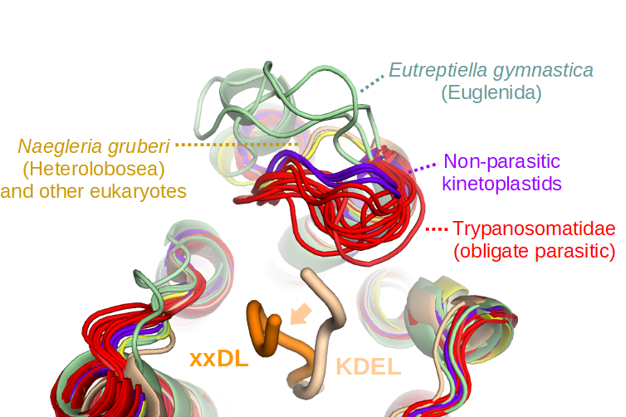

Supplement: S5 Fig — (TIF) [file pcbi.1011902.s026.tif]

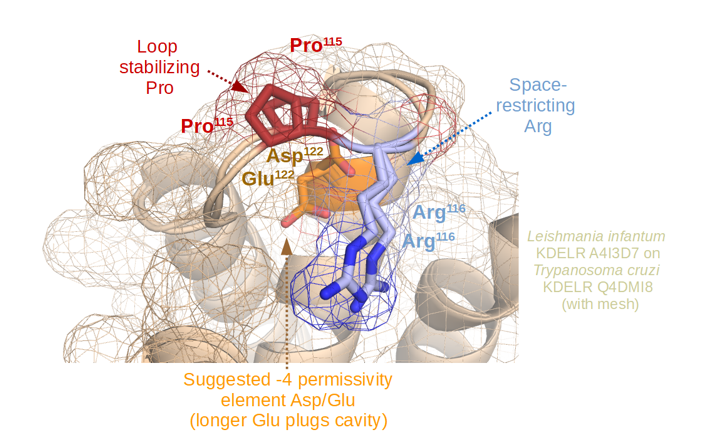

Supplement: S6 Fig — (TIF) [file pcbi.1011902.s027.tif]

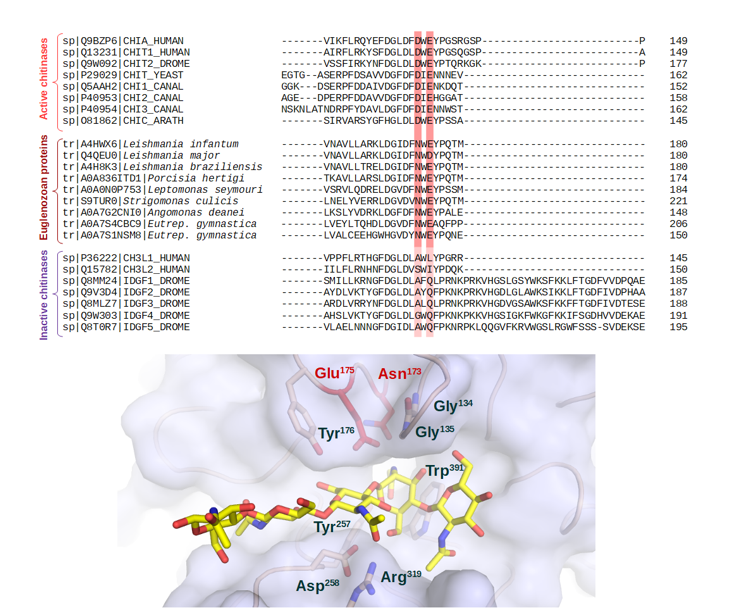

Supplement: S7 Fig — Despite a consistent exchange of the catalytic site Asp to Asn, these proteins all appear to be catalytically active (red = catalytic site residues). Structural model (below) of predicted ER-resident chitinase catalytic site from Leishmania infantum (A4HWX6_LEIIN), with the AlphaFold model superimposed on a bacterial chitinase crystal structure (pdb:1E6N), showing substrate oligosaccharide (yellow), selected important substrate coordinating residues (sticks) and conserved catalytic residues (red sticks) (TIF) [file pcbi.1011902.s028.tif]

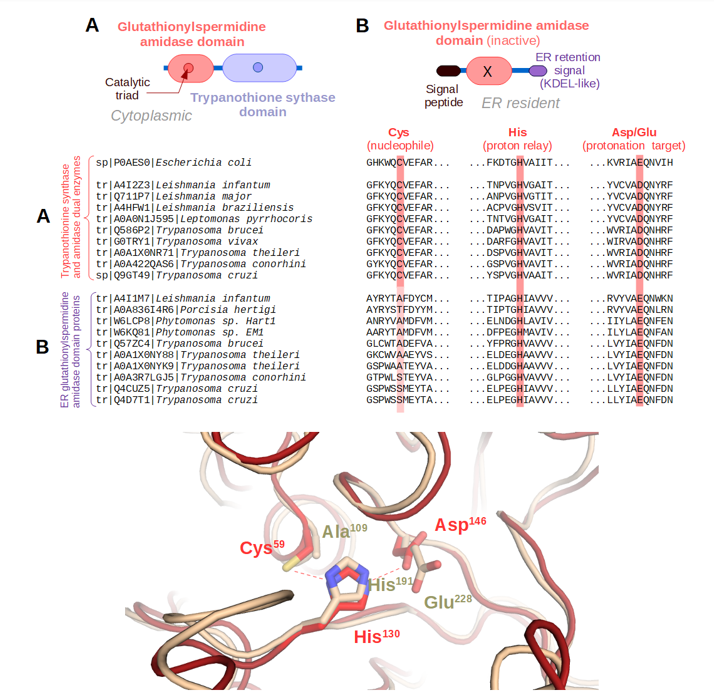

Supplement: S8 Fig — Schematic domain architecture and ClustalO sequence alignment of catalytic triad residues (above) in bacterial and kinetoplastid trypanothione synthase enzymes (A) and the related ERGAD proteins (B), showing that the latter are likely inactive as hydrolases. Structural model of AlphaFold predicted ER-resident glutathionylspermidine amidase domain protein (below) from Leishmania infantum (A4I1M7_LEIIN, wheat), superimposed onto the amidase catalytic site of Leishmania major bifunctional trypanothione synthase-amidase protein (pdb: 2VPS, red). These putative ER-targeted kinetoplastid proteins lack the Cys amino acid, rendering them inactive as a hydrolase, unlike their closely related cytoplasmic homologs. (TIF) [file pcbi.1011902.s029.tif]

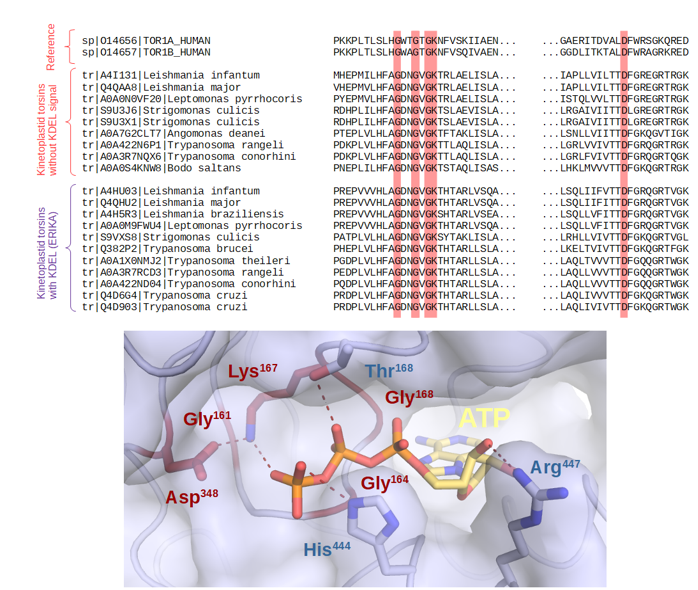

Supplement: S9 Fig — ClustalO sequence alignment (above) of the ATP binding pocket of two paralogous subfamilies of kinetoplastid torsins identified in the current study. Both the more conventional torsins (lacking the KDEL-like motif) and the retention signal containing ER-implicated kinetoplastid ATPases (ERIKA) conserve an intact ATPase catalytic site. Hyperconserved ATP coordinating residues (also found in human torsins) are highlighted in red. Structural model (below) of the ATPase catalytic site of the AlphaFold predicted Leishmania infantum ER retention signal containing kinetoplastid-specific torsin paralog ERIKA (A4HU03_LEIIN, ER-implicated kinetoplastid ATPase). The hyperconserved residues are shown as red sticks, while other important ATP-coordinating residues are also shown in stick representation. The position of ATP has been inferred by superposition onto the crystal structure of human Torsin 1A (pdb: 5J1S). The perfect ATP coordination implies an active enzyme. (TIF) [file pcbi.1011902.s030.tif]

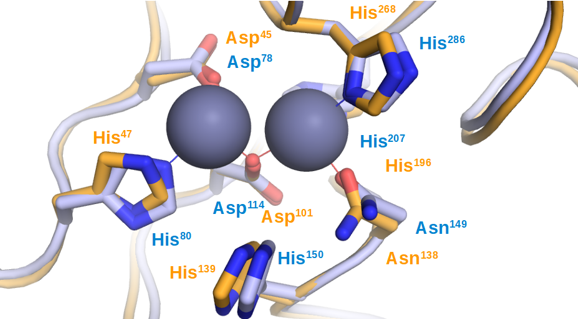

Supplement: S10 Fig — All metal ion coordinating and other catalytic site residues are perfectly conserved, implying an active enzyme. (TIF) [file pcbi.1011902.s031.tif]

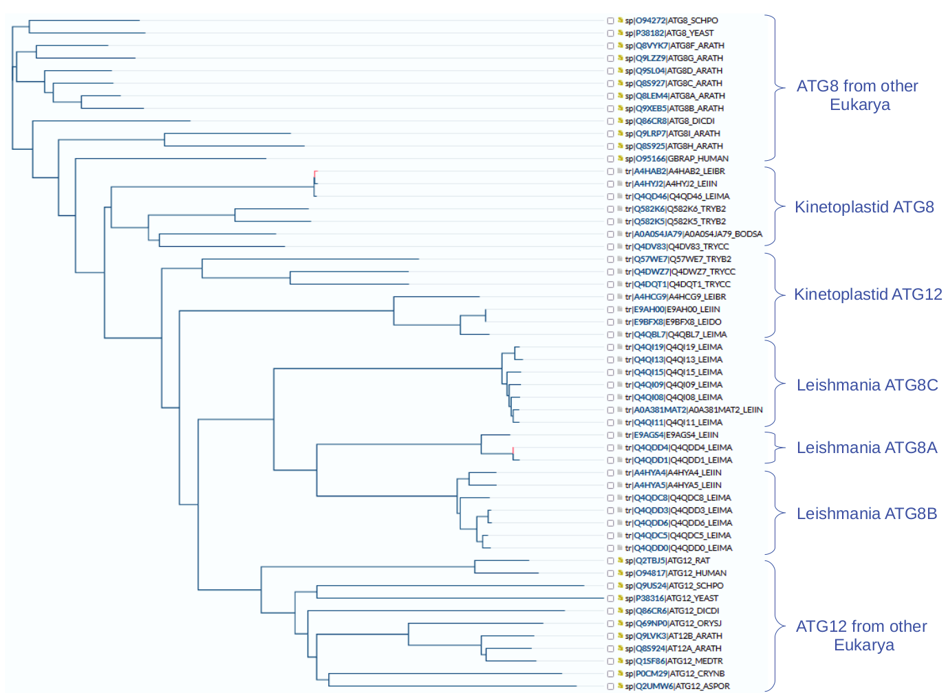

Supplement: S11 Fig — (TIF) [file pcbi.1011902.s032.tif]

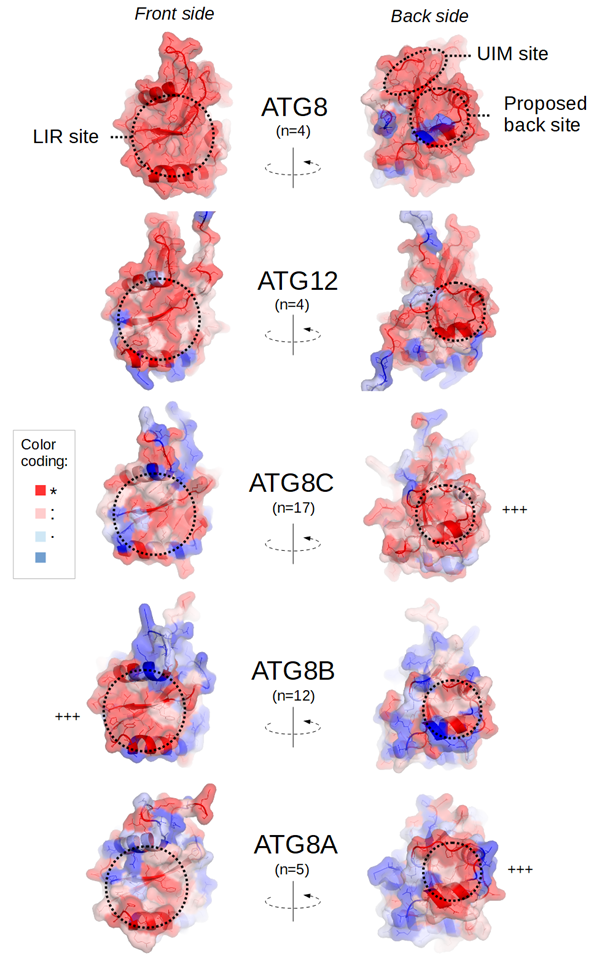

Supplement: S12 Fig — The coloring of residues reflects the alignment symbols used by ClustalO. Conserved (identical) regions are displayed in red. Putative protein-protein interaction surfaces are indicated on each protein, while the +++ symbols indicate the surface that is most conserved in each atypical ATG8 subfamily. The numbers in brackets indicate the number of non-identical sequences used for alignment. The structures are AlphaFold2-predicted proteins for Leishmania infantum, (A4HYJ2_LEIIN, E9AH00_LEIIN, A4HTT6_LEIIN, A4HYA4_LEIIN, E9AGS4_LEIIN) accessed from the EBI website (identical to main Fig 3) (TIF) [file pcbi.1011902.s033.tif]

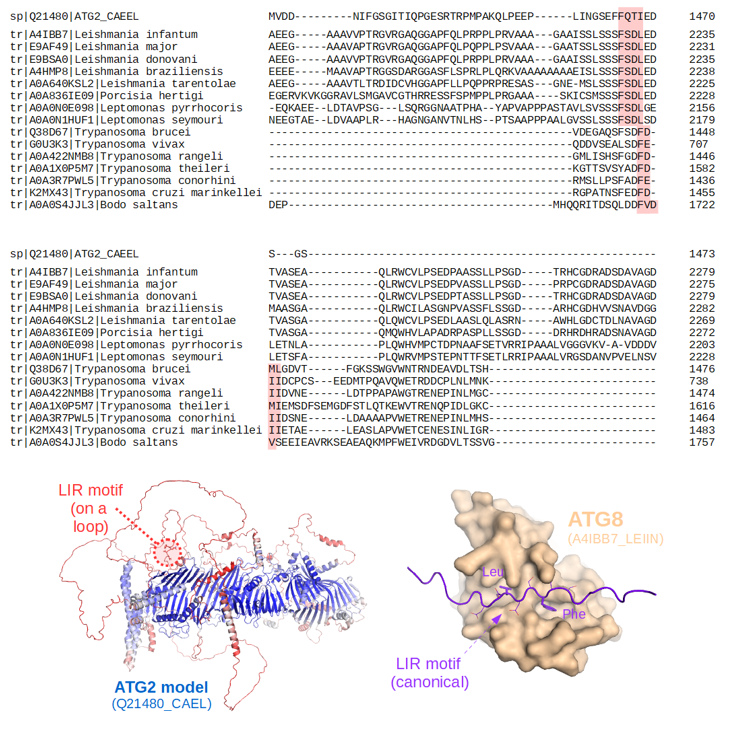

Supplement: S13 Fig — To illustrate conservation, the Caenorhabditis elegans motif (conserved up to human ATG2A and ATG2B) is shown as a reference. The lack of perfect alignment in certain species is likely due to very low conservation of the surrounding sequence. The LIR motif is located on a disordered loop of the protein, as suggested by AlphaFold2 models (below). The AlphaFold2 multimer modelled structure of Leishmania infantum ATG8 with the ATG2 peptide also shows excellent surface compatibility. (TIF) [file pcbi.1011902.s034.tif]

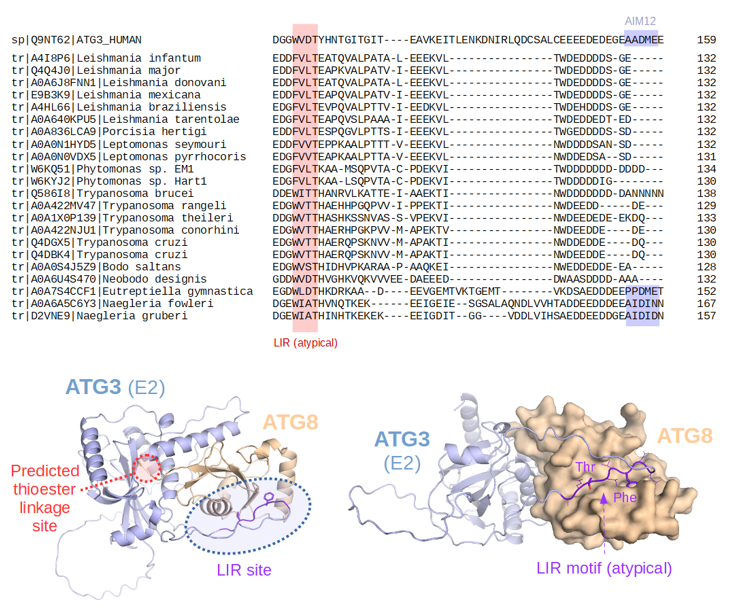

Supplement: S14 Fig — The human protein is shown in the alignment to illustrate conservation. The AlphaFold2 multimer modelled structure of the complex is shown below. The atypical LIR motif is located on a loop that fits the surface of Leishmania infantum ATG8. (TIF) [file pcbi.1011902.s035.tif]

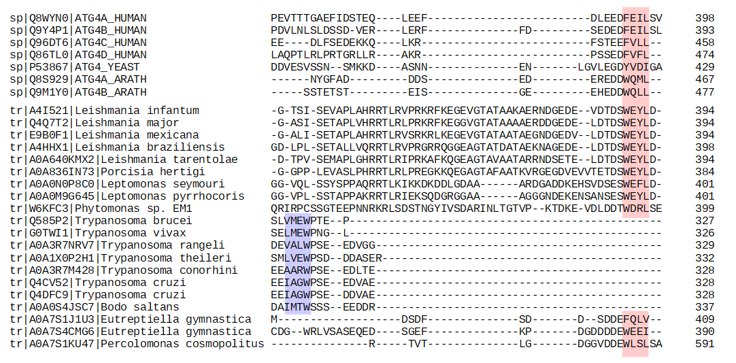

Supplement: S15 Fig — The motif turned into an atypical (possibly reverse) variant among certain kinetoplastid genera (Trypanosoma spp. and Bodo saltans) but is retained in its original form in others. Multiple human, yeast and Arabidopsis reference sequences illustrate conservation. (TIF) [file pcbi.1011902.s036.tif]
