# Supplementary material for: Linear motifs regulating protein secretion, sorting and autophagy in Leishmania parasites are diverged with respect to their host equivalents
Source: PLoS Comput Biol. 2024 Feb 16;20(2):e1011902. doi: 10.1371/journal.pcbi.1011902 (PMC10903960; doi:10.1371/journal.pcbi.1011902)
Supplement: S1 Material — (PDF) [file pcbi.1011902.s037.pdf]

Supplementary Material for "Linear motifs regulating protein secretion, sorting and autophagy in Leishmania parasites are diverged with respect to their host equivalents"

Andras Zeke, Toby J. Gibson & Laszlo Dobson

Content

2	Scripts to screen LIR/KDEL motifs defined by regular expression in Leishmania proteomes
2	Web-servers to screen LIR/KDEL motifs defined by regular Expression
2	Availability of the Leisig software
3	KDEL receptors and identification of ER-resident proteins
62	ATG8 family protein alignments

Scripts to screen LIR/KDEL motifs defined by regular expression in Leishmania proteomes

Download Leishmania reference proteome from any website. For example you can use the UniProt FTP site to download the most current reference proteome:

https://ftp.uniprot.org/pub/databases/uniprot/current_release/knowledgebase/reference_proteomes/

Read the supplied fasta file into any kind of data structure using python, then iterate through all sequences. Using the following script, you can retrieve hits:

```
import re

ID=""          #this is a string that contains the protein identifier
sequence=""    #this is a string that contains the amino acid sequence
regex=""       #this is a string that contains the regular expression (considering
               #this paper: KDEL: "DL$"
               #this paper: LIR:
               "([^RK][^RK][DEST][WF][^PGRKFYWH][^PGRK][LIV])|([^RK][DEST][^RK][WF][^
               PGRKFYWH][^PGRK][LIV])|([DEST][^RK][^RK][WF][^PGRKFYWH][^PGRK][LIV])|(
               [^RK][^RK][^RK][WF][^PGRKFYWH][^PGRK][LIV][^RK]{0,1}[DE])"



p = re.compile(regex)
for m in p.finditer(sequence):
       print(ID,m.start(),sequence[m.start():m.end()])
```

Web-servers to screen LIR/KDEL motifs defined by regular expression

The provided regular expressions can be also used on the http://slim.icr.ac.uk/slimsearch/ website to find potential motif hits in selected proteomes.

Availability of the Leisig software

The LeiSig software is available at https://zenodo.org/records/10201868 and at https://leishmaniadb.ttk.hu/files/leishMANIAdb1.0.zip

KDEL receptors and identification of ER-resident proteins

01. Canopy architecture related Saponin B1 (CARSAB)

These proteins are alignment-wise restricted to kinetoplastids (homology is too weak outside this group). No ortholog was identified past B saltans. However, domain architecture analyses (InterPro) betray the presence of a Saponin B domain (often, but not always, involved in lipid transfer). The group is distantly related to Canopy proteins (Conserved ER proteins with KDEL, of unclear function) found in animals and plants. Forced alignment of animal (Human, Drosophila) Canopy proteins show that disulphide bridges are matching perfectly, implicating a conserved architecture (other Saponin B domains often have different disulphide architecture). Thus these two distant families (animal Canopy and kinetoplastid CARSAB) must be evolutionarily related, despite the distance.Note that the other Saponin B domain bearing ER protein family (SES1/SESSAB) identified in kinetoplastids have different architecture and do not align well. The ultimate proof for the distinct nature of these two families is the presence of both in the same organism (Arabidopsis thaliana has both SES1 and Canopy homologs).

Kinetoplastid

```
tr|A0A0S4JVF1|A0A0S4JVF1_BODSA      ------------------------------------------------------------    0
tr|Q381R4|Q381R4_TRYB2              ------------------------------------------------------------    0
tr|A0A1X0NUB5|A0A1X0NUB5_9TRYP      ------------------------------------------------------------    0
tr|Q4CZ44|Q4CZ44_TRYCC              MFACISVCLYFISLRGMNLCELCFKHRTSFSFFF-AIFFLYYPLFGWASAESSTKIKQKT    59
tr|Q4D5S5|Q4D5S5_TRYCC              MFACISPC--FISLRGMNLCKICFKHRTSFSFSFLPFFFFYYPLFGWASAESSTKTKQKK    58
tr|A0A3R7K0H2|A0A3R7K0H2_9TRYP      ------------------------------------------------------------    0
tr|A0A422NB75|A0A422NB75_TRYRA      ------------------------------------------------------------    0
tr|S9UAW3|S9UAW3_9TRYP              ------------------------------------------------------------    0
tr|A0A0N0DZ25|A0A0N0DZ25_LEPPY      ------------------------------------------------------------    0
tr|A4HKJ0|A4HKJ0_LEIBR              ------------------------------------------------------------    0
tr|Q4Q576|Q4Q576_LEIMA              ------------------------------------------------------------    0
tr|A4I822|A4I822_LEIIN              ------------------------------------------------------------    0

tr|A0A0S4JVF1|A0A0S4JVF1_BODSA      ----------------------------------------------MAVAFSV-------    7
tr|Q381R4|Q381R4_TRYB2              ------------------------MYA-----RLSNTSQSYYSII-----SKIFQLLAII    26
tr|A0A1X0NUB5|A0A1X0NUB5_9TRYP      ------------------------MRH--CFFSSRGINASSLLL---------ILCLLIT    25
tr|Q4CZ44|Q4CZ44_TRYCC              K-------TKENTIKGEILEKLGAMWF--FFLLFSFLNQKFMPF---------VIFLFTL    101
tr|Q4D5S5|Q4D5S5_TRYCC              KKKTKTKTKKENTIKGGILERLEAMWF--FLLLFSFLNRKFMPF---------VIFLFTL    107
tr|A0A3R7K0H2|A0A3R7K0H2_9TRYP      ------------------------MRL--CLPINSPVRQTSLFF---------ALCLFTL    25
tr|A0A422NB75|A0A422NB75_TRYRA      ------------------------------------------------------------    0
tr|S9UAW3|S9UAW3_9TRYP              --------------------------------------------------MKPFVTLLIA    10
tr|A0A0N0DZ25|A0A0N0DZ25_LEPPY      ------------------------MHTFHAKPLHPTLEQRVR-FAAHACALLFLLILLSL    35
tr|A4HKJ0|A4HKJ0_LEIBR              ------------------------MR-APSTPLLAPLQRNQRHFLLAVIAL--LVNLFLA    33
tr|Q4Q576|Q4Q576_LEIMA              ------------------------MR-IHSTFLLAPPQRHQRCCLLASVAL--LASLLLA    33
tr|A4I822|A4I822_LEIIN              ------------------------MQ-RHSTFLLAPPQRHQQCCLLALVAL--LAILLLA    33

tr|A0A0S4JVF1|A0A0S4JVF1_BODSA      -----VFLAFIASALEPL--SSQPQLQCSACLAIAELVGQKMNDTAKL----KTTFQASH    56
tr|Q381R4|Q381R4_TRYB2              GSISLVEAATVGDPLFRIHEPTYAFLDCSACIAFAGHLGRRMNESIRYGGRGGATFLSSH    86
tr|A0A1X0NUB5|A0A1X0NUB5_9TRYP      GDVLSVFAADPNDPRFRIHEPSYAYLDCSACLAFAEYLGRRMNASLEHGG-SGASFLTTH    84
tr|Q4CZ44|Q4CZ44_TRYCC              TDVMSASAAILKDPRFLIHGPSYAYLDCSACLAFAGYLGERMNASLEYDGRGGASFLATH    161
tr|Q4D5S5|Q4D5S5_TRYCC              ADFMSVSAAILKDPRFLIHGPSYAYLDCSACLAFAGYLGQRMNASLEYDGRGGASFLATH    167
tr|A0A3R7K0H2|A0A3R7K0H2_9TRYP      GGVLSGSAATLDDPRFRIHGPSYAYMDCSACIAFAGYLGQRMNASLEYDGRGGATFLSTH    85
tr|A0A422NB75|A0A422NB75_TRYRA      --MLSASAATLGDPRFRIHGPSYAYMDCSACIAFAGYLGQRMNASLEYDGRGGATFLSTH    58
tr|S9UAW3|S9UAW3_9TRYP              ALC--VVAGTAAIDLPQQHPSGYAHMDCSSCLAVAHVLSDKMNKSLLA---SSSSFLSSH    65
tr|A0A0N0DZ25|A0A0N0DZ25_LEPPY      AAPALVATAHTGHSVRPLHGPGYEHLDCSACLTVSRALFVRLNATLSE---SPSTYLASH    92
tr|A4HKJ0|A4HKJ0_LEIBR              SSLTLAATPAQGKSIRPLHGAGYEHLDCSACITVARTLFNRLNKTLAE---NPSTYLVSH    90
tr|Q4Q576|Q4Q576_LEIMA              LPAVLAVTPPQGKPIRPLHGAGYEHLDCSACITVARTLFERLNQTLAE---NPSTYLISH    90
tr|A4I822|A4I822_LEIIN              SPTVLAAPPPQGKPIRPLHGAGYEHLDCSACITVARTLFERLNQTLAE---NPSTYLISH    90
                                                              ::**:*::.:  :  ::* :      :::  :*

tr|A0A0S4JVF1|A0A0S4JVF1_BODSA      RLDARNKVKRIDYESSELRAYEILDGFCNELE-KDYIFRLGEN-NLRLFSSN--------    106
tr|Q381R4|Q381R4_TRYB2              RLGKDNKLKRRDYATSELRGAEVLEKICNDSLFDDYALHFRADMRIRVYDNG--------    138
tr|A0A1X0NUB5|A0A1X0NUB5_9TRYP      RLSETNKLKRQAYATSELRAVEVLEKICNDELEENFVLRLDTDKRIRVYESE--------    136
tr|Q4CZ44|Q4CZ44_TRYCC              RLNKENKLQRNLYATSELRAVEVLEKICSVALEENYVLHLDIDRRIRVYQSG--------    213
tr|Q4D5S5|Q4D5S5_TRYCC              RLNKENKLQRNLYATSELRAVEVLEKICSVTLEENYVLHLDIDRRIRVYQSG--------    219
tr|A0A3R7K0H2|A0A3R7K0H2_9TRYP      RLDAKNKLQRSVYATSELRSVEVLEKICNDALEENYVLRLDLDRRIRVYESE--------    137
tr|A0A422NB75|A0A422NB75_TRYRA      RLNEENKLQRSVYATSELRSVEVLEKICNDALRENYVLRLDLDRRIRVYESE--------    110
tr|S9UAW3|S9UAW3_9TRYP              RLDTANQLKRVQYADSELRAAEVLEDACNKEVYEWYHLRVNPLTKVRGYHFFYYSHIQAV    125
tr|A0A0N0DZ25|A0A0N0DZ25_LEPPY      RLNRVNQLKRRPYRNSELLVTEVMDNFCSSYKNDERTLRLHPKSKVRLYHQQIWKDVTLG    152
```

```
tr|A4HKJ0|A4HKJ0_LEIBR              RLSKQNQLRRRQYRSSELLVSEVMENMCATYQNDNRLLRLHPKSKVRLYHQQIFGDARLG    150
tr|Q4Q576|Q4Q576_LEIMA              RLGKKNQLRRRQYRNSELLVTEVMENVCTSYKNDARLLRLHPKSKVRLYHQQVFGDTRLG    150
tr|A4I822|A4I822_LEIIN              RLGKKNQLRRRQYRNSELLVTEVMENVCTSYKNDARLLRLHPKSKVRLYHQQVFGDTRLG    150
                                    **.  *:::*  *  ***   *:::  *       .   ::.  .:* :

tr|A0A0S4JVF1|A0A0S4JVF1_BODSA      --------------------------KSLTRANYYGKNDKKELRSV--SKRLRDICNEIT    138
tr|Q381R4|Q381R4_TRYB2              -------------------------ESEYPLAQHYSKEDSRILKAV-KTKAVHVFCTRVM    172
tr|A0A1X0NUB5|A0A1X0NUB5_9TRYP      -------------------------KSDLPFAQHYSTQDSKALKET-SKTAVRSFCTRVM    170
tr|Q4CZ44|Q4CZ44_TRYCC              -------------------------NTDFPYAQHYSVQDSDALKVI-SKTAVRDFCTRLM    247
tr|Q4D5S5|Q4D5S5_TRYCC              -------------------------NTDFPYAQHYSVQDSAALKVV-SKTAVRDFCTRLM    253
tr|A0A3R7K0H2|A0A3R7K0H2_9TRYP      -------------------------KSDSPFAQHYSLQDAEALKSV-SKTAVRSFCTRVM    171
tr|A0A422NB75|A0A422NB75_TRYRA      -------------------------KSDSPFAQHYSMQDADALKSP-SKTAVWTFCTRVM    144
tr|S9UAW3|S9UAW3_9TRYP              TRPILLEGVAFPANS---TYEDDRPSKMAAVAQLYSTSEKKKLGPL-HKNAPASLCVQIM    181
tr|A0A0N0DZ25|A0A0N0DZ25_LEPPY      VRHSLREDEVYPEGAHDPQWDDYVELRRYQVAKVYSPKDQGALHGMEMLAATPTMCATLV    212
tr|A4HKJ0|A4HKJ0_LEIBR              VRHALREDEVYPADADPAAWDNKLDGHLYPIARLYSVRDADALHGMQSLSATPTMCALLV    210
tr|Q4Q576|Q4Q576_LEIMA              VRHALREDEVYPADADPAAWAEKLDGQLYPIARLYSRRDADALQGLQSLSATPTMCALLV    210
tr|A4I822|A4I822_LEIIN              VRHALREDEVYPADADSAAWAEKLDGQLYPIARLYSRQDADALQGLQSLSATPTMCALLV    210
                                                                      *. *.    :   *             :*  :

tr|A0A0S4JVF1|A0A0S4JVF1_BODSA      EEDDTKIISLIKKERYLDEFQQKLCFSEGFK-----------------------------    169
tr|Q381R4|Q381R4_TRYB2              DEEEDAMTSLVREVELLSELEQRLCGGYLEM-RNIT---------------APPFPVEKA    216
tr|A0A1X0NUB5|A0A1X0NUB5_9TRYP      GEEEEAMIALVREEQNLTNVEWRLCDGSTDEN---S---------------TRPESLGES    212
tr|Q4CZ44|Q4CZ44_TRYCC              DEEEEAMMNVVREVRELAELELRLCGGNDVNRSSVK---------------TPLPKIEAL    292
tr|Q4D5S5|Q4D5S5_TRYCC              DEEEEAMMNVVREVRELAELELRLCGGNDVNRSSVK---------------TSLPKFETL    298
tr|A0A3R7K0H2|A0A3R7K0H2_9TRYP      DEEEDAMMALVREVRELAELERRLCGGNDVNSSAAT---------------TPPPPLEEL    216
tr|A0A422NB75|A0A422NB75_TRYRA      SEEEDAMMALVREVHDLTELERRLCGGNDVNSSAVT---------------TPPPPLENL    189
tr|S9UAW3|S9UAW3_9TRYP              AEAEEEIEDMVRHAQYQWEVERHLCGLDIGPWWPEE---GEQEEDEYNVRRPRWEGLEEP    238
tr|A0A0N0DZ25|A0A0N0DZ25_LEPPY      EDFEDEIEELVKLAHNLSDIEYGLCGLPLAD--PTDAAASGEAEAVR--------DAIRP    262
tr|A4HKJ0|A4HKJ0_LEIBR              EEAEEEIEELVKTARTQAEVEYRLCGMAFPLNKSFPTLDGETKENEV--------SSAPP    262
tr|Q4Q576|Q4Q576_LEIMA              EEFEDEIESLVKSVRSLEETEHSLCGMTLPVNKPRTVLDGETEGDEV--------SSVPL    262
tr|A4I822|A4I822_LEIIN              EEFEDEIESLVKSVRSLEETEHSLCGMALPVNKPRAALDGETEGDEV--------SSVPL    262
                                    : :  :  :::  .   : :  **

tr|A0A0S4JVF1|A0A0S4JVF1_BODSA      ----CCGTKKVEASKEKERERREKWLELKKVKDEKIKARAEREKRKKEREEEEKKRREES    225
tr|Q381R4|Q381R4_TRYB2              VTAVCVGVEPSIEAEVRRLEKYEKWQASISKGTSQD-KMKGA---EGAES-VVVK-----    266
tr|A0A1X0NUB5|A0A1X0NUB5_9TRYP      ITSVCVGTEASMKAELGRIQRYEKWQEGFTKRREEAIRNKSN---KTEEP-VELK-----    263
tr|Q4CZ44|Q4CZ44_TRYCC              VTAVCIGTEASIAAELGRIQRYEKWQESISRRREEAIKKIGG---KSEEP-IVAK-----    343
tr|Q4D5S5|Q4D5S5_TRYCC              VTAVCIGTEASIAAELGRIQRYEKWQESISRRREEAIKKLGG---KSEEP-IVAK-----    349
tr|A0A3R7K0H2|A0A3R7K0H2_9TRYP      ITAVCIGTEASLAAELGRIHRYEQWQDTFAKRREEAIEKSGG---RSSEP-VVAK-----    267
tr|A0A422NB75|A0A422NB75_TRYRA      VTAVCIGTEASLNAELGRIQRYELWQDSISKRREEALKNIRE---ESDEP-VFAK-----    240
tr|S9UAW3|S9UAW3_9TRYP              VLQVCAASHPIAEDARKDQVLWQRWTDIRERKKQEAAVRHSR---RFLDA-EDE------    288
tr|A0A0N0DZ25|A0A0N0DZ25_LEPPY      ITNICALTEQLREAAHQDQLRWSQYMRREERRKERLEEKEKV---LKRDA-EEPA-----    313
tr|A4HKJ0|A4HKJ0_LEIBR              ITNVCADVEVLRAAARRDQERWEQYQRREAKRKVEIAQRMET---AADVA-GAAT-----    313
tr|Q4Q576|Q4Q576_LEIMA              ITNVCADAEVLRAAARRDQQRWEQYERREAERKAKLAQRRKL---ET-AA-AAAT-----    312
tr|A4I822|A4I822_LEIIN              ITNVCADVEVLRAAARRDQQRWEQYERREAKRKAKLDQRRKL---ET-TA-AAAT-----    312
                                             *   .            . :      .

tr|A0A0S4JVF1|A0A0S4JVF1_BODSA      CSTTVDYLLFHFIPFNLRVEHTW-------------------------------------    248
tr|Q381R4|Q381R4_TRYB2              ---GVQYSKDDPLSFTVLRKPNAKSNGDTTEGGSDAAE----DVRK--------QPDKDS    311
tr|A0A1X0NUB5|A0A1X0NUB5_9TRYP      ---PVNHSAN-PEPFFKMSDLTGGISDD-DDDEE--------------------------    292
tr|Q4CZ44|Q4CZ44_TRYCC              ---EVVHGEKDPQPWFSVSKSTFSSSDS-EEGDDDD-E----GGLE--------------    380
tr|Q4D5S5|Q4D5S5_TRYCC              ---EVVHGEKDPQPWFSVSESTFSSGDR-EEGDDDD-E----GGLE--------------    386
tr|A0A3R7K0H2|A0A3R7K0H2_9TRYP      ---PVEHNANDTKPWVLFPELGADADQD-GDGDGDAGE----GDTG--------------    305
tr|A0A422NB75|A0A422NB75_TRYRA      ---PVEPDDERYKPWSLFPEMDAATGED-GDIDD---S----DDTA--------------    275
tr|S9UAW3|S9UAW3_9TRYP              -----EL-----------------------------------------------------    290
tr|A0A0N0DZ25|A0A0N0DZ25_LEPPY      ------------EKTEVETQEGSSSSGNTGEHNGDKGPAKSEDVVNEGTARSDAAPEAAA    361
tr|A4HKJ0|A4HKJ0_LEIBR              ----SELASEASTNTEVAAALDRSTPAAAGKS------------VDSSTEKATNNP----    353
tr|Q4Q576|Q4Q576_LEIMA              ----SESADEAPANAEAAAALASSAPGASGEP------------IDSPAEEAPYSP----    352
tr|A4I822|A4I822_LEIIN              ----SEPAEEAPTNAEAAAALASSAPGAPGEP------------IDSPTEEAPYSS----    352


tr|A0A0S4JVF1|A0A0S4JVF1_BODSA      ---------------------    248
tr|Q381R4|Q381R4_TRYB2              V--------------FMNFDL    318
tr|A0A1X0NUB5|A0A1X0NUB5_9TRYP      -----------------DMDL    296
tr|Q4CZ44|Q4CZ44_TRYCC              ---------------SIGIDL    386
tr|Q4D5S5|Q4D5S5_TRYCC              ---------------SIGIDL    392
tr|A0A3R7K0H2|A0A3R7K0H2_9TRYP      ---------------ERGMDL    311
tr|A0A422NB75|A0A422NB75_TRYRA      ---------------ERNMDL    281
tr|S9UAW3|S9UAW3_9TRYP              ---------------------    290
tr|A0A0N0DZ25|A0A0N0DZ25_LEPPY      EEAGSAAEDTTPHGDVEEGDL    382
tr|A4HKJ0|A4HKJ0_LEIBR              --VGGG--DKEVSDNYTDGEL    370
tr|Q4Q576|Q4Q576_LEIMA              --VGGV--LKEAAEDRTDGDL    369
tr|A4I822|A4I822_LEIIN              --ADGA--VKEAAEDRTDGDL    369
```

(overall sequence identity = 0.046)

Animal

```
tr|Q4Q576|Q4Q576_LEIMA    MRIHSTFLLAPPQRHQRCCLLASVALLASLLLALPAVLAVTPPQGKPIRPLHGAGYEHLD    60
tr|A4I822|A4I822_LEIIN    MQRHSTFLLAPPQRHQQCCLLALVALLAILLLASPTVLAAPPPQGKPIRPLHGAGYEHLD    60
sp|Q9BT09|CNPY3_HUMAN     -------MDSMPEPASRCLLLLP--LLLLLLLLLPAPELGPSQAGAE---ENDWVRLPSK    48
sp|Q9Y2B0|CNPY2_HUMAN     ------------------MKGWG--WLALL----LGALLGT---------AWARRSQDLH    27
sp|Q7JXF7|SEELE_DROME     -------------MLTKALILFG--LLA----------LAQ---------GYSFTSREVK    26
                                                    *                                  .

tr|Q4Q576|Q4Q576_LEIMA    CSACITVARTLFERLNQTLAENPSTYLISHRLGKK----NQLRRRQYRNSELLVTEVMEN    116
```

```
tr|A4I822|A4I822_LEIIN    CSACITVARTLFERLNQTLAENPSTYLISHRLGKK----NQLRRRQYRNSELLVTEVMEN    116
sp|Q9BT09|CNPY3_HUMAN     CEVCKYVAVELKSAFEETG---KTKEVIGTGYGILDQ---KASGVKYTKSDLRLIEVTET    102
sp|Q9Y2B0|CNPY2_HUMAN     CGACRALVDELEWEIAQVD---PKKTIQMGSFRINPDGSQSVVEVPYARSEAHLTELLEE    84
sp|Q7JXF7|SEELE_DROME     CHVCKAVVTELEEAIAKED---PHKMADVSGFRLDAQGNSISKKVRLVKSEMFLTELMEK    83
                          * .*  :.  *   : :         .                         .*:  : *: *

tr|Q4Q576|Q4Q576_LEIMA    VCTSYKNDARLLRLHPKSKVRLYHQQVFGDTRLGVRHALREDEVYPADADPAAWAEKLDG    176
tr|A4I822|A4I822_LEIIN    VCTSYKNDARLLRLHPKSKVRLYHQQVFGDTRLGVRHALREDEVYPADADSAAWAEKLDG    176
sp|Q9BT09|CNPY3_HUMAN     ICKRLLDYSLHKERT---GSNRFAKGM-SETFETLHNLVHKGVKVVMDIPYELWNET---    155
sp|Q9Y2B0|CNPY2_HUMAN     ICDRMKEYGEQIDPS---THRK----------NYVRVVGRNGESS--ELDLQGIRID---    126
sp|Q7JXF7|SEELE_DROME     ICEKMDDYLKATYKS---NGKF----------TLLKMIINGQMNP--DSSLVDFVQD---    125
                          :*       :                .                ::   .           :

tr|Q4Q576|Q4Q576_LEIMA    QLYPIARLYSRRDADALQGLQSLSATPTMCALLVEEFEDEIESLVKSVRSLEETEHSLCG    236
tr|A4I822|A4I822_LEIIN    QLYPIARLYSRQDADALQGLQSLSATPTMCALLVEEFEDEIESLVKSVRSLEETEHSLCG    236
sp|Q9BT09|CNPY3_HUMAN     -------------S------AEVADLKKQCDVLVEEFEEVIEDWYRNH-QEEDLTEFLCA    195
sp|Q9Y2B0|CNPY2_HUMAN     -------------S------DISGTLKFACESIVEEYEDELIEFFSRE--ADNVKDKLCS    165
sp|Q7JXF7|SEELE_DROME     -------------G------DLNKSLGHFCNEVLEDNDEIFVKAFQAEELGNDLDIKICS    166
                                           .                *  ::*: :: : .            ::       :*.

tr|Q4Q576|Q4Q576_LEIMA    MTLPVNKPRTVLDG-ETEGDEVSSVPLITNVCADAEVLRAAARRDQQRWEQYERREAERK    295
tr|A4I822|A4I822_LEIIN    MALPVNKPRAALDG-ETEGDEVSSVPLITNVCADVEVLRAAARRDQQRWEQYERREAKRK    295
sp|Q9BT09|CNPY3_HUMAN     NHVLKGKDTSCLA-EQWSGKKGDTAALGGKK----------SKKKSSR----AKAAGGRS    240
sp|Q9Y2B0|CNPY2_HUMAN     KRTDLCDHALHISHDEL-------------------------------------------    182
sp|Q7JXF7|SEELE_DROME     EQASYCDESPVQEEYDFDGKEEL-------------------------------------    189
                                  .        :

tr|Q4Q576|Q4Q576_LEIMA    AKLAQRRKLETAAAAATSESADEAPANAEAAAALASSAPGASGEPIDSPAEEAPYSPVGG    355
tr|A4I822|A4I822_LEIIN    AKLDQRRKLETTAAAATSEPAEEAPTNAEAAAALASSAPGAPGEPIDSPTEEAPYSSADG    355
sp|Q9BT09|CNPY3_HUMAN     SSSKQRKELGGLE-------GDPSPEEDE-------------GIQKASPLTHSPPDEL--    278
sp|Q9Y2B0|CNPY2_HUMAN     ------------------------------------------------------------    182
sp|Q7JXF7|SEELE_DROME     ------------------------------------------------------------    189


tr|Q4Q576|Q4Q576_LEIMA    VLKEAAEDRTDGDL    369
tr|A4I822|A4I822_LEIIN    AVKEAAEDRTDGDL    369
sp|Q9BT09|CNPY3_HUMAN     --------------    278
sp|Q9Y2B0|CNPY2_HUMAN     --------------    182
sp|Q7JXF7|SEELE_DROME     --------------    189
```

02. Sensitive to salt 1 / Saponin B (SES1 / SESSAB)

This obscure group does have homologs beyond Kinetoplastida: Many related sequences can be retrieved from green algae and higher plants (including A thaliana as well). All these homologs have a well-visible KDEL signal on them. Interestingly, a few animals also possess such proteins, but only among cnidarians, e.g. Hydra vulgaris (they might also have been acquired it from algae by horizontal gene transfer, as the gene is otherwise lacking from all other animals or even fungi) Seredipitiously, the Arabidopsis thaliana ortholog has already been studied experimentally (PMID:30287478). Because of the phenotype of the mutant, the gene was baptized SENSITIVE TO SALT 1 (SES1). Despite the presence of a Saponin B domain, the SES1/SESSAB proteins are rather different from Canopy/CARSAB family members. Both are suggested to be ER-localized chaperones serving different roles.

```
tr|S9W4D1|S9W4D1_9TRYP              -----------------------MKHLIHLLALCVGFLFLVCDVSGKKDLMLPT--PLPS    35
tr|S9UQQ8|S9UQQ8_9TRYP              -----------------------MFTPI----VLLLLLFSHGVASDGVEVIVPT--SLSP    31
tr|A0A0N0VFN2|A0A0N0VFN2_LEPPY      ---------------MHTMQLRVATPLLLVLCV-LVSSVGLERATARKELPLNT--AFPP    42
tr|A4HQ81|A4HQ81_LEIBR              -------MPCNPRSYVSSTQPLVTAALLLLVCV-LAVATG--CASARKELPLNI--PYPP    48
tr|Q4Q0J6|Q4Q0J6_LEIMA              ------MPCCSPPSHVAPTRPLVTTTLLLLVCV-LAAALG--CASARKQLPLNT--PYPP    49
tr|A4IDX9|A4IDX9_LEIIN              ------MPCCSPPSHVAPTRPLVTSALLLLVCV-LAAALG--CASARKQLPLNT--PYPP    49
tr|A0A0S4IVP5|A0A0S4IVP5_BODSA      ------------------M-----STCVWVIGV-VLWLFVYHAVSAKKEKLTFN--SYGP    34
tr|Q38A59|Q38A59_TRYB2              -------------MGKAPV-----KG--VFLVL-VALVPMFPAVDAKKEKPMNV--PFPQ    37
tr|A0A1X0P0S9|A0A1X0P0S9_9TRYP      ------------------M-----RSLMYFVLL-ISAATLLTLTNAKKEKPLNE--PFPK    34
tr|Q4CTW1|Q4CTW1_TRYCC              MDRIKVEMKSKQTREGRPM-----RFIKLCIFF-AVTLLFSAAGVAKKEKPPHA--PFPK    52
tr|Q4CWS6|Q4CWS6_TRYCC              MDRIKVEMKSKQTREGRPM-----RFLKLCIFF-AVTLLFSEAGVAKKEKPPHA--PLPK    52
tr|A0A422PSY9|A0A422PSY9_9TRYP      ------------------M-----RTFVVSLFI-LVTLLLSSAIDARKEKPLHD--AFPK    34
tr|A0A3R7NVI0|A0A3R7NVI0_TRYRA      ------------MRKDHQM-----RTFIITVCI-LVVLLFSSAVAAKKEKPLHI--PFPK    40
tr|A0A8J4C7T8|A0A8J4C7T8_9CHLO      --------------HFHIC----MMVRLLYV-LLFAFIAVASAQFGGPKRPKTK-APVNR    40
tr|A8J2L8|A8J2L8_CHLRE              ------------------M----ARLNLLQLCVLGLCLAVASAQFGGKRPPKPK-APVAK    37
tr|A0A150GFK4|A0A150GFK4_GONPE      ------------------M----KRNNLLRLLLLALV-VAASAQFGGPKRPKKK-SPVAK    36
tr|Q5VML4|Q5VML4_ORYSJ              ---------MARGGGVAVA----MAVAVAAVV-----LLLHPAASAAAAGPKKVATAARK    42
tr|A0A8B6XU83|A0A8B6XU83_HYDVU      ------------------M----IGIEICTAY-----LLIHFG-FGISQMPNKSPILPIV    32


tr|S9W4D1|S9W4D1_9TRYP              EVQAILNCDLCAYTLINAVLRVHEVQDALEAQELRLKEDDILS-VLETLCQPYTEYGQWM    94
```

```
tr|S9UQQ8|S9UQQ8_9TRYP            VVSEAFACEICGFTVMYALMAVSEKTAELEKADLVINEDDILS-LFEDICNPFMEKGEWI     90
tr|A0A0N0VFN2|A0A0N0VFN2_LEPPY    VVQDALRCDVCAFIVANSLNHVEAKRDELRAKRLALREDDVLE-ETENLCIPFKDQGQWI     101
tr|A4HQ81|A4HQ81_LEIBR            IVQNALRCNVCSFIVDNALIYVQGMRDEMSQHRLQLREDDVLD-ELEKLCVPFKDPGQWI     107
tr|Q4Q0J6|Q4Q0J6_LEIMA            VVQNALRCDVCSFIVVNSLYQIEAKREEQQRKRLQVREDDVLE-EVENMCVPFKDQGQWI     108
tr|A4IDX9|A4IDX9_LEIIN            VVQDALRCDVCSFIVVNALYQVEAKREEQQRKRLQVREDDVLE-EVENMCVPFKDQGQWI     108
tr|A0A0S4IVP5|A0A0S4IVP5_BODSA    AVERRHSCEICRTLVVEAYPIAEELHKNASRRGYALREEEVLSKVTEAICNPFVKAGQWI     94
tr|Q38A59|Q38A59_TRYB2            DAIDDVRCDVCRLMAKRAYGYVYELFVTSEHTRIPVNEENVLV-AIEDICNPLAASGQWI     96
tr|A0A1X0P0S9|A0A1X0P0S9_9TRYP    DAIDAISCDICSLLVRKTYLDVQALFAASVETRVRVNEDDILT-AIEDVCNPFAETGQWI     93
tr|Q4CTW1|Q4CTW1_TRYCC            DVVNAISCGVCNFMVGKAYGDVQLLVNASVQTRVRVNEDDVLT-AIEDLCNPFSDVGQWI     111
tr|Q4CWS6|Q4CWS6_TRYCC            DVVDAISCGVCNFMVGKAYGDVQLLFNASVQTRVRVNEDDVLT-AIEDVCNPFSDVGQWI     111
tr|A0A422PSY9|A0A422PSY9_9TRYP    EVIDGIRCGVCNFAVKQAYGNVLGLFNASIQTRVRMNEEDVLT-VLEDICNPFTEVGQWI     93
tr|A0A3R7NVI0|A0A3R7NVI0_TRYRA    DAVDGISCGVCTFVVKQVYRDVLVLFNASIRRRVRMSEDDVLT-ALEDVCNPFAETGQWI     99
tr|A0A8J4C7T8|A0A8J4C7T8_9CHLO    DDIKYIKCKVCEAMAKEARN-VVKELGEL-AGAKKINEADILE-RLEKMCNPDLNEGDWI     97
tr|A8J2L8|A8J2L8_CHLRE            GDIKYIKCQVCEAIAKQSIK-ATKDLIEQ-AGPKKVPEADILE-RVEKMCDPDTDDGEWI     94
tr|A0A150GFK4|A0A150GFK4_GONPE    DDIKYIKCQVCEAIAKQARN-IKKDLQAV-AGPKKVSEGDLLD-KFEKMCDPEQDDGDWI     93
tr|Q5VML4|Q5VML4_ORYSJ            EDIPYIRCQVCERIAREISAQVAKKQQAL-PATKKVPEIEIIE-IAENVCNLKKQEADWM     100
tr|A0A8B6XU83|A0A8B6XU83_HYDVU    EDLPFIKCDVCQKAAKVLFKTIEN----Q-RSEKKLDEDDVLS-IVEKSCDTDVLYGDWI     86
                                           * :*                      : * :::  *  *     .:*:

tr|S9W4D1|S9W4D1_9TRYP            RRVSIQLQADTHS---------EGKEKMLQTTVLPYVSTCKRTCSTTQAVCEQVLDSSEM     145
tr|S9UQQ8|S9UQQ8_9TRYP            RRAELTLDEKKR---------------TVSFNIGNNYTKCKRFCGTFSNVCEQVYDSDEA     135
tr|A0A0N0VFN2|A0A0N0VFN2_LEPPY    RQVSLQVAPKSS--------QDASEGHQMEVGVVNFYGRCGRTCDTVAALCEELMDRDVM     153
tr|A4HQ81|A4HQ81_LEIBR            RQVSLRVDETKVC-RNADTSVIEAPRQVMRVALVEYYSKCGRTCDTVALLCEEWMDSSYM     166
tr|Q4Q0J6|Q4Q0J6_LEIMA            RQVALQVEEVAPSERGTDRPAAATPRHHMSVGVVDYYSKCGRICETVAVLCEEWMDSGYM     168
tr|A4IDX9|A4IDX9_LEIIN            RQVALQMEEVAPSKRGADRPAAATPRYHMSVGVVDYYSKCGRICETVAVLCEEWMDSGYM     168
tr|A0A0S4IVP5|A0A0S4IVP5_BODSA    RLVHIKANE---------------KTLQTVLEPLEFYSQCKRDCTTVANSCSSVVDTDYG     139
tr|Q38A59|Q38A59_TRYB2            RQVAVDTSSP----------AAAKGPFHITLTELPHHSKCKRTCKTLSDACEAVVDDDDM     146
tr|A0A1X0P0S9|A0A1X0P0S9_9TRYP    RRVAINYSA-------------MTAPFLLGIEELPVFTKCKRTCTTIVEACEAIMDHDSM     140
tr|Q4CTW1|Q4CTW1_TRYCC            RQITITHES-------------DTAPFLLGVSRLPAYAKCKRVCSTVVEACEAVMDHENM     158
tr|Q4CWS6|Q4CWS6_TRYCC            RQITITHES-------------DTAPFLLGVSRLPAYAKCKRVCSTVVEACEAVMDHKNM     158
tr|A0A422PSY9|A0A422PSY9_9TRYP    RRITITHRR-------------DTDHL-LGVEELPVYTKCKRTCSTVVEACEAVMDHESM     139
tr|A0A3R7NVI0|A0A3R7NVI0_TRYRA    RRITITHKR-------------ETAPF-LGVEELQVYTKCKRTCSTVVEACEGVLDHESM     145
tr|A0A8J4C7T8|A0A8J4C7T8_9CHLO    AMYDIVKEGD-----------------ILNLKDTGMVGRCKSKCRTIARTCELIAEDLDL     140
tr|A8J2L8|A8J2L8_CHLRE            TKYDIVEEDS-----------------ALVLKDTGAVGRCKSECRTIARACEMISDDVDL     137
tr|A0A150GFK4|A0A150GFK4_GONPE    TQFDIVEEGS-----------------ALKLKDTGMMGKCKSECRTIAYACEKILDDVDL     136
tr|Q5VML4|Q5VML4_ORYSJ            LKIDIVEKGD-----------------KLELVEQDEEGHCNAECKTIERACQEVMGYAD-     142
tr|A0A8B6XU83|A0A8B6XU83_HYDVU    SRLDIVEKEN-----------------DLKVVEHPQEGKCDRECKTISRSCEDIIGDID-     128
                                           :                                 *   * * *.

tr|S9W4D1|S9W4D1_9TRYP            DDFSAAVLKLSQRHKTIAEPSVWEPLITQTCHQM-DMCLDATNKRNAFIRQYRKDPAVYQ     204
tr|S9UQQ8|S9UQQ8_9TRYP            TNLAADMLKLIKKRVGLVKTNEAEEIVNKVCKK--EFCEHKERNMAALHQMLAKDDNLWG     193
tr|A0A0N0VFN2|A0A0N0VFN2_LEPPY    DDFPGQLLKVSK-DANMADEAHRDAVFHQFCYAS-TYCKLHQKYVVALEKELRTNVNL-L     210
tr|A4HQ81|A4HQ81_LEIBR            DDFPSYLVREANSGYNISSEEHRSAVFSQFCAPT-PHCENIGSALRELSEELTKNSEL-R     224
tr|Q4Q0J6|Q4Q0J6_LEIMA            DGFSSRLVKEANAGRSISDAPHRDAVFDSFCAPS-PHCKKHAIFVRELDLALTTDTEL-R     226
tr|A4IDX9|A4IDX9_LEIIN            DGFSSRLVKEAKAGRNISDAAHRDVVFGSFCAPS-THCKKHATFVRKLDMALTKDTEL-R     226
tr|A0A0S4IVP5|A0A0S4IVP5_BODSA    DDLPGLLLKLKPV----------EVIDEAVCKRI-CIDTTAV---RTKPLSA----KM-I     180
tr|Q38A59|Q38A59_TRYB2            DQFSSKLLILKKY-------KDASALADATCGVS-AFCTSR------TGFSSKRYKEL-L     191
tr|A0A1X0P0S9|A0A1X0P0S9_9TRYP    DQLTPKLLHLPEY-------ADGETLARSLCQTS-PICTKR------KSLSAERYAEL-R     185
tr|Q4CTW1|Q4CTW1_TRYCC            DQLSPRLLRLNEY-------AGAEAFANALCLPS-SICTER------QGLSAARYEEV-R     203
tr|Q4CWS6|Q4CWS6_TRYCC            DQLSPRLLRLNEY-------AGAEAFANALCQSS-SICTER------QGLSAARYEEV-R     203
tr|A0A422PSY9|A0A422PSY9_9TRYP    DTLSPRLLHLTEY-------ADAGTFAQALCGPS-PICTKR------RRLLADRYDEL-V     184
tr|A0A3R7NVI0|A0A3R7NVI0_TRYRA    DMLSPRLLHLTEY-------ADADKFAEALCDRS-PICTKR------WGLTASRYDEL-T     190
tr|A0A8J4C7T8|A0A8J4C7T8_9CHLO    TDLSAMLFKGK-K---------RSAVTNWMCYDATDVCISKPPP-------------VPA     177
tr|A8J2L8|A8J2L8_CHLRE            TDLSAMLFKGK-K---------RAAISNWMCHDASDACSKKAPA-------------LTK     174
tr|A0A150GFK4|A0A150GFK4_GONPE    TELSSLVYAGK-K---------RAAVTNWLCHESSDACESKPPP-------------VPK     173
tr|Q5VML4|Q5VML4_ORYSJ            TDVAEFVYKKKPS---------ADQLVKFLCKDLSEACVVDPPP-------------VPK     180
tr|A0A8B6XU83|A0A8B6XU83_HYDVU    TDIGELLWKNEMK---------LATFINHVCYKLTNSCKAKKKY-------------VKG     166
                                  .   :                     .        *                        :

tr|S9W4D1|S9W4D1_9TRYP            KLIEDEAPAKLDEDEIEVEQMMDNFNRVD-----GKKTSVFSREEMVELQSALLRGDKKA     259
tr|S9UQQ8|S9UQQ8_9TRYP            TLVEDKS-EPADHDRIEVENLIAKME-RD-----GKRSNVYSRDHVKKLQAAVKQGDRRA     246
tr|A0A0N0VFN2|A0A0N0VFN2_LEPPY    EEMKADKPRRMKKEELEMETMMYRLMREQ-----RQSADVFSRDEIRKMQQAFIHGTKED     265
tr|A4HQ81|A4HQ81_LEIBR            EAIDADRPQEIKSEEREMEMMLHRLTREH-----GQSADVFSRDEVHRMKEAFLKGNREE     279
tr|Q4Q0J6|Q4Q0J6_LEIMA            ASIDADRPQEIETEEREMETMLHRLTREQ-----GQSADVFSRDEIRRMKEAFVTGNKED     281
tr|A4IDX9|A4IDX9_LEIIN            ESIDADRPQEIETEEREMETMLHRLTREQ-----GQSADVFSRDEIRRMKEAFVTGNKED     281
tr|A0A0S4IVP5|A0A0S4IVP5_BODSA    KEIAAEVPVEIDKKNLDIEEIMDDMERARGGYGGAPKMDVFSRDEMMDVQRAILERDGSK     240
tr|Q38A59|Q38A59_TRYB2            TLVASDVVEVIEQKEMDIERFMDEMERKG-----NRRQEIYSREEVIKMQKSLIEGDIET     246
tr|A0A1X0P0S9|A0A1X0P0S9_9TRYP    TMIKADVMEEIDPKEMEVERMMDQMERKE-----NRRHDIFSREEITAMQQGLLRGDREA     240
tr|Q4CTW1|Q4CTW1_TRYCC            NMIDEDTVEEIDPKEMEIERMMDHMERKE-----NRRQTIFSRDEITSMQKAFLRGDKEA     258
tr|Q4CWS6|Q4CWS6_TRYCC            NMIDEDTVEEIDPKEMEIERMMDHMERKE-----NRRQTIFSRDEITSMQKAFLRGDKEA     258
tr|A0A422PSY9|A0A422PSY9_9TRYP    TMMDADPMEEIDPKEMEVERMMDHMERKE-----NRRQTIFSREEITKMQEAFLRGDKEA     239
tr|A0A3R7NVI0|A0A3R7NVI0_TRYRA    TMIDEDTMEEIDPKEMEVERMMDHMERKE-----NRRHSIFYRDEIVKMQEAILRGDKEA     245
tr|A0A8J4C7T8|A0A8J4C7T8_9CHLO    DRPVGEPHEPLDEDEYRNIMMMRDMEAIG----V--SGSLYSRDTLTEELEEMQDMYGDD     231
tr|A8J2L8|A8J2L8_CHLRE            REGADEAHAPMDEDELRTERMMRSMKAAG----L--SGTMYNKETMQEELAEMADQYEDN     228
tr|A0A150GFK4|A0A150GFK4_GONPE    GRRADERHVPMSEDEVRTEKMLRNMKAAG----L--SGSMYSRETLEEELAEMAGEYSDD     227
tr|Q5VML4|Q5VML4_ORYSJ            DRVPGEPFAAKPSKDAEMDRILKSMEGIP----GAPSMKMYSRDDLMK--NNFGVDGDDD     234
tr|A0A8B6XU83|A0A8B6XU83_HYDVU    S-HKDYKFVEMSEKEKQARDLMRNMKSVP----GMPGMEMYSQDDLQNMREQMGVRNEDE     221
                                                   .        ::  :            :: :: :          .

tr|S9W4D1|S9W4D1_9TRYP            AARIDPSIVDLSDEEFESIQNLAAKQGYVRGGRG-DGEKEGLFHMTDDES----------     308
tr|S9UQQ8|S9UQQ8_9TRYP            ATKLDPTIRSLSDEEFEEVVRMAREEYNTDT-L-----EEDL------------------     282
tr|A0A0N0VFN2|A0A0N0VFN2_LEPPY    VAAVDPKAFDLSDEEFVALREHMRADERVTGGNR-RGQKQEGDED-GD------------     311
tr|A4HQ81|A4HQ81_LEIBR            LKAIDPAAFDLTDSEFSMLQNYIRGEAPEEKEWE-QQRGSSNPSDAED------------     326
tr|Q4Q0J6|Q4Q0J6_LEIMA            LQAVDPTAFDLTDDEFSTLKDYMHGEERKQQQQQ-RQRRPSAPSDADD------------     328
tr|A4IDX9|A4IDX9_LEIIN            LQAVDPTAFDLTDDEFSTLQDYMRGEERKQQ--Q-RQRRPSAPSDADD------------     326
tr|A0A0S4IVP5|A0A0S4IVP5_BODSA    LRDLDPSAEDLSDEELEYLQRMYAGEGDGGEEYP-TELGEDL------------------     281
tr|Q38A59|Q38A59_TRYB2            AAGIDPSLNKLTEEEQLALQQMLKNKPMGKKRPP-EDAGQAASPEGDS-AEVNPDL----     300
```

```
tr|A0A1X0P0S9|A0A1X0P0S9_9TRYP    VAKIDPSIMDLNNEEFAALQAMIRGKMPASQEKV-TGQQQGQSSDNMNSIGMDQDIYNGA    299
tr|Q4CTW1|Q4CTW1_TRYCC            VAQVDPSIMDLSDEEFSALQSMIRGRAMGQEQPE-ENGDDGEWKGRNK----NYPMYHSA    313
tr|Q4CWS6|Q4CWS6_TRYCC            VAQVDPSIMDLSDEDFSALQSMIRGRAMGQEQPE-ENGDDGEWKGRNK----NYPMYHST    313
tr|A0A422PSY9|A0A422PSY9_9TRYP    VAKVDPSIMDLSEDEFAAVQAMMRGRKDGKPQAG-ETDADGEAKVRRT----QRRKRRES    294
tr|A0A3R7NVI0|A0A3R7NVI0_TRYRA    VAKVDPSIADLSEEEFAAVQAMVRGKNNEKLRSG-THRADDEGKARDT----QRRKHRES    300
tr|A0A8J4C7T8|A0A8J4C7T8_9CHLO    P-------------DFAQV---MKDTGMDSFASRKPDEEPTSGETAVDSST---------    266
tr|A8J2L8|A8J2L8_CHLRE            E-------------DFARM---LEETGLDKHMPSRPGSDAQEPDSGVGGG--------AA    264
tr|A0A150GFK4|A0A150GFK4_GONPE    P-------------DFAKA---MADSGLDKHLQKGTEEEPTAADATAEQPSVASSVANAA    271
tr|Q5VML4|Q5VML4_ORYSJ            D-----D-DEDEDDDFPKN---LGKVFKDK------------------------------    255
tr|A0A8B6XU83|A0A8B6XU83_HYDVU    N-----Q-QD---SEQSEN---YDEVYRTK------------------------------    239
                                                   :

tr|S9W4D1|S9W4D1_9TRYP            ------------------------------------------------------DL    310
tr|S9UQQ8|S9UQQ8_9TRYP            --------------------------------------------------------    282
tr|A0A0N0VFN2|A0A0N0VFN2_LEPPY    -----------------L--------------------------------------    312
tr|A4HQ81|A4HQ81_LEIBR            -----------------L--------------------------------------    327
tr|Q4Q0J6|Q4Q0J6_LEIMA            -----------------L--------------------------------------    329
tr|A4IDX9|A4IDX9_LEIIN            -----------------L--------------------------------------    327
tr|A0A0S4IVP5|A0A0S4IVP5_BODSA    --------------------------------------------------------    281
tr|Q38A59|Q38A59_TRYB2            --------------------------------------------------------    300
tr|A0A1X0P0S9|A0A1X0P0S9_9TRYP    DYGNDE----------DSM-------------------------------IMDDDL    314
tr|Q4CTW1|Q4CTW1_TRYCC            DNGNDDGE--------NLN-------------------------------LEDEDF    330
tr|Q4CWS6|Q4CWS6_TRYCC            DNGNDDGG--------NLN-------------------------------LEDEDF    330
tr|A0A422PSY9|A0A422PSY9_9TRYP    GGGEDDWE--------DVG-------------------------------TGESDL    311
tr|A0A3R7NVI0|A0A3R7NVI0_TRYRA    DGGEGDLE--------NFE-------------------------------LEQEDL    317
tr|A0A8J4C7T8|A0A8J4C7T8_9CHLO    ---MTKLQETAAKAAGDIKDGA---AKLVEGAKKFMGKLFGGSKQDG--KSGEL--    312
tr|A8J2L8|A8J2L8_CHLRE            AGAVADAAARLQDTLTQVKEGA---SKLFDSAKGLVGKWFGKKEAAK--KPAGEL-    314
tr|A0A150GFK4|A0A150GFK4_GONPE    AGAASSLTEAASKAAESIKEGA---AKVVEGAKNVVGKLFGGGGKQE--EAGGNEL    322
tr|Q5VML4|Q5VML4_ORYSJ            GSPKKDLKQQ---VVKQIKDTGKKLKGHVNKVSKVVKKWWQGKKKPS--KSSKTEL    306
tr|A0A8B6XU83|A0A8B6XU83_HYDVU    LGFFET-------------------------LKFFLNDLWKKIKYLFSYISYKKEL    270
```

(overall sequence identity = 0.0265)

03. Chitinase with ER retention signal

These proteins are found in kinetoplastids with ER retention (KDEL) signals, and have a lot of homologs across other eukaryotes. However, most multicellular organisms (animals, plants, fungi) have secreted chitinases without any retention signal. The fact that chitinases from some green algae and other plant-like unicellular eukaryotes do have KDEL signal, shows that this feature is not a kinetoplastid innovation. Nevertheless, the role of such a glycosidase in the ER is unclear (externally digested, e.g. insect chitin is unlikely to reach the ER), but it likely processes endogenous glycans. Technically, these glycosidases can also be called Glycohydrolase-18 (PFAM/Interpro label).

```
tr|A0A0S4J833|A0A0S4J833_BODSA    ------------------------------------------------------------    0
tr|A0A250X009|A0A250X009_9CHLO    ------------------------------------------------------------    0
tr|C5K6G1|C5K6G1_PERM5            ------------------------------------------------------------    0
tr|C1N616|C1N616_MICPC            ---------------------------------------------------------MAS    3
tr|A0A7G2CNI0|A0A7G2CNI0_9TRYP    ------------------------------------------------------------    0
tr|S9TUR0|S9TUR0_9TRYP            --------------------MTFVSLSTTT----------QG-ILYRSTLFSFLFNFLCI    29
tr|A0A0M9FXG9|A0A0M9FXG9_LEPPY    ---------------------------------------------------------MCP    3
tr|A4H8K3|A4H8K3_LEIBR            ---------------------------------------------------------MEP    3
tr|A4HWX6|A4HWX6_LEIIN            ---------------------------------------------------------MVQ    3
tr|Q4QEU0|Q4QEU0_LEIMA            ---------------------------------------------------------MVQ    3
sp|O81862-2|CHIC_ARATH            ------------------------------------------------------------    0
tr|S8AUV1|S8AUV1_PENO1            ------------------------------------------------------------    0
tr|A0A179UX46|A0A179UX46_BLAGS    MSTYTVASGDSMWAISVARGISLDALIAANPQVSVPSQIEVGQVLNIPGGDAPADPFPAP    60
tr|M9NG70|M9NG70_DROME            -----------------------------------------------------MLPFR--    5
tr|Q22468|Q22468_CAEEL            ---------------------------------------------------------M--    1

tr|A0A0S4J833|A0A0S4J833_BODSA    ----MATVETIALVTITVLLLLSASTTPVT-----------------------AETTPEA    33
tr|A0A250X009|A0A250X009_9CHLO    ------------------------------------------------------------    0
tr|C5K6G1|C5K6G1_PERM5            -------MARLACLL-LLLLS--A--------------------------F---TVIADE    21
tr|C1N616|C1N616_MICPC            RGGGAGATARLALLL-VVLLLLPGRGAPDTAP---------------SSSFASPSPGAPA    47
tr|A0A7G2CNI0|A0A7G2CNI0_9TRYP    -------MKPTYILLCFVLLFCSCSGT-----------------------------SVPP    24
tr|S9TUR0|S9TUR0_9TRYP            MQHSWSSRRSAPAMVCTLLLLLLCSRLVLPTAAGSHS---NPEEEQRIRAAAEAYDVTDP    86
tr|A0A0M9FXG9|A0A0M9FXG9_LEPPY    SGKCRDSGNHAASFLLVLTAALLCIGSLPTEAQRSEPPSVRTAPATTDAARSSSSSNSSA    63
tr|A4H8K3|A4H8K3_LEIBR            RR------ELVLALCLAVVLHLSCLGAPLSTTVAAAASAAVRDSAARSSSQNASITTASP    57
tr|A4HWX6|A4HWX6_LEIIN            RS------ALLQLACLVAVLHSSCLSALLSSTIAAAAPAAARDTAISSR-HNTPITVASP    56
tr|Q4QEU0|Q4QEU0_LEIMA            RS------ALVRLACLVAVLYSSCLSAPLSSTIAAAAPAAARDTAISSR-HNTSVTAASP    56
sp|O81862-2|CHIC_ARATH            ------------------------------------------------------------    0
tr|S8AUV1|S8AUV1_PENO1            -----------------------------------------------------------M    1
tr|A0A179UX46|A0A179UX46_BLAGS    ----------TANVPPPQEVFTLLSGGVPPPVPAAAA---------VQPSLPPPVGPSPG    101
```

```
tr|M9NG70|M9NG70_DROME              ------------RGAAWQTLFLLCA---------------------LA-Y-----CINEA    26
tr|Q22468|Q22468_CAEEL              ------------RFVMWMWLFVVWI---------------------TVLFGVSNGGILDR    28


tr|A0A0S4J833|A0A0S4J833_BODSA      AQPFTVFGYLPEYRLR------------GYNYTAAFQTGLTHLIYFSLEVDART--FLP-    78
tr|A0A250X009|A0A250X009_9CHLO      ---------MPEYRLGS-----------NYNYEEAFKNGLTHVIFFSLEVSTSD--FIP-    37
tr|C5K6G1|C5K6G1_PERM5              QERFRVFGYLPEYRLSD-----------DVDFDGIFSHGVTDLIFFSVEVSYLG--II--    66
tr|C1N616|C1N616_MICPC              SSPFAVVAYLPEWRFAS-----------T-DWDA-VCVNVTHLILFSLEVTDDG--SL--    90
tr|A0A7G2CNI0|A0A7G2CNI0_9TRYP      PQPFNVFGYLPEYRANK-----------LFPYETFFANGLTHLIFFSVEINNST--LGID    71
tr|S9TUR0|S9TUR0_9TRYP              GRPFAVFGYLPEYRHQSYGADTCGRHKFYFPYEEFFTRGLTHLIFFSLEADPST--LRLA    144
tr|A0A0M9FXG9|A0A0M9FXG9_LEPPY      APPFAVFGYLPEYRQL------------RFDYEAFFRSGLTHLIFFSAEVDPTS--LQLT    109
tr|A4H8K3|A4H8K3_LEIBR              L-PFAVFGYLPEYRQS------------WFNYEAFFKSGLTHLIFFSAEVDPAT--LRLA    102
tr|A4HWX6|A4HWX6_LEIIN              SPLFTVFGYLPEYRQG------------TFNYEAFFKAGLTHLIFFSAEVDPAT--LRLI    102
tr|Q4QEU0|Q4QEU0_LEIMA              SPLFTVFGYLPEYRQG------------SFNYEAFFKAGLTHLIFFSAEVDPAT--LRLI    102
sp|O81862-2|CHIC_ARATH              ------------------------------------MSSTKLISLIVSIT--------FFLN    18
tr|S8AUV1|S8AUV1_PENO1              SAGLKSIAYFVNWAIYGR----------NYNPQDLPAGKLTHVLYSFANIHPETGEVYLT    51
tr|A0A179UX46|A0A179UX46_BLAGS      SEGFRTVGYFTNWGIYGR----------NYQPMDIPGNYITHILYSFANVRPDSGEVYLT    151
tr|M9NG70|M9NG70_DROME              SSEGRVVCYYTNWSVYRPGT-------AKFNPQNINPYLCTHLVYAFGGFTKDN-QMKPF    78
tr|Q22468|Q22468_CAEEL              SCGRRRVGYITSWGKHPF------------RD--DQAEKLTHLVFAFFVVDSDG-SVKLE    73
                                                                               ::

tr|A0A0S4J833|A0A0S4J833_BODSA      ----KAKDRLPTMIEAK-----------QAREAA-DAVGGKIILSFGGN-ARSNGFAEMV    121
tr|A0A250X009|A0A250X009_9CHLO      ----SALDRLPPPDILK-----------RARAAA-DAYGGKLLLCFGGN-SRTGGFPGMV    80
tr|C5K6G1|C5K6G1_PERM5              ----QQKQRLPGPAILE-----------KARAAA-DAYGGRLLVSIGGA-GRSSGFADAV    109
tr|C1N616|C1N616_MICPC              ----TSLDRFPSPEAMT-----------TLRAAA-AKRGVKLLLSLGGH-GRTRGFPIVA    133
tr|A0A7G2CNI0|A0A7G2CNI0_9TRYP      ----YIEERLPPMEEWN-----------KIRELA-DKYNVKLMVCVGGS-DRSEAYPTLV    114
tr|S9TUR0|S9TUR0_9TRYP              ----R-EDRLPRATEWA-----------MVRALA-DQHNVKLMVSIGGG-GRSSGFGPLV    186
tr|A0A0M9FXG9|A0A0M9FXG9_LEPPY      ----HVDDRLPSREKWK-----------TLRELA-DTYGVKLLLCIGGG-GRSAGFPRLV    152
tr|A4H8K3|A4H8K3_LEIBR              ----HVDDRLPSDGEWA-----------RIRRLA-DLHGAKLMLCIGGG-GRSSGFSDLV    145
tr|A4HWX6|A4HWX6_LEIIN              ----HVDDRLPSGDEWA-----------RIRRLA-DLHGVKLMLCIGGG-GRSAGFADLV    145
tr|Q4QEU0|Q4QEU0_LEIMA              ----HVDDRLPSGDEWA-----------RIRRLA-DLHGAKLMLCIGGG-GRSAGFADLV    145
sp|O81862-2|CHIC_ARATH              SQTNQV----T-VSSANQP---KFSTF--TQTVQRRNPSVKTLLSIGGGIADKTAYASMA    68
tr|S8AUV1|S8AUV1_PENO1              DSWSDVEKHYA-GDSWNDTGNNVYGCIKQLFLLKKKHRQLKVLLSIGGW-TYSANFAGPA    109
tr|A0A179UX46|A0A179UX46_BLAGS      DTWADVEKRYP-GDSWEEPGENVYGCIKQLYLLKKHYRHLKTLLSIGGW-TYSANFPVPA    209
tr|M9NG70|M9NG70_DROME              DKYQDI----------------EQGGYAKFTGLKTYNKQLKTMIAIGGWNEASSRFSPLV    122
tr|Q22468|Q22468_CAEEL              ---GDA----------------AKARLEHVKEVASRHPDLKLLYAVGGWEN-SQYFSVLT    113
                                                                               : : ..**.  :   .

tr|A0A0S4J833|A0A0S4J833_BODSA      ATPSSRRVFLGALEALLTQYDFDGVDYNWEYPRDAE-------EWRRWALLLKESKDTLK    174
tr|A0A250X009|A0A250X009_9CHLO      VDKARRRRFLEALSNLMVSHSFDGVDYNWEYPTKYV-------EWEGLADLMKETKALLN    133
tr|C5K6G1|C5K6G1_PERM5              AHNGDVRRLIKQVDDLLNKYQLDGVDFNWEYPQSET-------EWYNFKQMLRWLKVKLR    162
tr|C1N616|C1N616_MICPC              VEKTKRRRLARTLASFCATHGLHGVDYNWEYPASPA-------EWNGMFALLRTTRKVFD    186
tr|A0A7G2CNI0|A0A7G2CNI0_9TRYP      RSNH-TENFFSQLKSLYVDRKLDGFDFNWEYPALEE-------DYRKMNEFLIKARAALN    166
tr|S9TUR0|S9TUR0_9TRYP              RDAAKRRAFVAQLNELYVERRLDGVDVNWEYPQTME-------EWRLLGVLLREMRAALG    239
tr|A0A0M9FXG9|A0A0M9FXG9_LEPPY      GDVLGRRRFVSEVGRVLHERDLDGVDFNWEYPSSMP-------EWLNFGQFLTELRSALN    205
tr|A4H8K3|A4H8K3_LEIBR              GDPVRRLSFIEEVNAVLLTRELDGIDFNWEYPQTMT-------EWLNFGRFLLEIRSALG    198
tr|A4HWX6|A4HWX6_LEIIN              GDTVQRQAFIEEVNAVLLARKLDGIDFNWEYPQTMT-------EWLNFGRFLMELRSSLG    198
tr|Q4QEU0|Q4QEU0_LEIMA              GDTVQRKAFIEEVNAVLLARKLDGIDFNWDYPQTMT-------EWLNFGRFLMELRSSLG    198
sp|O81862-2|CHIC_ARATH              SNPTSRKSFIDSSIRVARSYGFHGLDLDWEYPSSAT-------EMTNFGTLLREWRSAVV    121
tr|S8AUV1|S8AUV1_PENO1              STPAGRARFAESATRLVLDLGLDGIDIDWEYPQDET-------QALNLVELLRACRHNLD    162
tr|A0A179UX46|A0A179UX46_BLAGS      STETGRKTFARTAVQLLADLGFDGIDIDWEYPQDAA-------QAENFVRLLKETREALD    262
tr|M9NG70|M9NG70_DROME              ASNERRQQFIKNILKFLRQNHFDGIDLDWEYPAHRE--GGKSRDRDNYAQFVQELRAEFE    180
tr|Q22468|Q22468_CAEEL              ADHSRRSILISNFVKVIKEYGFDGVDIDWEYPVTGGAVEGTPADRRNYVNLMRELRNELR    173
                                            :        .        :.*.* :*:**              :        ::   :  .

tr|A0A0S4J833|A0A0S4J833_BODSA      GGD-------------------RSQNIVTFTMYLDPKHADV-IQRFNMLEHADYVHCMAY    214
tr|A0A250X009|A0A250X009_9CHLO      G---------------------QVIVTSAFYPDPNQYII-IKALKLHEICHYLLSMTY    169
tr|C5K6G1|C5K6G1_PERM5              KRE-------------------K-PAIITLAYQPGGLQEDM-IAKLRFAKQCDYFLAMSY    201
tr|C1N616|C1N616_MICPC              GSP-------------------Q-RLTITMAYYPDGRQERE-LKRGGAEEHVELLHAMSY    225
tr|A0A7G2CNI0|A0A7G2CNI0_9TRYP      ST--------------------GRPPIVSMPAHPHPTTSSF-LRLSGINDAVDYIHWMAY    205
tr|S9TUR0|S9TUR0_9TRYP              FETVSGGPDGARP---RRRRMRARAPTLTIALHPHIQGGLL-LRRNDVLDAVDYVHWMAY    295
tr|A0A0M9FXG9|A0A0M9FXG9_LEPPY      RTTA-----------GDHHGRRVGPALITMALHPHPRIPHV-LRVSHVLPSLDYLHWMAY    253
tr|A4H8K3|A4H8K3_LEIBR              YAPAVGREHAAGLIGERHHRTRVRHAVLSMALHPHSSIAAV-LQSARVLRSLDYVHWMAY    257
tr|A4HWX6|A4HWX6_LEIIN              YAAAAGGEHAAGPIGERRHRTRVRGAALSMALHPHPSMAAV-LQSARVLHSLDYVHLMAY    257
tr|Q4QEU0|Q4QEU0_LEIMA              YAAAAGGEHAAEPIGERRYRTRVRGAVLSMALHPHPSMAAV-LQSARMLHSLDYVHLMAY    257
sp|O81862-2|CHIC_ARATH              AEASSSGK---------------PRLLLAAAVFYSNNYYSVLYPVSAVASSLDWVNLMAY    166
tr|S8AUV1|S8AUV1_PENO1              TAAG---P---------------HRKFYLSIACPAGPNNFNK-LKLHEMTPLLDFYNLMAY    204
tr|A0A179UX46|A0A179UX46_BLAGS      AYSAQHAQ--------------GRRLLLTVAVPCGETNYKK-LLMSDMDKYLDFWNLMCY    307
tr|M9NG70|M9NG70_DROME              REAEKTGR---------------TRLLLTMAVPAGIEYIDKGYDVPKLNKYLDWFNVLTY    225
tr|Q22468|Q22468_CAEEL              DLESETGK---------------S-YLISFAGAAGHWVLKPGYDLQQLMKYCDFVNVMSY    217
                                                              ::                       .        : *

tr|A0A0S4J833|A0A0S4J833_BODSA      DQ-HGE-----------------HSTYEFAVSGVRMA----------------------    233
tr|A0A250X009|A0A250X009_9CHLO      DMVPGK-----------------HSTYEFAVQTIEAW----------------------    189
tr|C5K6G1|C5K6G1_PERM5              EHPEGK-----------------GE--DLARVVVEAW----------------------    219
tr|C1N616|C1N616_MICPC              DHPRGS-----------------HSSTALARAAIRNA----------------------    245
tr|A0A7G2CNI0|A0A7G2CNI0_9TRYP      GTVERL-SV-----------------------LMQPLVL--------------------    220
tr|S9TUR0|S9TUR0_9TRYP              DFYLKP-NEQPD--KEP--PL-GHTDYRYASGMLSDEVLEGFLDPAASRGS---------    340
tr|A0A0M9FXG9|A0A0M9FXG9_LEPPY      DHIIAN-DS--------------HSSVAYAASVLQDDVIGDLDDAVYNARLKKIRRQAHG    298
tr|A4H8K3|A4H8K3_LEIBR              DHILGT-EP--------------HSSVEYAASVLSEEMIGLFNEATYNKRSGQTHQE---    299
tr|A4HWX6|A4HWX6_LEIIN              DHVVGT-GP--------------HSSVEYAASVLSEETIGFFNEAAYNRRLGQMHRR---    299
tr|Q4QEU0|Q4QEU0_LEIMA              DHVAGT-GP--------------HSSVEYAASVLSEETIGLFNEAVYNRRLGRMHQR---    299
sp|O81862-2|CHIC_ARATH              DFYGPGWSRV----TGPPAALFDPSNAGPS----GDA----------------------    195
tr|S8AUV1|S8AUV1_PENO1              DYAG-SWDRV----AGHQANLYPSRSNPASTPFSTIA----------------------    236
tr|A0A179UX46|A0A179UX46_BLAGS      DFAG-SWDRK----AGHMANIFPSRDVPESTPFNADE----------------------    339
tr|M9NG70|M9NG70_DROME              DFHS-SHEPSVNH-HAPLYSLEEDSEYNYDAELNIDY----------------------    260
tr|Q22468|Q22468_CAEEL              DYFG-AWASKWGAYTGPPAPLQFAMPKKFSGRMNVHA----------------------    253
```

```
tr|A0A0S4J833|A0A0S4J833_BODSA      -----------IEKKMTLSKFTLGVPFYARHVGNGE--PKTYGEIIKEIPK---------    271
tr|A0A250X009|A0A250X009_9CHLO      -----------KQQGLPLDKLALGVPFYGRHMQTGA--PDTYYDLFPKLEKR--------    228
tr|C5K6G1|C5K6G1_PERM5              -----------EQRGLDIRKLALGIPFYGRDLQTGE--ARTYSEISTIP-----------    255
tr|C1N616|C1N616_MICPC              -----------EEAGLDARKITIGLPFYGRHVETGE--WKTYAELDAAH-----------    281
tr|A0A7G2CNI0|A0A7G2CNI0_9TRYP      -----------EGFYDNIKKLTLGLDFYATNGPQNA---TTYSEVVVR--AL--------    256
tr|S9TUR0|S9TUR0_9TRYP              ------RRSKLDIYRDPRRKLTLGIPLFAKHIAENFRHPMPYKDVMTE--QV--------    384
tr|A0A0M9FXG9|A0A0M9FXG9_LEPPY      EAEEDAPQSRKPHEADHRRKLCLGIPFYGRHRADGRVPPETYEHLWQFLRQW--------    350
tr|A4H8K3|A4H8K3_LEIBR              ----------LRTEQDHRRKLTLGIPFYGRHRENRRRQPETYDRLWRSIQQW--------    341
tr|A4HWX6|A4HWX6_LEIIN              ----------PRTEQDHRRKLTLGIPFYGRHREDRRLQPEAYDRLWLFIQEW--------    341
tr|Q4QEU0|Q4QEU0_LEIMA              ----------PRTEQDHRRKLTLGIPFYGRHREDRRLQPEAYDRLWFFIQEW--------    341
sp|O81862-2|CHIC_ARATH              -----GTRSWI-QAGLPAKKAVLGFPYYGYAWRLTNANSHSYYAP--------TTGAAIS    241
tr|S8AUV1|S8AUV1_PENO1              -----ALDHYIGVGGVPPNKMILGMPLYGRAFEGTDGPGTPYSGV--------GPGSW--    281
tr|A0A179UX46|A0A179UX46_BLAGS      -----AITAYV-AGGVHPKKIVFGLPLYGRAFEQTDGPGHPFQGV--------GEGSW--    383
tr|M9NG70|M9NG70_DROME              -----SIKYYL-KAGADRDKLVLGIPTYGRSYTLINEESTELGAP---AEGPGEQGDATR    311
tr|Q22468|Q22468_CAEEL              -----TMKDYS-CQIKATDKINMGVPFYGRFWKNVGDAVDSTDDMWRTATATNSEG-TKF    306
                                                           *  :*.  :.

tr|A0A0S4J833|A0A0S4J833_BODSA      --------------------------------SKRWTQDRVGPYYLNSPSMIQKKTKLAI    299
tr|A0A250X009|A0A250X009_9CHLO      ------------YK----------------NLSKRHAVDELGAVFFNGRSTLKQKAELAI    260
tr|C5K6G1|C5K6G1_PERM5              ------------------------------D--------AQHKFVYDTPEEVQARTRYAL    277
tr|C1N616|C1N616_MICPC              ------------------------------GVSAEPSKDEAGGYAFNGADTIRLKTSEAK    311
tr|A0A7G2CNI0|A0A7G2CNI0_9TRYP      ------------MQSEKPATDE--------VVQRLKESSNEGGFDFTGYYGVQKKMDRAR    296
tr|S9TUR0|S9TUR0_9TRYP              ------------ARVAPPQSTASSAELERQMAAFIRPVNELDRYTFSGYNEIQKKVELAR    432
tr|A0A0M9FXG9|A0A0M9FXG9_LEPPY      ------------AGKRHPDWVE--------GGPELRSLSEYAGYDYNGYDDVRRKMRLAR    390
tr|A4H8K3|A4H8K3_LEIBR              ------------ARKKHPAWVE--------GGAELRALNNYGGYSFTGYNDVKRKMRLAR    381
tr|A4HWX6|A4HWX6_LEIIN              ------------ASKSHPTWVE--------GGAELRALSEYGGYSFTGYDDVKRKMQLTR    381
tr|Q4QEU0|Q4QEU0_LEIMA              ------------ASKNHPTWVE--------GGAELRALSEYGGYSFTCYDDVKRKMRLAR    381
sp|O81862-2|CHIC_ARATH              PDGSIGYGQIRKFIVDNG--ATTVYNS-TVVGDYCY--AGTNWIGYDDNQSIVTKVRYAK    296
tr|S8AUV1|S8AUV1_PENO1              ENGVWDYK----ALPRPG--ATEHLDT-EADASWSYDPASRTMVSYDTVVMSERKAAFVK    334
tr|A0A179UX46|A0A179UX46_BLAGS      ESGVWDYK----VLPQPG--SEEVNDD-DLKASWSYDRNARKMISYDTPAIVAQKADYIR    436
tr|M9NG70|M9NG70_DROME              EKGYLAYYEICQTLKDDPEWTVVQPNA-NVMGP--YAYRRNQWVGYDDEAIVRKKAEYVV    368
tr|Q22468|Q22468_CAEEL              EGGDVQWRDLHEKFDT----TKTKFHS-GSKTPFIWLSEQKTFVGYENAESLKHKVDYIV    361
                                                                                           :

tr|A0A0S4J833|A0A0S4J833_BODSA      DN----QLGGVMIWELGQDLQPE-S---------------HRNSLLRGILKATLTRG---    336
tr|A0A250X009|A0A250X009_9CHLO      RE----GLGGIMIWELGQDISPP-S---------------HESALMSGLGEAIVPMG---    297
tr|C5K6G1|C5K6G1_PERM5              EE----GLGGVMIWELGQDLPSS-----------------NERSLLAAIGKVVSSPS---    313
tr|C1N616|C1N616_MICPC              AA----GTGGVMIWEAGQDLHPS-----------------HARSLLAVVAAEAWGDE---    347
tr|A0A7G2CNI0|A0A7G2CNI0_9TRYP      QA----NFTGVMIWELGQDILSYPPEVLKKATDDATIEKLYNVSLMRSIHEKLT-EWKKA    351
tr|S9TUR0|S9TUR0_9TRYP              TGGPPGGIGGIMIWELGQDLLPPSE---------------HPLAAMNAISEVVQERWGGP    477
tr|A0A0M9FXG9|A0A0M9FXG9_LEPPY      SS----GIAGIMIWELGQDV-PPGT---------------SPMSLMTAVQEQLA-EWDKD    429
tr|A4H8K3|A4H8K3_LEIBR              AA----NLSGIMIWELGQDV-PPGT---------------SPMSLMAAVHEQLA-DWGLL    420
tr|A4HWX6|A4HWX6_LEIIN              AA----NLSGIMIWELGQDV-PPGT---------------SPMSLMTAVHEQLA-DWGLL    420
tr|Q4QEU0|Q4QEU0_LEIMA              AA----NLSGIMIWELGQDV-PPGT---------------SPMSLMTAVHEQLA-DWGLL    420
sp|O81862-2|CHIC_ARATH              QR----GLLGYFSWHVGADDNS-GLSR------------AASQAWDATTAT------TR-    332
tr|S8AUV1|S8AUV1_PENO1              YR----QLGGGMWWESSGDKGG-KDAN------------PAQGSLIGTF--------VD-    368
tr|A0A179UX46|A0A179UX46_BLAGS      KR----GMGGGMWWELSGDAPV-----------------GSERSLIATT--------VN-    466
tr|M9NG70|M9NG70_DROME              AQ----GLGGIMFWAIDNDDFR-GTCN------------GKPYPLIEAAKEAMVEALGL-    410
tr|Q22468|Q22468_CAEEL              EN----NIGGVMIWAIDFDDDQ-GTLL------------NSAA------AESICTTSTK-    397
                                             * : *  . *

tr|A0A0S4J833|A0A0S4J833_BODSA      LALPK----LQE-QLRAAK--EETPS---LKEPRDE---------L--------------    363
tr|A0A250X009|A0A250X009_9CHLO      SLQKT----VLGAGISSEVEIEEELN---VVLSTDDVATVNSKTEL--------------    336
tr|C5K6G1|C5K6G1_PERM5              --IEE----DGG---EKEEDDDEEL-----------------------------------    329
tr|C1N616|C1N616_MICPC              --GKN----AVG---EKKRAR-EEL-----------------------------------    362
tr|A0A7G2CNI0|A0A7G2CNI0_9TRYP      DPPKKD-I--------------------------------NEEGDL--------------    364
tr|S9TUR0|S9TUR0_9TRYP              ARPWTGEVRDGAGGVTREADAASTPTEKATAPPRE--ADYEELSDL--------------    521
tr|A0A0M9FXG9|A0A0M9FXG9_LEPPY      TASNA----DESDACLRTPARLEH---TRENATRLVGSDD-ADEDL--------------    467
tr|A4H8K3|A4H8K3_LEIBR              TDSGH----DSGGDVNGDNTG--H---LQQRQRRYLSSDVAEDGDL--------------    457
tr|A4HWX6|A4HWX6_LEIIN              TDGGR----GSGGNVNSDNAY--D---RPQPPPQHSSPDVAEDGDL--------------    457
tr|Q4QEU0|Q4QEU0_LEIMA              TDSGR----GIGGNVNSDNAH--D---RPQPSPQHSSPDVAEDGDL--------------    457
sp|O81862-2|CHIC_ARATH              ---------TIQ-KV---------------------------------------------    337
tr|S8AUV1|S8AUV1_PENO1              ---------GIG-GVTA-L---D----GIENALDYPESKYDNLRAGFPGQ----------    400
tr|A0A179UX46|A0A179UX46_BLAGS      ---------GLG-GVGN-L---D----HSENLLDYPASRYENLRKGFQ------------    496
tr|M9NG70|M9NG70_DROME              ---------GIN-EVAK-P---S----GPQKPSRSRSRDNASNRNRLNGKTEAPLSSRRP    452
tr|Q22468|Q22468_CAEEL              ---------SFN-YKCS-----------PVDDKRWWTYDDNEELAGMCGKSSPLIDGYYP    436


tr|A0A0S4J833|A0A0S4J833_BODSA      ------------------------------------------------------------    363
tr|A0A250X009|A0A250X009_9CHLO      ------------------------------------------------------------    336
tr|C5K6G1|C5K6G1_PERM5              ------------------------------------------------------------    329
tr|C1N616|C1N616_MICPC              ------------------------------------------------------------    362
tr|A0A7G2CNI0|A0A7G2CNI0_9TRYP      ------------------------------------------------------------    364
tr|S9TUR0|S9TUR0_9TRYP              ------------------------------------------------------------    521
tr|A0A0M9FXG9|A0A0M9FXG9_LEPPY      ------------------------------------------------------------    467
tr|A4H8K3|A4H8K3_LEIBR              ------------------------------------------------------------    457
tr|A4HWX6|A4HWX6_LEIIN              ------------------------------------------------------------    457
tr|Q4QEU0|Q4QEU0_LEIMA              ------------------------------------------------------------    457
sp|O81862-2|CHIC_ARATH              ------------------------------------------------------------    337
tr|S8AUV1|S8AUV1_PENO1              ------------------------------------------------------------    400
tr|A0A179UX46|A0A179UX46_BLAGS      ------------------------------------------------------------    496
tr|M9NG70|M9NG70_DROME              SA-------------------------------TRRPAVSSTQAPPPS--TTFKL-TEAE    478
tr|Q22468|Q22468_CAEEL              VCDPDDPGHACCGKYGYCGSGAEFCSCPECIDYGADPNLILKEPVKPSQKITWYTSDAGE    496
```

```
tr|A0A0S4J833|A0A0S4J833_BODSA     ------------------------------------------------------------     363
tr|A0A250X009|A0A250X009_9CHLO     ------------------------------------------------------------     336
tr|C5K6G1|C5K6G1_PERM5             ------------------------------------------------------------     329
tr|C1N616|C1N616_MICPC             ------------------------------------------------------------     362
tr|A0A7G2CNI0|A0A7G2CNI0_9TRYP     ------------------------------------------------------------     364
tr|S9TUR0|S9TUR0_9TRYP             ------------------------------------------------------------     521
tr|A0A0M9FXG9|A0A0M9FXG9_LEPPY     ------------------------------------------------------------     467
tr|A4H8K3|A4H8K3_LEIBR             ------------------------------------------------------------     457
tr|A4HWX6|A4HWX6_LEIIN             ------------------------------------------------------------     457
tr|Q4QEU0|Q4QEU0_LEIMA             ------------------------------------------------------------     457
sp|O81862-2|CHIC_ARATH             ------------------------------------------------------------     337
tr|S8AUV1|S8AUV1_PENO1             ------------------------------------------------------------     400
tr|A0A179UX46|A0A179UX46_BLAGS     ------------------------------------------------------------     496
tr|M9NG70|M9NG70_DROME             GSSLYIGGRASTTPP------PPTTPDPGSDFKCEEEGFFQHPRDC--------------     518
tr|Q22468|Q22468_CAEEL             GK----RGRCGRDVPPLEGEAPTCNPDDANAHCCSNGGYCGNSKEHCECNGCIDFAKQRD     552

tr|A0A0S4J833|A0A0S4J833_BODSA     ------------------------------------------------------------     363
tr|A0A250X009|A0A250X009_9CHLO     ------------------------------------------------------------     336
tr|C5K6G1|C5K6G1_PERM5             ------------------------------------------------------------     329
tr|C1N616|C1N616_MICPC             ------------------------------------------------------------     362
tr|A0A7G2CNI0|A0A7G2CNI0_9TRYP     ------------------------------------------------------------     364
tr|S9TUR0|S9TUR0_9TRYP             ------------------------------------------------------------     521
tr|A0A0M9FXG9|A0A0M9FXG9_LEPPY     ------------------------------------------------------------     467
tr|A4H8K3|A4H8K3_LEIBR             ------------------------------------------------------------     457
tr|A4HWX6|A4HWX6_LEIIN             ------------------------------------------------------------     457
tr|Q4QEU0|Q4QEU0_LEIMA             ------------------------------------------------------------     457
sp|O81862-2|CHIC_ARATH             ------------------------------------------------------------     337
tr|S8AUV1|S8AUV1_PENO1             ------------------------------------------------------------     400
tr|A0A179UX46|A0A179UX46_BLAGS     ------------------------------------------------------------     496
tr|M9NG70|M9NG70_DROME             ----KKYYWCLDSGPSGLGIVAH------------------MFTCPSGLYFNPAADSCDF     556
tr|Q22468|Q22468_CAEEL             FKYKPLEWWTFSENPANVGRCGYNAPRLSTGKIPKCDPDSESYCCSNSGYCGKGEQYCS-     611

tr|A0A0S4J833|A0A0S4J833_BODSA     ------------------------------------------------------------     363
tr|A0A250X009|A0A250X009_9CHLO     ------------------------------------------------------------     336
tr|C5K6G1|C5K6G1_PERM5             ------------------------------------------------------------     329
tr|C1N616|C1N616_MICPC             ------------------------------------------------------------     362
tr|A0A7G2CNI0|A0A7G2CNI0_9TRYP     ------------------------------------------------------------     364
tr|S9TUR0|S9TUR0_9TRYP             ------------------------------------------------------------     521
tr|A0A0M9FXG9|A0A0M9FXG9_LEPPY     ------------------------------------------------------------     467
tr|A4H8K3|A4H8K3_LEIBR             ------------------------------------------------------------     457
tr|A4HWX6|A4HWX6_LEIIN             ------------------------------------------------------------     457
tr|Q4QEU0|Q4QEU0_LEIMA             ------------------------------------------------------------     457
sp|O81862-2|CHIC_ARATH             ------------------------------------------------------------     337
tr|S8AUV1|S8AUV1_PENO1             ------------------------------------------------------------     400
tr|A0A179UX46|A0A179UX46_BLAGS     ------------------------------------------------------------     496
tr|M9NG70|M9NG70_DROME             ARNVPCKTKKSTTAAPVTSTTPATTTVRSNRVTAAPTSRPVYPRTTTTTSTTTTTTTTPS     616
tr|Q22468|Q22468_CAEEL             --CLGCADFKANP-----------------------------------------------     622

tr|A0A0S4J833|A0A0S4J833_BODSA     ------------------------------------------------------------     363
tr|A0A250X009|A0A250X009_9CHLO     ------------------------------------------------------------     336
tr|C5K6G1|C5K6G1_PERM5             ------------------------------------------------------------     329
tr|C1N616|C1N616_MICPC             ------------------------------------------------------------     362
tr|A0A7G2CNI0|A0A7G2CNI0_9TRYP     ------------------------------------------------------------     364
tr|S9TUR0|S9TUR0_9TRYP             ------------------------------------------------------------     521
tr|A0A0M9FXG9|A0A0M9FXG9_LEPPY     ------------------------------------------------------------     467
tr|A4H8K3|A4H8K3_LEIBR             ------------------------------------------------------------     457
tr|A4HWX6|A4HWX6_LEIIN             ------------------------------------------------------------     457
tr|Q4QEU0|Q4QEU0_LEIMA             ------------------------------------------------------------     457
sp|O81862-2|CHIC_ARATH             ------------------------------------------------------------     337
tr|S8AUV1|S8AUV1_PENO1             ------------------------------------------------------------     400
tr|A0A179UX46|A0A179UX46_BLAGS     ------------------------------------------------------------     496
tr|M9NG70|M9NG70_DROME             TVDEDLEYEEDTDELSPSKSTDAEEDPQVIKELIDLIRKVGGVEQLEKHLLRNKDGSITL     676
tr|Q22468|Q22468_CAEEL             ----AFEY----------------------------------------------------     626

tr|A0A0S4J833|A0A0S4J833_BODSA     ------------------------------------------------------------     363
tr|A0A250X009|A0A250X009_9CHLO     ------------------------------------------------------------     336
tr|C5K6G1|C5K6G1_PERM5             ------------------------------------------------------------     329
tr|C1N616|C1N616_MICPC             ------------------------------------------------------------     362
tr|A0A7G2CNI0|A0A7G2CNI0_9TRYP     ------------------------------------------------------------     364
tr|S9TUR0|S9TUR0_9TRYP             ------------------------------------------------------------     521
tr|A0A0M9FXG9|A0A0M9FXG9_LEPPY     ------------------------------------------------------------     467
tr|A4H8K3|A4H8K3_LEIBR             ------------------------------------------------------------     457
tr|A4HWX6|A4HWX6_LEIIN             ------------------------------------------------------------     457
tr|Q4QEU0|Q4QEU0_LEIMA             ------------------------------------------------------------     457
sp|O81862-2|CHIC_ARATH             ------------------------------------------------------------     337
tr|S8AUV1|S8AUV1_PENO1             ------------------------------------------------------------     400
tr|A0A179UX46|A0A179UX46_BLAGS     ------------------------------------------------------------     496
tr|M9NG70|M9NG70_DROME             KENSATGAATTPSTISKSLYDRVLSRPGTLNSFSRNRFKISEASETSTEPTTSSSSSRGS     736
tr|Q22468|Q22468_CAEEL             ------------------------------------------------------------     626

tr|A0A0S4J833|A0A0S4J833_BODSA     ------------------------------------------------------------     363
tr|A0A250X009|A0A250X009_9CHLO     ------------------------------------------------------------     336
```

```
tr|C5K6G1|C5K6G1_PERM5              ------------------------------------------------------------    329
tr|C1N616|C1N616_MICPC              ------------------------------------------------------------    362
tr|A0A7G2CNI0|A0A7G2CNI0_9TRYP      ------------------------------------------------------------    364
tr|S9TUR0|S9TUR0_9TRYP              ------------------------------------------------------------    521
tr|A0A0M9FXG9|A0A0M9FXG9_LEPPY      ------------------------------------------------------------    467
tr|A4H8K3|A4H8K3_LEIBR              ------------------------------------------------------------    457
tr|A4HWX6|A4HWX6_LEIIN              ------------------------------------------------------------    457
tr|Q4QEU0|Q4QEU0_LEIMA              ------------------------------------------------------------    457
sp|O81862-2|CHIC_ARATH              ------------------------------------------------------------    337
tr|S8AUV1|S8AUV1_PENO1              ------------------------------------------------------------    400
tr|A0A179UX46|A0A179UX46_BLAGS      ------------------------------------------------------------    496
tr|M9NG70|M9NG70_DROME              STLTSNTNSKYSSVLRGNSRQGPQNEGIEKLAEFDGFLKERKQYVTINRHRSASQGDEEE    796
tr|Q22468|Q22468_CAEEL              ------------------------------------------------------------    626

tr|A0A0S4J833|A0A0S4J833_BODSA      ------------------------------------------------------------    363
tr|A0A250X009|A0A250X009_9CHLO      ------------------------------------------------------------    336
tr|C5K6G1|C5K6G1_PERM5              ------------------------------------------------------------    329
tr|C1N616|C1N616_MICPC              ------------------------------------------------------------    362
tr|A0A7G2CNI0|A0A7G2CNI0_9TRYP      ------------------------------------------------------------    364
tr|S9TUR0|S9TUR0_9TRYP              ------------------------------------------------------------    521
tr|A0A0M9FXG9|A0A0M9FXG9_LEPPY      ------------------------------------------------------------    467
tr|A4H8K3|A4H8K3_LEIBR              ------------------------------------------------------------    457
tr|A4HWX6|A4HWX6_LEIIN              ------------------------------------------------------------    457
tr|Q4QEU0|Q4QEU0_LEIMA              ------------------------------------------------------------    457
sp|O81862-2|CHIC_ARATH              ------------------------------------------------------------    337
tr|S8AUV1|S8AUV1_PENO1              ------------------------------------------------------------    400
tr|A0A179UX46|A0A179UX46_BLAGS      ------------------------------------------------------------    496
tr|M9NG70|M9NG70_DROME              HADQQEEEENLAEVETTTRRPLSSITPSYTSLRRSRPTTVAPPAEESHEEAEQQTQTQVK    856
tr|Q22468|Q22468_CAEEL              ------------------------------------------------------------    626

tr|A0A0S4J833|A0A0S4J833_BODSA      ------------------------------------------------------------    363
tr|A0A250X009|A0A250X009_9CHLO      ------------------------------------------------------------    336
tr|C5K6G1|C5K6G1_PERM5              ------------------------------------------------------------    329
tr|C1N616|C1N616_MICPC              ------------------------------------------------------------    362
tr|A0A7G2CNI0|A0A7G2CNI0_9TRYP      ------------------------------------------------------------    364
tr|S9TUR0|S9TUR0_9TRYP              ------------------------------------------------------------    521
tr|A0A0M9FXG9|A0A0M9FXG9_LEPPY      ------------------------------------------------------------    467
tr|A4H8K3|A4H8K3_LEIBR              ------------------------------------------------------------    457
tr|A4HWX6|A4HWX6_LEIIN              ------------------------------------------------------------    457
tr|Q4QEU0|Q4QEU0_LEIMA              ------------------------------------------------------------    457
sp|O81862-2|CHIC_ARATH              ------------------------------------------------------------    337
tr|S8AUV1|S8AUV1_PENO1              ------------------------------------------------------------    400
tr|A0A179UX46|A0A179UX46_BLAGS      ------------------------------------------------------------    496
tr|M9NG70|M9NG70_DROME              SYATLSRTRGRTTSSPEVTEAAPSSTTNRYKYFERTRPTKSATAEDSEDPTEDEEEEYED    916
tr|Q22468|Q22468_CAEEL              ------------------------------------------------------------    626

tr|A0A0S4J833|A0A0S4J833_BODSA      ------------------------------------------------------------    363
tr|A0A250X009|A0A250X009_9CHLO      ------------------------------------------------------------    336
tr|C5K6G1|C5K6G1_PERM5              ------------------------------------------------------------    329
tr|C1N616|C1N616_MICPC              ------------------------------------------------------------    362
tr|A0A7G2CNI0|A0A7G2CNI0_9TRYP      ------------------------------------------------------------    364
tr|S9TUR0|S9TUR0_9TRYP              ------------------------------------------------------------    521
tr|A0A0M9FXG9|A0A0M9FXG9_LEPPY      ------------------------------------------------------------    467
tr|A4H8K3|A4H8K3_LEIBR              ------------------------------------------------------------    457
tr|A4HWX6|A4HWX6_LEIIN              ------------------------------------------------------------    457
tr|Q4QEU0|Q4QEU0_LEIMA              ------------------------------------------------------------    457
sp|O81862-2|CHIC_ARATH              ------------------------------------------------------------    337
tr|S8AUV1|S8AUV1_PENO1              ------------------------------------------------------------    400
tr|A0A179UX46|A0A179UX46_BLAGS      ------------------------------------------------------------    496
tr|M9NG70|M9NG70_DROME              EQKDIVTLRQPTTGSVSRRPVLSVRRRIINSPTSAAVETTTQQPAAEPPTTSKYSRLRSR    976
tr|Q22468|Q22468_CAEEL              ------------------------------------------------------------    626

tr|A0A0S4J833|A0A0S4J833_BODSA      ------------------------------------------------------------    363
tr|A0A250X009|A0A250X009_9CHLO      ------------------------------------------------------------    336
tr|C5K6G1|C5K6G1_PERM5              ------------------------------------------------------------    329
tr|C1N616|C1N616_MICPC              ------------------------------------------------------------    362
tr|A0A7G2CNI0|A0A7G2CNI0_9TRYP      ------------------------------------------------------------    364
tr|S9TUR0|S9TUR0_9TRYP              ------------------------------------------------------------    521
tr|A0A0M9FXG9|A0A0M9FXG9_LEPPY      ------------------------------------------------------------    467
tr|A4H8K3|A4H8K3_LEIBR              ------------------------------------------------------------    457
tr|A4HWX6|A4HWX6_LEIIN              ------------------------------------------------------------    457
tr|Q4QEU0|Q4QEU0_LEIMA              ------------------------------------------------------------    457
sp|O81862-2|CHIC_ARATH              ------------------------------------------------------------    337
tr|S8AUV1|S8AUV1_PENO1              ------------------------------------------------------------    400
tr|A0A179UX46|A0A179UX46_BLAGS      ------------------------------------------------------------    496
tr|M9NG70|M9NG70_DROME              PSATATAAAAAATTTTAAATTAFPAATSAPGGRTTSNIYLSKLKAKSGAAAAAAASGEAA    1036
tr|Q22468|Q22468_CAEEL              ------------------------------------------------------------    626

tr|A0A0S4J833|A0A0S4J833_BODSA      ------------------------------------------------------------    363
tr|A0A250X009|A0A250X009_9CHLO      ------------------------------------------------------------    336
tr|C5K6G1|C5K6G1_PERM5              ------------------------------------------------------------    329
tr|C1N616|C1N616_MICPC              ------------------------------------------------------------    362
```

```
tr|A0A7G2CNI0|A0A7G2CNI0_9TRYP    ------------------------------------------------------------    364
tr|S9TUR0|S9TUR0_9TRYP            ------------------------------------------------------------    521
tr|A0A0M9FXG9|A0A0M9FXG9_LEPPY    ------------------------------------------------------------    467
tr|A4H8K3|A4H8K3_LEIBR            ------------------------------------------------------------    457
tr|A4HWX6|A4HWX6_LEIIN            ------------------------------------------------------------    457
tr|Q4QEU0|Q4QEU0_LEIMA            ------------------------------------------------------------    457
sp|O81862-2|CHIC_ARATH            ------------------------------------------------------------    337
tr|S8AUV1|S8AUV1_PENO1            ------------------------------------------------------------    400
tr|A0A179UX46|A0A179UX46_BLAGS    ------------------------------------------------------------    496
tr|M9NG70|M9NG70_DROME            TLTPATSNISSSSNDITQKQHKFQPASFALRRQFQTRRLTTFAPAANGDESATEVPRTQN    1096
tr|Q22468|Q22468_CAEEL            ------------------------------------------------------------    626

tr|A0A0S4J833|A0A0S4J833_BODSA    ------------------------------------------------------------    363
tr|A0A250X009|A0A250X009_9CHLO    ------------------------------------------------------------    336
tr|C5K6G1|C5K6G1_PERM5            ------------------------------------------------------------    329
tr|C1N616|C1N616_MICPC            ------------------------------------------------------------    362
tr|A0A7G2CNI0|A0A7G2CNI0_9TRYP    ------------------------------------------------------------    364
tr|S9TUR0|S9TUR0_9TRYP            ------------------------------------------------------------    521
tr|A0A0M9FXG9|A0A0M9FXG9_LEPPY    ------------------------------------------------------------    467
tr|A4H8K3|A4H8K3_LEIBR            ------------------------------------------------------------    457
tr|A4HWX6|A4HWX6_LEIIN            ------------------------------------------------------------    457
tr|Q4QEU0|Q4QEU0_LEIMA            ------------------------------------------------------------    457
sp|O81862-2|CHIC_ARATH            ------------------------------------------------------------    337
tr|S8AUV1|S8AUV1_PENO1            ------------------------------------------------------------    400
tr|A0A179UX46|A0A179UX46_BLAGS    ------------------------------------------------------------    496
tr|M9NG70|M9NG70_DROME            PLFKRRLTLISTTPPSARTTNPPVSGLETTTTLYLNDDDEDQVAKSSIHTSRFNQIPEQV    1156
tr|Q22468|Q22468_CAEEL            ------------------------------------------------------------    626

tr|A0A0S4J833|A0A0S4J833_BODSA    ------------------------------------------------------------    363
tr|A0A250X009|A0A250X009_9CHLO    ------------------------------------------------------------    336
tr|C5K6G1|C5K6G1_PERM5            ------------------------------------------------------------    329
tr|C1N616|C1N616_MICPC            ------------------------------------------------------------    362
tr|A0A7G2CNI0|A0A7G2CNI0_9TRYP    ------------------------------------------------------------    364
tr|S9TUR0|S9TUR0_9TRYP            ------------------------------------------------------------    521
tr|A0A0M9FXG9|A0A0M9FXG9_LEPPY    ------------------------------------------------------------    467
tr|A4H8K3|A4H8K3_LEIBR            ------------------------------------------------------------    457
tr|A4HWX6|A4HWX6_LEIIN            ------------------------------------------------------------    457
tr|Q4QEU0|Q4QEU0_LEIMA            ------------------------------------------------------------    457
sp|O81862-2|CHIC_ARATH            ------------------------------------------------------------    337
tr|S8AUV1|S8AUV1_PENO1            ------------------------------------------------------------    400
tr|A0A179UX46|A0A179UX46_BLAGS    ------------------------------------------------------------    496
tr|M9NG70|M9NG70_DROME            RPREEYDLALPAQPLKSTSTTVSTTANAPLPVIGQGIRRQLIPRPRRPQSTTSTPPTTTT    1216
tr|Q22468|Q22468_CAEEL            ------------------------------------------------------------    626

tr|A0A0S4J833|A0A0S4J833_BODSA    ------------------------------------------------------------    363
tr|A0A250X009|A0A250X009_9CHLO    ------------------------------------------------------------    336
tr|C5K6G1|C5K6G1_PERM5            ------------------------------------------------------------    329
tr|C1N616|C1N616_MICPC            ------------------------------------------------------------    362
tr|A0A7G2CNI0|A0A7G2CNI0_9TRYP    ------------------------------------------------------------    364
tr|S9TUR0|S9TUR0_9TRYP            ------------------------------------------------------------    521
tr|A0A0M9FXG9|A0A0M9FXG9_LEPPY    ------------------------------------------------------------    467
tr|A4H8K3|A4H8K3_LEIBR            ------------------------------------------------------------    457
tr|A4HWX6|A4HWX6_LEIIN            ------------------------------------------------------------    457
tr|Q4QEU0|Q4QEU0_LEIMA            ------------------------------------------------------------    457
sp|O81862-2|CHIC_ARATH            ------------------------------------------------------------    337
tr|S8AUV1|S8AUV1_PENO1            ------------------------------------------------------------    400
tr|A0A179UX46|A0A179UX46_BLAGS    ------------------------------------------------------------    496
tr|M9NG70|M9NG70_DROME            SGSTLRSTTSAGHSAPPLSRRQQSRRRPHKYIEVYSRPPAKTAVISATSSSQFLDEGFPV    1276
tr|Q22468|Q22468_CAEEL            ------------------------------------------------------------    626

tr|A0A0S4J833|A0A0S4J833_BODSA    ------------------------------------------------------------    363
tr|A0A250X009|A0A250X009_9CHLO    ------------------------------------------------------------    336
tr|C5K6G1|C5K6G1_PERM5            ------------------------------------------------------------    329
tr|C1N616|C1N616_MICPC            ------------------------------------------------------------    362
tr|A0A7G2CNI0|A0A7G2CNI0_9TRYP    ------------------------------------------------------------    364
tr|S9TUR0|S9TUR0_9TRYP            ------------------------------------------------------------    521
tr|A0A0M9FXG9|A0A0M9FXG9_LEPPY    ------------------------------------------------------------    467
tr|A4H8K3|A4H8K3_LEIBR            ------------------------------------------------------------    457
tr|A4HWX6|A4HWX6_LEIIN            ------------------------------------------------------------    457
tr|Q4QEU0|Q4QEU0_LEIMA            ------------------------------------------------------------    457
sp|O81862-2|CHIC_ARATH            ------------------------------------------------------------    337
tr|S8AUV1|S8AUV1_PENO1            ------------------------------------------------------------    400
tr|A0A179UX46|A0A179UX46_BLAGS    ------------------------------------------------------------    496
tr|M9NG70|M9NG70_DROME            VHSQVAQRRRSGSILPTKNDPKVIVHGHGIIECRAQGNFPHPLNCRKFISCARFEETGGI    1336
tr|Q22468|Q22468_CAEEL            ------------------------------------------------------------    626

tr|A0A0S4J833|A0A0S4J833_BODSA    -------------------------------    363
tr|A0A250X009|A0A250X009_9CHLO    -------------------------------    336
tr|C5K6G1|C5K6G1_PERM5            -------------------------------    329
tr|C1N616|C1N616_MICPC            -------------------------------    362
tr|A0A7G2CNI0|A0A7G2CNI0_9TRYP    -------------------------------    364
tr|S9TUR0|S9TUR0_9TRYP            -------------------------------    521
```

```
tr|A0A0M9FXG9|A0A0M9FXG9_LEPPY     -------------------------------    467
tr|A4H8K3|A4H8K3_LEIBR             -------------------------------    457
tr|A4HWX6|A4HWX6_LEIIN             -------------------------------    457
tr|Q4QEU0|Q4QEU0_LEIMA             -------------------------------    457
sp|O81862-2|CHIC_ARATH             -------------------------------    337
tr|S8AUV1|S8AUV1_PENO1             -------------------------------    400
tr|A0A179UX46|A0A179UX46_BLAGS     -------------------------------    496
tr|M9NG70|M9NG70_DROME             VGWEYTCPKGLTYDGVGGMCTWSPSDQPCRD    1367
tr|Q22468|Q22468_CAEEL             -------------------------------    626
```

(overall sequence identity = 0.0079)

04. MANF (ARMET) family proteins

These proteins are called by the utter misnomer "Mesencephalic astrocyte-secreted neurotrophic factor" in animals, despite the fact that they are not secreted by default, but retained in the ER by a KDEL signal. The real physiological function of these proteins is unclear. However, they are a well conserved group in animals and some other eukaryotes, hinting at a critical (possibly protein chaperoning) function. Our alignments show that both the protein and its KDEL signal is an ancient heritage in kinetoplastids: already found in the distinctly related euglenids. While missing from fungi and higher plants, MANF (ARMET) proteins are still seen among SAR supergroup (Stramenopila-Alveolata-Rhizaria) organisms, showing that its loss is likely secondary.

```
tr|C1EB43|C1EB43_MICCC             ------------------------------------------------------------    0
tr|A0A0L1KUF0|A0A0L1KUF0_9EUGL     ------------------------------------------------------------    0
tr|A0A0S4IZ22|A0A0S4IZ22_BODSA     ------------------------------------------------------------    0
tr|Q57VE7|Q57VE7_TRYB2             ------------------------------------------------------------    0
tr|A0A1X0NHR2|A0A1X0NHR2_9TRYP     ------------------------------------------------------------    0
tr|Q4D5D6|Q4D5D6_TRYCC             ---------------------------------------------------MPPR-----    4
tr|Q4CP18|Q4CP18_TRYCC             ---------------------------------------------------MPPR-----    4
tr|A0A3R7N5F9|A0A3R7N5F9_9TRYP     ------------------------------------------------------M-----    1
tr|A0A422N9T4|A0A422N9T4_TRYRA     ------------------------------------------------------M-----    1
tr|S9WF29|S9WF29_9TRYP             ------------------------------------------------------------    0
tr|S9UQX2|S9UQX2_9TRYP             ------------------------------------------------------------    0
tr|S9UU37|S9UU37_9TRYP             --------------------------------MCAT--------F-----SSPPTFFFLL    15
tr|A0A0M9GAG4|A0A0M9GAG4_LEPPY     ------------------------------------------------------------    0
tr|A4HME3|A4HME3_LEIBR             ------------------------------------------------------------    0
tr|E9AEU4|E9AEU4_LEIMA             ------------------------------------------------------------    0
tr|A4IB15|A4IB15_LEIIN             ------------------------------------------------------------    0
tr|A0A833SEV5|A0A833SEV5_PHYIN     --MLTKHVMLLALVALVLGNAPYVQADNKECEVCVKVIDDLKATYAQLQEENPKGKTQAL    58
sp|Q9CXI5|MANF_MOUSE               --MWATRGLAVALALSVLP--DSRALRPGDCEVCISYLGRFYQDL---KD-RDVTFSPAT    52
sp|Q9XZ63|ARMET_DROME              MKTWYMVV-VIGFLATLAQ--TSLALKEEDCEVCVKTVRRFADSL---DD-ST-KKDYKQ    52

tr|C1EB43|C1EB43_MICCC             ---------------------------MR---------GWLKIAFIALL-----------    13
tr|A0A0L1KUF0|A0A0L1KUF0_9EUGL     ----------------------------M---------SFP-----ALL-----------    7
tr|A0A0S4IZ22|A0A0S4IZ22_BODSA     ----------------------------M---------QRSGLALLLCA-----------    12
tr|Q57VE7|Q57VE7_TRYB2             ----------M--------FSMSPCVL-------------TALLAAAVL-----------    18
tr|A0A1X0NHR2|A0A1X0NHR2_9TRYP     --------------------MRDIGLR-------------PLLLLL-VL-----------    15
tr|Q4D5D6|Q4D5D6_TRYCC             ---------RY--------LRNGAALL-------------PWLL--VLL-----------    21
tr|Q4CP18|Q4CP18_TRYCC             ---------RY--------LRNGAAPF-------------PWLL--VLL-----------    21
tr|A0A3R7N5F9|A0A3R7N5F9_9TRYP     ---------SH--------LRDRA----------------PLLLLLLLL-----------    17
tr|A0A422N9T4|A0A422N9T4_TRYRA     ---------PH--------LRDRVAVL-------------PLLFVLLLL-----------    20
tr|S9WF29|S9WF29_9TRYP             ---------------------------------------MRKLFIVAVL-----------    10
tr|S9UQX2|S9UQX2_9TRYP             ----------------------------M---------QRKLYLFLALT-----------    12
tr|S9UU37|S9UU37_9TRYP             VDFRFFFLCSY--------LCFRAALLKM---------QRKLYLFLALT-----------    47
tr|A0A0M9GAG4|A0A0M9GAG4_LEPPY     ------------------------------------------------------------    0
tr|A4HME3|A4HME3_LEIBR             ----------------------------------------MKAFFITVL-----------    9
tr|E9AEU4|E9AEU4_LEIMA             ----------------------------------------MKLFFAAVL-----------    9
tr|A4IB15|A4IB15_LEIIN             ----------------------------------------MKLFFAAVL-----------    9
tr|A0A833SEV5|A0A833SEV5_PHYIN     AEKAVTKLCGKKLSTKDNKLCYNLEPLKK-------DVARQVTFKKDTLKICKSLEKKNP    111
sp|Q9CXI5|MANF_MOUSE               IEEELIKFCRE-ARGKENRLCYYIGATDDAATKIINEVSKPLAHHIPVEKICEKLKKKDS    111
sp|Q9XZ63|ARMET_DROME              IETAFKKFCKA-QKNKEHRFCYYLGGLEESATGILNELSKPLSWSMPAEKICEKLKKKDA    111

tr|C1EB43|C1EB43_MICCC             ALLATPQCRAEEHEMPNFKTMKIKELKAILADRGRECKGCAEKADYVAMAADVWSLPIVE    73
tr|A0A0L1KUF0|A0A0L1KUF0_9EUGL     AVSILACHAAHCITDDEIRKMKMKELKEFLDDRDESCSGCVEKGDFVRIAIEVKDKKISQ    67
tr|A0A0S4IZ22|A0A0S4IZ22_BODSA     ALLFSAVTQAFAESEAEFKKMNVRQLKKFLDERDVDYRDITEKSEFVSRAVEWAGRN---    69
tr|Q57VE7|Q57VE7_TRYB2             AVLPLSSFASPELTEADFKRMKIKELRNFLEDRGLTCPGCQEKADFVRVAFTNRAKKPLS    78
tr|A0A1X0NHR2|A0A1X0NHR2_9TRYP     LFYLVCLTAMAEMTEQDFKRMKIKDLRHFLEERDLSCPGCQEKADFVRVAFQNRDKKPVS    75
tr|Q4D5D6|Q4D5D6_TRYCC             LMAFMVTATAAELTEADFKRMKIRELRDFLEDRGLTCPDCQEKADFARYAYQNRDKKPTS    81
tr|Q4CP18|Q4CP18_TRYCC             WMAFMVTAAAAELTEADFKRMKIRELRDFLEDRGLTCPDCQEKADFARYAYQNRDKKPTS    81
tr|A0A3R7N5F9|A0A3R7N5F9_9TRYP     LMVSVGPAAAAELTEADFKRMKIRELRRFLGERGLTCVGCQEKSDFSRYAYQNRDKKPLS    77
```

```
tr|A0A422N9T4|A0A422N9T4_TRYRA      QMAFVGPVTAAKLTEADFKRMKIRDLRSFLEERGLTCVGCQEKADFSRYAYQNRDKKPLS    80
tr|S9WF29|S9WF29_9TRYP              FALL--IAVNASLTEDDFKKMKMKDLRIFLSSRGLECVGCLEKSDFVRLAFQNKDKQPLA    68
tr|S9UQX2|S9UQX2_9TRYP              LCLLGAFVSASDMTMADFKKMKIKDLRTFLDERGLECNGCQEKSDFVRVAYEYRDKAPLG    72
tr|S9UU37|S9UU37_9TRYP              LCLLGAFVSASDMTMADFKKMKIKDLRTFLDERGLECNGCQEKSDFVRVAYEYRDKAPLG    107
tr|A0A0M9GAG4|A0A0M9GAG4_LEPPY      --------------------MKVKDLRIFLSDRGLECSGCQEKSDFVRMAHQYRSLNPAG    40
tr|A4HME3|A4HME3_LEIBR              VAILAATLVSAGMTESDFKKMKVKDIRAFLMDRGLECIGCQEKSDFVRMAYQHRDKSPIG    69
tr|E9AEU4|E9AEU4_LEIMA              IAVFAVILVSAGMTESDFKRMKVKDLRAFLQDRGLECAGCQEKSDFVRMAYQHRDTNPVG    69
tr|A4IB15|A4IB15_LEIIN              IAVFAVTLVSAGMTESDFKKMKVKDLRAFLQDRGLECAGCQEKSDFVRMAYQHRDTNPVG    69
tr|A0A833SEV5|A0A833SEV5_PHYIN      DFCSMRYPVKTDAN-TDYSKMRVKQLRKILAERGVECVGCVEKSDFIAKIKDTESLHTEL    170
sp|Q9CXI5|MANF_MOUSE                QICELKYDKQIDLSTVDLKKLRVKELKKILDDWGEMCKGCAEKSDYIRKINELMPKYAPK    171
sp|Q9XZ63|ARMET_DROME               QICDLRYEKQIDLNSVDLKKLKVRDLKKILNDWDESCDGCLEKGDFIKRIEELKPKYSRS    171
                                                             :.::::: :* . .   .  **.::

tr|C1EB43|C1EB43_MICCC              KPA-SGDETKEKPAEPDLND----------------------------ADQERIRRMMDE    104
tr|A0A0L1KUF0|A0A0L1KUF0_9EUGL      EKQKMKGYTGEYPKKSFWDFWTEESLQIAAS----------SELDTKGRKLIADAVETCF    117
tr|A0A0S4IZ22|A0A0S4IZ22_BODSA      KKV-----VRDLPKEPFWEVWAKISRDKCEAAVATKGLGES---GAKVCDAVASAVDSFF    121
tr|Q57VE7|Q57VE7_TRYB2              EEG-----KREIPKAPLWEVWRDNAKLVCEEAAKKRGLDVTAKPQSDICSAVALVVENFF    133
tr|A0A1X0NHR2|A0A1X0NHR2_9TRYP      EQG-----KREIPNASFWEVWKDNAKALCTEVVQKRGLDVSGKPQADICDAIAYVVENFF    130
tr|Q4D5D6|Q4D5D6_TRYCC              EQG-----KREVPNAPFWEVWRDIAKEVCEEAVKKRGLDVSAEPQSEVCSALAYVTESFF    136
tr|Q4CP18|Q4CP18_TRYCC              EQG-----KREVPNASFWEVWRDIAKEVCEEAVKKRGLDVSAEPQSEVCSALAYVTESFF    136
tr|A0A3R7N5F9|A0A3R7N5F9_9TRYP      EQG-----KRDVPDAPFWAVWRDLAKEVCEGAVRKRGLDVSAAPQSDVCAALAYVTESFF    132
tr|A0A422N9T4|A0A422N9T4_TRYRA      EQG-----KREVPDAPFWVVWRDIAKEVCEEAVKKRGLDVTSTPQSDVCNAIVYVTESFF    135
tr|S9WF29|S9WF29_9TRYP              SAT-----KREIPEGKFWEVWEANAKTKCEEAVKKRGGDPASKPFSDVCHTIEKAVEGYF    123
tr|S9UQX2|S9UQX2_9TRYP              IAA-----KREVPDKKFWEAWGANGKVQCEEAVTKRGSDPAAEPFVHVCDTIEKAIDSFF    127
tr|S9UU37|S9UU37_9TRYP              IAA-----KREVPDKKFWEAWGANGKVQCEEAVTKRGSDPAAEPFVHVCDTIEKAIDSFF    162
tr|A0A0M9GAG4|A0A0M9GAG4_LEPPY      SAE-----KRAVPAKKFWEAWADIAHAECEKAVRLRSNDPTTEPFKSVCSTLRSATDSYL    95
tr|A4HME3|A4HME3_LEIBR              SAK-----KREIPSKKFWEAWSDIAKQECQNAVKRRGNDADAEPFSIICDTIHSAADSYL    124
tr|E9AEU4|E9AEU4_LEIMA              SAV-----KREIPNKKFWEAWGDIAKNECQNAVRRRGNDEGTEPFSTICDTIHSAVDSYL    124
tr|A4IB15|A4IB15_LEIIN              SAV-----KREIPNKKFWEAWGDIAKNECQNAVRRRGNDEGTEPFSTICDTIHSAVDSYL    124
tr|A0A833SEV5|A0A833SEV5_PHYIN      ------------------------------------------------------------    170
sp|Q9CXI5|MANF_MOUSE                AASARTD----L------------------------------------------------    179
sp|Q9XZ63|ARMET_DROME               EL----------------------------------------------------------    173


tr|C1EB43|C1EB43_MICCC              MQNGPRPTGDPERD--AILKKLHSSGIKFSGGEGMPLDQLRNL----------EKAMGNI    152
tr|A0A0L1KUF0|A0A0L1KUF0_9EUGL      MQYGKSVATKLKKGPAELLK-TSLKSPYYQAGIRG-IQKLITLCASSPSLKNA-------    168
tr|A0A0S4IZ22|A0A0S4IZ22_BODSA      MMNGKRTASKLKKKPDALTK-TANGDIYYNAGSRI-IARLLGYCLNAK-NRASCSSSSQV    178
tr|Q57VE7|Q57VE7_TRYB2              MQHGKRVANKLRKNHEALLK-TSYKNVYYDAGHVL-LKRLTEYCLVSEENQNKCSSIGSL    191
tr|A0A1X0NHR2|A0A1X0NHR2_9TRYP      MQHGKRTANKLRKKADDLLK-TSYKNVYYDAGRVL-LERLANYCLASPANQEKCSSVGSL    188
tr|Q4D5D6|Q4D5D6_TRYCC              LQHGKRTAGKLRKKPEALLK-TSFKAVYYDAGRVL-LGRLADHCLASAGNQNTCSSMSKL    194
tr|Q4CP18|Q4CP18_TRYCC              LQHGKRTAGKLRKKPEALLK-TSFKAVYYDAGRVL-LGRLADHCLASAGNQNTCSSMSKL    194
tr|A0A3R7N5F9|A0A3R7N5F9_9TRYP      MQHGKRTASKLRKKPEALLK-TSFKGVYYDAGRLL-LERLANYCLASADHQSTCSSVGKL    190
tr|A0A422N9T4|A0A422N9T4_TRYRA      MQHGKRTANKLRKKPDALLK-TSHKGVYYDSGRLL-LERLVNYCLASADHQVKCSSVSKL    193
tr|S9WF29|S9WF29_9TRYP              MQNGRQVATKLKKQPLQLLK-TSYKDIYYDAGLRL-LEKLSNHCLSTPDQQKKCSSMSNV    181
tr|S9UQX2|S9UQX2_9TRYP              MQHGRQTANRLKKTPHHMLK-TSYKHVYYDVGVRL-LNKLINYCLASPSIQSKCESLSHV    185
tr|S9UU37|S9UU37_9TRYP              MQHGRQTANRLKKTPHHMLK-TSYKHVYYDVGVRL-LNKLINYCLASPSIQSKCESLSHV    220
tr|A0A0M9GAG4|A0A0M9GAG4_LEPPY      MQHGRKVANQLKKTPHHLLQ-TSFKDIYFEAGSHL-FQILADYCLASPAAQENCQSLGAV    153
tr|A4HME3|A4HME3_LEIBR              MQHGRRVANQLKKTPLHLLE-TSFKDVYYEAGLHL-FQTLADYCLASPSSQTACQSLGSV    182
tr|E9AEU4|E9AEU4_LEIMA              MQHGRRVANQLKKTPQDLLR-TSFKDVYFEAGSHL-FQMLADYCLGSPSLQKACQSLGSV    182
tr|A4IB15|A4IB15_LEIIN              MQHGRRVANQLKKTPQDLLK-TSFKDVYFEAGSHL-FQTLADYCLGSPSLQKACQSLGSV    182
tr|A0A833SEV5|A0A833SEV5_PHYIN      ------------------------------------------------------------    170
sp|Q9CXI5|MANF_MOUSE                ------------------------------------------------------------    179
sp|Q9XZ63|ARMET_DROME               ------------------------------------------------------------    173


tr|C1EB43|C1EB43_MICCC              KTKKQGDGDDE--L----------------------------    164
tr|A0A0L1KUF0|A0A0L1KUF0_9EUGL      ----ELQNACEKEFVPWITNVGIENTNPMYEILEQMKSEL--    204
tr|A0A0S4IZ22|A0A0S4IZ22_BODSA      VELMDKDTVKGTGFGAWITNVGIENTNPMYELSNSKSLHDEL    220
tr|Q57VE7|Q57VE7_TRYB2              TTMLES--GKMVDFAKWMTNVGIENTNPMYEVLDGRGDL---    228
tr|A0A1X0NHR2|A0A1X0NHR2_9TRYP      SSLIEG--SSVIDLVKWMTNVGIENTNPMYDFLELRDDL---    225
tr|Q4D5D6|Q4D5D6_TRYCC              MALTEE--SKIADLAKWMTNVGIENTNPMYEFIDQRDDL---    231
tr|Q4CP18|Q4CP18_TRYCC              MALAEE--SKIADLAKWMTNVGIENTNPMYEFIDQRDDL---    231
tr|A0A3R7N5F9|A0A3R7N5F9_9TRYP      MTLAEE--AKVVDLQGWMTNVGIENTNPMYELLDRRDDL---    227
tr|A0A422N9T4|A0A422N9T4_TRYRA      MTLAEE--AKVVDLQGWMTNVGIENTNPMYELLDRRDDL---    230
tr|S9WF29|S9WF29_9TRYP              MKLLE-----DEGFKVYITNVGIENTNPMYDIIDDAADL---    215
tr|S9UQX2|S9UQX2_9TRYP              LGVMEG--NKEGNFKSWITNVGIENTNPMYEILDDASDL---    222
tr|S9UU37|S9UU37_9TRYP              LGVMEG--NKEGNFKSWITNVGIENTNPMYEILDDASDL---    257
tr|A0A0M9GAG4|A0A0M9GAG4_LEPPY      MSAMDG--ACGADFKMWTTNVGIENTNPMYEIIDTRDDL---    190
tr|A4HME3|A4HME3_LEIBR              LSTMDG--SSGANFKVWTTNVGIENTNPMYDIIGEHADL---    219
tr|E9AEU4|E9AEU4_LEIMA              MSAMDG--SSGADFRIWTTNVGIENTNPMYEIIDGRDDL---    219
tr|A4IB15|A4IB15_LEIIN              MSAMDG--SSGADFRIWTTNVGIENTNPMYEIIDGRDDL---    219
tr|A0A833SEV5|A0A833SEV5_PHYIN      ------------------------------------------    170
sp|Q9CXI5|MANF_MOUSE                ------------------------------------------    179
sp|Q9XZ63|ARMET_DROME               ------------------------------------------    173
```

(overall sequence identity = 0.0088)

05. ER glutathionylspermidine amidase domain (ERGAD)

These proteins, technically CHAP (C51) domain peptidases have a lot of close relatives in bacteria, and even within kinetoplastids. The latter paralogs are the well-

known bifunctional trypanothione synthase enzymes (called glutathionylspermidine synthases/amidases). The distinction between the two subfamilies (Type II transmembrane versus soluble, ER-retained) is clear-cut in all kinetoplastid proteomes. Therefore this paralog has likely functionally distinct roles in trypanothione metabolism. The family can be more distantly traced back to prokaryotic D-alanyl-glycine endopeptidases, but it is sporadically found in multiple eukaryotic groups as well (typically without KDEL). It is unclear if its limited occurrance animals (only in rotifer worms) and other groups (e.g. pseudofungi) points to horizontal gene transfer events.

```
tr|A0A815F576|A0A815F576_9BILA     ------------------------------------------------------------    0
tr|A0A814QMX3|A0A814QMX3_9BILA     ------------------------------------------------------------    0
tr|K2MZ59|K2MZ59_TRYCR             ------------------------------------------------------------    0
tr|A0A0G4IUR7|A0A0G4IUR7_PLABS     ------------------------------------------------------------    0
tr|A0A0N0DV74|A0A0N0DV74_LEPPY     ------------------------------------------------------------    0
tr|A4HEJ1|A4HEJ1_LEIBR             ------------------------------------------------------------    0
tr|Q4Q9R0|Q4Q9R0_LEIMA             ------------------------------------------------------------    0
tr|A4I1M7|A4I1M7_LEIIN             ------------------------------------------------------------    0
tr|S9UNH8|S9UNH8_9TRYP             ------------------------------------------------------------    0
tr|S9UZF8|S9UZF8_9TRYP             ------------------------------------------------------------    0
tr|Q57ZC4|Q57ZC4_TRYB2             ------------------------------------------------------------    0
tr|A0A3R7LGJ5|A0A3R7LGJ5_9TRYP     ------------------------------------------------------------    0
tr|Q4CUZ5|Q4CUZ5_TRYCC             ------------------------------------------------------------    0
tr|Q4D7T1|Q4D7T1_TRYCC             ------------------------------------------------------------    0
tr|A0A3R7KMG1|A0A3R7KMG1_TRYRA     ------------------------------------------------------------    0
tr|Q4CYQ4|Q4CYQ4_TRYCC             ------------------------------------------------------------    0
tr|Q4CUZ4|Q4CUZ4_TRYCC             ------------------------------------------------------------    0
tr|Q4CUZ2|Q4CUZ2_TRYCC             ------------------------------------------------------------    0
tr|Q4CYQ2|Q4CYQ2_TRYCC             ------------------------------------------------------------    0
tr|Q4D7T2|Q4D7T2_TRYCC             ------------------------------------------------------------    0
tr|Q4D7T0|Q4D7T0_TRYCC             ------------------------------------------------------------    0
tr|A0A1X0NY88|A0A1X0NY88_9TRYP     ------------------------------------------------------------    0
tr|A0A1X0NYK9|A0A1X0NYK9_9TRYP     ------------------------------------------------------------    0
tr|A0A1X0NRB4|A0A1X0NRB4_9TRYP     ---------------------------------MHNNPPIT-------------------    8
tr|Q4D408|Q4D408_TRYCC             ---------------------------------------MN-------------------    2
tr|A0A422N172|A0A422N172_9TRYP     ---------------------------------------MK-------------------    2
tr|A0A422NKH5|A0A422NKH5_TRYRA     ----------------------------------------M-------------------    1
tr|A0A0S4ITA7|A0A0S4ITA7_BODSA     ------------------------------------------------------------    0
tr|A0A0N0DYP4|A0A0N0DYP4_LEPPY     ---------------------------------------MRNRRSSAPDVCRKNDA----    17
tr|A4HLZ6|A4HLZ6_LEIBR             ---------------------------------------MRSRRTASGAAEGPVGDAAAN    21
tr|Q4Q3Q0|Q4Q3Q0_LEIMA             MFPFAFEYPCEATFAHQMQNKQPSTLLASFFAYYHFLIPMRSRRSPAGDIEDRADVAAAT    60
tr|A4I9C6|A4I9C6_LEIIN             -----------------MKNKQPTTVFASLFAYHHFLIPMRSRQPPSGGVEDHADVAAST    43
tr|A0A0N0DYQ7|A0A0N0DYQ7_LEPPY     ------------------------------------MLPNNMGNSNPGSGGREAGDAAVP    24
tr|A4HLZ4|A4HLZ4_LEIBR             ------------------------------------------------------------    0
tr|Q4Q3Q2|Q4Q3Q2_LEIMA             ------------------------------------------------------------    0
tr|A4I9C4|A4I9C4_LEIIN             -------------------------------------------------------MAAAP    5
tr|Q384E7|Q384E7_TRYB2             ------------------------------------------------------------    0
tr|Q586Y2|Q586Y2_TRYB2             ------------------------------------------------------------    0
tr|Q586Y1|Q586Y1_TRYB2             ------------------------------------------------------------    0
tr|F9W970|F9W970_TRYCI             ------------------------------------------------------------    0
tr|F9W971|F9W971_TRYCI             ------------------------------------------------------------    0
tr|S9UGT3|S9UGT3_9TRYP             ------------------------------------------------------------    0
tr|A0A1X0NR96|A0A1X0NR96_9TRYP     ------------------------------------------------------------    0
tr|A0A1X0NR98|A0A1X0NR98_9TRYP     ------------------------------------------------------------    0
tr|Q4D409|Q4D409_TRYCC             ------------------------------------------------------------    0
tr|Q4D410|Q4D410_TRYCC             ------------------------------------------------------------    0
tr|Q4CKB1|Q4CKB1_TRYCC             ------------------------------------------------------------    0
tr|Q4DEC6|Q4DEC6_TRYCC             ------------------------------------------------------------    0
tr|A0A3R7N0H0|A0A3R7N0H0_9TRYP     ------------------------------------------------------------    0
tr|A0A3R7N2W0|A0A3R7N2W0_9TRYP     ------------------------------------------------------------    0
tr|A0A3S5IRC8|A0A3S5IRC8_TRYRA     ------------------------------------------------------------    0

tr|A0A815F576|A0A815F576_9BILA     ------------------------------------------------------------    0
tr|A0A814QMX3|A0A814QMX3_9BILA     ------------------------------------------------------------    0
tr|K2MZ59|K2MZ59_TRYCR             ------------------------------------------------------------    0
tr|A0A0G4IUR7|A0A0G4IUR7_PLABS     ------------------------------------------------------------    0
tr|A0A0N0DV74|A0A0N0DV74_LEPPY     ------------------------------------------------------------    0
tr|A4HEJ1|A4HEJ1_LEIBR             ------------------------------------------------------------    0
tr|Q4Q9R0|Q4Q9R0_LEIMA             ------------------------------------------------------------    0
tr|A4I1M7|A4I1M7_LEIIN             ------------------------------------------------------------    0
tr|S9UNH8|S9UNH8_9TRYP             ------------------------------------------------------------    0
tr|S9UZF8|S9UZF8_9TRYP             ------------------------------------------------------------    0
tr|Q57ZC4|Q57ZC4_TRYB2             ------------------------------------------------------------    0
tr|A0A3R7LGJ5|A0A3R7LGJ5_9TRYP     ------------------------------------------------------------    0
tr|Q4CUZ5|Q4CUZ5_TRYCC             ------------------------------------------------------------    0
tr|Q4D7T1|Q4D7T1_TRYCC             ------------------------------------------------------------    0
tr|A0A3R7KMG1|A0A3R7KMG1_TRYRA     ------------------------------------------------------------    0
tr|Q4CYQ4|Q4CYQ4_TRYCC             ------------------------------------------------------------    0
tr|Q4CUZ4|Q4CUZ4_TRYCC             ------------------------------------------------------------    0
```

```
tr|Q4CUZ2|Q4CUZ2_TRYCC              ------------------------------------------------------------    0
tr|Q4CYQ2|Q4CYQ2_TRYCC              ------------------------------------------------------------    0
tr|Q4D7T2|Q4D7T2_TRYCC              ------------------------------------------------------------    0
tr|Q4D7T0|Q4D7T0_TRYCC              ------------------------------------------------------------    0
tr|A0A1X0NY88|A0A1X0NY88_9TRYP      ------------------------------------------------------------    0
tr|A0A1X0NYK9|A0A1X0NYK9_9TRYP      ------------------------------------------------------------    0
tr|A0A1X0NRB4|A0A1X0NRB4_9TRYP      ---------------TETSTGPQNSRSSASSYSAT-----GREGANERTPMLGIRPMTV-    47
tr|Q4D408|Q4D408_TRYCC              ---------------SAVN------ASPSVTVSNL-----SAPPPNECTPMLRTRPVPM-    35
tr|A0A422N172|A0A422N172_9TRYP      ---------------RAVA------AP-------P-----PAAAPNERTPMLHPRPLPV-    28
tr|A0A422NKH5|A0A422NKH5_TRYRA      ---------------RAVE------VSPPATASKE-----AAAASTERTPLLKFHPRPI-    34
tr|A0A0S4ITA7|A0A0S4ITA7_BODSA      ---------------------------------------------------MAYNSTKVD    9
tr|A0A0N0DYP4|A0A0N0DYP4_LEPPY      --SDVRS------------------SSPVVGG----------------------------    29
tr|A4HLZ6|A4HLZ6_LEIBR              APHRLRS------------------PSPPSASSS--------------------------    37
tr|Q4Q3Q0|Q4Q3Q0_LEIMA              VLHSSSS------------------SSSAAAAAS--------------------------    76
tr|A4I9C6|A4I9C6_LEIIN              VLRSSAS------------------SSSAAAAAS--------------------------    59
tr|A0A0N0DYQ7|A0A0N0DYQ7_LEPPY      FLQRIRSESDTAEGNAAVSVQRNHSSSPHTATACYGGTETHALR--------KRNPKVSD    76
tr|A4HLZ4|A4HLZ4_LEIBR              ---------------------------------------------------MGGATRFV-    8
tr|Q4Q3Q2|Q4Q3Q2_LEIMA              ------------------------------------------------------------    0
tr|A4I9C4|A4I9C4_LEIIN              LSRTF---------------------SPKSSESSFSSHSQERMEQNYRESAAGFGPPPA-    43
tr|Q384E7|Q384E7_TRYB2              ---------------------------------------------MAKGS----------    5
tr|Q586Y2|Q586Y2_TRYB2              ---------------------------------------------MAARK----------    5
tr|Q586Y1|Q586Y1_TRYB2              ---------------------------------------------MEREP----------    5
tr|F9W970|F9W970_TRYCI              ------------------------------------------------------------    0
tr|F9W971|F9W971_TRYCI              ---------------------------------------------MQRET----------    5
tr|S9UGT3|S9UGT3_9TRYP              ------------------------------------------------------------    0
tr|A0A1X0NR96|A0A1X0NR96_9TRYP      ------------------------------------------------------------    0
tr|A0A1X0NR98|A0A1X0NR98_9TRYP      ---------------------------------------------MQRDT-ELWAVPRK-    13
tr|Q4D409|Q4D409_TRYCC              ------------------------------------------------------------    0
tr|Q4D410|Q4D410_TRYCC              ------------------------------------------------------------    0
tr|Q4CKB1|Q4CKB1_TRYCC              ------------------------------------------------------------    0
tr|Q4DEC6|Q4DEC6_TRYCC              ------------------------------------------MGTLRENN-AGFGDEKM-    16
tr|A0A3R7N0H0|A0A3R7N0H0_9TRYP      ------------------------------------------MDTTKGDH-AHFGEEKM-    16
tr|A0A3R7N2W0|A0A3R7N2W0_9TRYP      ------------------------------------------------------------    0
tr|A0A3S5IRC8|A0A3S5IRC8_TRYRA      ------------------------------------------------------------    0


tr|A0A815F576|A0A815F576_9BILA      ------------------------------------------------------------    0
tr|A0A814QMX3|A0A814QMX3_9BILA      ------------------------------------------------------------    0
tr|K2MZ59|K2MZ59_TRYCR              ------------------------------------------------------------    0
tr|A0A0G4IUR7|A0A0G4IUR7_PLABS      ------------------------------------------------------------    0
tr|A0A0N0DV74|A0A0N0DV74_LEPPY      ------------------------------------------------------------    0
tr|A4HEJ1|A4HEJ1_LEIBR              -----------------------------------------------------------M    1
tr|Q4Q9R0|Q4Q9R0_LEIMA              ------------------------------------------------------------    0
tr|A4I1M7|A4I1M7_LEIIN              ------------------------------------------------------------    0
tr|S9UNH8|S9UNH8_9TRYP              ------------------------------------------------------------    0
tr|S9UZF8|S9UZF8_9TRYP              ------------------------------------------------------------    0
tr|Q57ZC4|Q57ZC4_TRYB2              -----------------------------------------------------------M    1
tr|A0A3R7LGJ5|A0A3R7LGJ5_9TRYP      ------------------------------------------------------------    0
tr|Q4CUZ5|Q4CUZ5_TRYCC              ------------------------------MLPVCIFF-----------FFLQAGMWISF    19
tr|Q4D7T1|Q4D7T1_TRYCC              ------------------------------------------------------------    0
tr|A0A3R7KMG1|A0A3R7KMG1_TRYRA      -------------------------------MPVSSLYSADGQGNKRCWLSFSAGAWCST    29
tr|Q4CYQ4|Q4CYQ4_TRYCC              -----------------------------------------------------------M    1
tr|Q4CUZ4|Q4CUZ4_TRYCC              -----------------------------------------------------------M    1
tr|Q4CUZ2|Q4CUZ2_TRYCC              ------------------------------------------------------------    0
tr|Q4CYQ2|Q4CYQ2_TRYCC              ------------------------------------------------------------    0
tr|Q4D7T2|Q4D7T2_TRYCC              -----------------------------------------------------------M    1
tr|Q4D7T0|Q4D7T0_TRYCC              ------------------------------------------------------------    0
tr|A0A1X0NY88|A0A1X0NY88_9TRYP      ------------------------------------------------------------    0
tr|A0A1X0NYK9|A0A1X0NYK9_9TRYP      -----------------------------------------------------------M    1
tr|A0A1X0NRB4|A0A1X0NRB4_9TRYP      --------------------------------------------EIDSTTVR---EDYYE    60
tr|Q4D408|Q4D408_TRYCC              --------------------------------------------EAESMLRR---EGQDE    48
tr|A0A422N172|A0A422N172_9TRYP      --------------------------------------------EVGSMQLR---EEMDE    41
tr|A0A422NKH5|A0A422NKH5_TRYRA      --------------------------------------------EVGSMQQQ---EEMDE    47
tr|A0A0S4ITA7|A0A0S4ITA7_BODSA      SHDEEDSNDDFVVGV--KM-----KKAPY--------------PWVDDNHHE-----RTS    43
tr|A0A0N0DYP4|A0A0N0DYP4_LEPPY      ---------------AARIPPRRHKELDVEDAALCDAYAINLRRESEDME-------LRR    67
tr|A4HLZ6|A4HLZ6_LEIBR              -------------LLHSASLFCARKESDSSNVDVCDAYAIRLRNEAVDAV-------RYR    77
tr|Q4Q3Q0|Q4Q3Q0_LEIMA              -------------MMQDQVLRCVRKEPGPSNAAICDAYAMRLLQEATEEE-------RCR    116
tr|A4I9C6|A4I9C6_LEIIN              -------------LLQDEVLPRLRKESGPSNAAICDAYATRLRQEATEEE-------RRR    99
tr|A0A0N0DYQ7|A0A0N0DYQ7_LEPPY      SGERSSKDDGDVVVVPSRAGGGGGGSSGNNSRPDAGRDVEGECIVVNDNGTDTTADTCMG    136
tr|A4HLZ4|A4HLZ4_LEIBR              -SSGASTEDDDVVVRHSRRYGGATIQRNTS----CEIDNERTLFVQQENKRTDSGVACFG    63
tr|Q4Q3Q2|Q4Q3Q2_LEIMA              ------------------------------------------------------------    0
tr|A4I9C4|A4I9C4_LEIIN              -ESASNAANGDVVVCQSRSHRETTAQPNTS----GNNYGESTRVGARDNKSADSGIASAG    98
tr|Q384E7|Q384E7_TRYB2              ------DYDAVAYEPMPREGFDAGDEGPL----------------VGNHDQGF--LFDDQ    41
tr|Q586Y2|Q586Y2_TRYB2              ----------A------------GTARPLLQREGEAR--------------NF----DS-    24
tr|Q586Y1|Q586Y1_TRYB2              ----------AVTSKTNRLSSNEKDPHDVPETNDGAI--------------NG--SFNNR    39
tr|F9W970|F9W970_TRYCI              -------------------------MTPRANTEGCDT--------------LL--G-DDR    18
tr|F9W971|F9W971_TRYCI              ----------EGRPKRSRASSERQDINDILQEDNCNN--------------DY--TTDLK    39
tr|S9UGT3|S9UGT3_9TRYP              ------------------------------------------------------------    0
tr|A0A1X0NR96|A0A1X0NR96_9TRYP      ------------------------------MAAQRKRKESGATRPVNSSK-KT--IMSSF    27
tr|A0A1X0NR98|A0A1X0NR98_9TRYP      ----------DVREETPF--PEEEEVNVT-IPNGEDLKTEANEEPVDSLE-KR--V----    53
tr|Q4D409|Q4D409_TRYCC              ------------------MLPHG--------------------ARMRTSKESC--FFTGL    20
tr|Q4D410|Q4D410_TRYCC              ------------------------------------------------------------    0
tr|Q4CKB1|Q4CKB1_TRYCC              ---------------------------------------------MQSNDSQD--TF---    10
tr|Q4DEC6|Q4DEC6_TRYCC              -----DHSNNSIRVSTPRMQPGEAELVNSLDLNGPTNEFAGEQVEMQSNDSQD--TF---    66
tr|A0A3R7N0H0|A0A3R7N0H0_9TRYP      -----DHAHNHGFEPTTEMHPREMYVSDEHNTHKEANAFVSEQVEINSNEAD-----EAM    66
tr|A0A3R7N2W0|A0A3R7N2W0_9TRYP      ------------------MSPREATKRTRQA------------PRVNKGAAT-------S    23
tr|A0A3S5IRC8|A0A3S5IRC8_TRYRA      ------------------MSLRQVKKRTRQV------------SCVNKGSFT-------R    23
```

```
tr|A0A815F576|A0A815F576_9BILA    ----------------------------------------MKIVL--LIVLFTIIYRY--    16
tr|A0A814QMX3|A0A814QMX3_9BILA    ------------------------------------------------------------    0
tr|K2MZ59|K2MZ59_TRYCR            ------------------------------------------------------------    0
tr|A0A0G4IUR7|A0A0G4IUR7_PLABS    ------------------------------------------------------------    0
tr|A0A0N0DV74|A0A0N0DV74_LEPPY    ---------------------------------MNIVRLA-FCCITAALLSLGALFYF--    24
tr|A4HEJ1|A4HEJ1_LEIBR            HRHKERTCTHIDSRHIAIKYPPPIRTH---TLIMKLGYVV-VFCACAALLSLGSLFYY--    55
tr|Q4Q9R0|Q4Q9R0_LEIMA            ---------------------------------MNLVRVS-LLCACTTLLCLSALYYY--    24
tr|A4I1M7|A4I1M7_LEIIN            ---------------------------------MNLVRVA-LLCACTTLLCLGALYYY--    24
tr|S9UNH8|S9UNH8_9TRYP            -----------------------MRIS---QVFP-------FLAGFLLIVCVSSVVYF--    25
tr|S9UZF8|S9UZF8_9TRYP            -----------------------MKVT---HLFA--------LTATAACILLIYTTRF--    24
tr|Q57ZC4|Q57ZC4_TRYB2            VSL-----NRK--QKFQLAVPAT------------------LLLFLFVYVLLGGGS----    32
tr|A0A3R7LGJ5|A0A3R7LGJ5_9TRYP    ------------------------MKS---KSIC-------KVVPLVCALLCGGYLLW--    24
tr|Q4CUZ5|Q4CUZ5_TRYCC            FFFFLGYCLSR--SQKQHTMVAGFVEN---KSLG-------RVACLAFVLICGVLLLW--    65
tr|Q4D7T1|Q4D7T1_TRYCC            -------------------MAAGFMEN---KSLG-------RVACLACVLICGVLLLW--    29
tr|A0A3R7KMG1|A0A3R7KMG1_TRYRA    MYRIR-----K--QALGLSTP-AFLVF---PLLM-------LLLLLLGYVMLGGR-----    66
tr|Q4CYQ4|Q4CYQ4_TRYCC            IHTAR-----K--KQFGLSAL-A--------LFV-------LLLFLLVCVTLGLH-----    33
tr|Q4CUZ4|Q4CUZ4_TRYCC            IHTAR-----K--KQFGLSAL-A--------LFV-------LLLFLLVCVTLGLH-----    33
tr|Q4CUZ2|Q4CUZ2_TRYCC            ------------------------------------------------------------    0
tr|Q4CYQ2|Q4CYQ2_TRYCC            ------------------------------------------------------------    0
tr|Q4D7T2|Q4D7T2_TRYCC            IHTAR-----K--KQFGLSAL-A--------LFV-------LLLFLLVCITLGLH-----    33
tr|Q4D7T0|Q4D7T0_TRYCC            ------------------------------------------------------------    0
tr|A0A1X0NY88|A0A1X0NY88_9TRYP    -------------------MP-ALV-----------------ILTLLVYIIIGIGK----    19
tr|A0A1X0NYK9|A0A1X0NYK9_9TRYP    MM--------K--STGEIAVL-ALL-----------------LLLAIYYLLISDANIL--    31
tr|A0A1X0NRB4|A0A1X0NRB4_9TRYP    EPLWHRLS-----------SP-LHPVFRKVHDHCHYEWIAALLSVITLLLLFVFVFYY--    106
tr|Q4D408|Q4D408_TRYCC            MPFWERVS-----------SP-LHPVFRKVQEHCHYEWVAALLSAIMLLTLFVFIFYL--    94
tr|A0A422N172|A0A422N172_9TRYP    VPYWERIY-----------SP-LHPVLSKVQEHCHYEWVAALLSAMMLLALFVFVFYL--    87
tr|A0A422NKH5|A0A422NKH5_TRYRA    MSFWERVS-----------SP-LHPVLNKVQEHCHYEWVAALLSAIMLVALFVIVFYL--    93
tr|A0A0S4ITA7|A0A0S4ITA7_BODSA    SSWLPRSL-----------RD-GILVC-------IPACALLLLVVL-FLPH---------    74
tr|A0A0N0DYP4|A0A0N0DYP4_LEPPY    PSFWRIRC-----------SS-SIFVG-------LFIWALIGLVVLFKVS----------    98
tr|A4HLZ6|A4HLZ6_LEIBR            RSYWHVHC-----------SP-SILVA-------LLIWVTIGLVVYTKLD----------    108
tr|Q4Q3Q0|Q4Q3Q0_LEIMA            GPHWRARC-----------SP-SILVA-------LLIWATIGLVVYMKVD----------    147
tr|A4I9C6|A4I9C6_LEIIN            GPHWRARC-----------SP-SILVA-------LLIWATIALVVYMKVD----------    130
tr|A0A0N0DYQ7|A0A0N0DYQ7_LEPPY    KVRHFLLN-----------SD-SALYA-------LLKWGLAVLLLF-FLSFVVFGMIY--    174
tr|A4HLZ4|A4HLZ4_LEIBR            AVRRFVLT-----------RD-PLLYG-------LLKWGLSVFLLL-FLTFILLGLFF--    101
tr|Q4Q3Q2|Q4Q3Q2_LEIMA            -------------------------------------------------------MLY--    3
tr|A4I9C4|A4I9C4_LEIIN            VARCFVLT-----------HD-PLLYG-------ILKWGLATLLLL-FLAFVVFGMLY--    136
tr|Q384E7|Q384E7_TRYB2            ADGAHSTQ-----------LT-YIAPSVK-----KPVYALGITLLF-LLVAVVLTVIL-Q    82
tr|Q586Y2|Q586Y2_TRYB2            ----YKL------------------YA-------GASFAVGVVIIL-CLMKVPRA----E    50
tr|Q586Y1|Q586Y1_TRYB2            KRGIKRLC--------------SIAQA-------IFAWTFGVLVLL-FISFIAFGVLYSG    77
tr|F9W970|F9W970_TRYCI            EVVRRKSC--------------PA-YF-------LL--SFFLVILL-AMFYHAASPLWKE    53
tr|F9W971|F9W971_TRYCI            DKWRSRVC--------------STASF-------AFAWAFGVLIVL-FLVFIAFGVLYSG    77
tr|S9UGT3|S9UGT3_9TRYP            -----------------------------------MKWSFHFLLL--FFLIIIVGMLY--    21
tr|A0A1X0NR96|A0A1X0NR96_9TRYP    AKPFKSLP--------------YYIIS-------LFIWGTVASLVCMLLLAKSSREEYSH    66
tr|A0A1X0NR98|A0A1X0NR98_9TRYP    ----DRAT--------------SVLAV-------IIGWTLCVLVV-LFLGFIVFGILY--    85
tr|Q4D409|Q4D409_TRYCC            PTWFYSLS--------------HFFVA-------LGIWGSITCMVL-FLLTTSLRSKK-S    57
tr|Q4D410|Q4D410_TRYCC            ------------------------------------------------------------    0
tr|Q4CKB1|Q4CKB1_TRYCC            KKRMSRIN--------------AILTS-------VVIWSFLLLTLL-FLASIVFGIVY-S    47
tr|Q4DEC6|Q4DEC6_TRYCC            KKRMSRIN--------------AILTS-------VVIWSFLLLTLL-FLASIVFGIVY-S    103
tr|A0A3R7N0H0|A0A3R7N0H0_9TRYP    RKKFSRVN-----------AT-LTMVA-------GWSFGVLTLLFL-AFIV--FG-IL-Y    102
tr|A0A3R7N2W0|A0A3R7N2W0_9TRYP    MLWCPSLP--------------YCVIA-------VGIWGLLACLMA-LLLTRTSHLSR-L    60
tr|A0A3S5IRC8|A0A3S5IRC8_TRYRA    RLRWPSLS--------------CCIIA-------PGILGLLACLVA-LLHT-MCPIRQ-L    59


tr|A0A815F576|A0A815F576_9BILA    SKCDIVTQLKNF---------DSNLNSINARAFGNSTLAAYGAMVGIA-STNVPAYSNGN    66
tr|A0A814QMX3|A0A814QMX3_9BILA    ------------------------------MSNNVNTCVPFNEIEGIA-STNVPAYSNKK    29
tr|K2MZ59|K2MZ59_TRYCR            ---------------------MDNTDTQRGVHASETEHVSFGGVIGVT-DSGVPVFSNGE    38
tr|A0A0G4IUR7|A0A0G4IUR7_PLABS    ----------MW---------WPAVIVVAAVVVGADARAPFGDVLGVS-HGGVQVRSCHT    40
tr|A0A0N0DV74|A0A0N0DV74_LEPPY    STYDPEKHM------------LMRARVYSDYDPLEHCVTPFGVVLGYS--NGVPAFSNCI    70
tr|A4HEJ1|A4HEJ1_LEIBR            SVYDYQKHM------------SMVQRTYSTYDPLTDCAAPFGQLLGVA--DDVPAYSNCN    101
tr|Q4Q9R0|Q4Q9R0_LEIMA            SMYDYEKHM------------NMVQRKYSVYDPLTDCATPFGQLLGVA--DDVPAYSNCN    70
tr|A4I1M7|A4I1M7_LEIIN            SVYDYEKHM------------SMVQRKYSGYDPLTDCATPFGQLLGVA--DDVPAYSNCN    70
tr|S9UNH8|S9UNH8_9TRYP            ARDDYERHL------------LNQHKKYDTFDPLTNCTTPFDTLLGIA--DHVPAYSNCH    71
tr|S9UZF8|S9UZF8_9TRYP            AWSDYRPAV------------GTREREYDAYDPLTDCVTPFGTVIGYV--GPVPVLSNCN    70
tr|Q57ZC4|Q57ZC4_TRYB2            -----TQKS-------------KRHGMHMDFNPLEHCNAPMDSVVGYS--SDVPSMSNCH    72
tr|A0A3R7LGJ5|A0A3R7LGJ5_9TRYP    GWRGSTGGA-------------PSRETDPHFDPLEHCSTDLGTLLGYA--FSVPAFSNCH    69
tr|Q4CUZ5|Q4CUZ5_TRYCC            GWRGNKRNP-------------LSAEGNPLFDPLKHCRTRYGTILGYA--LSVPAFSNCH    110
tr|Q4D7T1|Q4D7T1_TRYCC            GWRGNKRNP-------------LSAEGDPLFDPLKHCRTRYGTILGYA--LSVPAFSNCH    74
tr|A0A3R7KMG1|A0A3R7KMG1_TRYRA    -WWRHTAGS-------------EKNSAQPSFNPLDHCGTPYGTLLGYA--HGIPAFSNCH    110
tr|Q4CYQ4|Q4CYQ4_TRYCC            -WWRDTSGP-------------ALHPPRSRFDPLAHCATPHGTLLGYA--LDIPAFSNCH    77
tr|Q4CUZ4|Q4CUZ4_TRYCC            -WWRDTSGP-------------ALHPPRSRFDPLAHCATPHGTLLGYA--LDIPAFSNCH    77
tr|Q4CUZ2|Q4CUZ2_TRYCC            ------------------------------------------------------------    0
tr|Q4CYQ2|Q4CYQ2_TRYCC            ------------------------------------------------------------    0
tr|Q4D7T2|Q4D7T2_TRYCC            -WWRDTSGP-------------ALHPPRSRFDPLAHCATPHGTLLGYA--LGIPAFSNCH    77
tr|Q4D7T0|Q4D7T0_TRYCC            ------------------------------------------------------------    0
tr|A0A1X0NY88|A0A1X0NY88_9TRYP    -RSNFFKAE-------------VQMPRRPVNHGLLDCVTPHSTLLGYA--SGIPAYSNCH    63
tr|A0A1X0NYK9|A0A1X0NYK9_9TRYP    GLNRYYNT--------------HLPSKDPRFDPLKHCTTPFGTFLGVA--HGIPAYSNCH    75
tr|A0A1X0NRB4|A0A1X0NRB4_9TRYP    ------N----------------------TPPVPVNCVTPFGQLLGEN--SGVMVFSNCR    136
tr|Q4D408|Q4D408_TRYCC            ------D----------------------TSPRPEHCVTPFGHLLGES--RGVKAFSNCN    124
tr|A0A422N172|A0A422N172_9TRYP    ------D----------------------TPPRPEHCVTPFGELLGES--KGVFAFSNCN    117
tr|A0A422NKH5|A0A422NKH5_TRYRA    ------D----------------------TPPRPVHCVTPFGELLGES--NGVLAFSNCN    123
tr|A0A0S4ITA7|A0A0S4ITA7_BODSA    ---NCRGGSM----------------SLREQRSSSGCDTAFGSVIGVSAVGSVTAYSNCN    115
tr|A0A0N0DYP4|A0A0N0DYP4_LEPPY    -GSAQVEAAST---ALIPAEAVLPDGCRGDYCFEEGGTEPFGAVLGAH--DGVYAYSNCY    152
tr|A4HLZ6|A4HLZ6_LEIBR            -SFHLRNGAAPTTSSAETVHSQLPDGCLDHHCLKDGGSELFGAVLGAH--NGVFAYSNCN    165
tr|Q4Q3Q0|Q4Q3Q0_LEIMA            -SSHLRPGAALATTSVETPLSQLPESCRGHYCLQEGGKEPFGAVLGAH--NGVFAYSNCN    204
tr|A4I9C6|A4I9C6_LEIIN            -NSRLRPGAAPDTTSVETPLSQLPESCRGHYCLREGGKEPFGAVLGAH--DGVFAYSNCN    187
```

```
tr|A0A0N0DYQ7|A0A0N0DYQ7_LEPPY    SEVRYNGNC--T---------VDAGVTDTPA-LPEGCESPFGAILGAF--QNTFAHSNCN    220
tr|A4HLZ4|A4HLZ4_LEIBR            NVFFYRGNC--E---------NTNETTKGKTDPKKECVTPFGAILGVY--DGVFGYSNCN    148
tr|Q4Q3Q2|Q4Q3Q2_LEIMA            SSIRYGRNC--T---------GTNE----TVKPLGPCVTPLGTILGVF--NGVFGYSNCN    46
tr|A4I9C4|A4I9C4_LEIIN            SSIRYGGNC--T---------GTNGTSGGTVKPREPCVTPFSTILGVF--NGVFGYSNCN    183
tr|Q384E7|Q384E7_TRYB2            P---------------------------TFLYGETCVTPFGTIIGVH--NGVFAYSNCR    112
tr|Q586Y2|Q586Y2_TRYB2            NSKDTVST---------------NTGSLSVGCQQNCSAPFGNVLGIY--NGVPAMSNCN    92
tr|Q586Y1|Q586Y1_TRYB2            IR--YPE-G--------------------SGKVEKHCLNPVGAILGAH--EGVFSYSNCG    112
tr|F9W970|F9W970_TRYCI            KGDDYLRTGA--------------GIVDVPPGCRRGCATPFGSVLGEY--NGVKGMSNCD    97
tr|F9W971|F9W971_TRYCI            --I------------------------RYRGGSNTCSNSFGAITGTH--SGVFSYSNCG    108
tr|S9UGT3|S9UGT3_9TRYP            SGVHYQGSTATV---------DTNDSSSGTTLDQSSCHTQFGSIMGVH--QGVFAYSNCN    70
tr|A0A1X0NR96|A0A1X0NR96_9TRYP    ETINYNSS-HSL-----------------NNSCTVDCATTFGTILGIH--NGIFAYSNCN    106
tr|A0A1X0NR98|A0A1X0NR98_9TRYP    SGIHYHGDKWHE-----------------DSDGQEKCSTPFGAIIGVH--NDVFAYSNCD    126
tr|Q4D409|Q4D409_TRYCC            ETSVAKTGGTFL-----------------PEGCRKGCDTTFGAILGIV--NGVVSYSNCN    98
tr|Q4D410|Q4D410_TRYCC            ------------------------------FFGESACVSPYGAILGVA--GDVFAYSNCI    28
tr|Q4CKB1|Q4CKB1_TRYCC            GIHY-HGPGWHD-----------------ASSAKSACVSPYGAILGVA--GDVFAYSNCI    87
tr|Q4DEC6|Q4DEC6_TRYCC            GIHY-HGPGWHD-----------------ASSAKSACVSPYGAILGAA--GDVFAYSNCI    143
tr|A0A3R7N0H0|A0A3R7N0H0_9TRYP    SGVHYHGESWHN-----------------VNSNTPECVTPFGSIIGVT--NGVFAYSNCN    143
tr|A0A3R7N2W0|A0A3R7N2W0_9TRYP    DTVHEGNDTYRL-----------------SEGCSIGCDTTFGTILGVA--NGVFGFSNCN    101
tr|A0A3S5IRC8|A0A3S5IRC8_TRYRA    DTLVEGNGTSPF-----------------HEGCSIGCDTTFGSILGIS--NGVFGYSNCN    100


tr|A0A815F576|A0A815F576_9BILA    DSYISNEPNYL--------------------YNIYTGMRWQCVEYARRWLFIRKGCV--    103
tr|A0A814QMX3|A0A814QMX3_9BILA    SQYFRCGRHYV--------------------YGVFTGYQWQCVEFARRWLLMRKSCT--    66
tr|K2MZ59|K2MZ59_TRYCR            KNFFSERISLAPDCRHIEAAT-----STTVIGTYDTLGYQWLCLEFTRRYLLMTKGVW--    91
tr|A0A0G4IUR7|A0A0G4IUR7_PLABS    ESPEAYREALRAG-----DPK-----YHAFL-GDVFTGFQWQCVELARRYLVVNAGVA--    87
tr|A0A0N0DV74|A0A0N0DV74_LEPPY    STFKTTYVNYVNLMNPLDVGR---RGDPSET-TVIMLGVRYTAMDFYIRWMAWNRGLVP-    125
tr|A4HEJ1|A4HEJ1_LEIBR            TKFTSTYINYVNLMDPMDNGR---RGDPSEM-RVIMTAYRYSAFDYYMRWLVWNRGIMP-    156
tr|Q4Q9R0|Q4Q9R0_LEIMA            TKFSSTYINYVNLMDPMDNGR---RGDPSET-RIVMTAYRYTAFDYCMRWLVWNRGVMP-    125
tr|A4I1M7|A4I1M7_LEIIN            TKFSSTYINYVSLMDPMDNGR---RGDPSET-RIVMTAYRYTAFDYCMRWLVWNRGVMP-    125
tr|S9UNH8|S9UNH8_9TRYP            TRFNMERVSYCNLWNPLDVGR---TADPADGVPRVLVGEHFTANDYVNRWLIFNRGVLI-    127
tr|S9UZF8|S9UZF8_9TRYP            RRFKSDITNFVNHFNPYDMEQ---PYNPGEPVQRSMTSTAYDCLDFVSRYMVYYRGLIV-    126
tr|Q57ZC4|Q57ZC4_TRYB2            RHWLSETYAYTTIGYPHEVMK---KMPYGSEWKRAPTGLCWTADEFVARYLLHTRGIFV-    128
tr|A0A3R7LGJ5|A0A3R7LGJ5_9TRYP    RSYKAMRLVVTRFGRPDFVLD---YN--DGAAGNYFSGTPWLSTEYVARFLFHHRGVTH-    123
tr|Q4CUZ5|Q4CUZ5_TRYCC            SSYSAERLVITRFGYPELVAD---HN--DGSKGNYCSGSPWSSMEYTARILFHYKGIKY-    164
tr|Q4D7T1|Q4D7T1_TRYCC            RSYSAERLVITRFGHPELVAD---HN--DGSKGNYCSGSPWSSMEYTARILFHYKGIKY-    128
tr|A0A3R7KMG1|A0A3R7KMG1_TRYRA    RLWKTHVYAFVALSHPHDVRH---APFSLGTLHSRGTGTCWDSAEYIVRFFYLIRGMSF-    166
tr|Q4CYQ4|Q4CYQ4_TRYCC            RRWNSKSQAFVTLEHPHDVRQ---EPFLLGTLRSRSTGVRWVAAEFIARTYYLHRGMSF-    133
tr|Q4CUZ4|Q4CUZ4_TRYCC            RRWNSKSQAFVTLEHPHDVRQ---EPFLLGTLRSRSTGVRWVAAEFIARTYYLHRGMSF-    133
tr|Q4CUZ2|Q4CUZ2_TRYCC            --------------------------FLLGTLRSRSTGVRWVAAEFIARTYYLHRGMSF-    33
tr|Q4CYQ2|Q4CYQ2_TRYCC            -------------------------------------------------------MSF-    3
tr|Q4D7T2|Q4D7T2_TRYCC            RRWNSKSQAFVTLEHPHDVRQ---EPFLLGTLRSRSTGIRWVAAEFIARTYYLHRGMSF-    133
tr|Q4D7T0|Q4D7T0_TRYCC            -------------------------------------------------------MSF-    3
tr|A0A1X0NY88|A0A1X0NY88_9TRYP    RLWSSDVMAFASLAFPDDVLK---EPFPLGDRRQLSTGKCWVAAEYVSRFYYLMRGLVF-    119
tr|A0A1X0NYK9|A0A1X0NYK9_9TRYP    RSFRSNRLILTRFGSPDVFLE---HS--SDQEGSFISGSPWAATEYVARFLFHTKGITI-    129
tr|A0A1X0NRB4|A0A1X0NRB4_9TRYP    RDYQNMEEHYL-----------------FIKSLRVYSGVKWKSVEYARRFWLIAKHPG-V    178
tr|Q4D408|Q4D408_TRYCC            RDYRNVEEHFV-----------------LVRTSLIYSGLKWRSVEYARRFWLLAKRPA-V    166
tr|A0A422N172|A0A422N172_9TRYP    RDYRNVEEHFV-----------------LVKTSPVHSGLKWHSVEYARRFWLLAKWPA-V    159
tr|A0A422NKH5|A0A422NKH5_TRYRA    RDYRDVEEHFV-----------------LVKTSQVHSGLKWRSVEYARRFWLLAKQPA-V    165
tr|A0A0S4ITA7|A0A0S4ITA7_BODSA    SDYISNLSNFVNVTIPGGAGG-------SVVVPDVYTGMPWQCVEYARRYWATT--SPYT    166
tr|A0A0N0DYP4|A0A0N0DYP4_LEPPY    AHSCVSFVD-FEYPIPLPPGAHTPLDDPQATTRLMRTGMKWQCVEYARRYWMLRGTPAPA    211
tr|A4HLZ6|A4HLZ6_LEIBR            SNTCISHLE-HQMEIPLPPGSRTTLDAPHATTRPMKTGMKWQCVEYARRYWMLHGKPTPA    224
tr|Q4Q3Q0|Q4Q3Q0_LEIMA            SDTCISSLQ-YQMAIPLPPGARTALDAPHATTRLMTTGMKWQCVEYARRYWMLRGTPTPA    263
tr|A4I9C6|A4I9C6_LEIIN            SDTCTSLLK-YQMAIPLPPGARTALDAPHATTRLMTTGMKWQCVEFARRYWMLRGKPTPA    246
tr|A0A0N0DYQ7|A0A0N0DYQ7_LEPPY    GYVVSTVDSYMNFTVPVMDAA---TGMLTMSSKEFYTGLAWQCVEYARRYWMINGRPKPA    277
tr|A4HLZ4|A4HLZ4_LEIBR            DDYESTKYKYVNLMVPVLNNK---TGKVEYTSKSFFTGLEWQCVEFARRFWMLSGKPEGA    205
tr|Q4Q3Q2|Q4Q3Q2_LEIMA            DSYVSTELRYINLTVPELNNE---TGQLTYISKQFYTGLAWQCVEYARRYWMQRGTPQPA    103
tr|A4I9C4|A4I9C4_LEIIN            DSYVSTELGYINLTVLELNNE---TGQVTYISKQFYTGLAWQCVEYARRYWMQSGTPQPA    240
tr|Q384E7|Q384E7_TRYB2            RDHISTKKN-S-----------------IKGEGSMETGMEWQCFEYVKRYWIMRGVPQSV    154
tr|Q586Y2|Q586Y2_TRYB2            SDSCTAELW-NTVKVEDIRIP---AGRVDPHAVPPIYGMQWQCVEYARRYWMLRGTPQPA    148
tr|Q586Y1|Q586Y1_TRYB2            ATENTTTYN-N----------------VTVAGTSYQSGLKWQCVEYARRYWMLRGTPQPA    155
tr|F9W970|F9W970_TRYCI            SDTCIADHW-HTIDVSELRDA------IRMETFPSSYGVKWQCVEYARRYWVLRGTPKPA    150
tr|F9W971|F9W971_TRYCI            MDVNAIEHH-N----------------VTVEGRTQTTGVKWQCVEYARRYWVLRGTPKPA    151
tr|S9UGT3|S9UGT3_9TRYP            SDYISEDDS-VI---------------KVSGAKNLYAGLRWQCVEYARRYWMLVGTPVPA    114
tr|A0A1X0NR96|A0A1X0NR96_9TRYP    STTCISNEW-HELEDPQISIA---FKGNT--TIGNSTGMKWQCVEYARRYWMLRGSPVPA    160
tr|A0A1X0NR98|A0A1X0NR98_9TRYP    EDY-ISKDY-NTLAY----------------TALSNTGLKWQCVEYARRYWMLRGSPVPA    168
tr|Q4D409|Q4D409_TRYCC            SSTCTSNLW-HPMSSLLAHAD---YNNENRWNNTNTSGLEWQCVEFARRYWMLRGTPVRA    154
tr|Q4D410|Q4D410_TRYCC            RNYDSK-------------------SRNNVSSQNKESGLEWDCMEFARRYWMLRGTPVRA    69
tr|Q4CKB1|Q4CKB1_TRYCC            RNYDSK-------------------SRNNVSSQNKESGLEWDCMEFARRYWMLRGTPVRA    128
tr|Q4DEC6|Q4DEC6_TRYCC            RNYDSK-------------------SRNNVSSQNKESGLKWDCMEFARRYWMLRGTPVRA    184
tr|A0A3R7N0H0|A0A3R7N0H0_9TRYP    EDYSSKENS-----TLL-------------GKDKLFTGLGWQCVEFARRYWMLRGTPVPA    185
tr|A0A3R7N2W0|A0A3R7N2W0_9TRYP    ATTCISNIW-HPMHALMGPAG---ISSSSGIGPGNMSGIEWQCVEFARRYWMLRGTPVPA    157
tr|A0A3S5IRC8|A0A3S5IRC8_TRYRA    ATTCISNKW-HPIHTFMTSGG---FTGRNILAPENTSGIKWQCVEFARRYWMLRGSPVPA    156


tr|A0A815F576|A0A815F576_9BILA    -FDSVDAANDMWSQIFKVQRVVDG----KCFSLKQYQNG------------------ST    139
tr|A0A814QMX3|A0A814QMX3_9BILA    -FKDIPCASNIWTDLSYIERITDG----QHFSLHSIPNG------------------SS    102
tr|K2MZ59|K2MZ59_TRYCR            -LASIPTAEDIWEMDEVFISVPTG----TPVPMEREKHG------------------ETT    128
tr|A0A0G4IUR7|A0A0G4IUR7_PLABS    -FDDVVNAYQIWDLDGVRR-VADG----ERVAWRRFANG-------------------SR    122
tr|A0A0N0DV74|A0A0N0DV74_LEPPY    -R-LVEDTSQFWKTNSFYN-PA--------KTAQDWEAVYITNYAEATD-----VEERRF    169
tr|A4HEJ1|A4HEJ1_LEIBR            -R-LVENTNQLWKRVDYFN-PA--------KPEQDWSAEYITNYEEVTS-----LEERKF    200
tr|Q4Q9R0|Q4Q9R0_LEIMA            -R-LVENTNQLWKTVDYFN-PA--------RPEQGWSAEYITNYEEVTD-----VEERKF    169
tr|A4I1M7|A4I1M7_LEIIN            -R-LVENTNQLWKTVDYFN-PA--------KPEQGWSAEYITNYEEVTG-----MEERKF    169
tr|S9UNH8|S9UNH8_9TRYP            -S-LFPYYSRYVSEASFAY-PT--------PTPSHRMTVRLHNKQRALD-----IEDRKL    171
tr|S9UZF8|S9UZF8_9TRYP            -S-MHHIFYEQWEYGSFIN-PAR--------PEVRRETEKYANYKVAST-----LDERKR    170
tr|Q57ZC4|Q57ZC4_TRYB2            -YFGGSRHDLYWENLKFFG-SS--------GMHRLYERINFENKEVTT-----TKRRKR    173
tr|A0A3R7LGJ5|A0A3R7LGJ5_9TRYP    -L-PADTPQDVWTSKYFYN-PLEG-E--VGGKRRRYEPVRIANYDAATT-----AKLRKR    172
tr|Q4CUZ5|Q4CUZ5_TRYCC            -F-EMPTPQEVWSTPYFFN-PLEA-A--ERDKERRYEPVRVANYEEATT-----AKERKR    213
```

```
tr|Q4D7T1|Q4D7T1_TRYCC              -F-ETPTPQEVWSTPYFFN-PLEA-A--ERDKERRYEPVRVANYEEAAT-----AKERKR    177
tr|A0A3R7KMG1|A0A3R7KMG1_TRYRA      -IYSTRDVVSHWSTLEFHG-GI--------ATDRSYEAIRLLNAAEATT-----VKLRKR    211
tr|Q4CYQ4|Q4CYQ4_TRYCC              -IYGAPDAPEYWLALEFHG-PM--------VREREYETLELLNSAEATT-----AKERKR    178
tr|Q4CUZ4|Q4CUZ4_TRYCC              -IYGAPDAPEYWLALEFHG-PM--------VREREYETLELLNSAEATT-----AKERKR    178
tr|Q4CUZ2|Q4CUZ2_TRYCC              -IYGAPDAPEYWLALEFHG-PM--------VREREYETLELLNSAEATT-----AKERKR    78
tr|Q4CYQ2|Q4CYQ2_TRYCC              -IYGAPDAPEYWLALEFHG-PM--------VREREYETLELLNSAEATT-----AKERKR    48
tr|Q4D7T2|Q4D7T2_TRYCC              -IYGAPDAPEYWSALEFHG-PM--------VREREYETLELLNSAEATT-----AKERKH    178
tr|Q4D7T0|Q4D7T0_TRYCC              -IYGAPDAPEYWSALEFHG-PM--------VREREYETLELLNSAEATT-----AKERKH    48
tr|A0A1X0NY88|A0A1X0NY88_9TRYP      -YYDSAEVRQFWSTIEFYS-TE--------GDKRKYEVIELPNYKEVKT-----AKERKR    164
tr|A0A1X0NYK9|A0A1X0NYK9_9TRYP      -H-DPGTTQEIWSIRYFYN-PLST-K--HSDNLRKYEPIQLENFMEVKT-----AKERKR    178
tr|A0A1X0NRB4|A0A1X0NRB4_9TRYP      AFEYVNSPVDIWTYVETAK-DVNG----KDLNLRKYPNSLTGKSISNISKDM--KKQWLA    231
tr|Q4D408|Q4D408_TRYCC              QFMSVQNAEDIWTRVVYAS-YITG----ERVKLFKYENMLACDQTPTKNTGM-KKSLWKH    220
tr|A0A422N172|A0A422N172_9TRYP      QFMSVRNAEDIWTRVNYAS-YTNG----KRVKLFKYENMLSCDRAPALNATM-KKHAWNS    213
tr|A0A422NKH5|A0A422NKH5_TRYRA      QFMSVTCAEDIWTRVDYAY-YING----KRVKLLKYENMMACDQAPLSKTTM-KKGQWKH    219
tr|A0A0S4ITA7|A0A0S4ITA7_BODSA      LFGSVDGASDIWTSLHHGT-FIESSDVVVQFDLQKFENGGV----------------NAT    209
tr|A0A0N0DYP4|A0A0N0DYP4_LEPPY      VFGAVEGAADMWTDLSFVT-LLDN-V--TTAPLWKYTNG--------------APVGSGG    253
tr|A4HLZ6|A4HLZ6_LEIBR              LFGTVVGAADIWHSIHSVT-FLDN-K--TTAPLLKFQNG--------------AKLGYGG    266
tr|Q4Q3Q0|Q4Q3Q0_LEIMA              FFGAVKGAADIWDSLTHVT-FLDN-A--TTAPLLKFKNG--------------ARLGYGG    305
tr|A4I9C6|A4I9C6_LEIIN              FFGPVVGAADIWDTLTHVT-FLDN-A--TTAPLLKFKNG--------------ARLGYGG    288
tr|A0A0N0DYQ7|A0A0N0DYQ7_LEPPY      YFGSVIGAADIWN-LTEVH-LLENTS--RTLPLRKYKSGDR-------------VVQDGL    320
tr|A4HLZ4|A4HLZ4_LEIBR              YFDSVVGAADIWN-LSFVR-LVSNTS--ATLPLQKYSNGGR-------------L-SDGL    247
tr|Q4Q3Q2|Q4Q3Q2_LEIMA              YFDSVLGAADVWN-LTFVR-LLSNAS--ITLPLRRYWNGDR-------------V-TDNH    145
tr|A4I9C4|A4I9C4_LEIIN              YFESVPGAADIWN-LTFVR-LLSNTS--MKLPLHRFRNGDP-------------V-TSNL    282
tr|Q384E7|Q384E7_TRYB2              ILPTARKSSELWS-FTQAT-FKNG-S--KV-QLERHD--------------------NGG    188
tr|Q586Y2|Q586Y2_TRYB2              TFGSVDGAADIWD-LKDVQ-LLNG-Q--KRKPLLKYHNG---------------NATSAN    188
tr|Q586Y1|Q586Y1_TRYB2              TFGSVDGAADIWD-LKDVQ-LLNG-Q--KRKPLLKYHNG---------------NATSAN    195
tr|F9W970|F9W970_TRYCI              AFGSVEGAADIWD-LDNVA-LLDG-T--T-RPLLKFQNE---------------NATAAG    189
tr|F9W971|F9W971_TRYCI              AFGSVEGAADIWD-LDNVA-LLDG-T--T-RPLLKFQNE---------------NATAAG    190
tr|S9UGT3|S9UGT3_9TRYP              YFGSINGAADIWA-LTIVY-TVSD-A--TTLPLHRFESMTKKDYESAQQTPSSAMGNITN    169
tr|A0A1X0NR96|A0A1X0NR96_9TRYP      TFSAVEGAADIWE-LQSVQ-LVNG-A--KT-PLLKYSNG--------------LSISAGG    200
tr|A0A1X0NR98|A0A1X0NR98_9TRYP      TFSSVEGAADIWE-LQSVQ-LVNG-A--KT-PLLKYSNG--------------LSISAGG    208
tr|Q4D409|Q4D409_TRYCC              TFDSVVGAADIWA-LNSVR-LLDG-S--KT-PLLKYPNG--------------LPRRDGG    194
tr|Q4D410|Q4D410_TRYCC              TFDSVVGAADIWA-LNSVR-LLDG-S--KT-PLLKYPNG--------------LPRRDGG    109
tr|Q4CKB1|Q4CKB1_TRYCC              TFDSVVGAADIWA-LNSVR-LLDG-S--KT-PLLKYPNG--------------LPRRDGG    168
tr|Q4DEC6|Q4DEC6_TRYCC              TFDSVVGAADIWA-LNFVR-LLDG-S--KT-PLLKYPNG--------------LPRRDGG    224
tr|A0A3R7N0H0|A0A3R7N0H0_9TRYP      SFEKVAGAADIWS-LTSVQ-LLDG-S--TK-PLLKYANN--------------VSARDGG    225
tr|A0A3R7N2W0|A0A3R7N2W0_9TRYP      SFEKVAGAADIWS-LTSVQ-LLDG-S--TT-PLLKYANN--------------VSARAGG    197
tr|A0A3S5IRC8|A0A3S5IRC8_TRYRA      SFATVEGAADIWA-LTSVQ-LLNG-S--TA-PLLKYANG--------------VSVRAGG    196


tr|A0A815F576|A0A815F576_9BILA      SPPKNESLLIYSLGQD-----M-PFGHVAVIVDVLK------------------------    169
tr|A0A814QMX3|A0A814QMX3_9BILA      KSPKKDSLLIYRRSKK-----M-TYGHVAIITDVTS------------------------    132
tr|K2MZ59|K2MZ59_TRYCR              NMPSVGDLLVWSRTED-----F-PYGHLAVVTHVSD------------------------    158
tr|A0A0G4IUR7|A0A0G4IUR7_PLABS      VRPEIGSLLIWRPAGF-----YERTGHVAVVVAVGD------------------------    153
tr|A0A0N0DV74|A0A0N0DV74_LEPPY      NAPRKADAILYNQDSK-----ILPDGHIAVIVKVEGDVEA--AGGTEKF---NELKKLRL    219
tr|A4HEJ1|A4HEJ1_LEIBR              NAPRRADAVIYRMDEK-----TLPAGHIAVVVKVEDDIEA--AGGLEKL---KDLKKMRL    250
tr|Q4Q9R0|Q4Q9R0_LEIMA              NAPRRGDAIVYRMDKN-----TIPAGHMAVVVKVEDDVEA--AGGPEKL---NELKKMRL    219
tr|A4I1M7|A4I1M7_LEIIN              NAPRRADAIVYRMDKN-----TIPAGHIAVVVKVEDDVEA--AGGPEKL---KELKKMRL    219
tr|S9UNH8|S9UNH8_9TRYP              NAPRRGDVVVFDAMYTEGQPHYLPSGHIAVVVKVENNLEHPDMQDKEKK---NKLIRERL    228
tr|S9UZF8|S9UZF8_9TRYP              FAPRVGDVIVYNADPA----MDLSAGHLAVVVRVEADMDDPVAKDKERL---RQLREQRL    223
tr|Q57ZC4|Q57ZC4_TRYB2              HTPLVGDVVVWDSDYK----AYFPRGHVAVVVKVEDDVSA--AGGEAAL---RELKKERR    224
tr|A0A3R7LGJ5|A0A3R7LGJ5_9TRYP      LAPRFADIVVWPAQSE----EGLPGGHVAVVVQVEDDVAA--AGGADRL---RALRRERL    223
tr|Q4CUZ5|Q4CUZ5_TRYCC              LAPRVFDIVVWPAQME----HELPEGHIAVVVQVEDDVEA--AGGEDRL---RELRKLRL    264
tr|Q4D7T1|Q4D7T1_TRYCC              LAPRVFDIVVWPAQME----HELPEGHIAVVVQVEDDVEA--AGGEDRL---RELRKLRL    228
tr|A0A3R7KMG1|A0A3R7KMG1_TRYRA      LAPKVADIVVWAAQRE----NDLPEGHLAVVVQVEHDVEA--AGGEDSL---RELQKERL    262
tr|Q4CYQ4|Q4CYQ4_TRYCC              LAPRVFDVVVWSPMEE----RDLPEGHIAVVVQVEDDVEA--AGGEDRL---RELRKLRL    229
tr|Q4CUZ4|Q4CUZ4_TRYCC              LAPRVFDVVVWSPMEE----RDLPEGHIAVVVQVEDDVEA--AGGEDRL---RELRKLRL    229
tr|Q4CUZ2|Q4CUZ2_TRYCC              LAPRVFDVVVWSPMEE----RDLPEGHIAVVVQVEDDVEA--AGGEDRL---RELRKLRL    129
tr|Q4CYQ2|Q4CYQ2_TRYCC              LAPRVFDVVVWSPMEE----RDLPEGHIAVVVQVEDDVEA--AGGEDRL---RELRKLRL    99
tr|Q4D7T2|Q4D7T2_TRYCC              LAPRVLDVVVWSPMEE----RDLPEGHIAVVVRVEDDVEA--AGGEDRL---RELRKLRL    229
tr|Q4D7T0|Q4D7T0_TRYCC              LAPRVLDVVVWSPMEE----RDLPEGHIAVVVQVEDDVEA--AGGEDRL---RELRKLRL    99
tr|A0A1X0NY88|A0A1X0NY88_9TRYP      LAPKFADVVVWASHPE----RELDEGHAAVVVHVEDDVEA--AGGEAQL---REMQKEHT    215
tr|A0A1X0NYK9|A0A1X0NYK9_9TRYP      LAPKFADVVVWASHPE----RELDDGHAAVVVHVEDDVEA--AGGEAQL---REMQKEHT    229
tr|A0A1X0NRB4|A0A1X0NRB4_9TRYP      SKPQVGDLLIYDHSK------KLPEGHVAVIVNVVPVSNI--NNG----------NDIFV    273
tr|Q4D408|Q4D408_TRYCC              YKPQVGDLLVYANDS------SIPGGHVAVVVGIVSMPSN--SGA----------NNSSM    262
tr|A0A422N172|A0A422N172_9TRYP      YRPQIGDLLVYANES------RLPGGHVAVVVGIASLPLQ--AAA----------NASRI    255
tr|A0A422NKH5|A0A422NKH5_TRYRA      YGPQIGDLLVYANDS------RLPGGHVAVVVGRVSQPPH--AVS----------NTSWI    261
tr|A0A0S4ITA7|A0A0S4ITA7_BODSA      SPPQVGDLFIYPIQP-----GGFPFGHVTVVLNDQLPPTL--NNG------------GND    250
tr|A0A0N0DYP4|A0A0N0DYP4_LEPPY      SAPRVGDLLIYPRDTD----GKFPFGHVAVIVGVELPGER--PQATTNSDE-----AHAA    302
tr|A4HLZ6|A4HLZ6_LEIBR              SAPRVGDLLIYPRDTL----GNFPFGHVAVVVGVEMPANA--EANDSYTDAEMAAAQLRQ    320
tr|Q4Q3Q0|Q4Q3Q0_LEIMA              SAPRVGDLLIYPRDTE----NVFPYGHVAVVVKVEMPTKA--EADDSYMDAGAASPKPRQ    359
tr|A4I9C6|A4I9C6_LEIIN              SAPRVGDLLIYPRDAE----GFFSYGHVAVVVRVEMTTKA--EADDSYMDAAVTSSKPRQ    342
tr|A0A0N0DYQ7|A0A0N0DYQ7_LEPPY      QPPQAGDIIIYPVQS-----GGFPVGHVAVVTKVEMGE----------------------    353
tr|A4HLZ4|A4HLZ4_LEIBR              QAPAPGDIIIYPVQG-----GGFPYGHVAVITKVDMAV----------------------    280
tr|Q4Q3Q2|Q4Q3Q2_LEIMA              QIPAIGDIIIYPVQD-----GGFPYGHVAVIANVELST----------------------    178
tr|A4I9C4|A4I9C4_LEIIN              QVPAVGDIIIYPVQD-----GGFPYGHVAVIASVEIST----------------------    315
tr|Q384E7|Q384E7_TRYB2              PQPLVGDLLVYREQP-----ALLPVGHVAVIVRVGK------------------------    219
tr|Q586Y2|Q586Y2_TRYB2              SKPRVGDLLIYPRQP-----NGFPYGHVAVVAGVTG------------------------    219
tr|Q586Y1|Q586Y1_TRYB2              SKPRVGDLLIYPRQP-----NGFPCGHVAVVAGVTG------------------------    226
tr|F9W970|F9W970_TRYCI              SRPRAGDLLIYPRQP-----NGFPYGHVAVVAGVEE------------------------    220
tr|F9W971|F9W971_TRYCI              SRPRAGDLLIYPRQP-----NGFPYGHVAVVAGVEE------------------------    221
tr|S9UGT3|S9UGT3_9TRYP              TVPRVGDILIYKREL-----PSFPYGHVAVVVEFVAGATT--AG------------AAAA    210
tr|A0A1X0NR96|A0A1X0NR96_9TRYP      SAPRVGDLLIYPRQK-----GGFVYGHVAVIVDVLS------------------------    231
tr|A0A1X0NR98|A0A1X0NR98_9TRYP      SAPRVGDLLIYPRQK-----GGFVYGHVAVIVDVLS------------------------    239
tr|Q4D409|Q4D409_TRYCC              SAPRAGDLLIYPRQR-----NDFPFGHVAVVVGVTK------------------------    225
tr|Q4D410|Q4D410_TRYCC              SAPRAGDLLIYPRQR-----NDFPFGHVAVVVGVTK------------------------    140
tr|Q4CKB1|Q4CKB1_TRYCC              SAPRAGDLLIYPRQR-----NDFPFGHVAVVVGVTK------------------------    199
```

```
tr|Q4DEC6|Q4DEC6_TRYCC              SAPRAGDLLIYPRQR-----EDFPFGHVAVVVGVTK------------------------   255
tr|A0A3R7N0H0|A0A3R7N0H0_9TRYP      SAPRVGDLLIYPRQE-----GGFPFGHVAVVVAVKS------------------------   256
tr|A0A3R7N2W0|A0A3R7N2W0_9TRYP      SAPRVGDLLIYPRQE-----GGFPFGHVAVVVAVKS------------------------   228
tr|A0A3S5IRC8|A0A3S5IRC8_TRYRA      SAPRIGDLLVYPKQG-----DGLPFGHVAVVVSVKS------------------------   227
                                    *   . .::              ** :::

tr|A0A815F576|A0A815F576_9BILA      --DSIRVAEQNYHAYYWS--G----------------NYSRQIPYVVKNGN-------YY   202
tr|A0A814QMX3|A0A814QMX3_9BILA      --DYVYIAEQNNLYHYWP--G----------------DYARRERLRFDNGN-------YY   165
tr|K2MZ59|K2MZ59_TRYCR              --KRVWVAEQNYEFKRWQPGK----------------NYSRRFDCEKREDG-------VT   193
tr|A0A0G4IUR7|A0A0G4IUR7_PLABS      --AFVDIVEQNVDDTVWEPGQ----------------TYSRRLKARSDAGS-------FT   188
tr|A0A0N0DV74|A0A0N0DV74_LEPPY      HPRRVYVAEQNFKNEDWG-GK----------------NYSRILTFQWRQVKT-GTTYEGF   261
tr|A4HEJ1|A4HEJ1_LEIBR              HPRRVYVAEQNCQNQPWG-EH----------------NYSRVLQFKWRAVSE-M-AHEGY   291
tr|Q4Q9R0|Q4Q9R0_LEIMA              HPRRVYVAEQNWKNQPWG-GH----------------NYSRVLQFKWRAVSE-K-AHEGG   260
tr|A4I1M7|A4I1M7_LEIIN              HPRRVYVAEQNWKNQPWG-GH----------------NYSRVLQFKWRAVAE-K-AYEGC   260
tr|S9UNH8|S9UNH8_9TRYP              QPRRVYIAEQNLYNRHWE-GK----------------NYSRVMDFYWEPTAN-EKEFRAI   270
tr|S9UZF8|S9UZF8_9TRYP              HPRKVYIAEQNFGNADWG-GK----------------NYTRICRFVWEPVRGATGLYESI   266
tr|Q57ZC4|Q57ZC4_TRYB2              QPSLVYIAEQNFDNKNWE-GR----------------NFSRVLKFTWMRGD------RAS   261
tr|A0A3R7LGJ5|A0A3R7LGJ5_9TRYP      QPQLVYIAEQNFDNAPWA-GR----------------NYSRVLRFYWENGK------EAV   260
tr|Q4CUZ5|Q4CUZ5_TRYCC              QPRLLYIAEQNFDNTHWG-GK----------------NYSRVLRFQWRNGR------EAV   301
tr|Q4D7T1|Q4D7T1_TRYCC              QPRLLYIAEQNFDNTHWG-CK----------------NYSRVLRFQWRNGR------EAV   265
tr|A0A3R7KMG1|A0A3R7KMG1_TRYRA      QPQLVYIAEQNFDNVLWD-GK----------------NYSRVLRFYWKNGK------EAM   299
tr|Q4CYQ4|Q4CYQ4_TRYCC              QPRLLYIAEQNFDNTHWG-GK----------------NYSRVLRFQWRNGR------EAV   266
tr|Q4CUZ4|Q4CUZ4_TRYCC              QPRLLYIAEQNFDNTHWG-GK----------------NYSRVLRFQWRNGR------EAV   266
tr|Q4CUZ2|Q4CUZ2_TRYCC              QPRLLYIAEQNFDNTHWG-GK----------------NYSRVLRFQWRNGR------EAV   166
tr|Q4CYQ2|Q4CYQ2_TRYCC              QPRLLYIAEQNFDNTHWG-GK----------------NYSRVLRFQWRNGR------EAV   136
tr|Q4D7T2|Q4D7T2_TRYCC              QPRLLYIAEQNFDNTHWG-CK----------------NYSRVLRFQWRNGR------EAV   266
tr|Q4D7T0|Q4D7T0_TRYCC              QPRLLYIAEQNFDNTHWG-CK----------------NYSRVLRFQWRNGR------EAV   136
tr|A0A1X0NY88|A0A1X0NY88_9TRYP      QPQLVYIAEQNFDNKDWE-GK----------------NYSRVLRFSWKNGK------EML   252
tr|A0A1X0NYK9|A0A1X0NYK9_9TRYP      QPQLVYIAEQNFDNKDWE-GK----------------NYSRVLRFSWKNGK------EML   266
tr|A0A1X0NRB4|A0A1X0NRB4_9TRYP      KYYNVYLAEQNWDNRPWV-EEEDLVTSSD-NNEPRALYYSRMVLLKEDMTT-----HRMT   326
tr|Q4D408|Q4D408_TRYCC              TQYSMLLAEQNWNNEPWP-HRSNLEGKDTRNDTRLLGYYSREVSLQEVESA-----QMVC   316
tr|A0A422N172|A0A422N172_9TRYP      RQYSLLLAEQNWDNARWL-CNDTLEGGT-STVTALPCQYSRKVLLQQVDSA-----RRMC   308
tr|A0A422NKH5|A0A422NKH5_TRYRA      RQCSLLLAEQNWDNARWL-YNETLRGRDTPTSTAVPLHYSRKVLLQEVDST-----RKMC   315
tr|A0A0S4ITA7|A0A0S4ITA7_BODSA      VTSEVHVGEQNWDSFQWQ-HA--------------AEGYSRKLQLTYHQLS-----RRWS   290
tr|A0A0N0DYP4|A0A0N0DYP4_LEPPY      REGRAYIAEQNWHSSPWP-EP--------------YHNYSRWLPLEVTATPQ-GTSVQYT   346
tr|A4HLZ6|A4HLZ6_LEIBR              RRGLVYIAEQNWDSVPWP-KP--------------YHNYSRSLPLVVLESAE-GQPLQYT   364
tr|Q4Q3Q0|Q4Q3Q0_LEIMA              RHSHVYVAEQNWDSVTWP-NP--------------YHNYSRSLPLVVLESAE-GRPLQYT   403
tr|A4I9C6|A4I9C6_LEIIN              RHGLVYLAEQNWDSATWP-NP--------------YHNYSRSLPLVVLESAE-GLPLQYT   386
tr|A0A0N0DYQ7|A0A0N0DYQ7_LEPPY      -QGFIYVAEQNWESTQWS-GP--------------FYNYSRKIPLRYDPLT-----TAII   392
tr|A4HLZ4|A4HLZ4_LEIBR              -NGAIYVAEQNWGSAMWV-DP--------------HHNYSRRIPLMYDMTT-----SSVT   319
tr|Q4Q3Q2|Q4Q3Q2_LEIMA              -HGAIYVAEQNWANAVWS-SP--------------HHNYTRRIPMFYDMLT-----STIT   217
tr|A4I9C4|A4I9C4_LEIIN              -HGAIYVAEQNWANAVWP-SA--------------YHNYSRRIPLYYEMLT-----STIT   354
tr|Q384E7|Q384E7_TRYB2              --THVWVAEQNWFNKQWH-PP--------------FHNFSRTIKMHHNAES-----QTYE   257
tr|Q586Y2|Q586Y2_TRYB2              --DRMFVAEQNWENTAWP-GP--------------YHNYSRVLNLSCNPNG-----TACT   257
tr|Q586Y1|Q586Y1_TRYB2              --DRMFVAEQNWENAAWP-GP--------------YHNYSRVLNLSCNPNG-----TACT   264
tr|F9W970|F9W970_TRYCI              --RRLFVAEQNWDNQQWP-GP--------------YHNYSRELKLTCDASS-----TRCS   258
tr|F9W971|F9W971_TRYCI              --RRLFVAEQNWDNQQWP-GP--------------YHNYSRELKLTCDASS-----TRCS   259
tr|S9UGT3|S9UGT3_9TRYP              SAPYVLVGEQNWDNEMWP-SP--------------YHNYSRRVNMVYDASK-----EVYT   250
tr|A0A1X0NR96|A0A1X0NR96_9TRYP      --AAVLVAEQNWDNTVWP-GP--------------FHNYSRSIPMQYDANS-----KAYT   269
tr|A0A1X0NR98|A0A1X0NR98_9TRYP      --AAVLVAEQNWDNTVWP-GP--------------FHNYSRSIPMQYDANS-----KAYT   277
tr|Q4D409|Q4D409_TRYCC              --NSVLVAEQNWDNKMWP-GP--------------YHNHSREIRMLYSPIH-----DAYN   263
tr|Q4D410|Q4D410_TRYCC              --NSVLVAEQNWDNKMWP-GP--------------YHNHSREIRMLYSPIH-----DAYN   178
tr|Q4CKB1|Q4CKB1_TRYCC              --NSVLVAEQNWDNKMWP-GP--------------YHNHSREIRMLYSPIH-----DAYN   237
tr|Q4DEC6|Q4DEC6_TRYCC              --NSVLVAEQNWDNKMWP-GP--------------YHNHSREIRMLYSPIH-----DAYN   293
tr|A0A3R7N0H0|A0A3R7N0H0_9TRYP      --ESLLVAEQNWDNKVWP-AP--------------HHNYSREIPMHHNTKE-----DIYE   294
tr|A0A3R7N2W0|A0A3R7N2W0_9TRYP      --ESLLVAEQNWDNKVWP-AP--------------HHNYSREIPMHHNTKE-----DIYE   266
tr|A0A3S5IRC8|A0A3S5IRC8_TRYRA      --ESLLVAEQNWDNNVWP-AP--------------HHDYSREIPMHHNVTE-----DTYK   265
                                              : ***    *                     .:*

tr|A0A815F576|A0A815F576_9BILA      IMD--SYKIYGWMSVEDNNQN---YPLNQSTINKIMEKNIS--FPNFICSKSVTFNQLKP   255
tr|A0A814QMX3|A0A814QMX3_9BILA      IDD--EDPIYGWMEIENNDEL---KPFDESNINNILQKYLE--FKSMDGV----------   208
tr|K2MZ59|K2MZ59_TRYCR              VFV-GELHLLGWVSIK--------APSYDFSLGDLPDKYRHILGPGHIVRRHLEREFLLP   244
tr|A0A0G4IUR7|A0A0G4IUR7_PLABS      VVDDYDGDVIGWIIMSIVTTE--RAPAYDDESDMDRDEL---------------------   225
tr|A0A0N0DV74|A0A0N0DV74_LEPPY      LTDPDSLSIIGFVRVGKPLPLRELSDPYDDALRDD-GGDL--------------------   300
tr|A4HEJ1|A4HEJ1_LEIBR              YVDPDGLDIIGCMRVGKSMPLRAVPDPYQETLDMENDGDL--------------------   331
tr|Q4Q9R0|Q4Q9R0_LEIMA              YVDPDELDIIGVVRVGKAMPLRAAPDPYEEALNMDNDGDL--------------------   300
tr|A4I1M7|A4I1M7_LEIIN              YVDPDGLGIIGVVRVGKAMPLRAAPDPYQEALDMDNDGDL--------------------   300
tr|S9UNH8|S9UNH8_9TRYP              LQDNDGLTVLGRIRAGRRVPTSENKPSYQLGLQEAAKEVKEKYGEEVEVVTPTDQE----   326
tr|S9UZF8|S9UZF8_9TRYP              LKDPDSLSIIGRLRIGRELSLTEEEPSAEEKGLQAMDEDKMKHKQGLK------------   314
tr|Q57ZC4|Q57ZC4_TRYB2              LEDPDGPPLMGHVRVGKLLED--ASFFGDL------------------------------   289
tr|A0A3R7LGJ5|A0A3R7LGJ5_9TRYP      LQDPSGARVLGLVRKGKLALNLEDDDAGDL------------------------------   290
tr|Q4CUZ5|Q4CUZ5_TRYCC              LQDPLGLKVLGLVRIGKRTVIFEDEDGGDL------------------------------   331
tr|Q4D7T1|Q4D7T1_TRYCC              LQDPLGLKVLGLVRIGKRTVIFEDEDGGDL------------------------------   295
tr|A0A3R7KMG1|A0A3R7KMG1_TRYRA      LHDSSGPQELGFVRKGQACRK--PQGRGCS------------------------------   327
tr|Q4CYQ4|Q4CYQ4_TRYCC              LQDPLGLPIIGRVRPGRPKQI---------------------------------------   287
tr|Q4CUZ4|Q4CUZ4_TRYCC              LQDPLGLPIIGRVRPGRPKQN--TDVEDDL------------------------------   294
tr|Q4CUZ2|Q4CUZ2_TRYCC              LQDPLGLPIIGRVRPGRPKQN--TDVEDDL------------------------------   194
tr|Q4CYQ2|Q4CYQ2_TRYCC              LQDPLGLPIIGRVRPGRPKQN--TDVEDDL------------------------------   164
tr|Q4D7T2|Q4D7T2_TRYCC              LQDPLELPIIGRVRPGKPKQN--TDVEDDL------------------------------   294
tr|Q4D7T0|Q4D7T0_TRYCC              LQDPLELPIIGRVRPGKPKQN--TDVEDDL------------------------------   164
tr|A0A1X0NY88|A0A1X0NY88_9TRYP      LEDSDDYNIIGRVRPGKVKRD--DMDEGDL------------------------------   280
tr|A0A1X0NYK9|A0A1X0NYK9_9TRYP      LEDSDDYNIIGRVRPGNLKRD--DMDEGDL------------------------------   294
tr|A0A1X0NRB4|A0A1X0NRB4_9TRYP      IEDAGG-MVLGWVRV---------------------------------------------   340
tr|Q4D408|Q4D408_TRYCC              VKDPWG-TVLGWVRA---------------------------------------------   330
tr|A0A422N172|A0A422N172_9TRYP      VTDPGG-TVLGWVRV---------------------------------------------   322
tr|A0A422NKH5|A0A422NKH5_TRYRA      VKDPRG-TVLGWVRV---------------------------------------------   329
tr|A0A0S4ITA7|A0A0S4ITA7_BODSA      VYDPQG-KIAGWVRRPSQRS----------------------------------------   309
```

```
tr|A0A0N0DYP4|A0A0N0DYP4_LEPPY      LHDKYH-AILGWMRYGEP------------------------------------------    363
tr|A4HLZ6|A4HLZ6_LEIBR              IEDSFH-GVQGWARYDDVP-----------------------------------------    382
tr|Q4Q3Q0|Q4Q3Q0_LEIMA              IEDSLH-GIQGWVRYDDEP-----------------------------------------    421
tr|A4I9C6|A4I9C6_LEIIN              IEDSLH-GIQGWVRYDDDP-----------------------------------------    404
tr|A0A0N0DYQ7|A0A0N0DYQ7_LEPPY      LNDPDG-KIIGWMRYG--------------------------------------------    407
tr|A4HLZ4|A4HLZ4_LEIBR              LNDPNG-IIIGWMRYG--------------------------------------------    334
tr|Q4Q3Q2|Q4Q3Q2_LEIMA              LDDSEH-QIIGWMRYG--------------------------------------------    232
tr|A4I9C4|A4I9C4_LEIIN              LDDPKG-KIIGWMRYG--------------------------------------------    369
tr|Q384E7|Q384E7_TRYB2              LEDMAGTTIKGWMRYKT-------------------------------------------    274
tr|Q586Y2|Q586Y2_TRYB2              VREKDNVTVQGWVRYE--------------------------------------------    273
tr|Q586Y1|Q586Y1_TRYB2              VREKDNVTVQGWVRYE--------------------------------------------    280
tr|F9W970|F9W970_TRYCI              VHEEGNIVVQGWVRYE--------------------------------------------    274
tr|F9W971|F9W971_TRYCI              VHEEGNIVVQGWVRYE--------------------------------------------    275
tr|S9UGT3|S9UGT3_9TRYP              LVDVEG-TLYGWMRYG--------------------------------------------    265
tr|A0A1X0NR96|A0A1X0NR96_9TRYP      LKESDDIIIQGWMRYSK-------------------------------------------    286
tr|A0A1X0NR98|A0A1X0NR98_9TRYP      LKESDDIIIQGWMRYSK-------------------------------------------    294
tr|Q4D409|Q4D409_TRYCC              ITEEENIIIDGWMRYAT-------------------------------------------    280
tr|Q4D410|Q4D410_TRYCC              ITEEENIIIDGWMRYAT-------------------------------------------    195
tr|Q4CKB1|Q4CKB1_TRYCC              ITEEEN------------------------------------------------------    243
tr|Q4DEC6|Q4DEC6_TRYCC              ITEEENIIIDGWMRYTT-------------------------------------------    310
tr|A0A3R7N0H0|A0A3R7N0H0_9TRYP      VAEADKRTITGWVRYAE-------------------------------------------    311
tr|A0A3R7N2W0|A0A3R7N2W0_9TRYP      VAEADKRTITGWVRYAE-------------------------------------------    283
tr|A0A3S5IRC8|A0A3S5IRC8_TRYRA      VIEADNVIVTGWVRYAM-------------------------------------------    282


tr|A0A815F576|A0A815F576_9BILA      IVSHHNDIRFWGKEV--AHEYISQL-----------------------------------    278
tr|A0A814QMX3|A0A814QMX3_9BILA      ------------------------------------------------------------    208
tr|K2MZ59|K2MZ59_TRYCR              WLNPSQRCDFFLKRSLTVGGYMGEDAVAEAHSVPDGFYIMDYDMWCRFRFATKKLHEVAM    304
tr|A0A0G4IUR7|A0A0G4IUR7_PLABS      ------------------------------------------------------------    225
tr|A0A0N0DV74|A0A0N0DV74_LEPPY      ------------------------------------------------------------    300
tr|A4HEJ1|A4HEJ1_LEIBR              ------------------------------------------------------------    331
tr|Q4Q9R0|Q4Q9R0_LEIMA              ------------------------------------------------------------    300
tr|A4I1M7|A4I1M7_LEIIN              ------------------------------------------------------------    300
tr|S9UNH8|S9UNH8_9TRYP              ------------------------------------------------------------    326
tr|S9UZF8|S9UZF8_9TRYP              ------------------------------------------------------------    314
tr|Q57ZC4|Q57ZC4_TRYB2              ------------------------------------------------------------    289
tr|A0A3R7LGJ5|A0A3R7LGJ5_9TRYP      ------------------------------------------------------------    290
tr|Q4CUZ5|Q4CUZ5_TRYCC              ------------------------------------------------------------    331
tr|Q4D7T1|Q4D7T1_TRYCC              ------------------------------------------------------------    295
tr|A0A3R7KMG1|A0A3R7KMG1_TRYRA      ------------------------------------------------------------    327
tr|Q4CYQ4|Q4CYQ4_TRYCC              ------------------------------------------------------------    287
tr|Q4CUZ4|Q4CUZ4_TRYCC              ------------------------------------------------------------    294
tr|Q4CUZ2|Q4CUZ2_TRYCC              ------------------------------------------------------------    194
tr|Q4CYQ2|Q4CYQ2_TRYCC              ------------------------------------------------------------    164
tr|Q4D7T2|Q4D7T2_TRYCC              ------------------------------------------------------------    294
tr|Q4D7T0|Q4D7T0_TRYCC              ------------------------------------------------------------    164
tr|A0A1X0NY88|A0A1X0NY88_9TRYP      ------------------------------------------------------------    280
tr|A0A1X0NYK9|A0A1X0NYK9_9TRYP      ------------------------------------------------------------    294
tr|A0A1X0NRB4|A0A1X0NRB4_9TRYP      ------------------------------------------------------------    340
tr|Q4D408|Q4D408_TRYCC              ------------------------------------------------------------    330
tr|A0A422N172|A0A422N172_9TRYP      ------------------------------------------------------------    322
tr|A0A422NKH5|A0A422NKH5_TRYRA      ------------------------------------------------------------    329
tr|A0A0S4ITA7|A0A0S4ITA7_BODSA      ------------------------------------------------------------    309
tr|A0A0N0DYP4|A0A0N0DYP4_LEPPY      ------------------------------------------------------------    363
tr|A4HLZ6|A4HLZ6_LEIBR              ------------------------------------------------------------    382
tr|Q4Q3Q0|Q4Q3Q0_LEIMA              ------------------------------------------------------------    421
tr|A4I9C6|A4I9C6_LEIIN              ------------------------------------------------------------    404
tr|A0A0N0DYQ7|A0A0N0DYQ7_LEPPY      ------------------------------------------------------------    407
tr|A4HLZ4|A4HLZ4_LEIBR              ------------------------------------------------------------    334
tr|Q4Q3Q2|Q4Q3Q2_LEIMA              ------------------------------------------------------------    232
tr|A4I9C4|A4I9C4_LEIIN              ------------------------------------------------------------    369
tr|Q384E7|Q384E7_TRYB2              ------------------------------------------------------------    274
tr|Q586Y2|Q586Y2_TRYB2              ------------------------------------------------------------    273
tr|Q586Y1|Q586Y1_TRYB2              ------------------------------------------------------------    280
tr|F9W970|F9W970_TRYCI              ------------------------------------------------------------    274
tr|F9W971|F9W971_TRYCI              ------------------------------------------------------------    275
tr|S9UGT3|S9UGT3_9TRYP              ------------------------------------------------------------    265
tr|A0A1X0NR96|A0A1X0NR96_9TRYP      ------------------------------------------------------------    286
tr|A0A1X0NR98|A0A1X0NR98_9TRYP      ------------------------------------------------------------    294
tr|Q4D409|Q4D409_TRYCC              ------------------------------------------------------------    280
tr|Q4D410|Q4D410_TRYCC              ------------------------------------------------------------    195
tr|Q4CKB1|Q4CKB1_TRYCC              ------------------------------------------------------------    243
tr|Q4DEC6|Q4DEC6_TRYCC              ------------------------------------------------------------    310
tr|A0A3R7N0H0|A0A3R7N0H0_9TRYP      ------------------------------------------------------------    311
tr|A0A3R7N2W0|A0A3R7N2W0_9TRYP      ------------------------------------------------------------    283
tr|A0A3S5IRC8|A0A3S5IRC8_TRYRA      ------------------------------------------------------------    282


tr|A0A815F576|A0A815F576_9BILA      ------------------------------------------------------------    278
tr|A0A814QMX3|A0A814QMX3_9BILA      ------------------------------------------------------------    208
tr|K2MZ59|K2MZ59_TRYCR              ETTRLILNSRDSEALLVQYFGLPTELHLQLRRSFETIPSMCGRFDFGYDGKEIMMLAYKC    364
tr|A0A0G4IUR7|A0A0G4IUR7_PLABS      ------------------------------------------------------------    225
tr|A0A0N0DV74|A0A0N0DV74_LEPPY      ------------------------------------------------------------    300
tr|A4HEJ1|A4HEJ1_LEIBR              ------------------------------------------------------------    331
tr|Q4Q9R0|Q4Q9R0_LEIMA              ------------------------------------------------------------    300
tr|A4I1M7|A4I1M7_LEIIN              ------------------------------------------------------------    300
tr|S9UNH8|S9UNH8_9TRYP              ------------------------------------------------------------    326
```

```
tr|S9UZF8|S9UZF8_9TRYP              ------------------------------------------------------------    314
tr|Q57ZC4|Q57ZC4_TRYB2              ------------------------------------------------------------    289
tr|A0A3R7LGJ5|A0A3R7LGJ5_9TRYP      ------------------------------------------------------------    290
tr|Q4CUZ5|Q4CUZ5_TRYCC              ------------------------------------------------------------    331
tr|Q4D7T1|Q4D7T1_TRYCC              ------------------------------------------------------------    295
tr|A0A3R7KMG1|A0A3R7KMG1_TRYRA      ------------------------------------------------------------    327
tr|Q4CYQ4|Q4CYQ4_TRYCC              ------------------------------------------------------------    287
tr|Q4CUZ4|Q4CUZ4_TRYCC              ------------------------------------------------------------    294
tr|Q4CUZ2|Q4CUZ2_TRYCC              ------------------------------------------------------------    194
tr|Q4CYQ2|Q4CYQ2_TRYCC              ------------------------------------------------------------    164
tr|Q4D7T2|Q4D7T2_TRYCC              ------------------------------------------------------------    294
tr|Q4D7T0|Q4D7T0_TRYCC              ------------------------------------------------------------    164
tr|A0A1X0NY88|A0A1X0NY88_9TRYP      ------------------------------------------------------------    280
tr|A0A1X0NYK9|A0A1X0NYK9_9TRYP      ------------------------------------------------------------    294
tr|A0A1X0NRB4|A0A1X0NRB4_9TRYP      ------------------------------------------------------------    340
tr|Q4D408|Q4D408_TRYCC              ------------------------------------------------------------    330
tr|A0A422N172|A0A422N172_9TRYP      ------------------------------------------------------------    322
tr|A0A422NKH5|A0A422NKH5_TRYRA      ------------------------------------------------------------    329
tr|A0A0S4ITA7|A0A0S4ITA7_BODSA      ------------------------------------------------------------    309
tr|A0A0N0DYP4|A0A0N0DYP4_LEPPY      ------------------------------------------------------------    363
tr|A4HLZ6|A4HLZ6_LEIBR              ------------------------------------------------------------    382
tr|Q4Q3Q0|Q4Q3Q0_LEIMA              ------------------------------------------------------------    421
tr|A4I9C6|A4I9C6_LEIIN              ------------------------------------------------------------    404
tr|A0A0N0DYQ7|A0A0N0DYQ7_LEPPY      ------------------------------------------------------------    407
tr|A4HLZ4|A4HLZ4_LEIBR              ------------------------------------------------------------    334
tr|Q4Q3Q2|Q4Q3Q2_LEIMA              ------------------------------------------------------------    232
tr|A4I9C4|A4I9C4_LEIIN              ------------------------------------------------------------    369
tr|Q384E7|Q384E7_TRYB2              ------------------------------------------------------------    274
tr|Q586Y2|Q586Y2_TRYB2              ------------------------------------------------------------    273
tr|Q586Y1|Q586Y1_TRYB2              ------------------------------------------------------------    280
tr|F9W970|F9W970_TRYCI              ------------------------------------------------------------    274
tr|F9W971|F9W971_TRYCI              ------------------------------------------------------------    275
tr|S9UGT3|S9UGT3_9TRYP              ------------------------------------------------------------    265
tr|A0A1X0NR96|A0A1X0NR96_9TRYP      ------------------------------------------------------------    286
tr|A0A1X0NR98|A0A1X0NR98_9TRYP      ------------------------------------------------------------    294
tr|Q4D409|Q4D409_TRYCC              ------------------------------------------------------------    280
tr|Q4D410|Q4D410_TRYCC              ------------------------------------------------------------    195
tr|Q4CKB1|Q4CKB1_TRYCC              ------------------------------------------------------------    243
tr|Q4DEC6|Q4DEC6_TRYCC              ------------------------------------------------------------    310
tr|A0A3R7N0H0|A0A3R7N0H0_9TRYP      ------------------------------------------------------------    311
tr|A0A3R7N2W0|A0A3R7N2W0_9TRYP      ------------------------------------------------------------    283
tr|A0A3S5IRC8|A0A3S5IRC8_TRYRA      ------------------------------------------------------------    282


tr|A0A815F576|A0A815F576_9BILA      ------------------------------------------------------------    278
tr|A0A814QMX3|A0A814QMX3_9BILA      ------------------------------------------------------------    208
tr|K2MZ59|K2MZ59_TRYCR              DSSAAMLECGDTQEKFTRHYGVKLGNSTGSFLYSRIVNYFSCLMQNECLCPHHKIVHFMI    424
tr|A0A0G4IUR7|A0A0G4IUR7_PLABS      ------------------------------------------------------------    225
tr|A0A0N0DV74|A0A0N0DV74_LEPPY      ------------------------------------------------------------    300
tr|A4HEJ1|A4HEJ1_LEIBR              ------------------------------------------------------------    331
tr|Q4Q9R0|Q4Q9R0_LEIMA              ------------------------------------------------------------    300
tr|A4I1M7|A4I1M7_LEIIN              ------------------------------------------------------------    300
tr|S9UNH8|S9UNH8_9TRYP              ------------------------------------------------------------    326
tr|S9UZF8|S9UZF8_9TRYP              ------------------------------------------------------------    314
tr|Q57ZC4|Q57ZC4_TRYB2              ------------------------------------------------------------    289
tr|A0A3R7LGJ5|A0A3R7LGJ5_9TRYP      ------------------------------------------------------------    290
tr|Q4CUZ5|Q4CUZ5_TRYCC              ------------------------------------------------------------    331
tr|Q4D7T1|Q4D7T1_TRYCC              ------------------------------------------------------------    295
tr|A0A3R7KMG1|A0A3R7KMG1_TRYRA      ------------------------------------------------------------    327
tr|Q4CYQ4|Q4CYQ4_TRYCC              ------------------------------------------------------------    287
tr|Q4CUZ4|Q4CUZ4_TRYCC              ------------------------------------------------------------    294
tr|Q4CUZ2|Q4CUZ2_TRYCC              ------------------------------------------------------------    194
tr|Q4CYQ2|Q4CYQ2_TRYCC              ------------------------------------------------------------    164
tr|Q4D7T2|Q4D7T2_TRYCC              ------------------------------------------------------------    294
tr|Q4D7T0|Q4D7T0_TRYCC              ------------------------------------------------------------    164
tr|A0A1X0NY88|A0A1X0NY88_9TRYP      ------------------------------------------------------------    280
tr|A0A1X0NYK9|A0A1X0NYK9_9TRYP      ------------------------------------------------------------    294
tr|A0A1X0NRB4|A0A1X0NRB4_9TRYP      ------------------------------------------------------------    340
tr|Q4D408|Q4D408_TRYCC              ------------------------------------------------------------    330
tr|A0A422N172|A0A422N172_9TRYP      ------------------------------------------------------------    322
tr|A0A422NKH5|A0A422NKH5_TRYRA      ------------------------------------------------------------    329
tr|A0A0S4ITA7|A0A0S4ITA7_BODSA      ------------------------------------------------------------    309
tr|A0A0N0DYP4|A0A0N0DYP4_LEPPY      ------------------------------------------------------------    363
tr|A4HLZ6|A4HLZ6_LEIBR              ------------------------------------------------------------    382
tr|Q4Q3Q0|Q4Q3Q0_LEIMA              ------------------------------------------------------------    421
tr|A4I9C6|A4I9C6_LEIIN              ------------------------------------------------------------    404
tr|A0A0N0DYQ7|A0A0N0DYQ7_LEPPY      ------------------------------------------------------------    407
tr|A4HLZ4|A4HLZ4_LEIBR              ------------------------------------------------------------    334
tr|Q4Q3Q2|Q4Q3Q2_LEIMA              ------------------------------------------------------------    232
tr|A4I9C4|A4I9C4_LEIIN              ------------------------------------------------------------    369
tr|Q384E7|Q384E7_TRYB2              ------------------------------------------------------------    274
tr|Q586Y2|Q586Y2_TRYB2              ------------------------------------------------------------    273
tr|Q586Y1|Q586Y1_TRYB2              ------------------------------------------------------------    280
tr|F9W970|F9W970_TRYCI              ------------------------------------------------------------    274
tr|F9W971|F9W971_TRYCI              ------------------------------------------------------------    275
tr|S9UGT3|S9UGT3_9TRYP              ------------------------------------------------------------    265
tr|A0A1X0NR96|A0A1X0NR96_9TRYP      ------------------------------------------------------------    286
```

```
tr|A0A1X0NR98|A0A1X0NR98_9TRYP      ------------------------------------------------------------    294
tr|Q4D409|Q4D409_TRYCC              ------------------------------------------------------------    280
tr|Q4D410|Q4D410_TRYCC              ------------------------------------------------------------    195
tr|Q4CKB1|Q4CKB1_TRYCC              ------------------------------------------------------------    243
tr|Q4DEC6|Q4DEC6_TRYCC              ------------------------------------------------------------    310
tr|A0A3R7N0H0|A0A3R7N0H0_9TRYP      ------------------------------------------------------------    311
tr|A0A3R7N2W0|A0A3R7N2W0_9TRYP      ------------------------------------------------------------    283
tr|A0A3S5IRC8|A0A3S5IRC8_TRYRA      ------------------------------------------------------------    282

tr|A0A815F576|A0A815F576_9BILA      ------------------------------------------------------------    278
tr|A0A814QMX3|A0A814QMX3_9BILA      ------------------------------------------------------------    208
tr|K2MZ59|K2MZ59_TRYCR              DDNDEERYTALYVMNAAESVGFRTKLCVKLSDFRFGSSTGEVDLDASPTERRSVVDLENE    484
tr|A0A0G4IUR7|A0A0G4IUR7_PLABS      ------------------------------------------------------------    225
tr|A0A0N0DV74|A0A0N0DV74_LEPPY      ------------------------------------------------------------    300
tr|A4HEJ1|A4HEJ1_LEIBR              ------------------------------------------------------------    331
tr|Q4Q9R0|Q4Q9R0_LEIMA              ------------------------------------------------------------    300
tr|A4I1M7|A4I1M7_LEIIN              ------------------------------------------------------------    300
tr|S9UNH8|S9UNH8_9TRYP              ------------------------------------------------------------    326
tr|S9UZF8|S9UZF8_9TRYP              ------------------------------------------------------------    314
tr|Q57ZC4|Q57ZC4_TRYB2              ------------------------------------------------------------    289
tr|A0A3R7LGJ5|A0A3R7LGJ5_9TRYP      ------------------------------------------------------------    290
tr|Q4CUZ5|Q4CUZ5_TRYCC              ------------------------------------------------------------    331
tr|Q4D7T1|Q4D7T1_TRYCC              ------------------------------------------------------------    295
tr|A0A3R7KMG1|A0A3R7KMG1_TRYRA      ------------------------------------------------------------    327
tr|Q4CYQ4|Q4CYQ4_TRYCC              ------------------------------------------------------------    287
tr|Q4CUZ4|Q4CUZ4_TRYCC              ------------------------------------------------------------    294
tr|Q4CUZ2|Q4CUZ2_TRYCC              ------------------------------------------------------------    194
tr|Q4CYQ2|Q4CYQ2_TRYCC              ------------------------------------------------------------    164
tr|Q4D7T2|Q4D7T2_TRYCC              ------------------------------------------------------------    294
tr|Q4D7T0|Q4D7T0_TRYCC              ------------------------------------------------------------    164
tr|A0A1X0NY88|A0A1X0NY88_9TRYP      ------------------------------------------------------------    280
tr|A0A1X0NYK9|A0A1X0NYK9_9TRYP      ------------------------------------------------------------    294
tr|A0A1X0NRB4|A0A1X0NRB4_9TRYP      ------------------------------------------------------------    340
tr|Q4D408|Q4D408_TRYCC              ------------------------------------------------------------    330
tr|A0A422N172|A0A422N172_9TRYP      ------------------------------------------------------------    322
tr|A0A422NKH5|A0A422NKH5_TRYRA      ------------------------------------------------------------    329
tr|A0A0S4ITA7|A0A0S4ITA7_BODSA      ------------------------------------------------------------    309
tr|A0A0N0DYP4|A0A0N0DYP4_LEPPY      ------------------------------------------------------------    363
tr|A4HLZ6|A4HLZ6_LEIBR              ------------------------------------------------------------    382
tr|Q4Q3Q0|Q4Q3Q0_LEIMA              ------------------------------------------------------------    421
tr|A4I9C6|A4I9C6_LEIIN              ------------------------------------------------------------    404
tr|A0A0N0DYQ7|A0A0N0DYQ7_LEPPY      ------------------------------------------------------------    407
tr|A4HLZ4|A4HLZ4_LEIBR              ------------------------------------------------------------    334
tr|Q4Q3Q2|Q4Q3Q2_LEIMA              ------------------------------------------------------------    232
tr|A4I9C4|A4I9C4_LEIIN              ------------------------------------------------------------    369
tr|Q384E7|Q384E7_TRYB2              ------------------------------------------------------------    274
tr|Q586Y2|Q586Y2_TRYB2              ------------------------------------------------------------    273
tr|Q586Y1|Q586Y1_TRYB2              ------------------------------------------------------------    280
tr|F9W970|F9W970_TRYCI              ------------------------------------------------------------    274
tr|F9W971|F9W971_TRYCI              ------------------------------------------------------------    275
tr|S9UGT3|S9UGT3_9TRYP              ------------------------------------------------------------    265
tr|A0A1X0NR96|A0A1X0NR96_9TRYP      ------------------------------------------------------------    286
tr|A0A1X0NR98|A0A1X0NR98_9TRYP      ------------------------------------------------------------    294
tr|Q4D409|Q4D409_TRYCC              ------------------------------------------------------------    280
tr|Q4D410|Q4D410_TRYCC              ------------------------------------------------------------    195
tr|Q4CKB1|Q4CKB1_TRYCC              ------------------------------------------------------------    243
tr|Q4DEC6|Q4DEC6_TRYCC              ------------------------------------------------------------    310
tr|A0A3R7N0H0|A0A3R7N0H0_9TRYP      ------------------------------------------------------------    311
tr|A0A3R7N2W0|A0A3R7N2W0_9TRYP      ------------------------------------------------------------    283
tr|A0A3S5IRC8|A0A3S5IRC8_TRYRA      ------------------------------------------------------------    282

tr|A0A815F576|A0A815F576_9BILA      ------------------------------------------------------------    278
tr|A0A814QMX3|A0A814QMX3_9BILA      ------------------------------------------------------------    208
tr|K2MZ59|K2MZ59_TRYCR              KVLLVWKTWAWDTVVQQYMEQRTQEGHGVSTKPTLSDILLNEHIRVLEPLWKAVTGSKAI    544
tr|A0A0G4IUR7|A0A0G4IUR7_PLABS      ------------------------------------------------------------    225
tr|A0A0N0DV74|A0A0N0DV74_LEPPY      ------------------------------------------------------------    300
tr|A4HEJ1|A4HEJ1_LEIBR              ------------------------------------------------------------    331
tr|Q4Q9R0|Q4Q9R0_LEIMA              ------------------------------------------------------------    300
tr|A4I1M7|A4I1M7_LEIIN              ------------------------------------------------------------    300
tr|S9UNH8|S9UNH8_9TRYP              ------------------------------------------------------------    326
tr|S9UZF8|S9UZF8_9TRYP              ------------------------------------------------------------    314
tr|Q57ZC4|Q57ZC4_TRYB2              ------------------------------------------------------------    289
tr|A0A3R7LGJ5|A0A3R7LGJ5_9TRYP      ------------------------------------------------------------    290
tr|Q4CUZ5|Q4CUZ5_TRYCC              ------------------------------------------------------------    331
tr|Q4D7T1|Q4D7T1_TRYCC              ------------------------------------------------------------    295
tr|A0A3R7KMG1|A0A3R7KMG1_TRYRA      ------------------------------------------------------------    327
tr|Q4CYQ4|Q4CYQ4_TRYCC              ------------------------------------------------------------    287
tr|Q4CUZ4|Q4CUZ4_TRYCC              ------------------------------------------------------------    294
tr|Q4CUZ2|Q4CUZ2_TRYCC              ------------------------------------------------------------    194
tr|Q4CYQ2|Q4CYQ2_TRYCC              ------------------------------------------------------------    164
tr|Q4D7T2|Q4D7T2_TRYCC              ------------------------------------------------------------    294
tr|Q4D7T0|Q4D7T0_TRYCC              ------------------------------------------------------------    164
tr|A0A1X0NY88|A0A1X0NY88_9TRYP      ------------------------------------------------------------    280
tr|A0A1X0NYK9|A0A1X0NYK9_9TRYP      ------------------------------------------------------------    294
tr|A0A1X0NRB4|A0A1X0NRB4_9TRYP      ------------------------------------------------------------    340
```

```
tr|Q4D408|Q4D408_TRYCC                ------------------------------------------------------------   330
tr|A0A422N172|A0A422N172_9TRYP        ------------------------------------------------------------   322
tr|A0A422NKH5|A0A422NKH5_TRYRA        ------------------------------------------------------------   329
tr|A0A0S4ITA7|A0A0S4ITA7_BODSA        ------------------------------------------------------------   309
tr|A0A0N0DYP4|A0A0N0DYP4_LEPPY        ------------------------------------------------------------   363
tr|A4HLZ6|A4HLZ6_LEIBR                ------------------------------------------------------------   382
tr|Q4Q3Q0|Q4Q3Q0_LEIMA                ------------------------------------------------------------   421
tr|A4I9C6|A4I9C6_LEIIN                ------------------------------------------------------------   404
tr|A0A0N0DYQ7|A0A0N0DYQ7_LEPPY        ------------------------------------------------------------   407
tr|A4HLZ4|A4HLZ4_LEIBR                ------------------------------------------------------------   334
tr|Q4Q3Q2|Q4Q3Q2_LEIMA                ------------------------------------------------------------   232
tr|A4I9C4|A4I9C4_LEIIN                ------------------------------------------------------------   369
tr|Q384E7|Q384E7_TRYB2                ------------------------------------------------------------   274
tr|Q586Y2|Q586Y2_TRYB2                ------------------------------------------------------------   273
tr|Q586Y1|Q586Y1_TRYB2                ------------------------------------------------------------   280
tr|F9W970|F9W970_TRYCI                ------------------------------------------------------------   274
tr|F9W971|F9W971_TRYCI                ------------------------------------------------------------   275
tr|S9UGT3|S9UGT3_9TRYP                ------------------------------------------------------------   265
tr|A0A1X0NR96|A0A1X0NR96_9TRYP        ------------------------------------------------------------   286
tr|A0A1X0NR98|A0A1X0NR98_9TRYP        ------------------------------------------------------------   294
tr|Q4D409|Q4D409_TRYCC                ------------------------------------------------------------   280
tr|Q4D410|Q4D410_TRYCC                ------------------------------------------------------------   195
tr|Q4CKB1|Q4CKB1_TRYCC                ------------------------------------------------------------   243
tr|Q4DEC6|Q4DEC6_TRYCC                ------------------------------------------------------------   310
tr|A0A3R7N0H0|A0A3R7N0H0_9TRYP        ------------------------------------------------------------   311
tr|A0A3R7N2W0|A0A3R7N2W0_9TRYP        ------------------------------------------------------------   283
tr|A0A3S5IRC8|A0A3S5IRC8_TRYRA        ------------------------------------------------------------   282


tr|A0A815F576|A0A815F576_9BILA        ------------------------------------------------------------   278
tr|A0A814QMX3|A0A814QMX3_9BILA        ------------------------------------------------------------   208
tr|K2MZ59|K2MZ59_TRYCR                LPFMYAVAPHHPNMVPASFYRTKEIISSPYLTKPVNGRVGKNMTIYDPEENPEAIAAAPY   604
tr|A0A0G4IUR7|A0A0G4IUR7_PLABS        ------------------------------------------------------------   225
tr|A0A0N0DV74|A0A0N0DV74_LEPPY        ------------------------------------------------------------   300
tr|A4HEJ1|A4HEJ1_LEIBR                ------------------------------------------------------------   331
tr|Q4Q9R0|Q4Q9R0_LEIMA                ------------------------------------------------------------   300
tr|A4I1M7|A4I1M7_LEIIN                ------------------------------------------------------------   300
tr|S9UNH8|S9UNH8_9TRYP                ------------------------------------------------------------   326
tr|S9UZF8|S9UZF8_9TRYP                ------------------------------------------------------------   314
tr|Q57ZC4|Q57ZC4_TRYB2                ------------------------------------------------------------   289
tr|A0A3R7LGJ5|A0A3R7LGJ5_9TRYP        ------------------------------------------------------------   290
tr|Q4CUZ5|Q4CUZ5_TRYCC                ------------------------------------------------------------   331
tr|Q4D7T1|Q4D7T1_TRYCC                ------------------------------------------------------------   295
tr|A0A3R7KMG1|A0A3R7KMG1_TRYRA        ------------------------------------------------------------   327
tr|Q4CYQ4|Q4CYQ4_TRYCC                ------------------------------------------------------------   287
tr|Q4CUZ4|Q4CUZ4_TRYCC                ------------------------------------------------------------   294
tr|Q4CUZ2|Q4CUZ2_TRYCC                ------------------------------------------------------------   194
tr|Q4CYQ2|Q4CYQ2_TRYCC                ------------------------------------------------------------   164
tr|Q4D7T2|Q4D7T2_TRYCC                ------------------------------------------------------------   294
tr|Q4D7T0|Q4D7T0_TRYCC                ------------------------------------------------------------   164
tr|A0A1X0NY88|A0A1X0NY88_9TRYP        ------------------------------------------------------------   280
tr|A0A1X0NYK9|A0A1X0NYK9_9TRYP        ------------------------------------------------------------   294
tr|A0A1X0NRB4|A0A1X0NRB4_9TRYP        ------------------------------------------------------------   340
tr|Q4D408|Q4D408_TRYCC                ------------------------------------------------------------   330
tr|A0A422N172|A0A422N172_9TRYP        ------------------------------------------------------------   322
tr|A0A422NKH5|A0A422NKH5_TRYRA        ------------------------------------------------------------   329
tr|A0A0S4ITA7|A0A0S4ITA7_BODSA        ------------------------------------------------------------   309
tr|A0A0N0DYP4|A0A0N0DYP4_LEPPY        ------------------------------------------------------------   363
tr|A4HLZ6|A4HLZ6_LEIBR                ------------------------------------------------------------   382
tr|Q4Q3Q0|Q4Q3Q0_LEIMA                ------------------------------------------------------------   421
tr|A4I9C6|A4I9C6_LEIIN                ------------------------------------------------------------   404
tr|A0A0N0DYQ7|A0A0N0DYQ7_LEPPY        ------------------------------------------------------------   407
tr|A4HLZ4|A4HLZ4_LEIBR                ------------------------------------------------------------   334
tr|Q4Q3Q2|Q4Q3Q2_LEIMA                ------------------------------------------------------------   232
tr|A4I9C4|A4I9C4_LEIIN                ------------------------------------------------------------   369
tr|Q384E7|Q384E7_TRYB2                ------------------------------------------------------------   274
tr|Q586Y2|Q586Y2_TRYB2                ------------------------------------------------------------   273
tr|Q586Y1|Q586Y1_TRYB2                ------------------------------------------------------------   280
tr|F9W970|F9W970_TRYCI                ------------------------------------------------------------   274
tr|F9W971|F9W971_TRYCI                ------------------------------------------------------------   275
tr|S9UGT3|S9UGT3_9TRYP                ------------------------------------------------------------   265
tr|A0A1X0NR96|A0A1X0NR96_9TRYP        ------------------------------------------------------------   286
tr|A0A1X0NR98|A0A1X0NR98_9TRYP        ------------------------------------------------------------   294
tr|Q4D409|Q4D409_TRYCC                ------------------------------------------------------------   280
tr|Q4D410|Q4D410_TRYCC                ------------------------------------------------------------   195
tr|Q4CKB1|Q4CKB1_TRYCC                ------------------------------------------------------------   243
tr|Q4DEC6|Q4DEC6_TRYCC                ------------------------------------------------------------   310
tr|A0A3R7N0H0|A0A3R7N0H0_9TRYP        ------------------------------------------------------------   311
tr|A0A3R7N2W0|A0A3R7N2W0_9TRYP        ------------------------------------------------------------   283
tr|A0A3S5IRC8|A0A3S5IRC8_TRYRA        ------------------------------------------------------------   282


tr|A0A815F576|A0A815F576_9BILA        ------------------------------------------------------------   278
tr|A0A814QMX3|A0A814QMX3_9BILA        ------------------------------------------------------------   208
tr|K2MZ59|K2MZ59_TRYCR                TTAAPVLGRSISAVLFDDAPVQPCRMPFDNVHENLTGRLFDSAVVYQSRVFLTRYEKKYS   664
tr|A0A0G4IUR7|A0A0G4IUR7_PLABS        ------------------------------------------------------------   225
tr|A0A0N0DV74|A0A0N0DV74_LEPPY        ------------------------------------------------------------   300
```

```
tr|A4HEJ1|A4HEJ1_LEIBR                  ------------------------------------------------------------    331
tr|Q4Q9R0|Q4Q9R0_LEIMA                  ------------------------------------------------------------    300
tr|A4I1M7|A4I1M7_LEIIN                  ------------------------------------------------------------    300
tr|S9UNH8|S9UNH8_9TRYP                  ------------------------------------------------------------    326
tr|S9UZF8|S9UZF8_9TRYP                  ------------------------------------------------------------    314
tr|Q57ZC4|Q57ZC4_TRYB2                  ------------------------------------------------------------    289
tr|A0A3R7LGJ5|A0A3R7LGJ5_9TRYP          ------------------------------------------------------------    290
tr|Q4CUZ5|Q4CUZ5_TRYCC                  ------------------------------------------------------------    331
tr|Q4D7T1|Q4D7T1_TRYCC                  ------------------------------------------------------------    295
tr|A0A3R7KMG1|A0A3R7KMG1_TRYRA          ------------------------------------------------------------    327
tr|Q4CYQ4|Q4CYQ4_TRYCC                  ------------------------------------------------------------    287
tr|Q4CUZ4|Q4CUZ4_TRYCC                  ------------------------------------------------------------    294
tr|Q4CUZ2|Q4CUZ2_TRYCC                  ------------------------------------------------------------    194
tr|Q4CYQ2|Q4CYQ2_TRYCC                  ------------------------------------------------------------    164
tr|Q4D7T2|Q4D7T2_TRYCC                  ------------------------------------------------------------    294
tr|Q4D7T0|Q4D7T0_TRYCC                  ------------------------------------------------------------    164
tr|A0A1X0NY88|A0A1X0NY88_9TRYP          ------------------------------------------------------------    280
tr|A0A1X0NYK9|A0A1X0NYK9_9TRYP          ------------------------------------------------------------    294
tr|A0A1X0NRB4|A0A1X0NRB4_9TRYP          ------------------------------------------------------------    340
tr|Q4D408|Q4D408_TRYCC                  ------------------------------------------------------------    330
tr|A0A422N172|A0A422N172_9TRYP          ------------------------------------------------------------    322
tr|A0A422NKH5|A0A422NKH5_TRYRA          ------------------------------------------------------------    329
tr|A0A0S4ITA7|A0A0S4ITA7_BODSA          ------------------------------------------------------------    309
tr|A0A0N0DYP4|A0A0N0DYP4_LEPPY          ------------------------------------------------------------    363
tr|A4HLZ6|A4HLZ6_LEIBR                  ------------------------------------------------------------    382
tr|Q4Q3Q0|Q4Q3Q0_LEIMA                  ------------------------------------------------------------    421
tr|A4I9C6|A4I9C6_LEIIN                  ------------------------------------------------------------    404
tr|A0A0N0DYQ7|A0A0N0DYQ7_LEPPY          ------------------------------------------------------------    407
tr|A4HLZ4|A4HLZ4_LEIBR                  ------------------------------------------------------------    334
tr|Q4Q3Q2|Q4Q3Q2_LEIMA                  ------------------------------------------------------------    232
tr|A4I9C4|A4I9C4_LEIIN                  ------------------------------------------------------------    369
tr|Q384E7|Q384E7_TRYB2                  ------------------------------------------------------------    274
tr|Q586Y2|Q586Y2_TRYB2                  ------------------------------------------------------------    273
tr|Q586Y1|Q586Y1_TRYB2                  ------------------------------------------------------------    280
tr|F9W970|F9W970_TRYCI                  ------------------------------------------------------------    274
tr|F9W971|F9W971_TRYCI                  ------------------------------------------------------------    275
tr|S9UGT3|S9UGT3_9TRYP                  ------------------------------------------------------------    265
tr|A0A1X0NR96|A0A1X0NR96_9TRYP          ------------------------------------------------------------    286
tr|A0A1X0NR98|A0A1X0NR98_9TRYP          ------------------------------------------------------------    294
tr|Q4D409|Q4D409_TRYCC                  ------------------------------------------------------------    280
tr|Q4D410|Q4D410_TRYCC                  ------------------------------------------------------------    195
tr|Q4CKB1|Q4CKB1_TRYCC                  ------------------------------------------------------------    243
tr|Q4DEC6|Q4DEC6_TRYCC                  ------------------------------------------------------------    310
tr|A0A3R7N0H0|A0A3R7N0H0_9TRYP          ------------------------------------------------------------    311
tr|A0A3R7N2W0|A0A3R7N2W0_9TRYP          ------------------------------------------------------------    283
tr|A0A3S5IRC8|A0A3S5IRC8_TRYRA          ------------------------------------------------------------    282


tr|A0A815F576|A0A815F576_9BILA          -------------------------------------------------------    278
tr|A0A814QMX3|A0A814QMX3_9BILA          -------------------------------------------------------    208
tr|K2MZ59|K2MZ59_TRYCR                  PIFCGWNVGGEFGGVIVREETLNTTGLSSLVIPSRVVRQHIPLHAVQESTTFGEV    719
tr|A0A0G4IUR7|A0A0G4IUR7_PLABS          -------------------------------------------------------    225
tr|A0A0N0DV74|A0A0N0DV74_LEPPY          -------------------------------------------------------    300
tr|A4HEJ1|A4HEJ1_LEIBR                  -------------------------------------------------------    331
tr|Q4Q9R0|Q4Q9R0_LEIMA                  -------------------------------------------------------    300
tr|A4I1M7|A4I1M7_LEIIN                  -------------------------------------------------------    300
tr|S9UNH8|S9UNH8_9TRYP                  -------------------------------------------------------    326
tr|S9UZF8|S9UZF8_9TRYP                  -------------------------------------------------------    314
tr|Q57ZC4|Q57ZC4_TRYB2                  -------------------------------------------------------    289
tr|A0A3R7LGJ5|A0A3R7LGJ5_9TRYP          -------------------------------------------------------    290
tr|Q4CUZ5|Q4CUZ5_TRYCC                  -------------------------------------------------------    331
tr|Q4D7T1|Q4D7T1_TRYCC                  -------------------------------------------------------    295
tr|A0A3R7KMG1|A0A3R7KMG1_TRYRA          -------------------------------------------------------    327
tr|Q4CYQ4|Q4CYQ4_TRYCC                  -------------------------------------------------------    287
tr|Q4CUZ4|Q4CUZ4_TRYCC                  -------------------------------------------------------    294
tr|Q4CUZ2|Q4CUZ2_TRYCC                  -------------------------------------------------------    194
tr|Q4CYQ2|Q4CYQ2_TRYCC                  -------------------------------------------------------    164
tr|Q4D7T2|Q4D7T2_TRYCC                  -------------------------------------------------------    294
tr|Q4D7T0|Q4D7T0_TRYCC                  -------------------------------------------------------    164
tr|A0A1X0NY88|A0A1X0NY88_9TRYP          -------------------------------------------------------    280
tr|A0A1X0NYK9|A0A1X0NYK9_9TRYP          -------------------------------------------------------    294
tr|A0A1X0NRB4|A0A1X0NRB4_9TRYP          -------------------------------------------------------    340
tr|Q4D408|Q4D408_TRYCC                  -------------------------------------------------------    330
tr|A0A422N172|A0A422N172_9TRYP          -------------------------------------------------------    322
tr|A0A422NKH5|A0A422NKH5_TRYRA          -------------------------------------------------------    329
tr|A0A0S4ITA7|A0A0S4ITA7_BODSA          -------------------------------------------------------    309
tr|A0A0N0DYP4|A0A0N0DYP4_LEPPY          -------------------------------------------------------    363
tr|A4HLZ6|A4HLZ6_LEIBR                  -------------------------------------------------------    382
tr|Q4Q3Q0|Q4Q3Q0_LEIMA                  -------------------------------------------------------    421
tr|A4I9C6|A4I9C6_LEIIN                  -------------------------------------------------------    404
tr|A0A0N0DYQ7|A0A0N0DYQ7_LEPPY          -------------------------------------------------------    407
tr|A4HLZ4|A4HLZ4_LEIBR                  -------------------------------------------------------    334
tr|Q4Q3Q2|Q4Q3Q2_LEIMA                  -------------------------------------------------------    232
tr|A4I9C4|A4I9C4_LEIIN                  -------------------------------------------------------    369
tr|Q384E7|Q384E7_TRYB2                  -------------------------------------------------------    274
tr|Q586Y2|Q586Y2_TRYB2                  -------------------------------------------------------    273
tr|Q586Y1|Q586Y1_TRYB2                  -------------------------------------------------------    280
```

```
tr|F9W970|F9W970_TRYCI                  -------------------------------------------------------   274
tr|F9W971|F9W971_TRYCI                  -------------------------------------------------------   275
tr|S9UGT3|S9UGT3_9TRYP                  -------------------------------------------------------   265
tr|A0A1X0NR96|A0A1X0NR96_9TRYP          -------------------------------------------------------   286
tr|A0A1X0NR98|A0A1X0NR98_9TRYP          -------------------------------------------------------   294
tr|Q4D409|Q4D409_TRYCC                  -------------------------------------------------------   280
tr|Q4D410|Q4D410_TRYCC                  -------------------------------------------------------   195
tr|Q4CKB1|Q4CKB1_TRYCC                  -------------------------------------------------------   243
tr|Q4DEC6|Q4DEC6_TRYCC                  -------------------------------------------------------   310
tr|A0A3R7N0H0|A0A3R7N0H0_9TRYP          -------------------------------------------------------   311
tr|A0A3R7N2W0|A0A3R7N2W0_9TRYP          -------------------------------------------------------   283
tr|A0A3S5IRC8|A0A3S5IRC8_TRYRA          -------------------------------------------------------   282
```

(overall sequence identity = 0.0074)

06. Leishmaniid small secreted / ER proteins (LESSER)

This is a very unique family identified only among kinetoplastids. These proteins are very small, and consist only of a single domain. Even though kinetoplastid organisms typically only have one ortholog, there is a significant variability: it is likely secreted in most groups (without KDEL), apparently retained in the ER in leishmaniids (Leishmania + Leptomonas group) and very rarely can even be anchored to the membrane in others. The biological function of these proteins is utterly unclear.

```
tr|A0A0S4JVT8|A0A0S4JVT8_BODSA          ------------------------------------------------------------    0
tr|A0A0M9G0R4|A0A0M9G0R4_LEPPY          ------------------------------------------------------------    0
tr|A4HNW9|A4HNW9_LEIBR                  ------------------------------------------------------------    0
tr|Q4Q1W7|Q4Q1W7_LEIMA                  ------------------------------------------------------------    0
tr|A4ICM5|A4ICM5_LEIIN                  ------------------------------------------------------------    0
tr|A0A1X0P821|A0A1X0P821_9TRYP          ------------------------------------------------------------    0
tr|S9TGZ6|S9TGZ6_9TRYP                  ------------------------------------------------------------    0
tr|A0A7G2C2Y3|A0A7G2C2Y3_9TRYP          --------------------------MFIRCRFAPFELKRISFF----------------    18
tr|A0A1X0P888|A0A1X0P888_9TRYP          ------------------------------------------------------------    0
tr|A0A3R7KQP7|A0A3R7KQP7_9TRYP          ------------------------------------------------------------    0
tr|A0A3R7NU99|A0A3R7NU99_TRYRA          ------------------------------------------------------------    0
tr|Q4CWZ8|Q4CWZ8_TRYCC                  MDGKYIYIRFCPMHGPFFFFFLVNRMIFACPPFAPMEFSLYLFLFFYSSPITHPGQNNNK    60
tr|Q4CZ14|Q4CZ14_TRYCC                  -----------------------------------MEFPLYLFLFFYSSPITHPGQNNK-    24

tr|A0A0S4JVT8|A0A0S4JVT8_BODSA          -------------------MLSLKFVL-SVVVVAFLVAAVTGEDGAPAVSVKRIG--TNS    38
tr|A0A0M9G0R4|A0A0M9G0R4_LEPPY          ----------------MRPFLR-AVSMVSLCFAYFLLVGVSAQA--P-----------PR    30
tr|A4HNW9|A4HNW9_LEIBR                  -----------------MPFLR-TFTVFCI-IAGVLLQSCAALT--P-----------SR    28
tr|Q4Q1W7|Q4Q1W7_LEIMA                  ----------------MVSFLR-TFAVLCI-IAGVLLQGGTARA--P-----------PR    29
tr|A4ICM5|A4ICM5_LEIIN                  ----------------MASFLR-TFAILCI-IAGVLLQGSTARA--P-----------PR    29
tr|A0A1X0P821|A0A1X0P821_9TRYP          ----------------------------MIIETA------------A-----------AA    9
tr|S9TGZ6|S9TGZ6_9TRYP                  --------------MTRRCFLVLFLAAVCLCFSA------------D-----------AA    23
tr|A0A7G2C2Y3|A0A7G2C2Y3_9TRYP          -----------------------TILITCF-IPTLLLLL--PPITAVYANPDRYA--TVS    50
tr|A0A1X0P888|A0A1X0P888_9TRYP          ----------------MSQ----NVLQTLA-LVLLSLLLVSAEHTLMQTRTNRIVSRG--    37
tr|A0A3R7KQP7|A0A3R7KQP7_9TRYP          ----------------MKG-VRTVWLVVCV-TLLLQVLSSRAEPSVVPARSQRQSVQHPS    42
tr|A0A3R7NU99|A0A3R7NU99_TRYRA          ----------------MRG-VSIVCFFLCV-TLAFQVLPLHAEPSPIPARAQRSSTQKIP    42
tr|Q4CWZ8|Q4CWZ8_TRYCC                  KKKKENNYLYLALMLGMRG-VCTVWIFVCV-VLMFGLSLSHAELSTTEIQFQELSPPQTS    118
tr|Q4CZ14|Q4CZ14_TRYCC                  KKKKENNYLYLALMLGMRG-VCTVWIFVCV-VLTFGLSLSHAELSTIETQFQELSPPQTS    82

tr|A0A0S4JVT8|A0A0S4JVT8_BODSA          RVRPI----RTTLQACRADFRKLCLKRGENTGAVAPVSRNSPNECLQNQIENIA------    88
tr|A0A0M9G0R4|A0A0M9G0R4_LEPPY          GWRPYESKFNSLSQACYGVLEDACP-----------PNSADRLYCLMLNQDKND------    73
tr|A4HNW9|A4HNW9_LEIBR                  AVRPFETKFSSLSEACRGVLETECP-----------PDRLGRADCLMARTEGNE------    71
tr|Q4Q1W7|Q4Q1W7_LEIMA                  AVRAFETKFNSVSQACRGVLETECP-----------SDGLARTDCLMEHIVDNA------    72
tr|A4ICM5|A4ICM5_LEIIN                  AVRAFETKFNSVSQACRGVLETECP-----------SDGLARTDCLMEHIVDNA------    72
tr|A0A1X0P821|A0A1X0P821_9TRYP          ATVPLYTAPQTITQACWGELQDECP-----------GP---DVDCLANNIIRIK------    49
tr|S9TGZ6|S9TGZ6_9TRYP                  TLGELRAAPRNIAQACRDELTATCD-----------HRYNDFPDCLLTHFKKVK------    66
tr|A0A7G2C2Y3|A0A7G2C2Y3_9TRYP          HAAGLPHDPPSVYHACAGEMALLCP-----------DTPHR-VVCFLSAITREGR-----    93
tr|A0A1X0P888|A0A1X0P888_9TRYP          -TEPSVNAPYFLFSVCEAEIQQFCP-----------SQKEAPLKCLLHHFSAARQNPRGG    85
tr|A0A3R7KQP7|A0A3R7KQP7_9TRYP          SRASPTKFLVFPFAACDADAKRLCP-----------TQKESPLKCLLQHFESQRFS----    87
tr|A0A3R7NU99|A0A3R7NU99_TRYRA          FRASPTDLLVFPFSACDADVKQLCP-----------TQKESPLKCLLQHFESQRSS----    87
tr|Q4CWZ8|Q4CWZ8_TRYCC                  SEVSETNSLTFPFFACEAELRQLCP-----------KQMEAPLKCLLQHFESQRNT----    163
tr|Q4CZ14|Q4CZ14_TRYCC                  SEVSETNSLTFPFFACEAEVRQLCL-----------KQMEAPLKCLLQHFESQRNT----    127
                                                      .*       *                    *:

tr|A0A0S4JVT8|A0A0S4JVT8_BODSA          ------------------------------------------SEVCKTWVSAEQSCTSAL    106
tr|A0A0M9G0R4|A0A0M9G0R4_LEPPY          ------------------------------------------NHECNMWLGWRTVCLSFV    91
tr|A4HNW9|A4HNW9_LEIBR                  ------------------------------------------NHECKLWLSWRATCFAYA    89
tr|Q4Q1W7|Q4Q1W7_LEIMA                  ------------------------------------------NHECKMWLSWRASCFAYA    90
tr|A4ICM5|A4ICM5_LEIIN                  ------------------------------------------NHECKMWLSWRANCFAYA    90
tr|A0A1X0P821|A0A1X0P821_9TRYP          ------------------------------------------SDICRSWITAREVCITHV    67
tr|S9TGZ6|S9TGZ6_9TRYP                  ------------------------------------------SPTCKTWLLARSICNSFV    84
tr|A0A7G2C2Y3|A0A7G2C2Y3_9TRYP          ------------------------------------TPDHTFSPTCQSYLSGRESCVRAV    117
tr|A0A1X0P888|A0A1X0P888_9TRYP          GAAQGRPHLLHAYTQQPQQTLQPTQRPQEEPAETQPRRPIHFGKECSSWLRAREVCVTYV    145
tr|A0A3R7KQP7|A0A3R7KQP7_9TRYP          --------------------------------EAPWRRRPAFSEACRRWLWARQECVSYV    115
tr|A0A3R7NU99|A0A3R7NU99_TRYRA          --------------------------------EYPWRRRPAFSKACSRWLWARQECVSYV    115
```

```
tr|Q4CWZ8|Q4CWZ8_TRYCC            --------------------------------KLPWRRRITFSGACSRWLWARQECVSYV    191
tr|Q4CZ14|Q4CZ14_TRYCC            --------------------------------KLLWRRRITFSSACSRWLWARQECVSFV    155
                                                                             .  *  ::  .  *

tr|A0A0S4JVT8|A0A0S4JVT8_BODSA    K------SNTKCPKNS------DIRRCFGKVSATELPDTCTSTEFYKSFAAVRIRGGL--    152
tr|A0A0M9G0R4|A0A0M9G0R4_LEPPY    EVK--LVPNGFCGFTKEESTPELVRQCLGIVDKKELPELCHKSPYYKSLKLHSPRLMD--    147
tr|A4HNW9|A4HNW9_LEIBR            MVK--LIPQGKCNFTSDQMTSELVLQCLRVTDAKELPTACSASPYFKSIMLHAPRRMD--    145
tr|Q4Q1W7|Q4Q1W7_LEIMA            TVF--LIPQGKCNFKPDQATSQLIRNCLRNTDKSELPAVCSGSPYYKSLTLRSPRRID--    146
tr|A4ICM5|A4ICM5_LEIIN            TVY--LIPQGKCNFKPHQATSQLIRNCLRNTEKSELPAVCSGSPYYKSLTLRVPRRMD--    146
tr|A0A1X0P821|A0A1X0P821_9TRYP    K------EVMKCNDD-------KYRQCIREAHRDELPFLCVNSPYYKSLYRNLPRQFL--    112
tr|S9TGZ6|S9TGZ6_9TRYP            K------NNNKCGYDQ---TIFNYRECIRNVPLDELPVQCRESDYYKSLKRFRFE-----    130
tr|A0A7G2C2Y3|A0A7G2C2Y3_9TRYP    RLGQNKFVSKLCGDTI------NVRECLRRVPSHSLPSKCARSLYYRSVLLAPMLSHGQN    171
tr|A0A1X0P888|A0A1X0P888_9TRYP    RRS------GRCRASE------TARECLRRVPASLLPPGCRNTDYHRSVMLYGEMKRQKR    193
tr|A0A3R7KQP7|A0A3R7KQP7_9TRYP    RLP------GRCRASE------SARDCLRRVPPRDLPPGCRDTEYYRSVLLYGEMKSKQG    163
tr|A0A3R7NU99|A0A3R7NU99_TRYRA    RLA------GKCRVSE------SARDCLRRVPPRDLPPGCRDTEYYRSVLLYGEMKRTQR    163
tr|Q4CWZ8|Q4CWZ8_TRYCC            RQT------GICRASE------SARNCLRRIPPRGIPSGCRETEYYRSVLLYGEMKRKQG    239
tr|Q4CZ14|Q4CZ14_TRYCC            RQT------GICRASE------SARNCLRRIPPRGIPSGCRDTEYYRSVLLYGEMKRKQG    203
                                                   *                *:       :*  *  : :.:*.

tr|A0A0S4JVT8|A0A0S4JVT8_BODSA    --------RNRTKTE--    159
tr|A0A0M9G0R4|A0A0M9G0R4_LEPPY    --------DEGDL----    152
tr|A4HNW9|A4HNW9_LEIBR            --------SDGTADL--    152
tr|Q4Q1W7|Q4Q1W7_LEIMA            --------SDAAADL--    153
tr|A4ICM5|A4ICM5_LEIIN            --------SDAAADL--    153
tr|A0A1X0P821|A0A1X0P821_9TRYP    --------R--------    113
tr|S9TGZ6|S9TGZ6_9TRYP            -----------------    130
tr|A0A7G2C2Y3|A0A7G2C2Y3_9TRYP    -----------------    171
tr|A0A1X0P888|A0A1X0P888_9TRYP    T----------------    194
tr|A0A3R7KQP7|A0A3R7KQP7_9TRYP    MRPRNKSNDNETQKLAE    180
tr|A0A3R7NU99|A0A3R7NU99_TRYRA    MRPPEKSKENRTEQHAE    180
tr|Q4CWZ8|Q4CWZ8_TRYCC            LWKPSNTPKKQQKD---    253
tr|Q4CZ14|Q4CZ14_TRYCC            LWKPSNTPKKQQKN---    217
```

(overall sequence identity = 0.0316)

07. Essential for viability in ER (EVER)

This another very unique protein, consisting of a single domain with a beta-sheet-only architecture (predicted).While its predicted structure loosely resembles certain plant lectins, the exact biological function is unclear.There has been an important publication (Plos Pathogens 2022, PMID: 35202447), where the knockout of this gene (LdBPK_211610) was shown to be lethal in Leishmania donovani. The authors assumed a secreted protein (judged by the signal), but now we know that all kinetoplastid members of this family carry a well-discernible KDEL-like signal, hinting at an ER-localized protein. In accordance with its purported essential function, it is found in all better-studied kinetoplastid genomes, except those where it has likely not been mapped yet.
Some organisms have even duplicated this gene.

```
tr|A0A0M9FY26|A0A0M9FY26_LEPPY    ----------------------------------------MGSAQRAFHAVVVATL--LV    18
tr|A4HBZ4|A4HBZ4_LEIBR            ---MVA------PLSAALALGSPGLRGG-----SFNHTTISSRATTAACRAVLVAV--LI    44
tr|Q4QC59|Q4QC59_LEIMA            -----------------------------------------------MLCTLIAAV--LI    11
tr|A4HZD4|A4HZD4_LEIIN            -----------------------------------------------MLCTLIAAV--LI    11
tr|A0A0S4IMV9|A0A0S4IMV9_BODSA    --------------------------MR--------VLII--AA----LCTIVVMHLSMS    20
tr|A0A0S4IJV6|A0A0S4IJV6_BODSA    ------------------------------------------------------------    0
tr|A0A1X0NHQ9|A0A1X0NHQ9_9TRYP    --------------------------MKC----TFSHVLVT-F-----ISLLFIFGCSLS    24
tr|Q38CG9|Q38CG9_TRYB2            ------------MRSAYCRKPSSGFMAHS----NVVVALLS----------LVALHFVPY    34
tr|K2NQ48|K2NQ48_TRYCR            -----------MQEHPFCVKRAPGMALTHRVHGGLAPLLLFSKA----KRVLWLVL-MVS    44
tr|V5BHB3|V5BHB3_TRYCR            ------------------------MAVTHWVHGGVVPPPLL-MA----KRVLWLVL-MMS    30
tr|Q4E1T8|Q4E1T8_TRYCC            ------------------------MAVTHWVHGGVVPPPLP-MA----KRVLWLVL-MMS    30
tr|Q4DB70|Q4DB70_TRYCC            ------------------------MAVTHWVHGGVVPPPLP-MA----KRVLWLVL-MMS    30
tr|A0A3R7N2P8|A0A3R7N2P8_9TRYP    --------------------------MRREARGGAASLELSSM-----KRPLWLLLLVMS    29
tr|A0A3R7KKV6|A0A3R7KKV6_TRYRA    --------------------------MRRRVCGDAVSLQLPIM-----KRVLWLLL-TLS    28
tr|A0A061IV15|A0A061IV15_TRYRA    MFFFVFFFFCCVEEHRFGLKGRPSRVMRRRVCGDAVSLQLPIM-----KGVLWLLL-ALS    54


tr|A0A0M9FY26|A0A0M9FY26_LEPPY    LLCTA------AGLASAAPSSRPFRCLRPFSAMDSFVAVAKADATDNCMFHYFTVTAAAA    72
tr|A4HBZ4|A4HBZ4_LEIBR            LMVAP------S-GAQGAVMPASFRCLRPFSATDTFSNVAKAQLSQNCDYFYFTVEDAKA    97
tr|Q4QC59|Q4QC59_LEIMA            LVVAP------P-GAQAAASPSSFRCLRPFSASATFPNVAKAHPAQNCDYFYFSVEDAKA    64
tr|A4HZD4|A4HZD4_LEIIN            LVVAP------P-GAQAAAGPSSFRCLRPFSATGTFTNVAKAHPTQNCDYFYFSVEDAKA    64
tr|A0A0S4IMV9|A0A0S4IMV9_BODSA    GVVAASSSSSPAPVNTPSPERHPFRCFRPFSALDKFAP-SSMHRRDNCEFQFVQVQDARV    79
tr|A0A0S4IJV6|A0A0S4IJV6_BODSA    ------------------------------------------------------------    0
tr|A0A1X0NHQ9|A0A1X0NHQ9_9TRYP    LVADAT--NNNNNTTTETAALTPFRCMRPFGSLSRFSP-SAMNAAENCKFGSFRILNASA    81
tr|Q38CG9|Q38CG9_TRYB2            YIQGT------VSADSGSNTTVGFRCMRPFASLPRFSS-TSMDAAENCKFKRYNVLNASA    87
tr|K2NQ48|K2NQ48_TRYCR            FYVDV------ASASDTLKSVAPFRCMRPFESLPRFSS-ANMDAGENCRLGPFRVINAKA    97
tr|V5BHB3|V5BHB3_TRYCR            LYVDA------ASASDTLKGVAPFRCMRPFESLPRFSP-ANMDSGENCRFGPFRVINAKA    83
```

```
tr|Q4E1T8|Q4E1T8_TRYCC            LYVDA------ASASDTLKGVAPFRCMRPFESLPRFSP-ANMDSGENCRLGPFRVINAKA    83
tr|Q4DB70|Q4DB70_TRYCC            LCVDA------ASASDILKGVAPFRCMRPFESLPRFSP-ANMDSGENCRLGPFRVINAKA    83
tr|A0A3R7N2P8|A0A3R7N2P8_9TRYP    FSLGA------ASTSAAVKDAAPFRCMRPFASLPRFSP-ANMDAAENCKFGSLRVINATA    82
tr|A0A3R7KKV6|A0A3R7KKV6_TRYRA    FFLGA------ASSSTAVKDTAPFRCMRPFGSLPRFSP-ANMDAAENCKFGLLRVINATA    81
tr|A0A061IV15|A0A061IV15_TRYRA    FFLGA------ASSSAAVKDTAPFRCMRPFGSLPRFSP-ANMDAAENCKFGLLRVINATA    107


tr|A0A0M9FY26|A0A0M9FY26_LEPPY    ELRCATTADRGTPPLSSQ-----AGEKAAATSCNVTVRWSMRRADDVAAVLRRQIGTDAD    127
tr|A4HBZ4|A4HBZ4_LEIBR            ELKCTSSSAAAT-------------VVSDDISCSVTVRWSMRRASDVTARLQRQVGTDKD    144
tr|Q4QC59|Q4QC59_LEIMA            ELKCASPSAVTT-------------AVSDEMSCNVTVRVSMRRASDAVSHLQRQVGTDED    111
tr|A4HZD4|A4HZD4_LEIIN            ELKCASPSAATT-------------AVSDEMSCNVTVRMSMRRASDVVPRLQRQVGTDKD    111
tr|A0A0S4IMV9|A0A0S4IMV9_BODSA    QVECEKGTGDTT-----------NENEGGVGGCYATLALDLSVTGQKLIEHE---L----    121
tr|A0A0S4IJV6|A0A0S4IJV6_BODSA    ---------------------------------------------------E---L----    2
tr|A0A1X0NHQ9|A0A1X0NHQ9_9TRYP    VLNCTHAKSTTMSNIDGQSTQAEENDKMLVPGCNAVIRIVFKMEEENKFGT---------    132
tr|Q38CG9|Q38CG9_TRYB2            VVKCESTGNAT-----GK-----AEPTAVTSTCSVTIHAAFRTMGGGKDDN---------    128
tr|K2NQ48|K2NQ48_TRYCR            MTQCVREYDDE-----DD------TARATSPTCSVTLRVTFQTEGDKKRHHA---G----    139
tr|V5BHB3|V5BHB3_TRYCR            MTQCVRD---D-----DD------TARAASPTCSVTLRVSFQTEGDKRRHRV---G----    122
tr|Q4E1T8|Q4E1T8_TRYCC            MTQCVRD---D-----DD------TTRAASPTCSVTLRVSFQTEGDKRRHRV---G----    122
tr|Q4DB70|Q4DB70_TRYCC            MTQCVRD---D-----DD------TARAASPTCSVTLRVSFQTEGDKRRHRV---G----    122
tr|A0A3R7N2P8|A0A3R7N2P8_9TRYP    RTQCLREDAAR-----GAS--TASEGEKPPPTCSVTLHLTFQTVGDEVDRPV---G----    128
tr|A0A3R7KKV6|A0A3R7KKV6_TRYRA    RTRCVRDDAAR-----GAS--TASNNDKMPPTCSVTLNVTFQAEGNEAYHAV---G----    127
tr|A0A061IV15|A0A061IV15_TRYRA    RTRCVRDDAAR-----GAS--TASNDDKQPPTCSVTLNVTFQAEGNEVYHAV---G----    153


tr|A0A0M9FY26|A0A0M9FY26_LEPPY    YFAANTAELSASSARTAQHSPQQQQQKKRSRAPTELFLTEDEDVWRYAVSLCAKGDSTVQ    187
tr|A4HBZ4|A4HBZ4_LEIBR            YFVHDASTASTATLPT---------RTRNTHQSARVFLTEDDDVWRYTVSLKAKGDSSIH    195
tr|Q4QC59|Q4QC59_LEIMA            YFTHEASTVSTTTSPT---------RSGNTCPSAAPFVTEDEDVWRYAVSLKAKGDSSIH    162
tr|A4HZD4|A4HZD4_LEIIN            YFTHEASTDSTTASPT---------RVGNTRPSAVPFLTEDEDVWRYAVSLKAKGDSSIH    162
tr|A0A0S4IMV9|A0A0S4IMV9_BODSA    --------------PQDSH------------EDVVSGRVRAHYHPYYTLELHALNHSKIT    155
tr|A0A0S4IJV6|A0A0S4IJV6_BODSA    --------------PQDSH------------EDVVSGRVRAHYHPYYTLELHALNHSKIT    36
tr|A0A1X0NHQ9|A0A1X0NHQ9_9TRYP    ---------------TESN------------NGLVE-----NDIYRFSLQLRAFNDSVVH    160
tr|Q38CG9|Q38CG9_TRYB2            ---------------------------------ADA-----ADVYRYSLQLRAINNSVVH    150
tr|K2NQ48|K2NQ48_TRYCR            ------------GSTVAGS------------EETFG-----SEAYRFSLRLRAFNDSVVE    170
tr|V5BHB3|V5BHB3_TRYCR            ------------GAALAGS------------DEAFG-----SEAYRFSLRLRAFNDSIVE    153
tr|Q4E1T8|Q4E1T8_TRYCC            ------------GAALAGS------------DEAFG-----SGAYRFSLRLRAFNDSIVE    153
tr|Q4DB70|Q4DB70_TRYCC            ------------GAALAGS------------DEAFG-----SEAYRFSLRLRAFNDSIVE    153
tr|A0A3R7N2P8|A0A3R7N2P8_9TRYP    ------------VSSVAGH------------RPAVG-----GEAYRFSLRLRAFNDSVVE    159
tr|A0A3R7KKV6|A0A3R7KKV6_TRYRA    ------------VSSVAGH------------EEAFG-----GEAYRFSLRLRAFNDSVVE    158
tr|A0A061IV15|A0A061IV15_TRYRA    ------------VSSVAGH------------KEAFG-----GEAYRFSLRLRAFNDSVVE    184
                                                                                      ::: * * ..* :

tr|A0A0M9FY26|A0A0M9FY26_LEPPY    YKGYNGGDGYSMCCDGIVE--GECAWMAAEQG-----DNDLA--------ADEEVNPDFA    232
tr|A4HBZ4|A4HBZ4_LEIBR            YKGFSKGNGYSLCCNALEE--ADCAWMTLEKG-----DNDLD--------CDEAIGSAFA    240
tr|Q4QC59|Q4QC59_LEIMA            YKGFNGGNGYSLCCAALEE--ADCAWITPQKG-----DKDLD--------CDEDVDVGFA    207
tr|A4HZD4|A4HZD4_LEIIN            YKGFNGGNGYSLCCAALEE--ADCAWMTPQKG-----DKDLD--------CDEDVDVRFA    207
tr|A0A0S4IMV9|A0A0S4IMV9_BODSA    YKGFD-GLGYDLCCDFLNESSAHCEWEDTKNT-VAGGDE-SSSSSLPPSEQEDAATQQQR    212
tr|A0A0S4IJV6|A0A0S4IJV6_BODSA    YKGFD-GLGYDLCCDFLNASSAHCEWEDTKKDTVAGGDESSSSSSLPPSEQEDAATQQQR    95
tr|A0A1X0NHQ9|A0A1X0NHQ9_9TRYP    YKGFD-QNGYSMCCDLIQE--GECVWEKNGNLSQTEGKENISKN--------TETSEITT    209
tr|Q38CG9|Q38CG9_TRYB2            YKGFD-QNGYGLCCSFIEG--AKCTWEERETSGVQEA-----------KQEVNATTSQAP    196
tr|K2NQ48|K2NQ48_TRYCR            YKGFD-QNGYSMCCQFIEG--SECSWEMKEKEKEEEGANSTQQESATSQEGDDGRMSHAP    227
tr|V5BHB3|V5BHB3_TRYCR            YKGFD-QNGYSMCCHFIEG--SECSWDVKEEE-----GNSTQQEAATPQKDDDGRASHAP    205
tr|Q4E1T8|Q4E1T8_TRYCC            YKGFD-QNGYSMCCHFIEG--SECSWDVKEEE-----GNSTQQEAATPQKDDDGRASHAP    205
tr|Q4DB70|Q4DB70_TRYCC            YKGFD-QNGYSMCCHFIEG--SECSWEVKEEE-----GNSTQQEAATPQKEDDGRASHAP    205
tr|A0A3R7N2P8|A0A3R7N2P8_9TRYP    YKGFD-QNGYSMCCDLIQG--AECAWAGEGG------SSAAAHSPAVTQEGDGGSTPDAP    210
tr|A0A3R7KKV6|A0A3R7KKV6_TRYRA    YKGFD-QNGYSMCCNFIQS--AECSWDEERK------SSGTPHYSASPQEDNGGNAPDAS    209
tr|A0A061IV15|A0A061IV15_TRYRA    YKGFD-QNGYSMCCNFIQG--AECSWDEERK------SSDTPHYSASPQEDDGGNAPDAA    235
                                  ***:.   **.:**  :..* *

tr|A0A0M9FY26|A0A0M9FY26_LEPPY    LQHRRMLRGCPLPFPATMPPTAAAEEADDNEADGAFGAAPGARVALTDLDEEDAAEGREE    292
tr|A4HBZ4|A4HBZ4_LEIBR            SQSTRTLRSCPLPLPARRCA--------AGDVDDDCGAWAGAGVVLTDLDE-----DEDS    287
tr|Q4QC59|Q4QC59_LEIMA            SQRSRVLRGCPLPFPAPCSA--------GGGV---FDAPAGARVLLTDLDE-----GESS    251
tr|A4HZD4|A4HZD4_LEIIN            PQRGRVLRGCPLPFPAPRSA--------GDGV---FGASAGARVLLTDLDE-----GEGS    251
tr|A0A0S4IMV9|A0A0S4IMV9_BODSA    IPRRQRLHHCAVTNPLTP----------------------------------------SG    232
tr|A0A0S4IJV6|A0A0S4IJV6_BODSA    IPRGQRLHHCAVTNPLTP----------------------------------------SG    115
tr|A0A1X0NHQ9|A0A1X0NHQ9_9TRYP    PQREPVVVSCPLYNKKIA----------------------------------------KG    229
tr|Q38CG9|Q38CG9_TRYB2            TVKEPAVVSCPLHHG-EE----------------------------------------EH    215
tr|K2NQ48|K2NQ48_TRYCR            QRREAMVVSCPIRNP-VV----------------------------------------PG    246
tr|V5BHB3|V5BHB3_TRYCR            RRREAMVVSCPIRNP-VV----------------------------------------PG    224
tr|Q4E1T8|Q4E1T8_TRYCC            RRREAMVVSCPIRNP-VV----------------------------------------AG    224
tr|Q4DB70|Q4DB70_TRYCC            RRREAMVVSCPIRNP-VV----------------------------------------PG    224
tr|A0A3R7N2P8|A0A3R7N2P8_9TRYP    RRREAVIVSCPLRSP-VV----------------------------------------PG    229
tr|A0A3R7KKV6|A0A3R7KKV6_TRYRA    RRREAMVVSCPIHSQ-VV----------------------------------------PG    228
tr|A0A061IV15|A0A061IV15_TRYRA    RRREAMVVTCPIGGQ-VV----------------------------------------PG    254
                                           :  * :

tr|A0A0M9FY26|A0A0M9FY26_LEPPY    GVFHGQITKPLHRLVEGPWEVTLQMWRRRQRRPTHAAATPSSSSVPTDSSAEAEVLGRVV    352
tr|A4HBZ4|A4HBZ4_LEIBR            GVFHGVITKALHRIVEGPWEMVVQMWRRRQWIPSNGA--EASYTIPTDEGVEAEVLGRIV    345
tr|Q4QC59|Q4QC59_LEIMA            GVFHAVITKPLHRIVEGPWEVMVQMWRRRQQMPRGGR--EDSFSVPADHSIEAEVLGRIM    309
tr|A4HZD4|A4HZD4_LEIIN            GVFHAVITKPLHRFVEGPWEVMVQMWRRRQHMPRVGR--EDSLSVPADHSIEAEVLGRIM    309
tr|A0A0S4IMV9|A0A0S4IMV9_BODSA    RIVSGSIRKPLHKIVAGDWEVRWTLRRGG------------------------ETVGRLT    268
tr|A0A0S4IJV6|A0A0S4IJV6_BODSA    RIVSGSIRKPLHKIVAGDWEVRWTLRRGS------------------------ETVGRLI    151
tr|A0A1X0NHQ9|A0A1X0NHQ9_9TRYP    NVFHGSLIKPLHRLAVTEWEARLEFWRGVK--------------------NEREVLGRLL    269
tr|Q38CG9|Q38CG9_TRYB2            IAFRGSTVKPLHRLVVGNWEARLEFWRGGP--------------------SNREVLGRLL    255
tr|K2NQ48|K2NQ48_TRYCR            IVFHGAITKPLHRLVITEWEARLELWRGRA--------------------HEKVFLGRVL    286
tr|V5BHB3|V5BHB3_TRYCR            IVFHGAITKPLYRLVITDWEARLELWRGRP--------------------HEKVFLGRVL    264
tr|Q4E1T8|Q4E1T8_TRYCC            IVFHGAITKPLHRLVITEWEARLELWRGRP--------------------HEKVFLGRVL    264
tr|Q4DB70|Q4DB70_TRYCC            IVFHGAITKPLHRLVITDWEARLELWRGRP--------------------HEKVFLGRVL    264
```

```
tr|A0A3R7N2P8|A0A3R7N2P8_9TRYP    VVFRGVVRKPLHRLLLTEWEARLEFWRGRR--------------------EERETLGRVL    269
tr|A0A3R7KKV6|A0A3R7KKV6_TRYRA    GVFHGVITKPLHRLVVTEWEARLEFWRGQR--------------------QERVMLGRVL    268
tr|A0A061IV15|A0A061IV15_TRYRA    DVFHGVITKPLHRLVVTEWEARLEFWRSQR--------------------EEREMLGRVL    294
                                  . .   * *:::     **       : *                          :**:

tr|A0A0M9FY26|A0A0M9FY26_LEPPY    VPFTVHLAELQREGRVXXXXXXXXXXX-------XXXXXXXXXXXXXXXXXXXXXXXXXX    405
tr|A4HBZ4|A4HBZ4_LEIBR            VPFTLNVADLQGQGRITYVPSMALAVE-------DIAEVVMVTDVKPAVEA---------    389
tr|Q4QC59|Q4QC59_LEIMA            VPFTLNLTELQKQALITQIPSMALTAE-------DVAGVAQAAGENAADET---------    353
tr|A4HZD4|A4HZD4_LEIIN            VPFTLNLTELQKQGRITQMPSMALTVE-------DVADVAQAAGEKAADEA---------    353
tr|A0A0S4IMV9|A0A0S4IMV9_BODSA    VPFHITDEILSGGGTPQQQGDTSAGAA--ATAAASLADSPTTVGTAVV-TVKE-------    318
tr|A0A0S4IJV6|A0A0S4IJV6_BODSA    VPFHITDEMLSGGTSQQVDTSASSAAA--PAASAPLVDSPT-TGTAVV-TVEE-------    200
tr|A0A1X0NHQ9|A0A1X0NHQ9_9TRYP    LPFKLTESDLAFLNESNRDRGTTGVSTNSWNNVGALV-EGKKEG----------------    312
tr|Q38CG9|Q38CG9_TRYB2            VPFRLTDENIAQGNNNTITERSDHTTA-VVVVAEQ----------------GQ-------    291
tr|K2NQ48|K2NQ48_TRYCR            VPFRLTEDDIASAQSNAAAKTTTTTST-AMTATESSK-SGKMSGGVLV-VTED-------    336
tr|V5BHB3|V5BHB3_TRYCR            VPFRLTEDDIASAQSNAAATTT---TT-AMTVTDSSA-SSKMAGGVLL-ATED-------    311
tr|Q4E1T8|Q4E1T8_TRYCC            VPFRLTEDDIASAQSNAAATTT---TT-AMTATDSSA-SGKMAGGVLL-VTED-------    311
tr|Q4DB70|Q4DB70_TRYCC            VPFRLTEDDIASAQFNAAATTT---TT-AMTATDSSA-SGKMAGGVLL-VTED-------    311
tr|A0A3R7N2P8|A0A3R7N2P8_9TRYP    VPFRLTEEDVASAYAADAAATT---SR-AVTAIDDSP-SGEQDVVAPL-ETGE-------    316
tr|A0A3R7KKV6|A0A3R7KKV6_TRYRA    LPFRLTEDDIASAYTSDAAATT-LTAT-AVAAIDDST-SGGRAGAAPI-ETGD-------    317
tr|A0A061IV15|A0A061IV15_TRYRA    LPFRLTEDDIASAYTSDAAATT-LTAT-AVAAIDDST-SGGRAGAAPT-ETGD-------    343
                                  :** :    :

tr|A0A0M9FY26|A0A0M9FY26_LEPPY    XXXXXXXXXXXXXXXXXXXXXXXXXXXXXXXXXXXXXXXLEEDVVEVAPAPAPPSSVDAG    465
tr|A4HBZ4|A4HBZ4_LEIBR            ------------------------------------------------------------    389
tr|Q4QC59|Q4QC59_LEIMA            ------------------------------------------------------------    353
tr|A4HZD4|A4HZD4_LEIIN            ------------------------------------------------------------    353
tr|A0A0S4IMV9|A0A0S4IMV9_BODSA    ---------------------------------------------------------QQR    321
tr|A0A0S4IJV6|A0A0S4IJV6_BODSA    ---------------------------------------------------------QR-    202
tr|A0A1X0NHQ9|A0A1X0NHQ9_9TRYP    ----------------------------------------------------------VK    314
tr|Q38CG9|Q38CG9_TRYB2            ---------------------------------------------------------LTG    294
tr|K2NQ48|K2NQ48_TRYCR            ---------------------------------------------------------VIK    339
tr|V5BHB3|V5BHB3_TRYCR            ---------------------------------------------------------VVK    314
tr|Q4E1T8|Q4E1T8_TRYCC            ---------------------------------------------------------VVK    314
tr|Q4DB70|Q4DB70_TRYCC            ---------------------------------------------------------VVK    314
tr|A0A3R7N2P8|A0A3R7N2P8_9TRYP    ---------------------------------------------------------NVG    319
tr|A0A3R7KKV6|A0A3R7KKV6_TRYRA    ---------------------------------------------------------VIG    320
tr|A0A061IV15|A0A061IV15_TRYRA    ---------------------------------------------------------VIG    346


tr|A0A0M9FY26|A0A0M9FY26_LEPPY    EAEDTGDL----------    473
tr|A4HBZ4|A4HBZ4_LEIBR            -----GDL----------    392
tr|Q4QC59|Q4QC59_LEIMA            -----GDL----------    356
tr|A4HZD4|A4HZD4_LEIIN            -----GDL----------    356
tr|A0A0S4IMV9|A0A0S4IMV9_BODSA    EHEQGDKVTHAPAKVEEL    339
tr|A0A0S4IJV6|A0A0S4IJV6_BODSA    -EHGSDKVTHAPAKVEEL    219
tr|A0A1X0NHQ9|A0A1X0NHQ9_9TRYP    EFNREDDL----------    322
tr|Q38CG9|Q38CG9_TRYB2            SRG---DL----------    299
tr|K2NQ48|K2NQ48_TRYCR            ERNGSEDL----------    347
tr|V5BHB3|V5BHB3_TRYCR            ERNGYEDL----------    322
tr|Q4E1T8|Q4E1T8_TRYCC            ERNGYEDL----------    322
tr|Q4DB70|Q4DB70_TRYCC            ERNGYEEL----------    322
tr|A0A3R7N2P8|A0A3R7N2P8_9TRYP    ERKGSADL----------    327
tr|A0A3R7KKV6|A0A3R7KKV6_TRYRA    KRKGSGDL----------    328
tr|A0A061IV15|A0A061IV15_TRYRA    KRKGSGDL----------    354
                                          .:
```

(overall sequence identity = 0.0395)

08. ER-implicated kinetoplastid ATPase (ERIKA)

These proteins belong to the greater Torsin (ClpA/ClpB) family of chaperone proteins. They are typically found at the nuclear membrane lumen as well as the ER, but usually have no KDEL signals. All parazitic kinetoplastids seem to have multiple torsin paralogues, and only one subtype (that we now cover under the acronym ERIKA) has the typical KDEL-like signal. While the KDEL-like signal is seen in Bodo saltans, it is not yet found in the early-branching kinetoplastid Perkinsela sp. The only other eukaryotic torsin protein with a clear KDEL-like retention signal was detected in the unicellular amoeba Acanthamoeba castellanii (the motif probably evolved independently).

```
tr|D7G3V5|D7G3V5_ECTSI            -------------------------------------------MAMIVSQAQMA------    11
tr|Q55FE2|Q55FE2_DICDI            -----------------------------MGRYFYLQLLFISILFLK--SYSAQN-N---    25
tr|L8GYA8|L8GYA8_ACACA            ------------------------MMQARL----RSLVLA---LVLLPALVMAA------    23
tr|A0A0L1L0A3|A0A0L1L0A3_9EUGL    ----MP------------------LPAFSMKAFILNLSLL---MTLSITVRSSNEDATRI    35
tr|A0A0S4KNW8|A0A0S4KNW8_BODSA    ------------------------MLAHK---GMLVATVA---VCMCI--LA--------    20
tr|A0A422N6P1|A0A422N6P1_TRYRA    ------------------------MGSCATA-TWVLAFLVVV-VAALTPWVA--------    26
tr|A0A3R7NQX6|A0A3R7NQX6_9TRYP    ----MP-----------------QMGPCATS-TCLLALLAAV-VAALVPWVA--------    29
tr|S9U3X1|S9U3X1_9TRYP            ------------------------------------------------------------    0
```

```
tr|S9U3J6|S9U3J6_9TRYP          ------------------------MNVGR-----TGAAFVVFLLALCVYLYG--------    23
tr|A0A0N0VF20|A0A0N0VF20_LEPPY  ------------------------MRSFL---ITALSIVCVCLACI---VYV--------    22
tr|A4I131|A4I131_LEIIN          ------------------------MHVFT----RILAVFVIVLAALLIPQFL--------    24
tr|Q4QAA8|Q4QAA8_LEIMA          ------------------------MHLFT----RILAVFVVVLVALLIPRFL--------    24
tr|A0A7G2CLT7|A0A7G2CLT7_9TRYP  ----MT------HT-RPFRV----------WRFSILLLLVSS---VLVSVRA--------    28
tr|S9VXS8|S9VXS8_9TRYP          ----MP-----------------RRPRVTHCGFALVLVLCMGVLAVAISLTAAA--AA--    35
tr|A0A0M9FWU4|A0A0M9FWU4_LEPPY  ----MP-----CHARREKRRAVPQLSYSSTV---RIAVLSLSLLVLLLSCVALP------    42
tr|A4H5R3|A4H5R3_LEIBR          MASFLPPQRQLSHISRRQPGWIPPSATAGKT---ALLILFAAAFFLALGSATAA--EGQQ    55
tr|Q4QHU2|Q4QHU2_LEIMA          -----------------------------MT---VILVVCTVVFVLVLGSATVA--DGRP    26
tr|A4HU03|A4HU03_LEIIN          -----------------------------MT---VALIVCSVVFVLVLGSATVA--GGRP    26
tr|Q382P2|Q382P2_TRYB2          ----MP-HSGYPFG-SSSVVGVEGDDRHSSVSLFVILFLCVLLTAVEPSVA---------    45
tr|A0A1X0NMJ2|A0A1X0NMJ2_9TRYP  --MIFF-SR------RSSS-----------III-AV--IFVVVLLALPTCLA--------    29
tr|Q4D6G4|Q4D6G4_TRYCC          ----MS-AR------RLFTRGIP------VATMAIA--VCIVIFLIFPA-----------    30
tr|Q4D903|Q4D903_TRYCC          ----MS-AR------RLFTRGIP------VATMAIA--VCIVIFLIFPA-----------    30
tr|A0A422ND04|A0A422ND04_9TRYP  ----MP-AR------RHSPRGVS------AVATVVA--ALILLLLIFPA-----------    30
tr|A0A3R7RCD3|A0A3R7RCD3_TRYRA  ----MF-AR------CRSLRGAP------AVTTLVA--ALTVLLLVFPA-----------    30


tr|D7G3V5|D7G3V5_ECTSI          ------ASVEE---------EPAAT-ATSAATI--SVLDA-------VRVMGSLTWWGVD    46
tr|Q55FE2|Q55FE2_DICDI          --DKIKNDD-------DSIYVNKIFGDCKEIYYNNKLNNDENYKNYIGSLFGTYFNNNNN    76
tr|L8GYA8|L8GYA8_ACACA          ------FDDCECNESWTSFACTRCFGPCPRTLE--PV-KRFEELTTK-------------    61
tr|A0A0L1L0A3|A0A0L1L0A3_9EUGL  NDDLTLLSYSNQTIYANKRICPCRP-LCSRGTG--FI-N--PICWGIPSLRY-RYQWSND    88
tr|A0A0S4KNW8|A0A0S4KNW8_BODSA  ----------EMVLGWSPSECPPPP-LCEGRNA--SY-QQQAWCQLGRDPLS--------    58
tr|A0A422N6P1|A0A422N6P1_TRYRA  ---R------VELHPGEAGDCTLRP-LCSSHP---TL-VKRLMCFLFQYPVD--------    64
tr|A0A3R7NQX6|A0A3R7NQX6_9TRYP  ---H------VELHPGEADDCMLRP-LCSSHP---TL-VKGLMCLLFRYPVD--------    67
tr|S9U3X1|S9U3X1_9TRYP          ---------------------MDVP-MCSSPSL--TA-LGHLYCTLAPTSLL--------    27
tr|S9U3J6|S9U3J6_9TRYP          --ASNTFSEEALLYRSPVQDCMDVP-MCSSPSL--TA-LGHLYCTLAPTSLL--------    69
tr|A0A0N0VF20|A0A0N0VF20_LEPPY  ----ETV---GSRLDCTALYCPAPP-LCDQATR--TF-SQRVVCGLGKSPLK--------    63
tr|A4I131|A4I131_LEIIN          --EPIYV---IPAADAVAQFCSEPP-LCARKDR--TM-KQQLLCTIRSAPLK--------    67
tr|Q4QAA8|Q4QAA8_LEIMA          --EPIYV---IPAADAVAPFCSEPP-LCARKDR--TM-KQQLLCTIRSAPLK--------    67
tr|A0A7G2CLT7|A0A7G2CLT7_9TRYP  ------APASHRERILASQACPAPP-LCEGSRL---SAAHIKYCNPLSATAVRFFRFTTN    78
tr|S9VXS8|S9VXS8_9TRYP          --EDTSEHVPHYGGGARRSLCPAGP-ICSRAHT--PFALKFKYCNKVTSFFTSFFGGGGG    90
tr|A0A0M9FWU4|A0A0M9FWU4_LEPPY  --QLARASPTVSTITSTSSLCPRCP-LCARRQT--SLRDRLKYCHSTSSYALSLFSSGGS    97
tr|A4H5R3|A4H5R3_LEIBR          QQRPLKATAAGASSHVSNSLCPRCP-LCSRPGT--PMKDWLKYCHTTTGYIFSLFSWGKS    112
tr|Q4QHU2|Q4QHU2_LEIMA          KQPISKGNVAEASSHASSSLCPRCP-LCSRPGT--PIKERLKYCHATSGYIFSLFSWGTS    83
tr|A4HU03|A4HU03_LEIIN          KQPISKGTTAGASSHASSSLCPRCP-LCSRSGT--PIKERLKYCHTTSGYIISLFSWGTS    83
tr|Q382P2|Q382P2_TRYB2          -GADVRSTVDEVVADTSEPTCPPAP-VCSRYKW---LSSSQLYCWMSKLP----------    90
tr|A0A1X0NMJ2|A0A1X0NMJ2_9TRYP  LQGKEQDKHFSSETQLREWKCPSSP-FCSKGNA--PLWTKLKYCLFSSIP----------    76
tr|Q4D6G4|Q4D6G4_TRYCC          --SSLRPLPVAAEGEEVERRCPRCP-ICSARGT--PFFLRLRYCVLTSLP----------    75
tr|Q4D903|Q4D903_TRYCC          --SSLRPLPVAAEGEEVERHCPRCP-ICSARGT--PFFLRLRYCVLTSLP----------    75
tr|A0A422ND04|A0A422ND04_9TRYP  --SPLRPSSVAAEVELVEWRCPRCP-ICAARGT--PLLLRLRHCVLTALP----------    75
tr|A0A3R7RCD3|A0A3R7RCD3_TRYRA  --SPLRPSFVSAEGEVVEWHCPRCP-ICAVRGT--PLLLQLRYCVLTSLP----------    75


tr|D7G3V5|D7G3V5_ECTSI          LAMET------------AGYGVCPFADDPVEA--ARRALQDGILAQPLALESLDGALSSW    92
tr|Q55FE2|Q55FE2_DICDI          KDNNNNN-------------NNCKFKKD---KNELLKSFKQYFEGFVSFQDDAIKEIESI    120
tr|L8GYA8|L8GYA8_ACACA          ----------------------------------------PGPAKKLMGQERAAVLIADA    81
tr|A0A0L1L0A3|A0A0L1L0A3_9EUGL  NIG------------------------------DIHSCLYSLFMQIKGQQRAIETLVGH    117
tr|A0A0S4KNW8|A0A0S4KNW8_BODSA  -------------------------HNSVEYIRMVQNRLSYFLSRSLLGQDHLAPRILAD    93
tr|A0A422N6P1|A0A422N6P1_TRYRA  -------------------------VDRSDYKQQVLVRLRRLTQRNLKGQTHVVEGVIRG    99
tr|A0A3R7NQX6|A0A3R7NQX6_9TRYP  -------------------------VDRGDYKQQVLVRLRRLTQRSLKGQRHVVEGVTRS    102
tr|S9U3X1|S9U3X1_9TRYP          --------------------------SRPGYADYVYQQLRARAHHSVKGQYI-IDPVLQL    60
tr|S9U3J6|S9U3J6_9TRYP          --------------------------SRPGYADYVYQQLRARAHHSVKGQYI-IDPVLQL    102
tr|A0A0N0VF20|A0A0N0VF20_LEPPY  -------------------------PGSKEFRAYVVTRLHGFVKESLKGQLM-APAVVEM    97
tr|A4I131|A4I131_LEIIN          -------------------------PASPVYRQYVVSRLKRYVDGSIRGQSV-APSVIER    101
tr|Q4QAA8|Q4QAA8_LEIMA          -------------------------PASPAYRQYVVSRLKRYVDGSIRGQSV-AASVIER    101
tr|A0A7G2CLT7|A0A7G2CLT7_9TRYP  KLSSAVSHAPH--TKTEYRPLQLPFPTDAVIRKNAKRNVEEYLKNYLAGQEHLLSPITRL    136
tr|S9VXS8|S9VXS8_9TRYP          ----------GGKIDFRHFPLQLPFATDTAMRQLAKAGLEHLLLMRLIGQDHLQQELLDV    140
tr|A0A0M9FWU4|A0A0M9FWU4_LEPPY  ELATATAAGASYREQRHRYPLELPFATDDAVRAQCVRDATDLLASRVRGQPQVVKPLLEV    157
tr|A4H5R3|A4H5R3_LEIBR          TLAGTQRTGGSIREQRQLYPLELPFATDDAVRRQCMRDLQDLLLARVRGQPQVVIPLLDV    172
tr|Q4QHU2|Q4QHU2_LEIMA          ALAGKQRTGVSNREQRQLYPLELPFATDDAVRRQCMRDMQDLLLARVRGQPQVVMPLLDV    143
tr|A4HU03|A4HU03_LEIIN          ALAGKQRTGVSNREQRQLYPLELPFATDDAVRKQCMRDMQDLLLARVRGQPQVVMPLLDV    143
tr|Q382P2|Q382P2_TRYB2          ---------------SRLVSRELPFATDDKARRLAISNVEELLRTRLVGQGHLTEAIASL    135
tr|A0A1X0NMJ2|A0A1X0NMJ2_9TRYP  ---------------LAKIRQELPFATSDAARRIAMENVEELLFTHVVGQDHVTHAIAEA    121
tr|Q4D6G4|Q4D6G4_TRYCC          ---------------AVLVKREFSVEKDEKLRHRVMEDVKELLLTRLVGQEHVKGAILEA    120
tr|Q4D903|Q4D903_TRYCC          ---------------AVLVKREFSVEKDEKLRHRVMEDVKELLLTRLVGQEHLKGVILEA    120
tr|A0A422ND04|A0A422ND04_9TRYP  ---------------AALVRREFALAKDEALRQRVMKDVEELLLTRVAGQEHVKGAILEA    120
tr|A0A3R7RCD3|A0A3R7RCD3_TRYRA  ---------------AALVRQEFAFSKQEALRHRVMNDVEELLFTRVAGQEHVKKAILEA    120
                                                                                            :

tr|D7G3V5|D7G3V5_ECTSI          HYSRQSDRYEPLVVALTGSTGTGKTETAWVLADALLTKRCRITGG---TRDIPRGLLVLN    149
tr|Q55FE2|Q55FE2_DICDI          IYRKYLHPDTKVVMHLFGDHGIGKTLSSKLVSRVLF-ENGS--------GLEGDGLLLIN    171
tr|L8GYA8|L8GYA8_ACACA          LR--AASGKKPLTFHFCGENGVGKSHTALLLAEAYF-AYKDK------KTDMYKGLLWIS    132
tr|A0A0L1L0A3|A0A0L1L0A3_9EUGL  INRKLYDPRASFLIHLAGDNGVGKTMSAALLSIAIS-LYPHKAH-----RNAGETLLIIS    171
tr|A0A0S4KNW8|A0A0S4KNW8_BODSA  VIHKLRRPNEPLILHFAGDNGVGKTSTAQLISASMS-FRCHRSN-TGYYCGLGDASLSLS    151
tr|A0A422N6P1|A0A422N6P1_TRYRA  IAAKLENPDKPLVLHFAGDNGVGKTTLAQLISLALG-LRCHD-----AACTTGDSALVLS    153
tr|A0A3R7NQX6|A0A3R7NQX6_9TRYP  LAAKLENPDKPLVLHFAGDNGVGKTTLAQLISLALG-LRCRD-----AACTTGDSTLVLS    156
tr|S9U3X1|S9U3X1_9TRYP          VKHKLRHRDHPLILHFAGDNGVGKTSLAEVISLAMA-LRCKSA----EDCHIGDSTLELS    115
tr|S9U3J6|S9U3J6_9TRYP          VKHKLRHRDHPLILHFAGDNGVGKTSLAEVISLAMA-LRCKSA----EDCHIGDSTLELS    157
tr|A0A0N0VF20|A0A0N0VF20_LEPPY  VEYKLHHPYEPMVLHFAGDNGVGKTRLAELISLAYG-QRCGD-----ELCTVGDSTLVLS    151
tr|A4I131|A4I131_LEIIN          VRYKLAHMHEPMILHFAGDNGVGKTRLAELISLAIG-QKCGD-----ATCSIGDTTLVLS    155
tr|Q4QAA8|Q4QAA8_LEIMA          VRYKLAHVHEPMVLHFAGDNGVGKTRLAELISLAMG-QKCGD-----ATCSIGDTTLVLS    155
tr|A0A7G2CLT7|A0A7G2CLT7_9TRYP  VQYKVDHPTEPLVLHLAGDNGVGKTFTAKLISLALS-LYCGAEGRDTLPCAHGDALLIVA    195
tr|S9VXS8|S9VXS8_9TRYP          LFKKLYFPATPLVLHLAGDNGVGKSYTAKLISLALS-LRCGADGDG--GCEAGDALLTIS    197
tr|A0A0M9FWU4|A0A0M9FWU4_LEPPY  LRRKLAFPREPVVVHLAGDNGVGKTHTARLVSQALS-LRCAADR-D--VCDAGDNLLVIA    213
tr|A4H5R3|A4H5R3_LEIBR          LRHKLAYPREPVVVHLAGDNGVGKSHTARLVSEALS-LRCALDR-D--VCDAGDNLLIIS    228
tr|Q4QHU2|Q4QHU2_LEIMA          LRRKLAYPREPVVVHLAGDNGVGKTHTARLVSQALS-LRCAPDR-D--VCDAGDNLLTIA    199
```

```
tr|A4HU03|A4HU03_LEIIN              LRRKLAYPREPVVVHLAGDNGVGKTHTARLVSQALS-LRCAPDR-D--VCDAGDNLLTIA    199
tr|Q382P2|Q382P2_TRYB2              MRKKLSYPHEPLVLHFAGDNGVGKTHTARLLSLATS-LRCANSRGR--QCDSGDNMLVIS    192
tr|A0A1X0NMJ2|A0A1X0NMJ2_9TRYP      IRQKMASPGDPLVLHFAGDNGVGKTHTARLLSLALS-LRCAQAR-P--QCDVGDNMLVIS    177
tr|Q4D6G4|Q4D6G4_TRYCC              VRRKLIYPRDPLVLHFAGDNGVGKTHTARLLSLATS-PHCAPSR-P--ACDMGENMLVIS    176
tr|Q4D903|Q4D903_TRYCC              VRRKLIYPRDPLVLHFAGDNGVGKTHTARLLSLATS-PHCAPSR-P--ACDMGENMLVIS    176
tr|A0A422ND04|A0A422ND04_9TRYP      VRRKLSYPQDPLVLHFAGDNGVGKTHTARLLSLATS-LRCAPSR-P--ACDMGDNMLVIS    176
tr|A0A3R7RCD3|A0A3R7RCD3_TRYRA      VRRKLSYPEDPLVLHFAGDNGVGKTHTARLLSLATS-LRCAPSR-P--MCDAGDNMLIIS    176
                                                     . . : *. * **: : :::                       * :

tr|D7G3V5|D7G3V5_ECTSI              GADYMVAAKVEEY---------------------Q---SLIRRKLGQRLEYCGGNVVVLF    185
tr|Q55FE2|Q55FE2_DICDI              GEEFRIIEQQPPTNNNDNNENTKIEDQEYLNIKIQHLRDKLYNTIINKLIECPY-SVIVF    230
tr|L8GYA8|L8GYA8_ACACA              GKQYQMAKSEEEI---------------------KAAREYIHEQIIDHLATCPQ-AIIVI    170
tr|A0A0L1L0A3|A0A0L1L0A3_9EUGL      GSEFASVSGDSAEE-------------------RQLVTRQIQSRLVTHVQKFSK-CVVLF    211
tr|A0A0S4KNW8|A0A0S4KNW8_BODSA      GINYHGVSPEEFR---------------------K----SIVPQVLAFAERHPH-GLVIF    185
tr|A0A422N6P1|A0A422N6P1_TRYRA      GVSYDGYSVQEFR---------------------R----VVVQRIVQHVLRFPKNGVVIV    188
tr|A0A3R7NQX6|A0A3R7NQX6_9TRYP      GVSYDGYSAQEFR---------------------R----VVVPRIVQHALRFPKNGVVIV    191
tr|S9U3X1|S9U3X1_9TRYP              GAAYDALTVAEFR---------------------R----FVVGAVTSHLQRHPRNAVIII    150
tr|S9U3J6|S9U3J6_9TRYP              GAAYDALTVAEFR---------------------R----FVVGAVTSHLQRHPRNAVIII    192
tr|A0A0N0VF20|A0A0N0VF20_LEPPY      GTGYDGLSTAEFR---------------------K----AVVELVTRHARRHPRDGVVVI    186
tr|A4I131|A4I131_LEIIN              GTSYDGMTVAEFR---------------------N----AVVPVVVRHAQRYPDNGVVIF    190
tr|Q4QAA8|Q4QAA8_LEIMA              GTSYDGMTVAEFR---------------------N----AVVPVVVRHAQRYPDNGVVIF    190
tr|A0A7G2CLT7|A0A7G2CLT7_9TRYP      CSSLRTLPVAQAR---------------------E----IVVTQVLEFAVVHPH-GVVLL    229
tr|S9VXS8|S9VXS8_9TRYP              GTAYDNEPVALAR---------------------A----RIVEQVTDFSTRHPH-GVVLL    231
tr|A0A0M9FWU4|A0A0M9FWU4_LEPPY      GTGFDGMPVSEAR---------------------G----RIVRQVTAHMEHYPH-GVVLI    247
tr|A4H5R3|A4H5R3_LEIBR              GTGFDGLSVAEAR---------------------Q----RIVGQIVAHVARYPH-GVVLI    262
tr|Q4QHU2|Q4QHU2_LEIMA              GTGFDGLSVAEAR---------------------Q----RIIGQIIAHTDCYPH-GVVLI    233
tr|A4HU03|A4HU03_LEIIN              GTGFDGLSVAEAR---------------------Q----RIIGQIITHTDRYPH-GVVLI    233
tr|Q382P2|Q382P2_TRYB2              GTGFGGLEGRDGL---------------------N----LLVRKITEHQRKYPH-GIVLL    226
tr|A0A1X0NMJ2|A0A1X0NMJ2_9TRYP      GTSFDELDITEAR---------------------Q----SIVRRITAHQRYYPH-GIILI    211
tr|Q4D6G4|Q4D6G4_TRYCC              GTGFDGMPIEDAR---------------------T----RIIQRVTAHQKVYPH-GIVLL    210
tr|Q4D903|Q4D903_TRYCC              GTGFDGMPIEDAR---------------------T----RIIQRVTAHQKVYPH-GIVLL    210
tr|A0A422ND04|A0A422ND04_9TRYP      GTGFDGMPVADAR---------------------L----RIMQRVTAHQKAYPH-GIVLL    210
tr|A0A3R7RCD3|A0A3R7RCD3_TRYRA      GTGFDGMPIADAR---------------------L----RIMQRITAHQKAYPH-GIVLL    210
                                                                               :   :            :::.

tr|D7G3V5|D7G3V5_ECTSI              DELQKAAPGTLDALAEAMSEHPRVTFE---------------------------------    212
tr|Q55FE2|Q55FE2_DICDI              DEIQKIDPYIISVIEPFLDGATITISDEKKTTT---------------------------    263
tr|L8GYA8|L8GYA8_ACACA              DEAEMMRADILRVVGAFMDDSQTTVSSLKDPSK---------------------------    203
tr|A0A0L1L0A3|A0A0L1L0A3_9EUGL      DEVTQMHPVLLQELNPLFSAIEHGESIN--------------------------------    239
tr|A0A0S4KNW8|A0A0S4KNW8_BODSA      NDMTELSPAQANVLMPLLGRSKHFPEDTHQ------------------------------    215
tr|A0A422N6P1|A0A422N6P1_TRYRA      NDLGALHPDLVRVLLPLLGRAPSFPEAS--------------------------------    216
tr|A0A3R7NQX6|A0A3R7NQX6_9TRYP      NDLGALHPDLVRVLLPLLGRAPSFPEAP--------------------------------    219
tr|S9U3X1|S9U3X1_9TRYP              NELTSLHPEKVMVLLPLLGRGSYFPEHP--------------------------------    178
tr|S9U3J6|S9U3J6_9TRYP              NELTSLHPEKVMVLLPLLGRGSYFPEHP--------------------------------    220
tr|A0A0N0VF20|A0A0N0VF20_LEPPY      NELSSLEPGKVRVLLPLLGRASEFPEHF--------------------------------    214
tr|A4I131|A4I131_LEIIN              NELTSLEPSKVRVLSPLLGRGTSFPENP--------------------------------    218
tr|Q4QAA8|Q4QAA8_LEIMA              NELTSLEPNKVRVLSPLLGRGTSFPESL--------------------------------    218
tr|A0A7G2CLT7|A0A7G2CLT7_9TRYP      DDLSALHPELIQGLSPLFGRAPYFPEQLLKEDK---------------------------    262
tr|S9VXS8|S9VXS8_9TRYP              DDVTAMDPELVRSLAPLLGRAAYFSDQLFENATAPDSAAEP-------------------    272
tr|A0A0M9FWU4|A0A0M9FWU4_LEPPY      DDLTAMEPALVAALAPLFGRASHFAEQLTDPQADSTHSSNVNN---------------DN    292
tr|A4H5R3|A4H5R3_LEIBR              DDLTAMDPVLVSVLAPLFGRASHFPEQLARSSSATAARVRHTTAKPLQTAHQWMKVFGRD    322
tr|Q4QHU2|Q4QHU2_LEIMA              DDLTAMDPSLVSALAPLFGRAAHFPEQLTRSSSATAAGARHAAAKSLQTIHASTKGLGPD    293
tr|A4HU03|A4HU03_LEIIN              DDLTAMDPSLVSALAPLFGRAAHFPEQLTHPSTATAAGARHAAAKSLQTMHESTKGLGPD    293
tr|Q382P2|Q382P2_TRYB2              DDLNAMHPSLVALLAPLFGRADRFEGQAAD------------------------------    256
tr|A0A1X0NMJ2|A0A1X0NMJ2_9TRYP      DDLTAMHPKLVAALAPLFGRAERFNEQPEN------------------------------    241
tr|Q4D6G4|Q4D6G4_TRYCC              DDLAAMHPKLVAALAPLLGRAERFEEQPED------------------------------    240
tr|Q4D903|Q4D903_TRYCC              DDLAAMHPKLVAALAPLLGRAERFEEQPEG------------------------------    240
tr|A0A422ND04|A0A422ND04_9TRYP      DDLTAMHPKLVAALAPLLGRAERFEEQPAG------------------------------    240
tr|A0A3R7RCD3|A0A3R7RCD3_TRYRA      DDLTAMHPKLVAALAPLLGRAERFEEQPEG------------------------------    240
                                    ::               :   :.

tr|D7G3V5|D7G3V5_ECTSI              ------------------------------------RGGQNVSVDSSRVVFLLVSDVGAE    236
tr|Q55FE2|Q55FE2_DICDI              -----------------------------------------TKINTSLGTYILTSDFDKE    282
tr|L8GYA8|L8GYA8_ACACA              ------------------------------------------RVNTKEAIIILISDFGRD    221
tr|A0A0L1L0A3|A0A0L1L0A3_9EUGL      ------------------------------------------GVPMTGVFCFLTSDFGSN    257
tr|A0A0S4KNW8|A0A0S4KNW8_BODSA      ------------------------------------------RVDLHKLMVVVTTDFGKQ    233
tr|A0A422N6P1|A0A422N6P1_TRYRA      ------------------------------------------NVPLGRLVVIVTTDFGRQ    234
tr|A0A3R7NQX6|A0A3R7NQX6_9TRYP      ------------------------------------------GVPLGRLFVIVTTDFGRQ    237
tr|S9U3X1|S9U3X1_9TRYP              ------------------------------------------QVSLRGAIVIITTDLGRE    196
tr|S9U3J6|S9U3J6_9TRYP              ------------------------------------------QVSLRGAIVIITTDLGRE    238
tr|A0A0N0VF20|A0A0N0VF20_LEPPY      ------------------------------------------DVKISTQLVVLTTDFGRE    232
tr|A4I131|A4I131_LEIIN              ------------------------------------------GVSIAPLLVILTTDFGRE    236
tr|Q4QAA8|Q4QAA8_LEIMA              ------------------------------------------GVSIAPLLVILTTDFGRE    236
tr|A0A7G2CLT7|A0A7G2CLT7_9TRYP      ----------------------------------------KEKLVSLSNLLVIITTDFGKQ    283
tr|S9VXS8|S9VXS8_9TRYP              ------------------------------------RLLRRLGPSLRHLLVIVTTDFGKQ    296
tr|A0A0M9FWU4|A0A0M9FWU4_LEPPY      HLRGSSSQKGL-----------------LSWAWRPFSSPLKPPPPLSQLLVFITTDFGRQ    335
tr|A4H5R3|A4H5R3_LEIBR              GGRGSAK-EGNDRKGEAAAGIDGAPPAASEMTSSLSRSGQPPPPPLSQLLVFITTDFGRQ    381
tr|Q4QHU2|Q4QHU2_LEIMA              GGTGSAAKKGESEKWEAAADTGGAQPAASEMASSSLSSGQP-PPPLSQLIIFITTDFGRQ    352
tr|A4HU03|A4HU03_LEIIN              GGRGSAAKKGEDEKREAAAGTGGAQPSTSEMASSSLSSGQP-PPPLSQLIIFVTTDFGRQ    352
tr|Q382P2|Q382P2_TRYB2              ------------------------------------------LPSLKELTVIVTTDFGKQ    274
tr|A0A1X0NMJ2|A0A1X0NMJ2_9TRYP      ------------------------------------------GPSLAQLTVVVTTDFGQQ    259
tr|Q4D6G4|Q4D6G4_TRYCC              ------------------------------------------TPSLAQLIVVVTTDFGKQ    258
tr|Q4D903|Q4D903_TRYCC              ------------------------------------------TPSLAQLIVIVTTDFGKQ    258
tr|A0A422ND04|A0A422ND04_9TRYP      ------------------------------------------TPSLAQLLVVVTTDFGQQ    258
tr|A0A3R7RCD3|A0A3R7RCD3_TRYRA      ------------------------------------------TPSLAQLLVVVTTDFGQQ    258
                                                                                        .: :*.. :
```

```
tr|D7G3V5|D7G3V5_ECTSI             GVNAAVLRYRKRSDVVPGALQSAVKRSLDEQWERLRFGKMVDKVVPYLPMDPASNLLVVE    296
tr|Q55FE2|Q55FE2_DICDI             GMTYNQSIL---------ELKRKATAMFKSIYGDSKFSKLVTESLPFLPLSKSNSKLQII    333
tr|L8GYA8|L8GYA8_ACACA             EIRTGDSWD---------EIAERVHRETKAILQEDLMVQRIQYHIPFSP-----------    261
tr|A0A0L1L0A3|A0A0L1L0A3_9EUGL     GKTLGMTST---------EVRTLVWEVIQETYKYTPA-LKKANVIPFLALSPADYQDAIR    307
tr|A0A0S4KNW8|A0A0S4KNW8_BODSA     GRTRGKSIE---------ELQQMVEQEVRGTFGALAG--SYLRTYAFIPATLPAVRDIVR    282
tr|A0A422N6P1|A0A422N6P1_TRYRA     GRTRGKSLM---------EMRRIVEDDFKSLYSQLSS--SMIETFPFLPAALDTAKEIVR    283
tr|A0A3R7NQX6|A0A3R7NQX6_9TRYP     GRTQGKSLA---------EMRRLVEEDFKSLYSQLSS--SMIETFPFLPAALDTAKEIVR    286
tr|S9U3X1|S9U3X1_9TRYP             GRTRGKNLA---------QMRALIESDFQDLYSKLST--SYLHTFPFLPITLDAATDIVR    245
tr|S9U3J6|S9U3J6_9TRYP             GRTRGKNLA---------QMRALIESDFQDLYSKLST--SYLHTFPFLPITLDAATDIVR    287
tr|A0A0N0VF20|A0A0N0VF20_LEPPY     GRTRGKGLS---------EMRSFINSEFTDLYSAQFA--SHVRTLPFLPISLDTAGEIVR    281
tr|A4I131|A4I131_LEIIN             GRTRGKSLF---------EMRAFITDEFADLYSKEAA--SHVRTFPFLPISLSTAGDIVR    285
tr|Q4QAA8|Q4QAA8_LEIMA             GRTRGKSLF---------EMRAFINDEFADLYSKEAA--SHVRTFPFLPISLSTAGDIVR    285
tr|A0A7G2CLT7|A0A7G2CLT7_9TRYP     GVTIGKSLD---------EIKALVDKDFASLYGSLLT--AHIYTFPYLAFNEQMGMEMIR    332
tr|S9VXS8|S9VXS8_9TRYP             GRTVGLTTS---------EIRAMVLEEFSALYGSLLP--AYTRTFIYVPFTEQTAVEVVL    345
tr|A0A0M9FWU4|A0A0M9FWU4_LEPPY     GRTVGKSRA---------DIEAMVQHDFASLYGTLLP--AYTRTFVFFPFTTQVAEEVVR    384
tr|A4H5R3|A4H5R3_LEIBR             GRTVGKSRA---------EIEAMIQHDFADLYGSLLP--AYTRTFVYFPFTQAMAEDVVR    430
tr|Q4QHU2|Q4QHU2_LEIMA             GRTVGKSRT---------EIEAMIQHDFADLYGTLLP--AYTRTFVYYPFTSAMAEDVVR    401
tr|A4HU03|A4HU03_LEIIN             GRTVGKSRT---------EIEAMIQHDFADLYGTLLP--AYTRTFVYYPFTSAMAEDVVR    401
tr|Q382P2|Q382P2_TRYB2             GRTFGKSVV---------EVEKMVRMEFNSLYGSFVP--AFVRTLAFAAFSKRSAEEMVR    323
tr|A0A1X0NMJ2|A0A1X0NMJ2_9TRYP     GRTWGKSVV---------EIEQMVRVDFAGLYGTLVP--AFARTMVFVSLSQQSAEEMVR    308
tr|Q4D6G4|Q4D6G4_TRYCC             GRTWGKSMA---------EIEQLVRDEFAGLYGTLLS--AFTRTMLFLPFSRHDAEEMIR    307
tr|Q4D903|Q4D903_TRYCC             GRTWGKSMA---------EIEQLVRDEFAGLYGTLLS--AFTRTMLFLPFSRHDAEEMIR    307
tr|A0A422ND04|A0A422ND04_9TRYP     GRTWGKSTA---------EVEQLVRDEFAGLYGTLTS--AFTRTMLFAAFSRRDAEKLVQ    307
tr|A0A3R7RCD3|A0A3R7RCD3_TRYRA     GRTWGKSMA---------EVEQLVRDEFSGLYGTLVS--AFTRTMLFTAFSRLDAEQMVR    307
                                                     :                        :

tr|D7G3V5|D7G3V5_ECTSI             LKLKKLAETLDGG--------------------LYTTS------GLRWHLVQ--------    322
tr|Q55FE2|Q55FE2_DICDI             KEIENSFCRENHL----------------------SILSIELKSQVSEIIFQKMNTLYPN    371
tr|L8GYA8|L8GYA8_ACACA             --VPDLNCTARVG-----------------------KVSAPVEELVSLLVRQLKEHPAAK    296
tr|A0A0L1L0A3|A0A0L1L0A3_9EUGL     YRLNTLKCQHNKKNS-LFGNTNI----------NILEFTFDEEVLVDFLYCKILSGI-PQ    355
tr|A0A0S4KNW8|A0A0S4KNW8_BODSA     LIFNDWACSEK-----------------------MNSLSVT-PEAIDVVVDGCVGRV-AF    317
tr|A0A422N6P1|A0A422N6P1_TRYRA     LTIQDYKCRHG---------------------ETIRELRVS-EDAVLWFVELVHDDL-PM    320
tr|A0A3R7NQX6|A0A3R7NQX6_9TRYP     LTILDYKCRHG---------------------EAIRELRVS-EDAVLWFVDLVRDDL-PV    323
tr|S9U3X1|S9U3X1_9TRYP             MGVADSRCAWS-----------------------M-NVTIS-DDAVAWMLEGAKPYL-AS    279
tr|S9U3J6|S9U3J6_9TRYP             MGVADSRCAWS-----------------------M-NVTIS-DDAVAWMLEGAKPYL-AS    321
tr|A0A0N0VF20|A0A0N0VF20_LEPPY     VVAKEIGCSAT---------------------PPV-RLAIT-DTAVLWLVEKTKGSL-AV    317
tr|A4I131|A4I131_LEIIN             VVVREIGCSAP---------------------QPL-CLAIS-DSAVVWLVERTKILL-PA    321
tr|Q4QAA8|Q4QAA8_LEIMA             VVVREIGCSAQ---------------------QPL-CLAIS-DSAVVWLVERTKMLL-SA    321
tr|A0A7G2CLT7|A0A7G2CLT7_9TRYP     HKISQLSCVPGHFLSSWFHLEEDEALRSILARSRVTASWME-EDAAKFLIDLHRPLWEAS    391
tr|S9VXS8|S9VXS8_9TRYP             ATIDYLPCQLLLA-----S----------GGAAALRASEID-ADAAAFLVHKYREVWQGR    389
tr|A0A0M9FWU4|A0A0M9FWU4_LEPPY     SVVTDLPCALG--------------------EHLIAESWIS-DDAVAFLVEQHRAAWAGK    423
tr|A4H5R3|A4H5R3_LEIBR             SAVTDLPCALG--------------------ERLIASSTIS-DEAVAFLVQQHRQLWSGK    469
tr|Q4QHU2|Q4QHU2_LEIMA             SAVTDLPCALG--------------------ERLIASSTIS-DEAVSFLVQQHRQLWSGK    440
tr|A4HU03|A4HU03_LEIIN             SAVTDLPCALG--------------------ERLIASSTIS-DEAVSFLVQQHRQLWSGK    440
tr|Q382P2|Q382P2_TRYB2             TTVITLPCTAYRY-----GFAGPN-----AYGGGVVASSID-DVAVSFLVERYREVWEGR    372
tr|A0A1X0NMJ2|A0A1X0NMJ2_9TRYP     KAAAALACLNNWG-----------------G-GTVMASTIE-ELAVAYLVERYRDTWEGR    349
tr|Q4D6G4|Q4D6G4_TRYCC             IAVAALPCGTYWN-----------------K-GAVVTSSIE-DLAVTFLVERHRETWEGK    348
tr|Q4D903|Q4D903_TRYCC             IAVAALPCGTYWN-----------------K-GAVVTSSIE-DLAVTFLVERHRETWEGR    348
tr|A0A422ND04|A0A422ND04_9TRYP     MAVAALPCTRYWS-----------------DGGAVVASSID-ELAVAFLVERHRETWEGK    349
tr|A0A3R7RCD3|A0A3R7RCD3_TRYRA     IAVAALPCAKYWS-----------------DSGSVVASSID-DLAVAFLVERHRETWEGK    349


tr|D7G3V5|D7G3V5_ECTSI             ------PQYIQYSSYHVTLPNDGREEIIRHQL------AAYGARDVEKVSMRRLTGAIRN    370
tr|Q55FE2|Q55FE2_DICDI             ----------------------------------------ENFRAIEKILNYLIFNKVSN    391
tr|L8GYA8|L8GYA8_ACACA             RDNIVIKKYLPFFATTVGADIHAIAEKLIHFMVAHEHYCERNYRGIEGLFATKVVGPI--    354
tr|A0A0L1L0A3|A0A0L1L0A3_9EUGL     ----------------------------------------RNGREIDRIFDDYIEGPL--    373
tr|A0A0S4KNW8|A0A0S4KNW8_BODSA     ----------------------------------------ENGRAVVLHMDVELVRL---    334
tr|A0A422N6P1|A0A422N6P1_TRYRA     ----------------------------------------ENGRCVAHAVSALVGPGI--    338
tr|A0A3R7NQX6|A0A3R7NQX6_9TRYP     ----------------------------------------ENGRCVAQAVSALVGPGI--    341
tr|S9U3X1|S9U3X1_9TRYP             ----------------------------------------ENGRAVIKEVLAAVEPLL--    297
tr|S9U3J6|S9U3J6_9TRYP             ----------------------------------------ENGRAVIKEVLAAVEPLL--    339
tr|A0A0N0VF20|A0A0N0VF20_LEPPY     ----------------------------------------ENGRAVAQETKLHVGSLM--    335
tr|A4I131|A4I131_LEIIN             ----------------------------------------ENGRAVAFETKLQLEALL--    339
tr|Q4QAA8|Q4QAA8_LEIMA             ----------------------------------------ENGRAVAFETKLQLEALL--    339
tr|A0A7G2CLT7|A0A7G2CLT7_9TRYP     ----------------------------------------ENGRSIEKNIIQ--------    403
tr|S9VXS8|S9VXS8_9TRYP             ----------------------------------------ENGHALRRALEDEVLTPL--    407
tr|A0A0M9FWU4|A0A0M9FWU4_LEPPY     ----------------------------------------ENGHALRRLVEDELVSQL--    441
tr|A4H5R3|A4H5R3_LEIBR             ----------------------------------------ENGHALRRLIEDEIVSQL--    487
tr|Q4QHU2|Q4QHU2_LEIMA             ----------------------------------------ENGHALRRLVEDEIVSQL--    458
tr|A4HU03|A4HU03_LEIIN             ----------------------------------------ENGHALRRLVEDEIVSQL--    458
tr|Q382P2|Q382P2_TRYB2             ----------------------------------------ENGHALRRAVEDSLLSLL--    390
tr|A0A1X0NMJ2|A0A1X0NMJ2_9TRYP     ----------------------------------------ENGHALRRVVQDTLVPLL--    367
tr|Q4D6G4|Q4D6G4_TRYCC             ----------------------------------------ENGHSLRRAVEDSLVSLL--    366
tr|Q4D903|Q4D903_TRYCC             ----------------------------------------ENGHSLRRAVEDSLVSLL--    366
tr|A0A422ND04|A0A422ND04_9TRYP     ----------------------------------------ENGHALRRAVEDSLISLL--    367
tr|A0A3R7RCD3|A0A3R7RCD3_TRYRA     ----------------------------------------ENGHALRRAVEDSLISLL--    367
                                                                              . : :

tr|D7G3V5|D7G3V5_ECTSI             HVLNPTCAATDEGAEETGSPEGQGRGGGGGRPAQGGYGGSSSSSRGGGGGRCWS------    424
tr|Q55FE2|Q55FE2_DICDI             FII----QDYDKLI-----EET-KINNNNND-------------N--ENEKIQQQNENYK    426
tr|L8GYA8|L8GYA8_ACACA             --L----KQMPEPR--PSTQ----------------------------------------    366
tr|A0A0L1L0A3|A0A0L1L0A3_9EUGL     --V----MKLAEFE--MCASEK-STSTGGSR-----------------ESNLWE------    401
tr|A0A0S4KNW8|A0A0S4KNW8_BODSA     -------QHPAGRS-----LQG-L--N-----------------T---TVTARN------    353
tr|A0A422N6P1|A0A422N6P1_TRYRA     --L----RHLLDSP-----LAP-V-------------------------SLHVDI-----    356
tr|A0A3R7NQX6|A0A3R7NQX6_9TRYP     --L----RHLPDNP-----SAL-V-------------------------SLYVDI-----    359
tr|S9U3X1|S9U3X1_9TRYP             --E----QGDKVR--------P-YIID---------------------------------    309
tr|S9U3J6|S9U3J6_9TRYP             --E----QGDKVR--------P-YIID---------------------------------    351
```

```
tr|A0A0N0VF20|A0A0N0VF20_LEPPY     --E----RLQADEA-----SHP-S--S-----------------K---TLRPRDGAAN--    359
tr|A4I131|A4I131_LEIIN             --E----QVMDNCT-----HES-GIIA-----------------T---DELYLDVDAT--    365
tr|Q4QAA8|Q4QAA8_LEIMA             --E----QVMGNYT-----YES-GIIA-----------------T---DELHLDVDGT--    365
tr|A0A7G2CLT7|A0A7G2CLT7_9TRYP     --L----ELWNTLR--TG-LKT-LYHHLGLS-------------Q------RFEVFRQ--    432
tr|S9VXS8|S9VXS8_9TRYP             --L----LAMEELQ-----QQP-ARHGRGQR-------------RAAAASTLWDPAAA--    440
tr|A0A0M9FWU4|A0A0M9FWU4_LEPPY     --I----VHWELHA-----VR----------------------------ERLL-------    455
tr|A4H5R3|A4H5R3_LEIBR             --V----AYWETHE-----EQK-AF------------------------ERLR-------    504
tr|Q4QHU2|Q4QHU2_LEIMA             --I----VYWETHA-----QQR-HF------------------------ERLR-------    475
tr|A4HU03|A4HU03_LEIIN             --V----VYWETHA-----QQK-HF------------------------ERLR-------    475
tr|Q382P2|Q382P2_TRYB2             --L----KYFDEHG-----HD----------------------------RRVW-------    404
tr|A0A1X0NMJ2|A0A1X0NMJ2_9TRYP     --L----QYFDREG-----HD----------------------------QAVW-------    381
tr|Q4D6G4|Q4D6G4_TRYCC             --L----QYFDQNG-----HD----------------------------KFVW-------    380
tr|Q4D903|Q4D903_TRYCC             --L----QYFDQHG-----HD----------------------------KFVW-------    380
tr|A0A422ND04|A0A422ND04_9TRYP     --L----QYFDQHG-----HD----------------------------TVVW-------    381
tr|A0A3R7RCD3|A0A3R7RCD3_TRYRA     --L----KYFDKHG-----QD----------------------------TVVW-------    381


tr|D7G3V5|D7G3V5_ECTSI             EPFM--VDIRYDAS----------TEQVSFHRCEPDYADGL-------------------    453
tr|Q55FE2|Q55FE2_DICDI             IPFNYVYDSIFKIIKNNKILENKNNHNDEVLITLSIKPNNL-------------------    467
tr|L8GYA8|L8GYA8_ACACA             --------------------------EVTVLVKVPFDQS--------VVQPARAPGGGR-    391
tr|A0A0L1L0A3|A0A0L1L0A3_9EUGL     ---------YLFLG----------SSSIDVSVSFLVV----------------------N    420
tr|A0A0S4KNW8|A0A0S4KNW8_BODSA     ---G---AVEISID----------RDADDAMSDL--------------------------    371
tr|A0A422N6P1|A0A422N6P1_TRYRA     -------------D----------DNGSVTVLPLPAG-----------------------    370
tr|A0A3R7NQX6|A0A3R7NQX6_9TRYP     -------------D----------DNGSVAVLPLPAR-----------------------    373
tr|S9U3X1|S9U3X1_9TRYP             ------------VD----------AHGHLALFSEEYTV----------------------    325
tr|S9U3J6|S9U3J6_9TRYP             ------------VD----------AHGHLALFSEEYTV----------------------    367
tr|A0A0N0VF20|A0A0N0VF20_LEPPY     CSVP---ACNIYVD----------DGGQIGLAC---------------------------    379
tr|A4I131|A4I131_LEIIN             CPYR---RCTVFLE----------GDGTLAMTCQGTGTHSR------------VPPG---    397
tr|Q4QAA8|Q4QAA8_LEIMA             CPYR---RCTVFLE----------GDGTLAMTCQGTGTHTR------------VSPD---    397
tr|A0A7G2CLT7|A0A7G2CLT7_9TRYP     ---L---EIVFYLR----------EETATVAVRING--------------IA------VD    456
tr|S9VXS8|S9VXS8_9TRYP             ---T---DVRFFLD----------AAQWRIHFSLLQY--------GEVVYTAGEPSGAWG    476
tr|A0A0M9FWU4|A0A0M9FWU4_LEPPY     --------VRFELD----------EAELCVVLRLPNQHATAV---F-------DVDAVMP    487
tr|A4H5R3|A4H5R3_LEIBR             --------VRFELD----------EASMRVLLRTPHASTTTA---L-------PPSSALS    536
tr|Q4QHU2|Q4QHU2_LEIMA             --------VHFDLD----------EASMHVLLRTPHTSSTIA---S-------QPPAALS    507
tr|A4HU03|A4HU03_LEIIN             --------VHFELD----------EASMRVLLRTPHTSRTTA---S-------QPPAALS    507
tr|Q382P2|Q382P2_TRYB2             --------ARFHLD----------EKVGEIVLDAGADPHSMNDL----------------    430
tr|A0A1X0NMJ2|A0A1X0NMJ2_9TRYP     --------ARFRLD----------TTAGKIILDTGRDENGLDPSYVDSVEAGTGTSGALG    423
tr|Q4D6G4|Q4D6G4_TRYCC             --------AHFRLD----------ERAAKIVLSTGWKYDHMETSSEEFFGVATEAERTVL    422
tr|Q4D903|Q4D903_TRYCC             --------AHFRLD----------ERAAKIVLSTGWNYDHMETSSEEFFGVAIEAEGTVL    422
tr|A0A422ND04|A0A422ND04_9TRYP     --------AYFRLD----------ELTAKIVLSTGAERYQTDTRSIGYSGVAAGPGGAAA    423
tr|A0A3R7RCD3|A0A3R7RCD3_TRYRA     --------AYFRLD----------ELTAKIVLSTGSEYNQADVRSEESSGITTGGGGAAT    423


tr|D7G3V5|D7G3V5_ECTSI             -TEL-------------------QREHPDVVEGPKCDLAWRGVLHEHGALA---------    484
tr|Q55FE2|Q55FE2_DICDI             -----------------------------------------------------NNLLIID    474
tr|L8GYA8|L8GYA8_ACACA             ------------------EDL---------------------------------------    394
tr|A0A0L1L0A3|A0A0L1L0A3_9EUGL     STE-----TQGLDKKLVIEVLLDQRKH---------------------------------    442
tr|A0A0S4KNW8|A0A0S4KNW8_BODSA     ------------------------------------------------------------    371
tr|A0A422N6P1|A0A422N6P1_TRYRA     ------------------------------------------------------------    370
tr|A0A3R7NQX6|A0A3R7NQX6_9TRYP     ------------------------------------------------------------    373
tr|S9U3X1|S9U3X1_9TRYP             ------------------------------------------------------------    325
tr|S9U3J6|S9U3J6_9TRYP             ------------------------------------------------------------    367
tr|A0A0N0VF20|A0A0N0VF20_LEPPY     ------------------------------------------------------------    379
tr|A4I131|A4I131_LEIIN             ------------------------------------------------------------    397
tr|Q4QAA8|Q4QAA8_LEIMA             ------------------------------------------------------------    397
tr|A0A7G2CLT7|A0A7G2CLT7_9TRYP     EIQAQAEAKQA--EEG--D-FVGK-KKVFLIIK-KISRKWDCYIVDFETKNSIFFLLLFF    509
tr|S9VXS8|S9VXS8_9TRYP             EVEG------------------------------DL------------------------    482
tr|A0A0M9FWU4|A0A0M9FWU4_LEPPY     APEDDAASDEG---------------T----AG-DL------------------------    503
tr|A4H5R3|A4H5R3_LEIBR             STEARAHVRDGVHNEG--------EEE----PS-DL------------------------    559
tr|Q4QHU2|Q4QHU2_LEIMA             SAEARAYDHGGADDRD--------GEE----RG-DL------------------------    530
tr|A4HU03|A4HU03_LEIIN             SVEARAYGHGGADDGD--------GEE----RS-DL------------------------    530
tr|Q382P2|Q382P2_TRYB2             ------------------------------------------------------------    430
tr|A0A1X0NMJ2|A0A1X0NMJ2_9TRYP     DSVG-------K---------KRRDEERHVIND-DF------------------------    442
tr|Q4D6G4|Q4D6G4_TRYCC             ---------SGEEKDGGGENVAERGKHASTLNE-DL------------------------    448
tr|Q4D903|Q4D903_TRYCC             ---------SGEDKEGGGEDVAEKGKHASTFNG-DL------------------------    448
tr|A0A422ND04|A0A422ND04_9TRYP     AAAAAAATTADRPGEGKAEEFAERGIGPYTLNG-DL------------------------    458
tr|A0A3R7RCD3|A0A3R7RCD3_TRYRA     -----------RPGE-EVDEVAARGKGPYTLNG-DL------------------------    446


tr|D7G3V5|D7G3V5_ECTSI             ------------------------    484
tr|Q55FE2|Q55FE2_DICDI             IF------------PSKNVYKNTN    486
tr|L8GYA8|L8GYA8_ACACA             ------------------------    394
tr|A0A0L1L0A3|A0A0L1L0A3_9EUGL     ------------------------    442
tr|A0A0S4KNW8|A0A0S4KNW8_BODSA     ------------------------    371
tr|A0A422N6P1|A0A422N6P1_TRYRA     ------------------------    370
tr|A0A3R7NQX6|A0A3R7NQX6_9TRYP     ------------------------    373
tr|S9U3X1|S9U3X1_9TRYP             ------------------------    325
tr|S9U3J6|S9U3J6_9TRYP             ------------------------    367
tr|A0A0N0VF20|A0A0N0VF20_LEPPY     ------------------------    379
tr|A4I131|A4I131_LEIIN             ------------------------    397
tr|Q4QAA8|Q4QAA8_LEIMA             ------------------------    397
tr|A0A7G2CLT7|A0A7G2CLT7_9TRYP     LFHKKIIKLKKKKWPPRKVCSS--    531
tr|S9VXS8|S9VXS8_9TRYP             ------------------------    482
tr|A0A0M9FWU4|A0A0M9FWU4_LEPPY     ------------------------    503
tr|A4H5R3|A4H5R3_LEIBR             ------------------------    559
tr|Q4QHU2|Q4QHU2_LEIMA             ------------------------    530
tr|A4HU03|A4HU03_LEIIN             ------------------------    530
```

```
tr|Q382P2|Q382P2_TRYB2              ------------------------    430
tr|A0A1X0NMJ2|A0A1X0NMJ2_9TRYP      ------------------------    442
tr|Q4D6G4|Q4D6G4_TRYCC              ------------------------    448
tr|Q4D903|Q4D903_TRYCC              ------------------------    448
tr|A0A422ND04|A0A422ND04_9TRYP      ------------------------    458
tr|A0A3R7RCD3|A0A3R7RCD3_TRYRA      ------------------------    446
```

(overall sequence identity = 0.0075)

09. BIP-like HSP70 chaperones

BIP-like chaperones (Binding immunoglobulin protein, BIP, also known as 78 kDa glucose-regulated proteins, GRP78) are core components of the eukaryotic folding machinery associated with translocons. Hence the kinetoplastid proteins align well with BIPs from all other major groups, including fungal, plant and animal orthologues. The KDEL-like retention signal is also extremely conserved. Although sharing a HSP70-type core, BIP proteins differ greatly from the other key ER-localized HSP70 group (HYOU1), and should be handled as an ancestrally separate family.

```
tr|A0A0L1KCY6|A0A0L1KCY6_9EUGL      ------------------------------------------------------------    0
tr|A0A0S4JK39|A0A0S4JK39_BODSA      ------------------------------------------------------MF----    2
tr|A0A7G2CL24|A0A7G2CL24_9TRYP      ------------------------------------------------------------    0
tr|A0A0S4IPK6|A0A0S4IPK6_BODSA      ------------------------------------------------------------    0
tr|A0A0N0DZK2|A0A0N0DZK2_LEPPY      ------------------------------------------------------------    0
tr|A4I3J9|A4I3J9_LEIIN              ------------------------------------------------------------    0
tr|Q4Q8E6|Q4Q8E6_LEIMA              ------------------------------------------------------------    0
tr|A4HGG7|A4HGG7_LEIBR              ------------------------------------------------------------    0
tr|S9U467|S9U467_9TRYP              MVLCFLGLLLFEYTVVLLHSLFLVCTFCTVYPLVLIANCDCEYCILSLQYRKKNFFSSTK    60
tr|S9W3A0|S9W3A0_9TRYP              ------------------------------------------------------------    0
tr|A0A1X0NYK3|A0A1X0NYK3_9TRYP      ------------------------------------------------------------    0
tr|Q4D620|Q4D620_TRYCC              ------------------------------------------------------------    0
tr|K2NLB6|K2NLB6_TRYCR              ------------------------------------------------------------    0
tr|A0A422P6Q7|A0A422P6Q7_9TRYP      ------------------------------------------------------------    0
tr|A0A422N1T5|A0A422N1T5_TRYRA      ------------------------------------------------------------    0
tr|F9WRV9|F9WRV9_TRYVY              ------------------------------------------------------------    0
tr|F9W3P7|F9W3P7_TRYCI              ------------------------------------------------------------    0
tr|Q384Q5|Q384Q5_TRYB2              ------------------------------------------------------------    0
tr|A0A1Y2AI48|A0A1Y2AI48_9FUNG      ------------------------------------------------------------    0
tr|A0A1Y1X8R1|A0A1Y1X8R1_9FUNG      ------------------------------------------------------------    0
sp|P11021|BIP_HUMAN                 ------------------------------------------------------------    0
tr|A0A0G4EPG2|A0A0G4EPG2_VITBC      ------------------------------------------------------------    0
tr|M1BLB0|M1BLB0_SOLTU              ------------------------------------------------------------    0
sp|Q9LKR3|BIP1_ARATH                ------------------------------------------------------------    0
tr|I1KPN3|I1KPN3_SOYBN              ------------------------------------------------------------    0


tr|A0A0L1KCY6|A0A0L1KCY6_9EUGL      -------------------MLQ--T--TAILFAACFLCSAKEEKKQLEFPVVGIDLGTTY    37
tr|A0A0S4JK39|A0A0S4JK39_BODSA      -----------RNVRPLVALLIVTAVLLLLAGAAHAGAANTAASNKVEPPCVGIDLGTTY    51
tr|A0A7G2CL24|A0A7G2CL24_9TRYP      ----------MNLFRKRS-LI---ATTLLLLSASVLVLAAPSGTGKVAGPCVGVDLGTTY    46
tr|A0A0S4IPK6|A0A0S4IPK6_BODSA      ----------MQVTRGL---V---AAVLVAAVVVGTVLAGEASTNKVEAPCVGIDLGTTY    44
tr|A0A0N0DZK2|A0A0N0DZK2_LEPPY      ----------MAVRDRLL-LL---AVCLVAALLIVSAAAAPDGSGKVEPPCIGVDLGTTY    46
tr|A4I3J9|A4I3J9_LEIIN              ----------MTRKDNLT-LM---AVCLVSAMLVMSAAAAADGSGKVESPCIGVDLGTTY    46
tr|Q4Q8E6|Q4Q8E6_LEIMA              ----------MTRKDNLT-LM---AVCLVSAILVVSAAAVPDGSGKVESPCIGVDLGTTY    46
tr|A4HGG7|A4HGG7_LEIBR              ----------MMRKDSLT-LV---GVCLVSVMLVLSAAAAPDGSGKVEPPCIGVDLGTTY    46
tr|S9U467|S9U467_9TRYP              RIDTYTSSLKMPSLKELV-LL---LT-VLLVSVALLVSAAPEGAGKVEAPCVGVDLGTTY    115
tr|S9W3A0|S9W3A0_9TRYP              ----------MPSLKELV-LL---LT-VLLVSVALLVSAAPEGAGKVEAPCVGVDLGTTY    45
tr|A0A1X0NYK3|A0A1X0NYK3_9TRYP      -----------MTRFPL--VV---VL-GIVVVLAATVYAAPDGTGKVEAPCVGIDLGTTY    43
tr|Q4D620|Q4D620_TRYCC              --------------MLL--QA---LL---VLSAVVVAVAAPDGTGKVEAPCVGIDLGTTY    38
tr|K2NLB6|K2NLB6_TRYCR              --------------MFL--QV---LL---VLSTVVAAVAAPDGTGKVEAPCVGIDLGTTY    38
tr|A0A422P6Q7|A0A422P6Q7_9TRYP      -----------MPRLLL--AA---LL---VLSA-VAAAAAPDGTGKVEAPCVGIDLGTTY    40
tr|A0A422N1T5|A0A422N1T5_TRYRA      -----------MARLLL--AA---LL---VLFT-VVAAAAPDGTGKVEAPCVGIDLGTTY    40
tr|F9WRV9|F9WRV9_TRYVY              ----------MAKAMRL--AA---AA-LLLVAAATGAWAAPEASGKVEAPCVGIDLGTTY    44
tr|F9W3P7|F9W3P7_TRYCI              -----------MSGTSL--RT---AA-VLLVVAAAVATAAPESGGKVEAPCVGIDLGTTY    43
tr|Q384Q5|Q384Q5_TRYB2              -----------MSRMWL--TT---AA-VFLTVTVAAVSAAPESGGKVEAPCVGIDLGTTY    43
tr|A0A1Y2AI48|A0A1Y2AI48_9FUNG      ----------MK-------LSS--IFSTVVVALAAIVRAADSDDNGIKGPVIGIDLGTTY    41
tr|A0A1Y1X8R1|A0A1Y1X8R1_9FUNG      ----------MRFSIK--KLLI--AAASVVAFSAPKVFAASDDDGKVKGPIIGIDLGTTY    46
sp|P11021|BIP_HUMAN                 -----------------MKLSL--VAAMLL--LLSAARAEEEDKKEDVGTVVGIDLGTTY    39
tr|A0A0G4EPG2|A0A0G4EPG2_VITBC      ------------------MRSL--IVGAVLVAAIATSALAAEEEKKIEGPVVGIDLGTTY    40
tr|M1BLB0|M1BLB0_SOLTU              ----------MATALKRRASSI--VFAIVLLGSLFA-FSIAKEEATKLGTVIGIDLGTTY    47
sp|Q9LKR3|BIP1_ARATH                ----------MARS-FGANSTV--VLAIIFFGCLFA-LSSAIEEATKLGSVIGIDLGTTY    46
```

```
tr|I1KPN3|I1KPN3_SOYBN            ----------MAGS-WARRSLI--VLAIISFGCLFA-ISIAKEEATKLGTVIGIDLGTTY    46
                                                                                   :*:******

tr|A0A0L1KCY6|A0A0L1KCY6_9EUGL    SVVGVMKNGEVQIIPNEMGNRITPSVVAFT-ESERLIGDGAKNQLPQNPMNTIYTIKRLI    96
tr|A0A0S4JK39|A0A0S4JK39_BODSA    SVVGVWQNGEVQIIPNELGNRITPSVVAFT-ESERLVGDGAKNQLPQNPENTIYAIKRLI    110
tr|A0A7G2CL24|A0A7G2CL24_9TRYP    SVVGVWQKGEVHIIPNEMGNRITPSVVAFT-DDERLIGDGAKNQLPQNPENTVYAIKRLI    105
tr|A0A0S4IPK6|A0A0S4IPK6_BODSA    SVVGVWQKGEGQIIPNELGNRITPSVVAFT-ETERLIGDGAKNQLPQNPYNTIYTIKRLI    103
tr|A0A0N0DZK2|A0A0N0DZK2_LEPPY    SVAGVWQKGEVHIITNEMGNRITPSVVAFT-ETERLVGDGAKNQLPQNPENTIYAIKRLI    105
tr|A4I3J9|A4I3J9_LEIIN            SVAGVWQKGEVHIVTNEMGNRITPSVVAFT-DAERLVGDGAKNQLPQNPENTIYAIKRLI    105
tr|Q4Q8E6|Q4Q8E6_LEIMA            SVAGVWQKGEVHIVTNEMGNRITPSVVAFT-DAERLVGDGAKNQLPQNPENTIYAIKRLI    105
tr|A4HGG7|A4HGG7_LEIBR            SVAGVWQKGEVHVVTNEMGNRITPSVVAFT-DTERLVGDGAKNQLPQNPENTIYAIKRLI    105
tr|S9U467|S9U467_9TRYP            SVVGVWQKGDVHIVPNEMGNRITPSVVAFT-ETERLIGDGAKNQLPQNPENTIYAIKRLI    174
tr|S9W3A0|S9W3A0_9TRYP            SVVGVWQKGDVHIVPNEMGNRITPSVVAFT-ETERLIGDGAKNQLPQNPENTIYAIKRLI    104
tr|A0A1X0NYK3|A0A1X0NYK3_9TRYP    SVVGVWQKGDVHIIPNEMGNRITPSVVAFT-ETERLIGDGAKNQLPQNPHNTIYAIKRLI    102
tr|Q4D620|Q4D620_TRYCC            SVVGVWQKGDVHIIPNDMGNRITPSVVAFT-ETERLIGDGAKNQLPQNPHNTIYAIKRLI    97
tr|K2NLB6|K2NLB6_TRYCR            SVVGVWQKGDVHIIPNDMGNRITPSVVAFT-ETERLIGDGAKNQLPQNPHNTIYAIKRLI    97
tr|A0A422P6Q7|A0A422P6Q7_9TRYP    SVVGVWQKGDVHIIPNEMGNRITPSVVAFT-DTERLIGDGAKNQLPQNPHNTIYAIKRLI    99
tr|A0A422N1T5|A0A422N1T5_TRYRA    SVVGVWQKGDVHIIPNEMGNRITPSVVAFT-DTERLIGDGAKNQLPQNPHNTIYAIKRLI    99
tr|F9WRV9|F9WRV9_TRYVY            SVVGVWQKGDVHIIPNEMGNRITPSVVAFT-ETERLIGDGAKNQLPQNPHNTIYTIKRLI    103
tr|F9W3P7|F9W3P7_TRYCI            SVVGVWQKGDVHIIPNEMGNRITPSVVAFT-DTERLIGDGAKNQLPQNPHNTIYTIKRLI    102
tr|Q384Q5|Q384Q5_TRYB2            SVVGVWQKGDVHIIPNEMGNRITPSVVAFT-DTERLIGDGAKNQLPQNPHNTIYTIKRLI    102
tr|A0A1Y2AI48|A0A1Y2AI48_9FUNG    SCVGVYKNGRVEIIANDQGHRITPSYVAFT-DEERLVGDAAKNQAPMNPTNTIFDAKRLV    100
tr|A0A1Y1X8R1|A0A1Y1X8R1_9FUNG    SCVGIYQNGRVEILANDQGHRITPSYVAFT-EEERLVGDAAKNQAPSNPKNTIFDIKRLI    105
sp|P11021|BIP_HUMAN               SCVGVFKNGRVEIIANDQGNRITPSYVAFTPEGERLIGDAAKNQLTSNPENTVFDAKRLI    99
tr|A0A0G4EPG2|A0A0G4EPG2_VITBC    SCVGIYKNGRVEIIPNDQGNRITPSYVAFT-EDERLIGEAAKNQATVNPSQTLFDVKRLI    99
tr|M1BLB0|M1BLB0_SOLTU            SCVGVYKNGHVEIIANDQGNRITPSWVAFT-DGERLIGEAAKNQAAVNPERTVFDVKRLI    106
sp|Q9LKR3|BIP1_ARATH              SCVGVYKNGHVEIIANDQGNRITPSWVGFT-DSERLIGEAAKNQAAVNPERTVFDVKRLI    105
tr|I1KPN3|I1KPN3_SOYBN            SCVGVYKNGHVEIIANDQGNRITPSWVAFT-DSERLIGEAAKNQAAVNPERTIFDVKRLI    105
                                  * .*: ::*  .:: *: *:***** *.** : ***:*:.****   ** .*::  ***:

tr|A0A0L1KCY6|A0A0L1KCY6_9EUGL    GRKYTDKTVQYDKKLLSFDIVSH-NNEPRVQVNYK-GEKKVFTPEEISAMILTKMKEIAE    154
tr|A0A0S4JK39|A0A0S4JK39_BODSA    GRRYSDATVQKDKKLLSYELVEDKDGKPRVRVAVD-GKARLFTPEEVSAMVLQKMKEIAE    169
tr|A0A7G2CL24|A0A7G2CL24_9TRYP    GRKYTDATVQADKKLLSYEVIADKGGKPIVQVKAG-GEKKTYSPEEISAMVLQKMKQIAE    164
tr|A0A0S4IPK6|A0A0S4IPK6_BODSA    GRKYRDETVQRDKKLLSYELVEDKDGKPRVQVEVD-GKKKQFTPEEVSAMVLQKMKEIAE    162
tr|A0A0N0DZK2|A0A0N0DZK2_LEPPY    GRKFSDATVQNDKKLLSYKIVSDKAGKPLVQVTVS-GAKKEFTPEEVSAMVLQKMKDISE    164
tr|A4I3J9|A4I3J9_LEIIN            GRKYADPTVQNDKKLLSYHIVADKTGKPLVQVTVK-GQQKRFTPEEVSAMVLQKMKEISE    164
tr|Q4Q8E6|Q4Q8E6_LEIMA            GRKYADPTVQNDKKLLSYHIVADKTGKPLVQVTVK-GQQKRFTPEEVSAMVLQKMKEISE    164
tr|A4HGG7|A4HGG7_LEIBR            GRKYSDPTVQNDKKLLSYQIVADKVGKPLVQVTVK-GQQKRFTPEEVSAMVLQKMKEISE    164
tr|S9U467|S9U467_9TRYP            GRKYSDVTVQADKKLLSYEVVADKTGKPLVRVTVK-GAKKDFTAEEISSMVLQKMKEIAE    233
tr|S9W3A0|S9W3A0_9TRYP            GRKYSDVTVQADKKLLSYEVVADKTGKPLVRVTVK-GAKKDFTAEEISSMVLQKMKEIAE    163
tr|A0A1X0NYK3|A0A1X0NYK3_9TRYP    GRKYSDATVQKDKKLLSYEVVADRDGKPKVQVEVG-GKKKQFTPEEVSAMVLQKMKEIAE    161
tr|Q4D620|Q4D620_TRYCC            GRKYSDATVQTDKKLLSYEVVADKDGKPKVQVEVG-GKKKQFTPEEVSAMVLQKMKEIAE    156
tr|K2NLB6|K2NLB6_TRYCR            GRKYSDATVQTDKKLLSYEVVADKDGKPKVQVEVG-GKKKQFTPEEVSAMVLQKMKEIAE    156
tr|A0A422P6Q7|A0A422P6Q7_9TRYP    GRKYGDTTVQKDRKLLSYEVVADKDGKPKVQVEVG-GKKKQFTPEEVSAMVLQKMKEIAE    158
tr|A0A422N1T5|A0A422N1T5_TRYRA    GRKYSDSTVQKDKKLLSYEVVADKDGKPKVQVEVS-GKKKQFTPEEVSAMVLQKMKEIAE    158
tr|F9WRV9|F9WRV9_TRYVY            GRKYSDPAVQADKKLLSYEVVADKDGKPKVQVTLG-GKKKEFTPEEVSAMVLQKMKEIAE    162
tr|F9W3P7|F9W3P7_TRYCI            GRKYTDAAVQADKKLLSYEVVADRDGKPKVQVMVG-GKPKQFTPEEISAMVLQKMKEIAE    161
tr|Q384Q5|Q384Q5_TRYB2            GRKYTDAAVQADKKLLSYEVIADRDGKPKVQVMVG-GKKKQFTPEEISAMVLQKMKEIAE    161
tr|A0A1Y2AI48|A0A1Y2AI48_9FUNG    GRRFDEQDVQNDMKHFPFKVVA-KDGKPIIQVKVK-GEDKKFTPEEISAMVLQKMKEIAE    158
tr|A0A1Y1X8R1|A0A1Y1X8R1_9FUNG    GRKYDEPAVQKDLKHFPFNVIK-KDEKPYVKVNVK-GTDKTFSPEEISAMILQKMKNIAE    163
sp|P11021|BIP_HUMAN               GRTWNDPSVQQDIKFLPFKVVE-KKTKPYIQVDIGGGQTKTFAPEEISAMVLTKMKETAE    158
tr|A0A0G4EPG2|A0A0G4EPG2_VITBC    GRKYKDTEVQRDKKLLPFILAE-KEGKPFIEVQVK-GETKSFAPEEISAMVLVKMKEVAE    157
tr|M1BLB0|M1BLB0_SOLTU            GRKFEDKEVQRDMKLVPYKIVN-RDGKPYIQVKIKDGETKVFSPEEVSAMILIKMKETAE    165
sp|Q9LKR3|BIP1_ARATH              GRKFEDKEVQKDRKLVPYQIVN-KDGKPYIQVKIKDGETKVFSPEEISAMILTKMKETAE    164
tr|I1KPN3|I1KPN3_SOYBN            GRKFEDKEVQKDMKLVPYKIVN-KDGKPYIQVKIKDGETKVFSPEEISAMVLIKMKETAE    164
                                  ** : :  ** * * . : :      :* :.*  *  : :: **:*:*:* ***: :*

tr|A0A0L1KCY6|A0A0L1KCY6_9EUGL    TYLGTTVKNAVVTVPAYFNDAQRQSTKDAGAIAGLNVARIINEPTAAAIAYGLDKK-GER    213
tr|A0A0S4JK39|A0A0S4JK39_BODSA    TYLGEKVKNAVVTVPAYFNDAQRQSTKDAGTIAGLNVVRIINEPTAAALAYGLNEA-GER    228
tr|A0A7G2CL24|A0A7G2CL24_9TRYP    TYIGEKVKNAVVTVPAYFNDAQRQSTKDAGTIAGLNVVRIINEPTAAALAYGLNKV-GEK    223
tr|A0A0S4IPK6|A0A0S4IPK6_BODSA    TYLGEKVKNAVVTVPAYFNDAQRQSTKDAGTIAGLNVVRIINEPTAAAIAYGLNKA-GEK    221
tr|A0A0N0DZK2|A0A0N0DZK2_LEPPY    TFLGEKVKNAVVTVPAYFNDAQRQATKDAGKIAGLNVVRIINEPTAAAIAYGLNKA-GEK    223
tr|A4I3J9|A4I3J9_LEIIN            TFLGEKVKNAVVTVPAYFNDAQRQATKDSGKIAGLNVVRIINEPTAAAIAYGLNKA-GEK    223
tr|Q4Q8E6|Q4Q8E6_LEIMA            TFLGEKVKNAVVTVPAYFNDAQRQATKDSGKIAGLNVVRIINEPTAAAIAYGLNKA-GEK    223
tr|A4HGG7|A4HGG7_LEIBR            TFLGEKVKNAVVTVPAYFNDAQRQATKDSGKIAGLNVVRIINEPTAAAIAYGLNKA-GEK    223
tr|S9U467|S9U467_9TRYP            TYLGEKVKNAVVTVPAYFNDAQRQSTKDAGTIAGLNVVRIINEPTAAAIAYGLNKA-GEK    292
tr|S9W3A0|S9W3A0_9TRYP            TYLGEKVKNAVVTVPAYFNDAQRQSTKDAGTIAGLNVVRIINEPTAAAIAYGLNKA-GEK    222
tr|A0A1X0NYK3|A0A1X0NYK3_9TRYP    TYLGEKVKNAVVTVPAYFNDAQRQSTKDAGTIAGLNVVRIINEPTAAAIAYGLNKA-GEK    220
tr|Q4D620|Q4D620_TRYCC            TYLGEKVKNAVVTVPAYFNDAQRQSTKDAGTIAGLNVVRIINEPTAAAIAYGLNKA-GEK    215
tr|K2NLB6|K2NLB6_TRYCR            TYLGEKVKNAVVTVPAYFNDAQRQSTKDAGTIAGLNVVRIINEPTAAAIAYGLNKA-GEK    215
tr|A0A422P6Q7|A0A422P6Q7_9TRYP    TYLGEKVKNAVVTVPAYFNDAQRQSTKDAGTIAGLNVVRIINEPTAAAIAYGLNKA-GEK    217
tr|A0A422N1T5|A0A422N1T5_TRYRA    TYLGEKVKNAVVTVPAYFNDAQRQSTKDAGTIAGLNVVRIINEPTAAAIAYGLNKA-GEK    217
tr|F9WRV9|F9WRV9_TRYVY            TYLGEKVKNAVVTVPAYFNDAQRQSTKDAGTIAGLNVVRIINEPTAAAIAYGLNKA-GEK    221
tr|F9W3P7|F9W3P7_TRYCI            TYLGEKVKNAVVTVPAYFNDAQRQSTKDAGTIAGLNVVRIINEPTAAAIAYGLNKA-GEK    220
tr|Q384Q5|Q384Q5_TRYB2            TYLGEKVKNAVVTVPAYFNDAQRQSTKDAGTIAGLNVVRIINEPTAAAIAYGLNKA-GEK    220
tr|A0A1Y2AI48|A0A1Y2AI48_9FUNG    SYLGEKVTHAVVTVPAYFNDAQRQATKDAGTIAGLTVARIINEPTAAAIAYGLDKTGSEK    218
tr|A0A1Y1X8R1|A0A1Y1X8R1_9FUNG    SYLGKSVGHAVVTVPAYFNDAQRQATKDAGTIAGLNILRVINEPTAAAIAYGLDKTGGEK    223
sp|P11021|BIP_HUMAN               AYLGKKVTHAVVTVPAYFNDAQRQATKDAGTIAGLNVMRIINEPTAAAIAYGLDKREGEK    218
tr|A0A0G4EPG2|A0A0G4EPG2_VITBC    AYLGKDVKHAVITVPAYFNDAQRQATKDAGTISGLNVIRIINEPTAAAIAYGMDKK-QEK    216
tr|M1BLB0|M1BLB0_SOLTU            AFLGKTIKDAVVTVPAYFNDAQRQATKDAGVIAGLNVARIINEPTAAAIAYGLDKKGGEK    225
sp|Q9LKR3|BIP1_ARATH              AYLGKKIKDAVVTVPAYFNDAQRQATKDAGVIAGLNVARIINEPTAAAIAYGLDKKGGEK    224
tr|I1KPN3|I1KPN3_SOYBN            AFLGKKINDAVVTVPAYFNDAQRQATKDAGVIAGLNVARIINEPTAAAIAYGLDKKGGEK    224
                                  :::*  : .**:************:***:* *:**.: *:********:***::    *:

tr|A0A0L1KCY6|A0A0L1KCY6_9EUGL    NILVFDLGGGTFDVSLLTIDDGFFEVIATNGDTHLGGEDFDNRMMKHFVDLIKKKSKIDI    273
tr|A0A0S4JK39|A0A0S4JK39_BODSA    NVLVFDLGGGTFDVSLLTIDDGFFEVLAVNGDTHLGGEDFDNNMMRHFVEGLRKKQGIDI    288
tr|A0A7G2CL24|A0A7G2CL24_9TRYP    NILVFDLGGGTFDVSLLTIDGDFFEVVATNGDTHLGGEDFDNNIMRYYVDNLKRKNSVDI    283
```

```
tr|A0A0S4IPK6|A0A0S4IPK6_BODSA      NILVFDLGGGTFDVSLLTIDDGFFEVVATNGDTHLGGEDFDNNMMRFYVDNLKKKKNIDI    281
tr|A0A0N0DZK2|A0A0N0DZK2_LEPPY      NILVFDLGGGTFDVSLLTIDEGFFEVVATNGDTHLGGEDFDNNMMKFFVDGLKRKQSIDI    283
tr|A4I3J9|A4I3J9_LEIIN              NILVFDLGGGTFDVSLLTIDEGFFEVVATNGDTHLGGEDFDNNMMKFFVDGLKRKQNVDI    283
tr|Q4Q8E6|Q4Q8E6_LEIMA              NILVFDLGGGTFDVSLLTIDEGFFEVVATNGDTHLGGEDFDNNMMKFFVDGLKRKQNVDI    283
tr|A4HGG7|A4HGG7_LEIBR              NILVFDLGGGTFDVSLLTIDEGFFEVVATNGDTHLGGEDFDNNMMKFFVDGLKRKQNVDI    283
tr|S9U467|S9U467_9TRYP              NILVFDLGGGTFDVSLLTIDEGFFEVVATNGDTHLGGEDFDNNLMRYFVDGLKRKKSIDI    352
tr|S9W3A0|S9W3A0_9TRYP              NILVFDLGGGTFDVSLLTIDEGFFEVVATNGDTHLGGEDFDNNLMRYFVDGLKRKKSIDI    282
tr|A0A1X0NYK3|A0A1X0NYK3_9TRYP      NILVFDLGGGTFDVSLLTIDEGFFEVVATNGDTHLGGEDFDNNMMRYFVDMLKKKKNVDV    280
tr|Q4D620|Q4D620_TRYCC              NILVFDLGGGTFDVSLLTIDEGFFEVVATNGDTHLGGEDFDNNMMRYFVDMLKKKKNVDV    275
tr|K2NLB6|K2NLB6_TRYCR              NILVFDLGGGTFDVSLLTIDEGFFEVVATNGDTHLGGEDFDNNMMRYFVDMLKKKKNVDV    275
tr|A0A422P6Q7|A0A422P6Q7_9TRYP      NILVFDLGGGTFDVSLLTIDEGFFEVVATNGDTHLGGEDFDNNMMRYFVDALKKKKGVDI    277
tr|A0A422N1T5|A0A422N1T5_TRYRA      NILVFDLGGGTFDVSLLTIDEGFFEVVATNGDTHLGGEDFDNNMMRYFVDMLKKKKGVDI    277
tr|F9WRV9|F9WRV9_TRYVY              NILVFDLGGGTFDVSLLTIDEGFFEVVATNGDTHLGGEDFDNNMMRYFVDMLKKKRNVDV    281
tr|F9W3P7|F9W3P7_TRYCI              NILVFDLGGGTFDVSLLTIDEGFFEVVATNGDTHLGGEDFDNNMMRYFVDMLKKKKNVDI    280
tr|Q384Q5|Q384Q5_TRYB2              NILVFDLGGGTFDVSLLTIDEGFFEVVATNGDTHLGGEDFDNNMMRHFVDMLKKKKNVDI    280
tr|A0A1Y2AI48|A0A1Y2AI48_9FUNG      NILVYDLGGGTFDVSVLTIDDGVFEVLATNGDTHLGGEDFDNRLIEHFTKSYKKKFKKDC    278
tr|A0A1Y1X8R1|A0A1Y1X8R1_9FUNG      NILVYDLGGGTFDVSLLSIDDGVFEVLATSGDTHLGGEDFDNRVIEYLTKQYQRKAKVDV    283
sp|P11021|BIP_HUMAN                 NILVFDLGGGTFDVSLLTIDNGVFEVVATNGDTHLGGEDFDQRVMEHFIKLYKKKTGKDV    278
tr|A0A0G4EPG2|A0A0G4EPG2_VITBC      NILVYDLGGGTFDVSLLTIDNGVFEVVATSGDTHLGGEDFDQRVMDHFIKVIKKKYDKDI    276
tr|M1BLB0|M1BLB0_SOLTU              NILVFDLGGGTFDVSILTIDNGVFEVLATNGDTHLGGEDFDQRIMEYFIKLIKKKHGKDI    285
sp|Q9LKR3|BIP1_ARATH                NILVFDLGGGTFDVSVLTIDNGVFEVLSTNGDTHLGGEDFDHRVMEYFIKLIKKKHQKDI    284
tr|I1KPN3|I1KPN3_SOYBN              NILVFDLGGGTFDVSILTIDNGVFEVLATNGDTHLGGEDFDQRIMEYFIKLIKKKHGKDI    284
                                    *:**:*********:*:** ..***::..**********:.:: .  .  ::*   *

tr|A0A0L1KCY6|A0A0L1KCY6_9EUGL      SKDQKALARLRKASEAAKRQLSSQPEAHVEVDGLVEGYDFSEKISRAKFEELSMDLFKST    333
tr|A0A0S4JK39|A0A0S4JK39_BODSA      SKDQRALARLRKACEAAKRQLSSQPEARVEVDSLVEGHDFSERVTRSKFEELNMALFKGT    348
tr|A0A7G2CL24|A0A7G2CL24_9TRYP      SKDQKALARLRKACEAAKRQLSSHPEARVEVDSLVEGFDFSEKLTRAKFEELNMDLFKNT    343
tr|A0A0S4IPK6|A0A0S4IPK6_BODSA      STDQKALARLRKACEAAKRQLSSQPEARVEVDSLVEGYDFSEKMTRSKFEELNMALFKGT    341
tr|A0A0N0DZK2|A0A0N0DZK2_LEPPY      SNDQKALARLRKACEAAKRQLSSHPEARVEVDSLVEGHDFSEKITRAKFEELNMAMFKNT    343
tr|A4I3J9|A4I3J9_LEIIN              SNDQKALARLRKACEAAKRQLSSHPEARVEVDSLVEGYDFSEKITRAKFEELNMALFKNT    343
tr|Q4Q8E6|Q4Q8E6_LEIMA              SNDQKALARLRKACEAAKRQLSSHPEARVEVDSLVEGYDFSEKITRAKFEELNMALFKNT    343
tr|A4HGG7|A4HGG7_LEIBR              SGDQKALARLRKACEAAKRQLSSHPEARVEVDSLVEGHDFSEKITRAKFEELNMALFKNT    343
tr|S9U467|S9U467_9TRYP              SKDLKALARLRKACEAAKRQLSSHPEARVEVDSLTEGFDFSEKITRAKFEELNMEMFKGT    412
tr|S9W3A0|S9W3A0_9TRYP              SKDLKALARLRKACEAAKRQLSSHPEARVEVDSLTEGFDFSEKITRAKFEELNMEMFKGT    342
tr|A0A1X0NYK3|A0A1X0NYK3_9TRYP      SKDQKALARLRKACEAAKRQLSSHPEARVEVDSLTEGFDFSEKITRAKFEELNMELFKGT    340
tr|Q4D620|Q4D620_TRYCC              SKDQKALARLRKACEAAKRQLSSHPEARVEVDSLTEGFDFSEKITRAKFEELNMELFKGT    335
tr|K2NLB6|K2NLB6_TRYCR              SKDQKALARLRKACEAAKRQLSSHPEARVEVDSLTEGFDFSEKITRAKFEELNMELFKGT    335
tr|A0A422P6Q7|A0A422P6Q7_9TRYP      GKDQKALARLRKACEAAKRQLSSHPEARVEVDSLTEGFDFSEKITRAKFEELNMELFKGT    337
tr|A0A422N1T5|A0A422N1T5_TRYRA      GKDQKALARLRKACEAAKRQLSSHPEARVEVDSLTEGFDFSEKITRAKFEELNMELFKGT    337
tr|F9WRV9|F9WRV9_TRYVY              SKDQKALARLRKACEAAKRQLSSHPEARVEVDSLTEGFDFSEKITRAKFEELNMDLFKGT    341
tr|F9W3P7|F9W3P7_TRYCI              SKDQKALARLRKACEAAKRQLSSHPEARVEVDSLTEGFDFSEKITRAKFEELNMDLFKGT    340
tr|Q384Q5|Q384Q5_TRYB2              SKDQKALARLRKACEAAKRQLSSHPEARVEVDSLTEGFDFSEKITRAKFEELNMDLFKGT    340
tr|A0A1Y2AI48|A0A1Y2AI48_9FUNG      TKDLKSMGKLKREVEKAKRALSSQMSVKVEIEAFYDGDDFSETLTRAKFEELNIDLFKKT    338
tr|A0A1Y1X8R1|A0A1Y1X8R1_9FUNG      SKDQRAIGKLKREVEKAKRTLSSQMSVRIEIESFYQGEDFSESLTRAKFEELNMDLFKKT    343
sp|P11021|BIP_HUMAN                 RKDNRAVQKLRREVEKAKRALSSQHQARIEIESFYEGEDFSETLTRAKFEELNMDLFRST    338
tr|A0A0G4EPG2|A0A0G4EPG2_VITBC      SKDKRALQKLRREVEKGKRALSSTHQARIEIESLVEGVDFSETLTRARFEELNNDLFQKT    336
tr|M1BLB0|M1BLB0_SOLTU              SKDNKALGKLRREAERAKRALSSQHQVRVEIESLFDGVDFSEPLTRARFEELNNDLFRKT    345
sp|Q9LKR3|BIP1_ARATH                SKDNKALGKLRRECERAKRALSSQHQVRVEIESLFDGVDFSEPLTRARFEELNNDLFRKT    344
tr|I1KPN3|I1KPN3_SOYBN              SKDNRALGKLRREAERAKRALSSQHQVRVEIESLFDGVDFSEPLTRARFEELNNDLFRKT    344
                                    * ::: :*::  * .** ***  ..::*::.: :* **** ::*::****.  :*: *

tr|A0A0L1KCY6|A0A0L1KCY6_9EUGL      LKPVEAVLADAKLKKSDIEDIVLVGGSTRIPKVQQLLKDFFNGKELNKGINPDEAVAYGA    393
tr|A0A0S4JK39|A0A0S4JK39_BODSA      LEPVRKVLEDAKLKKGDIDEIVLVGGSTRIPKVQQLIRDFFN-KEPNRGVNPDEAVAFGA    407
tr|A0A7G2CL24|A0A7G2CL24_9TRYP      LIPVQKVLEDARMKKTDIDEIVLVGGSTRIPKVQQLIKDFFGGKEPNKGINPDEAVAYGA    403
tr|A0A0S4IPK6|A0A0S4IPK6_BODSA      LEPVRKVLEDSKLKKSEIDEIVLVGGSTRIPKVQQLIRDFFN-KEPNKGINPDEAVAYGA    400
tr|A0A0N0DZK2|A0A0N0DZK2_LEPPY      LIPVQKVLEDAKLKKSDIDEIVLVGGSTRIPKVQQLIKDFFGGKEPNKGINPDEAVAYGA    403
tr|A4I3J9|A4I3J9_LEIIN              LVPVQKVLEDAKLKKSDIDEIVLVGGSTRIPKVQQLIKDFFSGKEPNKGINPDEAVAYGA    403
tr|Q4Q8E6|Q4Q8E6_LEIMA              LVPVQKVLEDAKLKKSDIDEIVLVGGSTRIPKVQQLIKDFFSGKEPNKGINPDEAVAYGA    403
tr|A4HGG7|A4HGG7_LEIBR              LLPVQKVLEDAKLKKSDIDEIVLVGGSTRIPKVQQLIKDFFGGKEPNKGINPDEAVAYGA    403
tr|S9U467|S9U467_9TRYP              LVPVQKVLEDAKLKKSDIDEIVLVGGSTRIPKVQQLIKDFFGGKEPNKGINPDEAVAYGA    472
tr|S9W3A0|S9W3A0_9TRYP              LVPVQKVLEDAKLKKSDIDEIVLVGGSTRIPKVQQLIKDFFGGKEPNKGINPDEAVAYGA    402
tr|A0A1X0NYK3|A0A1X0NYK3_9TRYP      LVPVQRVLEDAKLKKSDIDEIVLVGGSTRVPKVQQLIRDFFGGKEPNRGINPDEAVAYGA    400
tr|Q4D620|Q4D620_TRYCC              LVPVQRVLEDAKLKKSDIHEIVLVGGSTRVPKVQQLIRDFFGGKEPNRGINPDEAVAYGA    395
tr|K2NLB6|K2NLB6_TRYCR              LVPVQRVLEDAKLKKSDIHEIVLVGGSTRVPKVQQLIRDFFGGKEPNRGINPDEAVAYGA    395
tr|A0A422P6Q7|A0A422P6Q7_9TRYP      LVPVQRVLEDAKLKKSDIDEIVLVGGSTRVPKVQQLIRDFFGGKEPNRGINPDEAVAYGA    397
tr|A0A422N1T5|A0A422N1T5_TRYRA      LVPVQRVLEDAKLKKSEIDEIVLVGGSTRVPKVQQLIRDFFGGKEPNRGINPDEAVAYGA    397
tr|F9WRV9|F9WRV9_TRYVY              LVPVQRVLEDAKLKKSDIHEIVLVGGSTRVPKVQQLISDFFGGKELNRGINPDEAVAYGA    401
tr|F9W3P7|F9W3P7_TRYCI              LIPVQRVLEDAKLKKSDIHEIVLVGGSTRVPKVQQLISDFFGGKELNRGINPDEAVAYGA    400
tr|Q384Q5|Q384Q5_TRYB2              LVPVQRVLEDAKLKKSDIHEIVLVGGSTRVPKVQQLISDFFGGKELNRGINPDEAVAYGA    400
tr|A0A1Y2AI48|A0A1Y2AI48_9FUNG      LKPVEKVMKDAGLDKHEIHDIVLVGGSTRIPKVVQLLEDYFNGKKASKGINPDEAVAYGA    398
tr|A0A1Y1X8R1|A0A1Y1X8R1_9FUNG      IRPVEKVLKDAGLGKHEIHDIVLVGGSTRIPKIQQLIEDFFNGKKASKGINPDEAVAYGA    403
sp|P11021|BIP_HUMAN                 MKPVQKVLEDSDLKKSDIDEIVLVGGSTRIPKIQQLVKEFFNGKEPSRGINPDEAVAYGA    398
tr|A0A0G4EPG2|A0A0G4EPG2_VITBC      LKPVKNVLDDAGFKKTQIDEIVLVGGSTRIPKIQQLIKDFFNGKEPNRGINPDEAVAYGA    396
tr|M1BLB0|M1BLB0_SOLTU              MTPVKKAMEDAGLAKNQIDEIVLVGGSTRIPKVQQLLKDYFDGKEPSKGVNPDEAVAFGA    405
sp|Q9LKR3|BIP1_ARATH                MGPVKKAMDDAGLQKSQIDEIVLVGGSTRIPKVQQLLKDFFEGKEPNKGVNPDEAVAYGA    404
tr|I1KPN3|I1KPN3_SOYBN              MGPVKKAMEDAGLQKSQIDEIVLVGGSTRIPKVQQLLKDYFDGKEPNKGVNPDEAVAYGA    404
                                    : **. .: *: : * :*.:*********:**: **: ::*  *: .:*:*******:**

tr|A0A0L1KCY6|A0A0L1KCY6_9EUGL      AVQGAVLCGDS-AVKDKVLLVDVIPLSLGIETVGGVMTKLVERNTQIPAKKSQIFSTASD    452
tr|A0A0S4JK39|A0A0S4JK39_BODSA      AVQAAVLTGAR-DFKNTVVVADVVPLSLGIETVGGVMTRVIERNTQIPTKKSQTFTTYED    466
tr|A0A7G2CL24|A0A7G2CL24_9TRYP      AVQAAVMTGES-EVGGKVLLVDVISLSLGIETVGGVMTKLVERNTQIPAKKSQVFSTYQD    462
tr|A0A0S4IPK6|A0A0S4IPK6_BODSA      AVQAAVLTGDS-DLGNKVVLVDVIPLSLGIETVGGVMTKLIERNTQIPTKKSQVFSTYQD    459
tr|A0A0N0DZK2|A0A0N0DZK2_LEPPY      AVQAAVLMGES-EVGGKVVLVDVIPLSLGIETVGGVMTKLIERNTQIPTKKSQVFSTYQD    462
tr|A4I3J9|A4I3J9_LEIIN              AVQAAVLTGES-EVGGKVVLVDVIPLSLGIETVGGVMTKLIERNTQIPTKKSQIFSTYQD    462
tr|Q4Q8E6|Q4Q8E6_LEIMA              AVQAAVLTGES-EVGGKVVLVDVIPLSLGIETVGGVMTKLIERNTQIPTKKSQIFSTYQD    462
tr|A4HGG7|A4HGG7_LEIBR              AVQAAVLTGES-EVGGKVVLVDVIPLSLGIETVGGVMTKLIERNTQIPTKKSQVFSTYQD    462
tr|S9U467|S9U467_9TRYP              AVQAAVLTGES-EVGGKVVLVDVIPLSLGIETVGGVMTKLIERNTQIPTKKSQVFSTYQD    531
```

```
tr|S9W3A0|S9W3A0_9TRYP            AVQAAVLTGES-EVGGKVVLVDVIPLSLGIETVGGVMTKLIERNTQIPTKKSQVFSTYQD    461
tr|A0A1X0NYK3|A0A1X0NYK3_9TRYP    AVQAAVLTGES-EVGGRVVLVDVIPLSLGIETVGGVMTKLIERNTQIPTKKSQVFSTYQD    459
tr|Q4D620|Q4D620_TRYCC            AVQAAVLTGES-EVGGRVVLVDVIPLSLGIETVGGVMTKLIERNTQIPTKKSQVFSTYQD    454
tr|K2NLB6|K2NLB6_TRYCR            AVQAAVLTGES-EVGGRVVLVDVIPLSLGIETVGGVMTKLIERNTQIPTKKSQVFSTYQD    454
tr|A0A422P6Q7|A0A422P6Q7_9TRYP    AVQAAVLTGES-EVGGRVVLVDVIPLSLGIETVGGVMTKLIERNTQIPTKKSQVFSTYQD    456
tr|A0A422N1T5|A0A422N1T5_TRYRA    AVQAAVLTGES-EVGGRVVLVDVIPLSLGIETVGGVMTKLIERNTQIPTKKSQVFSTYQD    456
tr|F9WRV9|F9WRV9_TRYVY            AVQAAVLTGES-EVGGRVVLVDVIPLSLGIETVGGIMTKLIERNTQIPTKKSQVFSTHAD    460
tr|F9W3P7|F9W3P7_TRYCI            AVQAAVLTGES-EVGGRVVLVDVIPLSLGIETVGGVMTKLIERNTQIPTKKSQIFSTHAD    459
tr|Q384Q5|Q384Q5_TRYB2            AVQAAVLTGES-EVGGRVVLVDVIPLSLGIETVGGVMTKLIERNTQIPTKKSQVFSTHAD    459
tr|A0A1Y2AI48|A0A1Y2AI48_9FUNG    AVQGGVLSGNS-DELNNVLLLDVNPLTLGIETTGGVMTKLIARNTQIPTKKSQIFSTAAD    457
tr|A0A1Y1X8R1|A0A1Y1X8R1_9FUNG    AVQGGVLSGEDKENLSNVLLLDVNPLTLGIETTGGVMTKLIGRNTQIPTKKSQIFSTAAD    463
sp|P11021|BIP_HUMAN               AVQAGVLSGD--QDTGDLVLLDVCPLTLGIETVGGVMTKLIPRNTVVPTKKSQIFSTASD    456
tr|A0A0G4EPG2|A0A0G4EPG2_VITBC    AVQAGILSGEGGQ---ELLLLDVTPLTLGIETVGGVMTKLINRNTVIPTKKSQVFSTYQD    453
tr|M1BLB0|M1BLB0_SOLTU            AVQGGILSGEGGDETKDILLLDVAPLTLGIETVGGVMTKLIPRNTVIPTKKSQVFTTYQD    465
sp|Q9LKR3|BIP1_ARATH              AVQGGILSGEGGDETKDILLLDVAPLTLGIETVGGVMTKLIPRNTVIPTKKSQVFTTYQD    464
tr|I1KPN3|I1KPN3_SOYBN            AVQGSILSGEGGEETKDILLLDVAPLTLGIETVGGVMTKLIPRNTVIPTKKSQVFTTYQD    464
                                  ***..:: *        ::: **  *:*****.**:**::: *** :*:**** *:*  *

tr|A0A0L1KCY6|A0A0L1KCY6_9EUGL    NQPSVTIVVYEGERAMSKDNRELGKFDLGGITPAPRGSPQIEVTFDVDENGIMEVSAKDN    512
tr|A0A0S4JK39|A0A0S4JK39_BODSA    NQPGVLIQVFEGERQMTKDNRMLGKFELTGIRRAAKGVPQIEVTFDVDEGSILQVSATDK    526
tr|A0A7G2CL24|A0A7G2CL24_9TRYP    NQPGVLIQVFEGERQMTKDNRLLGKFELSGIPPAPRGVPQIEVSFDVDENSILQVSAVDK    522
tr|A0A0S4IPK6|A0A0S4IPK6_BODSA    NQPGVLIQVFEGERQMTKDNRLLGKFELSGIPPAPRGTPQIEVTFDVDENSILQVSATDK    519
tr|A0A0N0DZK2|A0A0N0DZK2_LEPPY    NQPGVLIQVFEGERQMTKDNRLLGKFELSGIPPAPRGVPQIEVSFDVDENSILQVTASDK    522
tr|A4I3J9|A4I3J9_LEIIN            NQPSVLIQVFEGERGMTKDNRLLGKFDLSGIPPAPRGVPQIEVAFDVDENSILQVTASDK    522
tr|Q4Q8E6|Q4Q8E6_LEIMA            NQPSVLIQVFEGERGMTKDNRLLGKFDLSGIPPAPRGVPQIEVAFDVDENSILQVTASDK    522
tr|A4HGG7|A4HGG7_LEIBR            NQPGVLIQVFEGERQMTKDNRLLGKFELSGIPPAPRGVPQIEVAFDVDENSILQVTASDK    522
tr|S9U467|S9U467_9TRYP            NQPGVLIQVFEGERQMTKDNRLLGKFELSGIPPAPRGVPQIEVSFDVDENSILQVGAVDK    591
tr|S9W3A0|S9W3A0_9TRYP            NQPGVLIQVFEGERQMTKDNRLLGKFELSGIPPAPRGVPQIEVSFDVDENSILQVGAVDK    521
tr|A0A1X0NYK3|A0A1X0NYK3_9TRYP    NQPGVLIQVFEGERQMTKDNRLLGKFELSGIPPAARGVPQIEVTFDVDENSILQVSAVDK    519
tr|Q4D620|Q4D620_TRYCC            NQPGVLIQVYEGERQMTKDNRLLGKFELSGIPPAPRGVPQIEVTFDVDENSILQVSAVDK    514
tr|K2NLB6|K2NLB6_TRYCR            NQPGVLIQVYEGERQMTKDNRLLGKFELSGIPPAPRGVPQIEVTFDVDENSILQVSAVDK    514
tr|A0A422P6Q7|A0A422P6Q7_9TRYP    NQPGVLIQVFEGERQMTKDNRLLGKFELSGIPPAARGVPQIEVTFDVDENSILQVSAVDK    516
tr|A0A422N1T5|A0A422N1T5_TRYRA    NQPGVLIQVFEGERQMTKDNRLLGKFELSGIPPAARGVPQIEVTFDVDENSILQVSAVDK    516
tr|F9WRV9|F9WRV9_TRYVY            NQPGVLIQVYEGERQLTKDNRLLGKFELSGIPPAPRGVPQIEVTFDVDENSILQVSAVDK    520
tr|F9W3P7|F9W3P7_TRYCI            NQPGVLIQVYEGERQLTKDNRLLGKFELTGIPPAARGVPQIEVTFDVDENSILQVSAMDK    519
tr|Q384Q5|Q384Q5_TRYB2            NQPGVLIQVYEGERQLTKDNRLLGKFELSGIPPAARGVPQIEVTFDVDENSILQVSAMDK    519
tr|A0A1Y2AI48|A0A1Y2AI48_9FUNG    NQPTVLIQVFEGERPLTKDNNLLGKFDLNGIPPAPRGTPQIEVTFEISVDGLLRVAAHDK    517
tr|A0A1Y1X8R1|A0A1Y1X8R1_9FUNG    NQSIVLIQVYEGERSMTKDNNLLGKFELKDIPPAPRGVPQIEVTFEIDVNGILKVSAEDK    523
sp|P11021|BIP_HUMAN               NQPTVTIKVYEGERPLTKDNHLLGTFDLTGIPPAPRGVPQIEVTFEIDVNGILRVTAEDK    516
tr|A0A0G4EPG2|A0A0G4EPG2_VITBC    NQPAVMIQVFEGERPMTKDNHLLGKFELSGIPPAPRGVPQIEVTFEIDSNGILNVSAEDK    513
tr|M1BLB0|M1BLB0_SOLTU            QQTTVTISVFEGERSMVKDCRLLGKFDLTGIPPAPRGTPQIEVTFEVDANGILNVKAEDK    525
sp|Q9LKR3|BIP1_ARATH              QQTTVSIQVFEGERSLTKDCRLLGKFDLNGIPPAPRGTPQIEVTFEVDANGILNVKAEDK    524
tr|I1KPN3|I1KPN3_SOYBN            QQTTVSIQVFEGERSLTKDCRLLGKFDLSGIPPAPRGTPQIEVTFEVDANGILNVKAEDK    524
                                  :*  * * *:**** : ** . **.*:* .*  * :* *****:*::. ..::.* * *:

tr|A0A0L1KCY6|A0A0L1KCY6_9EUGL    STNKEEKITITNDKGRLSQEEIDRMVREAEEHGEEDRIKKETIEARNHLESIAYSLKNAV    572
tr|A0A0S4JK39|A0A0S4JK39_BODSA    SSNTKEEITIRNDKGRLSQREIDEMVREAELFEEEDRKLRGKVEARTALESAVYSLKGEL    586
tr|A0A7G2CL24|A0A7G2CL24_9TRYP    SSGKREEIRISNDKGRLTDEEIDAMIREAAQFEEEDRKVRERVEARNSLEGIAYSLRNQV    582
tr|A0A0S4IPK6|A0A0S4IPK6_BODSA    SSNKREEITITNDKGRLSEEEIERMVREAAEFEEEDRKLREKVEARNSLESVAYSLKNQI    579
tr|A0A0N0DZK2|A0A0N0DZK2_LEPPY    SSGKREEITITNDKGRLSEAEIEAMVEEAAQFAEEDRKLRERVEAKNSLESIAYSLRNQV    582
tr|A4I3J9|A4I3J9_LEIIN            SSGKREEITITNDKGRLSEEEIERMVREAAEFEDEDRKVRERVEAKNSLESIAYSLRNQI    582
tr|Q4Q8E6|Q4Q8E6_LEIMA            SSGKREEITITNDKGRLSEEEIERMVREAAEFEDEDRKVRERVEAKNSLESIAYSLRNQI    582
tr|A4HGG7|A4HGG7_LEIBR            SSGKREEITITNDKGRLSEEEIERMVREAEEFEDEDRKVRERVEAKNSLESIAYSLRNQI    582
tr|S9U467|S9U467_9TRYP            SSGKKEEITITNDKGRLSEEDIERMVREAAENEESDRKVRERVEAKNSLESIAYSLRNQV    651
tr|S9W3A0|S9W3A0_9TRYP            SSGKKEEITITNDKGRLSEEDIERMVREAAENEESDRKVRERVEAKNSLESIAYSLRNQV    581
tr|A0A1X0NYK3|A0A1X0NYK3_9TRYP    SSGKKEEITITNDKGRLSEEEIERMVREAAEFEEEDRKVRERVDARNSLEGIAYSLRTQV    579
tr|Q4D620|Q4D620_TRYCC            SSGKKEEITITNDKGRLSEEEIERMVREAAEFEDEDRKVRERVDARNSLESVAYSLRNQV    574
tr|K2NLB6|K2NLB6_TRYCR            SSGKKEEITITNDKGRLSEEEIERMVREAAEFEDEDRKVRERVDARNSLESTAYSLRNQV    574
tr|A0A422P6Q7|A0A422P6Q7_9TRYP    SSGKKEEITITNDKGRLSEEEIERMVREAAEFEDEDRKVRERVEARNSLESIAYSLRNQV    576
tr|A0A422N1T5|A0A422N1T5_TRYRA    SSGKKEEITITNDKGRLSEEEIERMVREAAEFEDEDRKVRERVEARNSLESIAYSLRNQV    576
tr|F9WRV9|F9WRV9_TRYVY            SSGKKEEITITNDKGRLSEEEIERMVREAAEFENEDRKVRERVDARNTLESVTYSLRSQV    580
tr|F9W3P7|F9W3P7_TRYCI            SSGKKEEITITNDKGRLSEEEIERMVREAAEFEDEDRKVRERVEARNSLESAAYSLRNQV    579
tr|Q384Q5|Q384Q5_TRYB2            SSGKKEEITITNDKGRLSEEEIERMVREAAEFEDEDRKVRERVDARNSLESVAYSLRNQV    579
tr|A0A1Y2AI48|A0A1Y2AI48_9FUNG    GTGKSESITITNDKGRLTEEEIERMVREAEQFAEEDQLLKEKIEAKNGLENYIYQIKQQV    577
tr|A0A1Y1X8R1|A0A1Y1X8R1_9FUNG    GTGKSESITITNDKGRLSEEEIERMVAEAEQFAEEDKLLKEKIEAKNTLENYLYTIKNQV    583
sp|P11021|BIP_HUMAN               GTGNKNKITITNDQNRLTPEEIERMVNDAEKFAEEDKKLKERIDTRNELESYAYSLKNQI    576
tr|A0A0G4EPG2|A0A0G4EPG2_VITBC    GTGKSEKITITNDKGRLSQEDIEKMIKEAEDYAEEDKKVRERVDAKNSFDNYLHSMKQSV    573
tr|M1BLB0|M1BLB0_SOLTU            ASGKSEKITITNDKGRLSQEEIERMVKEAEEFAEEDKKVKERVDARNSLETYVYNMRNQI    585
sp|Q9LKR3|BIP1_ARATH              ASGKSEKITITNEKGRLSQEEIDRMVKEAEEFAEEDKKVKEKIDARNALETYVYNMKNQV    584
tr|I1KPN3|I1KPN3_SOYBN            GTGKSEKITITNEKGRLSQEEIDRMVREAEEFAEEDKKVKERIDARNSLETYVYNMKNQV    584
                                  .:.. :.* * *::.**:  :*: *: :*     :.*:  :  ::::. ::   : ::  :

tr|A0A0L1KCY6|A0A0L1KCY6_9EUGL    NDEEKLGNKLDDSEKETIKEAVRETIEWMDENPT-ADKSDYEERLEKLQSVTNPIIQKVN    631
tr|A0A0S4JK39|A0A0S4JK39_BODSA    GDVTKLVGL-AGDERAALEVAVREAMSFLDENPN-AELEEYAEARERLQSVAGPIMRRVQ    644
tr|A0A7G2CL24|A0A7G2CL24_9TRYP    NDKEKLGGKLSDEDKKAIEGAVKEALDFVDENPN-AEKEDYKTAQEKLQSVTNPIVQKVY    641
tr|A0A0S4IPK6|A0A0S4IPK6_BODSA    NDKDKLKDKLDADDKQAVEDAVKEAINFLDENPN-AEKEEYDEAKEKLQSVTNPIIQKAY    638
tr|A0A0N0DZK2|A0A0N0DZK2_LEPPY    NDKEKLGDKLDADDKKAIEAAVRVALDFVDENPN-ADKEEFEEAREQLQKVTNPIIQKVY    641
tr|A4I3J9|A4I3J9_LEIIN            NDKDKLGDKLAADDKKAIEEAVKDALDFVDENPN-ADREEFEAARTKLQSVTNPIIQKVY    641
tr|Q4Q8E6|Q4Q8E6_LEIMA            NDKDKLGDKLDADDKKAIEEAVKDALDFVDENPN-ADREEFEAARTKLQSVTNPIIQKVY    641
tr|A4HGG7|A4HGG7_LEIBR            NDKDKLGEKLDADDKKAVEEAVKDALAFVDENPN-ADREDFEGAREKLQSVTNPIIQKVY    641
tr|S9U467|S9U467_9TRYP            NDKEKLGGKIDAEDKKAVEDAVKEAIDFVDENPS-AEKEEYDAAREKLQSVTNPIIQKVY    710
tr|S9W3A0|S9W3A0_9TRYP            NDKEKLGGKIDAEDKKAVEDAVKEAIDFVDENPS-AEKEEYDAAREKLQSVTNPIIQKVY    640
tr|A0A1X0NYK3|A0A1X0NYK3_9TRYP    NDKDKLGGKLSADDKSAVEAAVKEAIQFLDENPN-AEKEEYDEAREKLQSVTNPIIQKAY    638
tr|Q4D620|Q4D620_TRYCC            NDKEKLGGKLSADDKSAVEAAVKEAMQFLDDNPN-ADKEEYDEARDKLQSVTNPIIQKVY    633
tr|K2NLB6|K2NLB6_TRYCR            NDKEKLGGKLSAEDKSAVEAAVKEAMQFLDDNPN-ADKEEYDEARDKLQSVTNPIIQKVY    633
tr|A0A422P6Q7|A0A422P6Q7_9TRYP    NDKEKLGGKLSAEDKSAVEDAVKEAIHFLDENPN-ADKEEYDEAREKLQSVTNPIIQKAY    635
tr|A0A422N1T5|A0A422N1T5_TRYRA    NDKEKLGGKLSAEDKSAVEAAVKEAIHFLDENPN-ADKEEYDEAREKLQGVTNPIIQKAY    635
```

```
tr|F9WRV9|F9WRV9_TRYVY            NDKDKLGGKLSADEKSTVEAAVKEAIRFLDENPN-AEKEEYDAAREKLQGVTNPIIQKAY    639
tr|F9W3P7|F9W3P7_TRYCI            NDKDKLGGKLSADDKAAVEAAVKEAIRFLDENPN-AEKEEYKTALDTMQSVTNPIVQKAY    638
tr|Q384Q5|Q384Q5_TRYB2            NDKDKLGGKLDPNDKAAVETAVAEAIRFLDENPN-AEKEEYKTALETLQSVTNPIIQKTY    638
tr|A0A1Y2AI48|A0A1Y2AI48_9FUNG    TDEDKLGGKIDEDDKKAILDAIKEANEWVDANIATATKEDIDEQKSELEAIVNPITSKLY    637
tr|A0A1Y1X8R1|A0A1Y1X8R1_9FUNG    NDEEGLGSKISEDDKKKILDAVEEKITWLDENGNTAVKEDFEEQKSAVESIVNGITSKLY    643
sp|P11021|BIP_HUMAN               GDKEKLGGKLSSEDKETMEKAVEEKIEWLESHQD-ADIEDFKAKKKELEEIVQPIISKLY    635
tr|A0A0G4EPG2|A0A0G4EPG2_VITBC    EDKDKLANKLDEDQKQQILDAVKDGEEWMQEHPE-EDAEEIKAKHKEIEEICNPIISAVY    632
tr|M1BLB0|M1BLB0_SOLTU            NDKDKLADKLESDEKEKIETATKEALEWLDDNQN-AEKEDYEEKLKEVEAVCNPIITAVY    644
sp|Q9LKR3|BIP1_ARATH              NDKDKLADKLEGDEKEKIEAATKEALEWLDENQN-SEKEEYDEKLKEVEAVCNPIITAVY    643
tr|I1KPN3|I1KPN3_SOYBN            SDKDKLADKLESDEKEKIETAVKEALEWLDDNQS-VEKEDYEEKLKEVEAVCNPIISAVY    643
                                  *   *    .::  :  *        ::: :   .:       :: :   *

tr|A0A0L1KCY6|A0A0L1KCY6_9EUGL    QESGSAGGYQSE-----TMEDL------------------    648
tr|A0A0S4JK39|A0A0S4JK39_BODSA    EATAGGAGDEPE-----SVDDL------------------    661
tr|A0A7G2CL24|A0A7G2CL24_9TRYP    QATGGAPDAEAE-----PMDDL------------------    658
tr|A0A0S4IPK6|A0A0S4IPK6_BODSA    RGSGGAGGEEGGADATESMDDL------------------    660
tr|A0A0N0DZK2|A0A0N0DZK2_LEPPY    QAAGGAAGEEEP----DAMDDL------------------    659
tr|A4I3J9|A4I3J9_LEIIN            QGAAGS-GAEEA----DAMDDL------------------    658
tr|Q4Q8E6|Q4Q8E6_LEIMA            QGTAGS-GAEEA----DAMDDL------------------    658
tr|A4HGG7|A4HGG7_LEIBR            QAGGAP-GSEPT----DAMDDL------------------    658
tr|S9U467|S9U467_9TRYP            AAAGGSPDSEAA----EPMDDL------------------    728
tr|S9W3A0|S9W3A0_9TRYP            AAAGGSPDSEAA----EPMDDL------------------    658
tr|A0A1X0NYK3|A0A1X0NYK3_9TRYP    QAGGGAADEE-------KXQSRWPCLRIWVTIKQISARQRQ    672
tr|Q4D620|Q4D620_TRYCC            QSGGGADGDE-------RPEPMDDL---------------    651
tr|K2NLB6|K2NLB6_TRYCR            QSGGGADGDE-------RPEPMDDL---------------    651
tr|A0A422P6Q7|A0A422P6Q7_9TRYP    QAGGASGEEG-------QPEPMDDL---------------    653
tr|A0A422N1T5|A0A422N1T5_TRYRA    QAGGASGEDE-------QPEPMDDL---------------    653
tr|F9WRV9|F9WRV9_TRYVY            QAG--GEKPQ-------PMDDL------------------    652
tr|F9W3P7|F9W3P7_TRYCI            QSAGAGDKPQ-------PMDDL------------------    653
tr|Q384Q5|Q384Q5_TRYB2            QSAGGGDKPQ-------PMDDL------------------    653
tr|A0A1Y2AI48|A0A1Y2AI48_9FUNG    GGSAGG---DGE------DVPDHDEL--------------    654
tr|A0A1Y1X8R1|A0A1Y1X8R1_9FUNG    GQQKGDATMEDE------DIPDHDEL--------------    663
sp|P11021|BIP_HUMAN               GSAGPPPTGEEDTAE-------KDEL--------------    654
tr|A0A0G4EPG2|A0A0G4EPG2_VITBC    GAAGGAGGPTGEE-----DYEAHDEL--------------    653
tr|M1BLB0|M1BLB0_SOLTU            QRSGGAPGGASEDSN--EDDDSHDEL--------------    668
sp|Q9LKR3|BIP1_ARATH              QRSGGAPGGAGGESSTEEEDESHDEL--------------    669
tr|I1KPN3|I1KPN3_SOYBN            QRSGGAPGGAGGEGED--EDDSHDEL--------------    667
```

(overall sequence identity = 0.3105)

10. HYOU1-like HSP70 chaperones

The animal Hypoxia upregulated protein 1 (HYOU1) is a representing member of this chaperone family that is found in almost all eukaryotes studied. All examined kinetoplastids also possess at least a single gene of this group. Although the KDEL signal can be missing in extreme cases, most members of this family present a very well conserved, ancestral retention signal at their C-termini. Note that HYOU1-like chaperones are rather different from BIP family members, despite the shared HSP70 domain core. They seem to have diverged already at the stem of Eukaryota, therefore they should be treated a separate family.

```
tr|A0A0C9MAJ6|A0A0C9MAJ6_9FUNG    ------------------------------------------------------------    0
sp|Q9Y4L1|HYOU1_HUMAN             -----------------------MADKVRRQRPRRRVCWALVAVL----LADLLALSDTL    33
sp|Q63617|HYOU1_RAT               -----------------------MAATVRRQRPRRLLCWALVAVL----LADLLALSDTL    33
tr|A0A067CBX2|A0A067CBX2_SAPPC    ---------------------------------MRL---SHLLPL----LLAALVATVSA    20
tr|D8RVG4|D8RVG4_SELML            ---------------------------------MRNLVLPALLL------LFSTLAIVQA    21
tr|A0A1P8B8R9|A0A1P8B8R9_ARATH    --------MWLLLYNCRGIAALYSRCLVYDWNMGKIFSWLVVLLS----LISLVPVPSES    48
tr|A0A176WKM4|A0A176WKM4_MARPO    -----------MAPEER---------GRRRWRSMASL--PVVLI------VLCLLRPANG    32
tr|A0A3R7K087|A0A3R7K087_9TRYP    --MNRWRLPGGLLAGRRGEASGRCKAAACCATRAPPLLTAAFFLLTLAS--IVACTAGAY    56
tr|Q4E175|Q4E175_TRYCC            MKMEGEQWMGGVAGVCRGKASHFLKATSCSTTRSPLLLFCIILLLTGTP--FMSFLVEAH    58
tr|Q4E0G9|Q4E0G9_TRYCC            MKMEGEQWMGGVAGVRRGKASHFLKATSCSTTRSLLLLFCIILLLTGTP--FMSLLVEAH    58
tr|A0A6A5CDJ2|A0A6A5CDJ2_NAEFO    ---------------------MF-GFTTPRSSGVGTLISVALFVIIL--SYILVEPSHQG    36
tr|A0A0S4IUZ2|A0A0S4IUZ2_BODSA    ---------------------MQAHKRSPRKRSLPLAM----LMLTVTAVLC--IGCTSG    33
tr|A0A0L1KXM1|A0A0L1KXM1_9EUGL    ---------------------------MSRSLISPVALTL--LSMAIVHELCPPICMVQA    31
tr|A0A0S4JJU2|A0A0S4JJU2_BODSA    ------------------------------------------MLKLLLCA-CILVVVANA    17
tr|A0A0N0VI62|A0A0N0VI62_LEPPY    -----------------------------------MACKNLLLAALMVAVFAILSSTSSA    25
tr|A4HND7|A4HND7_LEIBR            ---------------------------------MKLKYRVVFLAAVLACLFE--PLASLA    25
tr|A4IC10|A4IC10_LEIIN            ---------------------------------MKLKCKVAFLAYVLACLFI--AQASLA    25
tr|E9AFU8|E9AFU8_LEIMA            ---------------------------------MKPKCNVVLLAYALACFFI--TQASLA    25
tr|A0A7G2C9U8|A0A7G2C9U8_9TRYP    ------------------------------------------------------------    0
tr|S9TX72|S9TX72_9TRYP            ----------------------------MKA------LATFLLLA----FAVCFCGVASG    22
tr|S9UBL2|S9UBL2_9TRYP            ----------------------------MKA------LATFLLLA----FAVCFCGVASG    22
tr|Q38E16|Q38E16_TRYB2            ----------------------------MRVLANEIRRMLVAFLL----TAAFGYDVTSA    28
tr|F9W5M9|F9W5M9_TRYCI            ---------------------------------MHLGVGLVLLLV----TATFACDVTFG    23
tr|A0A1X0P4V9|A0A1X0P4V9_9TRYP    -------------------------------------MRIIFFFV----AVF-YLCTISY    18
tr|Q4CS27|Q4CS27_TRYCC            -------------------------------------MQIVSFVI----LAILLSYGASA    19
tr|Q4E4F4|Q4E4F4_TRYCC            -------------------------------------MQIVSFVI----LAILLSYGASA    19
tr|A0A422P1B1|A0A422P1B1_TRYRA    -------------------------------------MRIVFLVI----LTLLLSDGVSA    19
tr|A0A422N0X0|A0A422N0X0_9TRYP    -------------------------------------MRLITLVV----LTLLSSYGVSA    19
```

```
tr|A0A0C9MAJ6|A0A0C9MAJ6_9FUNG    --MSIDYGTEWFKVGLIK--P-GMP----LDVALNKDSKRKTQSVVTIR-----------    40
sp|Q9Y4L1|HYOU1_HUMAN             AVMSVDLGSESMKVAIVK--P-GVP----MEIVLNKESRRKTPVIVTLK-----------    75
sp|Q63617|HYOU1_RAT               AVMSVDLGSESMKVAIVK--P-GVP----MEIVLNKESRRKTPVTVTLK-----------    75
tr|A0A067CBX2|A0A067CBX2_SAPPC    SVAGVDFGGEFFKIALVK--P-GRP----FEIVTNVHSKRKTETIVSFN-----------    62
tr|D8RVG4|D8RVG4_SELML            AVFSVDLGAEWMKVAVVDVKPGQSP----IGVALNEMSKRKSSSVVAFS-----------    66
tr|A0A1P8B8R9|A0A1P8B8R9_ARATH    AVLSVDLGSEWVKVAVVNLKRGQSP----ISVAINEMSKRKSPALVAFQ-----------    93
tr|A0A176WKM4|A0A176WKM4_MARPO    AVFSIDLGSEWMKVAVVNLKPGQAP----IAIAINEMSKRKSPALVAFS-----------    77
tr|A0A3R7K087|A0A3R7K087_9TRYP    HVLAVDLGVEWAKAATLG---GGSGASSNPAVVLNDQANRKSPQCIAFRFLPYDTDDVLQ    113
tr|Q4E175|Q4E175_TRYCC            HILAVDLGVDWAKAATLG---GGSGASLIPTIVLNDQANRKSPQCIAFRFLPYDTDDILQ    115
tr|Q4E0G9|Q4E0G9_TRYCC            HILAVDLGVDWAKAATLG---GGSGASLIPTIVLNDQANRKSPQCIAFRFLPYDTDDILQ    115
tr|A0A6A5CDJ2|A0A6A5CDJ2_NAEFO    PIVGIDLGSRWLKVGIAK---SGSP----IDLVLNEQSKRKTSNIIGFR-----------    78
tr|A0A0S4IUZ2|A0A0S4IUZ2_BODSA    TIVAIDMGSEFLKIAAPK----DTS----IEVCLNEQSHRKSDTWIGFR-----------    74
tr|A0A0L1KXM1|A0A0L1KXM1_9EUGL    QVVGIDLGSEFIKIASVR---RSDG----IDIVLNEETRRKTPHYVGFR-----------    73
tr|A0A0S4JJU2|A0A0S4JJU2_BODSA    NVMGIDFGSEFVKVTSPH---GNAS----IDIVLNEQTRRKSNNFVGFR-----------    59
tr|A0A0N0VI62|A0A0N0VI62_LEPPY    HVIGVDFGSEYIKVSGPH---GDKG----LDIVLNELSMRKTENFIGFR-----------    67
tr|A4HND7|A4HND7_LEIBR            HVIGVDLGSEYIKVAGPH---GDKG----IDIVLNEQSRRKTDNFIGFH-----------    67
tr|A4IC10|A4IC10_LEIIN            HVIGVDLGSEYIKVAGPH---GDKG----VDIVLNEQSRRKTDNFIGFR-----------    67
tr|E9AFU8|E9AFU8_LEIMA            HVIGVDLGSEYIKVAGPH---GDKG----VDIVLNEQSRRKTDNFIGFR-----------    67
tr|A0A7G2C9U8|A0A7G2C9U8_9TRYP    ------------------------------------------------------------    0
tr|S9TX72|S9TX72_9TRYP            HVLGVDFGSEFIKIAAPH---GDQV----VDIVLNELSRRKTENFIGFR-----------    64
tr|S9UBL2|S9UBL2_9TRYP            HVLGVDFGSEFIKIAAPH---GDQV----VDIVLNELSRRKTENFIGFR-----------    64
tr|Q38E16|Q38E16_TRYB2            NVIGVDFGSDYIEVAGPI---NGVN----VDIILNEQSHRKTDNYIGFR-----------    70
tr|F9W5M9|F9W5M9_TRYCI            SVMGIDFGSDYIEIAGPI---NGVN----VNIVLNEQSHRKTDNYIGFR-----------    65
tr|A0A1X0P4V9|A0A1X0P4V9_9TRYP    ANVGIDFGSDYIEVAGPH---TGNN----VDIVLNEQSHRKTDSYIGFR-----------    60
tr|Q4CS27|Q4CS27_TRYCC            NVLGIDFGSEYIEVAGPH---NGNN----VDIVLNEQSHRKTDNYIGFK-----------    61
tr|Q4E4F4|Q4E4F4_TRYCC            NVLGIDFGSEYIEVAGPH---NGNN----VDIVLNEQSHRKTDNYIGFK-----------    61
tr|A0A422P1B1|A0A422P1B1_TRYRA    NVLGIDFGSDYIEVAGPH---NGNN----VDIVLNEQSHRKTDNYIGIK-----------    61
tr|A0A422N0X0|A0A422N0X0_9TRYP    NVLGIDFGSDYIEVAGPH---NGNN----VDIVLNEQSHRKTDNYIGFK-----------    61


tr|A0A0C9MAJ6|A0A0C9MAJ6_9FUNG    HDERIYGGDAISLAGRFPHLTYSNLKSIIAKKYDDPLTQEF----------------R--    82
sp|Q9Y4L1|HYOU1_HUMAN             ENERFFGDSAASMAIKNPKATLRYFQHLLGKQADNPHVALY----------------Q--    117
sp|Q63617|HYOU1_RAT               ENERFLGDSAAGMAIKNPKATLRYFQHLLGKQADNPHVALY----------------R--    117
tr|A0A067CBX2|A0A067CBX2_SAPPC    GDERVYGADAMNIEVRRPQVAYSQIRRFLGATVDHPLVSSL---------------TE--    105
tr|D8RVG4|D8RVG4_SELML            GGNRLLAEEAMGIAARFPERVYSRVRDMVGKPSES--VKRI---------------AS--    107
tr|A0A1P8B8R9|A0A1P8B8R9_ARATH    SGDRLLGEEAAGITARYPNKVYSQLRDMVGKPFKH--VKDF---------------ID--    134
tr|A0A176WKM4|A0A176WKM4_MARPO    NGDRLLAEEAAGIVARYPERVYSRVRDMIGKPFES--VKQT---------------LD--    118
tr|A0A3R7K087|A0A3R7K087_9TRYP    KVERIFSEQALALEPRFPEHVVCGPSLLAGRGVWRGFVAGSDTGAAA-----QAALAPED    168
tr|Q4E175|Q4E175_TRYCC            RVERFFSEQAQSLEPRFPDQVVCGPSLLAGRGVSRVVTDGNNTGGVTQGSNNKDALSLDE    175
tr|Q4E0G9|Q4E0G9_TRYCC            RVERFFSEQAQSLEPRFPDQVVCGPSLLAGRGVSRVVTDGNNTGGVTQGSNNKDTLSLDE    175
tr|A0A6A5CDJ2|A0A6A5CDJ2_NAEFO    GKDRYIGEAGYTMVARFPDKMLRFLNFALGKSYDLELNEVS---------------RRYN    123
tr|A0A0S4IUZ2|A0A0S4IUZ2_BODSA    GEERFFGADAKSLSARFPDAMIPAVPRLVGVPHDAAFQQGW---------------LN--    117
tr|A0A0L1KXM1|A0A0L1KXM1_9EUGL    GKDSYIGEDAKNLVGRFPDMIFTLLNRLIGLSPTESLRQWY---------------HS--    116
tr|A0A0S4JJU2|A0A0S4JJU2_BODSA    GDDRFFGDEAKNLAPRFPDNMFSMINQLIGFPFESNKTAQF---------------QQ--    102
tr|A0A0N0VI62|A0A0N0VI62_LEPPY    NGDIYIGDTAKGLAARFPLCTASAINQLVSIRKDSELHAVF---------------QD--    110
tr|A4HND7|A4HND7_LEIBR            RSDLYIGDTAKSLAARFPLCTASAVNQLIGIRKDSHLLSFF---------------FD--    110
tr|A4IC10|A4IC10_LEIIN            RSDLYIGDTAKSLAARFPLCTASAVNQLVRIRKDSSLLPFF---------------RD--    110
tr|E9AFU8|E9AFU8_LEIMA            RSDLYIGDTAKSLAARFPLCTASAVNQLVRIRKDSSLLPFF---------------SD--    110
tr|A0A7G2C9U8|A0A7G2C9U8_9TRYP    ----------------------------MGLKKDSPEWKEF---------------ES--    15
tr|S9TX72|S9TX72_9TRYP            NGERYIGNEAKTLAARFPLKIASAINQLIGVQDKTEELKDF---------------EQ--    107
tr|S9UBL2|S9UBL2_9TRYP            NGERYIGNEAKTLAARFPLKIASAINQLIGVQDKTEELKDF---------------EQ--    107
tr|Q38E16|Q38E16_TRYB2            NGERSIGAQAKSLAARFPTNMIAMINHLVGITYNSSDFANF---------------KK--    113
tr|F9W5M9|F9W5M9_TRYCI            NGERSIGAQAKSLAARFPQNMLTMINHLIGITRNSSEFSNF---------------KQ--    108
tr|A0A1X0P4V9|A0A1X0P4V9_9TRYP    NGDRYIGDQAKALAARFPLNMVTMMNKLIGVTYNSTDFENF---------------KK--    103
tr|Q4CS27|Q4CS27_TRYCC            NGERYIGDQAKALAARFPLNMVTMINQLIGILCDSTEFAML---------------KD--    104
tr|Q4E4F4|Q4E4F4_TRYCC            NGERYIGDQAKALAARFPLNMVTMINQLIGILCDSTEFTML---------------KD--    104
tr|A0A422P1B1|A0A422P1B1_TRYRA    NGERYIGEQAKALAARFPLNMVTMINRLIGILCNSTEFANF---------------QN--    104
tr|A0A422N0X0|A0A422N0X0_9TRYP    NGERYIGDQAKALAARFPLNMVTMINRMIGISYNSTEFSDF---------------RN--    104


tr|A0A0C9MAJ6|A0A0C9MAJ6_9FUNG    RRYINNMI-V-DNERDMPVFIH------NETTQLSIEELIAYQFQNAKHQASVT--AGEK    132
sp|Q9Y4L1|HYOU1_HUMAN             ARFPEHELTF-DPQRQTVHFQI------SSQLQFSPEEVLGMVLNYSRSLAEDF--AEQP    168
sp|Q63617|HYOU1_RAT               SRFPEHELNV-DPQRQTVRFQI------SPQLQFSPEEVLGMVLNYSRSLAEDF--AEQP    168
tr|A0A067CBX2|A0A067CBX2_SAPPC    NEYFPYTLTK-NLTRGSVALQH------SSEHTFHAEELAAMVFGHAKQITNDF--AEGP    156
tr|D8RVG4|D8RVG4_SELML            ASYLPYDFVE-ESPQVTSIRVD-------SQELYRSEELLGMILGYCRGLAEAN--VKAT    157
tr|A0A1P8B8R9|A0A1P8B8R9_ARATH    SVYLPFDIVE-DSRGAVGIKID------DGSTVYSVEELLAMILGYASNLAEFH--AKIP    185
tr|A0A176WKM4|A0A176WKM4_MARPO    ASYLPFDVIK-DDRGAVSIRTH------DKQSVFRSEELLAMVLSYGRDLAEAH--AKGV    169
tr|A0A3R7K087|A0A3R7K087_9TRYP    AASLTYAVVP-HASRDKLAVRIAGGRDKQSVLEFSAEELVGMFLTYLRRIAERGLDG-EP    226
tr|Q4E175|Q4E175_TRYCC            LASLTFSVVP-HSSRDKVAVRILGGKNK-SVLEFSTEELIGMFFAYLKRIAERGLNG-EP    232
tr|Q4E0G9|Q4E0G9_TRYCC            VASLTFSVVP-HSSRDKVTVRILGGKNK-SVLEFSTEELIGMFFAYLKRIAERGLNG-EP    232
tr|A0A6A5CDJ2|A0A6A5CDJ2_NAEFO    ELKVPVSL-VKNEERGTVDVKF------SNDATYSPEELLSMIFLYIKQLADD--GKTAA    174
tr|A0A0S4IUZ2|A0A0S4IUZ2_BODSA    KMRYTYHTASSGTTRGTVNVTFPASEG-VEERQYTADELMGMMLGYAKTQAEREISG--P    174
tr|A0A0L1KXM1|A0A0L1KXM1_9EUGL    DMLFTNALLP-IPERESMAFSVQRGAK-AVNIRYAIETLLGMTFEYVRSITASYVGEGCS    174
tr|A0A0S4JJU2|A0A0S4JJU2_BODSA    -LLTTFALKEGEERKGVNVVCR----Q-DPECNYSAELLIAMYFQYIKLITGK--DAKIK    154
tr|A0A0N0VI62|A0A0N0VI62_LEPPY    -FQYEYHVNF--NNHGSATVNI----C-NVEEPFTAEELFSLILSYCKAAAEH--DDVVT    160
tr|A4HND7|A4HND7_LEIBR            -LHYEYHIGF--NNHGSAIVSI----C-DNKDPFTAEELYSMVLSYCRTAAVN--DEVVD    160
tr|A4IC10|A4IC10_LEIIN            -LQYEYHVSF--NNHGSATVSI----C-DTKDPFTAEELYSMVLSYCKTTAVK--DDVVD    160
tr|E9AFU8|E9AFU8_LEIMA            -LQYEYHVGF--NNHGSATVSI----C-DTKDPFTAEELYSMVLSYCKTTAVK--DDVVD    160
tr|A0A7G2C9U8|A0A7G2C9U8_9TRYP    -LLLEYSIEA--NERGTPVVPF----G-EEHAPFSSEELFAFFLDYFYRIGVN--DGVVD    65
tr|S9TX72|S9TX72_9TRYP            -LLYEYKFGF--SPRGTSQVLI----D-GVKEPYTTEELYAMMFTYFKHIAVN--DDIID    157
tr|S9UBL2|S9UBL2_9TRYP            -LLYEYKFGF--SPRGTSQVLI----D-GVKEPYTTEELYAMMFTYFKHIAVN--DDIID    157
tr|Q38E16|Q38E16_TRYB2            -LQCEFDPHP--EERGTVGFRF----E-DNNDTYTAEEIYAMMLNYCRSISEK--AGVPN    163
tr|F9W5M9|F9W5M9_TRYCI            -LQCEFDPLA--EERDTVGFNF----T-DTGDTYTVEEIYAMMLNYCRSTSRN--AGVPS    158
tr|A0A1X0P4V9|A0A1X0P4V9_9TRYP    -LEYEFETRH--EEDNTVGFSF----G-GRDGNYTVEELYAMMLHYCQNIAQK--DGVND    153
tr|Q4CS27|Q4CS27_TRYCC            -LEFEFETRP--EERNTIGFCF----S--QDGNYTAEELYAMVLQYCQSISEK--DGVVD    153
tr|Q4E4F4|Q4E4F4_TRYCC            -LEFEFETRP--EERNTIGFCF----S--QDGNYTAEELYAMVLQYCQSISEK--DGVVD    153
```

```
tr|A0A422P1B1|A0A422P1B1_TRYRA      -MMLEFETRS--DERSTVGFSF----A--QDGNYTVEELYAMMLQYCQIISEK--DGIVN    153
tr|A0A422N0X0|A0A422N0X0_9TRYP      -LNFEFETRP--EERNTVGFSF----P--QDGSYTVEELYAMMLRYCQSISEK--DGVVD    153
                                                                      : : .  :

tr|A0A0C9MAJ6|A0A0C9MAJ6_9FUNG      VKDCVITVTPFANQFERQAILDAAELAGLNVLTLMHDETAVALNYAVNR--E-IG----K    185
sp|Q9Y4L1|HYOU1_HUMAN               IKDAVITVPVFFNQAERRAVLQAARMAGLKVLQLINDNTATALSYGVFRRKD-IN----T    223
sp|Q63617|HYOU1_RAT                 IKDAVITVPAFFNQAERRAVLQAARMAGLKVLQLINDNTATALSYGVFRRKD-IN----S    223
tr|A0A067CBX2|A0A067CBX2_SAPPC      VKDWVITVPMYFAEPQRQAIIDAAEIAGIRVLSLIDENTAAALHHGVDYEAP-EP----N    211
tr|D8RVG4|D8RVG4_SELML              VKDAVITVPPYFGQSERQAVIAAAQAAGINVLSLMNEHAAAALQYGIDKDF---S----T    210
tr|A0A1P8B8R9|A0A1P8B8R9_ARATH      VKDMVVSVPPYFGQAERRGLIQASQLAGVNVLSLVNEHSGAALQYGIDKDF---A----N    238
tr|A0A176WKM4|A0A176WKM4_MARPO      IKDAIITVPPYLGQAERQGILDAAQIAGITVLGLINEHSGAALQYGIDKDF---G----N    222
tr|A0A3R7K087|A0A3R7K087_9TRYP      LRHLVVAVSTHASLAQRQAVVDAAAVAGLRTVRLVHGTTAAAVQLAYLNADQFFAAAREN    286
tr|Q4E175|Q4E175_TRYCC              LRHLVVTVSAHASLAQRQTFVDAAAVAGLRAVRLVHGTTAAAVQLAHLNIEQFFSSAREK    292
tr|Q4E0G9|Q4E0G9_TRYCC              LRHLVVTVSAHASLAQRQTFVDAAAVAGLRAVRLVHGTTAAAVQLAHLNIEQFFSSAREQ    292
tr|A0A6A5CDJ2|A0A6A5CDJ2_NAEFO      VTDAIVSIPHFFTRAQRQAILDSASIANIKILALMHDHTATALQYGIKSAK--IIKELTK    232
tr|A0A0S4IUZ2|A0A0S4IUZ2_BODSA      VRDAILVVPRGYTSRQRQAMVDAAAITGLRVLSFIHPTTAAALQLGLQNRG--LG----D    228
tr|A0A0L1KXM1|A0A0L1KXM1_9EUGL      KQDAVLTVPHFFDMHQRRALFQSAALGNTNVIATIHTTTAIALQYGVQNRG--FG----S    228
tr|A0A0S4JJU2|A0A0S4JJU2_BODSA      PQEAVFTVPADWTMNQRQALVDAAGLTDLKVLSLMHSTTATALQYGMQKRG--FG----N    208
tr|A0A0N0VI62|A0A0N0VI62_LEPPY      PTGVVVTIPFHTSPVERRSILDAARFSGLKVLGLMHSTTAAAFYYGVRHRG--FG----N    214
tr|A4HND7|A4HND7_LEIBR              PNGIVVTIPFHTSPAARRAILDAARLSGLNVLGLMHSTTAAAFYYGVRHRG--FG----N    214
tr|A4IC10|A4IC10_LEIIN              PKGVVVTIPFHTSPAERRAILDAARLSGLSVLGLMHSTTAAAFYYGVRHRG--FG----N    214
tr|E9AFU8|E9AFU8_LEIMA              PKGVVVTIPFHTSPAERRAILNAARLSGLSVLGLMHSTTAAAFYYGVRHRG--FG----N    214
tr|A0A7G2C9U8|A0A7G2C9U8_9TRYP      PKNVVITVPFHATISERRSILLATHLADVSVLGFMHSTTAAAFYYGIRRRG--FD-----    118
tr|S9TX72|S9TX72_9TRYP              PKSVVLTLPFHSTLSERRSILEAAHFSDTRVLAYMHSTTAAAFYYGVRRRG--FE----G    211
tr|S9UBL2|S9UBL2_9TRYP              PKSVVLTLPFHSTLSERRSILEAAHFSDTRVLAYMHSTTAAAFYYGVRRRG--FE----G    211
tr|Q38E16|Q38E16_TRYB2              PQNVVITLPFHSSLGRRQTILEAARLVHINTLGLLHSTTATALYYGVRRRG--FG----N    217
tr|F9W5M9|F9W5M9_TRYCI              PESMVITLPYNSSFGKRQSVLEAARLVNLNVLGLMHSTTASALYYGIRRRG--FG----N    212
tr|A0A1X0P4V9|A0A1X0P4V9_9TRYP      PKNFVVTVPFHSSLGKRQIILEAARMVGLNVMGLMHSTTAAALYYGVRRRG--FE----N    207
tr|Q4CS27|Q4CS27_TRYCC              PKSVVITIPFHSSMGKRQAILEAARLVGMNVLGLMHSTTAAAFYYGIRRRG--LG----N    207
tr|Q4E4F4|Q4E4F4_TRYCC              PKSVVITIPFHSSMGKRQAILEAARLVGMNVLGLMHSTTAAAFYYGIRRRG--LG----N    207
tr|A0A422P1B1|A0A422P1B1_TRYRA      PKSVVITIPFHSSVGRRQTILEAARLVGANVLGLMHSTTAAALYYGIRRRG--LG----N    207
tr|A0A422N0X0|A0A422N0X0_9TRYP      PKSVVITIPFHSSLGRRQAILEAARLVGADVLGLMHSTTAAALYYGIRRRG--LG----N    207
                                            :. :     *: .. ::         :  :.  :. *.  .

tr|A0A0C9MAJ6|A0A0C9MAJ6_9FUNG      SAENHIFYDMGAGSTVASIVTFSN-VETKDG-----KISRSAPQLEVRG-VGFDRTLGGH    238
sp|Q9Y4L1|HYOU1_HUMAN               TAQNIMFYDMGSGSTVCTIVTYQM-VKTKEA-----G---MQPQLQIRG-VGFDRTLGGL    273
sp|Q63617|HYOU1_RAT                 TAQNIMFYDMGSGSTVCTIVTYQT-VKTKEA-----G---TQPQLQIRG-VGFDRTLGGL    273
tr|A0A067CBX2|A0A067CBX2_SAPPC      TPERIMLYNMGSTSLQVSIVEYKTRVVPDGF-----KKNKTIVEFDVLA-KAWDETLGGA    265
tr|D8RVG4|D8RVG4_SELML              EPRYVLFYDMGANSAFAAVVLFSS-YSAKEY-----GKNVSHNYFELKG-IRWDAEIGGQ    263
tr|A0A1P8B8R9|A0A1P8B8R9_ARATH      GSRHVIFYDMGSSSTYAALVYYSA-YSEKEY-----GKTVSVNQFQVKD-VRWDLGLGGQ    291
tr|A0A176WKM4|A0A176WKM4_MARPO      ETKHVVFYDMGANSLYAAVVYYSA-YSGKER-----GKTTSFNQFQVKG-IRWDATLGGQ    275
tr|A0A3R7K087|A0A3R7K087_9TRYP      ASKYVMVYDMGSRGTEVAVYAFAQSLQ-------------QPGTITLLA-AVVHDTLGGR    332
tr|Q4E175|Q4E175_TRYCC              GPKYIMVYDMGGRRTEVAVYEFAPSRQ-------------RLGTITLRT-AIVNNTLGGR    338
tr|Q4E0G9|Q4E0G9_TRYCC              GPKYIMVYDMGGRRTEVAVYEFAPSRQ-------------RLGTITLRT-AIVNNTLGGR    338
tr|A0A6A5CDJ2|A0A6A5CDJ2_NAEFO      PRHVALFYDIGSVSTTVSIAEYTQSK-----------EKTSLGNIKILG-FASDENLGGS    280
tr|A0A0S4IUZ2|A0A0S4IUZ2_BODSA      EPQHVLIYDMGSTKTEAAVYRFDPIATPAAAAKGGKKSTNSFGTISLVGSIASDMTLGGR    288
tr|A0A0L1KXM1|A0A0L1KXM1_9EUGL      MTKNVLILDMGASKTEVGIYTFTPAAD-GA------KRSESLGTLTTRA-IVTDPFFGGR    280
tr|A0A0S4JJU2|A0A0S4JJU2_BODSA      ETVNVVIYDMGSTKTEVGVYRYSPPEERKDG--KKVKVAVSLGHIETLH-IEVDSTLGGR    265
tr|A0A0N0VI62|A0A0N0VI62_LEPPY      NTVKLLVFDLGATHTEVGVYEISPAPR-RP------PLENAFGTLRTLG-VVEDRSLGGR    266
tr|A4HND7|A4HND7_LEIBR              NSLKMVVFDLGSTHTEVGVYEFLPAAK-RA------PFSSAFGVLRTLG-VVEDRSLGGR    266
tr|A4IC10|A4IC10_LEIIN              NTLRVVVFDLGSTHTEVGVYEFLPAAQ-KA------PLSSAFGTLRTLG-VVEDRSLGGR    266
tr|E9AFU8|E9AFU8_LEIMA              NTLKVVVFDLGSTHTEVGVYEYLPPAP-KA------PLSSAFGTLRTLG-VVEDRSLGGR    266
tr|A0A7G2C9U8|A0A7G2C9U8_9TRYP      KEVNIIVFDVGATHTEAGVYSLSPPGP-NA------TFGDKLGTLKTIK-VLSDKTLGGR    170
tr|S9TX72|S9TX72_9TRYP              RTVNLVIFDVGSTHSEIGVYTFSPPKN-NSA------FSEKLGTLSTRT-VLVDKTLGGR    263
tr|S9UBL2|S9UBL2_9TRYP              RTVNLVIFDVGSTHSEIGVYTFSPPKN-NSA------FSEKLGTLSTRT-VLVDKTLGGR    263
tr|Q38E16|Q38E16_TRYB2              RTVHLLVYDIGSTHTEVGVYKFSPPVQ-EQG--KRTKNVESFGTLTTMG-IVSDATLGGR    273
tr|F9W5M9|F9W5M9_TRYCI              RTVNVLIYDIGSTHTEVGVFKFSPPVQ-QPG--KRVRNVDSFGTLTTLA-IVADPTLGGR    268
tr|A0A1X0P4V9|A0A1X0P4V9_9TRYP      KTINLLIYDIGSTHTEVGIYKFSPPVQ-EGG--KKIKSADSFGTLTTMS-VVDDNFLGGR    263
tr|Q4CS27|Q4CS27_TRYCC              KTMNLLVYDIGSTHTEVGIYKFSPPVA-QSG--KKIKNADSFGTLTTMA-VVDDTFLGGR    263
tr|Q4E4F4|Q4E4F4_TRYCC              KTMNLLVYDIGSTHTEVGIYKFSPPVA-QSG--KKIKNADSFGTLTTMA-VVDDTFLGGR    263
tr|A0A422P1B1|A0A422P1B1_TRYRA      KTINLLVYDIGSTHTEVGIYKFSPPLV-QPG--KKIRNADSFGTLTTMA-VVDDTTLGGR    263
tr|A0A422N0X0|A0A422N0X0_9TRYP      KTINLLVYDIGSTHTEVGIYKFSPPVA-QPG--KKIRNADSFGTLTTMA-VVDDTMLGGR    263
                                            :. ::*.          :                         :        .  :**

tr|A0A0C9MAJ6|A0A0C9MAJ6_9FUNG      ELDVRLQQLLVAGFMKAHGG-----RVSTDIKTSSGAMTRLMKEANRVKQILSANTETMA    293
sp|Q9Y4L1|HYOU1_HUMAN               EMELRLRERLAGLFNEQRKG-----QRAKDVRENPRAMAKLLREANRLKTVLSANADHMA    328
sp|Q63617|HYOU1_RAT                 EMELRLREHLAKLFNEQRKG-----QKAKDVRENPRAMAKLLREANRLKTVLSANADHMA    328
tr|A0A067CBX2|A0A067CBX2_SAPPC      QFDLRLAEKFANEFNAKLTN-------GDDVRKIPRAMAKLRAAARKTKIVLSANEAIPV    318
tr|D8RVG4|D8RVG4_SELML              NLELRLVEHFAKEFEKKT---------GVDVRAFPKAVAKLKKQAKRAKEILSANTEASF    314
tr|A0A1P8B8R9|A0A1P8B8R9_ARATH      SMEMRLVEHFADEFNKQLGN-------GVDVRKFPKAMAKLKKQVKRTKEILSANTAAPI    344
tr|A0A176WKM4|A0A176WKM4_MARPO      TMEARLVDYFAKEFNAQVGM-------FFDVRKHPKAMAKLKKQVKRTKEILSANTEAPL    328
tr|A0A3R7K087|A0A3R7K087_9TRYP      AFDKCIARYIERELFPNARPKAIEPVLAASAPAARKAAASLLRAVKSARERLSVNQEAPV    392
tr|Q4E175|Q4E175_TRYCC              AFDRCIARYIEREHFPKARPKAIEPVLGVSSPAARKAAVSLMRAVQNARERLSVNQEAPV    398
tr|Q4E0G9|Q4E0G9_TRYCC              AFDRCIARYIEREHFPKARPKAIEPVLGVSKPAARKAAVSLMRAVQNARERLSVNQEAPV    398
tr|A0A6A5CDJ2|A0A6A5CDJ2_NAEFO      HFDNVLADFFAEKFIDKHKS---------DPRKEVRPLTRLVEESQKIKHILSANNDAYL    331
tr|A0A0S4IUZ2|A0A0S4IUZ2_BODSA      AVDACLAEFIENAYMTTAPTAGKQRVLTGTTPQSRKAVMSLLRAANKAKEMLSANREAPV    348
tr|A0A0L1KXM1|A0A0L1KXM1_9EUGL      IFDVAIAKMIAEDFHKK---TGIAVLGDTGSLDGRKGIAVLMRSANRAKEVLSSNKECPV    337
tr|A0A0S4JJU2|A0A0S4JJU2_BODSA      TFDACIARFLEEEIVKT---MKIPRIIGGTSLQQHKAMFSLIRAANGIKETLSANQAAPV    322
tr|A0A0N0VI62|A0A0N0VI62_LEPPY      AFDLCVAGIIENEAREK---LNIGSVLGGKTPAQLKSQFSLLRAANKVRETLSVNSVTPY    323
tr|A4HND7|A4HND7_LEIBR              AFDLCVARVIEKEARTK---LGIEPVLGGLSAVQLKSQFSLLRAANKVRETLSVNSNTPY    323
tr|A4IC10|A4IC10_LEIIN              AFDLCVARVIEAEARAK---LSIGPVLGGTTSAQLKSQFSLLRAANKVRETLSVNSNTPY    323
tr|E9AFU8|E9AFU8_LEIMA              AFDLCVARVIEAEARAK---LSIGPVLGGTTSTQLKSQFSLLRAANKVRETFSVNSNTPY    323
tr|A0A7G2C9U8|A0A7G2C9U8_9TRYP      AFDLCIAREMENEAVEK---LNISRVIGKKTAAELKSQYSLLRAANKVREVLSANTQTPY    227
tr|S9TX72|S9TX72_9TRYP              AFDLCIARVMEKEAVEK---MGIKPVIGGKTQAQLKSQYSLLRAANQVREILSVNSVTPF    320
tr|S9UBL2|S9UBL2_9TRYP              AFDLCIARVMEKEAVEK---MGIKPVIGGKTQAQLKSQYSLLRAANQVREILSVNSVTPF    320
tr|Q38E16|Q38E16_TRYB2              ALDSCIAKGIEAEAISK---MKISPVLGGSTVSQRKAQFSLFRAAKRAREVLSVNSKTPV    330
tr|F9W5M9|F9W5M9_TRYCI              TLDLCIAKGIEAEAMSK---IKISKVLGGTTIAQKKAQFSLFRAAKHAREVLSANSATPV    325
```

```
tr|A0A1X0P4V9|A0A1X0P4V9_9TRYP    AFDLCISRLLEKEAMAK---MKIGKVIGGKTVAERKSQFSLLRAAKKAREVLSANSKTPV    320
tr|Q4CS27|Q4CS27_TRYCC            AFDLCIAKIFEAEAMNK---MKISKVIGGKTIPERKSQFSLLRAAKKSREILSANSKTPV    320
tr|Q4E4F4|Q4E4F4_TRYCC            AFDLCIAKIFEAEAMNK---MKISKVIGGKTIPERKSQFSLLRAAKKSREILSANSKTPV    320
tr|A0A422P1B1|A0A422P1B1_TRYRA    AFDLCIAKLLETEAMNK---MKIPKVIGGKSIAERKSQFSLLRAAKKAREILSANSKTPV    320
tr|A0A422N0X0|A0A422N0X0_9TRYP    AFDLCIAKMLEVEAMNK---MKIPKVIGGKSIPERKSQFSLLRAAKKAREILSANSKTPV    320
                                  .: :   :                            *      .  : :* *

tr|A0A0C9MAJ6|A0A0C9MAJ6_9FUNG    SIEGLHE--GIDFKLKVTRAELEDICKDLIARVRNPLQTALEAA---NMTVDDIQSVVLV    348
sp|Q9Y4L1|HYOU1_HUMAN             QIEGLMD--DVDFKAKVTRVEFEELCADLFERVPGPVQQALQSA---EMSLDEIEQVILV    383
sp|Q63617|HYOU1_RAT               QIEGLMD--DVDFKAKVTRVEFEELCADLFDRVPGPVQQALQSA---EMSLDQIEQVILV    383
tr|A0A067CBX2|A0A067CBX2_SAPPC    IVPSLHA--DLDYKGHASRAELEEIAADLFARVLDPVKSALDQA---GLTVGDLSAVEII    373
tr|D8RVG4|D8RVG4_SELML            FVDSLYD--DQDFKSHITRQAFEELCSDLWERAVIPLQKVLEDV---GMTSEQLYAVELL    369
tr|A0A1P8B8R9|A0A1P8B8R9_ARATH    SVESLHD--DRDFRSTITREKFEELCKDLWERSLTPLKDVLKHS---GLKIDDISAVELI    399
tr|A0A176WKM4|A0A176WKM4_MARPO    SVEALHD--DRDFRSTITRKKFEELCSDLWERALIPLKDVLADA---GLKVEDVNAVELI    383
tr|A0A3R7K087|A0A3R7K087_9TRYP    VVQGVQEG-GGDFTATISRARFAQECAHLFDEAARLRDAAIAQTRGVVPSLRDLARFEVI    451
tr|Q4E175|Q4E175_TRYCC            VVQGVQEGDSGDFTTIISRAVFEQECAHLFDEAVRLRDEAIAQTKGIVSSVRELTRFEMI    458
tr|Q4E0G9|Q4E0G9_TRYCC            VVQGVQEGDSGDFTTIISRAVFEQECAHLFDEAVRLRDEAIAQTKGIVSSVRELTRFEVI    458
tr|A0A6A5CDJ2|A0A6A5CDJ2_NAEFO    SIENLYN--DRDLSIKITREEFEKLSEQLLNRLTKPITAALSVA---NLTLDDIDAFEMS    386
tr|A0A0S4IUZ2|A0A0S4IUZ2_BODSA    TVEGIAQ--DSDFSLRISRATLERECGGIWDRAVRVRDAALKSA---NVTLAQLSRFEVV    403
tr|A0A0L1KXM1|A0A0L1KXM1_9EUGL    TVEGITK--GRDHSTIVRRADFEAHCASLFAKVSTLVKSAIDQS---GLDLSEIHAVEVH    392
tr|A0A0S4JJU2|A0A0S4JJU2_BODSA    TVEGVAP--DRDFSGKVTREQFETECSALFERAVEIAKKAIDKS---GLALSDLSSFELM    377
tr|A0A0N0VI62|A0A0N0VI62_LEPPY    TVEGIVP--DRDFHSSMTRATFESGCSDIFDRVKDLAVNVTKSV---NISLSDLTAFEMM    378
tr|A4HND7|A4HND7_LEIBR            TVEGIAP--DRDFHSSMTRATFESECDPLFQRVKGLAANVASVV---NISFEEINSFEMM    378
tr|A4IC10|A4IC10_LEIIN            TVEGIAP--DRDYHSSMTRATFEAECASLFQRVKDLATSVTKVV---NISFKDLNSFEMM    378
tr|E9AFU8|E9AFU8_LEIMA            TVEGIAP--DRDYHSSMTRATFESECAPLFQRVKDLAMSVTDVV---NISFKDLNSFEMM    378
tr|A0A7G2C9U8|A0A7G2C9U8_9TRYP    TVEGISR--DKDFSSTFTREQFERACGGLFDRVKALATEVIKES---GIPVKKLDAFEMM    282
tr|S9TX72|S9TX72_9TRYP            TVEGIAP--DRDFDSHFSREQFEAECGHLFTRVTKLASDALAAS---GLSKSEIDAFEMM    375
tr|S9UBL2|S9UBL2_9TRYP            TVEGIAP--DRDFDSHFSREQFEAECGHLFTRVTKLASDALAAS---GLSKSEIDAFEMM    375
tr|Q38E16|Q38E16_TRYB2            TVEGIAP--DRDFSTEVSRKEFETNCSELLKRFPRVAQEAVTKS---SLTLKDIDAFEMM    385
tr|F9W5M9|F9W5M9_TRYCI            TVEGIAP--ERDFNTVISRKDFEANCSELFKRFPVVAREALNKS---SLSLGDIDAFEMM    380
tr|A0A1X0P4V9|A0A1X0P4V9_9TRYP    TVEGIVP--DRDFHTEITRKDFEEKCSELFERVPKVAEEALKKS---GLDLKDIDAFEMM    375
tr|Q4CS27|Q4CS27_TRYCC            TIEGIVP--ERDFSTEISRKDLEEKCSHLFERVPKLAEEALSKA---GLSLSDIDAFEMM    375
tr|Q4E4F4|Q4E4F4_TRYCC            TIEGIVP--ERDFSTEISRKDLEEKCSHLFERVPKLAEEALSKA---GLSLSDIDAFEMM    375
tr|A0A422P1B1|A0A422P1B1_TRYRA    TVEGIVP--DRDFNTQISRKDLEEKCSYLFERVPKVAEEAVSKS---GLSLKDIDAFEMM    375
tr|A0A422N0X0|A0A422N0X0_9TRYP    TVEGIVP--DRNFNTQISRKDLEVKCAYLFERVPKVVEEALSKS---GLSLKEIDAFEMM    375
                                  :  :   :      *  :  .  : .   .             .: . :

tr|A0A0C9MAJ6|A0A0C9MAJ6_9FUNG    GGGVRVPSVQKQLVDLVGAQ---KIAKNVNADEAAVLGAAFRGASLSNQFRLSKQISIKD    405
sp|Q9Y4L1|HYOU1_HUMAN             GGATRVPRVQEVLLKAVGKE---ELGKNINADEAAAMGAVYQAAALSKAFKVKP-FVVRD    439
sp|Q63617|HYOU1_RAT               GGPTRVPKVQEVLLKPVGKE---ELGKNINADEAAAMGAVYQAAALSKAFKVKP-FVVRD    439
tr|A0A067CBX2|A0A067CBX2_SAPPC    GGGVRIPKIQALLQAFVQ-R---DLGKRLNGDEAMALGAAFNAANLSTSFRV-RHVGMTD    428
tr|D8RVG4|D8RVG4_SELML            GGGTRVPKLQQVLAQALGKK---PLERHLDADEAITLGAALYAANISDGIKLNRKIGMFD    426
tr|A0A1P8B8R9|A0A1P8B8R9_ARATH    GGATRVPKLQSTIQEFIGKQ---QLDKHLDADEAIVLGSALHAANLSDGIKLKRRLGIVD    456
tr|A0A176WKM4|A0A176WKM4_MARPO    GGATRVPKLQAVLSDFLGKK---NLDRHLDADEAVVLGASLQAANLSDGFKLNRKLGMID    440
tr|A0A3R7K087|A0A3R7K087_9TRYP    GGATRMPKLLERLSDGYGR----AVDRTLNSDEATVVGAAYVGAARAG-IPVR-GFRVVE    505
tr|Q4E175|Q4E175_TRYCC            GGATRMPKLLERLSDGYGR----AVDRTLNSDEAAVVGASYMGAARAN-IPIR-GFAVVE    512
tr|Q4E0G9|Q4E0G9_TRYCC            GGATRMPKLLERLSDGYGR----AVDRTLNSDEAAVVGASYMGAARAN-IPIR-GFAVVE    512
tr|A0A6A5CDJ2|A0A6A5CDJ2_NAEFO    GGSSRIPAVQDRISKFVGSK--LMVGYSLNADEAIAMGSSFYGAMLSPSFKVT-AFKIFD    443
tr|A0A0S4IUZ2|A0A0S4IUZ2_BODSA    GGGLRVPIIIEKLSEGYNKAGSGVVDRTLNSDEAMVVGAGYYGAALSGHFRIK-GFRLVE    462
tr|A0A0L1KXM1|A0A0L1KXM1_9EUGL    GGAIRIPKLLDVLAEELGQ----PIQRTLNGDEAAVLGAAFHAARVSGTFGVK-GFAIQE    447
tr|A0A0S4JJU2|A0A0S4JJU2_BODSA    GGGVRPPKIVSDLSAFLGR----PVDRTLNGDEAAAFGAGYYGARLSGYFRVR-SFSIRD    432
tr|A0A0N0VI62|A0A0N0VI62_LEPPY    GGISRTPKIIADLSAFLGR----DVDRTMNMDEAAAMGAGYYAAKLSPLYHAK-SLKLDE    433
tr|A4HND7|A4HND7_LEIBR            GGLSRTPRIIADLSKVIGR----GVDRTMNMDEAAAIGAGYYAAKLSPLYHAK-SLKLDE    433
tr|A4IC10|A4IC10_LEIIN            GGLSRTPKIIADLSEAIGR----DVDRTMNMDEAAAIGAGYYAAKLSPLYRAK-SLKLDE    433
tr|E9AFU8|E9AFU8_LEIMA            GGLSRTPKIIADLSEVIGR----DVDRTMNMDEAAAIGAGYYAAKLSPLYRAK-SLKLDE    433
tr|A0A7G2C9U8|A0A7G2C9U8_9TRYP    GGVSRTPKIISDISAILKR----DVDRTMNMDEAAAIGSAYFAVKKSVHYKSK-SFRVDE    337
tr|S9TX72|S9TX72_9TRYP            GGASRTPKIITDVSVFLGK----DVDRTMNMDEAAAIGAAYYAVKISPHYRSK-SFKVQE    430
tr|S9UBL2|S9UBL2_9TRYP            GGASRTPKIITDVSVFLGK----DVDRTMNMDEAAAIGAAYYAVKISPHYRSK-SFKVQE    430
tr|Q38E16|Q38E16_TRYB2            GGASRTPKIISDLSTFWGK----EVNRTLNSDEAAAMGAAYYALRLSPHYRPH-SFRIIE    440
tr|F9W5M9|F9W5M9_TRYCI            GGTSRTPKIINDLSAFWGR----EVNRTLNSDEAAAMGAAYYALRLSPHYRPH-SFRIIE    435
tr|A0A1X0P4V9|A0A1X0P4V9_9TRYP    GGTSRTPKILADLSTFLGR----EVDRTLNSDESAAIGAAYYALRLSPFYRVR-SFRVVE    430
tr|Q4CS27|Q4CS27_TRYCC            GGTSRTPKIISDLSAMLGR----EVDRTLNSDEAAAVGAAYYALRLSPFYRVR-SFRVEE    430
tr|Q4E4F4|Q4E4F4_TRYCC            GGTSRTPKIISDLSAMLGR----EVDRTLNSDEAAAVGAAYYALRLSPFYRVR-SFRVEE    430
tr|A0A422P1B1|A0A422P1B1_TRYRA    GGTSRTPKIIAELSALLGR----EVDRTLNSDEAAAMGAAYHALRLSSFYRVR-SFRLEE    430
tr|A0A422N0X0|A0A422N0X0_9TRYP    GGTSRTPKIISDLSAMLGR----EVDRTLNSDEAAALGAAYYALRLSSFYRVR-SFRVEE    430
                                  **  * * :  :          :   :: **: ..*:   .   :         . : :

tr|A0A0C9MAJ6|A0A0C9MAJ6_9FUNG    VTLFPIEVTYKPENKG-K---EAKTAVD---------TTLFNKFDAIGTRKIMTFNRV-N    451
sp|Q9Y4L1|HYOU1_HUMAN             AVVYPILVEFTREVEE-EPGIHSLKHNK---------RVLFSRMGPYPQRKVITFNRYSH    489
sp|Q63617|HYOU1_RAT               AVIYPILVEFTREVEE-EPGLRSLKHNK---------RVLFSRMGPYPQRKVITFNRYSH    489
tr|A0A067CBX2|A0A067CBX2_SAPPC    IASFPVGVRLVDLASH------AADDDHPEAKHWVKRAALFGERQKLNVKKSVSFSH-AD    481
tr|D8RVG4|D8RVG4_SELML            GASYGVVLKVNDDGP------------YEL---------VVPRLRRIPVKLARAVKQQ-E    464
tr|A0A1P8B8R9|A0A1P8B8R9_ARATH    GSPYGFLVELEGPNVK------KDESTKQQ---------LVPRMKKLPSKMFRSFVLD-K    500
tr|A0A176WKM4|A0A176WKM4_MARPO    GASYSILMELDGASPG------ALENVDNV---------LVHRLKKIPSKAYRALKNQKS    485
tr|A0A3R7K087|A0A3R7K087_9TRYP    PLMNDVYFSLTPP--LQGGGG-----------AGTRHLVFAKARTLVPAVQSLRFKNRTA    552
tr|Q4E175|Q4E175_TRYCC            PLMNDVWFSLTPS--LQDPRDAAAPP-----RDSTRHLIFPKGSTILPAVRSLQLKNRTA    565
tr|Q4E0G9|Q4E0G9_TRYCC            PLMNDVWFSLTPL--LQDPRDAAAPP-----RNSTRHLIFPKGSTILPAVRSLQLKNRTA    565
tr|A0A6A5CDJ2|A0A6A5CDJ2_NAEFO    TVPHKVDFLLSGND--KE-------------------INLFNHKDEIDAKKTITVPRT-E    481
tr|A0A0S4IUZ2|A0A0S4IUZ2_BODSA    RLGFPVWITVALNSSSNTEATGTAEA-----PSSQQRKLLFPSEARLPTRLRVRVNATVS    517
tr|A0A0L1KXM1|A0A0L1KXM1_9EUGL    TIPYNITFSISPK--ASDKGLGTTED-----APVKSRGLFAN--ASWPAKKSVSVNRT-E    497
tr|A0A0S4JJU2|A0A0S4JJU2_BODSA    FFPQSIHFKIVSA--DGT--------------SSAIRPLFVK--SPFGARKSITVNRT-E    473
tr|A0A0N0VI62|A0A0N0VI62_LEPPY    TIPYSVEFEVHPR--LNKK------------KPLTRRPLFGTDGVLLGDAVSLTFNRT-D    478
tr|A4HND7|A4HND7_LEIBR            TIPYRIDFEVDPP--LSKE------------KPAARRPLFSVADFLLGDSVSLTFNRT-E    478
tr|A4IC10|A4IC10_LEIIN            TIPYGVDFEVEPP--LSKE------------KPAVRRPLFGADGFLLGDSVSLTFNRT-E    478
tr|E9AFU8|E9AFU8_LEIMA            TIPYRVDFEVEPP--LSKE------------KPAVRRPVFSADGFLLGDSVSITFNRT-E    478
tr|A0A7G2C9U8|A0A7G2C9U8_9TRYP    RVPYPVYFSLST-----NT------------SNSSRRLLLSS-DELIGQSFSITFEAD-T    378
tr|S9TX72|S9TX72_9TRYP            RVPFPLYFSVYPA--FHSN------------KPSARRMLLHS-DAFIGDAVSITFNRT-D    474
```

```
tr|S9UBL2|S9UBL2_9TRYP             RVPFPLYFSVYPA--FHSN------------KPSARRMLLHS-DAFIGDAVSITFNRT-D    474
tr|Q38E16|Q38E16_TRYB2             RVPYTFLFAVSPE--VKNS------------SNSKR--MLAN-NPIIGSRWSITVNRT-D    482
tr|F9W5M9|F9W5M9_TRYCI             RIPSTLSFVVNPR--CYEL-----------------------------------------    452
tr|A0A1X0P4V9|A0A1X0P4V9_9TRYP     HIPFKFFFTITPS--LNNS------------SVSKR--VLVE-QPIIGSRKSITLNRT-D    472
tr|Q4CS27|Q4CS27_TRYCC             HIPFVFFFSITSS--SKNS------------SQSRR--LLAE-NPALGMRQSITLNRT-E    472
tr|Q4E4F4|Q4E4F4_TRYCC             HIPFVFFFSITSS--SKNS------------SQSRR--LLAE-NPALGMRQSITLNRT-E    472
tr|A0A422P1B1|A0A422P1B1_TRYRA     HIPFVFYFRITPF--VKAS------------APSLR--LLAE-NPVLGMRRSVTLNRT-E    472
tr|A0A422N0X0|A0A422N0X0_9TRYP     HIPFVFSFCVTPF--LKTS------------SPSPR--LLVE-NPALGMRRSITLNRT-E    472
                                            . .

tr|A0A0C9MAJ6|A0A0C9MAJ6_9FUNG     DFEFDLSYGKNAAADVDG--------IAKVKVTGLTEAMKKHKDDIKAS-----------    492
sp|Q9Y4L1|HYOU1_HUMAN              DFNFHINYGDLGFLGPEDLRVFGSQNLTTVKLKGVGDSFKKYPDYE-S------------    536
sp|Q63617|HYOU1_RAT                DFNFHINYGDLGFLGPEDLRVFGSQNLTTVKLKGVGESFKKYPDYE-S------------    536
tr|A0A067CBX2|A0A067CBX2_SAPPC     DVSCTFRYDKPSALPLGVS-----SIISRYNITGIEAFAKKMEDKKLG------------    524
tr|D8RVG4|D8RVG4_SELML             DYELSLHYDPEGELPLGII----DREIAAFKISGVTDSVAKYSSYNLS------------    508
tr|A0A1P8B8R9|A0A1P8B8R9_ARATH     DFDVSLAYESEGILPPGTT----SPVFAQYSVSGLADASEKYSSRNLS------------    544
tr|A0A176WKM4|A0A176WKM4_MARPO     DFKVALKYD-ATLLPPGVI----SPEVATYQITGVADAANKYASYNVS------------    528
tr|A0A3R7K087|A0A3R7K087_9TRYP     DFTLTLEDDGGRF-------------ARATSIHGVGESISAA--------------RGRR    585
tr|Q4E175|Q4E175_TRYCC             DFTLTLEDESGHF-------------TRATFIRGVNESIMAAQLLPQSFRASDGAGKKRR    612
tr|Q4E0G9|Q4E0G9_TRYCC             DFTLTLEDESGHF-------------TRATFIRGVNESIIAAQLLPQSLRASDGAGKKRK    612
tr|A0A6A5CDJ2|A0A6A5CDJ2_NAEFO     DFTITLKYASEYL-PSGYST--EHAAFATYEITGVTKAMSNWTFEDE---------R---    526
tr|A0A0S4IUZ2|A0A0S4IUZ2_BODSA     DFIVTLDETTNSS----SSA--TIVSKSNTLVSGVLGALQQVGYGDVDAAD-----R---    563
tr|A0A0L1KXM1|A0A0L1KXM1_9EUGL     NFEMTFSDQNG--------------PLQVLKVSGLHDSLEKLGHFSPER-D-----P---    534
tr|A0A0S4JJU2|A0A0S4JJU2_BODSA     DFTIALLSTVDGE----SF-----TPLMSVNVSGVKSALEGLNYFNPTIVH-----E---    516
tr|A0A0N0VI62|A0A0N0VI62_LEPPY     DFSVDLFAGTD------AK-----LPIATIEVTDVKKALTNLGALSPTVQH-----P---    519
tr|A4HND7|A4HND7_LEIBR             DFSINFYTEGD------AT-----RPFVSVTVTGVKQALTRMNSLPSVVKH-----A---    519
tr|A4IC10|A4IC10_LEIIN             DFSLNFYIDDD------AA-----RPFVRVTVTGVKQALTDMKALSPAVEH-----A---    519
tr|E9AFU8|E9AFU8_LEIMA             DFSLNFYSD-D------AA-----TPFVCITVTGVKQALTDMNALSPVVKH-----A---    518
tr|A0A7G2C9U8|A0A7G2C9U8_9TRYP     NFELDFYDSDS------AP-----QPFLKETFSSVES---LLKLYEPSVQH-----E---    416
tr|S9TX72|S9TX72_9TRYP             DFTVRLYQSET------TE-----EPFAEVRVEGVKKKLDVMNFFEPLIAH-----E---    515
tr|S9UBL2|S9UBL2_9TRYP             DFTVRLYQSET------TE-----EPFAEVRVEGVKKKLDVMNFFEPLIAH-----E---    515
tr|Q38E16|Q38E16_TRYB2             DFSIQLFDSDA------R--------IGNVNITGVKGALERLGFFQPKLNH-----T---    520
tr|F9W5M9|F9W5M9_TRYCI             ------------------------------------------------------------    452
tr|A0A1X0P4V9|A0A1X0P4V9_9TRYP     DFSIQLFDSQD------F--------VGEISVTGVKEALKLLQEKKSDFDH-----P---    510
tr|Q4CS27|Q4CS27_TRYCC             DFTVEIFESNN------C--------VVKIQVMGVKTTLERLGFFAPTIVH-----P---    510
tr|Q4E4F4|Q4E4F4_TRYCC             DFTVEIFESNN------C--------VVKIQVIGVKTTLERLGFFAPTIVH-----P---    510
tr|A0A422P1B1|A0A422P1B1_TRYRA     DFVIELFESNN------F--------VGKITVSGVKTALDQLGYFEPAIVH-----P---    510
tr|A0A422N0X0|A0A422N0X0_9TRYP     DFAIELFESNT------I--------VGKITVIGVKAALEQLGYFTPTILH-----P---    510


tr|A0A0C9MAJ6|A0A0C9MAJ6_9FUNG     ----EIPPKVRVSIELSNSGLVSVPEATLQI------------GKLTFK---EKVKSFFG    533
sp|Q9Y4L1|HYOU1_HUMAN              -------KGIKAHFNLDESGVLSLDRVESVFETLVEDSAEEESTLTKLG---NTISSLFG    586
sp|Q63617|HYOU1_RAT                -------KGIKAHFNLDESGVLSLDRVESVFETLVEDSPEEESTLTKLG---NTISSLFG    586
tr|A0A067CBX2|A0A067CBX2_SAPPC     -----EP-KVTLSFNLDASGLASIVKAEATLEEEIEVPAPTKKAAKKADD--SSSSSAD-    575
tr|D8RVG4|D8RVG4_SELML             -----APIKSVLHFALSRSGVLSLDRAETVVEFTELVEVPVNVSTNATTTLESTSNASV-    562
tr|A0A1P8B8R9|A0A1P8B8R9_ARATH     -----APIKANLHFSLSRSGILSLDRGDAVIEITEWVDVPKKNVTIDSNTTTSTGNATD-    598
tr|A0A176WKM4|A0A176WKM4_MARPO     -----APLKTSLHFSLSRSGVISLDKAETVVEISEWYEVPVANATTDVNATVEAKSTEEG    583
tr|A0A3R7K087|A0A3R7K087_9TRYP     GGMSLEHTEVVVEVTVAESGIPFVSNAYLRATYVKRTAAARKGQTGSANTTRNEFFAA--    643
tr|Q4E175|Q4E175_TRYCC             ERLSLEHTEVMLEVTATESGIPFVSNAYLHATYLKVTVAKEPTD-----TVQNESNAG--    665
tr|Q4E0G9|Q4E0G9_TRYCC             ERLSLEHTEVMLEVTATESGIPFVSNAYLHATYLKVTVAKEPTD-----TVQNESNAG--    665
tr|A0A6A5CDJ2|A0A6A5CDJ2_NAEFO     ----GKKKKVKATFKVNKFGLVELDSVNAILEETITVR--VESNNT-----------T--    567
tr|A0A0S4IUZ2|A0A0S4IUZ2_BODSA     ----NITHTVWVDIRASEAAMVYIEKAEIEIHYFVNASRKVRKLVT-----------P--    606
tr|A0A0L1KXM1|A0A0L1KXM1_9EUGL     ----QNSCSVRIEAKLDGNGVLSASSVKL--KYNEWKVKSSESDES-----------S--    575
tr|A0A0S4JJU2|A0A0S4JJU2_BODSA     ----NNTHIVRVELKLSENGVVVVEDSEVRVRLAANVTKKLKTRKN-----------S--    559
tr|A0A0N0VI62|A0A0N0VI62_LEPPY     ----NNSHMIRMQVIMNETGLVQLEHTEALVRYAAQVTEKTKENVT-----------D--    562
tr|A4HND7|A4HND7_LEIBR             ----NNSHMIRLQIVLNETGLVQVEYAEAVVRYAEEVTQNTRENVT-----------G--    562
tr|A4IC10|A4IC10_LEIIN             ----NNSHMVRLQIVLNETGLVQVEHAEVVVRYAEEVTQKVTENVT-----------D--    562
tr|E9AFU8|E9AFU8_LEIMA             ----NNSHMVRLQIVLNETGLVEVEHAEVVVRYAEEVTQKVTENVT-----------D--    561
tr|A0A7G2C9U8|A0A7G2C9U8_9TRYP     ----NNTHMVRIQMRVNSTGLPQIEEADIIFRRVENATKHGKSGNE-----------T--    459
tr|S9TX72|S9TX72_9TRYP             ----NNSHLVRLQLLINETGLPMVDASDVVYRYVTNVTERHSVTDA-----------A--    558
tr|S9UBL2|S9UBL2_9TRYP             ----NNSHLVRLQLLINETGLPMVDASDVVYRYVTNVTERHSVTDA-----------A--    558
tr|Q38E16|Q38E16_TRYB2             ----NNSHIIRIQVRFNESGLLEVEEAGVFYRYAVNVSSSTKTTGV-----------Q--    563
tr|F9W5M9|F9W5M9_TRYCI             ------------------------------------------------------------    452
tr|A0A1X0P4V9|A0A1X0P4V9_9TRYP     ----NNTHIVRIQLLLNESGLFEVEEADVIIRYAVNVSVKVKVNTT-----------D--    553
tr|Q4CS27|Q4CS27_TRYCC             ----NNSHIVRVQLRINDSGLFEVDEADVIYRYAANVSSKIKLNTT-----------T--    553
tr|Q4E4F4|Q4E4F4_TRYCC             ----NNSHIVRVQLRINDSGLFEVDEADVIYRYAANVSSKIKLNTT-----------T--    553
tr|A0A422P1B1|A0A422P1B1_TRYRA     ----NNTHIVRVQLRLNDSGLFEVEEAEVIYRYAANVSRKIRVNAT-----------S--    553
tr|A0A422N0X0|A0A422N0X0_9TRYP     ----NNTHVVRVQLRLNDSGLFEVEEADAIYRYAANVSRKIKINAT-----------S--    553


tr|A0A0C9MAJ6|A0A0C9MAJ6_9FUNG     GKDSKDAEADKKNEDANQKNETAKT-----------------------------------    558
sp|Q9Y4L1|HYOU1_HUMAN              GGTTPDAKENGTDT--VQEEEESPAEGSKDEP--------------GE------------    618
sp|Q63617|HYOU1_RAT                GGTSSDAKENGTDA--VQEEEESPAEGSKDEP--------------AE------------    618
tr|A0A067CBX2|A0A067CBX2_SAPPC     -----DDKD---------------------------------------------------    579
tr|D8RVG4|D8RVG4_SELML             ------------------------------------------------------------    562
tr|A0A1P8B8R9|A0A1P8B8R9_ARATH     ----ENSQENKEDL---Q------T-----------------------------------    610
tr|A0A176WKM4|A0A176WKM4_MARPO     AGSPEEAKDEEVKL---SSEEVKQT-----------------------------------    605
tr|A0A3R7K087|A0A3R7K087_9TRYP     ----SAAEENATDTEASEEAQKHPTPQRPGEQAEEAGAGPTLPPPPPQPPQKQQQPAGGA    699
tr|Q4E175|Q4E175_TRYCC             ----SAAGENATDAEAPNGTEKSSKPQQPDEYP-------------GEEQEEQQQRDLGA    708
tr|Q4E0G9|Q4E0G9_TRYCC             ----SAAGENATDAEAPNGTEKSSKLQQPDEYP-------------GEEQEGQQQRDLGG    708
tr|A0A6A5CDJ2|A0A6A5CDJ2_NAEFO     ----T----------------------------------------SNE------------    571
tr|A0A0S4IUZ2|A0A0S4IUZ2_BODSA     ----PAEEKNASDGDASASPATEKTT-----------------PESGEEQPSTAEDAGGD    645
tr|A0A0L1KXM1|A0A0L1KXM1_9EUGL     ------------------------------------------------------------    575
tr|A0A0S4JJU2|A0A0S4JJU2_BODSA     ----SEEIPDQEK-----------TN-----------------DDSNE------------    575
tr|A0A0N0VI62|A0A0N0VI62_LEPPY     ----ST---------------------------------------TGE------------    567
tr|A4HND7|A4HND7_LEIBR             ----EQ---------------------------------------SSE------------    567
tr|A4IC10|A4IC10_LEIIN             ----KA---------------------------------------SGE------------    567
```

```
tr|E9AFU8|E9AFU8_LEIMA                  ----KA---------------------------------------SGE------------    566
tr|A0A7G2C9U8|A0A7G2C9U8_9TRYP          ------------------------------------------------------------    459
tr|S9TX72|S9TX72_9TRYP                  ----DEEE------------------------------------AHNE------------    566
tr|S9UBL2|S9UBL2_9TRYP                  ----DEED------------------------------------AHNE------------    566
tr|Q38E16|Q38E16_TRYB2                  ----ADS-----------------------------------------------------    566
tr|F9W5M9|F9W5M9_TRYCI                  ------------------------------------------------------------    452
tr|A0A1X0P4V9|A0A1X0P4V9_9TRYP          ----ELPNT--T------------------------------------------------    559
tr|Q4CS27|Q4CS27_TRYCC                  ----KDLNT--SD-----------IT-----------------NTSNI------------    567
tr|Q4E4F4|Q4E4F4_TRYCC                  ----NDLNT--SD-----------IT-----------------NTSNI------------    567
tr|A0A422P1B1|A0A422P1B1_TRYRA          ----NDLND--TD-----------N-----------------------------------    561
tr|A0A422N0X0|A0A422N0X0_9TRYP          ----N---D--TD-----------N-----------------------------------    558

tr|A0A0C9MAJ6|A0A0C9MAJ6_9FUNG          ---------------------------------DG--------------TADSKNKTAAE    571
sp|Q9Y4L1|HYOU1_HUMAN                   ----QVELKEEA----------------EAPVEDGSQPPPPEPKG----DATPEGEKATE    654
sp|Q63617|HYOU1_RAT                     ----QGELKEEA----------------EAPMEDTSQPPPSEPKG----DAAREGETPDE    654
tr|A0A067CBX2|A0A067CBX2_SAPPC          --------------------------------DDKTT--------------------PEN    587
tr|D8RVG4|D8RVG4_SELML                  ------------------------------------------------------AE-NVT    567
tr|A0A1P8B8R9|A0A1P8B8R9_ARATH          -------------------------------DAENS---------------TASNTTAEE    624
tr|A0A176WKM4|A0A176WKM4_MARPO          --------------------------------QADSSPEAGD-VS----GVSSEGEPSKD    628
tr|A0A3R7K087|A0A3R7K087_9TRYP          G-----AKWEEAPEAAVEAEAEEEVEEEEEAEVAQSNPEE-GQAGERGDNGTGEGAVPGV    753
tr|Q4E175|Q4E175_TRYCC                  E-----EKWDERQEKAAEEEGEEERKENHNVS---------DQASEKD-SNASEATAPRA    753
tr|Q4E0G9|Q4E0G9_TRYCC                  E-----EKWDERQEKAVEEEGEEERKENYNVS---------DQSSEKE-SNTSEATAPRA    753
tr|A0A6A5CDJ2|A0A6A5CDJ2_NAEFO          -----------T----------------------------------------------KT    574
tr|A0A0S4IUZ2|A0A0S4IUZ2_BODSA          SASDEGEISDEAPDATTTS--------------SSSKPSASDDITEGAKKASKDSTAPKK    691
tr|A0A0L1KXM1|A0A0L1KXM1_9EUGL          ------------------------------------------------------------    575
tr|A0A0S4JJU2|A0A0S4JJU2_BODSA          -----------T-------------------------------------------AQATE    581
tr|A0A0N0VI62|A0A0N0VI62_LEPPY          -----------V------------------------------------------------    568
tr|A4HND7|A4HND7_LEIBR                  -----------T------------------------------------------------    568
tr|A4IC10|A4IC10_LEIIN                  -----------R------------------------------------------------    568
tr|E9AFU8|E9AFU8_LEIMA                  -----------R------------------------------------------------    567
tr|A0A7G2C9U8|A0A7G2C9U8_9TRYP          ------------------------------------------------------------    459
tr|S9TX72|S9TX72_9TRYP                  -----------S------------------------------------------------    567
tr|S9UBL2|S9UBL2_9TRYP                  -----------S------------------------------------------------    567
tr|Q38E16|Q38E16_TRYB2                  ------------------------------------------------------------    566
tr|F9W5M9|F9W5M9_TRYCI                  ------------------------------------------------------------    452
tr|A0A1X0P4V9|A0A1X0P4V9_9TRYP          ------------------------------------------------------------    559
tr|Q4CS27|Q4CS27_TRYCC                  -----------T------------------------------------------------    568
tr|Q4E4F4|Q4E4F4_TRYCC                  -----------T------------------------------------------------    568
tr|A0A422P1B1|A0A422P1B1_TRYRA          -----------T------------------------------------------------    562
tr|A0A422N0X0|A0A422N0X0_9TRYP          -----------T------------------------------------------------    559

tr|A0A0C9MAJ6|A0A0C9MAJ6_9FUNG          EAK------ESVSIAKIPLTIEFIPTGLAP------------------------------    595
sp|Q9Y4L1|HYOU1_HUMAN                   KEN-----GDKSEAQKPSEKAEAGPEGVAPAPEGEKKQKPARKRRMVEEIGVELVVLDLP    709
sp|Q63617|HYOU1_RAT                     KES-----GDKSEAQKPNEKGQAGPEGVPPAPEEEKKQKPARKQKMVEEIGVELAVLDLP    709
tr|A0A067CBX2|A0A067CBX2_SAPPC          DEV-----PEMIKKTKVHRVTLTVVRSDAA-------------------HRP-EAGMAIL    622
tr|D8RVG4|D8RVG4_SELML                  QAP-----VETIMEKKLKKKTIRIPSKLEV-------------------LRD---T---G    597
tr|A0A1P8B8R9|A0A1P8B8R9_ARATH          PAV-----ASLGTEKKLKKRTFRIPLKVVE-------------------KTV-GPG---A    656
tr|A0A176WKM4|A0A176WKM4_MARPO          SPV-----AEPVVKKKLRRRTIRIPLKIKD-------------------LSK-GPN---M    660
tr|A0A3R7K087|A0A3R7K087_9TRYP          GSVSTAKREEEVMVVKRVVRGVPLRFA--P-----------------------APFSAGV    788
tr|Q4E175|Q4E175_TRYCC                  -----EDTSAEKEIVERVVRSFPLRLV--S-----------------------VPLSVGV    783
tr|Q4E0G9|Q4E0G9_TRYCC                  -----EDKSAEKEIVERVVRSFPLSLV--S-----------------------VPLSVGV    783
tr|A0A6A5CDJ2|A0A6A5CDJ2_NAEFO          EPV---Y--------KKDTRVTKVALDVKF-----------------------IDISVIK    600
tr|A0A0S4IUZ2|A0A0S4IUZ2_BODSA          KPT---YVTVTEVHVEARSRVFPLTLTTTF-----------------------AS--PAV    723
tr|A0A0L1KXM1|A0A0L1KXM1_9EUGL          -ST---EVS--EEISEPIKHDISLPMEWEY-----------------------IG--RRP    604
tr|A0A0S4JJU2|A0A0S4JJU2_BODSA          VSA---ELP--SVQIQMKKRSTSIKPTIEL-----------------------SQ--PSM    611
tr|A0A0N0VI62|A0A0N0VI62_LEPPY          KEV---DKT--HTVIRMRHKTAVVPAALIW-----------------------KN--PDA    598
tr|A4HND7|A4HND7_LEIBR                  KPV---EKS--VKVVKMRHQVVDLTATLAW-----------------------KN--PTE    598
tr|A4IC10|A4IC10_LEIIN                  KSV---EKS--VKVIKMRIRAADVTATLAW-----------------------KN--PTG    598
tr|E9AFU8|E9AFU8_LEIMA                  KSV---EKS--VKVIKMRSRAADVSATLAW-----------------------KN--PTG    597
tr|A0A7G2C9U8|A0A7G2C9U8_9TRYP          --------A--EPVLETRRHSFVLTSKPTW-----------------------YD--PAE    484
tr|S9TX72|S9TX72_9TRYP                  APH---VKY--VYKLKTRKRELHVPATLHW-----------------------FH--PRE    597
tr|S9UBL2|S9UBL2_9TRYP                  APH---VKY--VYKLKTRKRELHVPATLHW-----------------------FH--PRE    597
tr|Q38E16|Q38E16_TRYB2                  NSS---NES--GYVVEMKRKSARLSARVTF-----------------------TN--PEP    596
tr|F9W5M9|F9W5M9_TRYCI                  ------------------------------------------------------------    452
tr|A0A1X0P4V9|A0A1X0P4V9_9TRYP          ------EEV--KQVVKMKRETMSLSTKVRF-----------------------VN--PSL    586
tr|Q4CS27|Q4CS27_TRYCC                  NTS---YRT--VHNIKMKRTSATVATTVSF-----------------------TN--PSP    598
tr|Q4E4F4|Q4E4F4_TRYCC                  NTS---YRT--VHNIKMKRTSATVATTVSF-----------------------TN--PSP    598
tr|A0A422P1B1|A0A422P1B1_TRYRA          NTS---FQR--VYNIHMKRKSLLISTTVSF-----------------------TN--PSP    592
tr|A0A422N0X0|A0A422N0X0_9TRYP          NTS---YQK--VYSIIMKRKSLPISTAVSF-----------------------TN--PSP    589

tr|A0A0C9MAJ6|A0A0C9MAJ6_9FUNG          -LSEKDKAIAKKRIAELDALDSRKKLREESRNALETFVYKVQ--DFLYNDI--VALVATE    650
sp|Q9Y4L1|HYOU1_HUMAN                   DLPEDKLAQSVQKLQDLTLRDLEKQEREKAANSLEAFIFETQ--DKLYQPE--YQEVSTE    765
sp|Q63617|HYOU1_RAT                     DLPEDELAHSVQKLEDLTLRDLEKQEREKAANSLEAFIFETQ--DKLYQPE--YQEVSTE    765
tr|A0A067CBX2|A0A067CBX2_SAPPC          PMTFRDKKESIAMLAEMDKVDNIRKANAEAKNRLEAFVYESR--EYLSSHDDVLEKVTTT    680
tr|D8RVG4|D8RVG4_SELML                  AYSEDQVAQFRSRLDRLKELDDEKRQTEEAKNNLESYIYTTK--EMLDTIKD-LSKVSTE    654
tr|A0A1P8B8R9|A0A1P8B8R9_ARATH          PFSKESLAEAKIKLEALDKKDRERRRTAELKNNLESYIYATK--EKLETPE--FEKISTQ    712
tr|A0A176WKM4|A0A176WKM4_MARPO          RLSEKDIKEAVARMEKLHLKDAEKRKTAEAKNSLEAYIYATK--DKLESLEG-IETVSTE    717
tr|A0A3R7K087|A0A3R7K087_9TRYP          NLNKTETMAARDRLRAFQRIDDERLMRSAIRNDLEALILHYKSLDAWDKAT-----VG-S    842
tr|Q4E175|Q4E175_TRYCC                  NLNKLEAAAARDRLYAFQRVDDERLMRSALRNDIETLILHYKSLDAWDKAV------E-N    836
tr|Q4E0G9|Q4E0G9_TRYCC                  NLNKLEAAAARDRLYAFQRVDDERLMRSALRNDIETLILHYKSLDAWDKAV------E-N    836
tr|A0A6A5CDJ2|A0A6A5CDJ2_NAEFO          PLTPEQLKASREKVKNVEDFEALKRRISAARNNLESALYS-TREKLLDNPD-YKGFFT-T    657
tr|A0A0S4IUZ2|A0A0S4IUZ2_BODSA          PMSKNESILSRDLLRRWRRSDELHLQKSAARNDLEAYLIWALSDGFVYNDE-ALKVVGSQ    782
tr|A0A0L1KXM1|A0A0L1KXM1_9EUGL          L-STTEFESEKLLLKEQEAYEASKRAIAQARNDLETLIIGYEGDALEAL-Q-ESVGIS-I    660
tr|A0A0S4JJU2|A0A0S4JJU2_BODSA          G-TEEVLE-AKSILSGLEKRDKIKRDTATSKNNLETFIQWIRLDGLFENNE-ATSLMS-T    667
```

```
tr|A0A0N0VI62|A0A0N0VI62_LEPPY    L-TLQESSASEKKLADIWKAEHAKHVRATAKNNLETYIFWVKYDGVGGNET-LAAAVG-D   655
tr|A4HND7|A4HND7_LEIBR            L-TSEEMESSQAKLKTIWHAEHVKHLRATAKNNLETYIFWTKYEGVADNAE-LTATAG-A   655
tr|A4IC10|A4IC10_LEIIN            L-TSEEMETSQTKLKTIWQAEHVKHLRATAKNNLEAYIFWAKNEGVSDNAA-LTAAAG-A   655
tr|E9AFU8|E9AFU8_LEIMA            L-TPEEMQTSQAKLEAIWQAEHVKHLRATAKNNLEAYIFWAKNDGVSDNAA-LTAAAG-A   654
tr|A0A7G2C9U8|A0A7G2C9U8_9TRYP    P-SKEELEKSKEKISLIETSERIKYERAHAKNDLETFIFWAKREGVLENSD-VTSKIT-E   541
tr|S9TX72|S9TX72_9TRYP            L-TEEEAAHIAERLFAVDEHERVKAERAHARNDLESFIFWAKREGILENTT-IVKKTD-A   654
tr|S9UBL2|S9UBL2_9TRYP            L-TEEEAAHIAERLFAVDEHERVKAERAHARNDLESFIFWAKREGILENTT-IVKKTD-A   654
tr|Q38E16|Q38E16_TRYB2            L-SYELLNNSRVKIFQLLEKERKKHEAATAKNNLETYLFWAKTEGILENTT-ALEPYT-P   653
tr|F9W5M9|F9W5M9_TRYCI            ------------------------------------------------------------   452
tr|A0A1X0P4V9|A0A1X0P4V9_9TRYP    L-SEENFQKSRKKILDILDKERRKHEAATAKNNLETYVFWVKREGVLENTS-FLSLLS-E   643
tr|Q4CS27|Q4CS27_TRYCC            L-SEEAFRRSKRRIAEILDKERKKHEAAVAKNNLEAYVFWAKSEGILENET-ALGVIS-T   655
tr|Q4E4F4|Q4E4F4_TRYCC            L-SEEAFRRSKKRIAEILDKERKKHEAAVAKNNLEAYVFWAKSEGILENET-ALGVIS-T   655
tr|A0A422P1B1|A0A422P1B1_TRYRA    L-SGEVYQHLSEKNSSDT------------------------------------------   609
tr|A0A422N0X0|A0A422N0X0_9TRYP    L-SLEAFQHSREKISLILEKEQMKHEAAVAKNNLEAYVFWAKSEGILGNST-ALGLLS-A   646


tr|A0A0C9MAJ6|A0A0C9MAJ6_9FUNG    SDIEKLRERLSEVSDWIYDEG-----EHADTPVFTSKLKELQLIEQPIQHRVREYNERDN   705
sp|Q9Y4L1|HYOU1_HUMAN             EQREEISGKLSAASTWLEDEG-----VGATTVMLKEKLAELRKLCQGLFFRVEERKKWPE   820
sp|Q63617|HYOU1_RAT               EQREEISGKLSATSTWLEDEG-----FGATTVMLKDKLAELRKLCQGLFFRVEERRKWPE   820
tr|A0A067CBX2|A0A067CBX2_SAPPC    EQRETLSAAVDATEEWLYEDG-----DDLDAKAYNERLSGMRKQLDDMVFRVSELTALPE   735
tr|D8RVG4|D8RVG4_SELML            KQRQEFLARLDEAGEWLYSDG-----EAATASEFKKRLGELKAIGDPIFFRLEQLTARPA   709
tr|A0A1P8B8R9|A0A1P8B8R9_ARATH    EERKAFVEKLDEVQDWLYMDG-----EDANATEFEKRLDSLKAIGSPISFRSEELTARPV   767
tr|A0A176WKM4|A0A176WKM4_MARPO    EQRDSLRAELNEAEDWLYTDG-----EDATAAEFKKKLDGLKKSGDAIFSRLDELIARPA   772
tr|A0A3R7K087|A0A3R7K087_9TRYP    AGEGNWREVVADVARWLDDAS-----DNVALAELQRQQRRMKELKTGDAATG--------   889
tr|Q4E175|Q4E175_TRYCC            EGEANWREVVKEVEQWLDDTS-----DNVEISELQRQQQRMNKLQIGGATTG--------   883
tr|Q4E0G9|Q4E0G9_TRYCC            EGEANWREVVKEVEQWLDDTS-----DNVEISELQRQQQRMNKLQIGGATTG--------   883
tr|A0A6A5CDJ2|A0A6A5CDJ2_NAEFO    KERDEINTVIDELEVWLGENDEEGVVEPSRVDLFTSKLKLLTDLTDPIQDRWREHKGRKE   717
tr|A0A0S4IUZ2|A0A0S4IUZ2_BODSA    EEMDTTAEKLRELQNWLEDA--G-TEATVPTEDLQAKLRQAKLIVRTALREAPTVEETKA   839
tr|A0A0L1KXM1|A0A0L1KXM1_9EUGL    EQTNVLLATISEVKEWLYDG--DGVSDSASVDSFRSRQQEILTIVEGIRYANINGSDDVD   718
tr|A0A0S4JJU2|A0A0S4JJU2_BODSA    EEKQTIEETTEQVKEWYEDG--EGSYDGCTKAEFDEKLALLKNVTATIREKIQEKENERK   725
tr|A0A0N0VI62|A0A0N0VI62_LEPPY    AAVHHVLDEVAKIQDWLEDG--PGSSDHCSADEYDARHAELERLVYELTKEPESESSHD-   712
tr|A4HND7|A4HND7_LEIBR            TAIQRVMDEVTAVQEWLEGG--EGALDHCAASEYDSRLGNLRKMVSELTKKPEEATNIV-   712
tr|A4IC10|A4IC10_LEIIN            AAVQRVIDEATAMQEWLEEG--EGASDNCAASQYESRLENLRGMVAELTKQPEEATKTV-   712
tr|E9AFU8|E9AFU8_LEIMA            AAVQRVIDEATAVQEWLEEG--EGASDHCAASQYESRLESLRGMVAELTKQPEEATSTV-   711
tr|A0A7G2C9U8|A0A7G2C9U8_9TRYP    KQLEEYAALLEKSLNWLEDG--EGSYDSCTGEMYENKLSELKETLQSYLPVEEPKTNST-   598
tr|S9TX72|S9TX72_9TRYP            KLLGEFEQQLRDALEWLEDG--DGSMDSCPTKEYKSLLSQLKEQASGIQKPLEPVPVPS-   711
tr|S9UBL2|S9UBL2_9TRYP            KLLGEFEQQLRDALEWLEDG--DGSMDSCPTKEYKSLLSQLKEQASGIQKPLEPVPVPS-   711
tr|Q38E16|Q38E16_TRYB2            EEVNSLKNALAEAQEWLEDG--DGSNESCSKEEYENKLATLKELVKNSGKNKNTT-NTT-   709
tr|F9W5M9|F9W5M9_TRYCI            ------------------------------------------------------------   452
tr|A0A1X0P4V9|A0A1X0P4V9_9TRYP    VEIDRLKGKLSEIQEWLEDG--EGSYETCTKEEYEKKLKELKDLVRQETQNNTETRNPQ-   700
tr|Q4CS27|Q4CS27_TRYCC            EKIEMLRKKLSEVQEWLENE--DCGSEPCSKEEYEKKMEEIKQIVRQEPDATNETEVVI-   712
tr|Q4E4F4|Q4E4F4_TRYCC            EKVEMLRAKLSEVQEWLEDE--DCGSEQCSKEEYEKKMEEIKQIVRQEPDANNENEVVI-   712
tr|A0A422P1B1|A0A422P1B1_TRYRA    ------------------------------------------------------------   609
tr|A0A422N0X0|A0A422N0X0_9TRYP    EEAEVLRAKLSEVQEWIEDG--ICSYEACSKDEFEKKLQEIKQLVAQKRQSSAEAEPLK-   703


tr|A0A0C9MAJ6|A0A0C9MAJ6_9FUNG    NINLVDSTIKLAREFVTNISQVADDLRYHTEEELDGLLTAAEKLEDWMAEKVAAQKKLAN   765
sp|Q9Y4L1|HYOU1_HUMAN             RLSALDNLLNHSSMFLKGARLIPEMDQIFTEVEMTTLEKVINETWAWKNATLAEQAKLPA   880
sp|Q63617|HYOU1_RAT               RLSALDNLLNHSSIFLKGARLIPEMDQIFTDVEMTTLEKVINDTWTWKNATLAEQAKLPA   880
tr|A0A067CBX2|A0A067CBX2_SAPPC    AVAKAQQYAISTIDLM---AQWVDAKPQVTADERDDVLAKVSELKDWLKEQQAAQDAIAK   792
tr|D8RVG4|D8RVG4_SELML            AMEAARGSLVESEAAI---HEWKEKKPWISD---AEVRKEGQKLVDWIEAKESEQAKVAD   763
tr|A0A1P8B8R9|A0A1P8B8R9_ARATH    AIEYARKYLTELKEII---KEWETNKTWLPKEKIDEVSKEAEKVKSWLDKNVAEQEKTSL   824
tr|A0A176WKM4|A0A176WKM4_MARPO    AVSEARTYLKTITETL---EEWEESKPWIPVKSKDDVRKEVESLRQWLDDMESKQSKLSG   829
tr|A0A3R7K087|A0A3R7K087_9TRYP    ------------------------------------------------------------   889
tr|Q4E175|Q4E175_TRYCC            ------------------------------------------------------------   883
tr|Q4E0G9|Q4E0G9_TRYCC            ------------------------------------------------------------   883
tr|A0A6A5CDJ2|A0A6A5CDJ2_NAEFO    ALSYCKSVFNATNYAI---HYMRENQKHITEDELNELHQFNKETEMNITSILAKQDRSPL   774
tr|A0A0S4IUZ2|A0A0S4IUZ2_BODSA    P-----------------------------------------------------------   840
tr|A0A0L1KXM1|A0A0L1KXM1_9EUGL    D-----------------------------------------------------------   719
tr|A0A0S4JJU2|A0A0S4JJU2_BODSA    P-----------------------------------------------------------   726
tr|A0A0N0VI62|A0A0N0VI62_LEPPY    ------------------------------------------------------------   712
tr|A4HND7|A4HND7_LEIBR            ------------------------------------------------------------   712
tr|A4IC10|A4IC10_LEIIN            ------------------------------------------------------------   712
tr|E9AFU8|E9AFU8_LEIMA            ------------------------------------------------------------   711
tr|A0A7G2C9U8|A0A7G2C9U8_9TRYP    ------------------------------------------------------------   598
tr|S9TX72|S9TX72_9TRYP            ------------------------------------------------------------   711
tr|S9UBL2|S9UBL2_9TRYP            ------------------------------------------------------------   711
tr|Q38E16|Q38E16_TRYB2            ------------------------------------------------------------   709
tr|F9W5M9|F9W5M9_TRYCI            ------------------------------------------------------------   452
tr|A0A1X0P4V9|A0A1X0P4V9_9TRYP    ------------------------------------------------------------   700
tr|Q4CS27|Q4CS27_TRYCC            ------------------------------------------------------------   712
tr|Q4E4F4|Q4E4F4_TRYCC            ------------------------------------------------------------   712
tr|A0A422P1B1|A0A422P1B1_TRYRA    ------------------------------------------------------------   609
tr|A0A422N0X0|A0A422N0X0_9TRYP    ------------------------------------------------------------   703


tr|A0A0C9MAJ6|A0A0C9MAJ6_9FUNG    TDDPVLVTSHVVDRAQTVKEHLVKLMSKKKPKVPKKTPEVPK--NDT-E--KEQNDSNED   820
sp|Q9Y4L1|HYOU1_HUMAN             TEKPVLLSKDIEAKMMALDREVQYLLNKAKFTKPRPRPKDKN--GTRAE--PPLNASASD   936
sp|Q63617|HYOU1_RAT               TEKPVLLSKDIEAKMMALDREVQYLLNKAKFTKPRPRPKDKN--GTRTE--PPLNASAGD   936
tr|A0A067CBX2|A0A067CBX2_SAPPC    HDEPVFTSAHVLKKISGIKKVVATLAKKPKPTP-KPTEEAKPAEGDEAKKDDEPTTSSDE   851
tr|D8RVG4|D8RVG4_SELML            HEQPAFSSTEVYARIEKFRVLVARTGAQKAPKP-PKIEEVVK--NDAGSEEPKVGEENPG   820
tr|A0A1P8B8R9|A0A1P8B8R9_ARATH    WSKPVFTSTEVYAKVFTLQDKVTKVNKIPKPKP--KIEKVTK--TENTTKEEEQSKSSDE   880
tr|A0A176WKM4|A0A176WKM4_MARPO    HVEPAFTSDLVYTEVSRLRGKVLLLSKLQKPKP--KVEKPTE--NIEAETKPSESSQVDD   885
tr|A0A3R7K087|A0A3R7K087_9TRYP    ------------------------------------------------------------   889
tr|Q4E175|Q4E175_TRYCC            ------------------------------------------------------------   883
tr|Q4E0G9|Q4E0G9_TRYCC            ------------------------------------------------------------   883
tr|A0A6A5CDJ2|A0A6A5CDJ2_NAEFO    YEAPTVRAKDIKKQCGKVSERAFLLSKKPVPKPEKPATNET---NETT--IPSTNEETKT   829
```

```
tr|A0A0S4IUZ2|A0A0S4IUZ2_BODSA    ---------------------------KAPSVTTESAE-TAA--EGEA--VDEEIEEGDD    868
tr|A0A0L1KXM1|A0A0L1KXM1_9EUGL    --------------------------------PTNEDS--VA--GEGD----------EN    733
tr|A0A0S4JJU2|A0A0S4JJU2_BODSA    ---------------------------KPAPVPKKQPKKRKP--ETPQ--EP--AEV-PE    752
tr|A0A0N0VI62|A0A0N0VI62_LEPPY    ----------------------------------ASNN--G---------------VGGA    721
tr|A4HND7|A4HND7_LEIBR            ----------------------------------TE------------------------    714
tr|A4IC10|A4IC10_LEIIN            ----------------------------------TT------------------------    714
tr|E9AFU8|E9AFU8_LEIMA            ----------------------------------TT------------------------    713
tr|A0A7G2C9U8|A0A7G2C9U8_9TRYP    ----------------------------------TQEVNSTV--GEED--VD--FEMGTD    618
tr|S9TX72|S9TX72_9TRYP            ----------------------------------ENDA----------------------    715
tr|S9UBL2|S9UBL2_9TRYP            ----------------------------------ENDA----------------------    715
tr|Q38E16|Q38E16_TRYB2            ----------------------------------SGSD----------------------    713
tr|F9W5M9|F9W5M9_TRYCI            ------------------------------------------------------------    452
tr|A0A1X0P4V9|A0A1X0P4V9_9TRYP    ----------------------------------ESDD----------------------    704
tr|Q4CS27|Q4CS27_TRYCC            ----------------------------------ESSD----------------------    716
tr|Q4E4F4|Q4E4F4_TRYCC            ----------------------------------ESSD----------------------    716
tr|A0A422P1B1|A0A422P1B1_TRYRA    ------------------------------------------------------------    609
tr|A0A422N0X0|A0A422N0X0_9TRYP    ----------------------------------EATG----------------------    707

tr|A0A0C9MAJ6|A0A0C9MAJ6_9FUNG    S---EKKQQEEATQS---E--IPAAT-------SEEHEH---------------------    844
sp|Q9Y4L1|HYOU1_HUMAN             Q---GEKVIPPAGQT---EDAEPISEPEKVETGSEPGDTEPLELGGPGAEPEQKEQSTGQ    990
sp|Q63617|HYOU1_RAT               Q---EEKVIPPTGQT---EEAKAILEPDKEGLGTEAADSEPLELGGPGAESEQAEQTAGQ    990
tr|A0A067CBX2|A0A067CBX2_SAPPC    E----------PK--------TP------EPTEEE-------------------------    862
tr|D8RVG4|D8RVG4_SELML            ASSGGGDSNSGSTQEGEREIESPDQAQAFEDESST-------------------------    855
tr|A0A1P8B8R9|A0A1P8B8R9_ARATH    A----------AKEEESHDEL---------------------------------------    891
tr|A0A176WKM4|A0A176WKM4_MARPO    T----------AKQEEKIEETVPQGEGAEMNQESK-------------------------    910
tr|A0A3R7K087|A0A3R7K087_9TRYP    ------------------------------------------------------------    889
tr|Q4E175|Q4E175_TRYCC            ------------------------------------------------------------    883
tr|Q4E0G9|Q4E0G9_TRYCC            ------------------------------------------------------------    883
tr|A0A6A5CDJ2|A0A6A5CDJ2_NAEFO    E---ENTANSQEQQS-----TNE-------------------------------------    844
tr|A0A0S4IUZ2|A0A0S4IUZ2_BODSA    D---GA--------D-----S---------------------------------------    873
tr|A0A0L1KXM1|A0A0L1KXM1_9EUGL    A---PN--------D-----P---------------------------------------    738
tr|A0A0S4JJU2|A0A0S4JJU2_BODSA    E---PT--------E-----ANP-------------------------------------    759
tr|A0A0N0VI62|A0A0N0VI62_LEPPY    A---SN--------E-----GD--------------------------------------    727
tr|A4HND7|A4HND7_LEIBR            --------------S-----SD--------------------------------------    717
tr|A4IC10|A4IC10_LEIIN            --------------D-----SA--------------------------------------    717
tr|E9AFU8|E9AFU8_LEIMA            --------------D-----SA--------------------------------------    716
tr|A0A7G2C9U8|A0A7G2C9U8_9TRYP    E---PA--------E-----PD--------------------------------------    624
tr|S9TX72|S9TX72_9TRYP            --------------A-----AT--------------------------------------    718
tr|S9UBL2|S9UBL2_9TRYP            --------------A-----AT--------------------------------------    718
tr|Q38E16|Q38E16_TRYB2            --------------D-----A---------------------------------------    715
tr|F9W5M9|F9W5M9_TRYCI            ------------------------------------------------------------    452
tr|A0A1X0P4V9|A0A1X0P4V9_9TRYP    --------------D-----A---------------------------------------    706
tr|Q4CS27|Q4CS27_TRYCC            --------------D---------------------------------------------    717
tr|Q4E4F4|Q4E4F4_TRYCC            --------------D---------------------------------------------    717
tr|A0A422P1B1|A0A422P1B1_TRYRA    ------------------------------------------------------------    609
tr|A0A422N0X0|A0A422N0X0_9TRYP    --------------D---------------------------------------------    708

tr|A0A0C9MAJ6|A0A0C9MAJ6_9FUNG    ------DEL    847
sp|Q9Y4L1|HYOU1_HUMAN             KRPLKNDEL    999
sp|Q63617|HYOU1_RAT               KRPLKNDEL    999
tr|A0A067CBX2|A0A067CBX2_SAPPC    --PTHSDEL    869
tr|D8RVG4|D8RVG4_SELML            --TPLRDEL    862
tr|A0A1P8B8R9|A0A1P8B8R9_ARATH    ---------    891
tr|A0A176WKM4|A0A176WKM4_MARPO    --PTEHDEL    917
tr|A0A3R7K087|A0A3R7K087_9TRYP    ---------    889
tr|Q4E175|Q4E175_TRYCC            ---------    883
tr|Q4E0G9|Q4E0G9_TRYCC            ---------    883
tr|A0A6A5CDJ2|A0A6A5CDJ2_NAEFO    --QKQKDEL    851
tr|A0A0S4IUZ2|A0A0S4IUZ2_BODSA    --GASRDEL    880
tr|A0A0L1KXM1|A0A0L1KXM1_9EUGL    ------EEL    741
tr|A0A0S4JJU2|A0A0S4JJU2_BODSA    --EVPQEEL    766
tr|A0A0N0VI62|A0A0N0VI62_LEPPY    --DGAEGDL    734
tr|A4HND7|A4HND7_LEIBR            --DYNEDDL    724
tr|A4IC10|A4IC10_LEIIN            --DDNEGDL    724
tr|E9AFU8|E9AFU8_LEIMA            --DDNEDDL    723
tr|A0A7G2C9U8|A0A7G2C9U8_9TRYP    --ETLEGDL    631
tr|S9TX72|S9TX72_9TRYP            --SEGEEDL    725
tr|S9UBL2|S9UBL2_9TRYP            --SEGEEDL    725
tr|Q38E16|Q38E16_TRYB2            ----VKGDL    720
tr|F9W5M9|F9W5M9_TRYCI            ---------    452
tr|A0A1X0P4V9|A0A1X0P4V9_9TRYP    ----VKGDL    711
tr|Q4CS27|Q4CS27_TRYCC            ----DRGDL    722
tr|Q4E4F4|Q4E4F4_TRYCC            ----DRGDL    722
tr|A0A422P1B1|A0A422P1B1_TRYRA    ---------    609
tr|A0A422N0X0|A0A422N0X0_9TRYP    ----DRGDL    713
```

(overall sequence identity = 0.0132)

11. Shewanella-like phosphatases

The Shewanella-like phosphatases are an unusual family of enzymes with both prokaryotic and eukaryotic members. Although not present in animals, it might be an

important enzyme for several organisms, e.g. fungi, and might not only act as a protein phosphatase. The KDEL-containing branch we identified in kinetoplastids seems to be ancient, as other euglenids also seem to possess shewanella-like phosphatases of essentially the same architecture.

```
tr|C5L0A1|C5L0A1_PERM5              ------------------------------------------------------------    0
tr|A0A0S4JBT9|A0A0S4JBT9_BODSA      ------------------------------------------------------------    0
tr|Q583S8|Q583S8_TRYB2              ------------------------------------------------------------    0
tr|A0A1X0NU01|A0A1X0NU01_9TRYP      ------------------------------------------------------------    0
tr|A0A3R7MVL3|A0A3R7MVL3_9TRYP      ------------------------------------------------------------    0
tr|Q4DFK9|Q4DFK9_TRYCC              ------------------------------------------------------------    0
tr|Q4DQ38|Q4DQ38_TRYCC              ------------------------------------------------------------    0
tr|A0A0N0VHG5|A0A0N0VHG5_LEPPY      ------------------------------------------------------------    0
tr|A4HCJ2|A4HCJ2_LEIBR              ------------------------------------------------------------    0
tr|A4I008|A4I008_LEIIN              ------------------------------------------------------------    0
tr|Q4QBJ8|Q4QBJ8_LEIMA              ------------------------------------------------------------    0
sp|Q944L7|SLP2_ARATH                ------------------------------------------------------------    0
tr|A0A813HX77|A0A813HX77_POLGL      MSFAFMAVLGLSQDVVFSVEQAIEQDMVVNANFAFSHNMGHKGLEDAHSFADIFSWLRLG    60
tr|A0A0S4JMN0|A0A0S4JMN0_BODSA      ------------------------------------------------------------    0
tr|Q4CWC2|Q4CWC2_TRYCC              ------------------------------------------------------------    0
tr|A0A1Y2EN62|A0A1Y2EN62_9FUNG      ------------------------------------------------------------    0
tr|A0A0C9MUE8|A0A0C9MUE8_9FUNG      ------------------------------------------------------------    0

tr|C5L0A1|C5L0A1_PERM5              ------------------------------------------------------------    0
tr|A0A0S4JBT9|A0A0S4JBT9_BODSA      --------------------------------------------MVVFFLMLPQQERNKI    16
tr|Q583S8|Q583S8_TRYB2              ------------------------------------------------------------    0
tr|A0A1X0NU01|A0A1X0NU01_9TRYP      ------------------------------------------------------------    0
tr|A0A3R7MVL3|A0A3R7MVL3_9TRYP      ------------------------------------------------------------    0
tr|Q4DFK9|Q4DFK9_TRYCC              ------------------------------------------------------------    0
tr|Q4DQ38|Q4DQ38_TRYCC              ------------------------------------------------------------    0
tr|A0A0N0VHG5|A0A0N0VHG5_LEPPY      -----------------------------------------------------------M    1
tr|A4HCJ2|A4HCJ2_LEIBR              -----------------------------------------------------------M    1
tr|A4I008|A4I008_LEIIN              -----------------------------------------------------------M    1
tr|Q4QBJ8|Q4QBJ8_LEIMA              -----------------------------------------------------------M    1
sp|Q944L7|SLP2_ARATH                ------------------------------------------------------------    0
tr|A0A813HX77|A0A813HX77_POLGL      FLPLVIQPSWSYSESRDADSASVFDLAPMRASVSNFLVRQIQQDGIPFFLFHPSTFRETS    120
tr|A0A0S4JMN0|A0A0S4JMN0_BODSA      ------------------------------------------------------------    0
tr|Q4CWC2|Q4CWC2_TRYCC              ------------------------------------------------------------    0
tr|A0A1Y2EN62|A0A1Y2EN62_9FUNG      ------------------------------------------------------------    0
tr|A0A0C9MUE8|A0A0C9MUE8_9FUNG      ------------------------------------------------------------    0

tr|C5L0A1|C5L0A1_PERM5              ------------------------------------------------------------    0
tr|A0A0S4JBT9|A0A0S4JBT9_BODSA      ----------------------------NNNPSYNMRL-SLTIVTLCL--VA--------    37
tr|Q583S8|Q583S8_TRYB2              -------------------------------------MVGKYLLISLVLVAH--------    15
tr|A0A1X0NU01|A0A1X0NU01_9TRYP      -----------------------------------MRLVTPF--FVIILLVY--------    15
tr|A0A3R7MVL3|A0A3R7MVL3_9TRYP      -----------------------------------MKLAALLLLQLCLIIVT--------    17
tr|Q4DFK9|Q4DFK9_TRYCC              -----------------------------------MRLVEFLLPLLVILLGF--------    17
tr|Q4DQ38|Q4DQ38_TRYCC              -----------------------------------MRLVEFLLPLLVILLGF--------    17
tr|A0A0N0VHG5|A0A0N0VHG5_LEPPY      ----------------------------RRCAVNYYFTFFALVTSICSVLVA--------    25
tr|A4HCJ2|A4HCJ2_LEIBR              ----------------------------RNYVLRAVRCYSALLVSFSLLLVS--------    25
tr|A4I008|A4I008_LEIIN              ----------------------------KVCALRIGRCYGALLLLFYLLLIS--------    25
tr|Q4QBJ8|Q4QBJ8_LEIMA              ----------------------------KVCDLQIGRCYGALLLLFYVLLIS--------    25
sp|Q944L7|SLP2_ARATH                -------------------------MSSRENPSG------ICKSIPKLI-----------    18
tr|A0A813HX77|A0A813HX77_POLGL      SSDERGYIRFFTHTRGISFLASYVFIKFGARPAI-LAAFLIQKHVFNVFNVFRSRASLSE    179
tr|A0A0S4JMN0|A0A0S4JMN0_BODSA      -----------------------------------MNASLFATSIFVVLTLL--------    17
tr|Q4CWC2|Q4CWC2_TRYCC              -------------MRGV----------L-------------------LLLLF--------    10
tr|A0A1Y2EN62|A0A1Y2EN62_9FUNG      -------------MSDI----------LSANPSLTTSTSVFQSK-STSTVVP--------    28
tr|A0A0C9MUE8|A0A0C9MUE8_9FUNG      ----------------M----------V---------------K-FTSFLSA--------    10

tr|C5L0A1|C5L0A1_PERM5              ----------MTFHRI---------AVVAALLVVSVATSSSRPLKRIVAVADVHGDRRNL    41
tr|A0A0S4JBT9|A0A0S4JBT9_BODSA      -TTLFVDA-----------------------------RDAAGAPRRIIAVGDVHGDLAQL    67
tr|Q583S8|Q583S8_TRYB2              -LGIRCCANV--------------------DANPLSRNVIPIEIHRIVAVGDVHGDAERF    54
tr|A0A1X0NU01|A0A1X0NU01_9TRYP      -IPYFCEGES--------------------RSNLETHDTIPIEIHRIIAVGDVHGDLDHF    54
tr|A0A3R7MVL3|A0A3R7MVL3_9TRYP      -TVDVCYGES--------------------RANTHIGTTVPLEIHRIIAVGDVHGDADNF    56
tr|Q4DFK9|Q4DFK9_TRYCC              -PVVFCYGEG--------------------TASGGDVHAIPIEIHRIIAVGDVHGDTENF    56
tr|Q4DQ38|Q4DQ38_TRYCC              -PVVFCYGES--------------------TASGGDVHAIPIEIHRIIAVGDVHGDTENF    56
tr|A0A0N0VHG5|A0A0N0VHG5_LEPPY      -TPAAAT-------------------------------KELVEIHRIVTVGDVHGDAENF    53
tr|A4HCJ2|A4HCJ2_LEIBR              -FPVHCE-------------------------------RKLVEVHRIIAVGDVHGDADNF    53
tr|A4I008|A4I008_LEIIN              -CLARCE-------------------------------RKLVEVHRIIAVGDVHGDADNF    53
tr|Q4QBJ8|Q4QBJ8_LEIMA              -CFARCE-------------------------------RKLVEVHRIIAVGDVHGDADNF    53
sp|Q944L7|SLP2_ARATH                ------SSFVDTFVDYSVSGIFLPQDP---SSQNEILQTRFEKPERLVAIGDLHGDLEKS    69
tr|A0A813HX77|A0A813HX77_POLGL      CAPMGCNCQMLTSVELS-GGRWLRRWHPLLGVTFAVLAAGSSSPSRVVALADLHGDYDHA    238
tr|A0A0S4JMN0|A0A0S4JMN0_BODSA      ----NLASP---------------------HDGMASALFGYHPPPRLLAIGDLHGDITNA    52
tr|Q4CWC2|Q4CWC2_TRYCC              ----GAA------------------AYFVAFL-HVEAVNVRGTEPRLVAFGDIHGDVFRC    47
tr|A0A1Y2EN62|A0A1Y2EN62_9FUNG      ----GEQSKEDT-LDST------KKDSSTKEVKIEKRKKSEKVFKRVVAVGDIHGDYKKL    77
tr|A0A0C9MUE8|A0A0C9MUE8_9FUNG      ----GC----------------------LLFSSLVSAAISVEQDRRIVALGDLHGDLANT    44
                                                                             *:::..*:***  .

tr|C5L0A1|C5L0A1_PERM5              MQALENGRVLVHKHGPEQE-------------------GVEWHPEAS--DPAEPVMEGTQ    80
```

```
tr|A0A0S4JBT9|A0A0S4JBT9_BODSA    RAVLEMANVIND--------------------------EAQWIDRD-----------NTV    90
tr|Q583S8|Q583S8_TRYB2            RQILEMSGVISLRSNSSKQ----V--------VW----KPRWGTKEGNFFREYGTRLRTT    98
tr|A0A1X0NU01|A0A1X0NU01_9TRYP    LKILSMADVIAFPNNDTND----V--------VW----KPKWENKEIELHRTHRTKLRTT    98
tr|A0A3R7MVL3|A0A3R7MVL3_9TRYP    RQILFMTGIISYSSNTDKN----V--------IW----KPMWDEKEIELRKSHRTKLRTT    100
tr|Q4DFK9|Q4DFK9_TRYCC            RQILSMAGIVSYSSNADKQ----V--------LW----KPMWDEKEIELHKRHHTKLRST    100
tr|Q4DQ38|Q4DQ38_TRYCC            RQILSMAGIVSYSSNADKQ----L--------LW----KPMWDEKEIELHKRHHTKLRST    100
tr|A0A0N0VHG5|A0A0N0VHG5_LEPPY    LKVLRLADVVEEGTGGVVD----V--------LTN---PPQWKFSQ---APNASVTSWTT    95
tr|A4HCJ2|A4HCJ2_LEIBR            LKILRIANLIEDSASGASD----V--------LDS---PPRWKYSS---SQISDTTVRTT    95
tr|A4I008|A4I008_LEIIN            LKILRIANLIEDGVTGASG----V--------LDN---PPRWKYSS---SRPNGTAVRTT    95
tr|Q4QBJ8|Q4QBJ8_LEIMA            LKILRIANLIEDSVTGASG----V--------LDN---PPRWKYSS---SRTNGTSVRTT    95
sp|Q944L7|SLP2_ARATH              REAFKIAGLIDSS--------------------------DRWTGG------------STM    91
tr|A0A813HX77|A0A813HX77_POLGL    LAILRAAGLADENSEIGVSEADELLVAEAARKPWSRFRGVQWLGG------------NAT    286
tr|A0A0S4JMN0|A0A0S4JMN0_BODSA    KEILKFAQIVDCE--------------------------GKWIAG------------HDT    74
tr|Q4CWC2|Q4CWC2_TRYCC            RQILQLANITDTE--------------------------DRWIAG------------SSI    69
tr|A0A1Y2EN62|A0A1Y2EN62_9FUNG    IKVLYTAKIIDNK--------------------------SNWIAK------------DTI    99
tr|A0A0C9MUE8|A0A0C9MUE8_9FUNG    LEILRFSNIIDQD--------------------------NHWIGG------------DTI    66
                                           :       :                                 *

tr|C5L0A1|C5L0A1_PERM5            VVQLGDLVDRGPLGLQCYRLMQDLYVAE----GANEVVRVLGNHEVLNLLGMAGRYVTDE    136
tr|A0A0S4JBT9|A0A0S4JBT9_BODSA    VVQVGDLLDRGPHDKGVLDFMMQLQAEAPT--HGGKVVSILGNHELMNIMNQ-VHYVHPE    147
tr|Q583S8|Q583S8_TRYB2            LIQTGDLIDRGEEDLEVLEMAVSLFNEVRTNYTDDKVVLLMGNHELLNLQGH-FHYVHSK    157
tr|A0A1X0NU01|A0A1X0NU01_9TRYP    LVQMGDLIDRGANDLGVLEMVFSLFDQVKKNHTSDNLVLLLGNHELLNLQGQ-FYYVEPE    157
tr|A0A3R7MVL3|A0A3R7MVL3_9TRYP    LIQMGDLIDRGEDDLAVLEMAASLLDQVKSNHTSDNFVLLLGNHELLNLQQQ-FHYVHPE    159
tr|Q4DFK9|Q4DFK9_TRYCC            LIQMGDLIDRGEDDLGVLEMAFSLFEQVKSNHTSDNIVLLLGNHELLNLQEQ-FYYVHPE    159
tr|Q4DQ38|Q4DQ38_TRYCC            LIQMGDLIDRGEDDLGVLEMAFSLFEQVKSNHTSDNIVLLLGNHELLNLQEQ-FYYVHPE    159
tr|A0A0N0VHG5|A0A0N0VHG5_LEPPY    LVQMGDLVDRGEQDFESLNIAMALQEQTQNSLTSDRVILLIGNHELLNIQGH-YHYVNKR    154
tr|A4HCJ2|A4HCJ2_LEIBR            LVQVGDLIDRGEQDLETLNIAISLQEQTAQSGSQDKVVLLIGNHELLNLQGH-YHYVNEK    154
tr|A4I008|A4I008_LEIIN            LVQVGDLIDRGEQDLQALNIAISLQEQTAQSGTQDEVVLLIGNHELLNIQGH-YHYVNKN    154
tr|Q4QBJ8|Q4QBJ8_LEIMA            LVQVGDLIDRGEQDLEALNIAISLQEQTAQSGSQDEVVLLIGNHELLNIQGH-YHYVNKH    154
sp|Q944L7|SLP2_ARATH              VVQVGDVLDRGGEELKILYFLEKLKREAER--AGGKILTMNGNHEIMNIEGD-FRYVTKK    148
tr|A0A813HX77|A0A813HX77_POLGL    LVQTGDLVDRGPFARDLYALFSELRRQAPL--AGGQVINLIGNHEAMNVLGQ-LKYVTKE    343
tr|A0A0S4JMN0|A0A0S4JMN0_BODSA    VIQVGDVVDRGPHGHEIIDYFTTLKEDARQ--SGGEFIQLLGNHELMNMQGN-MKFANAQ    131
tr|Q4CWC2|Q4CWC2_TRYCC            VVQLGDIADRGLHPHEIYDLFASLERQAMK--AGGEFIFLVGNHELMNILGI-FYYVHPD    126
tr|A0A1Y2EN62|A0A1Y2EN62_9FUNG    LIQTGDLIDRGNDTILIFDLMMKLKEQAKK--HGCILYLLLGNHELMNLQED-YRYVTRG    156
tr|A0A0C9MUE8|A0A0C9MUE8_9FUNG    FVQTGDVLDRGLDTIKLYDLLQNLRDEAPL--QGGLVIPLLGNHELMNLVGD-WRYVYPG    123
                                  .:* **: ***               *                .  : **** :*:    :.

tr|C5L0A1|C5L0A1_PERM5            DVAEF------------------------------------------GGEEARRESWSP-    153
tr|A0A0S4JBT9|A0A0S4JBT9_BODSA    SIEYF------------------------------------------EGSVNRKRQFSA-    164
tr|Q583S8|Q583S8_TRYB2            SMGGF------------------------------------------LTRALRKRAFEL-    174
tr|A0A1X0NU01|A0A1X0NU01_9TRYP    SMGGF------------------------------------------LTKTLRKRAFEK-    174
tr|A0A3R7MVL3|A0A3R7MVL3_9TRYP    SMGGF------------------------------------------LTKALRKRAFES-    176
tr|Q4DFK9|Q4DFK9_TRYCC            TMGGF------------------------------------------LSKTLRKRAFEP-    176
tr|Q4DQ38|Q4DQ38_TRYCC            TMGGF------------------------------------------LSKTLRKRAFEP-    176
tr|A0A0N0VHG5|A0A0N0VHG5_LEPPY    NYGGF------------------------------------------ISRPLRMEAMNA-    171
tr|A4HCJ2|A4HCJ2_LEIBR            NHGGF------------------------------------------MSKALRAEGMKV-    171
tr|A4I008|A4I008_LEIIN            NYGGF------------------------------------------LSKALRAEGMKA-    171
tr|Q4QBJ8|Q4QBJ8_LEIMA            NYGGF------------------------------------------LSKALRAEGMKA-    171
sp|Q944L7|SLP2_ARATH              GLEEFQIWADWYCLGNKMKTLCSGLDKPKDPYEGIPMSFPRMRADCFEGIRARIAALRP-    207
tr|A0A813HX77|A0A813HX77_POLGL    DTDEF------------------------------------------GSHAEREQAFAA-    360
tr|A0A0S4JMN0|A0A0S4JMN0_BODSA    TVTAF------------------------------------------GGMESLKQAFDPI    149
tr|Q4CWC2|Q4CWC2_TRYCC            VMSAF------------------------------------------GGEEAYAAAFGP-    143
tr|A0A1Y2EN62|A0A1Y2EN62_9FUNG    DVISF------------------------------------------GGMANRRKEFSL-    173
tr|A0A0C9MUE8|A0A0C9MUE8_9FUNG    EPETF------------------------------------------GGLEARRKAFEK-    140
                                           *

tr|C5L0A1|C5L0A1_PERM5            GGEIWTILKDHYELVHVYGGHRFVHGGVMPALTDRSIDELNEQASRMIKN----------    203
tr|A0A0S4JBT9|A0A0S4JBT9_BODSA    EGVYGQWIRQN-PLLHVEGSTLFVHAGLSPQFAAKGVDALNKEAKEALSQ----------    213
tr|Q583S8|Q583S8_TRYB2            DGTFGGFILENFTVAYAVADTLFVHAGIDEHVVSDGIERLNREAKQAIRT----------    224
tr|A0A1X0NU01|A0A1X0NU01_9TRYP    NGEFGSFILENFTVLHLEDSTVFVHAGLNEQVASMGVEAINKVTKQAVQD----------    224
tr|A0A3R7MVL3|A0A3R7MVL3_9TRYP    NGIFGRFLLYNFTVAHFDAETLFVHAGLNEHFASMGVDRLNKETMQAVRE----------    226
tr|Q4DFK9|Q4DFK9_TRYCC            SGTFGRFLLDKFNVLYFDAETVFVHAGIDQRFASVGVEMLNKKTMQAIRE----------    226
tr|Q4DQ38|Q4DQ38_TRYCC            SGTFGRFLLDKFNVLYFDAETVFVHAGIDQHFASVGVEMLNKKTMQAIRE----------    226
tr|A0A0N0VHG5|A0A0N0VHG5_LEPPY    DGAFGKYIIDNFRTAYLDENTLFVHAGIEADMLLPDLNSLNSEVREALRK----------    221
tr|A4HCJ2|A4HCJ2_LEIBR            TGVFGKYIVDNFKVAHIDEGVLFVHGGIETGMNIKDVDALNEDVRSALRQ----------    221
tr|A4I008|A4I008_LEIIN            TGAFGKYIVDNFKAAHMDEGVLFVHAGIETSMNIKDVEALNADIREALRQ----------    221
tr|Q4QBJ8|Q4QBJ8_LEIMA            TGAFGKYIVDNFKAAHMDEGVLFIHAGIETSMNIKDVEALNADVREALRQ----------    221
sp|Q944L7|SLP2_ARATH              DGPIAKRFLTKNQTVAVVGDSVFVHGGLLAEHIEYGLERINEEVRGWING---------F    258
tr|A0A813HX77|A0A813HX77_POLGL    GGWVGRQILEEFKALAVVAETLFVHAGLLPEHAAMGVESLDLQVRTDLAK-----AAASR    415
tr|A0A0S4JMN0|A0A0S4JMN0_BODSA    TGAYGTYLTKTCDAAVLRNRTLFVHAGVLPQYAHLGVAELNRQVRNAILA----------    199
tr|Q4CWC2|Q4CWC2_TRYCC            EGPYGLYILQ-HPVTVVREGVVFAHAGITPEYAAKGVEGINAELMNGFRGERDLLLEGDA    202
tr|A0A1Y2EN62|A0A1Y2EN62_9FUNG    DGKYGKLLRSEMNATMIIDDTLFVHAGLTTVFAKYGVDQMNDHIHYILKT----YPADQL    229
tr|A0A0C9MUE8|A0A0C9MUE8_9FUNG    DGFIGEYLKL-LNITTKVGSTVFCHGGITPHFSRLGINWINDKTHETIVT----YMESQG    195
                                  *        :                * *.*:           .:  ::   .

tr|C5L0A1|C5L0A1_PERM5            -GALKN----PLL-LSESSPLWSRVYALGTD-EEACPPLLNVLRHY----------GVAR    246
tr|A0A0S4JBT9|A0A0S4JBT9_BODSA    -KSLRHLDRSALF--GVHGPIWTRLLITDAM-NGRCKDVEESLSLL---------PGIER    260
tr|Q583S8|Q583S8_TRYB2            -KNFGH----ILL--GSTGPLWSRKMFLDAS-NGRCADTKKALASL----------GVKR    266
tr|A0A1X0NU01|A0A1X0NU01_9TRYP    -KDYRH----PLL--GSSGPLWTRQMIMDAM-NGHCTNIQKMLLAI----------GAER    266
tr|A0A3R7MVL3|A0A3R7MVL3_9TRYP    -KDYQN----PLL--GAFGPLWTRRMLMDAT-NGHCTGIKKMISSI----------GAKR    268
tr|Q4DFK9|Q4DFK9_TRYCC            -KDYGN----PLL--GTSGPLWTRKMITDAA-NGRCTGIQKMLSFI----------GAKR    268
tr|Q4DQ38|Q4DQ38_TRYCC            -KDYGN----PLL--GTSGPLWTRKMITDAA-NGRCTGIQKMLSFI----------GAKR    268
tr|A0A0N0VHG5|A0A0N0VHG5_LEPPY    -RDFRH----AYL--RSNGPLWTRKMISDSM-MGDCDEVNKILQRF----------NVSR    263
tr|A4HCJ2|A4HCJ2_LEIBR            -NIFRH----SFL--RSSGPLWTRKMIMESM-SDECADVEAALKQL----------NASR    263
tr|A4I008|A4I008_LEIIN            -GIFRH----SFL--GSSGPLWTRKMIIESM-SEGCSDVRAALKQL----------NATR    263
tr|Q4QBJ8|Q4QBJ8_LEIMA            -GIFRH----SFL--GSSGPLWTRKMIIESM-SDECSDVRAALKQL----------NATR    263
sp|Q944L7|SLP2_ARATH              KGGRYA----PAYCRGGNSVVWLRKFSEEMAHKCDCAALEHALST---------IPGVKR    305
```

```
tr|A0A813HX77|A0A813HX77_POLGL    SGGHGQ----PLL--KSNGPLWVRQFAKGQD-SKVCPLLAATLKLVG----------AKR    458
tr|A0A0S4JMN0|A0A0S4JMN0_BODSA    -KNISD----PIL--LDDGPLWTRQIIYPAQ-KGNCSNLYESLKILSEYETKHGRKPVDR    251
tr|Q4CWC2|Q4CWC2_TRYCC            KGDGVH----PLS--NSSSPLWSRAVLDEAK-RGNCSLLMESLRLLSEHELASGRPPVRV    255
tr|A0A1Y2EN62|A0A1Y2EN62_9FUNG    ---FYA----PIF--SNNGPFWTRFLSMGQE-VAVCEELNMVLEMMN----------VKR    269
tr|A0A0C9MUE8|A0A0C9MUE8_9FUNG    HRGKSH----GIF--GGVGPTWYRGYALEEE-DEICSVLDEALELLE----------ANR    238
                                                   .  * *           *        :

tr|C5L0A1|C5L0A1_PERM5            MVVGHTPSEDGRMKVRCGGRAILADVALSRWMGR----------YPHHGHPAALEMTLVN    296
tr|A0A0S4JBT9|A0A0S4JBT9_BODSA    MVVGHTPQRSGRVETYCDDKLIAVDVGLSKWMY---------------GNLAALEITQKV    305
tr|Q583S8|Q583S8_TRYB2            VVVGHTPQRSGRVETFCGGSVIAIDVGMSRWMY---------------GNIAALEITVTS    311
tr|A0A1X0NU01|A0A1X0NU01_9TRYP    IVVGHTPQRSGHVETYCDDSVIAVDVGLSKWMY---------------GNLAALEILVKT    311
tr|A0A3R7MVL3|A0A3R7MVL3_9TRYP    IVVGHTPQRSGHVELFCDDSVIAVDVGLSRWMY---------------GNLAALELMVTT    313
tr|Q4DFK9|Q4DFK9_TRYCC            IVVGHTPQRSGHVEVFCNDSVIAIDVGLSRWMY---------------GNLAALELMVTS    313
tr|Q4DQ38|Q4DQ38_TRYCC            IVVGHTPQRSGHVEVFCNDSVIAIDVGLSRWMY---------------GNLAALELMVTS    313
tr|A0A0N0VHG5|A0A0N0VHG5_LEPPY    IVVGHTPQDSGQIEQYCNKRVIAADVGMSRWMY---------------NNVAALEMVFFK    308
tr|A4HCJ2|A4HCJ2_LEIBR            VVVGHTPQESGHIGQYCGGQVLAIDVGISRWMY---------------DRAVALELVFLK    308
tr|A4I008|A4I008_LEIIN            VVVGHTPQESGHIGQHCDGQVFAIDVGMSRWMY---------------DKVAALELVFSK    308
tr|Q4QBJ8|Q4QBJ8_LEIMA            IVVGHTPQESGHIGQHCDGQVLAIDVGMSRWMY---------------DKVAALELVFSK    308
sp|Q944L7|SLP2_ARATH              MIMGHTIQDAG-INGVCNDKAIRIDVGMSKGCAD--------------GLPEVLEIRRDS    350
tr|A0A813HX77|A0A813HX77_POLGL    MVIGHTQVDEGEIRQRCGNRLLLADTIISRQGYPECWEPDSWQRERCKGSLSYVELRGDE    518
tr|A0A0S4JMN0|A0A0S4JMN0_BODSA    MVIGHTIMQDGAIHQFCDGKLIAIDIAISQYMA------------SGGRQ-GILELRYDF    298
tr|Q4CWC2|Q4CWC2_TRYCC            MVGGHTVQEGGVMAVECNGSLVGADVGLSRFFS------------SYGGYVAYVEFLLDD    303
tr|A0A1Y2EN62|A0A1Y2EN62_9FUNG    MVVGHTVQENRKINTRCGNKFLLIDVGMSEFYG------------GAFAY---LEFLNDK    314
tr|A0A0C9MUE8|A0A0C9MUE8_9FUNG    MVVGHTVQHDGAIRTRCNGKVILIDIGISRAYN------------SGKGA---LEIRGNV    283
                                  :: ***   :   *.   .  *  :*.                          :*:

tr|C5L0A1|C5L0A1_PERM5            ATHLEKIEA-------------------HY-GPENDTGAGQHQLLWTNTD-GLIEVGKDE    335
tr|A0A0S4JBT9|A0A0S4JBT9_BODSA    D--GSVELREIIPHNVATPPAAPPAIETEGDNHDAKQGSGIEGGVSDPILLQELMEAIQE    363
tr|Q583S8|Q583S8_TRYB2            YSSGDGVEEEVVLH--------------EVLASDARRNGTLEDFLNDTLFLEELQHAVEE    357
tr|A0A1X0NU01|A0A1X0NU01_9TRYP    HKIGSGAKKSVEIR--------------EIIASDTRRSQTLDEALDDSLVLEELQHAVEE    357
tr|A0A3R7MVL3|A0A3R7MVL3_9TRYP    YKSDSGIWRDVVMR--------------EVTTSDSRRFRTLDESLTDPLLLEELNHAVEE    359
tr|Q4DFK9|Q4DFK9_TRYCC            YKCDLGICRDVVMR--------------EITNSDARRSQTIDESLKDSLLLEELMHAVEE    359
tr|Q4DQ38|Q4DQ38_TRYCC            YKCDLGICRDVVMR--------------EITNSDARRSQTIDESLKDSLLLEELMHAVEE    359
tr|A0A0N0VHG5|A0A0N0VHG5_LEPPY    YLDTDLQQ------------------------------------ISTEFIIRELREGVSA    332
tr|A4HCJ2|A4HCJ2_LEIBR            YTDSLTGS------------------------------------SSSSFVVSELREGVSG    332
tr|A4I008|A4I008_LEIIN            YKDNITGT------------------------------------GSASFVVRELREGVSG    332
tr|Q4QBJ8|Q4QBJ8_LEIMA            YMDKITGT------------------------------------GSASFVVRELREGVSG    332
sp|Q944L7|SLP2_ARATH              GVRI-----------VTSNPLYKENLYSHV-APDSKT--GLGLLV--PVP---KQVEVKA    391
tr|A0A813HX77|A0A813HX77_POLGL    AYAMRAL-G---IDAIESAPE--------------Q------RLL--PIEGESGE-----    547
tr|A0A0S4JMN0|A0A0S4JMN0_BODSA    ATH-----TDI-VTPYIQYPSQPFR--QHA--------------L--PAVMFPKRIPVPP    334
tr|Q4CWC2|Q4CWC2_TRYCC            SDP---------SR-RIAVPQYPFG--RAV-RPPTS------PLL--KDPMRPPRRMPDN    342
tr|A0A1Y2EN62|A0A1Y2EN62_9FUNG    NEIWAVY-SDT-NRERID------------------------------------------    330
tr|A0A0C9MUE8|A0A0C9MUE8_9FUNG    --VNALY-ADK-RVKLASPPPYKS----RV---------------L--AS----KKHDHDE    315


tr|C5L0A1|C5L0A1_PERM5            FADSDEDE--------------------------L-------------------------    344
tr|A0A0S4JBT9|A0A0S4JBT9_BODSA    MKVKQQAEKAALASKEQQE----VVARDEL------------------------------    389
tr|Q583S8|Q583S8_TRYB2            YNQRQT-----------------TKGKNDVLDD-L-------------------------    374
tr|A0A1X0NU01|A0A1X0NU01_9TRYP    FRHRQN-----------------TRDQEETLGD-L-------------------------    374
tr|A0A3R7MVL3|A0A3R7MVL3_9TRYP    YYQSEN-----------------TVPTEDLVGD-L-------------------------    376
tr|Q4DFK9|Q4DFK9_TRYCC            FYQSPK-----------------DNPPEDTDYE-L-------------------------    376
tr|Q4DQ38|Q4DQ38_TRYCC            FYQSPK-----------------DNPPEDTDYE-L-------------------------    376
tr|A0A0N0VHG5|A0A0N0VHG5_LEPPY    FSMTSDGRNDGEQLSPKSS----FIV-DDDNGD-L-------------------------    361
tr|A4HCJ2|A4HCJ2_LEIBR            FCYTCVEARDDTDSPRGGG----SVE-KDTYDD-L-------------------------    361
tr|A4I008|A4I008_LEIIN            FCHTCVEERSGLDTTGGGD----SDEENDIFDD-L-------------------------    362
tr|Q4QBJ8|Q4QBJ8_LEIMA            FCHTCLDERSGLDTTVGGD----SD-ENDIFDD-L-------------------------    361
sp|Q944L7|SLP2_ARATH              ------------------------------------------------------------    391
tr|A0A813HX77|A0A813HX77_POLGL    -APSGTLGK--LASWLQGDWPAWTNVEL--------------------------------    572
tr|A0A0S4JMN0|A0A0S4JMN0_BODSA    FEAKEKGGV-----QGA-----ALTIRT--YEDSMTKSELVLLC-----IAFFFVAGLVA    377
tr|Q4CWC2|Q4CWC2_TRYCC            WQPSSVRGK-----EGEGKWRRAVKRRTKRGDDGLLHVSVQTLCFFISVLAFFLVCVVRL    397
tr|A0A1Y2EN62|A0A1Y2EN62_9FUNG    ------------------------------------------------------------    330
tr|A0A0C9MUE8|A0A0C9MUE8_9FUNG    L-----------------------------------------------------------    316


tr|C5L0A1|C5L0A1_PERM5            ----------------------------    344
tr|A0A0S4JBT9|A0A0S4JBT9_BODSA    ----------------------------    389
tr|Q583S8|Q583S8_TRYB2            ----------------------------    374
tr|A0A1X0NU01|A0A1X0NU01_9TRYP    ----------------------------    374
tr|A0A3R7MVL3|A0A3R7MVL3_9TRYP    ----------------------------    376
tr|Q4DFK9|Q4DFK9_TRYCC            ----------------------------    376
tr|Q4DQ38|Q4DQ38_TRYCC            ----------------------------    376
tr|A0A0N0VHG5|A0A0N0VHG5_LEPPY    ----------------------------    361
tr|A4HCJ2|A4HCJ2_LEIBR            ----------------------------    361
tr|A4I008|A4I008_LEIIN            ----------------------------    362
tr|Q4QBJ8|Q4QBJ8_LEIMA            ----------------------------    361
sp|Q944L7|SLP2_ARATH              ----------------------------    391
tr|A0A813HX77|A0A813HX77_POLGL    ----------------------------    572
tr|A0A0S4JMN0|A0A0S4JMN0_BODSA    SQRVKDGGKGLFSLSWLGGRRRKLEHAA    405
tr|Q4CWC2|Q4CWC2_TRYCC            RRR--RAANGRHRSWWKL----------    413
tr|A0A1Y2EN62|A0A1Y2EN62_9FUNG    ----------------------------    330
tr|A0A0C9MUE8|A0A0C9MUE8_9FUNG    ----------------------------    316
```

(overall sequence identity = 0.0442)

12. Calreticulin chaperones

Calreticulins are essential chaperones for secreted and transmembrane protein folding, and are found in an ER-resident, KDEL-motif restreined form in most eukaryotes. Kinetoplastids are no exception.

```
tr|S9W2Z8|S9W2Z8_9TRYP            ---MSRTTTFLVLLAALCALCVVQAEIYYHEKFDNLD----EWVTSSH-S---KYGKVRF    49
tr|A4HJP8|A4HJP8_LEIBR            --MTQRTMLAAVVGVLVLCVCLVQAEIFFHEEFNTLD----GWVQSEHKD---DYGKVEL    51
tr|A4I765|A4I765_LEIIN            --MAQRAMLAAVVGVLVLCVYVVQAEIFFHEEFNTMD----GWVQSEHTS---DYGKVAL    51
tr|Q4Q601|Q4Q601_LEIMA            --MAQRAMLAAIVGVLVLCVYVVQAEIFFHEEFNTMD----GWVQSEHTS---DYGKVAL    51
tr|A0A0S4KMN1|A0A0S4KMN1_BODSA    ----MSRVIALVAA-AALLCAAAQAKVYFHEEFNSLD----GWVVSEAKE---GLGKAVL    48
tr|A0A1X0NIG9|A0A1X0NIG9_9TRYP    ----MRAIVLLCALLGLSMLVSVNGKILFHEDFSSLD----KWVQSKERD---DYGKVEL    49
tr|F9WHI5|F9WHI5_TRYCI            ----MRRGTIVCALLGLVAAATVQGTIYFHEKFTSIN----HWTPSKARS---DYGKVEL    49
tr|Q57YU3|Q57YU3_TRYB2            MLMCMRPVAVACVFVALATVATVHGAIHFHEKFSSID----HWTASKARS---DYGKVEL    53
tr|Q584K4|Q584K4_TRYB2            MLMCMRPVAVACVFVALATVATVHGAIHFHEKFSSID----HWTASKARS---DYGKVEL    53
tr|Q4CPZ0|Q4CPZ0_TRYCC            ----MRAAIFFCALLGLATLSAVHGTVYFHEEFKSME----HWTTSKHRD---DFGKVEI    49
tr|Q4DDX3|Q4DDX3_TRYCC            ----MRAAIFFCALLSLATLSAVHGTVYFHEEFKSME----HWTTSKHRD---DFGKVEI    49
tr|A0A3R7NR70|A0A3R7NR70_9TRYP    ----MRAALLVCALLGLAALSAVEATVYFHEEFSSLE----RWTPSKHRD---DLGKVEL    49
tr|A0A3R7KPX3|A0A3R7KPX3_TRYRA    ----MPAAILVCALLALATLAAVDATVYFHEEFKSLD----HWTPSKYRD---DLGKVEL    49
tr|A0A367JDX0|A0A367JDX0_RHIAZ    ----MKIPTI---TAILGLAALASAEVFFHETFSDGEGWKDRWTPSTYRE---DLGKLEV    50
tr|S2IZR0|S2IZR0_MUCC1            ----MKIPTI---AAVLGLATLVSAEVFLHETFSDGEGWKDRWTASEHRE---DLGKLEV    50
sp|O04151|CALR1_ARATH             MAKLNPKFIS---LILFALVVIVSAEVIFEEKFE--DGWEKRWVKSDWKKDDNTAGEWKH    55
sp|Q9ZNY3|CALR_EUGGR              --M--RKELW---LGLLLSSQAVLSTIYYKETFE--PDWETRWTHSTAKS---DYGKFKL    48
tr|Q6PE26|Q6PE26_DANRE            MRI--TAAVC---FISALA-FIAHADVYFKEQFLDGDGWKSRWVESKHKS---DYGQWKL    51
sp|P27797|CALR_HUMAN              MLL--SVPLL---LGLLGL-AVAEPAVYFKEQFLDGDGWTSRWIESKHKS---DFGKFVL    51
                                                            .   :  .* *      *  *   .         *:

tr|S9W2Z8|S9W2Z8_9TRYP            SSGEKPVDR-EMQRGLQLAEESSFYAVSKKLPTRITNDGKPLVVSFQLLHRGRFTCGGGY    108
tr|A4HJP8|A4HJP8_LEIBR            SAGALHVDA-AKEQGMKLTEDSKFYAISKELPTPVSNDGKPLVISFSVKNEQNLKCGGAY    110
tr|A4I765|A4I765_LEIIN            SVGAIHVDA-EKEQGLKLMEDAKFYAVSKKLPKAVSNDGKSIVVSFSVKNEQKLTCGGTY    110
tr|Q4Q601|Q4Q601_LEIMA            SVGAIHVDA-EKEQGLKLMEDAKFYAVSKKLPKAVSNDGKPIVVSFSVKNEQKLTCGGTY    110
tr|A0A0S4KMN1|A0A0S4KMN1_BODSA    SSGKFFGDE-KINQGLKTSEDAHFYAIAKTLPEAVTNEGKDLVVSISVKHEQGLDCGGGY    107
tr|A0A1X0NIG9|A0A1X0NIG9_9TRYP    SAGKNEPEGSSNKKGLRLTEDARFYAISTEFPTPISNENKELVFSFVVKHEQDLKCGGAY    109
tr|F9WHI5|F9WHI5_TRYCI            SAGKFFADA-EKSKGLRLTEDARFYALSTPLPTPISNEKKDFVVSFSVKHEQGLRCGGGY    108
tr|Q57YU3|Q57YU3_TRYB2            SAGKFYADA-EKSKGLRLTEDARFYALSTPLPTPITNEKKDFVVSFSVKHEQDLRCGGGY    112
tr|Q584K4|Q584K4_TRYB2            SAGKFYADA-EKSKGLRLTEDARFYALSTPLPTPITNEKKDFVVSFSVKHEQDLRCGGGY    112
tr|Q4CPZ0|Q4CPZ0_TRYCC            SAGKFYADA-EKSKGLRLTEDARFYALSTAFPTPINNEKKSLVVSFSVKHEQDLKCGGGY    108
tr|Q4DDX3|Q4DDX3_TRYCC            SAGKFYADA-EKSKGLRLTEDARFYALSTAFPTPITNEKKSLVVSFSVKHEQDLKCGGGY    108
tr|A0A3R7NR70|A0A3R7NR70_9TRYP    SAGDFYADA-EKSKGLRLTEDARFYAVSTALPTPITNDGKDLVVSFSVKHGQELKCGGGY    108
tr|A0A3R7KPX3|A0A3R7KPX3_TRYRA    SAGDFYADA-EKSKGLRLMDDARFYAFSTALPTPITNDGKDFVVSFSVKHGQDLKCGGGY    108
tr|A0A367JDX0|A0A367JDX0_RHIAZ    SPGKWYVDE-KANAGLRTTEDYRFYAVSSK-TKPFTNKDKNLVIQFDVKNEQDIDCGGSY    108
tr|S2IZR0|S2IZR0_MUCC1            SPGKWFADE-AYNAGLRTTEDYRFYATSTKIPKPFSNKDKDLVIQFDVKNEQDIDCGGSY    109
sp|O04151|CALR1_ARATH             TAGNWSGDA--NDKGIQTSEDYRFYAISAEF-PEFSNKDKTLVFQFSVKHEQKLDCGGGY    112
sp|Q9ZNY3|CALR_EUGGR              TSGKFYGDK-AKDAGIQTSQDAKFYAISSPIASSFSNEGKDLVLQFSVKHEQDIDCGGGY    107
tr|Q6PE26|Q6PE26_DANRE            TSGKFYGDA-ELDKGLQTSQDARFYALSSRF-DSFSNEGKTLVIQFTVKHEQKIDCGGGY    109
sp|P27797|CALR_HUMAN              SSGKFYGDE-EKDKGLQTSQDARFYALSASF-EPFSNKGQTLVVQFTVKHEQNIDCGGGY    109
                                  : *      :       . *::  ::  *** :          ..*. : :*..: : :   : *** *

tr|S9W2Z8|S9W2Z8_9TRYP            IKLLP-AMDQKDFHGETEYGLMFGPDICGG-ERRVHIIFNYKGQNYLWNKRELFV---VD    163
tr|A4HJP8|A4HJP8_LEIBR            LKFFS-ELNQKDLHSESPYWLMFGPDVCGF-QNRLHFIFNYNDENHLWRSFWRLTKELNE    168
tr|A4I765|A4I765_LEIIN            LKFFS-ELDQKDLHGESAYWLMFGPDTCGS-STRLQFILSYNGTNHLWKKLWRPK---TD    165
tr|Q4Q601|Q4Q601_LEIMA            LKFFS-ELDQKDLHGESAYWLMFGPDICGS-NKRLQFILSYNGTNHLWKKLSTPK---TD    165
tr|A0A0S4KMN1|A0A0S4KMN1_BODSA    AKFLP-ATELKGFNGDTEYLLMFGPDQCGY-NKRVHVIFNYKGTNLLWKKEPKYP---DD    162
tr|A0A1X0NIG9|A0A1X0NIG9_9TRYP    LKYLP-AMDPTKFNGESKYWLMFGPDRCGS-KNKVHIILHYNGVNHEWSKNFRYP---ED    164
tr|F9WHI5|F9WHI5_TRYCI            IKLMP-NMDPAEFKGETKYWLMFGPDRCGY-NNKIHIIINYNNTNMEWKKHPRYP---DD    163
tr|Q57YU3|Q57YU3_TRYB2            IKLLP-QMDPAELKGETKYWLMFGPDRCGY-DKKIHIIISYNGANREWKKRPSYP---DD    167
tr|Q584K4|Q584K4_TRYB2            IKLLP-QMDPAELKGETKYWLMFGPDRCGY-DKKIHIIISYNGANREWKKRPSYP---DD    167
tr|Q4CPZ0|Q4CPZ0_TRYCC            IKLLP-SMDPEKFHGETKYWLMFGPDRCGS-QNRVHIILHYNGENREWSKRIRFP---ED    163
tr|Q4DDX3|Q4DDX3_TRYCC            IKLLP-SMDPEKFHGETKYWLMFGPDRCGS-QNRVHIILHYNGENREWSKRIRFP---ED    163
tr|A0A3R7NR70|A0A3R7NR70_9TRYP    IKLLP-AMDAEKFNGETKYWLMFGPDRCGA-KSKVQIILHRNGENHEWSKEVRFP---VD    163
tr|A0A3R7KPX3|A0A3R7KPX3_TRYRA    IKLLP-AMDAEKFNGETKYWLMFGPDRCGA-KNKVHIILHRNGENHEWSKNIHFP---DD    163
tr|A0A367JDX0|A0A367JDX0_RHIAZ    LKFFGGEFDPKTFNGDTSYNIMFGPDICGP-KSIVHAIFNYNGTNYDFKKTISAP---KD    164
tr|S2IZR0|S2IZR0_MUCC1            LKIFG-DLDPKAFNGDSEYNIMFGPDICGP-KAMVHAIFNYNGTNHDLKKSISAP---KD    164
sp|O04151|CALR1_ARATH             MKLLSDDVDQTKFGGDTPYSIMFGPDICGYSTKKVHAILTYNGTNHLIKKEVPCE---TD    169
sp|Q9ZNY3|CALR_EUGGR              LKLLPS-VDAAKFTGDTPYHIMFGPDICGA-TKKIHFILTYKGKNLLWKKEPRCE---TD    162
tr|Q6PE26|Q6PE26_DANRE            VKVFPAEMDQTEMHGESQYYIMFGPDICGYSTKKVHVIFNYKGQNHLIKKDIKCK---DD    166
sp|P27797|CALR_HUMAN              VKLFPNSLDQTDMHGDSEYNIMFGPDICGPGTKKVHVIFNYKGKNVLINKDIRCK---DD    166
                                  * :      :   : .:: * :***** **     :: *:  :. *      .       :

tr|S9W2Z8|S9W2Z8_9TRYP            ELTHAYTLAIYPNNTYALYLDANLTVSGSIAEEWSMLPPEMIDDPSDKKPAHWDDEPAMI    223
tr|A4HJP8|A4HJP8_LEIBR            KATHVYTVQISPNNTYQLYVDGKYIQEGSLVDEWEMLPPKTIADPEEKKPSNWIEDS-MM    227
tr|A4I765|A4I765_LEIIN            KATHVYTVEIAPNNTYQLYVDGMHIQEGSFEEEWDMLPPKTIPDPTDEKPADWVDDM-MM    224
tr|Q4Q601|Q4Q601_LEIMA            TATHVYTVEIAPNNTYQLYVDGMHIQEGSLEEEWDMLPPKTISDPTDKKPADWVDDM-MM    224
tr|A0A0S4KMN1|A0A0S4KMN1_BODSA    KLTHVYTLRVTPENKYELYIDQELKESGALEADWEFLQPKEIDDATDSKPADWVDAS-QI    221
tr|A0A1X0NIG9|A0A1X0NIG9_9TRYP    SITHAYTLRIAPDNSYSFYLDGQERMKGTLEEDWSLLPAKEIADPSDVKPADWVDEP-TI    223
tr|F9WHI5|F9WHI5_TRYCI            KLTHAYTLHIAPDNSYKFYLDGSPTEKGNLEDDWDFLPPKEIEDPEDKKPADWVDAK-TI    222
tr|Q57YU3|Q57YU3_TRYB2            KLTHVYTLHITPSNSYEFFLDGVSKEKGTLEADWDFLPEKEIDDPEDKKPADWVDVP-TI    226
tr|Q584K4|Q584K4_TRYB2            RLTHVYTLHITPSNSYEFFLDGVSKEKGTLEADWDFLPEKEIDDPEDKKPADWVDVP-TI    226
tr|Q4CPZ0|Q4CPZ0_TRYCC            KLTHVYTLHIAADNSYEFFLDGESKAKGQLEEDWSLLLPREIVDETDKKPEDWVDEE-TM    222
tr|Q4DDX3|Q4DDX3_TRYCC            KLTHVYTLHIAADNSYEFFLDGESKAKGQLDGDWTLLPPREIVDETDKKPEDWVDEE-TM    222
tr|A0A3R7NR70|A0A3R7NR70_9TRYP    KLTHVYTLHIAADNTYEFFLDGELRAKGELEADWHLLPPREIVDPDDTKPADWVDAA-TI    222
tr|A0A3R7KPX3|A0A3R7KPX3_TRYRA    KLTHVYTLRIATDNTYEFFLDGESKAKGALEDDWSLLPPREITDETDKKPDDWVDEK-TM    222
tr|A0A367JDX0|A0A367JDX0_RHIAZ    TLTHTYTLIVKPDQTYEILVDSDKKASGSLLEDWDFLPPKQIKDPNAVKPADWVDDA-LI    223
tr|S2IZR0|S2IZR0_MUCC1            TLTHTYTLVVKPDQTYEILIDGKSKADGSLLEDWDFLPPKTIKDPNASKPEDWVEEA-MI    223
sp|O04151|CALR1_ARATH             QLTHVYTFVLRPDATYSILIDNVEKQTGSLYSDWDLLPAKKIKDPSAKKPEDWDDKE-YI    228
sp|Q9ZNY3|CALR_EUGGR              TLSHTYTAVIKADRTYEVLVDQVKKESGTLEEDWEILKPKTIPDPEDKKPADWVDEP-DM    221
tr|Q6PE26|Q6PE26_DANRE            ELTHLYTLILRPDQTYEVKIDNEKVESGSLEEDWDFLPPKKIKDPEAKKPDDWDDRA-KI    225
```

```
sp|P27797|CALR_HUMAN              EFTHLYTLIVRPDNTYEVKIDNSQVESGSLEDDWDFLPPKKIKDPDASKPEDWDERA-KI    225
                                  :* **  :  . .* . :*       * :  :* :*  . * *** .* :  :

tr|S9W2Z8|S9W2Z8_9TRYP            PDPDDTKPEDWDKEPATILDPDATKPNDWNDAEDGEWEPPIITNPNYKGDWFPRKIKNPK    283
tr|A4HJP8|A4HJP8_LEIBR            DDPYDTKPEDWDDELPTIADDTAVKPDDWVDEEDGEWEAPRIPNPKYRGAWVPRRIDNPN    287
tr|A4I765|A4I765_LEIIN            DDPSDTKPEHWDDEPATITDSEAVKPVDWDDAEDGVWEAPKIPNPNYRGAWTPRRIHNPD    284
tr|Q4Q601|Q4Q601_LEIMA            DDPSDTKPEDWEDEPATITDSEAVKPVDWDDSEDGVWEAPKVPNPNYRGAWKPRRIHNPD    284
tr|A0A0S4KMN1|A0A0S4KMN1_BODSA    DDPEDVKPADWESEPATVVDPEAKKPDDWDDEEDGAWEAPQIPNPKAKGEWRARRIPNPA    281
tr|A0A1X0NIG9|A0A1X0NIG9_9TRYP    VDPNDVKPADWDNEPAMIPDTEAKRPDDWDDAEDGEWEAPMVPNPNAKGEWHPKRIPNPA    283
tr|F9WHI5|F9WHI5_TRYCI            DDPEDKKPDDWDNEPETIVDPDAKKPDDWDNDAEDGTWEAPMIPNPKSKGPWSPRKITNPA    282
tr|Q57YU3|Q57YU3_TRYB2            DDPEDKKPEDWDSEPEKIVDPEAKKPEDWNDAEDGAWEAPMIPNPKSKGPWAPRKIPNPA    286
tr|Q584K4|Q584K4_TRYB2            DDPEDKKPEDWDSEPEKIVDPEAKKPEDWNDAEDGAWEAPMISNPKSKGPWAPRKIPNPA    286
tr|Q4CPZ0|Q4CPZ0_TRYCC            DDPEDKKPEDWDNEPAMIPDADAKKPDDWDDAEDGPWEAPMIPNPKSKGTWKPRQIPNPA    282
tr|Q4DDX3|Q4DDX3_TRYCC            DDPEDKKPEDWDNEPAMIPDADAKKPDDWDDAEDGPWEAPMIPNPKSKGTWKPRQIPNPA    282
tr|A0A3R7NR70|A0A3R7NR70_9TRYP    DDPADTKPADWDDEPAMIPDAAAKKPDDWDDAEDGEWEAPMVPNPKAKGAWTPRKIPNPA    282
tr|A0A3R7KPX3|A0A3R7KPX3_TRYRA    DDPEDKKPADWDEEPEMIPDEEAKKPEDWDDAEDGAWEAPMIPNPKAKGAWKPRQIPNPA    282
tr|A0A367JDX0|A0A367JDX0_RHIAZ    DDPNDVKPEGYDDIPEFIPDPNAKKPEDWDDDMDGEWEAPSIANPDYKGPWTPKKIPNPA    283
tr|S2IZR0|S2IZR0_MUCC1            VDETDVKPANYDDIPEFIPDPEAKKPEDWDDDMDGEWEAPSIANPEYQGEWSPKKIPNPL    283
sp|O04151|CALR1_ARATH             PDPEDTKPAGYDDIPKEIPDTDAKKPEDWDDEEDGEWTAPTIPNPEYNGEWKPKKIKNPA    288
sp|Q9ZNY3|CALR_EUGGR              VDPEDKKPEDWDKEPAQIPDPDATQPDDWDEEEDGKWEAPMISNPKYKGEWKAKKIPNPA    281
tr|Q6PE26|Q6PE26_DANRE            DDPEDTKPEDWDK-PENIPDPDAKKPDDWDEDMDGEWEPAMIPNPEYKGEWKPKQIDNPS    284
sp|P27797|CALR_HUMAN              DDPTDSKPEDWDK-PEHIPDPDAKKPEDWDEEMDGEWEPPVIQNPEYKGEWKPRQIDNPD    284
                                  *  * **  ::.     : *  * :* ** :  ** *      : **. .* *  ::* **

tr|S9W2Z8|S9W2Z8_9TRYP            YKGQWKPRQIKNPNFYPDPDLYRMKKPLEYVGIDVWTVEAGSIYTHIMIGDNVEEVLDVL    343
tr|A4HJP8|A4HJP8_LEIBR            YKGVWSPQQIPNPDYKEDPNLYKSPAPLKYVGIDVWQVESGSIFDNIIIGDDLQEVLKVV    347
tr|A4I765|A4I765_LEIIN            YKGKWAARQIPNPAYKEDPNLYRAPAPLQYVGIDVWQVEGGSIFDDIIIGDDITEVLGVV    344
tr|Q4Q601|Q4Q601_LEIMA            YKGEWAARQIPNPVYKKDPNLYKVPAPLQYVGIDVWQVEGGSIFDDIIIGDDITEVLRVV    344
tr|A0A0S4KMN1|A0A0S4KMN1_BODSA    YKGPWKARQVANPKYEEDAKLYLLRKPLAAVGIDVWQVKAGSIFDNIIVGDDLAEVNKLI    341
tr|A0A1X0NIG9|A0A1X0NIG9_9TRYP    YKGEWAPRMIPNPEYKEDKELYKVREPLAHVGIDVWQVESGSIFRDIIIGDDVNEVLEMV    343
tr|F9WHI5|F9WHI5_TRYCI            YKGPWAPRKIANPAYKSDPNLYMIPEPLTHVGIDVWQVESGSIYKDIVVGDDLKEVLDIV    342
tr|Q57YU3|Q57YU3_TRYB2            YKGPWAPRRIPNPAYKNDEELYKIPEPLTHVGIDVWQVESGSIFKDIIIGDDVKEVLDIV    346
tr|Q584K4|Q584K4_TRYB2            YKGPWAPRRIPNPAYKNDEELYKIPEPLTHVGIDVWQVESGSIFKDIIIGDDVKEVLDIV    346
tr|Q4CPZ0|Q4CPZ0_TRYCC            YKGVWEPRKIPNPDFVEDSELHKVPEPLTHVGIDVWQVESGSIFKDIVIGDDLKEVLDLV    342
tr|Q4DDX3|Q4DDX3_TRYCC            YKGVWEPRKIPNPDFVEDSELHKVPEPLTHVGIDVWQVESGSIFKDIMIGDDLKEVLELV    342
tr|A0A3R7NR70|A0A3R7NR70_9TRYP    YKGAWEPRKIPNPAYKPDDSLYKIPEPLAHVGIELWQVESGSIFKDIVLGDDVAEVLDLV    342
tr|A0A3R7KPX3|A0A3R7KPX3_TRYRA    YKGEWKPRVIPNPEYKPDDGLYKIPEPLAHVGIDIWQVESGSIFKNIVLGDDVAEVLELV    342
tr|A0A367JDX0|A0A367JDX0_RHIAZ    YKGEWEHPLIDNPDYKVDNEIYAYD--FANVGIDVWQVKSGTIFDNILITDDIEEAEKVR    341
tr|S2IZR0|S2IZR0_MUCC1            YKGEWVHPEIDNPEYKVDNEIYAYD--FANVGIDVWQVKSGTIFDNILITDDVEEAKKIR    341
sp|O04151|CALR1_ARATH             YKGKWKAPMIDNPEFKDDPELYVFP-KLKYVGVELWQVKSGSLFDNVLVSDDPEYAKKLA    347
sp|Q9ZNY3|CALR_EUGGR              YKGVWKPRDIPNPEYEADDKVHIFD-EIAAVGFDLWQVKSGTIFDNIIVTDSLAEAKAFY    340
tr|Q6PE26|Q6PE26_DANRE            YKGTWVHPEIDNPEYAADDAIYKFD-SIGVLGLDLWQVKSGTIFDNFLITDDVEEAEKFG    343
sp|P27797|CALR_HUMAN              YKGTWIHPEIDNPEYSPDPSIYAYD-NFGVLGLDLWQVKSGTIFDNFLITNDEAYAEEFG    343
                                  *** *    : ** :  *  ::    :  :*.::* *:.*::: ..:: :.   .  .

tr|S9W2Z8|S9W2Z8_9TRYP            KTTFVNINKLEARLEKAEREKMEREREEKALEEAEKDETAA-------------------    384
tr|A4HJP8|A4HJP8_LEIBR            ESTYGAMAEDEMNLIEAEAEERQRKERKQE---EENSLTEQEKENSV-----------TE    393
tr|A4I765|A4I765_LEIIN            KSTYGAMAEKERDLIQAEEKKEATKEPAEA---AAEK-------PNV-----------GE    383
tr|Q4Q601|Q4Q601_LEIMA            KSTYGAMAEKERDLIQAEAKKEAMKEPAEA---AAEK-------PNM-----------DE    383
tr|A0A0S4KMN1|A0A0S4KMN1_BODSA    DATWGATKDAEKAAEDAAAAATKAADE--AAKADESKEE---------------------    378
tr|A0A1X0NIG9|A0A1X0NIG9_9TRYP    KKTVEEERKKEEAEKE---KKEADAKKE-----EEKKEGEKK------------------    377
tr|F9WHI5|F9WHI5_TRYCI            QSTYGDMKKAEEEAFDAFEKKEEEERKSKEAKEEEKKEEEKKEEEKK-----------E-    390
tr|Q57YU3|Q57YU3_TRYB2            KSTYDGMKKAEEDALAAFEKKKEQDKKEENKKETDGEEDKKKK-----------------    389
tr|Q584K4|Q584K4_TRYB2            KSTYDGMKKAEEDALAAFEKKEEQDEKEEDKKETDGEEDKKKK-----------------    389
tr|Q4CPZ0|Q4CPZ0_TRYCC            EKTYGGLKKAEADALKVMEDMEKEKRKKEEEEEREKEKAEE-------------------    383
tr|Q4DDX3|Q4DDX3_TRYCC            EKTYGSLKKAEADALKVMEDMEKEKRKKEEEEEKEKEKAEE-------------------    383
tr|A0A3R7NR70|A0A3R7NR70_9TRYP    KKTYGSLKQAEAEALNAFEGKQKEAEK--QKEAEEQKEAEAA---EN-------------    384
tr|A0A3R7KPX3|A0A3R7KPX3_TRYRA    KETYGSLKQAEAEALKAFETKQEAKKAEEAKEAEEAKEAEDEKEAED-----------EK    391
tr|A0A367JDX0|A0A367JDX0_RHIAZ    NETKDLHEKEEDAKKAHEEKVDAENKAKAEEAEKDAK-----------------------    378
tr|S2IZR0|S2IZR0_MUCC1            EETQVLRSPEEGAQAAYNEKIQAEAKAKAEAEGAAPPDEGAEKIDLEKFEPAVKFDEVPP    401
sp|O04151|CALR1_ARATH             EETWGKHKDAEKAAFD---EAEKKRE---EEESKDAPAESDAEEEAE--------DDDNE    393
sp|Q9ZNY3|CALR_EUGGR              DQTNGATKDAEKKAFD---SAEADKRKKEEDERKKQEEEEKKTAE-E--------DEDDD    388
tr|Q6PE26|Q6PE26_DANRE            TDTWGATKGPEKKMKD---QQEEEERKKREEEEKSKKDDNEEDEEDE--------DEDEP    392
sp|P27797|CALR_HUMAN              NETWGVTKAAEKQMKD---KQDEEQRLKEEEEDKKRKEEEEAEDKED--------DEDKD    392
                                  *        *

tr|S9W2Z8|S9W2Z8_9TRYP            --------------------AAEDDEDLGDE-DEENTDL   402
tr|A4HJP8|A4HJP8_LEIBR            HVEEETE---------APVALNGANDNEGNQ-DSNNEDL   422
tr|A4I765|A4I765_LEIIN            HADHTP--------------------DEGD--SEDKEDL   400
tr|Q4Q601|Q4Q601_LEIMA            HADHTP--------------------DEDK--SEGEEDL   400
tr|A0A0S4KMN1|A0A0S4KMN1_BODSA    --------------------------------------L   379
tr|A0A1X0NIG9|A0A1X0NIG9_9TRYP    -----------------------------EA-DEDKGDL   386
tr|F9WHI5|F9WHI5_TRYCI            ------------------EEKKEEEKKEEGK-KEEKADL   410
tr|Q57YU3|Q57YU3_TRYB2            ---------------------------------EDKSDL   395
tr|Q584K4|Q584K4_TRYB2            ---------------------------------EDKSDL   395
tr|Q4CPZ0|Q4CPZ0_TRYCC            --------------------EKDEEELEEKG-DGDKEDL   401
tr|Q4DDX3|Q4DDX3_TRYCC            --------------------EKDEEELEEKG-DGDKEDL   401
tr|A0A3R7NR70|A0A3R7NR70_9TRYP    --EVEAE---------AAAMEDEEAEKKADA-DGDKEDL   411
tr|A0A3R7KPX3|A0A3R7KPX3_TRYRA    EAEDEKE---------SEEVKESEEAKDAGA-DGDKEDL   420
tr|A0A367JDX0|A0A367JDX0_RHIAZ    -KDQEPETPKEEEILEALEDAVEEAVEADAAKKPARDEL   416
tr|S2IZR0|S2IZR0_MUCC1            AAAEALEKAKEEEILEAIKEEAEKKVEEEDAKKPVKDEL   440
sp|O04151|CALR1_ARATH             -GDDSDNESKSEETKEA------EETKEAEETDAAHDEL   425
sp|Q9ZNY3|CALR_EUGGR              --DEEEEED-------D-----------------KKDEL   401
tr|Q6PE26|Q6PE26_DANRE            EEDDHTEEP-------P------EEEEEGEDDALPKDEL   418
sp|P27797|CALR_HUMAN              E-DEEDEED-------K------EEDEEEDVPGQAKDEL   417
                                                                         *
```

(overall sequence identity = 0.1594)

13. Endoplasmin-like HSP90 chaperones

Endoplasmins represent a nearly ubiquitous ER-resident clade of the greater HSP90 family. These essential chaperones contain KDEL-like motifs in most eukaryotic lineages examined.

```
tr|A0A0L1KBS8|A0A0L1KBS8_9EUGL    ---------------------------------MRFWSIALVLA-IASVSSSPT------    20
tr|A0A0L1KHT0|A0A0L1KHT0_9EUGL    ---------------------------------------MSRISVYSLLVFGILLLSG--    19
tr|A0A1X0P079|A0A1X0P079_9TRYP    ---------------------------------MSKRTLFLQGLLIVLVVLGVTAVED--    25
tr|Q4DW89|Q4DW89_TRYCC            ----------------------------------MARHPIIQAILIALIVLGVAVTGV--    24
tr|A0A422PWD8|A0A422PWD8_9TRYP    -------------------------------MLVRRW--ILKALLIAVVALSAVASEE--    25
tr|A0A422NRM6|A0A422NRM6_TRYRA    ---------------------------------MRGWHAILQILLVAVVALSVVATED--    25
tr|A0A0S4KLS2|A0A0S4KLS2_BODSA    ----------------------------------MRLSLIFRIACVALLAFA---IV---    20
tr|A0A0N0VF86|A0A0N0VF86_LEPPY    ----------------------------------MRRSPLLRVALVALIFLGTISAT---    23
tr|A4HH83|A4HH83_LEIBR            ----------------------------------MANSNVHRVVLVALLLLGSVT-V---    22
tr|E9ADS8|E9ADS8_LEIMA            ----------------------------------MANSSLLRVVLVALLLLGSVT-V---    22
sp|Q9NGD0|ENPL_LEIIN              ----------------------------------MANSSLLRVVLVALLLLGSVT-V---    22
tr|A4I4C9|A4I4C9_LEIIN            ----------------------------------MANSSLLRVVLVALLLLGSVT-V---    22
sp|Q8T7E0|ENPL_LEIDO              ----------------------------------MANSSLLRVVLVALLLLGSVT-V---    22
tr|S9U4L8|S9U4L8_9TRYP            -----------------------------MARLNKTVKVVLQIALVVCLLLGATVAK---    28
tr|A0A7G2C3S2|A0A7G2C3S2_9TRYP    MAYSSSLSLFCSCSLPIIIIIAYYYNDWLTHPMGKVTQVVLKVVLIALLLVGATIAK---    57
sp|P14625|ENPL_HUMAN              ----------------------------------MRALWVL-GLCCVLLTFGSVRA----    21
tr|Q7T3L3|Q7T3L3_DANRE            ----------------------------------MRRLWII-GLLCALLAFASVKA----    21
sp|Q9STX5|ENPL_ARATH              ------------------------MRKRTLVSVLFL-FSLL-FLL----PDQGRKLHANA    30
tr|A0A061FKM2|A0A061FKM2_THECC    ------------------------MRKWAIPSALILLCL-L-SLL----SDQGRKVHANA    30
tr|Q5Z9N8|Q5Z9N8_ORYSJ            ------------------------MRKWALSSALLLLLLLL-TTL----PDPAKKLQVNA    31
tr|W2ZJ09|W2ZJ09_PHYPR            ------------------------MKGG-CRSLLLLLLMAV-TLCYSSLSSSSAR--AEV    32
tr|W2ZJ74|W2ZJ74_PHYPR            ------------------------MKGG-CRSLLLLLLMAV-TLCYSSLSSSSAR--AEV    32
tr|F0Y644|F0Y644_AURAN            --------------------------------MKLSILKLV-A----------LVFGAVA    17
tr|D8LHY7|D8LHY7_ECTSI            ----------------------------------MRRPRFV-ALCAAAALQSSTPWGGSA    25


tr|A0A0L1KBS8|A0A0L1KBS8_9EUGL    ----------------M--------------------------------AVEENVAESGG    32
tr|A0A0L1KHT0|A0A0L1KHT0_9EUGL    --------------GVE--------------------------------KTFLGAEKGET    33
tr|A0A1X0P079|A0A1X0P079_9TRYP    --------------AAK--------------------------------SDGGAQDKGKP    39
tr|Q4DW89|Q4DW89_TRYCC            --------------TVK--------------------------------DD-GSVEKGRP    37
tr|A0A422PWD8|A0A422PWD8_9TRYP    --------------AAQ--------------------------------TD-ATVEKGKP    38
tr|A0A422NRM6|A0A422NRM6_TRYRA    --------------AVQ--------------------------------SD-VVVEKGKP    38
tr|A0A0S4KLS2|A0A0S4KLS2_BODSA    -------------------------------------------------GEESASGKGSP    31
tr|A0A0N0VF86|A0A0N0VF86_LEPPY    ---------------------------------------------------SAGDGRGTP    32
tr|A4HH83|A4HH83_LEIBR            ---------------------------------------------------SAGDARGSP    31
tr|E9ADS8|E9ADS8_LEIMA            ---------------------------------------------------SAGDGRGTP    31
sp|Q9NGD0|ENPL_LEIIN              ---------------------------------------------------SAGDGRGTP    31
tr|A4I4C9|A4I4C9_LEIIN            ---------------------------------------------------SAGDGRGTP    31
sp|Q8T7E0|ENPL_LEIDO              ---------------------------------------------------SAGDGRGTP    31
tr|S9U4L8|S9U4L8_9TRYP            -------------------------------------------------ETTEQVAKGEP    39
tr|A0A7G2C3S2|A0A7G2C3S2_9TRYP    -------------------------------------------------ENTEAIQKGSP    68
sp|P14625|ENPL_HUMAN              ------DDEVDVDGTVEEDLGKSREGSRTDDEVVQREEEAIQLDGLNASQIRELREKSEK    75
tr|Q7T3L3|Q7T3L3_DANRE            ------DDDVDIDGTVEEDLGKSRDGSRTDDEVVQREEEAIQLDGLNTSQLKEIRDKAEK    75
sp|Q9STX5|ENPL_ARATH              EES---SDDVTDPPKVEEKIGGH-GGLSTDSDVVHRESESMS--------KKTLRSNAEK    78
tr|A0A061FKM2|A0A061FKM2_THECC    D------EGAVDPPKVEEKIGAVPNGLQTDSDVVKRESESIS--------SRSLRSNAEK    76
tr|Q5Z9N8|Q5Z9N8_ORYSJ            DDS---TDELVDPPKVEEKIGGVPHGLSTDSEVVQREAESIS--------RKTLRSSAEK    80
tr|W2ZJ09|W2ZJ09_PHYPR            STGSDEVLGRST-PS-----------L----------LVDDL--------NDEPLTGGET    62
tr|W2ZJ74|W2ZJ74_PHYPR            STGSDEVLG-ST-PS-----------L----------LVDDL--------NDEPLTGGET    61
tr|F0Y644|F0Y644_AURAN            --------------S-----------L----------GTVAY--------AEDAGAAPET    34
tr|D8LHY7|D8LHY7_ECTSI            SSGLARAEEAVAPAS-----------T----------MDAAF--------EEQLKDGAEA    56


tr|A0A0L1KBS8|A0A0L1KBS8_9EUGL    HSYQAEVSKMLEILIHSLYTNRSIFLREIISNASDALDKIKYMYLTNPKSPKNKDGEEPT    92
tr|A0A0L1KHT0|A0A0L1KHT0_9EUGL    HKYKAEVGKMLDILINSLYTNHSIFLREIISNASDALDKVRFLYLTKPKNPKNDAGKEPT    93
tr|A0A1X0P079|A0A1X0P079_9TRYP    VAFQAEVSKMLDILINSLYTNRAVFLRELISNGSDALDKIRMLYLTAPKEPVNKDGVAPT    99
tr|Q4DW89|Q4DW89_TRYCC            ISFQAEVSKMLDILINSLYTNRAVFLRELISNGSDALDKIRMLYLTAPKEPKNKDGEVPA    97
tr|A0A422PWD8|A0A422PWD8_9TRYP    ISFQAEVSKMLDILINSLYTNRAVFLRELISNGSDALDKIRMLYLTAPKEPKNKDGEVPA    98
tr|A0A422NRM6|A0A422NRM6_TRYRA    ISFQAEVSKMLDILINSLYTNRAVFLRELISNGSDALDKIRMLYLTAPKEPKNKDGEVPA    98
tr|A0A0S4KLS2|A0A0S4KLS2_BODSA    ITFQAEVSKMLDILINSLYTNRNIFLREIISNASDALDKIRFFYLTTPREPTNANGEAPT    91
tr|A0A0N0VF86|A0A0N0VF86_LEPPY    ITFQAEVSKMLDILVNSLYTNRAIFLRELISNGSDALDKIRVLYLTAPKEPLNADGVSPT    92
tr|A4HH83|A4HH83_LEIBR            ITFQAEVSKMLDILVNSLYTNHAVFLRELISNGSDALDKIRVLYLTSPKEPLTKDGETPT    91
tr|E9ADS8|E9ADS8_LEIMA            IAFQAEVSKMLDILVNSLYTNRAVFLRELISNGSDALDKIRVLYLTSPKEPLTKDGEAPT    91
sp|Q9NGD0|ENPL_LEIIN              IAFQAEVSKMLDILVNSLYTNRAVFLRELISNGSDALDKIRVLYLTSPKEPLTKDGEAPT    91
tr|A4I4C9|A4I4C9_LEIIN            IAFQAEVSKMLDILVNSLYTNRAVFLRELISNGSDALDKIRVLYLTSPKEPLTKDGEAPT    91
sp|Q8T7E0|ENPL_LEIDO              IAFQAEVSKMLDILVNSLYTNRAVFLRELISNGSDALDKIRVLYLTSPKEPLTKDGEAPT    91
tr|S9U4L8|S9U4L8_9TRYP            TTFTAEVSKMLDILINSLYTNRAIFLRELISNGSDALDKVRVLYLTDPKNPVNKDGEPVT    99
tr|A0A7G2C3S2|A0A7G2C3S2_9TRYP    TTFTAEVSKMLDILINSLYTNRAIFLRELISNGSDALDKVRVTYLTEPKDPRNDAGEAPT    128
sp|P14625|ENPL_HUMAN              FAFQAEVNRMMKLIINSLYKNKEIFLRELISNASDALDKIRLISLTDENA--LSG--NEE    131
tr|Q7T3L3|Q7T3L3_DANRE            HAFQAEVNRMMKLIINSLYKNKEIFLRELISNASDALDKIRLLSLTNEDA--LAG--NEE    131
sp|Q9STX5|ENPL_ARATH              FEFQAEVSRLMDIIINSLYSNKDIFLRELISNASDALDKIRFLALTDKDV--LGEGDTAK    136
tr|A0A061FKM2|A0A061FKM2_THECC    FEFQAEVSRLMDIIINSLYSNKDIFLRELISNASDALDKIRFLSLTDKEV--LGEGDTAK    134
tr|Q5Z9N8|Q5Z9N8_ORYSJ            FEFQAEVSRLMDIIINSLYSNKDIFLRELISNASDALDKIRFLALTDKEV--LGEGDTAK    138
tr|W2ZJ09|W2ZJ09_PHYPR            FAFQAEVSRLMDILINSLYRTKEIFLRELISNASDALDKIRFLALSNNEL--LGK--LRD    118
tr|W2ZJ74|W2ZJ74_PHYPR            FAFQAEVSRLMDILINSLYRTKEIFLRELISNASDALDKIRFLALSNNEL--LGK--LRD    117
tr|F0Y644|F0Y644_AURAN            FEFQAEVNRLMDIIINSLYKNKEIFLREVISNGSDALDKIRFLAVSDAAA--LDT--KKE    90
```

```
tr|D8LHY7|D8LHY7_ECTSI            FEFQAEVNRLMDIIINSLYKNKDIFLRELISNASDALDKIRFLSVSDPDK--LGT--AKD    112
                                  : ***.:::.::::*** .: :****:***.******::   ::

tr|A0A0L1KBS8|A0A0L1KBS8_9EUGL    FDIHLKVDHEKRTLTLRDGGVGMTKDELSAHLGSLGASGTKKFVEKLDDTESKTQDDVNT    152
tr|A0A0L1KHT0|A0A0L1KHT0_9EUGL    MDVRILIDHETNELRLIDGGVGMTKSELSANLGSLGASGTKSFWENINESKNA-GEELSN    152
tr|A0A1X0P079|A0A1X0P079_9TRYP    LDMHVSIDPSNKTLTLRDGGIGMTREELENHLGSLGTSGTKRFMEKLQES------KDAS    153
tr|Q4DW89|Q4DW89_TRYCC            LEMRVIIDNERKTLTLRDGGIGMTKAELEEHLGSLGTSGTKRFMEKLKET------KDDS    151
tr|A0A422PWD8|A0A422PWD8_9TRYP    LEMRVTVDAERKTLTLRDGGIGMTREELEEHLGSLGTSGTKRFLERLKET------KDAS    152
tr|A0A422NRM6|A0A422NRM6_TRYRA    LEMRVTVDTERKTLTLRDGGIGMTRAELEEHLGSLGTSGTKRFMDRLKET------KDAS    152
tr|A0A0S4KLS2|A0A0S4KLS2_BODSA    MDIRIVVDREKRLFIMRDGGVGMTKEELAANLGSLGSSGTKRFLEKMKDS------SDAN    145
tr|A0A0N0VF86|A0A0N0VF86_LEPPY    MDIRISFNKEKSELVIRDGGVGMNKDDLAGHLGSLGTSGTKRFLEKLQEGG--TAGDQNN    150
tr|A4HH83|A4HH83_LEIBR            MDLRISFDNENHELILRDGGIGMTKEELTQHLGSLGSSGTKHFLEKLQEGSGAVGGDQSN    151
tr|E9ADS8|E9ADS8_LEIMA            MDLRISFDKEKSELILRDGGVGMTKEELAKHLGSLGTSGTKHFLEKLQEGVGAGGGDQNN    151
sp|Q9NGD0|ENPL_LEIIN              MDLRISFDKEKSELILRDGGVGMTKEELAKHLGSLGTSGTKHFLEKLQEGVGAGGGDQNN    151
tr|A4I4C9|A4I4C9_LEIIN            MDLRISFDKEKSELILRDGGVGMTKEELAKHLGSLGTSGTKHFLEKLQEGVGAVGGDQNN    151
sp|Q8T7E0|ENPL_LEIDO              MDLRISFDKEKSELILRDGGVGMTKEELAKHLGSLGTSGTKHFLEKLQEGVGAVGGDQNN    151
tr|S9U4L8|S9U4L8_9TRYP            MDIHISIDKEQKQLIIRDGGIGMTKEDLSKNLGSLGTSGTKRFLEGMKSG--ATSGDSSN    157
tr|A0A7G2C3S2|A0A7G2C3S2_9TRYP    MDIHISVSPETKELIIRDGGVGMTKEELGTHLGSLGTSGTKRFLENLKAS----STDNNN    184
sp|P14625|ENPL_HUMAN              LTVKIKCDKEKNLLHVTDTGVGMTREELVKNLGTIAKSGTSEFLNKMTEAQED-GQSTSE    190
tr|Q7T3L3|Q7T3L3_DANRE            LTIKIKSDKEKNMLHITDTGIGMTKEELVKNLGTIAKSGTSEFLNKMTEVQDD-SQSTSE    190
sp|Q9STX5|ENPL_ARATH              LEIQIKLDKAKKILSIRDRGIGMTKEDLIKNLGTIAKSGTSAFVEKMQ------SSGDLN    190
tr|A0A061FKM2|A0A061FKM2_THECC    LEIQIKLDKEKKILTLRDRGIGMTKEDLIKHLGTIAKSGTSAFVEKMQ------SSGDLN    188
tr|Q5Z9N8|Q5Z9N8_ORYSJ            LEIQIKLDKEKKILSIRDRGIGMTKEDLIKNLGTIAKSGTSAFVEKMQ------TGGDLN    192
tr|W2ZJ09|W2ZJ09_PHYPR            LEIRISFDKDAGTLTIRDTGVGMTKDDLVNNLGTVAKSGTANFVEAMQA---G--ADDSS    173
tr|W2ZJ74|W2ZJ74_PHYPR            LEIRISFDKDAGTLTIRDTGVGMTKDDLVNNLGTVAKSGTANFVEAMQA---G--ADDSS    172
tr|F0Y644|F0Y644_AURAN            LEIRISFDKDARTLTIQDSGVGMTKADLVANLGTVAKSGTTNFVEAMSG---D-ASGDLS    146
tr|D8LHY7|D8LHY7_ECTSI            LEIRISADKDARTLTIRDAGVGMTKADLISNLGTVARSGTTNFVEALT------ETGDLG    166
                                  : :::  .         : : * *:**.: :*  :**::. ***  * : :

tr|A0A0L1KBS8|A0A0L1KBS8_9EUGL    LIGQFGVGFYSVFLVADTIKVISKSDNSDEQWIWESNGDGKFQLYEDPNGNTLGRGTELV    212
tr|A0A0L1KHT0|A0A0L1KHT0_9EUGL    LIGQFGVGFYSVFLVADEVRVASKNDAEEKQWMWESKGDGKYFLYEDPRGNTLGRGTELV    212
tr|A0A1X0P079|A0A1X0P079_9TRYP    LIGQFGVGFYSAFLVADRVRVASKSDDSDKQWVWESAGDGQYYIYEDERGNTLGRGTEIT    213
tr|Q4DW89|Q4DW89_TRYCC            LIGQFGVGFYSAFLVADRVRVASKSDDSDVQWVWESAGDGQYYIYEDERGNTLGRGTEIT    211
tr|A0A422PWD8|A0A422PWD8_9TRYP    LIGQFGVGFYSAFLVAERVRVASKSDDNDTQWVWESAGDGQYFLYEDERGNTLGRGTEVT    212
tr|A0A422NRM6|A0A422NRM6_TRYRA    LIGQFGVGFYSAFLVAERVRVASKSDDSDTQWVWESTGDGQYYVYEDERGNTLGRGTEIT    212
tr|A0A0S4KLS2|A0A0S4KLS2_BODSA    FIGQFGVGFYSVFLVADKVRVASKHDDSEKQWVWESTGDGTFFLYEDERGNTLGRGSELT    205
tr|A0A0N0VF86|A0A0N0VF86_LEPPY    LIGQFGVGFYSVFLVGDRVRVVSKSDDGDEQYVWESSGNGQYFLYPDPRGNTLGRGTEIT    210
tr|A4HH83|A4HH83_LEIBR            LIGQFGVGFYSVFLVGNRVRVASKSDDSDEQYVWESKGDGEYFLYPDPRGNTLGRGTEIT    211
tr|E9ADS8|E9ADS8_LEIMA            LIGQFGVGFYSVFLVGDRVRVASKSDDSDEQYVWESKGDGQYFLYPDPRGNTLGRGTEIT    211
sp|Q9NGD0|ENPL_LEIIN              LIGQFGVGFYSVFLVGDRVRVASKSDDSDEQYVWESKGDGQYFLYPDPRGNTLGRGTEIT    211
tr|A4I4C9|A4I4C9_LEIIN            LIGQFGVGFYSVFLVGDRVRVASKSDDSDEQYVWESKGDGQYFLYPDPRGNTLGRGTEIT    211
sp|Q8T7E0|ENPL_LEIDO              LIGQFGVGFYSVFLVGDRVRVASKSDDSDEQYVWESKGDGQYFLYPDPRGNTLGRGTEIT    211
tr|S9U4L8|S9U4L8_9TRYP            LIGQFGVGFYSVFLVAKRVIVASKHDSSDVQYVWESHGDGRFFLYEDPRGNTLGRGTELR    217
tr|A0A7G2C3S2|A0A7G2C3S2_9TRYP    LIGQFGVGFYSVFLVGKNVRVASKSDADSKQYVWESSGNGQFFLYEDPRGNTLGRGTEIR    244
sp|P14625|ENPL_HUMAN              LIGQFGVGFYSAFLVADKVIVTSKHNN-DTQHIWESDSN-EFSVIADPRGNTLGRGTTIT    248
tr|Q7T3L3|Q7T3L3_DANRE            LIGQFGVGFYSAFLVADKVIVTSKHNN-DTQHMWESDSN-QFSVIEDPRGDTLGRGTTIT    248
sp|Q9STX5|ENPL_ARATH              LIGQFGVGFYSAYLVADYIEVISKHND-DSQYVWESKANGKFAVSEDTWNEPLGRGTEIR    249
tr|A0A061FKM2|A0A061FKM2_THECC    LIGQFGVGFYSVYLVADYVEVISKHND-DKQYVWESKADGAFAISEDTWNEPLGRGTEIR    247
tr|Q5Z9N8|Q5Z9N8_ORYSJ            LIGQFGVGFYSVYLVADYVEVISKHND-DKQHVWESKADGSFAISEDTWNEPLGRGTEIR    251
tr|W2ZJ09|W2ZJ09_PHYPR            LIGQFGVGFYSVYLVADRVRVVSKNNN-DDQYIWESDANASFTITKDPRGDTLGRGTEIT    232
tr|W2ZJ74|W2ZJ74_PHYPR            LIGQFGVGFYSVYLVADRVRVVSKNNN-DDQYIWESDANASFTITKDPRGDTLGRGTEIT    231
tr|F0Y644|F0Y644_AURAN            LIGQFGVGFYSVYLVADRVRVRSKHND-DDQHVWESAADSSFTVWQDPLGDTLGRGTEIK    205
tr|D8LHY7|D8LHY7_ECTSI            LIGQFGVGFYSVYLVADKVQVISKSND-DDQYVWESTADSTFTVSKDPRGNTLGRGTEIV    225
                                  :**********.:**.. : * ** :  . * :*** .:  : :  *  .: ****: :

tr|A0A0L1KBS8|A0A0L1KBS8_9EUGL    IYLRKDADEYMEEFKLKEIIHRYSQFVHFPIYLWNVKQKEKPKAKDENEEDSEDIVID--    270
tr|A0A0L1KHT0|A0A0L1KHT0_9EUGL    LKLKKEAAEEYLQEEKLKKIIHYYSEFINFPIYVGYATKDEREKQ----------------    256
tr|A0A1X0P079|A0A1X0P079_9TRYP    LEMKPDALEFLNSDNVRRIVHQYSEFVHFPIYMEKG------------------------    249
tr|Q4DW89|Q4DW89_TRYCC            LEMKPDALEFLSTDNVRDIVHQYSEFVHFPIYMQKG------------------------    247
tr|A0A422PWD8|A0A422PWD8_9TRYP    LEMKPDALEFLNIDGVRKIVHQYSEFVHFPIYMQKG------------------------    248
tr|A0A422NRM6|A0A422NRM6_TRYRA    LEMKPDALEFLSADNVRHIVHQYSEFVHFPIYMQKE------------------------    248
tr|A0A0S4KLS2|A0A0S4KLS2_BODSA    LELKKDADEYLDIDKVKEAIHKYSEFIHFPIYIQTTKTEKVKKAAEAESTEAK----E--    259
tr|A0A0N0VF86|A0A0N0VF86_LEPPY    IELKPDAEQFLSAETIKKTVHQYSEFINFPIYVEEEVTVEAAKKED--------ADTE--    260
tr|A4HH83|A4HH83_LEIBR            IELKPEDQEFLSAETIKKTIHQYSEFINFPIYVQEEVEVKPKTEIRTSEPGAEVGFMK--    269
tr|E9ADS8|E9ADS8_LEIMA            IELKPDAEQFLSAETIKKTIHQYSEFINFPIYVQEEVEVASTA--ATPEPAAEEGSLD--    267
sp|Q9NGD0|ENPL_LEIIN              IELKPDAEQFLSAETIKKTIHQYSEFINFPIYVQEEVEVASTA--ATPEPAAEEGSLD--    267
tr|A4I4C9|A4I4C9_LEIIN            IELKPDAEQFLSAETIKKTIHQYSEFINFPIYVQEEVEVASTA--ATPESAAEERSLD--    267
sp|Q8T7E0|ENPL_LEIDO              IELKPDAEQFLSAETIKKTIHQYSEFINFPIYVQEEVEVASTA--ATPESAAEERSLD--    267
tr|S9U4L8|S9U4L8_9TRYP            IELKPDSIEYLNMDRIKETIHQYSEFIDFPIHVQEQMAEDEVVKELQ----------A--    265
tr|A0A7G2C3S2|A0A7G2C3S2_9TRYP    IELKSDAVEYLKTDRVKKTIHQYSEFIDFPIYLEEAEKDNDQVQKEI----------E--    292
sp|P14625|ENPL_HUMAN              LVLKEEASDYLELDTIKNLVKKYSQFINFPIYVWSSKTETVEEPMEEEEAAKEEKEESD-    307
tr|Q7T3L3|Q7T3L3_DANRE            LVMKEEASDYLELETIKNLVKKYSQFINFPIYVWSSKTETVEEPIEDEAEAEKEEATED-    307
sp|Q9STX5|ENPL_ARATH              LHLRDEAGEYLEESKLKELVKRYSEFINFPISLWASKEVETEVPVEEDESADEETETTST    309
tr|A0A061FKM2|A0A061FKM2_THECC    LHLRDEAQEYLEEGKLKELVKKYSEFINFPIYIWASKEVDVEVPAEEDESSDEEETSDST    307
tr|Q5Z9N8|Q5Z9N8_ORYSJ            LHLRDEAKEYVEEDKLKDLVKKYSEFINFPIYLWATKEVDVEVPADEDESSESSEEEESS    311
tr|W2ZJ09|W2ZJ09_PHYPR            LFLKPDATEFQDQDKLKNLVGHYSEFITFPIYVNTTSTETYEVEEEPADVEEVVEEETDE    292
tr|W2ZJ74|W2ZJ74_PHYPR            LFLKPDATEFQDQDKLKNLVGHYSEFITFPIYVNTTSTETYEVEEEPADVEEVVEEETDE    291
tr|F0Y644|F0Y644_AURAN            LFLKEDAGEFLDQDRLEELVLRYSEFITFPIYLYKSKTETVEVDDDEDEDEEDLDEEEEE    265
tr|D8LHY7|D8LHY7_ECTSI            LHLKEDAGEFLSESTLKNLIHRYSEFITFPIYQLVEKEEEIEVEDDEEEEEDDGDEDSGD    285
                                  : :: :  :: .   :.  :  **:*: ***

tr|A0A0L1KBS8|A0A0L1KBS8_9EUGL    --------EENEDEKQEEVVSEEKNEPEVEQWVWEHVNEHEPIWIRDPAEVKEDDYVSFY    322
tr|A0A0L1KHT0|A0A0L1KHT0_9EUGL    -------------------------DEKYQGIDWELVNVIKPIWTRNPQTVERKEYVEFY    291
tr|A0A1X0P079|A0A1X0P079_9TRYP    ------------------------------DKWEVVNENKPIWTRKPSEVTEEEYVKFY    278
tr|Q4DW89|Q4DW89_TRYCC            ------------------------------EKWEVVNENKPIWTRKPSEVTEEEYHKFY    276
tr|A0A422PWD8|A0A422PWD8_9TRYP    ------------------------------DKWEVVNENKPIWTRKPAEVAADEYHKFY    277
tr|A0A422NRM6|A0A422NRM6_TRYRA    ------------------------------DKWEVVNENKPIWTRKPSEVTPDEYHKFY    277
```

```
tr|A0A0S4KLS2|A0A0S4KLS2_BODSA    --------DGDDE---APAEEKKVEEEEVVTHDWELINENKPIWTRKAVEITDAEYNSFF    308
tr|A0A0N0VF86|A0A0N0VF86_LEPPY    --------DKALD---EDEVEDEVEAVPVTERKWTLVNENRPIWTRPIGNVTEAEYNKFY    309
tr|A4HH83|A4HH83_LEIBR            --------EVVVE---EDNN--GEEKVPRMEKRWTLINENRPIWTRQIGNVTEEEYIKFY    316
tr|E9ADS8|E9ADS8_LEIMA            --------EGAVE---EDSDKEGGTQGVAKERRWVLVNENRPIWTRPIGNVTEEEYHKFY    316
sp|Q9NGD0|ENPL_LEIIN              --------EGAVE---EDPDKEGDTQGVVKERRWTLVNENRPIWTRPIGNVTEEEYHTFY    316
tr|A4I4C9|A4I4C9_LEIIN            --------EGAVE---EDPDKEGDTQGVVKEKRWTLVNENRPIWTRPIGNVTEEEYHKFY    316
sp|Q8T7E0|ENPL_LEIDO              --------EGAVE---EDPDKEGDTQGVVKEKRWTLVNENRPIWTRPIGNVTEEEYHKFY    316
tr|S9U4L8|S9U4L8_9TRYP            --------QGMVP---E----EVEAASKEPMYRWTLVNKNRPIWTRSFDEVKEADYDSFY    310
tr|A0A7G2C3S2|A0A7G2C3S2_9TRYP    --------YG------E------DVENTTGNKTWTLVNKNKPIWTRPTSEVTEEEYDSFY    332
sp|P14625|ENPL_HUMAN              --------DEAAVEEEEEEKKPKTKKVEKTVWDWELMNDIKPIWQRPSKEVEEDEYKAFY    359
tr|Q7T3L3|Q7T3L3_DANRE            --------EAEVEEEEEDKDKPKTKKVEKTVWDWELMNDIKPIWQRPAKEVEEDEYTAFY    359
sp|Q9STX5|ENPL_ARATH              E-EEK----EEDAEEEDGEKKQKTKKVKETVYEWELLNDVKAIWLRSPKEVTEEEYTKFY    364
tr|A0A061FKM2|A0A061FKM2_THECC    S-EEGEDEDAEKSEDEGTEKKPKTKKVKETSNEWELLNDVKAIWLRSPKEVTEEEYTKFY    366
tr|Q5Z9N8|Q5Z9N8_ORYSJ            P-ESTEEE----ETEESEEKKPKTKTVKETTTEWELLNDVKAIWLRSPKEVTEEEYTKFY    366
tr|W2ZJ09|W2ZJ09_PHYPR            E-KPSEEDEELEAVEEEDVKTPKTRAETRTVWKWERVNEVKAIWTRSKDEISDEEYESFY    351
tr|W2ZJ74|W2ZJ74_PHYPR            E-KPSEEDEELEAVEEEDVKTPKTRAETRTVWKWERVNEVKAIWTRSKDEISDEEYESFY    350
tr|F0Y644|F0Y644_AURAN            ESEEDLEDDEYE-----EEEDDEPRTTTVTNWDWHQVNSESAIWSRDASDVDDEEYRSFY    320
tr|D8LHY7|D8LHY7_ECTSI            E--DGDEEDEFE-----EV-EVDVKYKTVKTLDWERVNANVAVWARDKDEITDEEYHNFY    337
                                                                   *  :*    :* *    :   :*  *:

tr|A0A0L1KBS8|A0A0L1KBS8_9EUGL    KSMSHE--YEDPMHHYHFASKGDDVGFKSILYVPGRAPHDGYNSK-KGASHDNIRLYVRR    379
tr|A0A0L1KHT0|A0A0L1KHT0_9EUGL    RGFNKE--FDEPMYYSHFVASGGEIDFKALLYVPSTLPTNAFNVHD-ESSYKNMRLYVRR    348
tr|A0A1X0P079|A0A1X0P079_9TRYP    KALTHD--YRSPMYYSHFTVE-GEVEFSSVLFIPEEATQDIFINNE--NTRDNIKLYVRR    333
tr|Q4DW89|Q4DW89_TRYCC            KSLTHD--YRNPMYYSHFNVE-GEVEFSSVLFIPQEASQDIFVNNE--DTRDNIKLYVRR    331
tr|A0A422PWD8|A0A422PWD8_9TRYP    KALTHD--YRDPMYYSHFVVE-GEVEFSSVLFIPQEASQDIFVNNE--NTRDNIKLYVRR    332
tr|A0A422NRM6|A0A422NRM6_TRYRA    KTLTRD--YRDPMYYSHFMVE-GEVEFSSVLFIPQEASQDIFVNNE--NTRDNIKLYVRR    332
tr|A0A0S4KLS2|A0A0S4KLS2_BODSA    KSLTKD--YDDPMFYTHFSAE-GEVEFRSILFIPSHSNTNVF---DTSVVQANIRLYVRR    362
tr|A0A0N0VF86|A0A0N0VF86_LEPPY    KAFSGD--YRDPLYFNHFRVE-GEVEFDSILFVPAAVDPATF-SDDNTVPNTNIKLYVRR    365
tr|A4HH83|A4HH83_LEIBR            KAFSGD--YRDPLYFSHFKVE-GEVDFDSILFIPSTVDPASF-SDDKANPSTNIKLYVRR    372
tr|E9ADS8|E9ADS8_LEIMA            KAFSGD--YRDPLYFSHFKVE-GEVDFDSILFVPTTVDPASF-SDDNAAPNTNIKLYVRR    372
sp|Q9NGD0|ENPL_LEIIN              KAFSGD--YRDPLYFNHFKVE-GEVDFDSILFVPTTVDPASF-SDDNSVPNTNIKLYVRR    372
tr|A4I4C9|A4I4C9_LEIIN            KAFSGD--YRDPLYFNHFKVE-GEVDFDSILFVPTTVDPASF-SDDNAVPNTNIKLYVRR    372
sp|Q8T7E0|ENPL_LEIDO              KAFSGD--YRDPLYFNHFKVE-GEVDFDSILFVPTTVDPASF-SDDNAVPNTNIKLYVRR    372
tr|S9U4L8|S9U4L8_9TRYP            KALTHD--YREPLYYSHFKVE-GEVEFDSILFIPTEASEEEL---DSTQRTKNIKLYVRR    364
tr|A0A7G2C3S2|A0A7G2C3S2_9TRYP    KAVSSD--WRTPMYYSHFHVE-GEVEFDSILYIPEIINREEQATNDASMLNRNIKLYVRR    389
sp|P14625|ENPL_HUMAN              KSFSKE--SDDPMAYIHFTAE-GEVTFKSILFVPTSAPRGLFDEYG-SKKSDYIKLYVRR    415
tr|Q7T3L3|Q7T3L3_DANRE            KTFSRD--TDEPLSHIHFTAE-GEVTFKSILFVPASAPRGLFDEYG-TKKNDFIKLFVRR    415
sp|Q9STX5|ENPL_ARATH              HSLSKDFTDEKPMAWSHFNAE-GDVEFKAVLYVPPKAPHDLYESYY-NSNKANLKLYVRR    422
tr|A0A061FKM2|A0A061FKM2_THECC    QSLAKDFGDEKPLAWSHFTAE-GDVEFKAVLFVPPKAPHDLYESYY-NTNKANLKLYVRR    424
tr|Q5Z9N8|Q5Z9N8_ORYSJ            HSLAKDFGDDKPLSWSHFTAE-GDVEFKALLFVPPKAPHDLYESYY-NSNKSNLKLYVRR    424
tr|W2ZJ09|W2ZJ09_PHYPR            HSLQKTD-ITDPLTWIHFQAE-GEIEFKSILYVPGQAPRDLYTRFE-NK-KADIKLYVRK    407
tr|W2ZJ74|W2ZJ74_PHYPR            HSLQKTD-ITDPLTWIHFQAE-GEIEFKSILYVPGQAPRDLYTRFE-NK-KADIKLYVRK    406
tr|F0Y644|F0Y644_AURAN            KTLSK-D-TADPTTWIHFKAE-GEVEFKSILFVPGQVPFDMYDSYH-TK-SAQLRLYVRK    375
tr|D8LHY7|D8LHY7_ECTSI            KALSG-D-TSDAATWIHFKAE-GEVEFKSILFAPSKAPHNMYDHYY-DDSRGGLKLYVRK    393
                                  : .             **  . .:: * ::*: *                   ::*:**:

tr|A0A0L1KBS8|A0A0L1KBS8_9EUGL    IFITDEFKDLLPRYLSFVRGVVDSDDLPLNVSREILQESKIIKLIQKKLVRKCIDMFVEM    439
tr|A0A0L1KHT0|A0A0L1KHT0_9EUGL    VFITDEFKDLFPRYLNFVQGVVDSDDLPLNVSRELLQENRVLKTIKKQLVRKALGMFIEI    408
tr|A0A1X0P079|A0A1X0P079_9TRYP    IFITDEFRELLPRYLSFVRGVVDSNDLPLNVSREVLQESRILRVIKKKLVRKALGMISEI    393
tr|Q4DW89|Q4DW89_TRYCC            IFITDEFRQLLPRYLSFVRGIVDSNDLPLNVSREVLQESRILRVIKKKLVRKALSMISEI    391
tr|A0A422PWD8|A0A422PWD8_9TRYP    IFITDEFRELLPRYLNFVRGVVDSNDLPLNVSREVLQESRILRVIKKKLVRKALSMIAEI    392
tr|A0A422NRM6|A0A422NRM6_TRYRA    IFITDEFRELLPRYLSFVRGVVDSNDLPLNVSREVLQESRILRVIKKKLVRKALSMIAEI    392
tr|A0A0S4KLS2|A0A0S4KLS2_BODSA    VFITDDFRDLLPRYLNFIKGVVDSDDLPLNVSREVLQESRILRVIKKKLVRKALAMIADI    422
tr|A0A0N0VF86|A0A0N0VF86_LEPPY    VFITDEFRDLLPRYLNFVKGIVDSNDLPLNVSREVLQESRILRVIKKKLVRKALTMFSDI    425
tr|A4HH83|A4HH83_LEIBR            VFITDEFRDLLPRYLNFVKGIVDSNDLPLNVSREVLQESRILRVIKKKLVRKALNMFANI    432
tr|E9ADS8|E9ADS8_LEIMA            VFITDEFRDLLPRYLNFVKGIVDSNDLPLNVSREVLQESRILRVIKKKLVRKTLSMFADI    432
sp|Q9NGD0|ENPL_LEIIN              VFITDEFRDLLPRYLNFVKGIVDSNDLPLNVSREVLQESRILRVIKKKLVRKTLSMFADI    432
tr|A4I4C9|A4I4C9_LEIIN            VFITDEFRDLLPRYLNFVKGIVDSNDLPLNVSREVLQESRILRVIKKKLVRKTLSMFADI    432
sp|Q8T7E0|ENPL_LEIDO              VFITDEFRDLLPRYLNFVKGIVDSNDLPLNVSREVLQESRILRVIKKKLVRKTLSMFADI    432
tr|S9U4L8|S9U4L8_9TRYP            VFITDDFKDLLPRYLSFVKGVVDSNDLPLNVSRELLQESRVLQVIKKKLVRKALAMFADI    424
tr|A0A7G2C3S2|A0A7G2C3S2_9TRYP    VFITDEFKDLLPHYLNFVRGVVDSNDLPLNVSREVLQESRVLRVIKRKLVRKVLSMIADI    449
sp|P14625|ENPL_HUMAN              VFITDDFHDMMPKYLNFVKGVVDSDDLPLNVSRETLQQHKLLKVIRKKLVRKTLDMIKKI    475
tr|Q7T3L3|Q7T3L3_DANRE            VFITDDFHDMMPKYLNFIKGVVDSDDLPLNVSRETLQQHKLLKVIRKKLVRKTLDMIKKI    475
sp|Q9STX5|ENPL_ARATH              VFISDEFDELLPKYLSFLKGLVDSDTLPLNVSREMLQQHSSLKTIKKKLIRKALDMIRKL    482
tr|A0A061FKM2|A0A061FKM2_THECC    VFISDEFDELLPKYLNFLKGLVDSDTLPLNVSREMLQAHSSLKTIKKKLIRKALDMIRKI    484
tr|Q5Z9N8|Q5Z9N8_ORYSJ            VFISDEFDELLPKYLSFLKGLVDSDTLPLNVSREMLQQHSSLKTIKKKLIRKALDMIRKL    484
tr|W2ZJ09|W2ZJ09_PHYPR            VLITDDFDDFLPRYLNFIAGVVDSDDLPINVSRETLQENKILRVIRKKLVRKVLEMLRKL    467
tr|W2ZJ74|W2ZJ74_PHYPR            VLITDDFDDFLPRYLNFIAGVVDSDDLPINVSRETLQENKILRVIRKKLVRKVLEMLRKL    466
tr|F0Y644|F0Y644_AURAN            VLITDEFEDLVPRYLNFLRGVVDSDDLPLNVSRETLQQHKVLKVMGKKLVRKALEMLRKL    435
tr|D8LHY7|D8LHY7_ECTSI            VLITDEFDELLPRYLSFIRGVVDSDDLPLNVSREMLQEHKVLKVMAKKLVRKTLEMLRKL    453
                                  ::*:*:* ::.*:**.*: *:***: **:***** **    :: : ::*:** : *: .:

tr|A0A0L1KBS8|A0A0L1KBS8_9EUGL    AEDESGIKQKRKLRKK----------------------KREDGGEEFSYSKFWREFGKS    476
tr|A0A0L1KHT0|A0A0L1KHT0_9EUGL    SKEDDEVWKEIDATE---------------------DKELKEKKFSELKYPAFWDEYGKS    447
tr|A0A1X0P079|A0A1X0P079_9TRYP    AETDARIKDEMKDKPK-------DE-------NKT-EDKGSIKHPKEPLYPKFWAQFGKH    438
tr|Q4DW89|Q4DW89_TRYCC            AEKDARLKEGLEKEKSGEDGEAKDE-------NTTEKKS-DDDKGKEPLYPKFWAQFGKH    443
tr|A0A422PWD8|A0A422PWD8_9TRYP    AENDSRLKERLAKEKAEQKAT------------DAKDGDGDGKAKEEPMYPKFWAQFGKH    440
tr|A0A422NRM6|A0A422NRM6_TRYRA    GENDARLKERLEKEKTAAVEEQADK-------NATETKDDDDKSKKEPMYPKFWAQFGKH    445
tr|A0A0S4KLS2|A0A0S4KLS2_BODSA    AASDKKLEAAKDDDEAAEAEEK------------KDDVTAGNKQLKASTYPKFWEEYGKN    470
tr|A0A0N0VF86|A0A0N0VF86_LEPPY    AEQDEAIAKGT----QSES----------------PAP-SGHTHLTQPTYAKFWELFGKH    464
tr|A4HH83|A4HH83_LEIBR            AAQDVEIANKD----HAED----------------PAP-SGHTHLKKPTYAKFWDLFGKH    471
tr|E9ADS8|E9ADS8_LEIMA            AAQDEAIADGK----QVEN----------------PAL-SGHTHLKKPAYTKFWELYGKH    471
sp|Q9NGD0|ENPL_LEIIN              AAQDEAIANGK----QVES----------------PAP-SGHTHLKKPAYTKFWELYGKH    471
tr|A4I4C9|A4I4C9_LEIIN            AAQDEAIANGK----QVEN----------------PAP-SGHTHLKKPAYTKFWELYGKH    471
sp|Q8T7E0|ENPL_LEIDO              AAQDEAIANGK----QVEN----------------PAP-SGHTHLKKPAYTKFWELYGKH    471
tr|S9U4L8|S9U4L8_9TRYP            QAQDDRIAVLKKEGREVET----------------DAPNKGVRVLTKPTYKTFWELFGRH    468
tr|A0A7G2C3S2|A0A7G2C3S2_9TRYP    QVQDDNLRKLKEENKTFE-----------------PAKGTGVRQLKVETYPKFWETYGRF    492
```

```
sp|P14625|ENPL_HUMAN              ADDK--------------------------------------------YNDTFWKEFGTN    491
tr|Q7T3L3|Q7T3L3_DANRE            AEEQ--------------------------------------------YNDKFWKEFGTN    491
sp|Q9STX5|ENPL_ARATH              AEEDPDEIHDDEKKDVEK--------------------SGE-NDEKKGQYTKFWNEFGKS    521
tr|A0A061FKM2|A0A061FKM2_THECC    AEEDPDESSGKDEKEVEK--------------------SGTDDDEKKGQYAKFWNEFGKS    524
tr|Q5Z9N8|Q5Z9N8_ORYSJ            AEEDPDEYSNKDKTDEEK--------------------SA--MEEKKGQYAKFWNEFGKS    522
tr|W2ZJ09|W2ZJ09_PHYPR            AEKDEDDDEEDEEDDEVVDTSADESNDKKETEGDAEKEEEEEEEDGNTAYNKFWEEFGKN    527
tr|W2ZJ74|W2ZJ74_PHYPR            AEKDEDDDEEDEEDDEVVDTSADESNDKKETEGDAEKEEEEEEEDGNTAYNKFWEEFGKN    526
tr|F0Y644|F0Y644_AURAN            SQLKEKGDDDEDEDDEES------------------EDADESEDDDEDPYIGFWSKFGKN    477
tr|D8LHY7|D8LHY7_ECTSI            ATEE----------------------------------VEDEEGEEEHPYIQFWDEFGKS    479
                                           .                                                  **  :*

tr|A0A0L1KBS8|A0A0L1KBS8_9EUGL    IRLGIIEDSSNRQRLARLVRYPSSTTEFKATESSTSLNEYISRMKPRQRDIFYVVGESTS    536
tr|A0A0L1KHT0|A0A0L1KHT0_9EUGL    LRLGLVEDGGNRMRITKLLRYRSSMYPFHYN-KLVSLDDYVSRMKEKQNHIFFVVGEDIA    506
tr|A0A1X0P079|A0A1X0P079_9TRYP    IRLGILEDANNRGRLAKLLRYASSKS---NG-TLISLQEYTDRMKPDQKNIYYLTGDSVD    494
tr|Q4DW89|Q4DW89_TRYCC            IRLGILEDANNRGRLAKLLRYTSTKS---NG-TLVSLQEYTDRMKPEQKHIYFLTGDSVK    499
tr|A0A422PWD8|A0A422PWD8_9TRYP    IRLGILEDANNRGRLAKLLRYTSSKS---NG-TLVSLQDYTDRMKPGQKSIYFLTGESVS    496
tr|A0A422NRM6|A0A422NRM6_TRYRA    IRLGIIDDANNRGRLAKLLRYTSSTS---NG-TLVSLQEYTDRMKPGQKNIYFLTGDSVS    501
tr|A0A0S4KLS2|A0A0S4KLS2_BODSA    IRLGMIEDGSNRARLTKLLRYKSSKS---DN-KLISLQDYVDRMPESQKDIYYVSAESIE    526
tr|A0A0N0VF86|A0A0N0VF86_LEPPY    LRLGVMLDSNNRNRLTKLFRYKSSKS---DG-AYISLQTYVDRMKKGQKGIYYISGDTIE    520
tr|A4HH83|A4HH83_LEIBR            LRLGAITDSNNRNRLMKLFRYKSSAS---ET-EYISLQAYVDRMKKGQKGIYYLSGDSVD    527
tr|E9ADS8|E9ADS8_LEIMA            LRLGVMLDSNNRNRLTKLFRYKSSRS---ES-EYISLQTYVDRMKKGQKGIYYLSGDSVA    527
sp|Q9NGD0|ENPL_LEIIN              LRLGVMLDSNNRNRLTKLFRYKSSRS---ES-EYISLQTYVDRMKKGQKGIYYLSGDSVA    527
tr|A4I4C9|A4I4C9_LEIIN            LRLGVMLDSNNRNRLTKLFRYKSSRS---ES-EYISLQTYVDRMKKGQKGIYYLSGDSVA    527
sp|Q8T7E0|ENPL_LEIDO              LRLGVMLDSNNRNRLTKLFRYKSSRS---ES-EYISLQTYVDRMKKGQKGIYYLSGDSVA    527
tr|S9U4L8|S9U4L8_9TRYP            LRYGVVSDPNNRGRLLKLLRYRSSMS---RG-KYVTLKEYTDRMKKGQRGIYYLTGDSIE    524
tr|A0A7G2C3S2|A0A7G2C3S2_9TRYP    LRYGLIVDPSNRGRITKLLRYKTSRS---ED-RYVGLQDYIDHMQKGQKAIYYLSGDSIA    548
sp|P14625|ENPL_HUMAN              IKLGVIEDHSNRTRLAKLLRFQSSHH---PT-DITSLDQYVERMKEKQDKIYFMAGSSRK    547
tr|Q7T3L3|Q7T3L3_DANRE            IKLGVIEDHSNRTRLAKLLRFQTSHS---DT-VLSSLEQYVERMKEKQDKIYFMAGTSRK    547
sp|Q9STX5|ENPL_ARATH              VKLGIIEDAANRNRLAKLLRFETTKS---DG-KLTSLDQYIKRMKKSQKDIFYITGSSKE    577
tr|A0A061FKM2|A0A061FKM2_THECC    IKLGIIEDATNRNRLAKLLRFESTKS---DG-KLTSLDQYISRMRAGQKDIFYITGNSKE    580
tr|Q5Z9N8|Q5Z9N8_ORYSJ            VKLGIIEDATNRNRLAKLLRFESTKS---EG-KLASLDEYISRMKPGQKDIFYITGSSKE    578
tr|W2ZJ09|W2ZJ09_PHYPR            IKLGVMDDAANRAKLVKLLRFVTSES---DG-KWTSLEQYVDRMKDWQDSIYYIAAENAD    583
tr|W2ZJ74|W2ZJ74_PHYPR            IKLGVMDDAANRAKLVKLLRFVTSES---DG-KWTSLEQYVDRMKDWQDSIYYIAAENAD    582
tr|F0Y644|F0Y644_AURAN            IKLGIIEDSANRSKLTKLLRYKSNKS---GE-GYVSLEEYVENMKDWQKAIYYIAGESVA    533
tr|D8LHY7|D8LHY7_ECTSI            IKLGVMEDNANKSKLVKLLRFKTNKS---DG-KWVSLEDYVAGMPEWQSSIFYIAGESTE    535
                                  :: * : *  *: :: :*.*: :.          *. *   *   *  *::: .

tr|A0A0L1KBS8|A0A0L1KBS8_9EUGL    KLSKLAVVEDARNHGIEVLLMDDAIDEYVVASLTQYGGKNLVNLSKEGVQLEELTDY-EK    595
tr|A0A0L1KHT0|A0A0L1KHT0_9EUGL    KLSHLPVVEDAMNRGVEVLYMVDAIDEYVTSSVTDYSGYRLVNLALNMAQPEPPTEL-EE    565
tr|A0A1X0P079|A0A1X0P079_9TRYP    KMKQSPHMEEALQRDVEVIFMTDAIDEYVVGQIHDFANKKLINLAKEGAQFDEQTDK-EK    553
tr|Q4DW89|Q4DW89_TRYCC            KMRQSPHIEEALERDVEVLFMTDAIDEYVVSQVQDFGNKRLINLAKDNARLDEPTER-DK    558
tr|A0A422PWD8|A0A422PWD8_9TRYP    KMKQTPHIEEALQRDVEVLFMTDAIDEYVVSQIQDFANKRLVNLAKDSVQFEEQTEK-EK    555
tr|A0A422NRM6|A0A422NRM6_TRYRA    KMKQSPHIEEALQRGVEVLFMTDAIDEYVASQIHDFASKRLINLAKESAQFEELTER-EK    560
tr|A0A0S4KLS2|A0A0S4KLS2_BODSA    KIKQLPVLEDATNRNLEVLFMTDAIDEYVVGHVTDFAGKKLVNLAKEGVKFEDESKR-EK    585
tr|A0A0N0VF86|A0A0N0VF86_LEPPY    RIQKSPMLEDAVNHDVEVIFMTDAIDEYVVAQVTDFAGKKLINLAKEGVQFEETDAR-QR    579
tr|A4HH83|A4HH83_LEIBR            RIKKSPVLEDALNHGFEVIFMTDPIDEYAVSHLTDFAGNKLINLAKEGVAFEETDSR-QR    586
tr|E9ADS8|E9ADS8_LEIMA            RIKKSPVLEDAVNHDVEVIFMTDAIDEYVVSQLTDFAGKKLINLAKEGVQFEESDAR-QR    586
sp|Q9NGD0|ENPL_LEIIN              RIKKSPVLEDAVNHDVEVIFMTDAIDEYVVSQLTDFAGKKLINLAKEGVQLEESDAR-QR    586
tr|A4I4C9|A4I4C9_LEIIN            RIKKSPVLEDAVNHDVEVIFMTDAIDEYVVSQLTDFAGKKLINLAKEGVQFEESDAR-QR    586
sp|Q8T7E0|ENPL_LEIDO              RIKKSPVLEDAVNHDVEVIFMTDAIDEYVVSQLTDFAGKKLINLAKEGVQFEESDAR-QR    586
tr|S9U4L8|S9U4L8_9TRYP            LIERSPLLQDAKKRGIEVLYMTDTIDEYVVNNVPDFAGKKLINIAAADADYEEDDDR-QR    583
tr|A0A7G2C3S2|A0A7G2C3S2_9TRYP    KIKTSPLLQDALKRNVEVIYMTDAVDEYVVAQVPDFSGKKLVNLAKEGLQFDEENSPEAL    608
sp|P14625|ENPL_HUMAN              EAESSPFVERLLKKGYEVIYLTEPVDEYCIQALPEFDGKRFQNVAKEGVKFDESEKTKES    607
tr|Q7T3L3|Q7T3L3_DANRE            EAESSPFVEKLLKKGYEVVYLTEPVDEYCIQALPEFDGKRFQNVAKEGVKFDESDKAKEK    607
sp|Q9STX5|ENPL_ARATH              QLEKSPFLERLIKKGYEVIFFTDPVDEYLMQYLMDYEDKKFQNVSKEGLKVGKDSKDK--    635
tr|A0A061FKM2|A0A061FKM2_THECC    QLEKSPFLERLKKKNYEVIFFTDPVDEYLMQYLMDYDGKQFQNVSKEGLKIGKDSKNK--    638
tr|Q5Z9N8|Q5Z9N8_ORYSJ            QLEKSPFLERLTKKNYEVIYFTDPVDEYLMQYLMDYEDKKFQNVSKEGLKLGKDSKLK--    636
tr|W2ZJ09|W2ZJ09_PHYPR            ACEKSPFMEKMRAKGLEVLYFVDALDEYMVSHISEFDGKKLVSITKEGIKFGDEDESLTQ    643
tr|W2ZJ74|W2ZJ74_PHYPR            ACEKSPFMEKMRAKGLEVLYFVDALDEYMVSHISEFDGKKLVSITKEGIKFGDEDESLTQ    642
tr|F0Y644|F0Y644_AURAN            AVEESPFLEKCKAKDLEVLYLVDPIDEYAIQHVTEFDGKKLQSVTKEGLKFGDENEDVEK    593
tr|D8LHY7|D8LHY7_ECTSI            AVEKSPFLEKLNKKGLEVLYLTEPIDEMTMGSITDFEDKKMQSVTKEGLSFGDEDVADVK    595
                                           ::      :. **: : : :**   : :: . .: .::

tr|A0A0L1KBS8|A0A0L1KBS8_9EUGL    EIEVLREKKYATFIQWFSKIIEKATHRKINKIVLGKRKTEQPLVLSSPSDGMTPNLARIL    655
tr|A0A0L1KHT0|A0A0L1KHT0_9EUGL    SLKEKREEKYKPLIEWFGKLLEDHKV-HSDKIVLCHRHTSRPAIIASPKIGTTANMARIN    624
tr|A0A1X0P079|A0A1X0P079_9TRYP    AIEKKRIEKYRPLTDRLARMFKRSNV---RKVILTKRQTSEPFILSSQEGELTARMVNIM    610
tr|Q4DW89|Q4DW89_TRYCC            AIEKERKKKYEPLTERLLTLFGKSQV---RKVILTRRQSSEAFILSTQENDMTPRMVNVM    615
tr|A0A422PWD8|A0A422PWD8_9TRYP    AIEKKRIEKYQPLTERLANLFKKNDV---RKVILTKRQSSEAFILSTQENDLTPRMVNVM    612
tr|A0A422NRM6|A0A422NRM6_TRYRA    AIEKKRIERYEPLTSRLATLFKKSGV---RKVILTKRQSSEAFILSTQESDLTPRMVSVM    617
tr|A0A0S4KLS2|A0A0S4KLS2_BODSA    AIDAKRKEKYEPVLKYFKDLLGE-QV---TKVVLTKRKTSEPIILSSRQHDVTARMANII    641
tr|A0A0N0VF86|A0A0N0VF86_LEPPY    VIDKKRKEKYEALFTRLRSLFGYAEV---RKVILTKRLTNEAFILSSGENQITARLANIM    636
tr|A4HH83|A4HH83_LEIBR            VVDKKRREKYNSLFTHLRGIFGYSEV---RKVILTRRMTNEAFIVSSGDNQITARLASIM    643
tr|E9ADS8|E9ADS8_LEIMA            VADRKRKEKYDSFFTHLRVLFGYSEV---RKVILTKRMTNEAFIVSSGENQITARLASIM    643
sp|Q9NGD0|ENPL_LEIIN              VADRKRKEKYDSFFTHLRALFGYSEV---RKVILTKRMTNEAFLVSSGENQITARLASIM    643
tr|A4I4C9|A4I4C9_LEIIN            VVDRKRKEKYDSFFTHLRALFGYSEV---RKVILTKRMTNEAFIVSSSENQITARLASIM    643
sp|Q8T7E0|ENPL_LEIDO              VVDRKRKEKYDSFFTHLRALFGYSEV---RKVILTKRMTNEAFIVSSSENQITARLASIM    643
tr|S9U4L8|S9U4L8_9TRYP            ALRQKREERFEPLFSLLRQLFGEKEV---KKIVLTKRHTDLPFLLTNDENHMSIRMQKLM    640
tr|A0A7G2C3S2|A0A7G2C3S2_9TRYP    NLEKKRNEKYAPLLDRLKRLFGK-QV---RKLVLTKRYTTEPFLLIADENHLTARMANIL    664
sp|P14625|ENPL_HUMAN              --REAVEKEFEPLLNWMKDKALKDKI---EKAVVSQRLTESPCALVASQYGWSGNMERIM    662
tr|Q7T3L3|Q7T3L3_DANRE            --REALEKEFEPLTTWMKDKALKEQI---EKAVLSQRLTNSPCALVASQYGWSGNMERIM    662
sp|Q9STX5|ENPL_ARATH              ----ELKEAFKELTKWWKGNLASENV---DDVKISNRLADTPCVVVTSKFGWSANMERIM    688
tr|A0A061FKM2|A0A061FKM2_THECC    ----ELKESFKELTKWWKGALTSENV---DDVKITNRLDNTPCVVVTSKFGWSANMERIM    691
tr|Q5Z9N8|Q5Z9N8_ORYSJ            ----DLKESFKELTDWWKKALDTESV---DSVKISNRLSDTPCVVVTSKYGWSANMEKIM    689
tr|W2ZJ09|W2ZJ09_PHYPR            KREQLYADKYVALTTALKT-LYGDKI---SRVTMSQRVVDSPAVMVTSQWGYSANMQRIM    699
tr|W2ZJ74|W2ZJ74_PHYPR            KREQLYADKYVALTTALKT-LYGDKI---SRVTMSQRVVDSPAVMVTSQWGYSANMQRIM    698
tr|F0Y644|F0Y644_AURAN            KRMQLYKETFKPLTDYLKA-LYGDAV---VKVSVSRRVETTPTVIVTSQYGNSANMERIM    649
tr|D8LHY7|D8LHY7_ECTSI            KREKYYKKMFTPLAEHLKD-MFKGKI---SKVSVSQRVEGTPAIIVSAAYGYSANMERIM    651
```

```
                                           . :  .                    : .*    :        : .:  :

tr|A0A0L1KBS8|A0A0L1KBS8_9EUGL    KGQTFGDTRM--YVKD--AEKIVEVNYLHPAVEEIFNRFQEDENDEVSMNAALLLYETAC    711
tr|A0A0L1KHT0|A0A0L1KHT0_9EUGL    KGQTLGDH----GHKS--ADKIVEINHLSPIMDEIYRRVIVDENDEVAKNAAILVFETAC    678
tr|A0A1X0P079|A0A1X0P079_9TRYP    NQQAISSM----QQMH--HTRVLEINYRHPLVQDLLKRFEADPKDQVAVDVAWVLFGTAN    664
tr|Q4DW89|Q4DW89_TRYCC            NQQAISSL----HTMR--YSRVLELNHRHPLVRDLLTRFEADANDQTAIDVAWVLFGTAN    669
tr|A0A422PWD8|A0A422PWD8_9TRYP    NQQAISST----QTMR--YSRVLEINHRHPLVLDLLTRFKADPKDQTAIDVAWVLFGTAN    666
tr|A0A422NRM6|A0A422NRM6_TRYRA    NQQAISSS----QMMR--YSRVLEINHRHPLVRDLLTRFESDPKDQTAIDVAWVLFGTAN    671
tr|A0A0S4KLS2|A0A0S4KLS2_BODSA    RGQALGDAKQ-NEAQT--AKRVMEINHLHPLIEEIFKRVKADDKDKVAEDVALVLFDTAN    698
tr|A0A0N0VF86|A0A0N0VF86_LEPPY    RGQSMALM---DQQTT--AERVLEVNYRHPLVDEMFKRFAVDEDDAVAMDVAWVLYDTAN    691
tr|A4HH83|A4HH83_LEIBR            RGQSMSLA---DQKVT--AERVLEVNYRHPLVDEMFKRFTVNEDDEVATDIAWVLYDTAN    698
tr|E9ADS8|E9ADS8_LEIMA            RGQSMSLA---NQQMT--AERVLEVNYRHPLVDEMFKRFTVDENDEVATDIAWVLYDTAN    698
sp|Q9NGD0|ENPL_LEIIN              RGQSMSLA---NQQMT--AERVLEVNYRHPLVDEMFKRFTVDEDDEVATDIAWVLYDTAN    698
tr|A4I4C9|A4I4C9_LEIIN            RGQSMSLA---NQQMT--AERVLEVNYRHPLVDEMFKRFTVDEDDEVATDIAWVLYDTAN    698
sp|Q8T7E0|ENPL_LEIDO              RGQSMSLA---NQQLT--AERVLEVNYRHPLVDEMFKRFTVDEDDEVATDIAWVLYDTAN    698
tr|S9U4L8|S9U4L8_9TRYP            RSQMTGST---SSMLR--AQRVLEVNYRHPLVDELYRRFIVDPNDNIAEDIAWVLFDTAN    695
tr|A0A7G2C3S2|A0A7G2C3S2_9TRYP    KSQSLGRGATEDVPLT--AERVVEVNWRHPLVDEVYKRFLVDENDLVAEDIAWTLFDTAN    722
sp|P14625|ENPL_HUMAN              KAQAYQTGKDISTNYYASQKKTFEINPRHPLIRDMLRRIKEDEDDKTVLDLAVVLFETAT    722
tr|Q7T3L3|Q7T3L3_DANRE            KAQAYQTGKDISTNYYASQKKTLEINPKHPLIKEMLRRVNEDAEDKTAADLAVVLFETAT    722
sp|Q9STX5|ENPL_ARATH              QSQTLSDANKQAY-MR--GKRVLEINPRHPIIKELKDRIASDPEDESVKETAQLMYQTAL    745
tr|A0A061FKM2|A0A061FKM2_THECC    QSQTLSDASKQAY-MR--GKRVLEINPRHPIIKELRERVVKDPEDEGVKQTAQLIYQTAL    748
tr|Q5Z9N8|Q5Z9N8_ORYSJ            QSQTLSDASKQAY-MR--GKRVLEINPRHPIIKELRDKVAQDSESESLKQTAKLVYQTAL    746
tr|W2ZJ09|W2ZJ09_PHYPR            KAQTFGNGDKNSP-MYGTGSAILELNPRHPIVSKLNELMVSDPEKEETKDLAWLLYDTAL    758
tr|W2ZJ74|W2ZJ74_PHYPR            KAQTFGNGDKNSP-MYGTGSAILELNPRHPIVSKLNELMVSDPEKEETKDLAWLLYDTAL    757
tr|F0Y644|F0Y644_AURAN            RAQAFSEKQAMGQ-MS--SQKTMEINPRHPIIAKLKELVAAEKDDEYTSDLSWLLLDNAL    706
tr|D8LHY7|D8LHY7_ECTSI            KAQTLADSKQMGL-LG--GHRSMEINPRHPIVHELNKKIEEDPDSEETKDLSWLLYETAL    708
                                  . *                       .*:*   * : .:   .  : ..   : :  :  .*

tr|A0A0L1KBS8|A0A0L1KBS8_9EUGL    HQSAFDIDEVHNYAARMYAMIAQGLDVENHNTRLTEQASDYEAPQEDL------------    759
tr|A0A0L1KHT0|A0A0L1KHT0_9EUGL    LESGFEVEDLQSFSDRVLKVIYQGLDMGLDQAMLEEDLSQYQVESNSNADQHVEL-----    733
tr|A0A1X0P079|A0A1X0P079_9TRYP    LQAEFPITDQAMFAKRMNRLLRGRVGISTDDTLLPPDDDEYDISDVKPDTAATDENVLLP    724
tr|Q4DW89|Q4DW89_TRYCC            LQAGFPVSNQAMYAKRVNRLLRGRVGLAADDTILPPDDDEYDISDVKPDTTGTDEGLLLP    729
tr|A0A422PWD8|A0A422PWD8_9TRYP    LQGGFPVSNQAMYAKRVSRLLRGRVGLAADDTMLPPDDNEYDVSDVEPDTAGTDEGLLLP    726
tr|A0A422NRM6|A0A422NRM6_TRYRA    LQGDFPVSNQAMYAKRVNRLLRGRVGLAADDTMLPPDDNEYDVSDIKPSTSGTDEGLLLP    731
tr|A0A0S4KLS2|A0A0S4KLS2_BODSA    LQNGFDIEDTLAFSRRMSRLLRQSVDIPADAAMLTEDVSEYEIEDNEDAEDDE-------    751
tr|A0A0N0VF86|A0A0N0VF86_LEPPY    LQAEFPVADVAAYSRRINRLLRSSVDLAADETLLPPDDAEYAVSTTEAAEEEE-------    744
tr|A4HH83|A4HH83_LEIBR            LQAEFPVADVAAYAKRINRLLRSSVDLNADDSLLPPDDDEYTVFDTETEEEKP-------    751
tr|E9ADS8|E9ADS8_LEIMA            LQAEFPVADVAAYSKRINRLLRSSVDLSADDSLLPPDDAEYTVSDTEAEEEEE-------    751
sp|Q9NGD0|ENPL_LEIIN              LQAEFPVADVAAYSKRINRLLRSSVDLSADDSLLPPDDAEYTVSDTEAEEEEE-------    751
tr|A4I4C9|A4I4C9_LEIIN            LQAEFPVADVAAYSKRINRLLRSSVDLSADDSLLPPDDAEYTVSDTETEEEEE-------    751
sp|Q8T7E0|ENPL_LEIDO              LQAEFPVADVAAYSKRINRLLRSSVDLSADDSLLPPDDAEYTVSDTETEEEEE-------    751
tr|S9U4L8|S9U4L8_9TRYP            MQAEFPVLDVAAYSQRVVRLLATSVDLDPDQPLMAEDNGEAEEEETEDL-----------    744
tr|A0A7G2C3S2|A0A7G2C3S2_9TRYP    LQGDFPVVDTQAYAQRVTRLLSSSVDLDPTMGLLPDVDDDGLGDL---------------    767
sp|P14625|ENPL_HUMAN              LRSGYLLPDTKAYGDRIERMLRLSLNIDPDAKVEEEPEEEP----EETAEDTTEDTE-QD    777
tr|Q7T3L3|Q7T3L3_DANRE            LRSGYQLQDTKAYGERIERMLRLSMNVDLDAQVEEEPEEEP----EEQTEEAEDEEE-VQ    777
sp|Q9STX5|ENPL_ARATH              IESGFILTDPKDFAARIYNSVKSGLNISPDAVADEEIEAAE----EPETSEATETK----    797
tr|A0A061FKM2|A0A061FKM2_THECC    MESGFSLPDPKDFASRIYSSVKSSLNISPDATIEDDDDVEE----T-ETE--TETE----    797
tr|Q5Z9N8|Q5Z9N8_ORYSJ            MESGFNLPDPKDFASSIYRSVQKSLDLSPDAAVEEEEEVEE----A-EVE--EKES----    795
tr|W2ZJ09|W2ZJ09_PHYPR            INSGFDMTDTTQFSTRVHRIMKSSMGIDSLELEPEI-EVPE----EEQEVDEEEEA--ED    811
tr|W2ZJ74|W2ZJ74_PHYPR            INSGFDMTDTTQFSTRVHRIMKSSMGIDSLELEPEI-EVPE----EEQEVDEEEEA--ED    810
tr|F0Y644|F0Y644_AURAN            MQSGFEASDVAAFSERALRILKSGLSVESMDLLPEI-EVPD----EPEEEEEEEDA--VD    759
tr|D8LHY7|D8LHY7_ECTSI            TASGFQVEDTEAFANRVQRAMAKTLNLSSMDLLEEM-EIPD----EEEEEEGEGDEQFQD    763
                                           :   :   :.       :   :.:

tr|A0A0L1KBS8|A0A0L1KBS8_9EUGL    ------------------------------------------------------------    759
tr|A0A0L1KHT0|A0A0L1KHT0_9EUGL    ------------------------------------------------------------    733
tr|A0A1X0P079|A0A1X0P079_9TRYP    IDKETDEIPEAEKEGSTEGTKTAA-------EG--------------STEEKEKEKEKSK    763
tr|Q4DW89|Q4DW89_TRYCC            VDNDGDESSDKEDAEPTAAEM--------------------------------------K    751
tr|A0A422PWD8|A0A422PWD8_9TRYP    VDKEEGEATAAAAEEPTAAEAAEEPTAAAAAEEPTAAAAAEEPTAAAAAEEPKAAAAESK    786
tr|A0A422NRM6|A0A422NRM6_TRYRA    VDKDGEETSSEAAAAE-------Q-----------------------------PAAAESK    755
tr|A0A0S4KLS2|A0A0S4KLS2_BODSA    -------APKADADDDKEEL----------------------------------------    764
tr|A0A0N0VF86|A0A0N0VF86_LEPPY    -------GEAAAENANAAET----------------------------------------    757
tr|A4HH83|A4HH83_LEIBR            -------TNDAEYTVSDTATEEEKPTNDA----------------------------DAS    776
tr|E9ADS8|E9ADS8_LEIMA            -------QP----------------KVDA----------------------------NAD    760
sp|Q9NGD0|ENPL_LEIIN              -------QP----------------KVDA----------------------------NAD    760
tr|A4I4C9|A4I4C9_LEIIN            -------QP----------------KVDT----------------------------NAH    760
sp|Q8T7E0|ENPL_LEIDO              -------QP----------------KVDT----------------------------NAH    760
tr|S9U4L8|S9U4L8_9TRYP            ------------------------------------------------------------    744
tr|A0A7G2C3S2|A0A7G2C3S2_9TRYP    ------------------------------------------------------------    767
sp|P14625|ENPL_HUMAN              EDEEMDVGT-------DEEEETAKEST---------------------------------    797
tr|Q7T3L3|Q7T3L3_DANRE            ADEA----E-------EESEA---------------------------------------    787
sp|Q9STX5|ENPL_ARATH              ---SDDLAG--------GLNIEAEPVEQQE------------------------------    816
tr|A0A061FKM2|A0A061FKM2_THECC    ---TKEGAG--------SSKGEAEPANNDAD-----------------------------    817
tr|Q5Z9N8|Q5Z9N8_ORYSJ            ---S-------------NIKEEAEPSSYDKD-----------------------------    810
tr|W2ZJ09|W2ZJ09_PHYPR            LDESEAQAE--------ATEEADEPVVDGKD-----------------------------    834
tr|W2ZJ74|W2ZJ74_PHYPR            LDESEAQAE--------ATEEADEPVVDGKD-----------------------------    833
tr|F0Y644|F0Y644_AURAN            LDDFEDAGE--------GE-----------------------------------------    770
tr|D8LHY7|D8LHY7_ECTSI            LDEL--------------------------------------------------------    767


tr|A0A0L1KBS8|A0A0L1KBS8_9EUGL    -----------    759
tr|A0A0L1KHT0|A0A0L1KHT0_9EUGL    -----------    733
tr|A0A1X0P079|A0A1X0P079_9TRYP    EDAAAQDAGDL    774
tr|Q4DW89|Q4DW89_TRYCC            PTKTEDDAGDL    762
tr|A0A422PWD8|A0A422PWD8_9TRYP    PAAKDDGADDL    797
tr|A0A422NRM6|A0A422NRM6_TRYRA    PATKDDDVGDL    766
tr|A0A0S4KLS2|A0A0S4KLS2_BODSA    -----------    764
```

```
tr|A0A0N0VF86|A0A0N0VF86_LEPPY    ---DDDDEADL    765
tr|A4HH83|A4HH83_LEIBR            ETTGADSDGDL    787
tr|E9ADS8|E9ADS8_LEIMA            EKAEAVDEGDL    771
sp|Q9NGD0|ENPL_LEIIN              EEAEAVGEDDL    771
tr|A4I4C9|A4I4C9_LEIIN            EEAETDGEGDL    771
sp|Q8T7E0|ENPL_LEIDO              EEAETDGEGDL    771
tr|S9U4L8|S9U4L8_9TRYP            -----------    744
tr|A0A7G2C3S2|A0A7G2C3S2_9TRYP    -----------    767
sp|P14625|ENPL_HUMAN              -----AEKDEL    803
tr|Q7T3L3|Q7T3L3_DANRE            -----TSKDEL    793
sp|Q9STX5|ENPL_ARATH              ----ENTKDEL    823
tr|A0A061FKM2|A0A061FKM2_THECC    ----TDLKDEL    824
tr|Q5Z9N8|Q5Z9N8_ORYSJ            ----EL-----    812
tr|W2ZJ09|W2ZJ09_PHYPR            ----E-----L    836
tr|W2ZJ74|W2ZJ74_PHYPR            ----E-----L    835
tr|F0Y644|F0Y644_AURAN            ----------L    771
tr|D8LHY7|D8LHY7_ECTSI            -----------    767
```

(overall sequence identity = 0.1247)

14. Protein disulphide isomerases (Thioredoxins)

Protein disulfide isomerases (often consisting of multiple thioredoxin domains, arranged in tandem) regulate the oxidative crosslinking of cysteines and their correct pairing, that is absolutely necessary for luminal and extracellular protein domain functionality. Intracellular forms of thioredoxins also exist (without signal peptides) alongside with secreted ones. Although not all secreted thioredoxins have ER retention signals, those that do are often very highly conserved across many eukaryotic lineages, including kinetoplastids.

```
tr|W6KQG4|W6KQG4_9TRYP            ------------------MRSVLLF--FAVLIAL--------------------------T    15
tr|A0A0S4KL52|A0A0S4KL52_BODSA    ------------------MKAF-PLIALLAVAII--------------------------A    16
tr|A0A1X0NMX9|A0A1X0NMX9_9TRYP    -------------------MRLLLLIAF-LLSLL--------------------------R    15
tr|Q4E3F7|Q4E3F7_TRYCC            ------------------MIHFIFFVAL-FFCSL--------------------------R    16
tr|A0A3R7L858|A0A3R7L858_TRYRA    ------------------MMRFLLLATL-LLCSL--------------------------R    16
tr|A0A422Q717|A0A422Q717_9TRYP    ------------------MIRFLVFVTL-LLCSL--------------------------R    16
tr|A0A7G2CFK3|A0A7G2CFK3_9TRYP    ------------------MAKL-TAALLASLLLA--------------------------A    16
tr|S9V6G7|S9V6G7_9TRYP            ------------------MSKFFTLCILAVLLLS--------------------------T    17
tr|S9VLI6|S9VLI6_9TRYP            ------------------MSKFFTLCILAVLLLS--------------------------T    17
tr|A4HQL6|A4HQL6_LEIBR            ------------------MKLLYVVFFVCT-LLL--------------------------C    16
tr|A7YBW7|A7YBW7_LEIAM            ------------------MQRSFLAFVLCA-LLF--------------------------C    16
tr|Q4Q059|Q4Q059_LEIMA            ------------------MQRSFLVFVLCA-LLF--------------------------C    16
tr|Q8I8E1|Q8I8E1_LEIMA            ------------------MQRSFLVFVLCA-LLF--------------------------C    16
tr|A4ICD5|A4ICD5_LEIIN            ------------------MQRSFLAFVVCA-ILF--------------------------C    16
tr|B3VA16|B3VA16_LEIDO            ------------------MQRSFLAFVVCA-ILF--------------------------C    16
tr|A0A0M9G4E8|A0A0M9G4E8_LEPPY    ------------------MKQSFLLLAVCA-LFL--------------------------C    16
tr|A0A0N0P805|A0A0N0P805_LEPSE    ------------------MKQPLFLLALCA-LFL--------------------------C    16
tr|A0A7E6F1P9|A0A7E6F1P9_OCTVU    ---------------MALFSS-RLNYLLLLLLALISQVIDFRCYGEEADEADEATDDDNEP    44
tr|E3NBU5|E3NBU5_CAERE            -------------------------MSWIQAALVASFLAF--------------------A    16
tr|A0A4Z2C989|A0A4Z2C989_9TELE    ---------------------------MLKLMLLAALTGF--------------------T    14
tr|W5KW40|W5KW40_ASTMX            ------------------MAN-SVSGLLSGALFVCLWGGS--------------------V    22
tr|A0A0G4EK32|A0A0G4EK32_VITBC    ---------------MKLVIS-VLAICLAAPAVV--------------------------RA    20
tr|A0A058Z7G2|A0A058Z7G2_FONAL    ---------MAPA----TRSAFGLA-LMALLALT-------------------------AI    22
tr|G4MPX2|G4MPX2_MAGO7            ---------------MHTVRSFALGLLATAAVVS-------------------------AS    21
tr|A0A8J2IGP4|A0A8J2IGP4_FUSEQ    ---------MQHKKIACSFMA--------ALAAY-------------------------AS    19
tr|A0A8I3AHZ3|A0A8I3AHZ3_9PEZI    MGRSTPPPPQASGTRACLLASYRASSPSPSLIVH-------------------------HI    36


tr|W6KQG4|W6KQG4_9TRYP            RASADVVELTSKNFDEVIAKD-GITFIKFFTTWCGYCKKAAPEFANASDILKD---VATL    71
tr|A0A0S4KL52|A0A0S4KL52_BODSA    VVADDAIDAKDADFDEVVSRN-ALTLVKFYAPWCGHCKRIAPEWDKAATALVG---KAGL    72
tr|A0A1X0NMX9|A0A1X0NMX9_9TRYP    ADASDVVAATVDDFDSVIKKG-EIALVKFYAPWCGHCQKLAPEWEKAAKEVPG---EAIM    71
tr|Q4E3F7|Q4E3F7_TRYCC            AEGSEVVEATDKDFDDVISSG-EIALVKFYAPWCGHCQKLAPEWEKAAKEIPS---GAVM    72
tr|A0A3R7L858|A0A3R7L858_TRYRA    VEGSEVVAATDKNFDSVIARS-KVALVKFYAPWCGHCQKLAPEWEKAAKEIPS---EAIM    72
tr|A0A422Q717|A0A422Q717_9TRYP    VEGSEVVAATDKDFDSVLARG-EVALVKFYAPWCGHCQKLAPEWEKAAKEIPS---EAIM    72
tr|A0A7G2CFK3|A0A7G2CFK3_9TRYP    GCQGKVASLNEADFETGIKKT-EYTLVKFFAPWCGHCKKLAPEFEKASDALGD---KATL    72
tr|S9V6G7|S9V6G7_9TRYP            FACAEVITGTEKNLDDIIAKD-KVSIVKFYAPWCGHCKSLAPEFEKAADALKE---QASL    73
tr|S9VLI6|S9VLI6_9TRYP            FACAEVITGTEKNLDDIIAKD-KVSIVKFYAPWCGHCKSLAPEFEKAADALKE---QASL    73
tr|A4HQL6|A4HQL6_LEIBR            LTSAEVQVATQDNFDNVVSG--DLTLVKFYAPWCGHCKTLAPEFVKAAEMLTG---IATL    71
tr|A7YBW7|A7YBW7_LEIAM            VASAEVQVATKDNFDKIVSG--DLTLVKFYAPWCGHCKTLAPEFIKAAEMLAG---VATL    71
tr|Q4Q059|Q4Q059_LEIMA            VASAEVQVATKDNFDKVVSG--DLTLVKFYAPWCGHCKTLAPEFVKAADMLAG---IATL    71
tr|Q8I8E1|Q8I8E1_LEIMA            VASAEVQVATKDNFDKVVIG--DLTLVKFYAPWCGHCKTLAPEFVKAADMLAG---IATL    71
tr|A4ICD5|A4ICD5_LEIIN            VASAEVQVATKDNFDKIVSG--DLTLVKFYAPWCGHCKTLAPEFVKAADMLAG---IATL    71
tr|B3VA16|B3VA16_LEIDO            VASAEVQVATKDNFDKIVSG--DLTLVKFYAPWCGHCKTLAPEFIKAADMLAG---IATL    71
tr|A0A0M9G4E8|A0A0M9G4E8_LEPPY    VASAEVQVATKSNFDKIIGG--DLTLVKFYAPWCGHCKTLAPEFEKASVTLKG---VATL    71
tr|A0A0N0P805|A0A0N0P805_LEPSE    VASAEVQVATKSNFDEAISG--DLTLVKFYAPWCGHCKTLAPEFEKAAEALKG---VATL    71
tr|A0A7E6F1P9|A0A7E6F1P9_OCTVU    IEEDDVLVLNNNNFDDVIYSR-DTVLVEFYAPWCGHCKRLAPEYEKAAQKLAVNDPPIPL    103
```

```
tr|E3NBU5|E3NBU5_CAERE            SAGGAVLEYTDGNFDDLIQTH-DIALVKFYAPWCGHCKKIAPEYEKAAPKLASNDPPVAL    75
tr|A0A4Z2C989|A0A4Z2C989_9TELE    QAASDVLEFTDNDFESKIGDH-EIILVEFFAPWCGHCKKLAPEYEKAATALKG---VVPL    70
tr|W5KW40|W5KW40_ASTMX            SARSDVVELTDVDFDYVAAEH-ETMLVKFYAPWCGHCKKLAPEFETAAKRLKG---VVTL    78
tr|A0A0G4EK32|A0A0G4EK32_VITBC    EDEEAVVTLGTDNFTSFIEQN-PLSLVEFYAPWCGHCKRLAPEYEKAAKKLKESG-GPPL    78
tr|A0A058Z7G2|A0A058Z7G2_FONAL    AAASDVVDLNKDNFDSVVNAEGATSMVEFFAPWCGHCKQLAPKYEEAATKLKGI---ATI    79
tr|G4MPX2|G4MPX2_MAGO7            DAPSDVVQLKEDTFDAFIKEN-DLVLAEFFAPWCGHCKALAPHYEKAATSLKEKN--IKL    78
tr|A0A8J2IGP4|A0A8J2IGP4_FUSEQ    AADSDVKQLTKDTFDEFVKSN-DLVLAEFFAPWCGHCKALAPEYEEAATTLKEKN--IRL    76
tr|A0A8I3AHZ3|A0A8I3AHZ3_9PEZI    KGSKCTASLKKDTFDDFVKTN-DLVLAEFFAPWCGHCKALAPEYEEAATSLKEKN--IKL    93
                                           .       :        : :*:: ***:*:  **.:  *:  :        :

tr|W6KQG4|W6KQG4_9TRYP            AEVDCDKERNICKRFEVNGYPKFLLFRKG-VNISTFSGQRVASEMVDYVKVQMASDTNSV    130
tr|A0A0S4KL52|A0A0S4KL52_BODSA    VRVDCTTETAVAQKFGIQGYPTIKVFREG-QLVGDYDAGRTSEAIVQYVISNSGPAVTVV    131
tr|A0A1X0NMX9|A0A1X0NMX9_9TRYP    VDVDCTKETKLAEKYSIKGFPTIILFRNG-EEVETYKGARQSDAIVKYIKANVGPAAVYP    130
tr|Q4E3F7|Q4E3F7_TRYCC            VDVDCTKESNLAQKYSIKGFPTIILFRDG-KEVEHYKGGRKSSDIVNYVKANLGTAVVHV    131
tr|A0A3R7L858|A0A3R7L858_TRYRA    VDVDCTKEKNLAKKYSIQGFPTIILFREG-EEVEHYKGARKSTDLINYVKANVGPAAVYP    131
tr|A0A422Q717|A0A422Q717_9TRYP    VDVDCTKEKELAKKYSIQGFPTIILFREG-KEVEHYKGGRQSTDLVNYVKANVGPAVVYP    131
tr|A0A7G2CFK3|A0A7G2CFK3_9TRYP    AEVDCTQNEKLCGKFDVKGYPTLILFKNG-EKHLDFDGGRTAPEIISYVKSQYEPAVSVS    131
tr|S9V6G7|S9V6G7_9TRYP            VKVDCTVEKKLAEKYDIKGFPTLIVFRKG-EKVENYEGGRTAEEIISYVRSLSSPLFTPV    132
tr|S9VLI6|S9VLI6_9TRYP            VKVDCTVEKKLAEKYDIKGFPTLIVFRKG-EKVENYEGGRTAEEIISYVRSLSSPLFTPV    132
tr|A4HQL6|A4HQL6_LEIBR            AEVDCTTEKALAEKYEVKGFPTLYVFRNG-VKVKAYDGPRTADGIASYMKSHVGPSMKVV    130
tr|A7YBW7|A7YBW7_LEIAM            AEVDCTKEEALAEKYEVKGFPTLYLFRNG-EKVKDYDGPRTAAGIASYMKAQVGPSMKTI    130
tr|Q4Q059|Q4Q059_LEIMA            AEVDCTKEESLAEKYEIKGFPTLYIFRNG-EKVKIYDGPRTAAGIASYMKAHVGPSMKAI    130
tr|Q8I8E1|Q8I8E1_LEIMA            AEVDCTKEESLAEKYEIKGFPTLYIFRNG-EKVKIYDGPRTAAGIASYMKAHVGPSMKAI    130
tr|A4ICD5|A4ICD5_LEIIN            AEVDCTKEESLAEKYQIKGFPTLYIFRNG-EKVKDYDGPRTAAGIASYMKAHVGPSIKAI    130
tr|B3VA16|B3VA16_LEIDO            AEADCTKEESLAEKYQIKGFPTLYIFRNG-EKVKDYDGPRTAAGIASYMKAHVGPSIKAI    130
tr|A0A0M9G4E8|A0A0M9G4E8_LEPPY    AEVDCTKETELASKFDIKGFPTMMIFRNG-EKVEDYSGPRTAAGITAYIKAQVGPAVTAI    130
tr|A0A0N0P805|A0A0N0P805_LEPSE    VKVDCTKETELASKFGIKGFPTLIIFRNG-EKLEDYNGPRTAAGITAHMKAHVGPAVTTI    130
tr|A0A7E6F1P9|A0A7E6F1P9_OCTVU    AKIDATIATTSAMKYEVSGYPTLKIFRAG-KQ-FEYKGPREDYGIVNYMISQQGGASKLQ    161
tr|E3NBU5|E3NBU5_CAERE            VKVDCTTEKTVCDKFGVKGFPTLKIFRNG-VPAQDYDGPRDADGIVKFMRGQSGPSSKEL    134
tr|A0A4Z2C989|A0A4Z2C989_9TELE    AKVDCTSNSNICSKYQVSGYPTLKVFRDG-EESGAYDGPRTSDGIVSYFKKQVGPASVAL    129
tr|W5KW40|W5KW40_ASTMX            AKVDCTSSTEVCGRFGVTGYPTLKIFRNG-RESSSYDGPRSAVGIVDQMKKQAGPDSVPL    137
tr|A0A0G4EK32|A0A0G4EK32_VITBC    AKVDATQEKELAEKYEVRGYPTIKFFRNG--QAEEYDGGRTEPTIIEWIEKRTGPAVRVV    136
tr|A0A058Z7G2|A0A058Z7G2_FONAL    AKVDCTEQTELCQKHGIRGYPTLKVFRNKGDSVVDYSGAREVDALVSFMKKQVLPAVSTF    139
tr|G4MPX2|G4MPX2_MAGO7            IKVDCTEEQDLCQKHGVEGYPTLKVFRGL-DNVVPYKGQRQDDGIISYMVKQSLPAVSTI    137
tr|A0A8J2IGP4|A0A8J2IGP4_FUSEQ    AKIDCTEESDLCKDHGVEGYPTLKVFRGL-ENVTPYSGQRKAAGITSYMIKQSLPAVSLL    135
tr|A0A8I3AHZ3|A0A8I3AHZ3_9PEZI    AKIDCTEEADLCQTYGVEGYPTLKVFRGP-DNISPYSGQRKAAAITSYMIKQSLPAVSLL    152
                                           *.      .  . : *:*.: .*:            :.. *    :   .

tr|W6KQG4|W6KQG4_9TRYP            SSEEEFEKVKSENPRVCLIKTKSDETELAKLVNKLIPKYKATYRFVVINDEKVSPDDS--    188
tr|A0A0S4KL52|A0A0S4KL52_BODSA    SSADELEVLKKESPIVVVVFSAEEGTTSAVTHAEAAKTLRSKYRFVRVADATLFD-GA--    188
tr|A0A1X0NMX9|A0A1X0NMX9_9TRYP    TTAEEVEKLKEEHEILCVGLTSDAESPLAKELLSVAQRLRMKMKFAIVTKPEIVPGEK--    188
tr|Q4E3F7|Q4E3F7_TRYCC            ETAEELEKLREEHNAVCVGVTSDMESTLSKTLATSAEGLRMKMKFVVITDSNILPDEK--    189
tr|A0A3R7L858|A0A3R7L858_TRYRA    SSAEELAKLKEEHDAVCVGVTSDTESALSKALETSAKNLRMKLKFAVITDSKILPEEK--    189
tr|A0A422Q717|A0A422Q717_9TRYP    SSAEELAKLKEEHDAVCVGVASGTESALSTTLANSAQRLRMKLKFAVITDPEILPEEK--    189
tr|A0A7G2CFK3|A0A7G2CFK3_9TRYP    KTQADVDKLVKEGLPVVVIKTASETSQTFKTLEPIAKTRRLSNAFTLVSDAAVVKASP--    189
tr|S9V6G7|S9V6G7_9TRYP            KSQKELDELIALGLGVALVKTASEDGDVVTRLTPIAKDLSKVVAFAVGTDASIAADAP--    190
tr|S9VLI6|S9VLI6_9TRYP            KSQKELDELIALGLGVALVKTASEDGDVVTRLTPIAKDLSKVVAFAVGTDASIAADAP--    190
tr|A4HQL6|A4HQL6_LEIBR            AKAEELEDLMKENFPLCVVKTANADSEMASMMTKLADSLRSEMNFALVTDTAISPADA--    188
tr|A7YBW7|A7YBW7_LEIAM            AKAGELEDLKKEAFPVCVVKTASADSEMALMMTKVANFLRSQMNFVLVTDATISPDDG--    188
tr|Q4Q059|Q4Q059_LEIMA            STAEELEELKKETFPVCVVKTASTDSEMASMITKVADSLRSQMNFVLVTDAAISPNDA--    188
tr|Q8I8E1|Q8I8E1_LEIMA            STAEELEELKKETFPVCVVKTASTDSEMASMITKVADSLRSQMNFVLVTDAAISPNDA--    188
tr|A4ICD5|A4ICD5_LEIIN            SKAEELEELKKETFPLCVVKTASADSEMASMITKVADSLRSQMNFVLVTDAAISPNDA--    188
tr|B3VA16|B3VA16_LEIDO            SKAEELEELKKETFPVCVVKTASADSEMASMITKVADSLRTQMNFVFVTDAAISPNDA--    188
tr|A0A0M9G4E8|A0A0M9G4E8_LEPPY    EKAEQLDELKKEDLPVCVVKTASADSALATTMTKVANVFRTQVNFALVTDEAIAADAA--    188
tr|A0A0N0P805|A0A0N0P805_LEPSE    EKAEQLEEIKKGDLPVCIIKTADAKSALVETMTKVANSLRTQMNFVLITDAAISADDT--    188
tr|A0A7E6F1P9|A0A7E6F1P9_OCTVU    ASAKELQKYMKEDDITIVGFFNSLDAPLMSSYLDAANEVRDDYKFAHVLDKDVTKSYDLV    221
tr|E3NBU5|E3NBU5_CAERE            KTVAEFEKFTGGDENVVVGFFESE-SKLKDSFLKVADTERDRFAFAHTSNKDIIKKAG-Y    192
tr|A0A4Z2C989|A0A4Z2C989_9TELE    AGEEELQKFISEKDSSVVGFFADDKSTAQAEFLKAASALRDKYRFAHTNAEVLLKSQNVG    189
tr|W5KW40|W5KW40_ASTMX            HNEKDLETFVNHYDASVVGVFESSDSPQLVEFLKGANLMRESFRFAHTTDLQLGLKYNLT    197
tr|A0A0G4EK32|A0A0G4EK32_VITBC    TG-EELEKLKKDKDILMVAFVKDQEAELFKTYEKVADKNRDLALFIAVVSGDA-------    188
tr|A0A058Z7G2|A0A058Z7G2_FONAL    EKADELTKFIEDDRVVVVGFFEEGDA-KAANFKNVAEKLREKMSFASTTSADLAKAHGVT    198
tr|G4MPX2|G4MPX2_MAGO7            TT-DSLEEFKKTDKVVVVAYLSSDDKTSTETFTQAAEKLRDHYPFGSSTDAALAEAEGVK    196
tr|A0A8J2IGP4|A0A8J2IGP4_FUSEQ    TK-DTLEEFKTADKVVVVAYLNADDKSSNETFSKLAEGLRDTYLFGGVNDAAVAEAEGVK    194
tr|A0A8I3AHZ3|A0A8I3AHZ3_9PEZI    TK-DTLEEFKTADKVVLVAYIAADDKASNETFNKVAESLRDNYLFGGVNDAAVAEAEGVK    211
                                           .       :                         *

tr|W6KQG4|W6KQG4_9TRYP            MESITVYR------KGDDKE--VFDGNLS---SEALSTFLDKAPVMFIGTLSPESIPFYS    237
tr|A0A0S4KL52|A0A0S4KL52_BODSA    SDSIVIYK------QFDEGRVPYTGDVSK---VADLTAFLSTTSVRVFDEIGPENYKMYM    239
tr|A0A1X0NMX9|A0A1X0NMX9_9TRYP    AETVVVYR------KDDEKE--VYQGNME---ADNLKNFLETAVVPFAGEIGPANYLRYA    237
tr|Q4E3F7|Q4E3F7_TRYCC            PESIIVFR------KGGEKE--VFDGAME---TADLKSFLEVAFIPFMGEINPNTYLDYA    238
tr|A0A3R7L858|A0A3R7L858_TRYRA    LESIVVFR------KGGEKE--IYEGAME---VKELNSFLAVSFLPFAGEINPKTYMNYA    238
tr|A0A422Q717|A0A422Q717_9TRYP    PESIIVFR------KGGEKE--VYEGAME---AEELNSFLEVSVLPFAGEIGPRTYMNYA    238
tr|A0A7G2CFK3|A0A7G2CFK3_9TRYP    MESITFLA------NGESAVLEIKDGVS----EKDINKFLDLNSVPLFGTITPENFKNYM    239
tr|S9V6G7|S9V6G7_9TRYP            MESIVFYS------GGKVHK--YTDDLLSSDFEDKFTKFLNFAKVATFGEVTPSSFKNYL    242
tr|S9VLI6|S9VLI6_9TRYP            MESIVFYS------GGKVHK--YTDDLLSSDFEDKFTKFLNFAKVATFGEVTPSSFKNYL    242
tr|A4HQL6|A4HQL6_LEIBR            MESVTVYR------KGKERE--AYDSASPMT-DASMKNFLATAVLDFFGELGQMSFQKYM    239
tr|A7YBW7|A7YBW7_LEIAM            MESVTVYR------QNMERE--AYTGATPIT-MESVNSFLATATLDFFGELGQQSFQKYM    239
tr|Q4Q059|Q4Q059_LEIMA            MESVTVYR------KNAERE--AYTGATPMT-AESVKSFLTSAVLDYFGELGQESFQKYM    239
tr|Q8I8E1|Q8I8E1_LEIMA            MESVTVYR------KNAERE--AYTGATPMT-AESVKSFLTSAVLDYFGELGQESFQKYM    239
tr|A4ICD5|A4ICD5_LEIIN            MESVTVYR------KNAERE--AYTGAAPMT-AESVKRFLATAVLDYFGELGQESFQKYM    239
tr|B3VA16|B3VA16_LEIDO            MESVTVYR------KNAERE--AYTGAAPMT-AESVKRFLATAVLDYFGELGQESFQKYM    239
tr|A0A0M9G4E8|A0A0M9G4E8_LEPPY    MESVTVYR------HDKEHE--AYAGVLPVT-VESAKQFLSEAQLDFLGELGQETFQSYM    239
tr|A0A0N0P805|A0A0N0P805_LEPSE    MESVTVYR------HGTERE--AYSGELPMT-VESAKQFLTEAMIDSFGELGEGSFMNYM    239
tr|A0A7E6F1P9|A0A7E6F1P9_OCTVU    ANSIVVFVPERFQTEYEPKKHVLNKDDAQ---RSDILSFIEKNQFPLVGHYNFNNEKKYN    278
tr|E3NBU5|E3NBU5_CAERE            SDDIAVFVPKKLHNKFDSNEFKY-DGNYD---TDKIKNFLTHETVGLAGIRTQGNLFQFE    248
tr|A0A4Z2C989|A0A4Z2C989_9TELE    PEGIVLFRPPTLKNKFEDSSVVKYSEEKYT---SNKIKRFIQDNVLGFCPHMTEDNKDQLM    246
tr|W5KW40|W5KW40_ASTMX            SEGILLFRPPRLASKFEESVVSH-TGPFS---VTALRRFIRDNIFGICPHLTKENIDQLR    253
```

```
tr|A0A0G4EK32|A0A0G4EK32_VITBC    KDEVIAYR------T-DEDPTTCT--GKS---ADEIEAFVKDESFPLFGAISGENYSKYV    236
tr|A0A058Z7G2|A0A058Z7G2_FONAL    APAVVLFK------KFDEGKNILQGSDFS---VDTVQKFVSDNSVALMEDLNQSNYQHFA    249
tr|G4MPX2|G4MPX2_MAGO7            APAIVVYK------TFDEGKAVFD-KKFD---VEEIEKFAKTAATPLIGEVGPETYSDYM    246
tr|A0A8J2IGP4|A0A8J2IGP4_FUSEQ    APALVVYK------AFDERKNTFT-EKFE---EEAISTFISTSATPLIGEVGPETYAGYM    244
tr|A0A8I3AHZ3|A0A8I3AHZ3_9PEZI    APAVVLYK------SFDEGKTTFS-DKFE---VEAIEKFAKTSATPLIGEVGPDTYSGYM    261
                                           :                                 *               .

tr|W6KQG4|W6KQG4_9TRYP            -KLSH--EKIGILLMGSE-----TTDERIADLRLVAKKYRN---KITVISFEGESNE-VS    285
tr|A0A0S4KL52|A0A0S4KL52_BODSA    -ERG---LPIAWLFVKPSAD---NFEALKTQVSAVASAHQG---QLSVVWVDADKYGAMA    289
tr|A0A1X0NMX9|A0A1X0NMX9_9TRYP    -ELS---KPVGWVLLKPKEE---LSKELQPKLLEVGKKMRQ---HLAILWVDAEQYP-VW    286
tr|Q4E3F7|Q4E3F7_TRYCC            -GIS---GPVAWVLLKPSEE---ESKELKSKLLDVGKKMRR---LMVLLWVDAEQYG-VA    287
tr|A0A3R7L858|A0A3R7L858_TRYRA    -AIS---FPVGWVLLKPSGE---ESQELKPKLVDLGRRMRR---QVVLLWADADQYG-VA    287
tr|A0A422Q717|A0A422Q717_9TRYP    -GLS---TPVGWVLLKPSGE---ASQELKPKLVELGRKMRR---HVVLLWVDAEQYG-VA    287
tr|A0A7G2CFK3|A0A7G2CFK3_9TRYP    -AAGEHGYAIGFLFTKDE-----KESPETKAAAAVAKQYRDN--KVILVTVNGALYGAFG    291
tr|S9V6G7|S9V6G7_9TRYP            -DISNMKFPLGYLFVTTL-----VCTE-AKAVKAIAEKMRP---RIIFASVDSGLYGGFG    292
tr|S9VLI6|S9VLI6_9TRYP            -DISNMKFPLGYLFVTTL-----VCTE-AKAVKAIAEKMRP---RIIFASVDSGLYGGFG    292
tr|A4HQL6|A4HQL6_LEIBR            -EAN-KEKPLGWVFIDKN-----TDPTLKKSLEAVAEKYRS---QVLMTYIDGDQYRPVS    289
tr|A7YBW7|A7YBW7_LEIAM            -EVN-KDKPLGWVFIDKN-----TDPALKGSLVAVAEKYRS---QVLLTYIDGDQYRPVS    289
tr|Q4Q059|Q4Q059_LEIMA            -EAN-KDKPLGWVFIDKN-----TDSALKGSLVAVAEKYRS---QVLLTYIDGDQYRPVS    289
tr|Q8I8E1|Q8I8E1_LEIMA            -EAN-KDKPLGWVFIDKN-----TDSALKGSLVAVAEKYRS---QVLLTYIDGDQYRPVS    289
tr|A4ICD5|A4ICD5_LEIIN            -EAN-KGKPLGWVFVDKN-----TDPALKGSLVAVAEKYRS---QVLLTFIDGDQYRPVS    289
tr|B3VA16|B3VA16_LEIDO            -EAN-KGKPLGWVFVDKN-----TDPALKGSLVAVAEKYRS---QVLLTFIDGDQYRPVS    289
tr|A0A0M9G4E8|A0A0M9G4E8_LEPPY    -EAN-KAKPLGWLFVDKD-----TAPALKESLVAVAEKFRS---QVLMSWVDGDKYRQVA    289
tr|A0A0N0P805|A0A0N0P805_LEPSE    -EAN-KVKPLGWFFLDKD-----TAPALRKSIAAVAEKYRS---QVLMSWINGDKFRQVS    289
tr|A0A7E6F1P9|A0A7E6F1P9_OCTVU    TKYP---LCLVFYTVDWSFDYRKATQIWRKKIAKIARNYKS----ITFAVANEDDHSNLM    331
tr|E3NBU5|E3NBU5_CAERE            -QKP---IVVVYYNVDYVKD-PKGSNYWRNRVLKVAQNYKR---KVQFAVSNKEEFSSEI    300
tr|A0A4Z2C989|A0A4Z2C989_9TELE    -GKD---LLVAYYDVDYERN-PKGSNYWRNRVMKVAKTFLDEGKKLNFAVANKARFMSVL    301
tr|W5KW40|W5KW40_ASTMX            -KRD---LLTAYYDLDYVHN-PKGSNYWRNRVMKVASQYSSRGL--LFSVANRKDFEDEL    306
tr|A0A0G4EK32|A0A0G4EK32_VITBC    -SRS---QDLVWACMSPD-DAK----KHGDAMRSAASKFRQ---SYSMVHLNTEEFGSHA    284
tr|A0A058Z7G2|A0A058Z7G2_FONAL    -SSP---LPMAVLFVTTDEHRA----TLGKGVEAIAKELKG---KMNFVYLDATKYGAFA    298
tr|G4MPX2|G4MPX2_MAGO7            -SAG---IPLAYIFAETAEERT----TLSEALKSIAEKHRG---AINFATIDAKAFGAHA    295
tr|A0A8J2IGP4|A0A8J2IGP4_FUSEQ    -SAG---IPLAYIFSETEEERK----ELGDALKPIAEKYKG---KINFATIDAKAFGAHA    293
tr|A0A8I3AHZ3|A0A8I3AHZ3_9PEZI    -EAG---LPLAYIFAETAEERE----ELSKALKPIAEKQRG---VINFATIDAKSFGAHA    310
                                                                    .        .   :

tr|W6KQG4|W6KQG4_9TRYP            -KNIGL--PEDTKYPAFVLTHD--NTAYPHPTDIQANSSTIDAFMQKYLNGEVKPIQKSQ    340
tr|A0A0S4KL52|A0A0S4KL52_BODSA    -ERLGV--RKGA-FPAFVVDRS--GEHFVLAEDKEISTAIVGDFVTAVLADTLKPTIRST    343
tr|A0A1X0NMX9|A0A1X0NMX9_9TRYP    -KNLGV--SEDAKYPAFVIAKG--DKHHVLPTSEPATVESIEEFIVKYAEGKTDAELKSQ    341
tr|Q4E3F7|Q4E3F7_TRYCC            -SSLGL--SDDAKYPAFVIARG--EDHFVHPSTEPVTAESIEKFIIEYSEKKLSPEIKSQ    342
tr|A0A3R7L858|A0A3R7L858_TRYRA    -SSLGL--PEDAKYPAFAIVQG--EDHFVHPSAEPVTAESIEKFIVGFSEGKITKEIKSQ    342
tr|A0A422Q717|A0A422Q717_9TRYP    -PSLDL--PEDAKYPAFVIARG--EDHFVHPSTEPVTAEGIEKFIVEVSEGKVAKELKSQ    342
tr|A0A7G2CFK3|A0A7G2CFK3_9TRYP    -DQLGA--PKE--YPSLVIDKS--RTKFLFPPAQKMTEEELKSFLDQFTNGKLAPHLKSE    344
tr|S9V6G7|S9V6G7_9TRYP            -EQLSL--PKDTK-TGFVIEWD--HKHYVFPLDKKIESKALEEFITAVLDKKISPTLKSE    346
tr|S9VLI6|S9VLI6_9TRYP            -EQLSL--PKDTK-TGFVIEWD--HKHYVFPLDKKIESKALEEFITAVLDKKISPTLKSE    346
tr|A4HQL6|A4HQL6_LEIBR            -RQLGI--PEGAEFPAFVIDHD--RRHHVMPVDIPVTVESITEFIEKYIKGETKQTLMSD    344
tr|A7YBW7|A7YBW7_LEIAM            -RQLGI--PEDAKFPAFVVDYE--HRHHVMDAATPVTSESVAAFVEKYIKGETQQTVMSD    344
tr|Q4Q059|Q4Q059_LEIMA            -RQLGI--PEDAKFPAFVVDFE--RRHHVMGTDTPVTSESVAAFVEKYVKGETKQTVMSD    344
tr|Q8I8E1|Q8I8E1_LEIMA            -RQLGI--PEDAKFPAFVVDFE--RRHHVMGTDTPVTSESVAAFVEKYVKGETKQTVMSD    344
tr|A4ICD5|A4ICD5_LEIIN            -RQLGI--PEDAKFPAFVVDFE--RRHHVMETYTPVTAESVAAFVEKYIKGETKQTLMSD    344
tr|B3VA16|B3VA16_LEIDO            -RQLGI--PEDAKFPAFVVDFE--RRHHVMETYTPVTAESVAAFVEKYIKGETKQTLMSD    344
tr|A0A0M9G4E8|A0A0M9G4E8_LEPPY    -FQLGM--PKDVKFPAFVLDFE--RHHHVMPTETPITAESVSEFVEKYSKGETAETLMSE    344
tr|A0A0N0P805|A0A0N0P805_LEPSE    -LQLGV--PKDVKFPFVLVIDFE--RRHHLMPIDIPITAESVSEFVGKYIKGETTQTMMSE    344
tr|A0A7E6F1P9|A0A7E6F1P9_OCTVU    -KDFGFEDSGE-DMN-IGIMT--EEKRYTMEPMDEFESSHIESFLDDFVSGNLKPHIKSQ    386
tr|E3NBU5|E3NBU5_CAERE            -ETNGLGERKDSDKPIVAMLTN-EG-KFPMDQEFSV--ENLQQFVDEVLAGNSEPYMKSE    355
tr|A0A4Z2C989|A0A4Z2C989_9TELE    -SEFGLED-HSSAGPLVTIRTA-KGEKYAMTEEFLPDGKALERFLLSYFDGSLKPYLKSE    358
tr|W5KW40|W5KW40_ASTMX            EEDYGLGSTEGSEVPFVTIRTR-LGHKYSMREEFTRDGKSLERFLEDYYANSLKRYIKSE    365
tr|A0A0G4EK32|A0A0G4EK32_VITBC    -ENA-L---GVSEFPAVVVQKK--SGRYVYPTELAIEAEKLVSFLEDVSAGKVEKAIKSE    337
tr|A0A058Z7G2|A0A058Z7G2_FONAL    -EALNL---KQ-EFPAFAIQGQ-KGAKFPLSQDAAITAEAIGKLAKGMLDGSVTPTLKSE    352
tr|G4MPX2|G4MPX2_MAGO7            -GNLNL---KADKFPAFAIQETVKNQKFPFDQDKEITAEAISKFVEDFVAGKIEPSVKSE    351
tr|A0A8J2IGP4|A0A8J2IGP4_FUSEQ    -GNLNL---KTDKFPSFAIQEVVKNQKFPFDQEKEITHDSIAKFVEEFDAGKIEPSIKSE    349
tr|A0A8I3AHZ3|A0A8I3AHZ3_9PEZI    -GNLNL---QADKFPAFAIQETVKNQKFPFDQEKKITVEAITSFVDDFVAGKIEPSIKSE    366
                                                   . :      .                :  :           *

tr|W6KQG4|W6KQG4_9TRYP            PIPEEPTKDGLTILVGKTIDSY-IKKGKNLMIFFNAPWCGHCNKLHPIYDEFAKSHG---    396
tr|A0A0S4KL52|A0A0S4KL52_BODSA    EAPEEHTKDGLTTVVGSTFDDLVINSGKDVLIEFYAPWCGHCKKLQPTFEQVAKELK---    400
tr|A0A1X0NMX9|A0A1X0NMX9_9TRYP    PVPEVETVDGLTTIVGKTMNKY-LSSGKDILIEFFAPWCGHCKNFAGTYAKVAKEFE---    397
tr|Q4E3F7|Q4E3F7_TRYCC            PVPEIETVEGLTTVVGKTLDKY-LSSGKDMLIEFFAPWCGHCKNLAPIYAKVAKEFE---    398
tr|A0A3R7L858|A0A3R7L858_TRYRA    PIPEVETVEGLTTVVAKTLDKH-LSSGKDILIEFFAPWCGHCKNLAPTYAKLAKELE---    398
tr|A0A422Q717|A0A422Q717_9TRYP    PVPEVETVEGLTTVVAKTLDKH-LSSGKDMLIEFFAPWCGHCKNLAPTYAKLAKEFE---    398
tr|A0A7G2CFK3|A0A7G2CFK3_9TRYP    AIPEKETVDGLTTLVGKSFESH-LNKGKDMFVLFYAPWCGHCKKLHPDYEKMAKELE---    400
tr|S9V6G7|S9V6G7_9TRYP            EAPKTETTNGLTTLVGTTFAKY-VQEKKPMFVLFYAPWCGHCKKLHPVFDKLAEAFE---    402
tr|S9VLI6|S9VLI6_9TRYP            EAPKTETTNGLTTLVGTTFAKY-VQEKKPMFVLFYAPWCGHCKKLHPVFDKLAEAFE---    402
tr|A4HQL6|A4HQL6_LEIBR            EVPATETVNGLTTVVGQTISKY-TDGKQNVMLLFYAPWCGHCQKLHPDYEKMAENLQ---    400
tr|A7YBW7|A7YBW7_LEIAM            AIPAKETVNGLTTVVGHTFSKY-TDSTQNVMLLFYAPWCGHCQKLHPAYEKVAKSFE---    400
tr|Q4Q059|Q4Q059_LEIMA            AIPAKETVNGLTTVVGQTFAKY-TDGTQNVMLLFYAPWCGHCKKLHPVYDKVAKSFE---    400
tr|Q8I8E1|Q8I8E1_LEIMA            AIPAKETVNGLTTVVGQTFAKY-TDGTQNVMLLFYAPWCGHCKKLHPVYDKVAKSFE---    400
tr|A4ICD5|A4ICD5_LEIIN            AIPAKETVNGLTTVVGQTFAKY-TDGTQNVMLLFYAPWCGHCQKLHPVYEKVAKSLE---    400
tr|B3VA16|B3VA16_LEIDO            AIPAKETVNGLTTVVGQTFAKY-TDGTQNVMLLFYAPWCGHCQKLHPVYEKVAKSLE---    400
tr|A0A0M9G4E8|A0A0M9G4E8_LEPPY    SVPEVETVEGLTTIVGKTVDKY-TDGSKNIFVLFYAPWCGHCKKLHPEFEKLAKELE---    400
tr|A0A0N0P805|A0A0N0P805_LEPSE    SVPDVETVDGLTTIVGHTIAKY-TDGSKNAFVMFYAPWCGHCKKLHPDFEKLAKELE---    400
tr|A0A7E6F1P9|A0A7E6F1P9_OCTVU    PIPKKDK-GPVVTVVANNFDSVVLDKSKDVLIEFYAPWCGHCQKLEPIYKKLAKSLQ---    442
tr|E3NBU5|E3NBU5_CAERE            PIPEE-Q-GDVKVAVGKNFKQLIMDSDKDVLIEFYAPWCGHCKSLAPKYDELAEKLN---    410
tr|A0A4Z2C989|A0A4Z2C989_9TELE    PIPENND-GPVKVVVAENFDSIVNDDSKDVLIEFYAPWCGHCKSLEPKYKELGEKLA---    414
tr|W5KW40|W5KW40_ASTMX            PVPAKNN-GPVKVVVADTFEEIVNDPEKDVLIEFYAPWCGHCKKLEPKYTELGQELS---    421
tr|A0A0G4EK32|A0A0G4EK32_VITBC    AIPEKND-EAVKVVVAKNFEEMVLRDDKDVMLEVYAPWCGHCKKLEPVYKEFAEKLQKA-    395
tr|A0A058Z7G2|A0A058Z7G2_FONAL    PIPEKQE-GPITVVVGKTYDSIVNEKGKDVLVQFYAPWCGHCKNLIPIYDELAGDLK---    408
tr|G4MPX2|G4MPX2_MAGO7            PIPETND-GPVSVVVAHTYNDIVLDDTKDVLIEFYAPWCGHCKALAPKYEELGALYQKSE    410
```

```
tr|A0A8J2IGP4|A0A8J2IGP4_FUSEQ     PIPETQE-GPVTVVVAKSYNDIVLDDTKDVLIEFYAPWCGHCKALAPKYDDLASQYAASE    408
tr|A0A8I3AHZ3|A0A8I3AHZ3_9PEZI     PIPETQE-GPVTVVVAKNYEQIVLDDTKDVLIEFYAPWCGHCKALAPKYEELATLYGESE    425
                                   *        :   *. .  .      :  :: . *******: :   : ...

tr|W6KQG4|W6KQG4_9TRYP             SDN-LIISKIDGTANDFNR-TMFNVNGFPTIYFIPAGSQ-EAMFYNGKRTVEDLAAFVKK    453
tr|A0A0S4KL52|A0A0S4KL52_BODSA     DVDGIRIAQIDAGENDFNT-KLFTVSGFPTLYFVPANGS--PKLFEGSRSVMGILNFLKE    457
tr|A0A1X0NMX9|A0A1X0NMX9_9TRYP     SSD-VIIAAIDATANDVDR-SLFDVTGFPTLYFVPSGGK--PILYNGDRTFYDLYKFIRD    453
tr|Q4E3F7|Q4E3F7_TRYCC             SSD-VIIAAMDATANQMDN-SLFDVSGFPTIYFVPHGGK--PIMYDGGRTFYEIYKFVHE    454
tr|A0A3R7L858|A0A3R7L858_TRYRA     SSN-VIIAAMDATANHVNQ-SVFEVSGFPTIYFVPHGKK--PIIYEGERSFDDMYKFVRE    454
tr|A0A422Q717|A0A422Q717_9TRYP     SSD-VIIAAMDATANHVDQ-SVFAVSGFPTIYFVPHGKP--PISYEGDRTFNDMYEFVRE    454
tr|A0A7G2CFK3|A0A7G2CFK3_9TRYP     KED-VVIAKIDATANDVDP-SKYKVQGFPTIFFIPSGGA--PVSYNGDRSIDDMKKFIKE    456
tr|S9V6G7|S9V6G7_9TRYP             KED-VLIAKLDATENDVDR-ELFEVKGFPTLYFIKNG-A--GISYSGDRTLEDMTKFVRS    457
tr|S9VLI6|S9VLI6_9TRYP             KED-VLIAKLDATENDVDR-ELFEVKGFPTLYFIKNG-A--GISYSGDRTLEDMTKFVRS    457
tr|A4HQL6|A4HQL6_LEIBR             SEN-VMIAKMDATTNDFDR-EKFQVSGFPTIYFIPAGKP--PMMYEGGRSAKEMEEFVRS    456
tr|A7YBW7|A7YBW7_LEIAM             SEN-VIIAKMDATTNDFDR-KKFDVSGFPTIYFIPAGKP--PVAYEGDRTADDMMAFVKS    456
tr|Q4Q059|Q4Q059_LEIMA             SEN-VIIAKMDATTNDFDR-EKFEVSGFPTIYFIPAGKP--PIVYEGGRTADEIQVFVKS    456
tr|Q8I8E1|Q8I8E1_LEIMA             SEN-VIIAKMDATTNDFDR-EKFEVSGFPTIYFIPAGKP--PIVYEGGRTADEIQVFVKS    456
tr|A4ICD5|A4ICD5_LEIIN             SEN-VIIAKMDATTNDFDR-EKFEVSGFPTIYFIPAGKP--PIVYEGGRTADDIKAFVKS    456
tr|B3VA16|B3VA16_LEIDO             SEN-VIIAKMDATTNDFDR-EKFEVSGFPTIYFIPAGKP--PIVYEGGRTADDIKAFVKS    456
tr|A0A0M9G4E8|A0A0M9G4E8_LEPPY     SAD-VIIGKIDATANDFDR-AKFSVNGFPTLFFIPAGGK--TESYEGGRSLAEMKAFVVS    456
tr|A0A0N0P805|A0A0N0P805_LEPSE     AVN-VVIGKIDATTNDFDR-EKFVVNGFPTLYFIPAGGE--PLAYEGGRSTADMKAYVLS    456
tr|A0A7E6F1P9|A0A7E6F1P9_OCTVU     KNQNLVIAKMDATANDVPS--NFKTEGFPTIYFAPTNNKENPVKFTGGRELKDFVKFLEE    500
tr|E3NBU5|E3NBU5_CAERE             KE-DVIIAKMDATANDVPP--LFEVRGFPTLFWLPKNSKSNPIPYNGGREVKDFVNFISK    467
tr|A0A4Z2C989|A0A4Z2C989_9TELE     DDPNIVIAKMDATANDVPS--PYEVSGFPTLYFSPAGQKRNPKKYEGGREVSDFLSYLKR    472
tr|W5KW40|W5KW40_ASTMX             SDPNIVIAKMDATANDVPE--GYDVHGFPTIYFVPAGRKAEPKRYEGAREVKDFLNFLKR    479
tr|A0A0G4EK32|A0A0G4EK32_VITBC     SIDHIVVAKMDGTANESPV-EGFDWSGFPTIFWIPAGAK-GPSKYNGARTTEGLLKWVKE    453
tr|A0A058Z7G2|A0A058Z7G2_FONAL     SNSNIVIAKMDATANDLPLDAGFEIHGFPTIKLVTADGK--VVDFNGERTVEGFKKFIEA    466
tr|G4MPX2|G4MPX2_MAGO7             FKDKVVIAKVDATANDVPD----EIQGFPTIKLYAAGKKDSPATYSGSRTIEDLITFVKE    466
tr|A0A8J2IGP4|A0A8J2IGP4_FUSEQ     FKDKVVIAKVDATLNDVPD----EIQGFPTIKLYPAGGKDAPVTYQGSRTVEDLANFVKE    464
tr|A0A8I3AHZ3|A0A8I3AHZ3_9PEZI     FKDKVVIAKVDATLNDVPD----EIQGFPTIKLYPAGGKSEPVTYSGSRTIEDLIEFVRD    481
                                             : :. :*.  *.              ****:    .        : * *    :  ::

tr|W6KQG4|W6KQG4_9TRYP             HMKEPVEPKQED----------------------------LEGGDL    471
tr|A0A0S4KL52|A0A0S4KL52_BODSA     ASSVTFELPSEDR-------------------------------AEL    473
tr|A0A1X0NMX9|A0A1X0NMX9_9TRYP     HSSSFKDDKGASVPKE-----------EKK-----EEVRSDEDRGDL    484
tr|Q4E3F7|Q4E3F7_TRYCC             HSSTLKDVP---IPEE-----------VKR-----EE-EKNGDDDDL    481
tr|A0A3R7L858|A0A3R7L858_TRYRA     HSSALKDAP---IPEK-----------IKN-----ENKEKNGDEGDL    482
tr|A0A422Q717|A0A422Q717_9TRYP     HSTALKDTP---VSEE-----------VRN-----KEEDENGQEGDL    482
tr|A0A7G2CFK3|A0A7G2CFK3_9TRYP     HSSKKGGDAKKA--------------------------DAGDNADL    476
tr|S9V6G7|S9V6G7_9TRYP             HMEAPSSDDSV----------------------------------DL    470
tr|S9VLI6|S9VLI6_9TRYP             HMEAPSSDDSA----------------------------------DL    470
tr|A4HQL6|A4HQL6_LEIBR             HMTTSSG-----S----------------S------------DEGDL    470
tr|A7YBW7|A7YBW7_LEIAM             HLTASAG-----P------------------------SDKSDEEDL    473
tr|Q4Q059|Q4Q059_LEIMA             HLTASAA-----P----------------S-----GGPSGNSEEEDL    477
tr|Q8I8E1|Q8I8E1_LEIMA             HLTASAA-----P----------------S-----GGPSGNSEEEDL    477
tr|A4ICD5|A4ICD5_LEIIN             HLTASAA-----P----------------S-----GGPSGNSDEEDL    477
tr|B3VA16|B3VA16_LEIDO             HLTASAA-----P----------------S-----GGPSGNSEEEDL    477
tr|A0A0M9G4E8|A0A0M9G4E8_LEPPY     HMASTPEATPSSP----------------S-----AAPTDEND-DDL    481
tr|A0A0N0P805|A0A0N0P805_LEPSE     HITEAPELTSS------------------A-----AAPTDDQD-KDL    479
tr|A0A7E6F1P9|A0A7E6F1P9_OCTVU     HSTVSLNTKHI---------------------------------EL    513
tr|E3NBU5|E3NBU5_CAERE             HSTDGLKGFNRDG------------KKK--------------KHTEL    488
tr|A0A4Z2C989|A0A4Z2C989_9TELE     EATNAPVVQEETK-----------KKKK--------------KRVEL    494
tr|W5KW40|W5KW40_ASTMX             EATSSLVLKGVKE-----------DL---------------------    494
tr|A0A0G4EK32|A0A0G4EK32_VITBC     KASKPVVHDEL------------------------------------    464
tr|A0A058Z7G2|A0A058Z7G2_FONAL     NGTAPKPAAAAAAAE-------EEKKEEKKDEKKDEEKKDHHAHGDL    506
tr|G4MPX2|G4MPX2_MAGO7             NGKYKAEVSVEAEKETPVAPAATESEEAKATEAAEKK---KDEHDEL    510
tr|A0A8J2IGP4|A0A8J2IGP4_FUSEQ     NGKYKAEISVKEEGTEEAAPAASEEKKEKKAEKAAEE---EDVHDEL    508
tr|A0A8I3AHZ3|A0A8I3AHZ3_9PEZI     NGKYKAEASKKEEVVAEESQAAPAAT---EGEAAKES---DESHDEL    522
```

(overall sequence identity = 0.0563)

15. Peptidyl-prolyl cis-trans isomerases (Cyclophilins)

Peptidyl-prolyl disulfide isomerases (PPDIs, also called cyclophilins) accelerate the cis-trans isomerization of the proline peptide bond, thereby accelerating protein folding. They exist both intra- and extracellularly. Interestingly, while many PPDIs are secreted into the endoplasmic reticulum (ER), only a smaller portion of them possess KDEl-like signals. The rest are likely retained in the ER by indirect protein-protein interactions. The ER-localized PPDIs we found in kinetoplastids are more closely related to bacterial PPDI domains, even though the same PPDI lineage is also found in Dinoflagellates and their relatives. Therefore it is also possible that their KDEL signals evolved convergently, and are not immediately homologous to mammalian PPDIs.

```
tr|A0A7G2CH91|A0A7G2CH91_9TRYP     --------------MRLLTVVTFAALLLVSSTTVAGEDSYWVREMKRVILNAADDYMRYV    46
tr|A0A0M9G5W9|A0A0M9G5W9_LEPPY     -------MARSAV-----PTALVAALLLLLASAVSVQASYWSDEVTRVRTYAAQHYMDRT    48
tr|A0A0N1II12|A0A0N1II12_LEPSE     -----MKMARSAV-----LTVLVAT-LLLLASTISVQASYWSDEVTRVRTYSAQHYMDRT    49
```

```
tr|A4HA48|A4HA48_LEIBR             -------MNRFSTMRCVFAAVATLLLLLLTLCVVSVEASYWSNEVNRVRTYTAFNYMARM    53
tr|Q4QDB9|Q4QDB9_LEIMA             -------MARFSSLHSVLAAAATL--LLLALCVVSVEASYWSEEVNRVRTYAAVNYLERI    51
tr|A4HYB4|A4HYB4_LEIIN             -------MTRFSSLHSVLATAATL---LLTLCVLSVEASYWSEEVNRVRTYAAVNYLERI    50
tr|A0A3S5H764|A0A3S5H764_LEIDO     -------MTRFSSLHSVLATAATL---LLTLCVLSVEASYWSEEVNRVRTYAAVNYLERI    50
tr|Q4D932|Q4D932_TRYCC             -----MHRENYFSKMAFCLLGVLFLSCITSVQTVSGDAASHEERMNNYRKRVGRLFMEQK    55
sp|Q09734|MIP_TRYCR                -----MHRENYFSKIAFCLLGVLFLSCITSVQTVSGDAASHEERMNNYRKRVGRLFMEQK    55
tr|A0A3S5IR72|A0A3S5IR72_TRYRA     MQLRPTKTGKFYSRATLCLMAALMLLSMSVVLTVSGETLSSEVRLNNYRKRVGRAFIKEK    60
tr|A0A3R7NGG2|A0A3R7NGG2_9TRYP     -------------------MAALMLLSLSSAPTVSGETLSPEDRINNYRKRVGRAFIKEK    41
tr|A0A812UP16|A0A812UP16_SYMMI     --------------MK----ASVLSL----LLLF--YGAVAAADMNKYYKRTGKKFLDEK    36
tr|A0A7S1LN17|A0A7S1LN17_NEODS     --------------MRAIATAA-LVA----LLLL--AATAAAGDMTKYYKRTGAKFLSTK    39
tr|A0A250XD25|A0A250XD25_9CHLO     --------------MRIFLLAAAFLC----AILH---------HATAGSNEKGLAFLEEN    33
tr|A0A0M0JR19|A0A0M0JR19_9EUKA     --------------MRSTLLV---------ATLV---------GLVAASNEVGTKFLEEN    28
tr|A0A7S1FII8|A0A7S1FII8_NOCSC     --------------MLSTFALT--TL----VALC---------RVAVASNEFGLSFLEGN    31
tr|R1B618|R1B618_EMIHU             ---------------------MA--VL----AVTL---------FSLLASNDVGVKFLEEN    25
tr|R1D2M1|R1D2M1_EMIHU             ---------------------------------------------------VGVKFLEEN    9
                                                                                 .  ::

tr|A0A7G2CH91|A0A7G2CH91_9TRYP     ESWGNVTILPSGMGMNILQRGHGD-VAPGPNDSCLIRYTGMY-------RFPGVFEDTRQ    98
tr|A0A0M9G5W9|A0A0M9G5W9_LEPPY     AKDPNVSALPSGLLVTILRRGAGD-RAPAAEDVCEVHYTIHH-------RFPGIVDDTRD    100
tr|A0A0N1II12|A0A0N1II12_LEPSE     SKSPNVSALPSGMLVTVLGRGSGD-RAPAANDVCEIHYTIHH-------RYPGIVDSTRN    101
tr|A4HA48|A4HA48_LEIBR             AKQPNVSALPSGVLFTIERRGFGD-RAPAPEDKCEMHYTIHQ-------RFPDIVESTRH    105
tr|Q4QDB9|Q4QDB9_LEIMA             AKQPNVSALPSGLLFTIERRGFGD-RAPAAEDKCEMHYTIHY-------RFPGIVENTRH    103
tr|A4HYB4|A4HYB4_LEIIN             AKQPNVSALPSGLLFTIERRGFGD-RAPAAEDKCEMHYTIHY-------RFPGIVESTRH    102
tr|A0A3S5H764|A0A3S5H764_LEIDO     AKQPNVSALPSGLLFTIERRGFGD-RAPAAEDKCEMHYTIHY-------RFPGIVESTRH    102
tr|Q4D932|Q4D932_TRYCC             AAQPDAVKLPSGLVFQRIARGSGK-RAPAIDDKCEVHYTGRL-------RDGTVFDSSRE    107
sp|Q09734|MIP_TRYCR                AAQPDAVKLPSGLVFQRIARGSGK-RAPAIDDKCEVHYTGRL-------RDGTVFDSSRE    107
tr|A0A3S5IR72|A0A3S5IR72_TRYRA     AAQAGAVTLPSGLVFRRIARGSGK-RAAAADDECEVHYTGKL-------RDGTVFDSSRE    112
tr|A0A3R7NGG2|A0A3R7NGG2_9TRYP     SAQPDAVTLPSGLVFQRIARGSGK-RAAVDDECEVHYTGKL-------RDGSVFDSSRE    93
tr|A0A812UP16|A0A812UP16_SYMMI     AKEPNIITLKSGMLVEVLKQGPDDGKSPNKGDACEVTYKGTF-------KDGKGFDQ---    86
tr|A0A7S1LN17|A0A7S1LN17_NEODS     EAEEGVYKLPSGMLFKILEKGTGA-TSPRVADPCEVHYEGTL-------PNGNVFDSSFK    91
tr|A0A250XD25|A0A250XD25_9CHLO     KGKPGVITLPSGLQYKVLRQGEGA-YHPTADSSCDCHYKGTL-------IDGTVFDSSYD    85
tr|A0A0M0JR19|A0A0M0JR19_9EUKA     KSREGVITLPSGLQYKVLRSGDGD-SHPTPDSSCECHYEGRIAQKW---PSGETFDSSYK    84
tr|A0A7S1FII8|A0A7S1FII8_NOCSC     KNKEGVITLPSGLQYKVLRAGDGT-DHPTADSSCECHYEGRTAQSY---PDGTTFDSSYA    87
tr|R1B618|R1B618_EMIHU             AKKEGVISLPSGLQYKVLRAGDGD-SHPTASSSCECHYEGRTAQEYSKEPKGDTFDSSYA    84
tr|R1D2M1|R1D2M1_EMIHU             AKKEGVISLPSGLQYKVLRAGDGD-SHPTASSSCECHYEGRTAQEYSKEPKGDTFDSSYA    68
                                           .   * **:         * .      . *    *                  .:.

tr|A0A7G2CH91|A0A7G2CH91_9TRYP     YPFPVRRTPSQMIQGVGEALQYMREGDRWFLYVPYRLAYGREGNATLKIQPYMNLRYDLE    158
tr|A0A0M9G5W9|A0A0M9G5W9_LEPPY     KPYPVRRSPSQLLLGMAEAMQLMREGDRWFLYVPSKLAYGTEGWKERRVAGLANVRVDLE    160
tr|A0A0N1II12|A0A0N1II12_LEPSE     RPYPVRRSPSQLIPGMAEAMQLMREGDHWFLYVPSELAYGTKGWKERRVAGLSIVRVDLE    161
tr|A4HA48|A4HA48_LEIBR             NPYPVCRSPSQLIPGMAEAMQLMREGDRWHLYVPYQLAYGVEGCKEKKVAPMSSVRVEIE    165
tr|Q4QDB9|Q4QDB9_LEIMA             HPYPVRRSPSQLIPGMAEAMQLMREGDRWYLHVPYQLGYGYKEGCKEKKVPSLSNLRVEME    163
tr|A4HYB4|A4HYB4_LEIIN             HPYPVRRSPSQLIPGMAEAMQLMREGDRWYLHVPYQLGYGYKEGCKEKKVPSLSNLRVEIE    162
tr|A0A3S5H764|A0A3S5H764_LEIDO     HPYPVRRSPSQLIPGMAEAMQLMREGDRWYLHVPYQLGYGYKEGCKEKKVPSLSNLRVEIE    162
tr|Q4D932|Q4D932_TRYCC             RGKPTTFRPNEVIKGWTEALQLMREGDRWRLFIPYDLAYGVTGGG-GMIPPYSPLEFDVE    166
sp|Q09734|MIP_TRYCR                RGKPTTFRPNEVIKGWTEALQLMREGDRWRLFIPYDLAYGVTGGG-GMIPPYSPLEFDVE    166
tr|A0A3S5IR72|A0A3S5IR72_TRYRA     RGRPISFRPSGVIKGWAEALQLMREGDRWLLFIPHELGYGEAGAG-TSIPPFSPLEFDVE    171
tr|A0A3R7NGG2|A0A3R7NGG2_9TRYP     RGEPVSFRPRGVIKGWTEALQLMREGDRWRIFIPHELGYGAAGAG-PKIPPFSPLEFDVE    152
tr|A0A812UP16|A0A812UP16_SYMMI     --GTTSFAPNQVIKGWTEAMQFMGEGDKWKLYIPYDLAYGERGSP-PKIPPFTPLVFEIE    143
tr|A0A7S1LN17|A0A7S1LN17_NEODS     RGSPSSFAPNQVIKGWTEALQLMREGDKWEVYIPHELAYGARGAG-GVIPGYAALTFKMQ    150
tr|A0A250XD25|A0A250XD25_9CHLO     RGQPTAFAPNQVIKGWTEAMQLMVEGDKWEMYIPSDLGYGDGGSP-PKIGGGDTLIFQME    144
tr|A0A0M0JR19|A0A0M0JR19_9EUKA     RGSPTSFAPNQVIKGWTEAMQLMVEGDKWEMYIPSELGYGERGSP-PNIGGGDVLVFTME    143
tr|A0A7S1FII8|A0A7S1FII8_NOCSC     RGSPTSFAPNQVIKGWTEAMQLMVEGDKWEMYIPSELGYGDRGSP-PKIGGGDVLVFTME    146
tr|R1B618|R1B618_EMIHU             RGSPTSFAPNQVIKGWTEAMQLMVEGDKWEMYIPSELGYGDSGSP-PKIGGGDVLVFTME    143
tr|R1D2M1|R1D2M1_EMIHU             RGSPTSFAPNQVIKGWTEAMQLMVEGDKWEMYIPSELGYGDSGSP-PKIGGGDVLVFTME    127
                                            *   :: *   **:* * ***:* :.:*   *.**   *         :        :    ::

tr|A0A7G2CH91|A0A7G2CH91_9TRYP     VVSCDAPSGKKSAEID-AFLEPL--------LKR--PMPS-------------KT-----    189
tr|A0A0M9G5W9|A0A0M9G5W9_LEPPY     VLKCENPRGKTSEEID-AYLAPF--------LKT--PMPA-------------KS-----    191
tr|A0A0N1II12|A0A0N1II12_LEPSE     VFKCESPHGKTSEEID-AYLAPY--------LKT--PMPP-------------KS-----    192
tr|A4HA48|A4HA48_LEIBR             LYKCKSPSGKTSTEID-AYLAQF--------VKS--PMPV-------------KT-----    196
tr|Q4QDB9|Q4QDB9_LEIMA             LYKCESASGKTSAEID-AYLAKY--------MKT--RIPE-------------KA-----    194
tr|A4HYB4|A4HYB4_LEIIN             MYKCESASGKTSAEID-AYLAKY--------MKT--RIPE-------------KA-----    193
tr|A0A3S5H764|A0A3S5H764_LEIDO     LYKCESASGKTSAEID-AYLAKY--------MKT--RIPE-------------KA-----    193
tr|Q4D932|Q4D932_TRYCC             LISIKDGGKGRTAEEVDEILRKAEEDREDM------------------------------    196
sp|Q09734|MIP_TRYCR                LISIKDGGKGRTAEEVDEILRKAEEDREDM------------------------------    196
tr|A0A3S5IR72|A0A3S5IR72_TRYRA     LLRIKGGGTGRTAEEVDKILREVEGDRGDM------------------------------    201
tr|A0A3R7NGG2|A0A3R7NGG2_9TRYP     LIRIKGGGTGRTAEEVDKILREAEEERGDM------------------------------    182
tr|A0A812UP16|A0A812UP16_SYMMI     IHKVKSGGKPVSEARD--MLKNAIATGAEL------------------------------    171
tr|A0A7S1LN17|A0A7S1LN17_NEODS     LLKVKSGGKPAAEADA--AIQEKLGK---AYADL--------------------------    179
tr|A0A250XD25|A0A250XD25_9CHLO     LIKIKGGRVPALKCVP--STKTDCSEKEQAYIDKVSTLDTTGIAKELVRLKAMKSKPMTP    202
tr|A0A0M0JR19|A0A0M0JR19_9EUKA     ILKIQGKKVPANKCDV--KTYAGCSEKEETYIKSKVEMTAEALGAEIKRLGGMASQKMSS    201
tr|A0A7S1FII8|A0A7S1FII8_NOCSC     ILKIKGNKVPASRCDV--KSLEGCSDKEKEYITKQSANGKEKVESELNRLSGMKGGSMKD    204
tr|R1B618|R1B618_EMIHU             IIKIKGEKVPASKCDV--KSLDGCTEQEAKYASSKKSLDGDGIAAEMKRLQGIAGQKMGP    201
tr|R1D2M1|R1D2M1_EMIHU             IIKIK-------------------------------------------------------    132
                                   :   .

tr|A0A7G2CH91|A0A7G2CH91_9TRYP     PKIDWEKM-----------------    197
tr|A0A0M9G5W9|A0A0M9G5W9_LEPPY     MPINYADL-----------------    199
tr|A0A0N1II12|A0A0N1II12_LEPSE     VLAYDTDM-----------------    200
tr|A4HA48|A4HA48_LEIBR             EPLDYADL-----------------    204
tr|Q4QDB9|Q4QDB9_LEIMA             APVDYTDL-----------------    202
tr|A4HYB4|A4HYB4_LEIIN             APVDYTDL-----------------    201
tr|A0A3S5H764|A0A3S5H764_LEIDO     APVDYTDL-----------------    201
tr|Q4D932|Q4D932_TRYCC             -------------------------    196
sp|Q09734|MIP_TRYCR                -------------------------    196
tr|A0A3S5IR72|A0A3S5IR72_TRYRA     -------------------------    201
```

```
tr|A0A3R7NGG2|A0A3R7NGG2_9TRYP     -------------------------    182
tr|A0A812UP16|A0A812UP16_SYMMI     -------------------------    171
tr|A0A7S1LN17|A0A7S1LN17_NEODS     -------------------------    179
tr|A0A250XD25|A0A250XD25_9CHLO     ELLNWLERRIRILGLLDKTEL----    223
tr|A0A0M0JR19|A0A0M0JR19_9EUKA     TQSVWLSKRVGLLNKLKELKGKEEL    226
tr|A0A7S1FII8|A0A7S1FII8_NOCSC     DKLGWLNKRINLLQKLKDEL-----    224
tr|R1B618|R1B618_EMIHU             KQASWLAKRLSLLTKLKDEL-----    221
tr|R1D2M1|R1D2M1_EMIHU             -------------------------    132
```

(overall sequence identity = 0.0795)

ATG8 family group alignments (visualized on SFigure 12)

ATG8 (canonical)

```
CLUSTAL O(1.2.4) multiple sequence alignment


tr|Q4QD46|Q4QD46_LEIMA            MSSRVAGSYKKAHTLEARLRDAEKVRERAPDRILVICEKAENSPVPDLDKSKFLVPPDAT    60
tr|A4HYJ2|A4HYJ2_LEIIN            MSSRVAGSYKKAHTLEARLRDAEKVRERAPDRILVICEKAENSPVPDLDKSKFLVPPDAT    60
tr|A4HAB2|A4HAB2_LEIBR            MSSRVAGSYKKAHTLEARLRDAEKVRERAPDRILVICEKAENSPVPDLDKSKFLVPPDAT    60
tr|A0A0M9G613|A0A0M9G613_LEPPY    -MSKVAGSYKRTHTLSARRRDAEKVRERAPDRILVICEKVENSPVPDLDKSKFLVPPDAT    59
                                  *:******::***.** *******************.*******************

tr|Q4QD46|Q4QD46_LEIMA            VGGFLVSIRRRITMEAEKALFLFVGDSVPANSTLMSDLFNRYKDEDGFLYVTYSGENTYG    120
tr|A4HYJ2|A4HYJ2_LEIIN            VGGFLVSIRRRITMEAEKALFLFVGDSVPANSTLMSDLFNRYKDEDGFLYVTYSGENTYG    120
tr|A4HAB2|A4HAB2_LEIBR            VGGFLVSIRRRITMEAEKALFLFVGDSVPANSTLMSDLFNRYKDEDGFLYVTYSGENTYG    120
tr|A0A0M9G613|A0A0M9G613_LEPPY    VGGFLVSIRRRITMEAEKALFLFVADSVPANSTLMSDLYNTYKDEDGFLYVTYSGENTYG    119
                                  ***********************.*************:* ******************

tr|Q4QD46|Q4QD46_LEIMA            GQGLH    125
tr|A4HYJ2|A4HYJ2_LEIIN            WQGLH    125
tr|A4HAB2|A4HAB2_LEIBR            -----    120
tr|A0A0M9G613|A0A0M9G613_LEPPY    -----    119
```

(overall sequence identity = 0.8790)

ATG12

```
CLUSTAL O(1.2.4) multiple sequence alignment


tr|A0A0N0DZX5|A0A0N0DZX5_LEPPY    MPRHRYGGESSHTQRRGQHVSCSEAKRNGEAERSTSGDGSAATVPALQVVHSPAYHTASL    60
tr|A4HCG9|A4HCG9_LEIBR            ------------------------------------------------------------    0
tr|E9AH00|E9AH00_LEIIN            ------------------------------------------------------------    0
tr|Q4QBL7|Q4QBL7_LEIMA            ------------------------------------------------------------    0


tr|A0A0N0DZX5|A0A0N0DZX5_LEPPY    CSSSASSDAFVSPCDSEHGDGLVDESSLPCESTIAINSTGSKERQRSSREVRVGRQVSDR    120
tr|A4HCG9|A4HCG9_LEIBR            ------------------------------------------------------------    0
tr|E9AH00|E9AH00_LEIIN            ------------------------------------------------------------    0
tr|Q4QBL7|Q4QBL7_LEIMA            ------------------------------------------------------------    0


tr|A0A0N0DZX5|A0A0N0DZX5_LEPPY    HAQPEQRAFAAPTLPTASTIHATHNQEYPSRVSNPAASSSHAAASPSLSPPPPPRTHFQY    180
tr|A4HCG9|A4HCG9_LEIBR            ---------------------------------------MPAPPPPPLLPPPPRTYYQY    20
tr|E9AH00|E9AH00_LEIIN            ------------------------------------------MHAPPKPPPPPRTHYQY    17
tr|Q4QBL7|Q4QBL7_LEIMA            ------------------------------------------MHAPPQPPPPPRTHYQY    17
                                                                                   ******::**

tr|A0A0N0DZX5|A0A0N0DZX5_LEPPY    VHSFEYRCCLAAKIRALYGAESIPVIVEPAESHLRTPSPSAPPYESPRLAAETRIHIAGI    240
tr|A4HCG9|A4HCG9_LEIBR            MHSFECRCLLSSKIRRLYGAGTVPIIVEPTESYLRLSPPPASPHSKAKSSTGS----T-G    75
tr|E9AH00|E9AH00_LEIIN            MHSFECRCLLSKKMLRLYGAGTVPVIVEPTESHLRLSPSPPSLHGDSKSSAGS----A-G    72
tr|Q4QBL7|Q4QBL7_LEIMA            MHSFEHRCLLSKKMLRLYGASTVPVIVEPTESHLRLSPSPPSLHGESKSSAGS----A-G    72
                                  :**** ** *: *:  **** ::*:****:**:**        : . : :: :       :

tr|A0A0N0DZX5|A0A0N0DZX5_LEPPY    GQGLLARFRTQETSTRSSSAGTTTSPPLQASTASALASPSTRSTLKCVLPRSKTVAEVIL    300
tr|A4HCG9|A4HCG9_LEIBR            GAGGYARHRQLFTS------DGANASVTVSSTASAFTSPSTKSTLKCVLPCSKSVAEVIL    129
tr|E9AH00|E9AH00_LEIIN            SFGGYARNGQLSSS------GRSGAGVTASSAASAFTSPSAKSTLKCILPRSKSVAEVIL    126
tr|Q4QBL7|Q4QBL7_LEIMA            GFGGYARHGQLSSS------GRAGAGVTASSLASAFTSPSAKSTLKCILLRSKSVAEVIL    126
                                  . *  **  :*       . : :    :* ***::***::*****:*  **:******

tr|A0A0N0DZX5|A0A0N0DZX5_LEPPY    TLRDRLALDSCQSLFLSVGESDALVPGNSLLGDLYQRYHHRDGLLYLSYLLENTFG----    356
tr|A4HCG9|A4HCG9_LEIBR            ALRGRLALDSCQSLFLSVGENNVLVPGNSLLGDLYERYRNPDGFLYLGYLLENTFGGDVR    189
tr|E9AH00|E9AH00_LEIIN            TLRGRLALDSCQSLFLSVGENDVLVPGNSLLGDLYERYRNPDGFLYLGYLLENTFGGDVR    186
tr|Q4QBL7|Q4QBL7_LEIMA            TLRGRLALDSCQSLFLSVGENDVLVPGNSLLGDLYERYRNADGFLYLGYLLENTFGGDVR    186
                                  :**.***************.:.************:**:: **:***.********

tr|A0A0N0DZX5|A0A0N0DZX5_LEPPY    -----------------    356
tr|A4HCG9|A4HCG9_LEIBR            STAEEAAVIL-------    199
tr|E9AH00|E9AH00_LEIIN            SAAVEASVHTVR-----    198
tr|Q4QBL7|Q4QBL7_LEIMA            SAAGEAASHTVRRPVQR    203
```

(overall sequence identity = 0.2633)

ATG8C

```
CLUSTAL O(1.2.4) multiple sequence alignment


tr|A0A0M9FWM3|A0A0M9FWM3_LEPPY      MSAYVSSTRVEERIAKRASLNAGSCDVPVVVE--SAQGGKAHFSVLPRDATVAQLTSAVR    58
tr|A0A0M9FWE5|A0A0M9FWE5_LEPPY      MSAYVSSTRVEERIAKRASLNAGSCDVPVVVE--SAQGGKAHFSVLPRDATVAQLTSAVR    58
tr|A0A0N0VEA7|A0A0N0VEA7_LEPPY      MSAYVSSTRVEERIAKRASLNAGSCDVPVVVE--SAQGGKAHFSVLPRDATVAQLTSAVR    58
tr|A0A0N0DTH2|A0A0N0DTH2_LEPPY      MSAYVSSTRVEERIAKRASLNAGSCDVPVVVE--SAQGGKAHFSVLPRDATVAQLTSAVR    58
tr|A4H5J2|A4H5J2_LEIBR              MSAYVSSTPLEARVARCASLRATN-AVPVVVEEAQARGGKAHFSALARETTAAQLVAAVR    59
tr|A4H5J3|A4H5J3_LEIBR              MSAYVSSTPLEARVARCASLRATN-AVPVVVEEAQARGGKAHFSALARETTAAQLVASVR    59
tr|Q4QI11|Q4QI11_LEIMA              MSAYVLSTPLEARVAKCASLRAAN-AVPVVVEEAQARGGKAHFSALARETTVAQLVAAVR    59
tr|A0A381MCF0|A0A381MCF0_LEIIN      MSAYVLSTPLEARVAKCASLRAAN-AVPVVVEEAQARGGKAHFSALARETTVAQLVAAVR    59
tr|A0A381MAT2|A0A381MAT2_LEIIN      MSAYVLSTPLEARVAKCASLRAAN-AVPVVVEEAQARGGKAHFSALARETTVAQLVAAVR    59
tr|A0A381MBG2|A0A381MBG2_LEIIN      MSAYVLSTPLEARVAKCASLRAAN-AVPVVVEEAQARGGKAHFSALARETTVAQLVAAVR    59
tr|A4HTT6|A4HTT6_LEIIN              MSAYVLSTPLEARVAKCASLRAAN-AVPVVVEEAQARGGKAHFSALARETTVAQLVAAVR    59
tr|Q4QI09|Q4QI09_LEIMA              MSAYVLSTPLEARVAKCASLRAAN-AVPVVVEEAQARGGKAHFSALARETTVAQLVAAVR    59
tr|Q4QI15|Q4QI15_LEIMA              MSAYVLSTPLEARVAKCASLRAAN-AVPVVVEEAQARGGKAYFSALARETTVAQLVAAVR    59
tr|Q4QI18|Q4QI18_LEIMA              MSAYVLSTPLEARVAKCASLRAAN-AVPVVVEEAQARGGKAYFSALARETTVAQLVAAVR    59
tr|Q4QI08|Q4QI08_LEIMA              MSAYVLSTPLEARVAKCASLRAAN-AVPVVVEEAQARGGKAYFSALARETTVAQLVAAVR    59
tr|Q4QI19|Q4QI19_LEIMA              MSAYVLSTPLEARVAKCASLRAAN-AVPVVVEEAQARGGKAYFSALVRETTVAQLVAAVR    59
tr|Q4QI13|Q4QI13_LEIMA              MSAYVLSTPLEARVAKCASLRAAN-AVPVVVEEAQARGGKAYFSALVRETTVAQLVAAVR    59
                                    ***** ** :* *:*: ***.* .  ******  .*:****:**.* *::*.***.::**

tr|A0A0M9FWM3|A0A0M9FWM3_LEPPY      RLDGVDARKAVSLAVAQCAVAPTTTLGELHDACKQADDGMLYVTYTAEAEHGRCRVDA--    116
tr|A0A0M9FWE5|A0A0M9FWE5_LEPPY      RLDGVDARKAVSLAVAQCAVAPTTTLGELHDACKQADDGMLYVTYTAEAAMGATVNVCGM    118
tr|A0A0N0VEA7|A0A0N0VEA7_LEPPY      RLDGVDARKAVSLAVAQCAVAPTTTLGELHDACKQADDGMLYVTYTAEAAMGATVNVCGM    118
tr|A0A0N0DTH2|A0A0N0DTH2_LEPPY      RLDGVDARKAVSLAVAQCAVAPTTTLGELHDACKQADDGMLYVTYTAEAAMGACRTASPV    118
tr|A4H5J2|A4H5J2_LEIBR              AFRGVAANKPVTLTVAGCSVSPSATLGELHDACKQADDGMLYVAYTAERSMGAATWKPCG    119
tr|A4H5J3|A4H5J3_LEIBR              AFRGVAANKPVTLTVAGCSVSPSATLGELHDACKQADDGMLYVAYTAERSMGAATWKPCG    119
tr|Q4QI11|Q4QI11_LEIMA              GFRGVDAKKPVALTVAGCSVSPSATLGELHDACRQADDGMLYVAYTAERCMGAAVCTPCG    119
tr|A0A381MCF0|A0A381MCF0_LEIIN      GFRGVDAKKPVALTVAGCSVSPSATLGELHDACRRADDGMLYVAYTAERCMGAAVCTPCG    119
tr|A0A381MAT2|A0A381MAT2_LEIIN      GFRGVDAKKPVALTVAGCSVSPSATLGELHDACRRADDGMLYVAYTAERCMGAAVCTPCG    119
tr|A0A381MBG2|A0A381MBG2_LEIIN      GFRGVDAKKPVALTVAGCSVSPSATLGELHDACRRADDGMLYVAYTAERCMGAAVCTPCG    119
tr|A4HTT6|A4HTT6_LEIIN              GFRGVDAKKPVALTVAGCSVSPSATLGELHDACRRADDGMLYVAYTAERCMGAAVCTPCG    119
tr|Q4QI09|Q4QI09_LEIMA              GFRGVDAKKPVALTVAGCSVSPSATLGELHDACRQADDGMLYVAYTAERCMGAAVCMPCD    119
tr|Q4QI15|Q4QI15_LEIMA              GFRGVDAKKPVALTVAGCSVSPSATLGELHDACRQADDGMLYVAYTAERCMGAAVCMSCG    119
tr|Q4QI18|Q4QI18_LEIMA              GFRGVDAKKPVALTVAGCSVSPSATLGELHDACRQADDGMLYVAYTAERCMGAAVCTPCG    119
tr|Q4QI08|Q4QI08_LEIMA              GFRGVDAKKPVALTVAGCSVSPSATLGELHDACRQADDGMLYVAYTAERCMGAAVCTPCG    119
tr|Q4QI19|Q4QI19_LEIMA              GFRGVDAKKPVALTVAGCSVSPSATLGELHDACRQADDGMLYVAYTAERCMGAAVCTPCG    119
tr|Q4QI13|Q4QI13_LEIMA              GFRGVDAKKPVALTVAGCSVSPSATLGELHDTCRQADDGMLYVAYTAERCMGTAVCTPCG    119
                                    : ** *.* *:*:** *:*:*::*******:*::********:****    *

tr|A0A0M9FWM3|A0A0M9FWM3_LEPPY      -VLWRHLELSDETHASRVPSVP    137
tr|A0A0M9FWE5|A0A0M9FWE5_LEPPY      TAAW------------------    122
tr|A0A0N0VEA7|A0A0N0VEA7_LEPPY      TAANNC----------------    124
tr|A0A0N0DTH2|A0A0N0DTH2_LEPPY      VYP-------------------    121
tr|A4H5J2|A4H5J2_LEIBR              SCSWD-----------------    124
tr|A4H5J3|A4H5J3_LEIBR              SCSWD-----------------    124
tr|Q4QI11|Q4QI11_LEIMA              SCAWDGSSADDDVII-------    134
tr|A0A381MCF0|A0A381MCF0_LEIIN      SCAWN-----------------    124
tr|A0A381MAT2|A0A381MAT2_LEIIN      SCAWDGSTADDDVII-------    134
tr|A0A381MBG2|A0A381MBG2_LEIIN      SCACN-----------------    124
tr|A4HTT6|A4HTT6_LEIIN              SCA-------------------    122
tr|Q4QI09|Q4QI09_LEIMA              SCAWDGSSADDDVII-------    134
tr|Q4QI15|Q4QI15_LEIMA              SCALN-----------------    124
tr|Q4QI18|Q4QI18_LEIMA              SCAWDGSSADDDVII-------    134
tr|Q4QI08|Q4QI08_LEIMA              LCA-------------------    122
tr|Q4QI19|Q4QI19_LEIMA              SCSWN-----------------    124
tr|Q4QI13|Q4QI13_LEIMA              SCSWN-----------------    124
```

(overall sequence identity = 0.4681)

ATG8B

```
CLUSTAL O(1.2.4) multiple sequence alignment


tr|A0A0M9G5V4|A0A0M9G5V4_LEPPY      ---------------------------------------MASYRDAVSLEDRKLESQRLL    21
tr|A0A0M9G5H5|A0A0M9G5H5_LEPPY      ---------------------------------------MSAYQTNNSVEVRRAECARLQ    21
tr|A4HA43|A4HA43_LEIBR              ---------------------------------------MSAYQSSNTAEARRAECARLQ    21
tr|A4HA42|A4HA42_LEIBR              ---------------------------------------MSAYQSSNTAEARRAECARLQ    21
tr|Q4QDC8|Q4QDC8_LEIMA              ---------------------------------------MSAYHSSNPVEARRAECARLQ    21
tr|A4HYA4|A4HYA4_LEIIN              ---------------------------------------MCAYHSSNPVEARRAECARLQ    21
tr|A0A381MFR9|A0A381MFR9_LEIIN      ---------------------------------------MSAYHSSNPVEARRAECARLQ    21
tr|A4HYA5|A4HYA5_LEIIN              MTPLPCHSSPCLPPSLPLCAAPLHAAAHLPSHRTLSRPPMSAYHSSNPVEARRAECARLQ    60
tr|Q4QDD6|Q4QDD6_LEIMA              ---------------------------------------MSAYHSSNPVEARRAECARLQ    21
tr|Q4QDD3|Q4QDD3_LEIMA              ---------------------------------------MSAYHSSNPVEARRAECARLQ    21
tr|Q4QDC5|Q4QDC5_LEIMA              ---------------------------------------MSAYHSSNPVEARRAECARLQ    21
tr|Q4QDD0|Q4QDD0_LEIMA              ---------------------------------------MSAYHSSNPVEARRAECARLQ    21
                                                                               *.:*:    * *: *. **

tr|A0A0M9G5V4|A0A0M9G5V4_LEPPY      LKYPGHVAVVVKQVGK-ETKVHLLALPGDATVAELEATAAELLGVPGTKLALSVSGFTPA    80
tr|A0A0M9G5H5|A0A0M9G5H5_LEPPY      AKYPAHVAMVVEAAR--SSKAHFLALPRDATVAELEAAVRAALSISATKMSLAVDGCTPA    79
tr|A4HA43|A4HA43_LEIBR              AKYPGHVAMVVEAAK--SSKAHLLALPRDATVAELEAAVRQALGISAAKVTLAVEGCTPA    79
tr|A4HA42|A4HA42_LEIBR              AKYPGHVAMVVEAAK--SSKAHLLALPRDATVAELEAAVRQALGISAAKVTLAVEGCTPA    79
```

```
tr|Q4QDC8|Q4QDC8_LEIMA              AKYPGHVAVVVEAAEKAGSKVHFLALPRDATVAELEAAVRQALGTSAKKVTLAIEGSTPA    81
tr|A4HYA4|A4HYA4_LEIIN              AKYPGHVAVVVEAAEKAGSKAHFLALPRDATVAELEAAVRQALSTSVKKVTLAIEGSAPA    81
tr|A0A381MFR9|A0A381MFR9_LEIIN      AKYPGHVAVVVEAAEKAGSKAHFLALPRDATVAELEAAVRQALSTSVKKVTLAIEGSAPA    81
tr|A4HYA5|A4HYA5_LEIIN              AKYPGHVAVVVEAAEKAGSKAHFLALPRDATVAELEAAVRQALSTSVKKVTLAIEGSAPA    120
tr|Q4QDD6|Q4QDD6_LEIMA              AKYPGHAAVVVEAAEKAGSKVHFLALPRDATVAELEAAVRQALGTSAKEVTLAIEGSTPA    81
tr|Q4QDD3|Q4QDD3_LEIMA              AKYPGHAAVVVEAAEKAGSKVHFLALPRDATVAELEAAVRQALGTSAKKVTLAIEGSTPA    81
tr|Q4QDC5|Q4QDC5_LEIMA              AKYPGHVAVVVEAAEKAGSKVHFLALPRDATVAELEAAVRQALGTSAKKVTLAIEGSTPA    81
tr|Q4QDD0|Q4QDD0_LEIMA              AKYPGHAAVVVEAAEKAGSKVHFLALPRDATVAELEAAVRQALGTSAKEVTLAIEGSTPA    81
                                    ***.*.*:**: .    :*.*:**** *********:.   *.        :::*::.* :**

tr|A0A0M9G5V4|A0A0M9G5V4_LEPPY      SSTLLEDLYRHCKCADGFLYVRCGLVGTLGASAHIPCFGTSIIKEDFWWSPVPAKIEN    138
tr|A0A0M9G5H5|A0A0M9G5H5_LEPPY      AAATVGDLSDACKHADGFLYVAVRAEKAMGALA-VPCFASDTI---------------    121
tr|A4HA43|A4HA43_LEIBR              ATAAVGDVADACKHADGFLYVSVRGEQAMGIFS--LCFPFF-----------------    118
tr|A4HA42|A4HA42_LEIBR              ATASVGDVADACKHADGFLYVSVRGEQAMGIFS--LCFPFF-----------------    118
tr|Q4QDC8|Q4QDC8_LEIMA              VTATVGDIADACKRDDGFLYVSVRTEQAMGGIA-GLCFASDSGGI-------------    125
tr|A4HYA4|A4HYA4_LEIIN              VTAAVGDIADACKRDDGFLYVSVRTEQAMGGIA-GPCFASDSGGM-------------    125
tr|A0A381MFR9|A0A381MFR9_LEIIN      VTAAVGDIADACKRDDGFLYVSVRTEQAMGGIA-GLCFASDSGGM-------------    125
tr|A4HYA5|A4HYA5_LEIIN              VTAAVGDIADACKRDDGFLYVSVRTEQAMGAFA-SPCLSVA-----------------    160
tr|Q4QDD6|Q4QDD6_LEIMA              VTATVGDIADACKRDDGFLYVSVRTEQAMGAFA-SPCLSTY-----------------    121
tr|Q4QDD3|Q4QDD3_LEIMA              VTATVGDIADACKRDDGFLYVSVRTEQAMGAFA-SPCLSTY-----------------    121
tr|Q4QDC5|Q4QDC5_LEIMA              VTATVGDIADACKRDDGFLYVSVRTEQAMGAFA-SPCFSVA-----------------    121
tr|Q4QDD0|Q4QDD0_LEIMA              VTATVGDIADACKRDDGFLYVSVRTEQAMGAFA-SPCFSVA-----------------    121
                                    :: : *:   **  ******      ::*  :   *:
```

(overall sequence identity = 0.2542)

<u>ATG8A</u>

```
CLUSTAL O(1.2.4) multiple sequence alignment


tr|A0A0N0DXB5|A0A0N0DXB5_LEPPY      -MSYAKSVAVSVRTAECRRLQQEHPNEIPVVMEGPN-GRVHFLTVPHDATAATLEVAVRS    58
tr|A4HA39|A4HA39_LEIBR              MSVYRSLISADARRAECERVCREHPEQVPVVVESANSSHIRFLAMPRDAMVADLEATVRQ    60
tr|E9AGS4|E9AGS4_LEIIN              MSMYQSLIPADARRAECERVCREHPEQLPVVVESANSSHVRFLVVPRDATVADLEAEVRQ    60
tr|Q4QDD1|Q4QDD1_LEIMA              MSMYQSLIPADARRAECERVRREHPEQLPVVVESANSSHVRFLAVQRDATVADLEAEVRQ    60
tr|Q4QDD4|Q4QDD4_LEIMA              MSMYQSLIPADARRAECERVRREHPEQLPVVVESANSSHVRFLAVQRDATVADLEAEVRQ    60
                                             * . : ...* ***.*: :***:::***:*. * .:::**.: :** .* **. **.

tr|A0A0N0DXB5|A0A0N0DXB5_LEPPY      AAAITGKKLGIVVRGCSPAPSTAMADLYDACRCEDGFLYVAFRGEGAMGARNIYCVGNTG    118
tr|A4HA39|A4HA39_LEIBR              TLGATSKKVALAIEGCSPAAMTMVGDIFDAYKQPDGFLYVSCARESAMGAKDVCCVGDTG    120
tr|E9AGS4|E9AGS4_LEIIN              TLGTTNKKVALAIEGCSPAATTVVGDIFDACKQVDGFLYVSCAREPSMGAKDFCCFGNTG    120
tr|Q4QDD1|Q4QDD1_LEIMA              ALGRTNKKVALAIEGCSPAATTVMGDIFDACKQVDSFLHVSCARESSMGAKDLCCFGNTG    120
tr|Q4QDD4|Q4QDD4_LEIMA              ALGRTNKKVALAIEGCSPAATTVMGDIFDACKQVDGFLHVSCARESSMGAKDLCCFGNTG    120
                                    : . *.**:.:.:.*****  * :.*::** :  *.**:*:   * :***::. *.*:**

tr|A0A0N0DXB5|A0A0N0DXB5_LEPPY      RYLEDIENNPDLFGSYTA    136
tr|A4HA39|A4HA39_LEIBR              KFFEDIENNPNLLGSM--    136
tr|E9AGS4|E9AGS4_LEIIN              KYFADIENNPNLLGSL--    136
tr|Q4QDD1|Q4QDD1_LEIMA              KYFADIENNPDLLGSL--    136
tr|Q4QDD4|Q4QDD4_LEIMA              KYFADIENNPDLLGSL--    136
                                    ::: ******:*:**
```

(overall sequence identity = *0.4088*)
